# What Is in a Structure? Cell Permeability and Solubility of Series of Macrocycles and Linear Matched Pairs

**DOI:** 10.1021/acs.jmedchem.6c00830

**Published:** 2026-06-22

**Authors:** Mohit Tyagi, Vasanthanathan Poongavanam, Stefanie Zich, Marika Lindhagen, Ioannis Asproudis, Okky Dwichandra Putra, Jie Yang, Zackary J. R. Ashworth, Anna Guadagni, Luca J. Hagemeyer, Alessandro Oliva, Peter Sjö, Stefan Schiesser, Jan Kihlberg

**Affiliations:** † Department of Medicinal Chemistry, Discovery Sciences, 128698 R&D, AstraZeneca, Gothenburg, Mölndal 431 83, Sweden; ‡ Department of Chemistry for Life Sciences, Uppsala University, Box 576, Uppsala 751 23, Sweden; § Department of Chemistry and Molecular Biology, University of Gothenburg, Medicinaregatan 7b, Gothenburg 41390, Sweden; ∥ Early Product Development, Pharmaceutical Sciences, R&D, AstraZeneca, Gothenburg, Mölndal 431 83, Sweden; ⊥ Drugs for Neglected Diseases Initiative (DNDi), 15 Chemin Louis Dunant, Geneva 1202, Switzerland

## Abstract

Macrocycles are a highly interesting modality to modulate difficult-to-drug targets, but often reside in chemical space where obtaining sufficient cell permeability and solubility is challenging. We have determined permeability across Caco-2 cells, aqueous solubility and log *D* for four series of semipeptidic macrocycles and one series of linear matched molecular pairs. By using X-ray crystallography, NMR spectroscopy, and computational chemistry, unexpected permeability differences between series and matched pairs were explained by differences in conformational preferences that determine the formation of intramolecular interactions. Macrocycles that formed intramolecular NH–π interactions and hydrogen bonds were more permeable than matched pairs unable to form such interactions. The elevated permeability of linear compounds was concluded to result from their greater conformational flexibility, allowing them to shield amide bonds and expose nonpolar groups to a greater extent than their macrocyclic matched pairs. Solubility was less dependent on specific intramolecular interactions and was predominantly low at log *D* >2.5.

## Graphical abstract

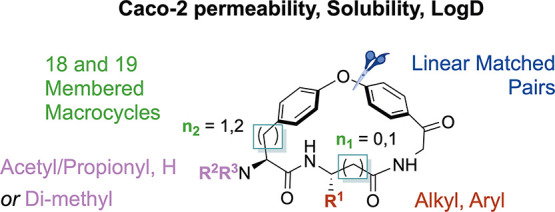

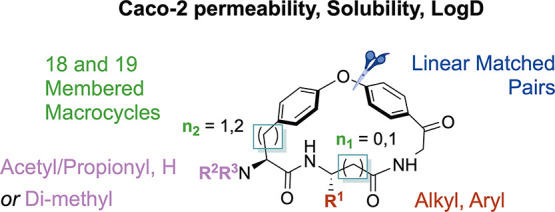

## Introduction

Many drug targets involved in human diseases cannot be effectively modulated with small molecules.^1–4^ This is often due to the binding sites of such difficult-to-drug targets being large and featureless. Macrocycles, defined as molecules that contain a ring of at least 12 atoms, offer advantages in this context because of their preorganized nature.^5–7^ This predisposes them to adopt spherical and disc-like conformations that allow modulation of tunnel- and groove-shaped as well as flat binding sites, in contrast to traditional, nonmacrocyclic drugs that frequently bind in pockets.^8^ The majority of macrocyclic drugs and clinical candidates are natural products or derivatives thereof, but de novo-designed macrocyclic drugs have begun to enter the market during the last two decades, illustrating our increasing ability to design macrocycles that modulate difficult-to-drug targets.^8^

To bind with sufficient potency to targets that have large and featureless binding sites, macrocycles are usually larger than drugs that comply with Lipinski’s rule of 5 (Ro5).^9^ Consequently, they often reside in the chemical space beyond the Ro5 (bRo5 space),^8,10^ where obtaining satisfactory solubility, cell permeability, and oral bioavailability is challenging.^11^ For example, FDA-approved orally absorbed macrocyclic drugs were found to have a median molecular weight (MW) and topological polar surface area (TPSA) of 830 Da and 201 Å^2^, respectively,^8^ far above the guidelines of the Ro5 ^9^ and Vebers rule^12^ (≤500 Da and ≤140 Å^2^, respectively). 2D descriptors, i.e., a hydrogen bond donor (HBD) count ≤7 in combination with either MW < 1000 Da or clog *P* > 2.5, identify the chemical space where most current oral macrocyclic drugs are found and may be used as filters early in design.^8^ However, the discovery of novel oral macrocycles within or beyond this space is far from trivial, and strategies for how to succeed remain to be developed.

As for other drug modalities, adequate cell permeability and aqueous solubility are essential for macrocycles to display oral bioavailability. Unfortunately, permeability and solubility data have only been reported for a small number of nonpeptidic macrocycles, often using different experimental protocols. For example, permeability across Caco-2 cell monolayers, the preferred cell model for oral absorption, has only been reported for 555 macrocycles according to the “membrane permeability database for nonpeptidic macrocycles”.^13^ Consequently, only limited and fragmented experimental data are available to build models that support property-based design of cell-permeable and orally bioavailable macrocyclic drugs.

In the absence of reliable models for prediction of cell permeability, mechanistic understanding and insight into the influence of different functional groups is valuable for property-based design of macrocycles. Determination of the permeability of 200 de novo-designed, natural product-inspired macrocycles across Caco-2 cells highlighted the importance of stereo- and regiochemistry, as well as the role of different functional groups, for cell permeability.^14^ Somewhat unexpectedly, tertiary amines were found to have a positive impact on permeability, while ureas and sulfonamides had a particularly large negative impact. For these 200 macrocycles, some stereoisomers behave as molecular chameleons that adjust their shape and properties to the environment by undergoing conformational changes.^15,16^ This behavior is considered to contribute to the ability of molecular chameleons to display both high aqueous solubility and high cell permeability, ideally providing them with improved oral bioavailability as compared to nonchameleons. A more recent study of 3600 Ro5-like macrocycles also pointed out the importance of the 3D conformation and of substructural elements for passive permeability across an artificial membrane.^17^ Even though knowledge about what structural features provide molecular chameleonicity is beginning to emerge, just as high-throughput assays for characterization of molecular chameleons, to date, few, if any, chameleonic drugs have been designed in a prospective manner; instead, their chameleonicity was elucidated retrospectively.^15^

Herein, we have designed, synthesized, and characterized 12 macrocycles and seven nonmacrocyclic controls to increase our understanding of how structural variations affect permeability across Caco-2 cells and aqueous solubility. The compounds expand our previous communication of how permeability is affected by the formation of a chameleonic intramolecular NH–π interaction between one side-chain and an amide bond located within the macrocyclic ring.^18^ In addition, we have now investigated how chameleonic intramolecular hydrogen bonding, expansion of the macrocyclic ring, and the replacement of an exocyclic acetylamino group by a dimethylamine affect permeability and solubility. Lastly, the properties of matched pairs of macrocycles and linear controls are compared. The systematic design and evaluation of the compounds provides a detailed mechanistic understanding of how structural alterations affect cell permeability and solubility, often by influencing intramolecular interactions which result in shifts in conformational ensembles.

## Results

### Design of Macrocycles

The first and main series of macrocycles investigated herein were inspired by the natural product hymenocardine^19^ and contain an 18-membered ring, which features a bisaryl-ether group cyclized via a peptidic backbone (Figure 1A). Two series have 19-membered macrocyclic rings, in which an additional methylene group has been inserted at different positions in the ring (Figure 1B). These three series have relatively rigid macrocyclic rings^20,21^ and were designed to explore how an NH–π interaction or an intramolecular hydrogen bond (IMHB) between the side-chain at the R^1^-position and the peptide backbone influences cell permeability and aqueous solubility.^18^ The 19-membered series provide additional insight into how a somewhat increased ring size and flexibility^21^ influences compound properties. In the fourth series, the exocyclic *N*-acyl amino group, i.e., the other substituent on the macrocyclic ring, was replaced by a dimethylamine, thereby introducing a basic group (Figure 1C). Lastly, the fifth series was designed to explore the effect of conversion of some of the macrocycles from the main series into fully flexible linear analogues (Figure 1D).

**Figure 1:**
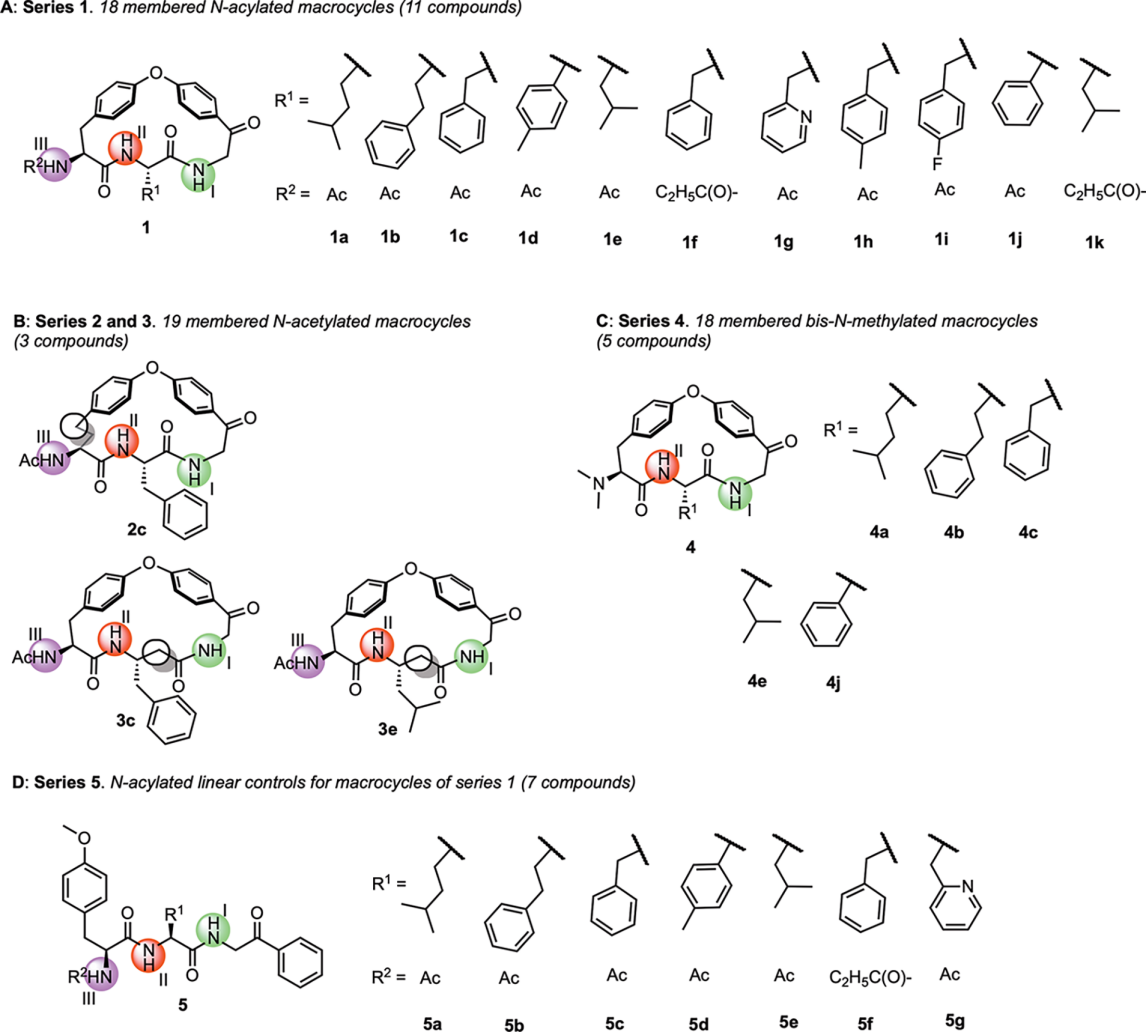
(A–C) Structures of the macrocycles belonging to series 1–4 and (D) of the linear analogues of series 5. Each compound is numbered by the series it belongs to, followed by a letter that describes the side chain at the R^1^-position, as detailed for series 1. Amide protons are labeled (NH-I, -II, and -III) and indicated by color. Macrocycles **1a**–**1d**, **1f**, **1g**, and **1j** have been reported previously,^18,20^ while all other compounds are novel.

The descriptors of Lipinskís^9^ and Vebeŕs^12^ rules were calculated to provide an indication of how the structural differences between the series and between matched molecular pairs in different series might influence properties such as lipophilicity and polarity. The Kieŕs flexibility index^22^ describes the flexibility of both macrocycles and nonmacrocyclic compounds and was calculated to allow a better comparison of compound flexibility than using the rotatable bond count (NRotB), which does not account for flexibility of cyclic compounds.^23^ Overall, the compounds in the five series complied with Lipinskís and Vebeŕs rules (Table S1). For instance, MWs ranged from 450 to 520 Da, clog *P*s from 1.9 to 3.4, and TPSAs from 88 to 126 Å^2^. Only the flexibility of the series of linear compounds (NRotB 11–13) exceeded the guideline of Vebeŕs rule (NRotB 10). Matched pair analysis suggested that series 4 compounds should be more lipophilic (clog *P* 0.4–0.45 units higher) and less polar (TPSA on average 28 Å^2^ lower) than the corresponding compounds in series 1, whereas matched pairs in series 5 and 1 had very similar lipophilicities (clog *P* difference ≤0.1 units) and identical TPSAs. As expected, the Kieŕs flexibility index, just as NRotB, predicted that the linear compounds of series 5 were substantially more flexible than their matched macrocyclic pairs in series 1.

### Synthesis of Macrocycles and Linear Controls

Macrocycles **1e**, **1h**, **1i**, **1k**, **2c**, **3c**, and **3e** were synthesized using the route developed previously by us for the synthesis of **1a**–**1d**, **1f**, **1g,** and **1j**.^18,20^ Thus, dipeptides **13e**, **13h**, **13i**, **14c**, **15c,** and **15e** were assembled in three steps from building block **8**, commercially available Boc protected amino acids (**6c**, **6e**, **6h**, **6i**, **7c**, and **7e**) and protected tyrosine or homotyrosine derivatives **11** and **12** using hexafluorophosphate azabenzotriazole tetramethyl uronium/*N*,*N*-diisopropylethylamine (HATU/DIPEA) as peptide coupling agents (Scheme 1). Cyclization of the tripeptides was achieved by a one-pot deprotection of the *tert*-butyldimethylsilyl (TBDMS) group and intramolecular cyclization, which was promoted by cesium fluoride to provide the required macrocycles **16e**, **16h**, **16i**, **17c**, **18c**, and **18e** in high yields (38–84%). The nitro group of these macrocycles was removed by reduction to the corresponding aniline using Pd–C/H_2_, followed by diazotization with NaNO_2_, Cu_2_O, and H_3_PO_2_. The hydroxyl group was then oxidized using 2-iodoxybenzoic acid (IBX), followed by purification of the product obtained after three synthetic steps using HPLC to provide **19e**, **19h**, **19i**, **20c**, **21c**, and **21e** in 7–43% yield. Subsequently, the Boc group was removed, after which the liberated primary amine was acylated to provide macrocycles **1e**, **1h**, **1i**, **1k**, **2c**, **3c**, and **3e**.

**Scheme 1:**
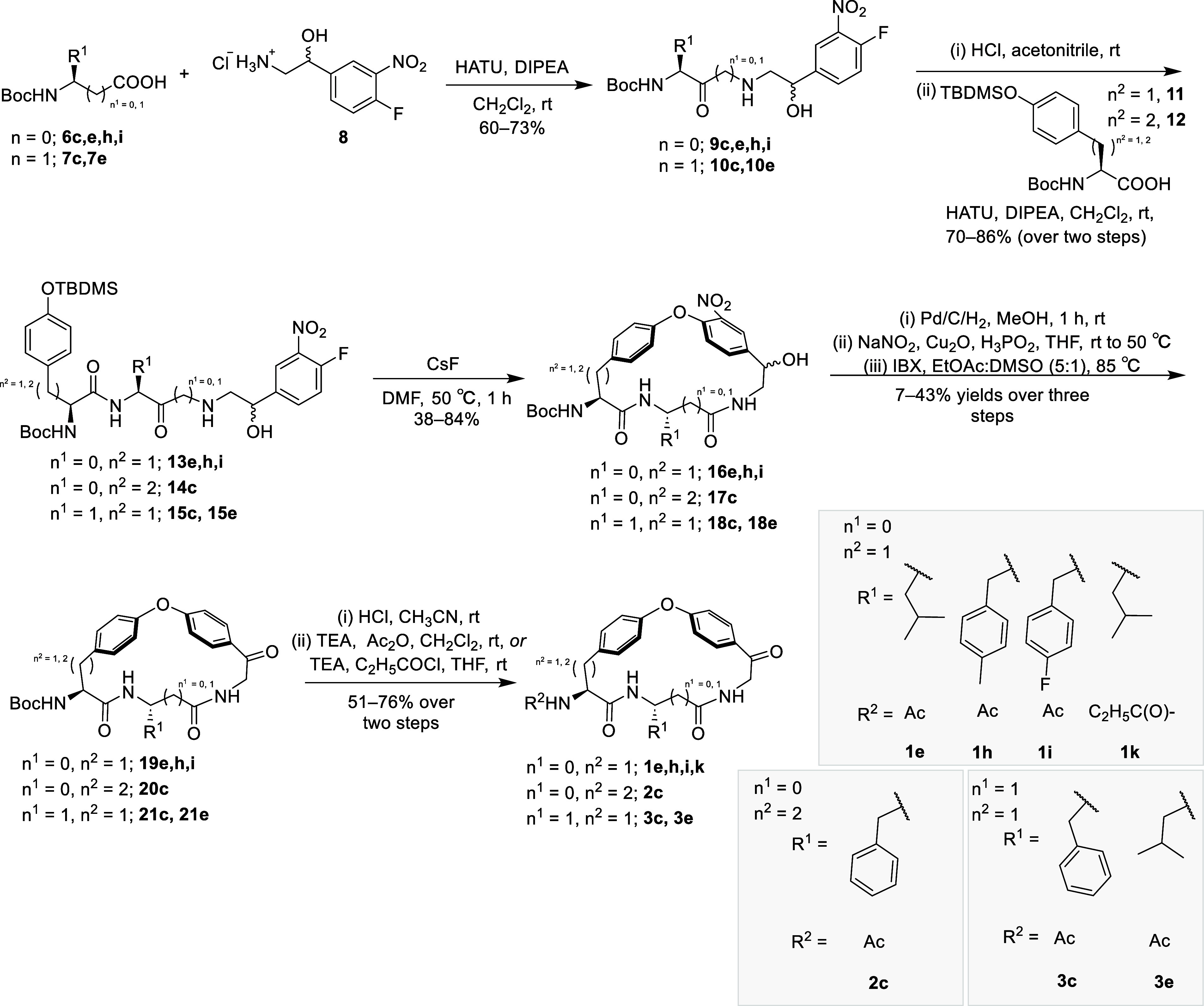
Synthesis of Macrocycles Belonging to Series 1, 2, and 3

Bis-methylated macrocycles were synthesized starting from Boc-protected **19a**,^18^ **19b**,^18^ **19c**,^18^ **19e,** and **19j** ^18^ (Scheme 2). The Boc group was cleaved by aq. HCl in acetonitrile, followed by bis-methylation of the amino group by reductive amination with HCHO in sodium citrate buffer using NaBH_3_CN in one pot to provide **4a**, **4b**, **4c**, **4e,** and **4j** in 29–67% yields after purification by HPLC (Scheme 2). Reductive amination at a controlled pH of 5–6 helped in keeping the ketone intact.

**Scheme 2:**
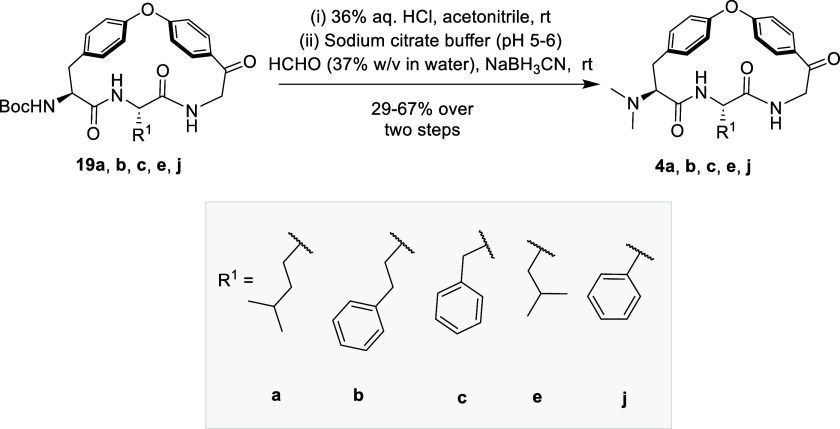
Synthesis of the Bis-*N*-Methylated Macrocycles of Series 4

Synthesis of linear analogues **5a**–**f** was initiated from commercially available Boc amino acids **6a**–**e** and building block **23** (Scheme 3A). Coupling of **6a**–**e** to **23** using HATU/DIPEA in a dichloromethane-DMF mixture provided **24a**–**e** (58–91%). Boc deprotection of **24a**–**e,** followed by coupling with commercially available **25,** then gave **26a**–**e** (52–90%), which were subjected to Boc deprotection, acetylation/propionylation, and oxidation by IBX to provide **5a**-**f** in 7–53% yields.

**Scheme 3:**
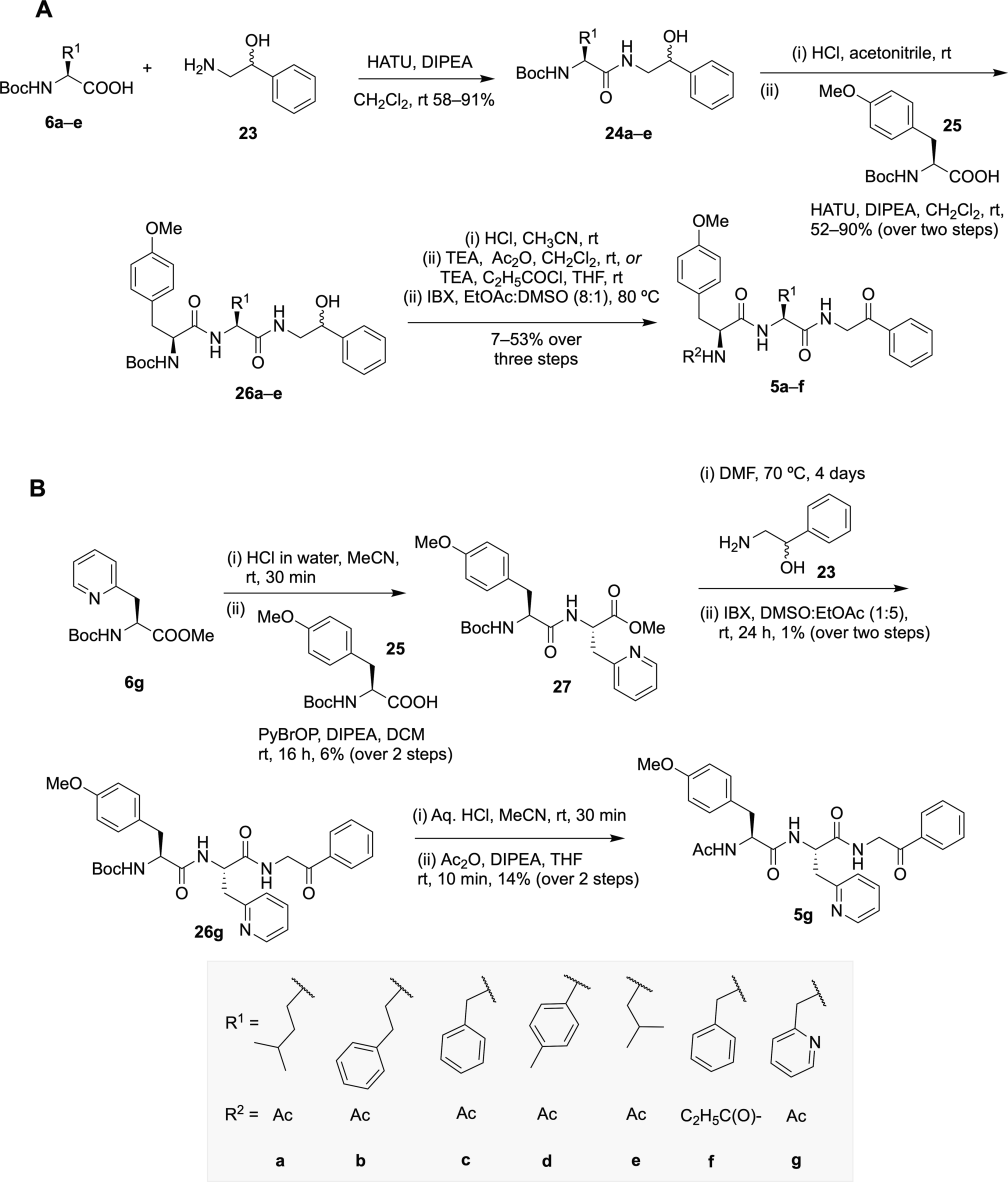
Synthesis of the Linear Analogues of Series 5

Surprisingly, following a similar synthetic strategy for linear analog **5g** proved challenging. While reacting the acid analog of compound **6g** with aminoalcohol **23** resulted in the desired dipeptide, the subsequent formation of the tripeptide using compound **25** gave complex reaction mixtures using various coupling reagents [e.g., hexafluorophosphate azabenzotriazole tetramethyl uronium (HATU), bromo-tris-pyrrolidino-phosphonium hexafluorophosphate (PyBroP), and benzotriazole-1-yl-oxy-tris-pyrrolidino-phosphonium hexafluorophosphate (PyBOP)]. Therefore, dipeptide **27** was first prepared by Boc-deprotection of **6g**, followed by coupling of **25** using PyBroP/DIPEA to provide **27** in 6% yield over two steps. Saponification of the methyl ester in compound **27,** followed by attempted amide bond formation to **23,** did not result in synthetically useful yields. However, we were pleased to find that direct reaction of **27** with amino alcohol **23** at 70 °C in DMF over 4 days gave the desired tripeptide, which was oxidized using IBX to give **26g** in 1% yield over two steps. Finally, the Boc group was cleaved, followed by acetylation to provide linear analogue **5g**.

### Side Chains and Macrocycle Ring Size

The *N*-acylated, 18- and 19-membered macrocycles had lipophilicities in the drug-like range (log *D*_7.4_ 1–3, Figure 2A). Overall, efflux-inhibited, passive permeabilities across Caco-2 cell monolayers^24^ increased proportionally to log *D*_7.4_, with four of the 14 macrocycles having low cell permeability (<1 × 10^–6^ cm/s) and four having high cell permeability (>5 × 10^–6^ cm/s). Closer inspection revealed that the macrocycles adhered to either of two correlations between permeability and lipophilicity (Figure 2A). In line with our previous finding for **1c**,^18^ macrocycles having a benzyl group at the R^1^-position (**1f** and **2c**), or a substituted version (**1h**), had a higher permeability at a specific log *D*_7.4_ than those having aliphatic side-chains or those having the phenyl group closer (**1d** and **1j**) or more distant (**1b**) from the macrocyclic ring.^18^ For **1c,** this increase was concluded to originate from the shielding of the polarity of the NH-I amide proton in the macrocyclic ring via the formation of an intramolecular NH–π interaction with the phenyl ring in a nonpolar but not in a polar environment,^18,20^ i.e., from a chameleonic behavior.^15^ The permeability increases for **1f**, **1h,** and **2c**, all of which can form an intramolecular NH–π interaction, provide further evidence for the contribution of such a chameleonic interaction to increased cell permeability. Macrocycle (**1i**), which has a *p*-F-benzyl group at the R^1^-position, stands apart from the other members of this subseries, most likely due to the weakening of the NH–π interaction, as indicated by the chemical shift of NH-I (cf. Table 1). Macrocycle **1g**, the 2-pyridyl analogue of **1c**, also adheres to the higher permeability–lipophilicity correlation due to the formation of an environment-dependent IMHB between the pyridyl nitrogen atom and the NH-I amide proton. This chameleonic IMHB was predicted by conformational sampling and MD simulations,^20^ observed by NMR spectroscopy in CDCl_3_ but not in DMSO (cf. Table 1, below) and formed upon minimization of a crystal structure of **1g** (Figures 3A and S1). Compound **1j** stands out as an outlier that has an unexpectedly low cell permeability among the set of macrocycles that are unable to form an intramolecular interaction with NH-I (Figure 2A). In order to investigate if the low permeability originates from a conformational change, we determined the structure of **1j** by X-ray diffraction analysis of a single crystal obtained in chlorobenzene. Comparison of the structure of **1j** (Figure S2) with that of **1c** ^18^ revealed that the macrocyclic ring of both compounds adopts very similar conformations (Figure S11), ruling out a major conformational change as the reason for the surprisingly low permeability of **1j**. In conclusion, the shielding of NH-I by the formation of chameleonic intramolecular interactions with the R^1^ side chain led to a permeability increase that, on average, amounted to 0.55 logarithmic units, i.e., a 3.5-fold increase for some of the 18-membered macrocycles of series 1.

**Figure 2:**
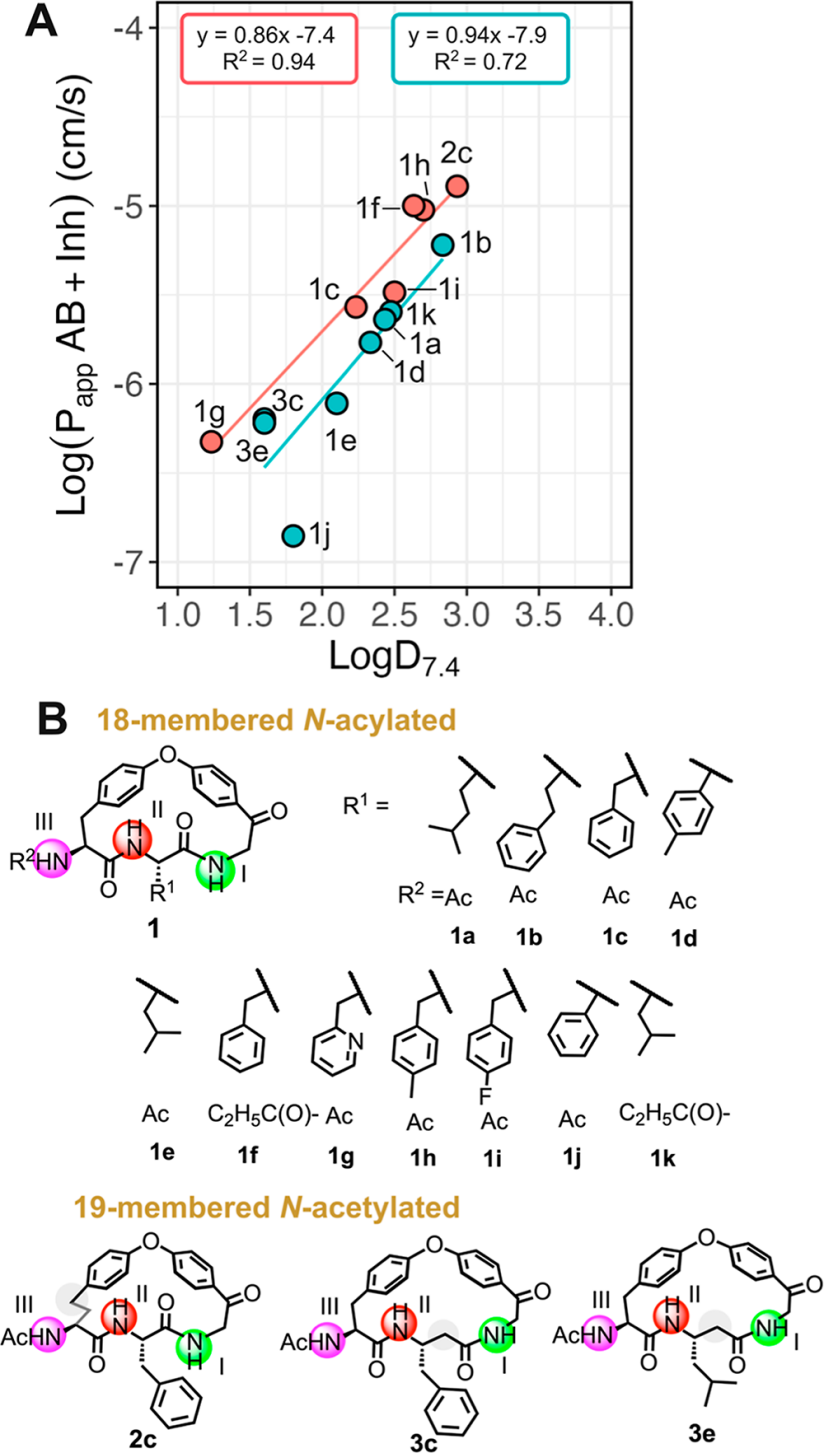
(A) Efflux-inhibited, passive cell permeability across a Caco-2 cell monolayer [log­(*P*_app_ AB + Inh)] as a function of lipophilicity (log *D*_7.4_, determined by the shake-flask method) for the series of 18-membered macrocycles and three 19-membered matched pairs. Correlations between log­(*P*_app_ AB + Inh) and log *D*_7.4_ have been derived for macrocycles, for which NH-I can form an NH–π interaction or an IMHB with the R^1^ side chain (in red), and for those macrocycles in which such interactions cannot be formed (in cyan). The plotted mean values were obtained from three to six repeats for log­(*P*_app_ AB + Inh) and from three or four repeats for log *D*_7.4_. (B) Structures of the macrocycles included in the correlations in panel A.

**Table 1:**
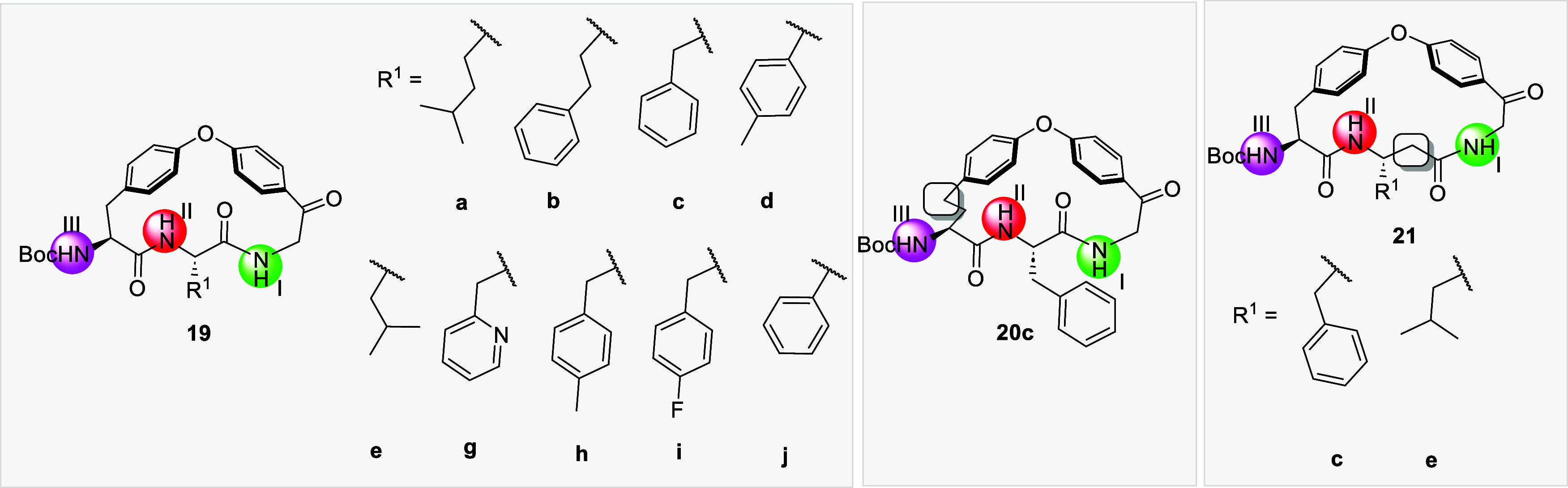
Chemical Shifts of Amide N*H*’s in Macrocycle Series **19**, **20,** and **21** ^a^

	CDCl_3_			DMSO-*d*_6_		
macrocycle	NH (I)	NH (II)	NH (III)	NH (I)	NH (II)	NH (III)
**19a**	6.22	6.28	5.10	8.50	6.42	7.32
**19b**	5.64	6.27	5.03	8.56	6.63	7.32
**19c**	***4.88***	***6.28***	***5.05***	8.31	6.63	7.20
**19d**	5.90	6.87	4.97	8.75	7.24	7.25
**19e**	5.67	6.23	4.99	8.57	6.49	7.40
**19g**	***8.15***	***6.43***	***5.06***	8.32	6.88	7.17
**19h**	***4.74***	***6.24***	***5.03***	8.32	6.62	7.23
**19i**	5.16	6.23	5.02	8.36	6.69	7.22
**19j**	5.91	6.89	4.96	8.79	7.31	7.27
**20c**	5.28	7.11	4.61	8.70	7.35	7.13
**21c**	5.45	6.42	5.11	8.10	7.13	7.54
**21e**	5.41	6.72	4.98	8.20	7.08	7.55

a Spectra were recorded at 400 or 500 MHz and 298 K. In CDCl_3_ NH (I) of **19c**, **19g** and **19h** displays a large up- or downfield shift.This is indicated using boldface italics for the shifts of the three NH-protons for these three compounds.

**Figure 3:**
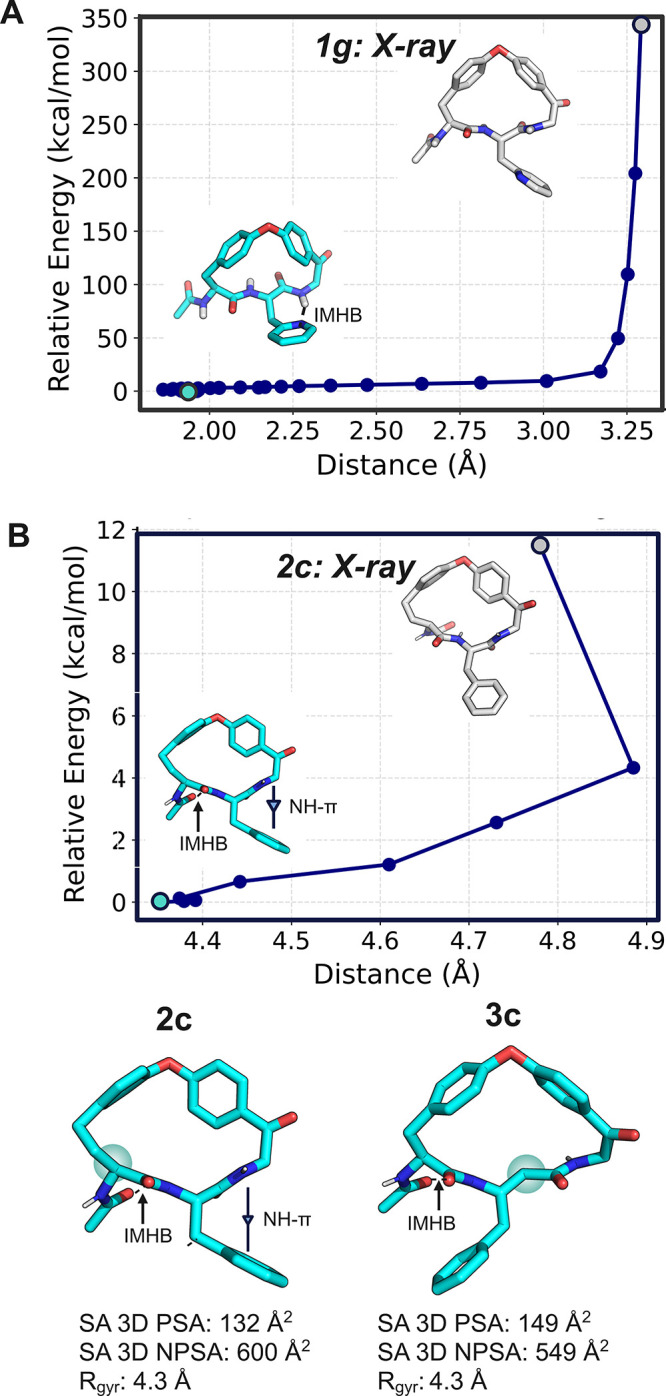
(A) Energy profile for the minimization of the crystal structures of **1g** and **2c** with B3LYP in implicit chloroform (ε = 4.8). In both panels, the crystal structure is shown at the top right and the optimized conformation at the bottom left. For **1g**, the *x*-axis shows the distance between NH-I and the pyridyl nitrogen atom in the R^1^ side chain during the optimization, while the distance between NH-I and the center of the phenyl ring is shown for **2c**. (B) Comparison of the conformations of the 19-membered **2c** and **3c** obtained after energy minimization with B3LYP in implicit chloroform (ε = 4.8). The structure of **3c** was obtained by replacement of the Boc group in the crystal structure of **21c** by an acetyl group, followed by energy minimization. The cyan sphere in each structure indicates the location of the methylene group inserted in the macrocyclic ring, as compared to the parent, 18-membered **1c**.

Three matched molecular pairs illustrate the strong correlation between passive cell permeability and lipophilicity for these macrocycles. Matched pairs **1f**/**1h** and **1k**/**1a** differ by the shift of a methylene group from the *N*-propionyl group of **1f** and **1k** to the R^1^ side-chain in **1h** and **1a**, respectively (Figure 2B). The members of each of these two pairs have very similar lipophilicities and also similar passive permeabilities. Since **1f**/**1h** both have a benzyl side-chain, this matched pair resides on the elevated lipophilicity–permeability correlation. 19-Membered macrocycles **3c** and **3e** also have almost identical lipophilicities and permeabilities, revealing that insertion of the methylene group in the macrocyclic ring prevents **3c** from forming an intramolecular NH–π interaction. This conclusion is also supported by the chemical shift of NH-I in the Boc-protected analogues of **3c** and **3e** (cf. Table 1). Interestingly, matched pairs **2c** and **3c**, which differ only by the position of the methylene group inserted in the macrocyclic ring, have lipophilicities that differ by 1.4 log units and permeabilities that differ by more than 1 order of magnitude. To some extent, these differences most likely originate from the inability of the R^1^ phenyl ring of **3c** to shield the NH-I amide proton via an intramolecular NH–π interaction, while shielding takes place in **2c** (cf. the crystal structure section, below).

The ^1^H NMR chemical shift of the NH-I proton in the Boc-protected precursors of the series 1 and 2 macrocycles supports the formation of the intramolecular NH–π and hydrogen bonds discussed above (Table 1). Because of the limited solubility of some of the *N*-acylated macrocycles in chloroform, the NMR studies were performed on their Boc-protected precursors. In chloroform, 18-membered macrocycles **19c** and **19h**, which have a benzyl or *p*-methyl benzyl group at the R^1^-position, respectively, display a substantial shielding of the NH-I proton as compared to **19a**, **19b,** and **19e** (appr. one ppm, Table 1), in which no or only a weak intramolecular NH–π interaction can be formed. Compound **19i**, which has a fluorine atom on the R^1^-benzyl group, shows a weaker shielding of the NH-I proton (appr. 0.7 ppm), indicating a weaker NH–π interaction in agreement with the reduced permeability elevation of **1i** (Figure 2). The large deshielding of NH-I of **19g** (2.3 ppm) reveals the formation of a strong IMHB between NH-I and the pyridyl moiety in chloroform. The chemical shifts of the two other amide-type protons (NH-II and NH-III) showed only a limited variation between the 18-membered macrocycles, revealing NH-II and NH-III to be in similar chemical environments in chloroform. This was also the case for all three amide-type protons in DMSO-*d*_6_, which illustrated that the intramolecular interactions between the R^1^ side chain and NH-I observed for four of the 18-membered macrocycles in chloroform were disrupted in a polar environment.

In chloroform, ring expansion to give 19-membered macrocycle **20c** led to the chemical shift of all three amide NHs differing from the shifts of the corresponding NHs in the 18-membered reference macrocycles **19a**, **19b,** and **19e**, i.e., NH-I and NH-III in **20c** both display a modest shielding while NH-II shows a large deshielding (Table 1). The calculated minimum energy conformation^21^ of a substituted derivative of **20c** clearly suggested that these shift differences originate from a substantial difference in the conformations populated by the macrocyclic ring in the 19-membered **20c** as compared to in the series of 18-membered macrocycles. This is also supported by the conformation adopted by **2c** in the crystalline state (cf. below). For **20c**, the large deshielding observed for NH-II most likely originates from the formation of an intramolecular hydrogen bond with the carbonyl oxygen atom of the adjacent Boc group. Macrocycles **21c** and **21e** constitute a matched pair of 19-membered macrocycles that have benzyl and isobutyl side chains at the R^1^ position, respectively (Table 1). The chemical shifts of the three amide NH protons are very similar between **21c** and **21e**, both in chloroform and in DMSO. This confirms the lack of an intramolecular NH–π interaction between the R^1^ benzyl group of **21c** and any of the NH protons, as discussed above for the lipophilicity and permeability of **3c** and **3e**. However, the deshielding of NH-II, as compared to NH-I and NH-III, suggests that NH-II in **21c** and **21e** forms an intramolecular hydrogen bond with the adjacent Boc group, just as in **20c**.

The reported crystal structure of macrocycle **1c**,^18^ and the novel crystal structures of macrocycles **1g**, **2c,** and **21c** reported herein (Figures 3, S1–S11 and Table S2), provide high-resolution structural information that contributes to rationalizing the structure–permeability relationships displayed by macrocycles from series 1–3 (Figure 2). The crystal structures of 18-membered **1g** and the two 19-membered macrocycles **2c** and **21c** were unequivocally determined by single-crystal X-ray diffraction analysis. Single crystals of **1g**, **2c,** and **21c** were obtained via slow solvent evaporation from trichloroethane, chlorobenzene, and acetonitrile, respectively. Notably, the crystal structure of **2c** is a chlorobenzene monosolvate, whereas **1g** and **21c** crystallized in the nonsolvated form. The hydrogen bond donors and acceptors in the amide backbone of these three macrocycles, and in the backbone of **1j** (cf. above), formed intermolecular hydrogen bonds with adjacent molecules in the crystal (Figures S6–S9). Thus, the conformations adopted in the crystal are likely to resemble those in an aqueous environment where the amide bonds of the backbone are solvated. In order to get insight into the conformations in a nonpolar environment, such as the interior of a cell membrane, the geometries of the structures of **1g** and **2c** were optimized to reduce the influence of crystal packing, as reported previously for **1c**.^18^ The Boc group in the structure of **21c** was replaced by an acetyl group before optimization to provide structural information for **3c**. Optimization was performed at the density functional theory (DFT) level in implicit chloroform (ε = 4.8), as it has a similar dielectric constant to that determined^25^ for the interior of a cell membrane (ε = 3.0).

Analogous to **1c**,^18^ the optimized crystal structures of **1g** and **2c** revealed the formation of an IMHB or an NH–π interaction to NH-I, respectively (Figure 3A). For **2c,** an intramolecular hydrogen bond between the *N*-acetyl group and NH-II was also formed in the optimization. The larger decrease in energy in the optimization of **1g**, as compared to **2c**, most likely originates from the fact that **1g** undergoes a larger conformational change than **2c** when the pyridyl group rotates to form the IMHB. The greater strength of an IMHB as compared to an NH–π interaction is also likely to contribute. The optimized structures of **1g** and **2c**, just as the reported optimized structure of **1c**,^18^ support that intramolecular interactions to NH-I contribute to the elevation of the cell permeability for some of the macrocycles from series 1, 2, and 4.

Isosteric, 19-membered macrocycles **2c** and **3c** differ only by the location of the methylene group that has been introduced in the macrocyclic ring as compared to in the 18-membered **1c** (Figure 3B), but both log *D*_7.4_ and the cell permeability of **2c** are more than 1 order of magnitude higher than for **3c**. While the geometry-optimized structures of **2c** and **3c** both revealed an IMHB between NH-II and the adjacent *N*-acetyl group, only macrocycle **2c** displayed an intramolecular NH–π interaction to NH-I (Figure 3B). For **3c**, the added methylene group prevented the formation of such an interaction, leaving the amide bond to NH-I exposed. In addition, this amide bond was rotated 180° in the optimized structure of **3c** as compared to in **2c**, as a result of the difference in location of the methylene group introduced in the two macrocycles. These structural differences resulted in a substantially lower solvent-accessible 3D polar surface area (SA 3D PSA) and higher solvent-accessible 3D nonpolar surface area (SA 3D NPSA) for the minimum energy conformation of **2c** as compared to **3c**, while their shapes were identical as revealed by their radius of gyration (*R*_gyr_) (Figure 3B). As indicated by the two 3D PSA descriptors **3c** can be expected to pay a higher desolvation penalty than **2c** when transitioning from the extracellular aqueous environment into the cell membrane, rationalizing the lower cell permeability of **3c**.

### *N*-Dimethyl Versus *N*-Acetyl Amine Side Chains

An investigation of the impact of different substituents on the permeability of a library of natural-product derived macrocycles found tertiary amines to be associated with enhanced cell permeability.^14^ Here, we investigate the effect of replacing an exocyclic *N*-acetylamino group with a dimethylamino group on the physicochemical properties and cell permeability of a subset of the 18-membered series 1 macrocycles (Figure 4). On average, the introduction of a dimethylamino group led to a log *D*_7.4_ increase by 0.54 units for the five matched pairs of macrocycles (Figure 4A); a value that agrees well with a clog *P* difference of approximately 0.4 units (Table S1). Just as for the *N*-acetylamino series, passive cell permeability across Caco-2 cells increased linearly with increasing log *D*_7.4_ for the members of the dimethylamine series. As revealed by the separate log *D*-permeability correlations for the two series, the dimethylamino series also showed an overall increase in cell permeability ranging from 0.65 to 1.4 logarithmic units between matched pairs, i.e., a 5–24-fold increase in permeability as compared to the corresponding macrocycles of the *N*-acetylamino series.

**Figure 4:**
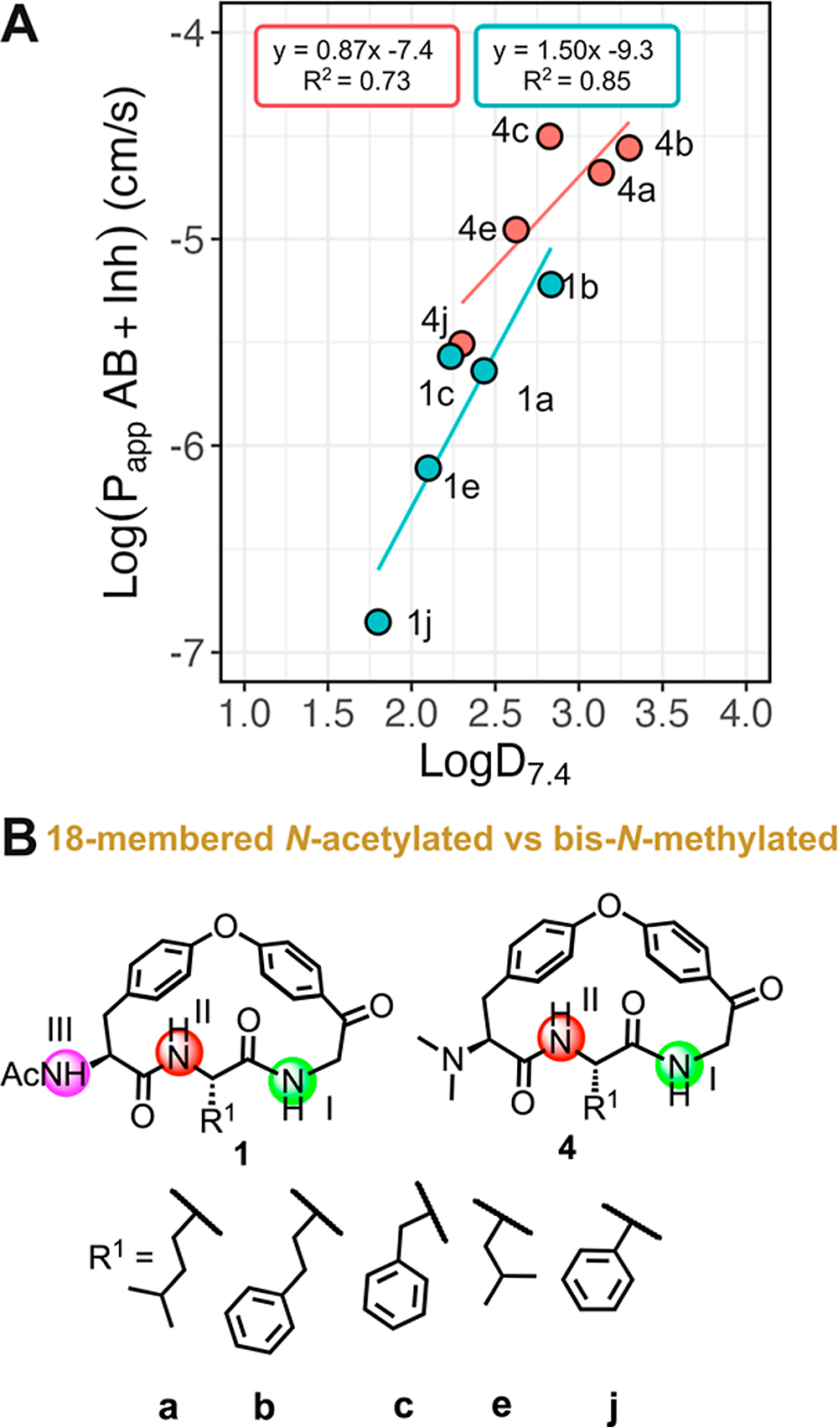
(A) Correlations between efflux-inhibited, passive cell permeability across a Caco-2 cell monolayer [log­(*P*_app_ AB + Inh)] and lipophilicity (log *D*_7.4_, determined by the shake-flask method) for five 18-membered, *N*-acetylated macrocycles (in cyan) and a series of dimethylamine matched molecular pairs (in red). The plotted mean values were obtained from three to six repeats for log­(*P*_app_ AB + Inh) and from three or four repeats for log *D*_7.4_. (B) Structures of the macrocycles included in the correlations in panel A.

The increases in log *D*_7.4_ and cell permeability obtained on replacement of the *N*-acetylamino with a dimethylamino group can be rationalized after consideration of the charge and polarity of the two groups. Caco-2 cell permeability was determined with a pH of 6.5 on the apical and 7.4 on the basolateral side of the cell monolayer. At pH 6.5, the dimethylamino group of the series 4 macrocycles is protonated to an extent of approximately 50–70% (Table 2), leaving 30–50% of the uncharged form available for passive membrane permeation. The finding that the dimethylamino series still displays a higher permeability than the *N*-acetylamino series is an illustrative example of the high polarity of secondary amides.^26^ While an uncharged dimethylamine only serves as a hydrogen bond acceptor, the amide bond is highly polar, with the oxygen atom being a strong hydrogen bond acceptor and the N–H hydrogen atom a strong donor.^26^ Consequently, the desolvation energy for transfer of the dimethylamines from an aqueous environment into the interior of a cell membrane is expected to be lower than for the corresponding *N*-acetylamines, rationalizing the higher permeability of the dimethylamino series. The increased permeability of macrocycles in which a secondary amide has been replaced by a tertiary amine, in combination with the fact that higher solubilities are more likely for partially charged tertiary amines (cf. the section on solubility, below), could result in a higher oral bioavailability for the tertiary amines.

**Table 2:**
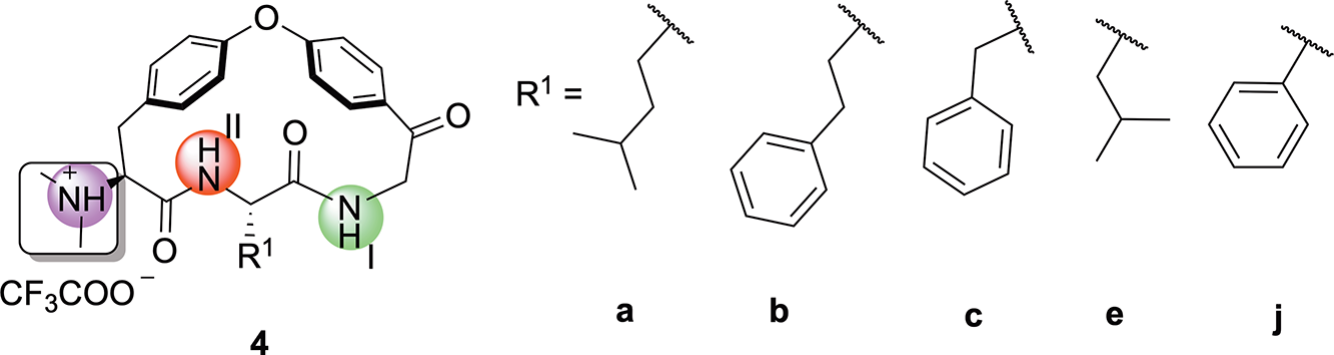
Basicity and ^1^H NMR Chemical Shifts of Amide N*H*s for the Macrocycles of Series 4

				CDCl_3_ ^a^		DMSO-*d*_6_ ^a^	
macrocycle	p*K*_a_ ^b^	% charged at pH 6.5	% charged at pH 7.4	NH (I)	NH (II)	NH (I)	NH (II)
**4a**	6.76	64	19	5.59	6.87	8.55	8.21
**4b**	6.77	64	19	5.41	6.96	8.63	8.31
**4c**	6.65	59	15	***4.71***	***6.92***	8.24	8.34
**4e**	6.54	52	12	5.54	6.98	8.62	8.29
**4j**	6.84	69	22	5.60	7.32	8.75	8.97

a NMR spectra were recorded at 400 or 500 MHz and at 298 K after protonation of the tertiary amine with TFA. In CDCl_3_ the chemical shift of NH (I) displays a large upfield shift, indicated by the use of boldface italics for both NH protons.

b Measured by potentiometry. Standard deviation ≤0.08 p*K*_a_ units based on three repeats.

Macrocycle **4c** showed an elevated permeability as compared to the other four members of the dimethylamino series (Figure 4A). Just as for its matched pair in series 1, i.e., **1c**, this can be understood from the formation of an intramolecular NH–π interaction between the phenyl ring of the R^1^ side-chain and the amide NH-I in the macrocyclic ring. The formation of such an intramolecular NH–π interaction is supported by the shielding of NH-I of **4c** in chloroform (Table 2), in analogy with the shielding observed for NH-I of **19c** and **19h** (Table 1).

### Linear Versus Macrocyclic Compounds

Compounds **5a**–**5g**, which are linear analogues of macrocycles **1a**–**1g**, were prepared to explore the impact of macrocyclization on the Caco-2 cell permeability of the series of 18-membered macrocycles (Figure 5). Disconnection of the macrocyclic ring was done at the bisaryl ether moiety to keep the connectivity of the peptidic backbone intact, while the resulting phenolic oxygen atom was methylated to avoid the addition of a hydrogen bond donor to the linear analogues. All linear analogues have higher log *D*_7.4_ values and also higher passive cell permeabilities compared to their corresponding macrocycles (Figure 5A). As mentioned above, the lipophilicity of the compounds in each matched pair from series 1 and 5 was predicted to be almost identical (Δclog *P* < 0.1, Table S1). For these two series, predicted lipophilicities agreed better with the experimental data for the compounds in the linear series than for those of the macrocyclic series. No increase in permeability resulting from an intramolecular NH–π interaction was observed for the linear analogues **5c** and **5f**, unlike for the macrocyclic parents **1c** and **1f**. Similarly, the permeability of linear **5g** did not appear to be influenced by the formation of an IMHB, in contrast to macrocyclic **1g**.

**Figure 5:**
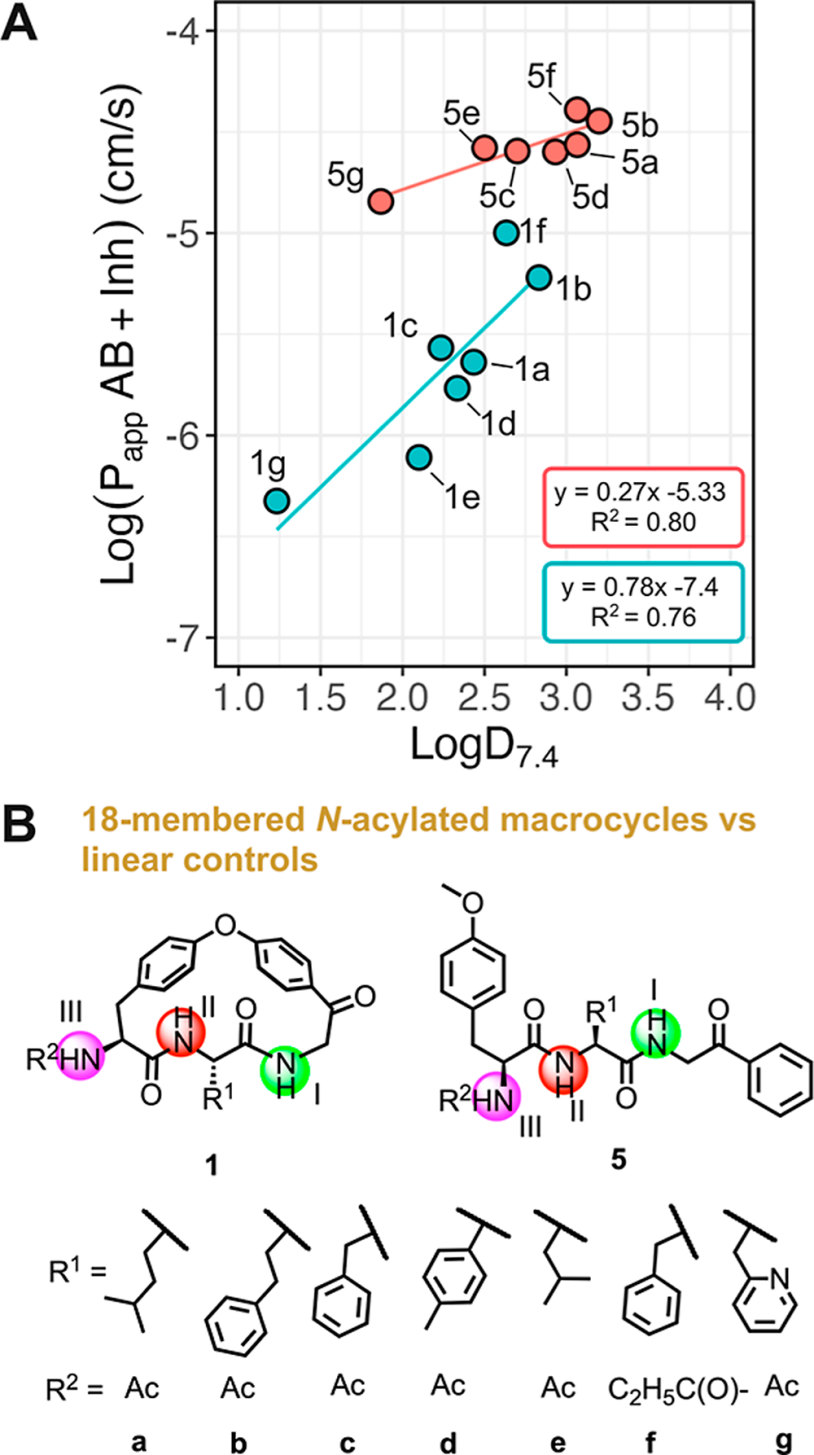
(A) Correlations between efflux-inhibited, passive cell permeability across a Caco-2 cell monolayer [log­(*P*_app_ AB + Inh)] and lipophilicity (log *D*_7.4_, determined by the shake-flask method) for seven 18-membered macrocycles (in cyan) and a series of linear matched molecular pairs (in red). The plotted mean values were obtained from three to six repeats for log­(*P*_app_ AB + Inh) and from three or four repeats for log *D*_7.4_. (B) Structures of the macrocycles and linear analogues included in the correlations in panel A.

The disconnection of the macrocyclic ring and the subsequent addition of a methyl group to the phenolic oxygen atom that converted the series 1 macrocycles into linear analogues led to log *D*_7.4_ increasing by, on average, 0.5 units for the seven matched pairs (Figure 5A). Interestingly, the replacement of the *N*-acetyl group by an *N*-propionyl group (cf. **1c** → **1f** and **5c** → **5f**), i.e., the addition of a methylene group, led to an increase in log *D*_7.4_ of approximately 0.4 units. This suggests that the increase in log *D*_7.4_ for the linear analogues mainly originated from the methyl group added to the phenolic oxygen.

Distinct correlations between cell permeability and log *D*_7.4_ were observed for the two series, with the linear analogues having an increased cell permeability over the series 1 macrocycles at a specific log *D*_7.4_ value (Figure 5A). This increase in permeability ranged from 0.5 to 1.2 logarithmic units, i.e., from a 3- to 16-fold increase. We speculate that the increase originates from the fact that the more flexible linear analogues can adopt low-energy conformations that are less polar than those of the corresponding macrocycles when crossing a cell membrane. This hypothesis was investigated by in silico conformational analysis of macrocycle **1c** and linear-matched pair **5c**, followed by variable-temperature (VT) NMR studies of these two compounds. Both conformational ensembles were also compared to the reported crystal structure of **1c** ^18^ and the novel structure of **5c**, disclosed herein.

The crystal structure of linear analogue **5c** (Figure S5) was determined by X-ray diffraction analysis of a crystal obtained via slow solvent evaporation from acetone. Just as for the crystal structures of macrocycles **1g**, **2c**, and **21c**, the amide backbone of **5c** forms an intermolecular network of hydrogen bonds in the crystal (Figure S10). Thus, the conformation of **5c** in the crystal is most likely representative of a conformation in an aqueous ensemble of **5c**. Because of the very high flexibility of linear analogue **5c**, we judged that optimization of its crystal structure, as performed for **1g**, **2c**, and **21c** (cf. above), would only identify a local minimum instead of providing a satisfactory description of the conformational landscape populated by **5c** in a nonpolar environment. Instead, a protocol that has been reported^20^ to provide an accurate description of the conformational ensemble of the Boc-protected analogue of **1c** was used to gain insight into the ensemble of **5c** in a membrane-like environment. The protocol employed Monte Carlo conformational sampling in an implicit nonpolar environment, followed by clustering based on the *R*_gyr_ and SA 3D PSA of the conformations (Figure S12) and quantum mechanical (QM) energy minimization. In order to limit the computational expense, the ensemble obtained by Monte Carlo sampling was divided into ten clusters before QM energy minimization. The in silico ensemble of **1c** was obtained from that of its Boc-protected analogue^20^ by replacement of the Boc group in each of the ten conformations by an acetyl group, followed by energy minimization.

Comparison of the ten conformations in the ensembles of macrocycle **1c** and linear matched pair **5c** rationalized the higher permeability of **5c**. The conformations in the ensemble of **5c** were less polar and substantially more lipophilic, i.e., they had a lower solvent-accessible 3D polar surface area (SA 3D PSA) and a higher solvent-accessible 3D nonpolar surface area (SA 3D NPSA), than those in the ensemble of **1c** (Figure 6A,B and Table S3). This observation is illustrated by the differences in the Boltzmann-weighted averages of these two polarity descriptors between **5c** and **1c** (ΔSA 3D PSA, −15 Å^2^, ΔSA 3D NPSA +113 Å^2^). While the ensemble of **5c** has a somewhat larger *R*_gyr_ than that of **1c** (Δ*R*_gyr_ 0.7 Å), the *R*_gyr_ of the ensemble for both compounds is far below 7 Å, the value above which passive permeability has been proposed to be adversely influenced by size.^27^ The higher polarity of the ensemble of macrocycle **1c** originates from the fact that the two amide bonds containing NH-II and NH-III are exposed to the surrounding environment in most of the conformations, just as in the crystal structure of **1c**, in combination with the fact that macrocyclization reduces the exposure of the lipophilic phenyl groups of the bisaryl ether moiety to the surrounding solution (Figure 6C). In contrast, the increased flexibility obtained by opening of the macrocyclic ring at the bisaryl ether to give **5c** allows greater exposure of these two phenyl groups and a larger degree of shielding of the three polar amide bonds, e.g., through a higher degree of formation of IMHBs (Figures 6D and S13). As expected from the intermolecular networks in the crystals of **1c** and **5c**, the conformation adopted in the solid state by each compound had a substantially higher SA 3D PSA than the corresponding in silico ensembles (Figure 6A).

**Figure 6:**
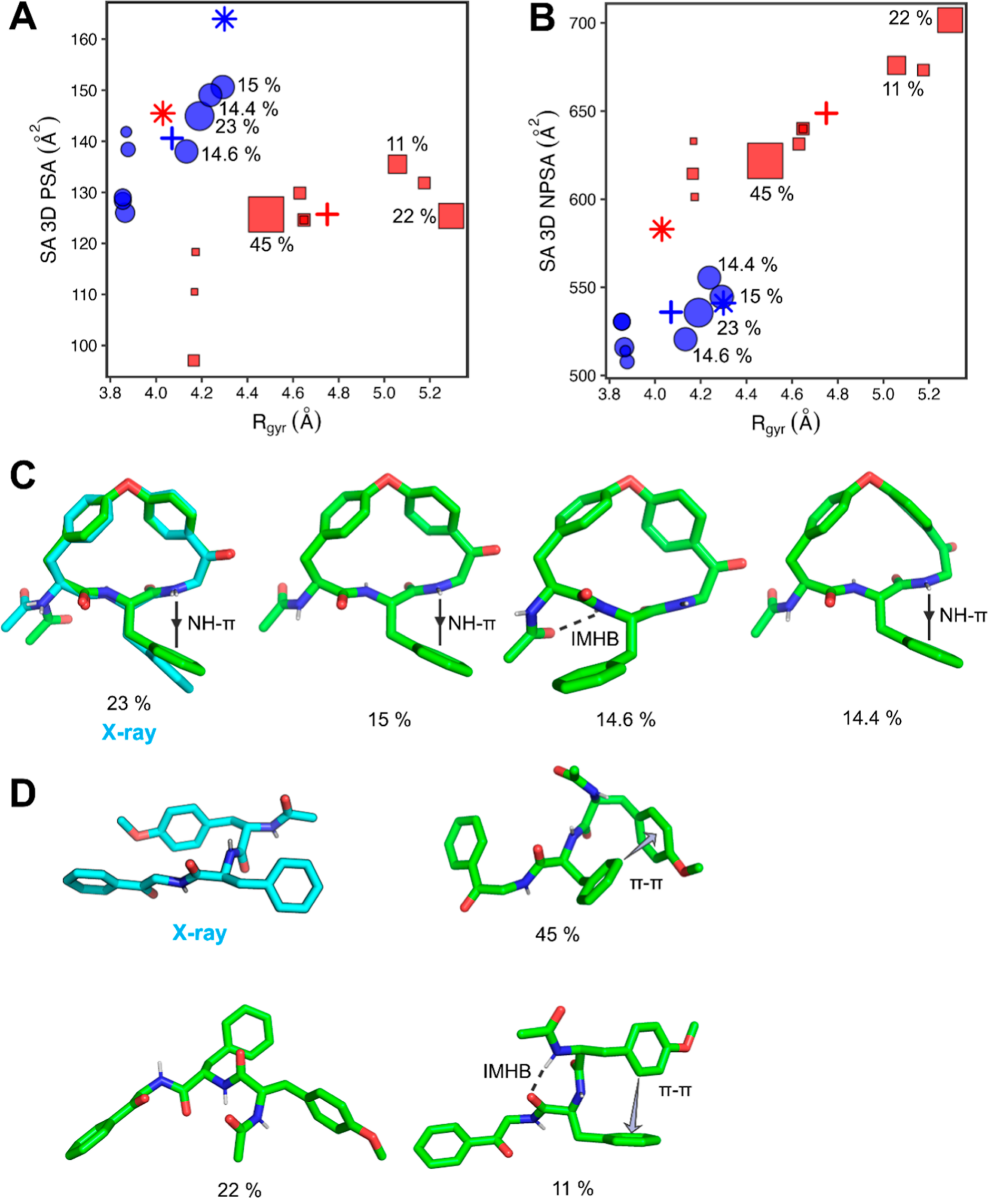
(A) Solvent-accessible 3D polar surface area (SA 3D PSA) and (B) solvent-accessible 3D nonpolar surface area (SA 3D NPSA) versus the radius of gyration (*R*_gyr_) of the conformations from the in silico conformational analysis of macrocycle **1c** (in blue) and linear matched pair **5c** (in red). The area of each circle or square is proportional to the population of the corresponding conformation. The populations (in %) of the conformations that have a population >10% are indicated. Boltzmann-weighted averages are shown as plus signs for each compound, while the star indicates the descriptors calculated for the crystal structure of each compound. (C,D) Structures of the conformations that have a population >10% of macrocycle **1c** and linear matched pair **5c**, respectively. Intramolecular interactions are indicated. In panel C, the most populated conformation of **1c** has been superimposed on the crystal structure of **1c** (in cyan). In panel D, the crystal structure of **5c** is shown in cyan. IMHB, intramolecular hydrogen bond.

VT NMR spectroscopy was performed in CDCl_3_ and DMSO-*d*_6_ to further investigate to what extent the three amide protons of macrocycle **1c** and linear analogue **5c** were solvent-exposed or shielded from the solvent. As **19c**, the Boc-protected precursor of **1c**, was more soluble in CDCl_3_ and gave spectra of higher quality, both compounds were studied. In CDCl_3,_ the temperature dependence of the chemical shift was low for the three NH protons in macrocycles **1c** and **19c** (Table 3), indicating the NH protons to be either exposed to the solvent or shielded by strong intramolecular interactions.^28^ The upfield chemical shift of NH-I in **19c** (4.88 ppm, Table 1) is diagnostic of it being involved in an intramolecular NH–π interaction. This NH–π interaction is also found in the minimum energy conformation of **1c**, which is almost identical to the crystal structure of **1c** (Figure 6C; rmsd 0.17 Å, Figure S14),^18^ and it is also present in all but one of the other in silico conformations of this macrocycle (Figures 6C and S15). In contrast, NH-II and NH-III are solvent-exposed in most of the conformations in the in silico ensemble of **1c** (Figures 6C and S15), just as in the crystal structure. For linear analogue **5c,** the temperature coefficient was also low for NH-I in CDCl_3_, but it was substantially larger for NH-II and NH-III, indicating that these two NH protons were shielded at low temperature, but exposed to the solvent as the temperature increased (Table 3).^28^ NH-I is solvent-exposed in conformations representing most of the in silico ensemble of **5c** (86%, Figures 6D and S16), suggesting this to be the reason for its low temperature coefficient. In conclusion, the NMR data obtained in CDCl_3_ agree with rigid conformations for macrocycles **1c** and **19c**, in which NH-I is involved in an intramolecular NH–π interaction while NH-II and NH-III are solvent-exposed. The VT NMR data in CDCl_3_ also fit with the greater conformational flexibility found in the in silico conformational analysis of **5c**.

**Table 3:**
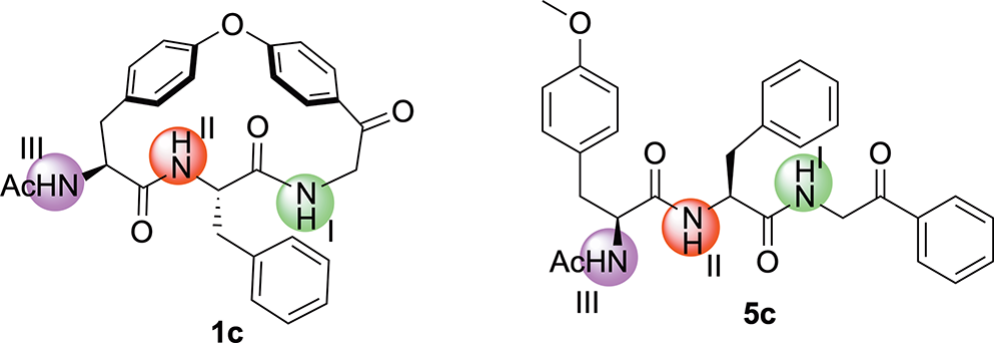
Temperature Coefficients (Δδ/*T*, ppb/K) for the NH Protons of **1c**, **19c** and **5c** in CDCl_3_ and DMSO-*d*_6_

	CDCl_3_			DMSO-*d*_6_		
macrocycle	NH (I)	NH (II)	NH (III)	NH (I)	NH (II)	NH (III)
**1c**	–1.52	–2.36	–2.74	–3.44	–4.90	–4.90
**19c**	–0.74	–1.66	–1.26	–3.90	–2.26	–7.40
**5c**	–1.35	–5.20	–4.12	–5.94	–6.42	–4.60

In DMSO-*d*_6_, the temperature coefficients of all three NH protons in each of **1c**, **19c,** and **5c** increased as compared to those in CDCl_3_ (Table 3). This reveals that the intramolecular NH–π interaction between NH-I and the adjacent benzyl side chain is disrupted in a polar environment,^29^ in agreement with previous conformational studies of **1c** and **19c**.^18,20^ In addition, the high temperature coefficients for NH-II and NH-III in the three compounds indicate that these NH protons are involved in hydrogen bonds to DMSO that form and break dynamically.

### Solubility

The aqueous solubility of the macrocycles belonging to series 1 and 4, determined in PBS at pH 7.4 using the dried DMSO principle,^30^ showed a large variation between individual macrocycles in each series (Figure 7A,B). That is, the amino acid that contributed the R^1^ side chain has a pronounced influence on solubility. For series 1, solubility ranged from very low for **1b** (17 μM, R^1^ = phenylethyl) to high for **1g** (≥1 mM, R^1^ = 2-pyridylmethyl), while it ranged from moderate for **4c** (217 μM, R^1^ = benzyl) to high for **4a** (908 μM, R^1^ = 3-methylbutyl) in series 4. These two series of macrocycles showed an inverse correlation between solubility and log *D*_7.4_, where solubility dropped rapidly for series 1 as log *D*_7.4_ increased above a threshold at approximately 2.5. For the macrocycles of series 4, the decrease in solubility with increasing log *D*_7.4_ was much less pronounced. Linear compounds **5a**–**5f**, which have nonpolar R^1^ side chains, all had a log *D*_7.4_ value ≥2.5, providing an explanation for their low solubilities (2–40 μM), most of which were far below their macrocyclic matched pairs (Figure 7C). Only compound **5g**, which has a 2-pyridylmethyl side chain and a log *D*_7.4_ value of 1.9, stood out by having a high solubility (>1 mM) similar to its macrocyclic parent **1g**. Analogously, only the most lipophilic macrocycle **1b** (log *D*_7.4_ = 2.8) had a low solubility, comparable to its linear analogue **5b**. No distinct influence of conformational preferences was observed for the solubility within each of the series of compounds studied herein. This is likely explained by the fact that the intramolecular interactions that reduce the polarity in nonpolar environments and thus increase the permeability for some of the series 1 macrocycles, and for **2c** over **3c**, are disrupted in aqueous solution. In summary, the aqueous solubilities of the macrocycles in series 1 and 4 varied substantially depending on the nature of the R^1^ side chain, i.e., solubility decreased with increasing lipophilicity, with series 1 showing a sharp drop at log *D*_7.4_ > 2.5. Most likely, the low solubilities of all but **5g** of the linear analogues originate from their high lipophilicities (log *D*_7.4_ > 2.5). It is worth noting that while cell permeability showed a strong linear correlation to log *D*_7.4_, the inverse correlation between solubility and log *D*_7.4_ was weaker and sigmoidal for series 1.

**Figure 7:**
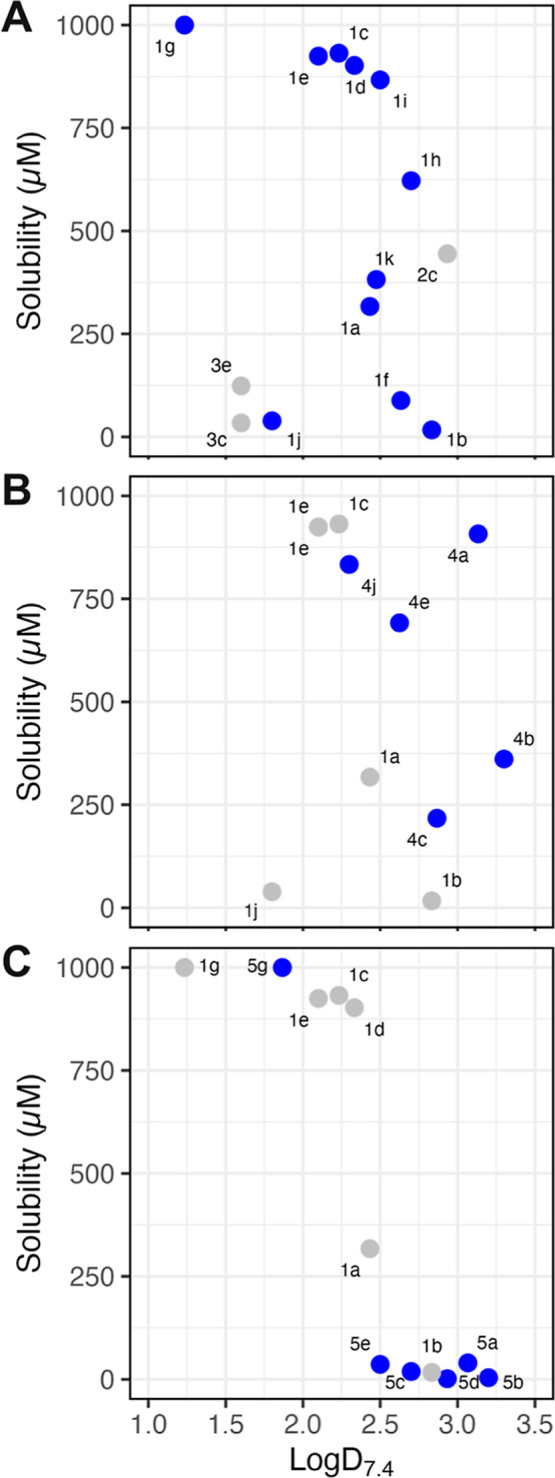
Correlations between aqueous solubility, determined in PBS at pH 7.4 using the dried DMSO principle,^30^ and lipophilicity (log *D*_7.4_, determined by the shake-flask method) for (A) the macrocycles belonging to series 1 (in blue), 2 and 3 (both in gray), (B) the macrocycles of series 4 (in blue) with matched molecular pairs from series 1 (in gray), and (C) the linear analogues of series 5 (in blue) with matched pairs of macrocycles from series 1 (in gray). Compound **5g** has a solubility ≥1000 μM and has been placed at 1000 μM in panel C. The plotted mean values were obtained from one to four repeats for the aqueous solubility and from three or four repeats for log *D*_7.4_. The structures of the compounds are found in Figure 1.

## Discussion and Conclusions

Macrocyclization is employed to induce conformational ensembles that are entropically disfavored for linear analogues.^31,32^ This conformational restriction allows macrocycles to bind to difficult-to-drug targets that have large and featureless binding sites,^6–8^ but it may also provide them with physicochemical and ADME properties that differ substantially from their nonmacrocyclic counterparts.^33–35^ Unfortunately, predicting the conformational ensembles of macrocycles, with the objective of predicting their properties, remains challenging and often provides results that do not agree with experimental data.^36,37^ Reasons for these difficulties include that macrocycles retain some flexibility, form noncovalent transannular interactions such as intramolecular hydrogen bonds, and that their conformations also are influenced by steric interactions and ring strain.^38^ In addition, conformational analysis is further complicated since these interactions are often coupled, because of which small structural modifications to a macrocyclic scaffold can change the conformational preferences of remote parts of the macrocycle. The results reported herein further exemplify how seemingly minor structural modifications of macrocycles can result in unexpected conformational changes and how they impact cell permeability.

The 18-membered macrocycles of series 1 reveal how the correct positioning of a phenyl or pyridyl group in a side chain can form environment-dependent, chameleonic NH–π or IMHB interactions with an adjacent amide bond that, on average, result in a 3.5-fold increase in passive permeability across Caco-2 cells. As for macrocycle **1g**, chameleonic masking of an amide bond by an adjacent pyridyl or *N*,*N*-pyrrolidinylglutamine group has previously been reported to enhance the permeability in a cyclic hexapeptide model system.^39,40^ Herein, we also demonstrate that removal or introduction of a single methylene group in the side chain either does not allow or weakens the intramolecular interaction and results in loss of the increased permeability. Molecular chameleonicity is considered to be of particular importance for the ADME properties of macrocycles and other compounds in the bRo5 space^15,16^ and was recently reported to provide up to a 2-fold improvement in oral absorption for compounds in the 800–1000 Da MW range.^41^

The isomeric, 19-membered macrocycles **2c** and **3c**, which differ only by the position of a single methylene group within the macrocyclic ring, had lipophilicities and passive permeabilities that differed by more than one order of magnitude. QM optimization of the crystal structures of the two macrocycles revealed that the location of the methylene group in the less permeable **3c** prevented the formation of a chameleonic NH–π interaction observed for the more permeable **2c**. The location of the methylene group in **3c** also induced a conformational change that exposed one of the amide bonds to a greater extent than in **2c**. As a consequence, the surface-accessible 3D polar surface area of **2c** was lower than that of **3c**, rationalizing the permeability difference between these two macrocycles.

Early reports in the literature have sometimes been misinterpreted to conclude that macrocyclization of linear compounds often leads to increased cell permeability.^5,33^ However, this is not necessarily the case—in fact, results vary substantially between studies. Thus, increases in permeability that range from close to 100-fold,^33,42^ via approximately 10-fold^43,44^ to very small^35,45,46^ have been reported for cyclization of lead-like semipeptides,^35,45^ peptides,^33,43^ peptoids,^44^ peptide-peptoid hybrids,^42^ and depsipeptides.^47^ In contrast, another study found linear peptides to be more permeable than their macrocyclic matched pairs.^34^ Notably, the influence of macrocyclization may vary from negligible to very high within series of structurally related macrocycles.^33,42^ The influence of macrocyclization on both cell permeability and aqueous solubility has also been investigated.^35^ Matched pairs in series of macrocycles and linear analogues were found to have similar permeabilities, while the linear compounds had higher aqueous solubilities. Herein, we found that opening of the ring of several of the macrocycles in series 1 led to a substantial increase in cell permeability for the resulting linear compounds in series 5 and often also to a large drop in aqueous solubility. The combined use of computational conformational analysis and variable temperature NMR spectroscopy allowed us to trace the origin of these property differences to major differences between the conformational ensembles for a matched pair from the two series. The ensemble of macrocycle **1c** was found to be restricted to similar conformations that exposed the polar amide bonds to the surrounding environment. In contrast, the linear matched pair **5c** populated different, often more extended conformations, many of which provided a larger degree of shielding of the amide bonds and also exposure of the lipophilic aryl groups. Solubility did not depend on conformational preferences within the different series, but was predominantly low at log *D*_7.4_ > 2.5.

Macrocyclic drugs often reside far into the challenging bRo5 space, e.g., at MWs of 700–1000 Da, where conversion of a single functional group can have a major impact on the drugs’ properties. For instance, the substitution of a single hydroxyl group in the orally bioavailable drug sirolimus by a tetrazole provided zotarolimus, which has an increased lipophilicity and a reduced solubility making it ideal for slow release from coronary stents.^11^ Exposed secondary amides in the backbone of macrocyclic peptides are notorious for reducing cell permeability and potentially also oral bioavailability. While N-methylation of exposed amides constitutes an established strategy for improving the cell permeability of cyclic peptides,^48^ amide-to-ester substitution has been reported as a useful alternative more recently.^49,50^ For the 18-membered macrocycles of series 1, replacement of the exocyclic *N*-acetylamino substituent by a dimethylamino group to give the series 4 macrocycles on average led to an increase in cell permeability by an order of magnitude. This permeability increase can be rationalized from the low p*K*_a_ (6.54–6.84) of the dimethylamino group in the series 4 macrocycles, leaving substantial amounts unprotonated at physiological pH values. Similarly, an earlier study of a library of natural product-inspired macrocycles identified dimethylamines and some other functional groups as being more compatible with high cell permeability, while other, more polar groups such as ureas and sulfonamides were associated with low permeability.^14^

In conclusion, the particular conformational properties of macrocycles provide them with physicochemical and ADME properties that may differ substantially between apparently similar macrocycles, and even more from their linear matched molecular pairs. As reported herein, appropriately located side chains may shield adjacent polar groups via intramolecular NH–π interactions and IMHBs, thereby enhancing cell permeability. However, no influence was observed for the more flexible linear matched pairs, indicating such intramolecular interactions to be dependent on the overall conformation of the molecular backbone. In addition, expansion of the macrocyclic ring by a methylene group at two positions induced different conformations that either exposed or shielded an amide bond, which resulted in a >10-fold permeability difference. Our results, and those reported previously by others,^34,35^ illustrate that macrocyclization does not guarantee desirable physicochemical and in vitro ADME properties. Instead, the outcome depends on the balance between polar and nonpolar surface area imposed by the macrocyclization as compared to the more flexible linear matched pairs. We have not implemented the structural and mechanistic results reported herein in a lead optimization project where maintaining the activity against a therapeutic target is crucial, but we are hopeful that our results will contribute to the improved design of cell-permeable and orally absorbed macrocycles in drug discovery.

## Experimental Section

### General Synthetic and Analytical Methods

The synthesis and characterization of macrocycles **1** (**a**, **b**, **c**, **d**, **f**, **g**, **j**), **19** (**a**, **b**, **c**, **d**, **f**, **g**, **j**) and building block *tert*-butyl ((2*S*)-1-((2-(4-fluoro-3-nitrophenyl)-2-hydroxyethyl)­amino)-1-oxo-3-phenylpropan-2-yl)­carbamate (**9c**), 2-(4-fluoro-3-nitrophenyl)-2-hydroxyethan-1-ammonium chloride (**8**), (*S*)-2-((*tert*-butoxycarbonyl)­amino)-3-(4-((*tert*-butyldimethylsilyl)­oxy)­phenyl)­propanoic acid (**11**), and (*S*)-2-((*tert*-butoxycarbonyl)­amino)-4-(4-((*tert*-butyldimethylsilyl)­oxy)­phenyl)­butanoic acid (**12**) were performed as reported in our previous work.^13^

Glassware used to carry out these experiments was dried in an oven. All nonaqueous reactions were performed in oven-dried glassware under an argon atmosphere. Büchi rotavapor R-114 was used for evaporating solvents. Hydrogenation was performed in a Parr hydrogenator (series 5100). Reactions were monitored by LC–MS (an Agilent 1100 series HPLC having a C18 Atlantis T3 column) (3.0 × 50 mm, 5 μm) connected to a Waters micromass Z_Q_ (model code: MM1) mass spectrometer with electrospray ionization mode, which was used for detection of molecular ions and acetonitrile–water (flow rate 0.75 mL/min over 6 min was used as the mobile phase), and thin layer chromatography on silica gel 60 F_254_ plates from Merck. TLC was visualized by UV light (254 nm) and staining with phosphomolybdic acid in ethanol or ethanolic H_2_SO_4_ (5% v/v).

Compounds were purified by column chromatography using silica gel (Matrex, 60 Å, 35–70 μm, Grace Amicon), a Gilson HPLC (equipped with Gilson 322 pump, UV/Visible-156 detector and 202 collector, a Kromasil C8 column (250 × 21.2 mm, 5 μm) using acetonitrile–water gradients as eluents with a flow rate of 15 mL/min and detection at 214 or 254 nm), and a Waters Fraction Lynx system with a Waters Acquity SQD using a Waters binary gradient module 2525 equipped with a Waters XBridge C18 5 μ ODB 19 × 150 mm and 10 × 100 mm column at ambient temperature.

Protons in the ^1^H NMR of compounds were assigned with the help of ^13^C, COSY, HSQC, and HMBC. ^13^C,^19^F coupling in compounds **9** (**e**, **h**, **i**), **10** (**c**, **e**), **13** (**e**, **h**, **i**), **14** (**c**), and **15** (**c**, **e**) were not assigned because of the complexity of the spectra; instead, peaks observed in the ^13^C NMR are listed. NMRs were recorded on 298 K on an Agilent Technologies 400 MR spectrometer at 400 MHz (^1^H) or 100 MHz (^13^C), on an OXFORD AS500 spectrometer at 500 MHz (^1^H) or 126 MHz (^13^C), and on an uncalibrated temperature of 25 °C on a Bruker AVANCE III 500 spectrometer with a 5 mm QNP cryo-probe at a frequency of 500 MHz (^1^H) or 126 MHz (^13^C). The residual peak of the respective solvent was used as internal standard [CDCl_3_ (CHCl_3_ δH 7.26 ppm, CDCl_3_ δC 77.0 ppm) or CD_3_SOCD_3_ (CD_2_HSOCD_3_ δH 2.50 ppm, CD_3_SOCD_3_ δC 39.5 ppm)]. HRMS for all newly synthesized compounds used in this manuscript were recorded on an LCT Premier connected to a Waters Acquity UPLC I-class in electrospray ionization (ESI) and APCI mode using acetonitrile/water as the mobile phase (1:1, with a flow rate of 0.25 mL/min). Out of the 26 compounds reported in the article, 17 are ≥95% pure, six are 93–94% pure, while **5f**, **5b**, and **5c** have 82, 89, and 90% purity, respectively, as determined by reversed phase HPLC. log *D*_7.4_ and permeability across Caco-2 cells are not affected for the three compounds having lower purities since detection and quantification are performed by LCMS. It cannot be ruled out that the 82% purity of compound **5f** might have influenced its aqueous solubility; therefore, **5f** was not included in the discussion of how solubility correlates to log *D*_7.4_ (Figure 7C).

#### *tert*-Butyl ((2*S*)-1-((2-(4-Fluoro-3-nitrophenyl)-2-hydroxyethyl)­amino)-4-methyl-1-oxopentan-2-yl)­carbamate (**9e**)

Boc-leucine-OH (**6e**) (2.52 g, 10.9 mmol) and HATU (4.91 g, 12.9 mmol) were dissolved in DCM (33 mL), and DMF (1 mL) and DIPEA (2.60 mL, 14.9 mmol) were added. After stirring for 10 min at room temperature, **8** (2.37 g, 10.0 mmol) was added, followed by the addition of another portion of DIPEA (2.60 mL, 14.9 mmol). TLC showed complete conversion after stirring at room temperature for 40 min. The reaction mixture was diluted with DCM (100 mL), washed with 1 M HCl (1 × 100 mL), saturated aqueous sodium bicarbonate solution (2 × 100 mL), and brine (1 × 100 mL), dried over Na_2_SO_4_, filtered, and concentrated under reduced pressure. The resulting residue was purified on a silica gel column using 75% EtOAc in *n*-hexane as the mobile phase to give **9e** (2.92 g, 7.06 mmol, 70% yield) as light-yellow foam. HRMS (ESI) *m*/*z*: calcd for C_20_H_31_N_3_O_6_F [M + H]^+^, 428.2197; found, 428.2190. ^1^H NMR (400 MHz, CDCl_3_): δ 8.11–8.05 (m, 1H), 7.68–7.61 (m, 1H), 7.25–7.21 (m, 1H), 7.11 (s, 1H), 5.24–5.12 (m, 1H), 4.95–4.89 (m, 1H), 4.13–4.02 (m, 1H), 3.71–3.58 (m, 1H), 3.42–3.23 (m, 1H), 1.68–1.43 (m, 4H), 1.38 (s, 9H), 0.93–0.86 (m, 6H). ^13^C NMR (101 MHz, CDCl_3_): δ 174.7, 156.2, 139.1, 139.1, 137.2, 133.2, 133.1, 133.1, 133.0, 123.6, 123.6, 123.5, 118.6, 118.5, 118.4, 118.3, 80.7, 77.3, 71.9, 53.5, 47.5, 47.5, 41.1, 28.3, 24.8, 24.8, 22.9, 21.9, 21.9.

#### *tert*-Butyl ((2*S*)-1-((2-(4-Fluoro-3-nitrophenyl)-2-hydroxyethyl)­amino)-1-oxo-3-(*p*-tolyl)­propan-2-yl)­carbamate (**9h**)

(*S*)-Boc-*p*-methylphenylalanine-OH (**6h**) (2.20 g, 7.8 mmol) and HATU (3.50 g, 9.2 mmol) were dissolved in DCM (36 mL) and DMF (1 mL), and DIPEA (1.60 mL, 9.2 mmol) was added. After stirring for 10 min at room temperature, **8** (1.84 g, 7.8 mmol) was added, followed by the addition of another portion of DIPEA (1.60 mL, 9.2 mmol). TLC showed complete conversion after stirring at room temperature for 40 min. The reaction mixture was diluted with DCM (100 mL), washed with 1 M HCl (1 × 100 mL), saturated aqueous sodium bicarbonate solution (2 × 100 mL), and brine (1 × 100 mL), dried over Na_2_SO_4_, filtered, and concentrated under reduced pressure. The resulting residue was purified on a silica gel column using 50% EtOAc in *n*-hexane as the mobile phase to give **9h** (2.65 g, 5.74 mmol, 73% yield) as light-yellow foam. HRMS (ESI) *m*/*z*: calcd for C_23_H_28_FN_3_O_6_FNa [M + Na]^+^, 484.1860; found, 484.1855. ^1^H NMR (400 MHz, CDCl_3_): δ 8.07–8.02 (m, 1H), 7.61–7.68 (m, 1H), 7.27–7.17 (m, 2H), 7.15–7.02 (m, 3H), 6.71–6.58 (m, 1H), 5.19–5.15 (m, 1H), 4.87–4.84 (m, 1H), 4.79–4.76 (m, 1H), 4.35–4.21 (m, 1H), 3.68–3.51 (m, 1H), 3.26–3.13 (m, 1H), 3.04–2.96 (m, 2H), 2.32, 2.31 (2s, 3H), 1.39, 1.38 (2s, 9H). ^13^C NMR (101 MHz, CDCl_3_): δ 173.2, 173.1, 156.0, 156.0, 153.4, 153.4, 138.8, 138.8, 138.7, 138.7, 137.2, 137.2, 137.1, 137.1, 136.8, 136.7, 133.1, 133.1, 132.9, 132.9, 132.8, 132.8, 129.4, 129.4, 129.1, 129.0, 123.4, 123.4, 123.3, 118.5, 118.4, 118.3, 118.2, 80.7, 77.3, 77.2, 77.0, 76.7, 71.6, 71.4, 56.2, 47.6, 47.5, 37.9, 37.7, 28.2, 28.2, 21.3, 21.0.

#### *tert*-Butyl ((2*S*)-1-((2-(4-Fluoro-3-nitrophenyl)-2-hydroxyethyl)­amino)-3-(4-fluorophenyl)-1-oxopropan-2-yl)­carbamate (**9i**)

(*S*)-Boc-*p*-fluorophenylalanine-OH (**6i**) (2.20 g, 7.76 mmol) and HATU (2.95 g, 7.76 mmol) were dissolved in DCM (30 mL) and DMF (2 mL), and DIPEA (2.70 mL, 15.53 mmol) was added. After stirring for 10 min at room temperature, **8** (1.83 g, 7.8 mmol) was added, followed by the addition of another portion of DIPEA (2.70 mL, 15.53 mmol). TLC showed complete conversion after stirring at room temperature for 40 min. The reaction mixture was diluted with DCM (100 mL), washed with 1 M HCl (1 × 100 mL), saturated aqueous sodium bicarbonate solution (2 × 100 mL), and brine (1 × 100 mL), dried over Na_2_SO_4_, filtered, and concentrated under reduced pressure. The resulting residue was purified on a silica gel column using 50% EtOAc in *n*-hexane as the mobile phase to give **9i** (2.49 g, 5.37 mmol, 69% yield) as light-yellow foam. ^1^H NMR (400 MHz, CDCl_3_): δ 8.10–8.01 (m, 1H), 7.30–7.10 (m, 3H), 6.98 (m, 2H), 6.72 (s, 1H), 5.20–5.09 (m, 1H), 4.91–4.78 (m, 1H), 4.36–4.23 (m, 1H), 3.70–3.50 (m, 1H), 3.33–2.88 (m, 3H). ^13^C NMR (101 MHz, CDCl_3_): δ 173.0, 172.9, 163.2, 163.1, 160.7, 160.7, 156.1, 156.0, 155.7, 153.4, 153.4, 138.7, 138.7, 138.7, 138.6, 137.2, 137.2, 137.2, 137.1, 132.9, 132.9, 132.8, 132.8, 132.0, 132.0, 130.7, 130.7, 130.7, 130.7, 123.4, 123.4, 118.6, 118.5, 118.3, 118.3, 115.7, 115.6, 115.4, 115.4, 80.9, 76.7, 71.8, 71.4, 56.2, 47.5, 47.4, 37.3, 28.2.

#### *tert*-Butyl ((2*S*)-4-((2-(4-Fluoro-3-nitrophenyl)-2-hydroxyethyl)­amino)-3-oxo-1-phenylbutan-2-yl)­carbamate (**10c**)

(*S*)-Boc-β-phenylalanine-OH (**7c**) (1.0 g, 3.6 mmol) and HATU (1.59 g, 4.2 mmol) were dissolved in DCM (20 mL) and DMF (1 mL), and DIPEA (0.730 mL, 4.2 mmol) was added. After stirring for 10 min at room temperature, **8** (0.851 g, 3.6 mmol) was added, followed by the addition of another portion of DIPEA (0.730 mL, 4.2 mmol). TLC showed complete conversion after stirring at room temperature for 40 min. The reaction mixture was diluted with DCM (100 mL), washed with 1 M HCl (1 × 100 mL), saturated aqueous sodium bicarbonate solution (2 × 100 mL), and brine (1 × 100 mL), dried over Na_2_SO_4_, filtered, and concentrated under reduced pressure. The resulting residue was purified on a silica gel column using 50% EtOAc in *n*-hexane as the mobile phase to give **10c** (1.04 g, 2.26 mmol, 63% yield) as light-yellow foam. HRMS (ESI) *m*/*z*: calcd for C_23_H_28_FN_3_O_6_FNa [M + Na]^+^, 484.1854; found, 484.1870. ^1^H NMR (500 MHz, DMSO-*d*_6_): δ 8.10–8.07 (m, 1H), 7.93–7.90 (m, 1H), 7.76–7.73 (m, 1H), 7.54–7.50 (m, 1H), 7.26–7.23 (t, *J* = 7.4 Hz, 2H), 7.18–7.15 (m, 1H), 7.10–7.08 (m, 2H), 6.66–6.62 (m, 1H), 5.80–5.79 (m, 1H), 4.78–4.66 (m, 1H), 3.92–3.80 (m, 1H), 3.41–3.33 (m, 1H), 3.26–3.19 (m, 1H), 2.56–2.51 (m, 2H), 2.24–2.14 (m, 2H), 1.30 (s, 9H). ^13^C NMR (126 MHz, DMSO-*d*_6_): δ 170.3, 154.7, 154.6, 154.6, 152.5, 152.5, 141.1, 141.1, 141.0, 138.8, 136.4, 136.3, 134.1, 134.0, 129.0, 127.9, 125.8, 125.8, 123.4, 123.4, 118.1, 118.0, 117.9, 117.9, 77.4, 77.4, 69.8, 69.8, 49.2, 49.1, 45.9, 45.9, 28.2.

#### *tert*-Butyl ((3*S*)-1-((2-(4-Fluoro-3-nitrophenyl)-2-hydroxyethyl)­amino)-5-methyl-2-oxohexan-3-yl)­carbamate (**10e**)

(*S*)-Boc-β-leucine-OH (**7e**) (0.883 g, 3.6 mmol) and HATU (1.59 g, 4.2 mmol) were dissolved in DCM (20 mL) and DMF (1 mL), and DIPEA (0.730 mL, 4.2 mmol) was added. After stirring for 10 min at room temperature, **8** (0.657 g, 3.27 mmol) was added, followed by the addition of another portion of DIPEA (0.730 mL, 4.2 mmol). TLC showed complete conversion after stirring at room temperature for 40 min. The reaction mixture was diluted with DCM (100 mL), washed with 1 M HCl (1 × 100 mL), saturated aqueous sodium bicarbonate solution (2 × 100 mL), and brine (1 × 100 mL), dried over Na_2_SO_4_, filtered, and concentrated under reduced pressure. The resulting residue was purified on a silica gel column using 50% EtOAc in *n*-hexane as the mobile phase to give **10e** (0.820 g, 1.92 mmol, 60% yield) as light-yellow foam. HRMS (ESI) *m*/*z*: calcd for C_20_H_30_FN_3_O_6_FNa [M + Na]^+^, 450.2011; found, 450.2017. ^1^H NMR (500 MHz, DMSO-*d*_6_): δ 8.09–8.06 (m, 1H), 7.91–7.82 (m, 1H), 7.76–7.73 (m, 1H), 7.56–7.52 (m, 1H), 6.55–6.52 (m, 1H), 5.79–5.77 (m, 1H), 4.75–4.69 (m, 1H), 3.80–3.65 (m, 1H), 3.41–3.28 (m, 2H), 3.28–3.14 (m, 1H), 2.20–2.03 (m, 2H), 1.55–1.45 (m, 1H), 1.22–1.15 (m, 1H), 0.94–0.86 (m, 1H), 0.82–0.74 (m, 6H). ^13^C NMR (126 MHz, DMSO-*d*_6_): δ 170.4, 155.0, 155.0, 154.7, 154.6, 152.6, 152.6, 141.2, 141.1, 136.4, 136.4, 136.3, 136.3, 134.2, 134.1, 134.1, 134.1, 123.5, 123.5, 123.4, 118.1, 118.1, 118.0, 117.9, 77.3, 77.3, 69.9, 69.8, 45.8, 45.8, 45.7, 45.7, 43.1, 43.1, 41.7, 40.1, 40.0, 39.9, 39.9, 39.8, 39.7, 39.6, 39.5, 39.4, 39.4, 39.2, 39.0, 28.2, 28.1, 24.2, 24.2, 23.2, 21.6, 21.6.

#### *tert*-Butyl ((2*S*)-3-(4-((*tert*-Butyldimethylsilyl)­oxy)­phenyl)-1-(((2*S*)-1-((2-(4-fluoro-3-nitrophenyl)-2-hydroxyethyl)­amino)-4-methyl-1-oxopentan-2-yl)­amino)-1-oxopropan-2-yl)­carbamate (**13e**)

**10e** (2.65 g, 6.41 mmol) was dissolved in acetonitrile (50 mL), and 36% aq. HCl solution (10.0 mL) was added to it portionwise over 20 min at room temperature, and stirring was continued for another 20 min. The reaction mixture was then concentrated under reduced pressure, coevaporated with toluene (10 mL), and further dried under high vacuum. Then compound **11** (2.72 g, 6.88 mmol), HATU (2.62 g, 6.89 mmol), and DIPEA (1.20 mL, 6.89 mmol) were added to DCM (30 mL), followed by the addition of another portion of DIPEA (1.20 mL, 6.89 mmol). LC–MS showed complete conversion after stirring at room temperature for 45 min. The reaction mixture was diluted with DCM (100 mL), washed with an aqueous 1 M HCl solution (2 × 50 mL), saturated aqueous sodium bicarbonate solution (2 × 50 mL), and brine (2 × 50 mL), dried over anhydrous Na_2_SO_4_, filtered, and concentrated under reduced pressure. The resulting residue was then loaded on a silica gel column and purified using 50% EtOAc in *n*-hexane as the mobile phase to give **13e** (3.74 g, 5.41 mmol, 84%) as a light-yellow foam. HRMS (APCI) *m*/*z*: calcd for C_34_H_52_FN_4_O_8_FSi [M + H]^+^, 691.3533; found, 691.3528. ^1^H NMR (400 MHz, CDCl_3_): δ 8.11–8.07 (m, 2H), 7.66–7.61 (m, 2H), 7.24–7.20 (m, 2H), 7.05–6.99 (m, 4H), 6.77–6.74 (m, 4H), 6.60 (d, 1H), 6.53 (d, 1H), 5.06 (s, 2H), 4.88–4.83 (m, 2H), 4.49–4.41 (m, 2H), 4.27–4.19 (m, 2H), 3.72–3.52 (m, 6H), 3.24 (s, 3H), 3.04–2.89 (m, 1H), 2.62 (s, 1H), 1.72–1.60 (m, 2H), 1.55–1.40 (m, 4H), 1.37 (s, 9H), 1.34 (s, 9H), 0.96 (s, 9H), 0.94 (s, 9H), 0.89–0.87 (m, 12H), 0.16 (s, 6H), 0.15 (s, 6H). ^13^C NMR (101 MHz, CDCl_3_): δ 173.6, 156.1, 154.9, 154.9, 153.4, 153.4, 137.3, 137.2, 133.2, 133.1, 133.1, 130.3, 128.5, 123.7, 123.6, 123.6, 120.5, 120.5, 118.2, 81.2, 77.3, 71.9, 58.4, 56.5, 56.4, 52.1, 51.9, 47.7, 47.6, 40.6, 40.5, 28.2, 28.2, 25.7, 24.8, 24.7, 23.0, 23.0, 18.2, −4.3.

#### *tert*-Butyl ((2*S*)-3-(4-((*tert*-Butyldimethylsilyl)­oxy)­phenyl)-1-(((2*S*)-1-((2-(4-fluoro-3-nitrophenyl)-2-hydroxyethyl)­amino)-1-oxo-3-(*p*-tolyl)­propan-2-yl)­amino)-1-oxopropan-2-yl)­carbamate (**13h**)

**10h** (2.31 g, 5.02 mmol) was dissolved in acetonitrile (50 mL), and 36% aq. HCl solution (9.0 mL) was added to it portionwise over 20 min at room temperature, and stirring was continued for another 20 min. The reaction mixture was then concentrated under reduced pressure, coevaporated with toluene (10 mL), and further dried under high vacuum. Then compound **11** (2.18 g, 5.52 mmol), HATU (2.10 g, 5.53 mmol), and DIPEA (0.991 mL, 5.53 mmol) were added to DCM (30 mL), followed by the addition of another portion of DIPEA (0.991 mL, 5.53 mmol). LC–MS showed complete conversion after stirring at room temperature for 45 min. The reaction mixture was diluted with DCM (100 mL), washed with an aqueous 1 M HCl solution (2 × 50 mL), saturated aqueous sodium bicarbonate solution (2 × 50 mL), and brine (2 × 50 mL), dried over anhydrous Na_2_SO_4_, filtered, and concentrated under reduced pressure. The resulting residue was then loaded on a silica gel column and purified using 60% EtOAc in *n*-hexane as the mobile phase to give **13h** (2.71 g, 3.66 mmol, 73%) as a light-yellow foam. HRMS (ESI) *m*/*z*: calcd for C_38_H_51_FN_4_O_8_FSiNa [M + Na]^+^, 761.3358; found, 761.3364. ^1^H NMR (400 MHz, CDCl_3_): δ 8.09–8.03 (m, 1H), 7.66–7.60 (m, 1H), 7.29–7.20 (m, 2H), 7.12–6.97 (m, 4H), 6.92–6.88 (m, 2H), 6.84–6.80 (m, 2H), 6.31–6.25 (m, 1H), 4.86–4.74 (m, 2H), 4.70–4.62 (m, 1H), 4.15–4.05 (m,1H), 3.63–3.56 (m, 1H), 3.46–3.38 (m, 1H), 3.35–3.25 (m, 1H), 3.22–3.08 (m, 1H), 3.05–2.76 (m, 3H), 2.30 (s, 1H), 2.30 (s, 1H), 1.25 (s, 9H), 0.97 (s, 9H), 0.97 (s, 9H), 0.19 (s, 6H), 0.19 (s, 6H). ^13^C NMR (101 MHz, CDCl_3_): δ 172.6, 172.1, 171.2, 171.1, 156.2, 156.1, 156.0, 155.1, 155.0, 153.3, 139.0, 138.9, 138.8, 138.8, 137.2, 137.2, 136.9, 136.8, 133.0, 133.0, 132.9, 132.9, 132.6, 132.4, 130.1, 129.9, 129.5, 129.5, 129.0, 129.0, 128.1, 128.0, 123.5, 123.5, 123.5, 123.4, 120.5, 118.3, 118.3, 118.1, 118.1, 81.2, 81.2, 77.3, 77.2, 77.0, 76.7, 72.3, 71.4, 56.6, 53.5, 53.3, 48.1, 47.8, 36.4, 36.2, 28.0, 28.0, 27.9, 25.6, 21.0, 18.1, −4.4, −4.4.

#### *tert*-Butyl ((2*S*)-3-(4-((*tert*-Butyldimethylsilyl)­oxy)­phenyl)-1-(((2*S*)-1-((2-(4-fluoro-3-nitrophenyl)-2-hydroxyethyl)­amino)-3-(4-fluorophenyl)-1-oxopropan-2-yl)­amino)-1-oxopropan-2-yl)­carbamate (**13i**)

**10i** (2.31 g, 4.98 mmol) was dissolved in acetonitrile (30 mL), and 36% aq. HCl solution (6.0 mL) was added to it portionwise over 20 min at room temperature, and stirring was continued for another 40 min. The reaction mixture was then concentrated under reduced pressure, coevaporated with toluene (10 mL), and further dried under high vacuum. Then compound **11** (2.16 g, 5.47 mmol), HATU (1.89 g, 4.97 mmol), and DIPEA (1.73 mL, 9.95 mmol) were added to DCM (30 mL) and DMF (2 mL), followed by the addition of another portion of DIPEA (1.73 mL, 9.95 mmol). LC–MS showed complete conversion after stirring at room temperature for 30 min. The reaction mixture was diluted with DCM (100 mL), washed with an aqueous 1 M HCl solution (2 × 50 mL), saturated aqueous sodium bicarbonate solution (2 × 50 mL), and brine (2 × 50 mL), dried over anhydrous Na_2_SO_4_, filtered, and concentrated under reduced pressure. The resulting residue was then loaded on a silica gel column and purified using 50% EtOAc in *n*-hexane as the mobile phase to give **13i** (2.68 g, 3.66 mmol, 73%) as a light-yellow foam. ^1^H NMR (400 MHz, CDCl_3_): δ 8.07 (m, *J* = 13.6, 7.1, 2.2 Hz, 2H), 7.69–7.56 (m, 2H), 7.31–7.19 (m, 3H), 7.09–6.91 (m, 13H), 6.82 (t, *J* = 8.0 Hz, 4H), 6.24 (d, *J* = 8.2 Hz, 1H), 6.16 (d, *J* = 8.2 Hz, 1H), 4.80 (q, *J* = 4.7 Hz, 4H), 4.67 (dd, *J* = 13.6, 7.3 Hz, 2H), 4.11 (dq, *J* = 16.6, 5.8 Hz, 2H), 3.60 (ddd, *J* = 14.1, 6.9, 2.6 Hz, 1H), 3.45 (t, *J* = 9.5 Hz, 1H), 3.37–3.09 (m, 4H), 3.09–2.76 (m, 6H), 1.28 (d, *J* = 2.0 Hz, 18H), 0.97 (d, *J* = 2.8 Hz, 17H), 0.19 (d, *J* = 4.0 Hz, 11H). ^13^C NMR (101 MHz, CDCl_3_): δ 172.4, 171.7, 171.3, 171.1, 163.3, 163.2, 160.8, 160.7, 156.3, 156.0, 155.2, 155.0, 153.4, 138.9, 138.9, 138.8, 138.7, 137.2, 132.9, 132.9, 131.6, 131.6, 131.4, 131.3, 130.7, 130.6, 130.18, 128.09, 127.95, 123.58, 123.56, 123.53, 123.50, 120.61, 118.44, 118.41, 118.23, 118.20, 115.8, 115.7, 115.6, 115.5, 81.5, 81.4, 72.4, 71.4, 56.6, 56.6, 53.4, 53.2, 48.0, 47.7, 36.4, 35.9, 28.0, 28.0, 25.6, 18.1, −4.4, −4.4.

#### *tert*-Butyl ((2*S*)-4-(4-((*tert*-Butyldimethylsilyl)­oxy)­phenyl)-1-(((2*S*)-1-((2-(4-fluoro-3-nitrophenyl)-2-hydroxyethyl)­amino)-1-oxo-3-phenylpropan-2-yl)­amino)-1-oxobutan-2-yl)­carbamate (**14c**)

**9c** (1.74 g, 3.90 mmol) was dissolved in acetonitrile (40 mL), and 36% aq. HCl solution (9.0 mL) was added to it portionwise over 20 min at room temperature, and stirring was continued for another 20 min. The reaction mixture was then concentrated under reduced pressure, coevaporated with toluene (10 mL), and further dried under high vacuum. Then compound **12** (1.76 g, 4.29 mmol), HATU (1.63 g, 4.30 mmol), and DIPEA (0.770 mL, 5.53 mmol) were added to DCM (30 mL), followed by the addition of another portion of DIPEA (0.770 mL, 5.53 mmol). LC–MS showed complete conversion after stirring at room temperature for 45 min. The reaction mixture was diluted with DCM (100 mL), washed with an aqueous 1 M HCl solution (2 × 50 mL), saturated aqueous sodium bicarbonate solution (2 × 50 mL), and brine (2 × 50 mL), dried over anhydrous Na_2_SO_4_, filtered, and concentrated under reduced pressure. The resulting residue was then loaded on a silica gel column and purified using 50% EtOAc in *n*-hexane as the mobile phase to give **14c** (2.5 g, 3.39 mmol, 86%) as a light-yellow foam. HRMS (ESI) *m*/*z*: calcd for C_38_H_51_FN_4_O_8_SiNa [M + Na]^+^, 761.3352; found, 761.3365. ^1^H NMR (500 MHz, DMSO-*d*_6_): δ 8.11–8.04 (m, 2H), 7.80–7.75 (m, 1H), 7.72–7.63 (m, 1H), 7.51–7.45 (m, 1H), 7.23–7.09 (m, 5H), 7.01–6.93 (m, 3H), 6.77–6.67 (m, 2H), 5.84–5.80 (m, 1H), 4.71–4.68 (m, 1H), 4.54–4.45 (m, 1H), 3.86–3.77 (m, 1H), 3.30–3.14 (m, 1H), 2.90–2.84 (m, 1H), 2.78–2.63 (m, 1H), 2.45–2.27 (m, 2H), 1.71–1.56 (m, 2H), 1.38 (s, 3H), 1.38 (s, 3H), 0.94 (s, 9H), 0.16 (s, 6H). ^13^C NMR (126 MHz, DMSO-*d*_6_): δ 171.6, 171.5, 171.1, 155.2, 154.6, 154.6, 153.0, 152.5, 152.5, 141.0, 141.0, 140.9, 140.9, 137.6, 136.4, 136.4, 136.4, 136.3, 134.2, 134.1, 134.1, 134.0, 134.0, 129.2, 129.1, 127.9, 126.1, 123.5, 123.4, 123.4, 123.3, 119.5, 118.0, 117.8, 79.1, 78.2, 69.7, 69.6, 54.3, 54.2, 53.5, 53.3, 46.0, 46.0, 40.1, 40.0, 39.9, 39.8, 39.7, 39.6, 39.6, 39.5, 39.4, 39.3, 39.1, 39.0, 37.8, 37.6, 34.0, 34.0, 30.6, 28.1, 27.8, 25.5, 17.9, −4.5.

#### *tert*-Butyl ((2*S*)-3-(4-((*tert*-Butyldimethylsilyl)­oxy)­phenyl)-1-(((2*S*)-4-((2-(4-fluoro-3-nitrophenyl)-2-hydroxyethyl)­amino)-3-oxo-1-phenylbutan-2-yl)­amino)-1-oxopropan-2-yl)­carbamate (**15c**)

**10c** (1.73 g, 3.77 mmol) was dissolved in acetonitrile (40 mL), and 36% aq. HCl solution (9.0 mL) was added to it portionwise over 20 min at room temperature, and stirring was continued for another 20 min. The reaction mixture was then concentrated under reduced pressure, coevaporated with toluene (10 mL), and further dried under high vacuum. Then compound **11** (1.64 g, 4.14 mmol), HATU (1.56 g, 4.14 mmol), and DIPEA (0.576 mL, 5.53 mmol) were added to DCM (30 mL), followed by the addition of another portion of DIPEA (0.576 mL, 5.53 mmol). LC–MS showed complete conversion after stirring at room temperature for 45 min. The reaction mixture was diluted with DCM (100 mL), washed with an aqueous 1 M HCl solution (2 × 50 mL), saturated aqueous sodium bicarbonate solution (2 × 50 mL), and brine (2 × 50 mL), dried over anhydrous Na_2_SO_4_, filtered, and concentrated under reduced pressure. The resulting residue was then loaded on a silica gel column and purified using 75% EtOAc in *n*-hexane as the mobile phase to give **15c** (1.95 g, 2.63 mmol, 70%) as a light-yellow foam. HRMS (ESI) *m*/*z*: calcd for C_38_H_51_FN_4_O_8_SiNa [M + Na]^+^, 761.3352; found, 761.3364. ^1^H NMR (500 MHz, DMSO-*d*_6_): δ 8.09–8.07 (m, 1H), 7.96–7.93 (m, 1H), 7.82–7.78 (m, 1H), 7.76–7.72 (m, 1H), 7.54–7.50 (m, 1H), 7.25–7.22 (m, 2H), 7.18–7.13 (m, 1H), 7.13–7.11 (m, 2H), 7.05–7.03 (m, 2H), 6.78–6.75 (m, 1H), 6.73–6.64 (m, 2H), 5.81–5.79 (m, 1H), 4.78–4.67 (m, 1H), 4.20–4.13 (m, 1H), 4.01–3.97 (m, 1H), 3.40–3.32 (m, 1H), 3.27–3.18 (m, 1H), 2.74–2.70 (m, 1H), 2.62–2.60 (m, 2H), 2.23–2.09 (m, 2H), 1.29 (s, 6H), 0.92 (s, 9H), 0.14 (s, 6H). ^13^C NMR (126 MHz, DMSO-*d*_6_): δ 170.8, 170.1, 155.0, 154.6, 153.3, 152.5, 152.5, 141.1, 141.1, 138.4, 138.4, 136.4, 136.3, 134.0, 134.0, 134.0, 133.9, 131.0, 130.1, 129.2, 128.0, 125.9, 123.4, 123.3, 119.2, 118.1, 118.1, 118.0, 117.9, 77.8, 69.8, 69.8, 55.9, 47.6, 46.0, 45.9, 36.8, 28.1, 25.5, 17.9, −4.5.

#### *tert*-Butyl ((2*S*)-3-(4-((*tert*-Butyldimethylsilyl)­oxy)­phenyl)-1-(((3*S*)-1-((2-(4-fluoro-3-nitrophenyl)-2-hydroxyethyl)­amino)-5-methyl-2-oxohexan-3-yl)­amino)-1-oxopropan-2-yl)­carbamate (**15e**)

**10e** (1.05 g, 2.45 mmol) was dissolved in acetonitrile (30 mL), and 36% aq. HCl solution (8.0 mL) was added to it portionwise over 20 min at room temperature, and stirring was continued for another 20 min. The reaction mixture was then concentrated under reduced pressure, coevaporated with toluene (10 mL), and further dried under high vacuum. Then compound **11** (1.07 g, 2.72 mmol), HATU (1.02 g, 2.72 mmol), and DIPEA (0.283 mL, 2.72 mmol) were added to DCM (30 mL), followed by the addition of another portion of DIPEA (0.283 mL, 2.72 mmol). LC–MS showed complete conversion after stirring at room temperature for 45 min. The reaction mixture was diluted with DCM (100 mL), washed with an aqueous 1 M HCl solution (2 × 50 mL), saturated aqueous sodium bicarbonate solution (2 × 50 mL), and brine (2 × 50 mL), dried over anhydrous Na_2_SO_4_, filtered, and concentrated under reduced pressure. The resulting residue was then loaded on a silica gel column and purified using 75% EtOAc in *n*-hexane as the mobile phase to give **15e** (1.31 g, 1.86 mmol, 76%) as a light-yellow foam. ^1^H NMR (500 MHz, DMSO-*d*_6_): δ 8.09–8.06 (m, 2H), 7.92–7.87 (m, 2H), 7.76–7.73 (m, 2H), 7.66–7.61 (m, 2H), 7.56–7.52 (m, 2H), 7.09–7.07 (m, 3H), 6.85–6.81 (m, 2H), 6.72–6.70 (m, 3H), 5.80–5.78 (m, 2H), 4.73–4.69 (m, 2H), 4.11–3.93 (m, 3H), 3.38–3.30 (m, 6H), 3.24–3.19 (m, 2H), 2.82–2.80 (m, 2H), 2.68–2.57 (m, 2H), 2.17–2.02 (m, 3H), 1.28–1.15 (m, 5H), 0.80–0.74 (m, 9H), 0.15 (s, 9H). ^13^C NMR (126 MHz, DMSO-*d*_6_): δ 170.7, 170.7, 170.2, 170.2, 155.1, 154.6, 154.6, 153.4, 152.6, 152.6, 141.2, 141.1, 141.1, 136.4, 136.3, 134.1, 134.1, 131.0, 130.2, 123.5, 123.5, 123.4, 119.3, 118.1, 118.1, 118.0, 117.9, 79.2, 77.9, 69.9, 69.8, 56.0, 56.0, 45.8, 44.0, 42.8, 42.8, 41.2, 40.1, 40.0, 39.9, 39.9, 39.8, 39.7, 39.6, 39.5, 39.4, 39.4, 39.2, 39.0, 36.6, 28.1, 27.8, 25.6, 23.9, 23.9, 23.3, 21.5, 21.4, 17.9, −4.6, −4.6.

#### *tert*-Butyl ((8*S*,11*S*)-4-Hydroxy-8-isobutyl-32-nitro-7,10-dioxo-2-oxa-6,9-diaza-1,3­(1,4)-dibenzenacyclododecaphane-11-yl)­carbamate (**16e**)

Compound **13e** (3.47 g, 5.02 mmol) was dissolved in DMF (420 mL), and CsF (12.2 g, 80.30 mmol) was added portionwise over 45 min and stirred at 50 °C. LC–MS showed complete conversion after 45 min. DMF was evaporated under reduced pressure at 70 °C, and ethyl acetate (100 mL) was added and washed with water (1 × 40 mL), concentrated under reduced pressure, and purified via column chromatography using 80% EtOAc in *n*-hexane as the mobile phase to give **16e** (2.37 g, 4.26 mmol, 84%) as a light-yellow viscous oil. HRMS (ESI) *m*/*z*: calcd for C_28_H_37_N_4_O_8_FSi [M + H]^+^, 557.2606; found, 557.2608. ^1^H NMR (500 MHz, DMSO-*d*_6_): δ 8.31 (s, 1H), 7.90–7.82 (m, 3H), 7.73 (d, 1H), 7.48 (d, *J* = 8.2 Hz, 1H), 7.32 (d, *J* = 7.8 Hz, 1H), 7.24–7.05 (m, 8H), 6.95 (s, 4H), 6.36 (d, *J* = 7.1 Hz, 1H), 6.25 (d, *J* = 7.4 Hz, 1H), 5.80 (d, *J* = 4.5 Hz, 1H), 5.75 (d, *J* = 4.5 Hz, 1H), 4.96 (s, 1H), 4.55–4.47 (m, 1H), 4.11–4.02 (m, 1H), 4.00–3.91 (m, 2H), 3.76–3.70 (m, 1H), 3.60–3.42 (m, 3H), 2.96–2.89 (m, 2H), 2.84–2.62 (m, 5H), 2.55–2.52 (m, 1H), 1.45–1.34 (m, 19H), 1.24–1.16 (m, 2H), 0.83–0.72 (m, 6H), 0.67–0.63 (m, 5H). ^13^C NMR (126 MHz, DMSO-*d*_6_): δ 170.5, 169.5, 168.9, 168.8, 158.8, 154.7, 142.4, 139.9, 134.4, 134.4, 132.8, 132.7, 130.9, 124.4, 121.7, 121.4, 121.3, 79.1, 78.0, 70.0, 69.3, 55.9, 50.2, 50.0, 42.8, 42.5, 35.8, 28.1, 23.4, 23.2, 23.2, 23.1, 22.7, 22.7.

#### *tert*-Butyl ((8*S*,11*S*)-4-Hydroxy-8-(4-methylbenzyl)-32-nitro-7,10-dioxo-2-oxa-6,9-diaza-1,3­(1,4)-dibenzenacyclododecaphane-11-yl)­carbamate (**16h**)

**13h** (1.47 g, 1.98 mmol) was dissolved in DMF (198 mL), and CsF (4.81 g, 31.68 mmol) was added portionwise over 45 min and stirred at 50 °C. LC–MS showed complete conversion after 45 min. DMF was evaporated under reduced pressure at 70 °C, and ethyl acetate (100 mL) was added and washed with water (1 × 40 mL), concentrated under reduced pressure, and purified via column chromatography using 30% EtOAc in *n*-hexane to 80% EtOAc in *n*-hexane as the mobile phase to give **16h** (805 mg, 1.33 mmol, 67%) as a light-yellow viscous oil. HRMS (ESI) *m*/*z*: calcd for C_32_H_36_N_4_O_8_Na [M + Na]^+^, 627.2425; found, 627.2440. ^1^H NMR (400 MHz, DMSO-*d*_6_): δ 7.94–7.83 (m, 3H), 7.54–7.44 (m, 1H), 7.37–7.30 (m, 1H), 7.27–7.03 (m, 7H), 6.95–6.75 (m, 8H), 6.23–6.21 (m, 1H), 6.11–6.10 (m, 1H), 5.82–5.78 (m, 2H), 4.98–4.96 (m, 1H), 4.55–4.50 (m, 1H), 4.17–4.06 (m, 2H), 3.71–3.69 (m, 1H), 3.60–3.58 (m, 1H), 3.02–2.61 (m, 9H), 2.19 (2s, 6H), 1.43 (s, 14H). ^13^C NMR (101 MHz, DMSO-*d*_6_): δ 169.9, 169.7, 167.6, 159.2, 159.0, 155.1, 152.8, 152.4, 142.8, 140.5, 140.1, 135.4, 135.2, 134.6, 134.6, 133.6, 133.3, 133.1, 131.2, 130.1, 130.0, 128.7, 128.6, 122.3, 121.8, 79.6, 78.5, 70.6, 52.8, 52.6, 37.2, 36.9, 36.6, 28.6, 21.1.

#### *tert*-Butyl ((8*S*,11*S*)-8-(4-Fluorobenzyl)-4-hydroxy-32-nitro-7,10-dioxo-2-oxa-6,9-diaza-1,3­(1,4)-dibenzenacyclododecaphane-11-yl)­carbamate (**16i**)

**13i** (1.00 g, 1.34 mmol) was dissolved in DMF (134 mL), and CsF (2.04 g, 13.46 mmol) was added portionwise over 45 min and stirred at 50 °C. LC–MS showed complete conversion after 45 min. DMF was evaporated under reduced pressure at 70 °C, and ethyl acetate (100 mL) was added and washed with water (1 × 40 mL), concentrated under reduced pressure, and purified via column chromatography using 30% EtOAc in *n*-hexane to 70% EtOAc in *n*-hexane as the mobile phase to give **16i** (538 mg, 0.883 mmol, 66%) as a light-yellow viscous oil. ^1^H NMR (500 MHz, DMSO-*d*_6_): δ 8.06–7.74 (m, 2H), 7.57–6.69 (m, 10H), 6.26–6.31 (m, 1H), 6.24–6.12 (m, 1H), 5.85–5.76 (m, 1H), 4.98–4.97 (m, 1H), 4.52–4.51 (m, 1H), 4.18 (s, 1H), 3.84–3.50 (m, 2H), 3.02–2.61 (m, 5H), 1.42 (d, *J* = 1.9 Hz, 7H). ^13^C NMR (126 MHz, DMSO-*d*_6_): δ 170.2, 169.9, 168.4, 167.5, 162.4, 162.3, 160.5, 160.4, 159.2, 159.0, 155.3, 152.8, 152.4, 142.8, 142.5, 140.5, 140.1, 134.6, 134.6, 133.3, 133.1, 133.0, 133.0, 132.6, 132.0, 131.9, 131.9, 131.3, 130.4, 124.7, 122.3, 121.8, 118.5, 115.2, 114.7, 114.6, 114.6, 114.5, 79.6, 78.5, 70.6, 70.3, 69.8, 56.6, 56.1, 52.7, 52.5, 45.6, 37.0, 36.7, 36.3, 28.6, 28.6, 28.5.

#### *tert*-Butyl ((8*S*,11*S*)-8-Benzyl-4-hydroxy-32-nitro-7,10-dioxo-2-oxa-6,9-diaza-1,3­(1,4)-dibenzenacyclotridecaphane-11-yl)­carbamate (**17c**)

**14c** (2.50 g, 3.38 mmol) was dissolved in DMF (338 mL), and CsF (8.19 g, 54.0 mmol) was added portionwise over 45 min and stirred at 50 °C. LC–MS showed complete conversion after 45 min. DMF was evaporated under reduced pressure at 70 °C, and ethyl acetate (100 mL) was added and washed with water (1 × 40 mL), concentrated under reduced pressure, and purified via column chromatography using 50% EtOAc in *n*-hexane to 100% EtOAc in *n*-hexane as the mobile phase and subsequently on HPLC using 35% acetonitrile/water to 85% acetonitrile water to give **17c** (776 mg, 1.28 mmol, 38%) as a colorless powder. HRMS (ESI) *m*/*z*: calcd for C_32_H_36_N_4_O_8_Na [M + Na]^+^, 627.2425; found, 627.2440. ^1^H NMR (500 MHz, DMSO-*d*_6_): δ 7.98–7.95 (m, 1H), 7.89–7.87 (m, 2H), 7.42–7.40 (m, 1H), 7.29–7.27 (m, 1H), 7.21–7.05 (m, 10H), 7.05–6.94 (m, 7H), 6.90–6.88 (m, 1H), 6.79–6.77 (m, 1H), 6.68–6.66 (m, 1H), 6.60–6.58 (m, 1H), 5.86–5.85 (m, 1H), 5.75–5.74 (m, 1H), 4.91–4.89 (m, 1H), 4.56–4.54 (m, 1H), 4.20–4.09 (m, 2H), 3.74–3.69 (m, 1H), 3.46–3.45 (m, 1H), 3.05–3.01 (m, 1H), 2.93–2.65 (m, 6H), 2.42–2.32 (m, 2H), 1.88–1.69 (m, 4H), 1.43 (s, 9H), 1.41 (s, 9H). ^13^C NMR (126 MHz, DMSO-*d*_6_): δ 170.6, 169.9, 169.3, 157.3, 157.3, 156.4, 156.3, 151.9, 151.8, 142.0, 141.7, 139.9, 139.5, 139.0, 138.9, 137.3, 137.1, 132.9, 132.6, 130.8, 130.8, 129.7, 129.7, 128.3, 128.2, 126.7, 126.5, 123.0, 122.7, 122.3, 122.0, 121.9, 121.7, 78.9, 78.8, 69.8, 69.6, 53.4, 53.2, 52.1, 51.8, 45.6, 45.5, 32.6, 32.1, 32.0, 31.8, 28.6, 28.6, 28.2.

#### *tert*-Butyl ((9*S*,12*S*)-9-Benzyl-4-hydroxy-32-nitro-7,11-dioxo-2-oxa-6,10-diaza-1,3­(1,4)-dibenzenacyclotridecaphane-12-yl)­carbamate (**18c**)

**15c** (1.50 g, 2.02 mmol) was dissolved in DMF (202 mL), and CsF (4.85 g, 32.0 mmol) was added portionwise over 45 min and stirred at 50 °C. LC–MS showed complete conversion after 45 min. DMF was evaporated under reduced pressure at 70 °C, and ethyl acetate (100 mL) was added and washed with water (1 × 40 mL), concentrated under reduced pressure, and purified via column chromatography using 70% EtOAc in *n*-hexane to 5% MeOH in EtOAc as the mobile phase to give **18c** (732 mg, 1.21 mmol, 60%) as a light-yellow viscous oil. HRMS (ESI) *m*/*z*: calcd for C_32_H_36_N_4_O_8_Na [M + Na]^+^, 627.2425; found, 627.2445. ^1^H NMR (500 MHz, DMSO-*d*_6_): δ 7.88–7.86 (m, 1H), 7.63–7.56 (m, 1H), 7.53–7.47 (m, 1H), 7.35–7.31 (m, 1H), 7.17–7.09 (m, 4H), 7.07–7.01 (m, 3H), 7.01–6.93 (m, 3H), 6.91–6.81 (m, 2H), 5.82–5.80 (m, 1H), 5.66 (s, 1H), 4.95–4.93 (m, 1H), 4.56–4.54 (m, 1H), 3.96–3.74 (m, 3H), 3.45–3.36 (m, 1H), 3.00–2.94 (m, 1H), 2.89–2.84 (m, 1H), 2.82–2.69 (m, 3H), 2.69–2.59 (m, 1H), 2.25–2.26 (m, 1H), 1.92–1.87 (m, 1H), 1.69–1.64 (m, 1H), 1.42, 1.42 (2s, 9H), 0.76–0.68 (m, 1H).

^13^C NMR (126 MHz, DMSO-*d*_6_): δ 170.7, 170.4, 169.2, 168.7, 157.7, 154.7, 154.6, 150.9, 150.7, 142.2, 141.8, 140.4, 139.8, 137.2, 137.1, 133.4, 133.3, 132.9, 132.7, 130.6, 130.1, 129.9, 127.9, 127.6, 127.5, 125.7, 125.6, 123.6, 123.4, 122.3, 120.1, 120.0, 114.7, 79.1, 77.9, 77.8, 70.5, 70.2, 56.2, 55.8, 46.1, 45.9, 45.4, 44.9, 37.9, 37.8, 37.4, 28.2, 28.1.

#### *tert*-Butyl ((9*S*,12*S*)-4-Hydroxy-9-isobutyl-32-nitro-7,11-dioxo-2-oxa-6,10-diaza-1,3­(1,4)-dibenzenacyclotridecaphane-12-yl)­carbamate (**18e**)

**15e** (2.50 g, 3.54 mmol) was dissolved in DMF (354 mL), and CsF (8.53 g, 56.64 mmol) was added portionwise over 45 min and stirred at 50 °C. LC–MS showed complete conversion after 45 min. DMF was evaporated under reduced pressure at 70 °C, and ethyl acetate (100 mL) was added and washed with water (1 × 40 mL), concentrated under reduced pressure, and purified via column chromatography using 70% EtOAc in *n*-hexane to 5% MeOH in EtOAc as the mobile phase to give **18e** (1.21 g, 2.12 mmol, 60%) as a light-yellow viscous oil. HRMS (ESI) *m*/*z*: calcd for C_29_H_38_N_4_O_8_Na [M + Na]^+^, 593.2582; found, 593.2596. ^1^H NMR (500 MHz, DMSO-*d*_6_): δ 7.92–7.81 (m, 1H), 7.63–7.54 (m, 1H), 7.41–7.35 (m, 1H), 7.30–7.29 (m, 1H), 7.20–7.17 (m 1H), 7.08–7.09 (m, 2H), 7.01–6.99 (m, 1H), 6.96–6.84 (m, 2H), 5.82–5.81 (m, 1H), 5.70–5.69 (m, 1H), 4.95–4.93 (m, 1H), 4.63–4.59 (m, 1H), 3.85–3.70 (m, 2H), 3.08–3.04 (m, 1H), 2.93–2.84 (m, 1H), 2.83–2.66 (m, 2H), 1.76–1.71 (m, 1H), 1.56–1.51 (m, 1H), 1.36 (s, 9H), 1.02–0.96 (m, 1H), 0.88–0.57 (m, 8H). ^13^C NMR (126 MHz, DMSO-*d*_6_): δ 169.7, 169.4, 169.4, 169.3, 157.9, 157.8, 154.7, 150.9, 150.7, 142.3, 142.0, 140.6, 140.1, 133.6, 133.6, 132.9, 130.8, 130.7, 124.0, 122.3, 120.3, 120.2, 79.2, 77.9, 77.9, 70.6, 70.1, 56.3, 55.8, 45.4, 44.8, 43.1, 41.8, 41.4, 40.1, 40.0, 39.9, 39.9, 39.8, 39.7, 39.6, 39.5, 39.4, 39.4, 39.2, 39.0, 37.1, 28.1, 23.9, 23.3, 23.2, 22.2, 22.2.

#### *tert*-Butyl ((8*S*,11*S*)-8-Isobutyl-4,7,10-trioxo-2-oxa-6,9-diaza-1,3­(1,4)-dibenzenacyclododecaphane-11-yl)­carbamate (**19e**)

**16e** (2.33 g, 4.19 mmol) was added to Pd/C (10%, 1.12 g, 1.05 mmol Pd), followed by the addition of MeOH (15 mL) under an argon atmosphere. The atmosphere was exchanged to 1 atm. H_2_ via purging the system three times with hydrogen in a Parr hydrogenator. LC–MS analysis showed complete conversion after stirring at room temperature for 74 min. The reaction mixture was filtered through a pad of Celite, concentrated, and dried under reduced pressure. Then THF (19.2 mL) was added, followed by the addition of 50% aqueous hypophosphorus acid (3.82 mL, 70.6 mmol), Cu_2_O (10 mg, 69.9 μmol), and a sodium nitrite (446 mg, 6.46 mmol) solution in water (720 μL), and stirred at 50 °C. LC–MS showed complete conversion with formation of side products within 10 min. The reaction was allowed to cool to room temperature, and ethyl acetate (100 mL) was added. The organic phase was washed with saturated aqueous sodium bicarbonate solution (2 × 50 mL), brine (2 × 50 mL), dried over anhydrous Na_2_SO_4_, filtered, and concentrated under reduced pressure. The crude reaction mixture was dissolved in ethyl acetate (15 mL) and DMSO (3 mL), and IBX (45 wt %, 8.56 g, 13.8 mmol) was added portionwise over 60 min with stirring at 85 °C and monitoring by LC–MS. After 65 min, the reaction mixture was allowed to cool to room temperature and centrifuged (to separate undissolved IBX), and ethyl acetate was added (2 × 50 mL), washed with saturated aqueous sodium bicarbonate solution (2 × 50 mL), dried over anhydrous Na_2_SO_4_, and concentrated under reduced pressure. Purification on reverse phase HPLC, using a gradient from 30% to 80% acetonitrile in water, affords **19e** (155 mg, 0.30 mmol, 7% yield) as a colorless powder. HRMS (ESI) *m*/*z*: calcd for C_28_H_36_N_3_O_6_ [M + H]^+^, 510.2599; found, 510.2620. ^1^H NMR (400 MHz, CDCl_3_): δ 7.66 (d, *J* = 8.6 Hz, 2H), 7.34–7.28 (m, 1H), 6.97–6.91 (m, 2H), 6.47 (d, *J* = 7.1 Hz, 1H), 6.23 (d, *J* = 7.7 Hz, 1H), 5.67 (d, *J* = 9.6 Hz, 1H), 5.29 (dd, *J* = 15.1, 10.3 Hz, 1H), 5.00 (d, *J* = 8.9 Hz, 1H), 3.94–3.84 (m, 2H), 3.71 (q, *J* = 7.0 Hz, 1H), 3.49 (d, *J* = 15.2 Hz, 1H), 2.99 (t, *J* = 11.9 Hz, 1H), 2.89–2.82 (m, 1H), 1.65 (s, 2H), 1.56 (s, 1H), 1.43 (s, 9H), 1.24 (t, *J* = 7.0 Hz, 1H), 0.91–0.79 (m, 6H).

#### *tert*-Butyl ((8*S*,11*S*)-8-(4-Methylbenzyl)-4,7,10-trioxo-2-oxa-6,9-diaza-1,3­(1,4)-dibenzenacyclododecaphane-11-yl)­carbamate (**19h**)

**16h** (800 mg, 1.32 mmol) was added to Pd/C (10%, 1.00 g, 0.936 mmol Pd), followed by the addition of MeOH (5 mL) under an argon atmosphere. The atmosphere was exchanged to 1 atm. H_2_ via purging the system three times with hydrogen in a Parr hydrogenator. LC–MS analysis showed complete conversion after stirring at room temperature for 1 h. The reaction mixture was filtered through a pad of Celite, concentrated, and dried under reduced pressure. Then THF (6 mL) was added, followed by the addition of 50% aqueous hypophosphorus acid (1.20 mL, 22.2 mmol), Cu_2_O (3 mg, 22.05 μmol), and a sodium nitrite (140 mg, 2.03 mmol) solution in water (227 μL), and stirred at 50 °C. LC–MS showed complete conversion with formation of side products within 10 min. The reaction was allowed to cool to room temperature, and ethyl acetate (100 mL) was added. The organic phase was washed with saturated aqueous sodium bicarbonate solution (2 × 50 mL), brine (2 × 50 mL), dried over anhydrous Na_2_SO_4_, filtered, and concentrated under reduced pressure. The crude reaction mixture was dissolved in ethyl acetate (10 mL) and DMSO (3 mL), and IBX (45 wt %, 2.70 g, 4.35 mmol) was added portionwise over 60 min with stirring at 85 °C and monitoring by LC–MS. After 1 h, the reaction mixture was allowed to cool to room temperature and centrifuged (to separate undissolved IBX), and ethyl acetate was added (2 × 50 mL), washed with saturated aqueous sodium bicarbonate solution (2 × 50 mL), dried over anhydrous Na_2_SO_4_, and concentrated under reduced pressure. Purification on reverse phase HPLC, using a gradient from 25% to 70% acetonitrile in water, afforded **19h** (161 mg, 290 μmol, 22%) as a colorless powder. HRMS (ESI) *m*/*z*: calcd for C_32_H_35_N_3_O_6_Na [M + Na]^+^, 580.2418; found, 580.2436. ^1^H NMR (500 MHz, chloroform-*d*): δ 7.63–7.53 (m, 2H), 7.33–7.28 (m, 4H), 7.08 (d, *J* = 7.7 Hz, 2H), 7.00 (d, *J* = 7.6 Hz, 2H), 6.97–6.89 (m, 3H), 6.52–6.41 (m, 1H), 6.26 (d, *J* = 7.3 Hz, 1H), 5.14 (dd, *J* = 15.5, 10.3 Hz, 1H), 5.05 (d, *J* = 9.1 Hz, 1H), 4.80–4.71 (m, 1H), 3.97–3.92 (m, 1H), 3.82–3.78 (m, 1H), 3.15 (d, *J* = 15.7 Hz, 1H), 3.11–3.02 (m, 1H), 3.02–2.88 (m, 2H), 2.59 (t, *J* = 11.5 Hz, 1H), 2.33 (s, 3H), 1.49 (s, 9H). ^13^C NMR (126 MHz, CDCl_3_): δ 198.6, 169.9, 168.5, 165.2, 159.9, 154.9, 137.0, 133.2, 132.9, 131.7, 130.2, 129.9, 129.5, 129.4, 129.0, 123.2, 122.8, 121.5, 58.2, 56.2, 47.3, 39.3, 37.9, 28.3, 21.1.

#### *tert*-Butyl ((8*S*,11*S*)-8-(4-Fluorobenzyl)-4,7,10-trioxo-2-oxa-6,9-diaza-1,3­(1,4)-dibenzenacyclododecaphane-11-yl)­carbamate (**19i**)

**16i** (0.500 g, 0.820 mmol) was subjected to a similar hydrogenation, deamination, and oxidation sequence, as described for the synthesis of **19i,** and purified on reverse phase HPLC using a gradient from 30% to 70% acetonitrile in water to give **19i** (92 mg, 163 μmol, 20%) as a colorless powder. ^1^H NMR (500 MHz, CDCl_3_): δ 7.60 (d, *J* = 8.5 Hz, 2H), 7.33 (d, *J* = 7.8 Hz, 1H), 7.08 (dd, *J* = 8.0, 5.3 Hz, 2H), 7.01–6.91 (m, 5H), 6.46 (d, *J* = 8.3 Hz, 1H), 6.23 (d, *J* = 7.3 Hz, 1H), 5.17 (dd, *J* = 15.4, 10.2 Hz, 1H), 5.02 (d, *J* = 8.6 Hz, 1H), 4.86 (d, *J* = 10.3 Hz, 1H), 3.93 (s, 1H), 3.82 (s, 1H), 3.21 (d, *J* = 15.4 Hz, 1H), 3.00 (t, *J* = 12.9 Hz, 2H), 2.93 (dd, *J* = 12.3, 3.7 Hz, 1H), 2.69 (t, *J* = 11.3 Hz, 1H), 1.50 (s, 9H). ^13^C NMR (126 MHz, CDCl_3_): δ 198.3, 170.0, 168.3, 165.3, 163.0, 161.0, 159.9, 154.9, 133.2, 131.8, 131.7, 131.7, 130.7, 130.7, 130.3, 129.8, 129.6, 123.2, 122.8, 121.5, 115.7, 115.5, 80.3, 58.1, 55.9, 47.3, 38.9, 37.8, 29.7, 28.3.

#### *tert*-Butyl ((8*S*,11*S*)-8-Benzyl-4,7,10-trioxo-2-oxa-6,9-diaza-1,3­(1,4)-dibenzenacyclotridecaphane-11-yl)­carbamate (**20c**)

**17c** (750 mg, 1.24 mmol) was subjected to a similar hydrogenation, deamination, and oxidation sequence, as described for the synthesis of **19h,** and purified on reverse phase HPLC using a gradient from 40% to 90% acetonitrile in water to give **20c** (297 mg, 533 μmol, 43%) as a colorless powder. HRMS (ESI) *m*/*z*: calcd for C_32_H_35_N_3_O_6_Na [M + Na]^+^, 580.2418; found, 580.2424. ^1^H NMR (500 MHz, CDCl_3_): δ 7.71–7.64 (m, 1H), 7.26 (s, 5H), 7.14–7.08 (m, 3H), 7.02–6.90 (m, 3H), 5.31–5.15 (m, 2H), 4.61 (d, *J* = 8.5 Hz, 1H), 4.07–3.99 (m, 1H), 3.31 (d, *J* = 15.4 Hz, 1H), 3.13–3.06 (m, 1H), 3.03 (dt, *J* = 13.7, 4.0 Hz, 1H), 2.95 (dd, *J* = 12.8, 4.4 Hz, 1H), 2.78 (dd, *J* = 12.7, 10.1 Hz, 1H), 2.56 (td, *J* = 13.4, 4.1 Hz, 1H), 2.39 (t, *J* = 14.0 Hz, 1H), 1.72–1.60 (m, 2H), 1.54 (s, 9H). ^13^C NMR (126 MHz, CDCl_3_): δ 196.9, 171.8, 169.7, 165.5, 157.6, 156.5, 137.6, 136.4, 130.8, 130.8, 130.5, 129.2, 128.8, 128.8, 128.6, 127.2, 123.5, 120.2, 81.1, 56.9, 51.1, 47.4, 39.6, 31.0, 30.9, 28.4.

#### *tert*-Butyl ((9*S*,12*S*)-9-Benzyl-4,7,11-trioxo-2-oxa-6,10-diaza-1,3­(1,4)-dibenzenacyclotridecaphane-12-yl)­carbamate (**21c**)

**18c** (720 mg, 1.19 mmol) was subjected to a similar hydrogenation, deamination, and oxidation sequence, as described for the synthesis of **19h,** and purified on reverse phase HPLC using a gradient from 35% to 80% acetonitrile in water to give **21c** (218 mg, 392 μmol, 33%) as a colorless powder. HRMS (ESI) *m*/*z*: calcd for C_32_H_35_N_3_O_6_Na [M + Na]^+^, 580.2418; found, 580.2436. ^1^H NMR (500 MHz, chloroform-*d*): δ 7.76 (d, *J* = 8.2 Hz, 1H), 7.24 (dd, *J* = 12.4, 5.5 Hz, 2H), 7.20–7.12 (m, 3H), 6.97–6.90 (m, 4H), 6.72 (s, 1H), 5.41 (s, 1H), 4.98 (s, 1H), 4.51 (s, 1H), 4.31 (s, 1H), 4.21 (s, 1H), 3.22 (d, *J* = 12.7 Hz, 1H), 2.98 (t, *J* = 11.2 Hz, 1H), 2.74 (dd, *J* = 13.7, 5.3 Hz, 1H), 1.80 (dd, *J* = 15.8, 5.6 Hz, 1H), 1.52 (s, 9H). ^13^C NMR (126 MHz, CDCl_3_): δ 198.3, 170.1, 169.5, 165.5, 158.8, 155.2, 137.5, 134.1, 130.8, 129.5, 129.2, 128.7, 126.8, 123.2, 119.9, 80.5, 56.5, 47.4, 46.8, 38.8, 38.0, 37.3, 29.8, 28.5.

#### *tert*-Butyl ((9*S*,12*S*)-9-Isobutyl-4,7,11-trioxo-2-oxa-6,10-diaza-1,3­(1,4)-dibenzenacyclotridecaphane-12-yl)­carbamate (**21e**)

**18e** (750 mg, 1.31 mmol) was subjected to a similar hydrogenation, deamination, and oxidation sequence, as described for the synthesis of **19h,** and purified on reverse phase HPLC using a gradient from 30% to 90% acetonitrile in water to give **21e** (144 mg, 275 μmol, 21%) as a colorless powder. HRMS (ESI) *m*/*z*: calcd for C_29_H_37_N_3_O_6_Na [M + Na]^+^, 546.2575; found, 546.2574. ^1^H NMR (500 MHz, chloroform-*d*): δ 7.65 (d, *J* = 8.4 Hz, 2H), 7.18 (d, *J* = 8.0 Hz, 2H), 6.90 (d, *J* = 7.9 Hz, 2H), 6.80 (d, *J* = 8.3 Hz, 2H), 6.42 (d, *J* = 9.4 Hz, 1H), 5.45 (t, *J* = 6.4 Hz, 1H), 5.11 (d, *J* = 9.1 Hz, 1H), 4.50 (dd, *J* = 16.1, 6.5 Hz, 1H), 4.23 (tt, *J* = 10.7, 5.1 Hz, 2H), 4.07 (d, *J* = 8.5 Hz, 2H), 3.17–2.98 (m, 2H), 1.46 (s, 9H), 1.43–1.30 (m, 3H), 0.82 (d, *J* = 6.5 Hz, 6H). ^13^C NMR (126 MHz, CDCl_3_): δ 199.2, 170.1, 169.5, 165.4, 158.7, 155.3, 134.4, 130.7, 130.5, 129.6, 123.1, 119.9, 80.4, 77.4, 77.4, 77.2, 76.9, 59.7, 56.9, 47.7, 43.8, 42.9, 40.5, 38.3, 37.6, 31.4, 29.9, 28.5, 25.2, 22.9, 22.4.

#### *N*-((8*S*,11*S*)-8-Isobutyl-4,7,10-trioxo-2-oxa-6,9-diaza-1,3­(1,4)-dibenzenacyclododecaphane-11-yl)­acetamide (**1e**)

**19e** (40 mg, 78 μmol) was dissolved in acetonitrile (1 mL), and an aqueous conc. HCl solution (1.0 mL) was added. LC–MS showed complete conversion after stirring at room temperature for 30 min. The reaction mixture was concentrated under reduced pressure and dried by coevaporation with toluene (2 × 5 mL) at high vacuum for 1 h. This material was dissolved in DCM (1 mL), and TEA (64 μL, 0.46 mmol) and acetic anhydride (25 μL, 0.26 mmol) were added simultaneously. The reaction was complete after stirring at room temperature for 10 min, as shown by LC–MS. Ethyl acetate (50 mL) was added, washed with an aqueous 1 M HCl solution (30 mL), saturated aqueous sodium bicarbonate solution (2 × 50 mL), and brine (2 × 50 mL), concentrated under reduced pressure, and purified on reverse phase HPLC using a gradient from 10% to 60% acetonitrile in water to give **1e** (19 mg, 43 μmol, 56%) as a colorless powder. HRMS (ESI) *m*/*z*: calcd for C_25_H_29_N_3_O_5_Na [M + Na]^+^, 474.2005; found, 474.2010. ^1^H NMR (500 MHz, DMSO-*d*_6_): δ 8.59 (dd, *J* = 8.9, 2.8 Hz, 1H), 8.36 (d, *J* = 7.9 Hz, 1H), 7.59–7.53 (m, 2H), 7.31 (d, *J* = 36.3 Hz, 2H), 7.03 (dd, *J* = 8.7, 2.0 Hz, 2H), 6.92–6.84 (m, 1H), 6.79 (d, *J* = 8.4 Hz, 1H), 6.57 (s, 1H), 4.90 (dd, *J* = 15.8, 9.0 Hz, 1H), 4.16 (ddd, *J* = 11.5, 8.0, 3.5 Hz, 1H), 3.93–3.84 (m, 1H), 3.42 (dd, *J* = 15.8, 2.5 Hz, 1H), 2.88 (t, *J* = 12.0 Hz, 1H), 2.71 (dd, *J* = 12.0, 3.5 Hz, 1H), 1.81 (d, *J* = 2.1 Hz, 3H), 1.34–1.15 (m, 3H), 0.77 (dt, *J* = 6.0, 2.4 Hz, 6H). ^13^C NMR (126 MHz, DMSO-*d*_6_): δ 198.9, 169.8, 168.9, 168.6, 164.5, 159.0, 134.3, 132.0, 130.0, 122.9, 122.5, 121.1, 79.1, 55.9, 50.8, 47.3, 43.6, 40.1, 40.0, 39.9, 39.8, 39.7, 39.6, 39.6, 39.5, 39.4, 39.3, 39.1, 39.0, 36.0, 23.9, 22.8, 22.5, 22.2.

#### *N*-((8*S*,11*S*)-8-(4-Methylbenzyl)-4,7,10-trioxo-2-oxa-6,9-diaza-1,3­(1,4)-dibenzenacyclododecaphane-11-yl)­acetamide (**1h**)

**19h** (100 mg, 179 μmol) was subjected to Boc deprotection and acetylation similarly as described for the synthesis of **1e** and purified on reverse phase HPLC using a gradient from 10% to 65% acetonitrile in water to give **1h** (58 mg, 0.10 mmol, 65%) as a colorless powder. HRMS (ESI) *m*/*z*: calcd for C_29_H_29_N_3_O_6_Na [M + Na]^+^, 522.1999; found, 522.2010. ^1^H NMR (500 MHz, DMSO-*d*_6_): δ 8.36–8.31 (m, 1H), 8.28 (d, *J* = 8.1 Hz, 1H), 7.51 (d, *J* = 8.7 Hz, 2H), 7.34–7.20 (m, 2H), 7.07–7.02 (m, 2H), 6.97 (d, *J* = 7.8 Hz, 2H), 6.89–6.79 (m, 3H), 6.77 (d, *J* = 7.5 Hz, 1H), 6.63 (d, *J* = 8.3 Hz, 1H), 4.72 (dd, *J* = 15.9, 8.6 Hz, 1H), 4.22 (ddd, *J* = 12.0, 8.0, 4.1 Hz, 1H), 3.91 (td, *J* = 7.0, 5.2 Hz, 1H), 3.36 (d, *J* = 3.2 Hz, 1H), 2.83 (t, *J* = 11.9 Hz, 1H), 2.76–2.67 (m, 2H), 2.59 (dd, *J* = 13.3, 5.2 Hz, 1H), 2.22 (s, 3H), 1.87 (s, 3H). ^13^C NMR (126 MHz, DMSO-*d*_6_): δ 199.7, 169.4, 169.1, 168.9, 164.7, 159.6, 137.2, 136.6, 135.5, 134.5, 133.6, 132.0, 130.9, 130.4, 129.6, 128.8, 128.1, 127.4, 126.8, 123.2, 122.7, 121.6, 79.6, 56.1, 54.1, 47.9, 37.0, 22.8, 21.1.

#### *N*-((8*S*,11*S*)-8-(4-Fluorobenzyl)-4,7,10-trioxo-2-oxa-6,9-diaza-1,3­(1,4)-dibenzenacyclododecaphane-11-yl)­acetamide (**1i**)

**19i** (40 mg, 80 μmol) was subjected to Boc deprotection and acetylation similarly as described for the synthesis of **1e** and purified on reverse phase HPLC using a gradient from 10% to 65% acetonitrile in water to give **1i** (24 mg, 0.047 mmol, 69%) as a colorless powder. HRMS (ESI) *m*/*z*: calcd for C_28_H_27_FN_3_O_5_ [M + H]^+^, 504.1929; found, 504.1932. ^1^H NMR (500 MHz, DMSO-*d*_6_): δ 8.39 (dd, *J* = 8.6, 3.3 Hz, 1H), 8.27 (d, *J* = 7.9 Hz, 1H), 7.57–7.44 (m, 2H), 7.31 (s, 1H), 7.25 (s, 1H), 7.10–7.01 (m, 2H), 7.02–6.91 (m, 3H), 6.84 (s, 1H), 6.80 (d, *J* = 7.4 Hz, 1H), 6.63 (s, 1H), 4.74 (dd, *J* = 15.9, 8.6 Hz, 1H), 4.21 (ddd, *J* = 11.9, 7.9, 4.1 Hz, 1H), 3.98–3.88 (m, 1H), 3.38 (dd, *J* = 15.9, 3.2 Hz, 1H), 2.82 (t, *J* = 11.9 Hz, 1H), 2.79–2.67 (m, 2H), 2.67–2.56 (m, 1H), 1.87 (s, 3H). ^13^C NMR (126 MHz, DMSO-*d*_6_): δ 199.7, 169.5, 169.2, 168.8, 164.7, 162.4, 160.5, 159.6, 134.4, 132.9, 132.9, 132.0, 131.6, 131.5, 130.9, 130.5, 130.4, 123.2, 122.7, 121.7, 115.0, 114.8, 56.0, 53.9, 47.9, 38.5, 36.9, 22.8.

#### *N*-((8*S*,11*S*)-8-Isobutyl-4,7,10-trioxo-2-oxa-6,9-diaza-1,3­(1,4)-dibenzenacyclododecaphane-11-yl)­propionamide (**1k**)

**19e** (40 mg, 78 μmol) was dissolved in acetonitrile (1 mL), and an aqueous conc. HCl solution (0.5 mL) was added. LC–MS showed complete conversion after stirring at room temperature for 30 min. The reaction mixture was concentrated under reduced pressure and dried by coevaporation with toluene (2 × 5 mL) at high vacuum for 1 h. This material was dissolved in THF (1 mL), followed by the addition of TEA (0.655 mL, 4.68 mmol) and propionyl chloride (23 μL, 0.26 mmol). The reaction was complete after stirring at room temperature for 1 h, as shown by LC–MS. Ethyl acetate (50 mL) was added, washed with an aqueous 1 M HCl solution (30 mL), saturated aqueous sodium bicarbonate solution (2 × 50 mL), and brine (2 × 50 mL), concentrated under reduced pressure, and purified on reverse phase HPLC using a gradient from 10% to 60% acetonitrile in water to give **1k** (18 mg, 39 μmol, 51%) as a colorless powder. HRMS (ESI) *m*/*z*: calcd for C_26_H_31_N_3_O_5_Na [M + Na]^+^, 488.2166; found, 488.2158. ^1^H NMR (500 MHz, DMSO-*d*_6_): δ 8.59 (dd, *J* = 9.0, 2.8 Hz, 1H), 7.57 (d, *J* = 7.9 Hz, 2H), 7.34 (s, 1H), 7.29 (s, 1H), 7.07–6.97 (m, 2H), 6.89 (s, 1H), 6.69 (d, *J* = 8.5 Hz, 1H), 6.59 (s, 1H), 4.90 (dd, *J* = 15.7, 8.9 Hz, 1H), 4.15 (ddd, *J* = 11.8, 8.2, 3.6 Hz, 1H), 3.91 (q, *J* = 7.4 Hz, 1H), 3.43 (dd, *J* = 15.8, 2.7 Hz, 1H), 2.91 (t, *J* = 12.0 Hz, 1H), 2.70 (dd, *J* = 11.9, 3.6 Hz, 1H), 2.15–2.00 (m, 2H), 1.32–1.15 (m, 3H), 0.97 (td, *J* = 7.6, 1.9 Hz, 2H), 0.75 (t, *J* = 5.7 Hz, 4H). ^13^C NMR (126 MHz, DMSO): δ 198.8, 172.7, 169.9, 168.6, 164.5, 159.0, 134.4, 130.0, 130.0, 121.1, 79.1, 55.7, 50.6, 47.3, 43.7, 40.1, 40.0, 39.9, 39.8, 39.7, 39.6, 39.6, 39.5, 39.4, 39.3, 39.1, 39.0, 39.0, 35.8, 28.1, 23.8, 22.7, 22.6, 9.8.

#### *N*-((8*S*,11*S*)-8-Benzyl-4,7,10-trioxo-2-oxa-6,9-diaza-1,3­(1,4)-dibenzenacyclotridecaphane-11-yl)­acetamide (**2c**)

**20c** (33 mg, 60 μmol) was subjected to Boc deprotection and acetylation similarly as described for the synthesis of **1e** and purified on reverse phase HPLC using a gradient from 23% to 70% acetonitrile in water to give **2c** (18 mg, 37 μmol, 62%) as a colorless powder. HRMS (ESI) *m*/*z*: calcd for C_29_H_29_N_3_O_6_Na [M + Na]^+^, 522.1999; found, 522.2014. ^1^H NMR (500 MHz, DMSO-*d*_6_): δ 8.72 (dd, *J* = 9.4, 2.3 Hz, 1H), 8.26 (d, *J* = 7.4 Hz, 1H), 7.68–7.61 (m, 2H), 7.30 (d, *J* = 7.9 Hz, 1H), 7.22 (dd, *J* = 8.1, 6.5 Hz, 2H), 7.19–7.14 (m, 1H), 7.11 (d, *J* = 8.3 Hz, 2H), 7.07–7.00 (m, 5H), 4.97 (dd, *J* = 15.8, 9.2 Hz, 1H), 4.18–4.06 (m, 1H), 3.27 (dd, *J* = 15.8, 2.3 Hz, 1H), 3.03 (ddd, *J* = 9.9, 7.3, 2.0 Hz, 1H), 2.90 (dt, *J* = 13.3, 3.9 Hz, 1H), 2.79–2.65 (m, 2H), 2.37 (td, *J* = 13.3, 4.1 Hz, 1H), 1.99 (s, 3H), 1.96–1.85 (m, 1H), 1.83–1.71 (m, 1H). ^13^C NMR (126 MHz, DMSO-*d*_6_): δ 196.4, 171.1, 170.9, 169.3, 164.6, 156.7, 138.4, 136.7, 130.4, 130.3, 128.8, 128.6, 128.1, 126.3, 123.1, 119.6, 79.1, 54.4, 48.9, 47.1, 38.2, 30.2, 29.9, 22.3.

#### *N*-((9*S*,12*S*)-9-Benzyl-4,7,11-trioxo-2-oxa-6,10-diaza-1,3­(1,4)-dibenzenacyclotridecaphane-12-yl)­acetamide (**3c**)

**21c** (30 mg, 53 μmol) was subjected to Boc deprotection and acetylation similarly as described for the synthesis of **1e** and purified on reverse phase HPLC using a gradient from 10% to 60% acetonitrile in water to give **3c** (20 mg, 40 μmol, 76%) as a colorless powder. HRMS (ESI) *m*/*z*: calcd for C_29_H_29_N_3_O_6_Na [M + Na]^+^, 522.1999; found, 522.1997. ^1^H NMR (500 MHz, DMSO-*d*_6_): δ 8.23–8.14 (m, 2H), 7.69 (d, *J* = 8.6 Hz, 1H), 7.47 (d, *J* = 8.7 Hz, 2H), 7.16–7.06 (m, 6H), 6.96 (ddd, *J* = 13.3, 6.9, 2.0 Hz, 3H), 4.61–4.49 (m, 2H), 3.85–3.79 (m, 1H), 3.61 (dd, *J* = 16.7, 3.6 Hz, 1H), 2.85 (dd, *J* = 13.4, 5.7 Hz, 1H), 2.71–2.54 (m, 2H), 2.28 (dd, *J* = 13.4, 7.3 Hz, 1H), 1.85 (s, 3H), 0.66 (dd, *J* = 16.5, 2.6 Hz, 1H). ^13^C NMR (126 MHz, DMSO-*d*_6_): δ 201.4, 169.5, 168.5, 168.5, 162.6, 157.7, 137.6, 133.6, 131.6, 130.5, 129.6, 129.4, 127.6, 125.7, 121.4, 120.6, 52.7, 48.4, 44.7, 37.0, 22.4.

#### *N*-((9*S*,12*S*)-9-Isobutyl-4,7,11-trioxo-2-oxa-6,10-diaza-1,3­(1,4)-dibenzenacyclotridecaphane-12-yl)­acetamide (**3e**)

**21e** (30 mg, 57 μmol) was subjected to Boc deprotection and acetylation similarly as described for the synthesis of **1e** and purified on reverse phase HPLC using a gradient from 10% to 70% acetonitrile in water to give **3e** (16 mg, 35 μmol, 62%) as a colorless powder. HRMS (ESI) *m*/*z*: calcd for C_26_H_31_N_3_O_5_Na [M + Na]^+^, 488.2156; found, 488.2170. ^1^H NMR (500 MHz, DMSO-*d*_6_): δ 8.23 (d, *J* = 8.2 Hz, 1H), 8.12 (dd, *J* = 8.2, 3.8 Hz, 1H), 7.67 (d, *J* = 9.6 Hz, 1H), 7.44 (d, *J* = 8.6 Hz, 2H), 7.14 (dd, *J* = 10.5, 8.4 Hz, 4H), 7.06–6.94 (m, 2H), 4.55 (ddd, *J* = 11.6, 8.2, 5.6 Hz, 1H), 4.46 (dd, *J* = 16.7, 8.1 Hz, 1H), 3.80 (tdd, *J* = 13.2, 10.3, 3.1 Hz, 1H), 3.59 (dd, *J* = 16.7, 3.7 Hz, 1H), 2.89 (dd, *J* = 13.5, 5.6 Hz, 1H), 2.78–2.59 (m, 2H), 1.83 (s, 3H), 1.68 (dd, *J* = 15.8, 11.4 Hz, 1H), 1.40–1.32 (m, 1H), 0.95 (ddd, *J* = 14.1, 10.3, 4.1 Hz, 1H), 0.83 (ddd, *J* = 13.2, 9.6, 3.4 Hz, 1H), 0.72 (dd, *J* = 6.6, 3.6 Hz, 6H), 0.51 (dd, *J* = 15.8, 2.7 Hz, 1H). ^13^C NMR (126 MHz, DMSO): δ 201.8, 169.6, 168.6, 168.3, 162.5, 157.9, 133.6, 132.0, 130.5, 129.4, 121.5, 120.8, 79.2, 52.9, 48.6, 44.3, 42.0, 41.8, 40.1, 40.0, 39.9, 39.9, 39.8, 39.7, 39.6, 39.5, 39.4, 39.4, 39.2, 39.1, 39.0, 37.2, 24.0, 23.5, 22.4, 21.7.

#### (8*S*,11*S*)-11-(Dimethylamino)-8-isopentyl-2-oxa-6,9-diaza-1,3­(1,4)-dibenzenacyclododecaphane-4,7,10-trione (**4a**)

**19a** (27 mg, 51 μmol) was dissolved in acetonitrile (1 mL), and an aqueous HCl solution (37%, 2 mL) was added to it. LC–MS showed complete Boc deprotection within 30 min. The reaction mixture was concentrated under reduced pressure and dried by coevaporation with toluene (2 × 10 mL) at high vacuum for 1 h. Sodium citrate buffer (pH 4.8; 5 mL) was then added to this dried material, followed by the addition of formaldehyde (30% w/v solution in water; 327 μL, 2.99 mmol), and stirred at room temperature for 10 min before the addition of sodium cyanoborohydride (4.71 mg, 0.075 mmol). The reaction was completed after stirring at room temperature for 3–4 min, monitored by LC–MS, and subsequently quenched with saturated aqueous sodium bicarbonate (10 mL). Ethyl acetate (25 mL) was added to the reaction, washed with saturated aqueous sodium bicarbonate solution (2 × 10 mL), concentrated under reduced pressure, and purified on reverse phase HPLC using a gradient from 10% to 65% acetonitrile in water to give **4a** (15.7 mg, 34.9 μmol, 35%) as a colorless powder. HRMS (ESI) *m*/*z*: calcd for C_26_H_34_N_3_O_4_ [M + H]^+^, 452.2549; found, 452.2544. ^1^H NMR (400 MHz, CDCl_3_): δ 7.65 (d, *J* = 8.7 Hz, 2H), 7.32–7.15 (m, 2H), 6.93 (d, *J* = 8.4 Hz, 2H), 6.84–6.75 (m, 1H), 6.40–6.38 (br s, 1H), 6.26 (d, *J* = 10.2 Hz, 1H), 6.01 (s, 1H), 5.26 (dd, *J* = 15.3, 10.4 Hz, 1H), 3.94–3.90 (m, 1H), 3.51 (dd, *J* = 15.3, 2.4 Hz, 1H), 3.14–3.01 (m, 1H), 2.91–2.78 (m, 2H), 2.42 (s, 6H), 1.75–1.51 (m, 2H), 1.10–1.02 (m, 2H), 0.81 (dd, *J* = 6.6, 3.1 Hz, 6H). ^13^C NMR (101 MHz, CDCl_3_): δ 198.2, 169.6, 165.5, 159.7, 131.9, 130.5, 129.7, 129.4, 122.9, 122.5, 121.6, 53.6, 47.5, 41.9, 34.7, 33.7, 32.2, 29.6, 27.9, 22.5, 22.2.

#### (8*S*,11*S*)-11-(Dimethylamino)-8-phenethyl-2-oxa-6,9-diaza-1,3­(1,4)-dibenzenacyclododecaphane-4,7,10-trione (**4b**)

**19b** (61 mg, 110 μmol) was dissolved in acetonitrile (2 mL), and an aqueous HCl solution (37%, 4 mL) was added to it. LC–MS showed complete Boc deprotection within 30 min. The reaction mixture was concentrated under reduced pressure and dried by coevaporation with toluene (2 × 10 mL) at high vacuum for 1 h. Sodium citrate buffer (pH 4.8; 10 mL) was then added to this dried material, followed by the addition of formaldehyde (30% w/v solution in water; 0.6 mL, 6.00 mmol), and stirred at room temperature for 10 min before the addition of sodium cyanoborohydride (9.42 mg, 0.150 mmol). The reaction was completed after stirring at room temperature for 3–4 min, monitored by LC–MS, and subsequently quenched with saturated aqueous sodium bicarbonate (20 mL). Ethyl acetate (50 mL) was added to the reaction, washed with saturated aqueous sodium bicarbonate solution (2 × 10 mL), concentrated under reduced pressure, and purified on reverse phase HPLC using a gradient from 10% to 65% acetonitrile in water to give **4b** (18.4 mg, 38.5 μmol, 35%) as a colorless powder. HRMS (ESI) *m*/*z*: calcd for C_29_H_32_N_3_O_4_ [M + H]^+^, 486.2393; found, 486.2406. ^1^H NMR (400 MHz, CDCl_3_): δ 7.63 (d, *J* = 8.4 Hz, 2H), 7.26–7.14 (m, 5H), 7.07 (d, *J* = 7.4 Hz, 2H), 6.92 (d, *J* = 8.4 Hz, 2H), 6.81 (d, *J* = 8.1 Hz, 1H), 6.40 (br s, 1H), 6.09–5.93 (m, 2H), 5.24 (dd, *J* = 15.3, 10.5 Hz, 1H), 4.00–3.94 (m, 1H), 3.45 (dd, *J* = 15.3, 2.6 Hz, 1H), 3.11 (t, *J* = 11.7 Hz, 1H), 2.84 (t, *J* = 15.7 Hz, 2H), 2.65–2.47 (m, 2H), 2.44 (s, 6H), 2.05–1.91 (m, 2H). ^13^C NMR (101 MHz, CDCl_3_): δ 198.1, 169.3, 165.5, 159.7, 140.1, 131.9, 130.4, 129.6, 129.4, 128.6, 128.1, 126.3, 122.9, 122.5, 121.6, 72.7, 53.3, 47.5, 42.0, 35.4, 34.6, 31.1, 29.6.

#### (8*S*,11*S*)-8-Benzyl-11-(dimethylamino)-2-oxa-6,9-diaza-1,3­(1,4)-dibenzenacyclododecaphane-4,7,10-trione (**4c**)

**19c** (50 mg, 90 μmol) was dissolved in acetonitrile (2 mL), and an aqueous HCl solution (36%, 4 mL) was added to it. LC–MS showed complete Boc deprotection within 30 min. The reaction mixture was concentrated under reduced pressure and dried by coevaporation with toluene (2 × 10 mL) at high vacuum for 1 h. Sodium citrate buffer (pH 4.8; 10 mL) was then added to this dried material, followed by the addition of formaldehyde (30% w/v solution in water; 0.491 mL, 4.91 mmol), and stirred at room temperature for 10 min before the addition of sodium cyanoborohydride (7.72 mg, 0.122 mmol). The reaction was completed after stirring at room temperature for 3–4 min, monitored by LC–MS, and subsequently quenched with saturated aqueous sodium bicarbonate (20 mL). Ethyl acetate (50 mL) was added to the reaction, washed with saturated aqueous sodium bicarbonate solution (2 × 10 mL), concentrated under reduced pressure, and purified on reverse phase HPLC using a gradient from 10% to 65% acetonitrile in water to give **4c** (28 mg, 59 μmol, 67%) as a colorless powder. HRMS (ESI) *m*/*z*: calcd for C_28_H_30_N_3_O_4_ [M + H]^+^, 472.2236; found, 472.2241. ^1^H NMR (400 MHz, CDCl_3_): δ 7.54 (d, *J* = 8.9 Hz, 2H), 7.32–7.17 (m, 6H), 7.17–7.07 (m, 2H), 6.92 (dd, *J* = 20.9, 8.4 Hz, 2H), 6.42 (d, *J* = 8.6 Hz, 1H), 6.11 (br s, 1H), 5.09 (dd, *J* = 15.5, 10.4 Hz, 1H), 4.72 (dd, *J* = 10.5, 2.7 Hz, 1H), 3.90–3.85 (m, 1H), 3.28–2.99 (m, 3H), 2.89 (t, *J* = 13.5 Hz, 2H), 2.64–2.54 (m, 1H), 2.47 (s, 6H). ^13^C NMR (101 MHz, CDCl_3_): δ 198.3, 168.7, 165.4, 159.6, 136.1, 131.9, 130.1, 129.6, 129.4, 129.0, 128.8, 127.4, 123.1, 122.7, 121.4, 56.3, 47.3, 41.9, 40.6, 34.6.

#### (8*S*,11*S*)-11-(Dimethylamino)-8-isobutyl-2-oxa-6,9-diaza-1,3­(1,4)-dibenzenacyclododecaphane-4,7,10-trione (**4e**)

**19e** (58.1 mg, 110 μmol) was dissolved in acetonitrile (2 mL), and an aqueous HCl solution (37%, 4 mL) was added to it. LC–MS showed complete Boc deprotection within 30 min. The reaction mixture was concentrated under reduced pressure and dried by coevaporation with toluene (2 × 10 mL) at high vacuum for 1 h. Sodium citrate buffer (pH 4.8; 10 mL) was then added to this dried material, followed by the addition of formaldehyde (30% w/v solution in water; 0.6 mL, 6.00 mmol), and stirred at room temperature for 10 min before the addition of sodium cyanoborohydride (9.42 mg, 0.150 mmol). The reaction was completed after stirring at room temperature for 3–4 min, monitored by LC–MS, and subsequently quenched with saturated aqueous sodium bicarbonate (20 mL). Ethyl acetate (50 mL) was added to the reaction, washed with saturated aqueous sodium bicarbonate solution (2 × 10 mL), concentrated under reduced pressure, and purified on reverse phase HPLC using a gradient from 10% to 65% acetonitrile in water to give **4e** (14.8 mg, 33.8 μmol, 29%) as a colorless powder. HRMS (ESI) *m*/*z*: calcd for C_25_H_32_N_3_O_4_ [M + H]^+^, 438.2387; found, 438.2389. ^1^H NMR (500 MHz, CDCl_3_): δ 7.66–7.62 (m, 2H), 7.30–7.27 (m, 1H), 7.19–7.15 (m, 1H), 6.96–6.90 (m, 3H), 6.43 (d, *J* = 6.5 Hz, 1H), 5.87 (d, *J* = 6.5 Hz, 1H), 5.76 (d, *J* = 9.6 Hz, 1H), 5.32–5.25 (m, 1H), 3.99–3.93 (m, 1H), 3.53–3.47 (m, 1H), 3.13 (t, *J* = 11.7 Hz, 1H), 2.85–2.73 (m, 2H), 2.39 (s, 6H), 1.57–1.40 (m, 3H), 0.91–0.87 (m, 6H). ^13^C NMR (126 MHz, CDCl_3_): δ 198.2, 170.5, 167.7, 159.6, 135.3, 132.5, 130.5, 129.5, 129.4, 123.3, 122.7, 121.7, 77.3, 73.1, 52.4, 47.6, 44.5, 42.1, 34.1, 24.9, 23.0, 22.6.

#### (8*S*,11*S*)-11-(Dimethylamino)-8-phenyl-2-oxa-6,9-diaza-1,3­(1,4)-dibenzenacyclododecaphane-4,7,10-trione (**4j**)

**19j** (35 mg, 60 μmol) was dissolved in acetonitrile (1.5 mL), and an aqueous HCl solution (37%, 3 mL) was added to it. LC–MS showed complete Boc deprotection within 30 min. The reaction mixture was concentrated under reduced pressure and dried by coevaporation with toluene (2 × 10 mL) at high vacuum for 1 h. Sodium citrate buffer (pH 4.8; 5 mL) was then added to this dried material, followed by the addition of formaldehyde (30% w/v solution in water; 0.327 mL, 3.27 mmol), and stirred at room temperature for 10 min before the addition of sodium cyanoborohydride (5.14 mg, 82 μmol). The reaction was completed after stirring at room temperature for 3–4 min, monitored by LC–MS, and subsequently quenched with saturated aqueous sodium bicarbonate (20 mL). Ethyl acetate (50 mL) was added to the reaction, washed with saturated aqueous sodium bicarbonate solution (2 × 10 mL), concentrated under reduced pressure, and purified on reverse phase HPLC using a gradient from 10% to 65% acetonitrile in water to give **4j** (17 mg, 37 μmol, 57%) as a colorless powder. HRMS (ESI) *m*/*z*: calcd for C_27_H_27_N_3_O_4_ [M + H]^+^, 458.2080; found, 458.2085. ^1^H NMR (500 MHz, CDCl_3_): δ 7.66 (d, *J* = 9.0 Hz, 2H), 7.32–7.26 (m, 2H), 7.20–7.17 (m, 1H), 7.01 (dd, *J* = 19.7, 8.0 Hz, 3H), 6.57 (dd, *J* = 8.5, 2.5 Hz, 1H), 6.50 (d, *J* = 6.0 Hz, 1H), 5.61 (dd, *J* = 10.2, 2.9 Hz, 1H), 5.11 (dd, *J* = 15.5, 10.1 Hz, 1H), 4.63 (d, *J* = 6.0 Hz, 1H), 3.41 (dd, *J* = 15.5, 2.9 Hz, 1H), 3.07 (t, *J* = 11.2 Hz, 1H), 2.80 (t, *J* = 12.7 Hz, 2H), 2.20 (s, 6H). ^13^C NMR (126 MHz, CDCl_3_): δ 193.6, 163.3, 160.6, 154.9, 132.9, 127.4, 125.5, 125.4, 124.8, 124.1, 123.8, 122.3, 118.2, 117.8, 116.9, 52.6, 43.1, 37.3, 30.7, 20.4.

#### (8*S*,11*S*)-8-Isopentyl-*N*,*N*-dimethyl-4,7,10-trioxo-2-oxa-6,9-diaza-1,3­(1,4)-dibenzenacyclododecaphan-11-aminium 2,2,2-Trifluoroacetate (**22a**)

To **a**(8.0 mg, 17.7 μmol) was added 0.16 M TFA in CH_2_Cl_2_ (3 mL) and the solution was concentrated under reduced pressure on a rotary evaporator, then this operation was repeated twice. Water (3 mL) was then added to the residue and lyophilized. HRMS (ESI) *m*/*z*: calcd for C_26_H_34_N_3_O_4_ [M + H]^+^, 452.2549; found, 452.2541. ^1^H NMR (400 MHz, CDCl_3_): δ 7.69–7.57 (m, 2H), 7.27 (d, *J* = 5.3 Hz, 2H), 7.12 (d, *J* = 6.2 Hz, 1H), 6.87 (d, *J* = 8.3 Hz, 2H), 6.46 (d, *J* = 8.5 Hz, 1H), 6.07 (d, *J* = 10.1 Hz, 1H), 5.30 (dd, *J* = 15.4, 10.2 Hz, 1H), 4.05 (dd, *J* = 12.0, 3.3 Hz, 1H), 3.82–3.69 (m, 1H), 3.61–3.44 (m, 1H), 3.30–3.10 (m, 2H), 2.96 (s, 4H), 1.77–1.54 (m, 2H), 1.52–1.36 (m, 2H), 1.32–1.16 (m, 7H), 1.12–0.92 (m, 3H), 0.92–0.76 (m, 7H). ^13^C NMR (101 MHz, CDCl_3_): δ 199.0, 168.5, 165.4, 163.6, 161.8, 161.5, 160.6, 131.6, 130.4, 130.0, 129.9, 123.3, 121.6, 67.9, 54.8, 47.8, 33.8, 32.8, 31.1, 29.6, 27.8, 22.5, 22.1.

#### (8*S*,11*S*)-*N*,*N*-Dimethyl-4,7,10-trioxo-8-phenethyl-2-oxa-6,9-diaza-1,3­(1,4)-dibenzenacyclododecaphan-11-aminium 2,2,2-Trifluoroacetate (**22b**)

To **4b** (7.0 mg, 14.6 μmol) was added 0.16 M TFA in CH_2_Cl_2_ (3 mL) and the solution was concentrated under reduced pressure on a rotary evaporator, then this operation was repeated twice. Water (3 mL) was then added to the residue and lyophilized. HRMS (ESI) *m*/*z*: calcd for C_29_H_32_N_3_O_4_ [M + H]^+^, 486.2393; found, 486.2385. ^1^H NMR (500 MHz, CDCl_3_): δ 7.58–7.48 (m, 1H), 7.27–7.11 (m, 3H), 7.11–6.98 (m, 2H), 6.84–6.73 (m, 1H), 6.38 (dd, *J* = 8.7, 2.5 Hz, 1H), 5.58 (s, 1H), 5.20 (dd, *J* = 15.5, 10.5 Hz, 1H), 4.03 (dd, *J* = 12.1, 3.0 Hz, 1H), 3.76–3.67 (m, 1H), 3.36 (dd, *J* = 15.6, 2.3 Hz, 1H), 3.16 (t, *J* = 11.8 Hz, 1H), 3.02 (dd, *J* = 11.6, 3.0 Hz, 1H), 2.59–2.39 (m, 1H), 2.06–1.88 (m, 1H). ^13^C NMR (126 MHz, CDCl_3_): δ 198.6, 168.1, 165.4, 163.7, 160.7, 139.6, 131.5, 130.4, 130.0, 129.9, 129.7, 128.8, 128.0, 126.6, 123.6, 123.4, 121.6, 67.9, 54.4, 47.7, 34.1, 32.6, 31.2, 29.7.

#### (8*S*,11*S*)-8-Benzyl-*N*,*N*-dimethyl-4,7,10-trioxo-2-oxa-6,9-diaza-1,3­(1,4)-dibenzenacyclododecaphan-11-aminium 2,2,2-Trifluoroacetate (**22c**)

To **4c** (15.0 mg, 31.6 μmol) was added 0.16 M TFA in CH_2_Cl_2_ (3 mL) and the solution was concentrated under reduced pressure on a rotary evaporator, then this operation was repeated twice. Water (3 mL) was then added to the residue and lyophilized. HRMS (APCI) *m*/*z*: calcd for C_28_H_30_N_3_O_4_ [M + H]^+^, 472.2236; found, 472.2226. ^1^H NMR (400 MHz, CDCl_3_): δ 7.57–7.48 (m, 2H), 7.29–7.20 (m, 5H), 7.17–7.07 (m, 2H), 6.94–6.81 (m, 2H), 6.41 (d, *J* = 8.4 Hz, 1H), 5.41 (dd, *J* = 10.3, 2.5 Hz, 1H), 5.16 (dd, *J* = 15.6, 10.3 Hz, 1H), 4.04 (dd, *J* = 12.0, 3.1 Hz, 1H), 3.86 (ddd, *J* = 10.4, 6.2, 4.5 Hz, 1H), 3.29–3.15 (m, 2H), 3.1–3.01 (m, 2H), 2.91 (s, 5H), 2.68 (dd, *J* = 12.9, 10.1 Hz, 1H). ^13^C NMR (101 MHz, CDCl_3_): δ 199.3, 167.8, 165.3, 163.8, 162.5, 162.1, 161.8, 161.4, 160.6, 135.6, 131.5, 130.2, 130.0, 130.0, 129.8, 129.1, 128.7, 127.4, 123.5, 123.2, 121.5, 117.2, 114.3, 67.7, 56.7, 47.5, 39.7, 32.9, 29.6.

#### (8*S*,11*S*)-8-Isobutyl-*N*,*N*-dimethyl-4,7,10-trioxo-2-oxa-6,9-diaza-1,3­(1,4)-dibenzenacyclododecaphan-11-aminium 2,2,2-Trifluoroacetate (**22e**)

To **5e** (4.0 mg, 9.1 μmol) was added 0.16 M TFA in CH_2_Cl_2_ (1 mL) and the solution was concentrated under reduced pressure on a rotary evaporator, then this operation was repeated twice. Water (3 mL) was then added to the residue and lyophilized. HRMS (APCI) *m*/*z*: calcd for C_25_H_32_N_3_O_4_ [M + H]^+^, 438.2387; found, 438.2376. ^1^H NMR (500 MHz, CDCl_3_): δ 7.67 (d, *J* = 8.3 Hz, 2H), 7.38–7.28 (m, 2H), 6.99–6.86 (m, 3H), 6.47 (d, *J* = 7.6 Hz, 1H), 5.54 (d, *J* = 10.2 Hz, 1H), 5.32 (dd, *J* = 15.2, 10.7 Hz, 1H), 3.89 (d, *J* = 10.9 Hz, 1H), 3.84 (d, *J* = 6.2 Hz, 1H), 3.51 (d, *J* = 15.3 Hz, 1H), 3.22 (t, *J* = 11.4 Hz, 1H), 3.15 (d, *J* = 11.5 Hz, 1H), 2.92 (s, 4H), 1.48 (dd, *J* = 16.9, 6.2 Hz, 3H), 0.90 (t, *J* = 6.9 Hz, 5H). ^13^C NMR (126 MHz, CDCl_3_): δ 198.2, 169.0, 165.6, 163.4, 162.5, 162.2, 160.8, 131.9, 130.6, 130.3, 130.2, 129.8, 123.8, 123.6, 121.8, 68.3, 53.3, 47.7, 43.4, 32.2, 25.0, 23.1, 22.1.

#### (8*S*,11*S*)-*N*,*N*-Dimethyl-4,7,10-trioxo-8-phenyl-2-oxa-6,9-diaza-1,3­(1,4)-dibenzenacyclododecaphan-11-aminium 2,2,2-Trifluoroacetate (**22j**)

To 5j (5.0 mg, 10.8 μmol) was added 0.16 M TFA in CH_2_Cl_2_ (1 mL) and the solution was concentrated under reduced pressure on a rotary evaporator, then this operation was repeated twice. Water (3 mL) was then added to the residue and lyophilized. HRMS (APCI) *m*/*z*: calcd for C_27_H_27_N_3_O_4_ [M + H]^+^, 458.2080; found, 458.2076. ^1^H NMR (500 MHz, CDCl_3_): δ 7.62 (d, *J* = 8.5 Hz, 2H), 7.37–7.28 (m, 2H), 7.27–7.22 (m, 4H), 7.20 (d, *J* = 5.0 Hz, 2H), 6.84 (dd, *J* = 8.5, 2.3 Hz, 1H), 6.50 (dd, *J* = 8.4, 2.5 Hz, 1H), 5.56 (dd, *J* = 10.5, 2.8 Hz, 1H), 5.11 (dd, *J* = 15.4, 10.4 Hz, 1H), 4.42 (d, *J* = 5.4 Hz, 1H), 4.02 (dd, *J* = 11.0, 4.4 Hz, 1H), 3.30 (dd, *J* = 15.4, 2.8 Hz, 1H), 3.09–3.03 (m, 2H), 2.62 (s, 6H). ^13^C NMR (126 MHz, CDCl_3_): δ 198.5, 166.7, 165.2, 163.9, 161.8, 160.7, 136.7, 131.5, 130.3, 130.3, 130.1, 129.2, 129.1, 126.8, 123.4, 123.4, 121.7, 67.4, 58.2, 48.0, 33.5, 29.7, 25.1.

#### *tert*-Butyl ((2*S*)-1-((2-Hydroxy-2-phenylethyl)­amino)-5-methyl-1-oxohexan-2-yl)­carbamate (**24a**)

(*S*)-Boc-homoleucine-OH (**6a**) (922 mg, 3.76 mmol), **23** (516 mg, 3.76 mmol), and HATU (618 mg, 4.5 mmol) were dissolved in DCM (20 mL), followed by the addition of DIPEA (0.914 mL, 5.26 mmol). LC–MS showed complete conversion after stirring at room temperature for 40 min. The reaction mixture was diluted with DCM (100 mL), washed with 1 M HCl (1 × 100 mL), saturated aqueous sodium bicarbonate solution (2 × 100 mL), and brine (1 × 100 mL), dried over Na_2_SO_4_, filtered, and concentrated under reduced pressure. The resulting residue was purified on a silica gel column using 50% EtOAc in *n*-hexane as the mobile phase to give **24a** (822 mg, 2.25 mmol, 60% yield) as a colorless viscous liquid. HRMS (ESI) *m*/*z*: calcd for C_20_H_33_N_2_O_4_ [M + H]^+^, 365.2450; found, 365.2441. ^1^H NMR (400 MHz, DMSO-*d*_6_): δ 7.76–7.68 (m, 1H), 7.34–7.18 (m, 5H), 6.80–6.67 (m, 1H), 5.45–5.37 (m, 1H), 4.64–4.53 (m, 1H), 3.89–3.75 (m, 1H), 3.37–3.28 (m, 1H), 3.25–3.18 (m, 1H), 3.14–3.04 (m, 1H), 1.55–1.39 (m, 3H), 1.36 (s, 9H), 1.25 (s, 1H), 1.14–1.00 (m, 2H), 0.80 (d, *J* = 6.6 Hz, 6H). ^13^C NMR (101 MHz, DMSO-*d*_6_): δ 172.2, 155.2, 143.5, 127.9, 126.9, 126.0, 77.9, 71.2, 54.5, 46.5, 34.4, 30.1, 28.1, 27.2, 22.5, 22.3.

#### *tert*-Butyl ((2*S*)-1-((2-Hydroxy-2-phenylethyl)­amino)-1-oxo-4-phenylbutan-2-yl)­carbamate (**24b**)

(*S*)-Boc-homophenylalanine-OH (**6b**) (1.05 g, 3.75 mmol), **23** (516 mg, 3.76 mmol), and HATU (618 mg, 4.5 mmol) were dissolved in DCM (20 mL), followed by the addition of DIPEA (0.914 mL, 5.26 mmol). LC–MS showed complete conversion after stirring at room temperature for 40 min. The reaction mixture was diluted with DCM (100 mL), washed with 1 M HCl (1 × 100 mL), saturated aqueous sodium bicarbonate solution (2 × 100 mL), and brine (1 × 100 mL), dried over Na_2_SO_4_, filtered, and concentrated under reduced pressure. The resulting residue was purified on a silica gel column using 50% EtOAc in *n*-hexane as the mobile phase to give **24b** (1.37 g, 3.43 mmol, 91% yield) as a colorless viscous liquid. HRMS (ESI) *m*/*z*: calcd for C_23_H_31_N_2_O_4_ [M + H]^+^, 399.2284; found, 399.2290. ^1^H NMR (400 MHz, DMSO-*d*_6_): δ 7.83–7.65 (m, 1H), 7.39–7.09 (m, 10H), 6.98 (m, 1H), 5.50–5.39 (m, 1H), 4.69–4.51 (m, 1H), 3.98–3.84 (m, 1H), 3.46–3.29 (m, 1H), 3.25–3.21 (m, 1H), 3.16–3.04 (m, 1H), 1.89–1.63 (m, 2H), 1.40 (s, 9H). ^13^C NMR (101 MHz, DMSO-*d*_6_): δ 172.0, 155.3, 143.5, 143.5, 141.4, 128.2, 128.2, 128.2, 127.9, 127.9, 126.9, 126.0, 126.0, 125.7, 79.1, 78.0, 71.2, 71.1, 54.0, 54.0, 46.6, 46.5, 33.9, 33.9, 31.6, 31.5, 28.2.

#### *tert*-Butyl ((2*S*)-1-((2-Hydroxy-2-phenylethyl)­amino)-1-oxo-3-phenylpropan-2-yl)­carbamate (**24c**)

(*S*)-Boc-phenylalanine-OH (**6c**) (7.95 g, 29.9 mmol), **23** (2.74 g, 19.9 mmol), and HATU (4.9 g, 35.8 mmol) were dissolved in DCM (160 mL), followed by the addition of DIPEA (7.27 mL, 41.84 mmol). LC–MS showed complete conversion after stirring at room temperature for 40 min. The reaction mixture was diluted with DCM (400 mL), washed with 1 M HCl (1 × 200 mL), saturated aqueous sodium bicarbonate solution (2 × 200 mL), and brine (1 × 200 mL), dried over Na_2_SO_4_, filtered, and concentrated under reduced pressure. The resulting residue was purified on a silica gel column using 50% EtOAc in *n*-hexane as the mobile phase to give **24c** (5.52 g, 14.3 mmol, 72% yield) in the form of a colorless foam. HRMS (ESI) *m*/*z*: calcd for C_22_H_29_N_2_O_4_ [M + H]^+^, 385.2127; found, 385.2121. ^1^H NMR (400 MHz, CDCl_3_): δ 7.35–7.13 (m, 20H), 6.62–6.46 (m, 3H), 5.28–5.23 (m, 2H), 4.74–4.71 (m, 1H), 4.63–4.60 (m, 1H), 4.36–4.32 (m, 2H), 3.64–3.51 (m, 3H), 3.25–3.12 (m, 3H), 3.12–2.91 (m, 5H), 1.38 (2s, 18H). ^13^C NMR (101 MHz, CDCl_3_): δ 172.4, 172.2, 155.5, 141.5, 141.4, 136.7, 136.6, 129.3, 129.3, 128.6, 128.6, 128.4, 127.7, 127.7, 126.9, 126.9, 125.8, 125.7, 80.3, 77.3, 77.2, 77.0, 76.7, 73.0, 72.8, 56.0, 47.4, 47.3, 38.8, 38.6, 38.5, 28.2, 28.2.

#### *tert*-Butyl ((1*S*)-2-((2-Hydroxy-2-phenylethyl)­amino)-2-oxo-1-(*p*-tolyl)­ethyl)­carbamate (**24d**)

(*S*)-Boc-*p*-methylphenylglycine-OH (**6d**) (1.00 g, 3.76 mmol), **23** (516 mg, 3.76 mmol), and HATU (618 mg, 4.5 mmol) were dissolved in DCM (20 mL), followed by the addition of DIPEA (0.914 mL, 5.26 mmol). LC–MS showed complete conversion after stirring at room temperature for 40 min. The reaction mixture was diluted with DCM (100 mL), washed with 1 M HCl (1 × 100 mL), saturated aqueous sodium bicarbonate solution (2 × 100 mL), and brine (1 × 100 mL), dried over Na_2_SO_4_, filtered, and concentrated under reduced pressure. The resulting residue was purified on a silica gel column using 50% EtOAc in *n*-hexane as the mobile phase to give **24d** (838 mg, 2.18 mmol, 58% yield) in the form of a colorless viscous liquid. HRMS (ESI) *m*/*z*: calcd for C_22_H_29_N_2_O_4_ [M + H]^+^, 385.2127; found, 385.2121. ^1^H NMR (500 MHz, CDCl_3_): δ 7.33–7.19 (m, 7H), 7.16–7.12 (m, 2H), 6.64 (s, 1H), 5.89–5.85 (m, 1H), 5.18 (s, 1H), 4.79–4.74 (m, 1H), 3.72–3.50 (m, 2H), 3.34–3.29 (m, 1H), 2.35 (2s, 3H), 1.41 (2s, 9H). ^13^C NMR (126 MHz, CDCl_3_): δ 171.7, 171.3, 141.5, 141.4, 138.1, 138.1, 135.1, 135.0, 129.6, 129.6, 128.4, 127.7, 127.7, 127.1, 125.8, 77.3, 77.0, 76.8, 73.0, 47.4, 47.4, 28.3, 21.1.

#### *tert*-Butyl ((2*S*)-1-((2-Hydroxy-2-phenylethyl)­amino)-4-methyl-1-oxopentan-2-yl)­carbamate (**24e**)

(*S*)-Boc-leucine-OH (**6e**) (1.85 g, 8.01 mmol), **23** (1.10 g, 8.01 mmol), and HATU (3.65 g, 9.61 mmol) were dissolved in DCM (33 mL) and DMF (1 mL), followed by the addition of DIPEA (1.95 mL, 11.21 mmol). After stirring for 10 min at room temperature, **19** (1.00 g, 7.28 mmol) was added, followed by the addition of another portion of DIPEA (1.95 mL, 11.21 mmol). LC–MS showed complete conversion after stirring at room temperature for 40 min. The reaction mixture was diluted with DCM (100 mL), washed with 1 M HCl (1 × 100 mL), saturated aqueous sodium bicarbonate solution (2 × 100 mL), and brine (1 × 100 mL), dried over Na_2_SO_4_, filtered, and concentrated under reduced pressure. The resulting residue was purified on a silica gel column using 50% EtOAc in *n*-hexane as the mobile phase to give **6e** (1.81 g, 5.17 mmol, 71% yield) as a colorless viscous liquid. HRMS (ESI) *m*/*z*: calcd for C_19_H_31_N_2_O_4_ [M + H]^+^, 351.2284; found, 351.2283. ^1^H NMR (400 MHz, CDCl_3_): δ 7.40–7.23 (m, 6H), 6.88–6.85 (m, 1H), 5.17–5.11 (m, 1H), 4.89–4.78 (m, 1H), 4.14–4.09 (m, 1H), 3.71–3.63 (m, 1H), 3.38–3.25 (m, 1H), 1.66–1.58 (m, 2H), 1.43, 1.42 (2s, 9H), 0.98–0.85 (m, 6H). ^13^C NMR (101 MHz, CDCl_3_): δ 173.9, 155.9, 141.6, 128.4, 128.4, 127.7, 127.7, 125.8, 125.8, 80.2, 77.3, 77.2, 77.0, 76.7, 73.3, 72.8, 53.3, 47.4, 47.4, 41.3, 28.3, 24.7, 24.7, 22.9, 22.0.

#### Methyl (*S*)-2-((*S*)-2-((*tert*-Butoxycarbonyl)­amino)-3-(4-methoxyphenyl)­propanamido)-3-(pyridin-2-yl)­propanoate (**27**)

To a solution of methyl (*S*)-2-((*tert*-butoxycarbonyl)­amino)-3-(pyridin-2-yl)­propanoate **6g** (0.10 g, 0.36 mmol) in acetonitrile (1.0 mL), a 12 M hydrogen chloride solution (0.77 mL, 9.3 mmol) was added portionwise over 20 min at room temperature, and the resulting mixture was then stirred at room temperature. After 10 min, the solvent was removed in vacuo, and the resulting residue was coevaporated with toluene (1.0 mL) and further dried under high vacuum for 1 h. The residue was dissolved in dichloromethane (1.0 mL), and (*S*)-2-((*tert*-butoxycarbonyl)­amino)-3-(4-methoxyphenyl)­propanoic acid **25** (0.14 g, 0.46 mmol), *N*,*N*-di-*iso*-propylethylamine (0.25 mL, 1.4 mmol), and PyBroP (0.25 g, 0.54 mmol) were added, and the resulting mixture was stirred at room temperature. After 16 h, the reaction mixture was diluted with dichloromethane (5 mL), washed with a saturated aqueous sodium bicarbonate solution (2 5 mL), and brine (2 5 mL), the organic phase was dried over anhydrous magnesium sulfate, filtered, and the solvent was removed in vacuo. The crude product was purified using silica gel chromatography with a gradient of 50–100% ethyl acetate in heptane, followed by a purification using reversed phase HPLC with a gradient of 5–95% acetonitrile in water (modified with 0.1 M ammonium bicarbonate, pH 9), to give methyl (*S*)-2-((*S*)-2-((*tert*-butoxycarbonyl)­amino)-3-(4-methoxyphenyl)­propanamido)-3-(pyridin-2-yl)­propanoate **27** (9.3 mg, 20 μmol, 6%) as a colorless solid. HRMS (ESI) *m*/*z*: calcd for C_24_H_32_N_3_O_6_ [M + H]^+^, 458.2286; found, 458.2307. ^1^H NMR (500 MHz, CDCl_3_): δ 8.40 (d, *J* = 4.4 Hz, 1H), 7.57 (td, *J* = 7.7, 1.9 Hz, 1H), 7.38 (s, 1H), 7.14–7.11 (m, 3H), 7.08–7.06 (m, 1H), 6.81–6.78 (m, 2H), 5.03 (d, *J* = 6.7 Hz, 1H), 4.92–4.88 (m, 1H), 4.35 (s, 1H), 3.77 (s, 3H), 3.66 (s, 3H), 3.31 (dd, *J* = 14.9, 5.5 Hz, 1H), 3.20 (dd, *J* = 14.9, 4.7 Hz, 1H), 3.05–2.98 (m, 2H), 1.39 (s, 9H). ^13^C NMR (126 MHz, CDCl_3_): δ 171.4, 170.9, 158.5, 156.9, 155.2, 149.0, 136.6, 130.5, 128.6, 123.7, 121.9, 113.9, 79.8, 55.6, 55.2, 52.3, 51.7, 38.4, 37.8, 28.3.

#### *tert*-Butyl ((2*S*)-1-(((2*S*)-1-((2-Hydroxy-2-phenylethyl)­amino)-5-methyl-1-oxohexan-2-yl)­amino)-3-(4-methoxyphenyl)-1-oxopropan-2-yl)­carbamate (**26a**)

**24a** (822 mg, 2.25 mmol) was dissolved in acetonitrile (25 mL), and an aqueous 36% conc. HCl solution (5 mL) was added portionwise over 20 min at room temperature, and stirring was continued for another 10 min. The reaction mixture was then concentrated under reduced pressure, coevaporated with toluene (10 mL), and further dried under high vacuum for 1 h. Then **21** (0.729 g, 2.47 mmol), HATU (0.852 g, 2.25 mmol), and DIPEA (0.436 mL, 4.5 mmol) in DCM (10 mL) were added to it. LC–MS showed complete conversion after stirring at room temperature for 45 min. The reaction mixture was diluted with DCM (100 mL), washed with an aqueous 1 M HCl solution (2 × 50 mL), saturated aqueous sodium bicarbonate solution (2 × 50 mL), and brine (2 × 50 mL), dried over anhydrous Na_2_SO_4_, filtered, and concentrated under reduced pressure. The resulting residue was then loaded on a silica gel column and purified using 3.3% MeOH in EtOAc as the mobile phase to give **26a** (1.10 g, 2.03 mmol, 90%) as a colorless viscous liquid. HRMS (APCI) *m*/*z*: calcd for C_30_H_44_N_3_O_6_ [M + H]^+^, 542.3230; found, 542.3234. ^1^H NMR (400 MHz, DMSO-*d*_6_): δ 7.92–7.86 (m, 1H), 7.80–7.75 (m, 1H), 7.35–7.27 (m, 3H), 7.25–7.21 (m, 1H), 7.17–7.15 (m, 2H), 6.96–6.91 (m, 1H), 6.83–6.81 (m, 2H), 5.45–5.44 (m, 1H), 4.62–4.56 (m, 1H), 4.26–4.17 (m, 1H), 4.13–4.01 (m, 1H), 3.70 (s, 3H), 3.24–3.21 (m, 1H), 2.90–2.85 (m, 1H), 2.70–2.60 (m, 1H), 1.63–1.51 (m, 1H), 1.49–1.37 (m, 2H), 1.30, 1.31 (2s, 6H), 1.14–1.08 (m, 2H), 0.83, 0.81 (2s, 6H). ^13^C NMR (126 MHz, DMSO-*d*_6_): δ 171.5, 171.5, 157.7, 143.6, 143.5, 130.1, 130.0, 127.9, 127.0, 126.1, 126.0, 113.4, 78.0, 71.1, 54.9, 46.6, 33.9, 28.1, 27.2, 22.5, 22.4, 22.3, 22.3.

#### *tert*-Butyl ((2*S*)-1-(((2*S*)-1-((2-Hydroxy-2-phenylethyl)­amino)-1-oxo-4-phenylbutan-2-yl)­amino)-3-(4-methoxyphenyl)-1-oxopropan-2-yl)­carbamate (**26b**)

**24b** (1.19 mg, 2.98 mmol) was dissolved in acetonitrile (33 mL), and an aqueous 36% conc. HCl solution (6.6 mL) was added portionwise over 20 min at room temperature, and stirring was continued for another 10 min. The reaction mixture was then concentrated under reduced pressure, coevaporated with toluene (10 mL), and further dried under high vacuum for 1 h. Then **21** (0.962 g, 3.26 mmol), HATU (1.12 g, 2.98 mmol), and DIPEA (0.575 mL, 5.94 mmol) in DCM (13 mL) were added to it. LC–MS showed complete conversion after stirring at room temperature for 45 min. The reaction mixture was diluted with DCM (100 mL), washed with an aqueous 1 M HCl solution (2 × 50 mL), saturated aqueous sodium bicarbonate solution (2 × 50 mL), and brine (2 × 50 mL), dried over anhydrous Na_2_SO_4_, filtered, and concentrated under reduced pressure. The resulting residue was then loaded on a silica gel column and purified using 3.3% MeOH in EtOAc as the mobile phase to give **26b** (1.28 g, 2.22 mmol, 88%) as a colorless viscous liquid. HRMS (ESI) *m*/*z*: calcd for C_33_H_42_N_3_O_6_ [M + H]^+^, 576.3074; found, 576.3070. ^1^H NMR (400 MHz, DMSO-*d*_6_): δ 8.08–7.91 (m, 1H), 7.90–7.79 (m, 1H), 7.40–7.10 (m, 10H), 7.09–6.94 (m, 1H), 6.89–6.77 (m, 2H), 5.51–5.39 (m, 1H), 4.69–4.52 (m, 1H), 4.36–4.05 (m, 2H), 3.70 (s, 3H), 3.32 (s, 2H), 3.23 (q, *J* = 5.6 Hz, 1H), 3.11 (m, 1H), 2.98–2.85 (m, 1H), 2.79–2.62 (m, 1H), 1.99–1.64 (m, 2H), 1.31 (s, 9H). ^13^C NMR (101 MHz, DMSO-*d*_6_): δ 171.6, 171.3, 157.7, 155.3, 143.4, 141.4, 130.1, 128.2, 128.2, 127.9, 127.0, 126.0, 126.0, 125.7, 113.4, 78.1, 71.1, 56.1, 54.9, 52.0, 46.6, 36.2, 34.3, 31.0, 28.1.

#### *tert*-Butyl ((2*S*)-1-(((2*S*)-1-((2-Hydroxy-2-phenylethyl)­amino)-1-oxo-3-phenylpropan-2-yl)­amino)-3-(4-methoxyphenyl)-1-oxopropan-2-yl)­carbamate (**26c**)

**24c** (769 mg, 2.00 mmol) was dissolved in acetonitrile (22 mL), and an aqueous 36% conc. HCl solution (4.4 mL) was added portionwise over 20 min at room temperature, and stirring was continued for another 10 min. The reaction mixture was then concentrated under reduced pressure, coevaporated with toluene (10 mL), and further dried under high vacuum for 1 h. Then **21** (0.641 g, 2.17 mmol), HATU (0.746 g, 2.00 mmol), and DIPEA (0.383 mL, 3.96 mmol) in DCM (9 mL) were added to it. LC–MS showed complete conversion after stirring at room temperature for 45 min. The reaction mixture was diluted with DCM (100 mL), washed with an aqueous 1 M HCl solution (2 × 50 mL), saturated aqueous sodium bicarbonate solution (2 × 50 mL), and brine (2 × 50 mL), dried over anhydrous Na_2_SO_4_, filtered, and concentrated under reduced pressure. The resulting residue was then loaded on a silica gel column using 50% EtOAc in *n*-hexane to 10% MeOH in EtOAc as the mobile phase to give **26c** (763 mg, 1.36 mmol, 68%) as a colorless viscous liquid. HRMS (ESI) *m*/*z*: calcd for C_32_H_39_N_3_O_6_ [M – H]^−^, 560.2761; found, 560.2741. ^1^H NMR (400 MHz, DMSO-*d*_6_): δ 8.08 (m, 2H), 7.90 (t, *J* = 8.4 Hz, 1H), 7.36–7.01 (m, 15H), 6.88–6.68 (m, 4H), 5.47 (s, 1H), 4.64–4.41 (m, 3H), 4.10–3.92 (m, 2H), 3.68 (s, 4H), 3.30–3.06 (m, 3H), 2.97–2.84 (m, 2H), 2.80–2.66 (m, 3H), 2.55 (m, 1H), 1.28 (2s, 9H). ^13^C NMR (101 MHz, DMSO-*d*_6_): δ 171.7, 171.6, 171.4, 171.3, 158.1, 155.4, 144.0, 143.9, 138.0, 130.5, 130.3, 129.7, 128.4, 127.5, 127.4, 126.6, 126.5, 126.4, 113.8, 78.5, 78.5, 71.7, 71.6, 56.6, 56.5, 55.3, 54.0, 47.2, 38.3, 37.1, 28.5, 28.2.

#### *tert*-Butyl ((2*S*)-1-(((1*S*)-2-((2-Hydroxy-2-phenylethyl)­amino)-2-oxo-1-(*p*-tolyl)­ethyl)­amino)-3-(4-methoxyphenyl)-1-oxopropan-2-yl)­carbamate (**26d**)

**6d** (1.35 g, 3.52 mmol) was dissolved in acetonitrile (35 mL), and an aqueous 36% conc. HCl solution (6.8 mL) was added portionwise over 20 min at room temperature, and stirring was continued for another 10 min. The reaction mixture was then concentrated under reduced pressure, coevaporated with toluene (10 mL), and further dried under high vacuum for 1 h. Then **21** (1.15 g, 3.90 mmol), HATU (1.34 g, 1.46 mmol), and DIPEA (1.23 mL, 7.05 mmol) in DCM (14 mL) were added to it. LC–MS showed complete conversion after stirring at room temperature for 45 min. The reaction mixture was diluted with DCM (100 mL), washed with an aqueous 1 M HCl solution (2 × 50 mL), saturated aqueous sodium bicarbonate solution (2 × 50 mL), and brine (2 × 50 mL), dried over anhydrous Na_2_SO_4_, filtered, and concentrated under reduced pressure. The resulting residue was then loaded on a silica gel column and purified using 50% EtOAc in *n*-hexane to 10% MeOH in EtOAc as the mobile phase to give **26d** (1.68 g, 2.68 mmol, 52%) as a colorless viscous liquid. HRMS (APCI) *m*/*z*: calcd for C_32_H_40_N_3_O_6_ [M + H]^+^, 562.2912; found, 562.2912. ^1^H NMR (500 MHz, DMSO-*d*_6_): δ 8.42–8.21 (m, 2H), 7.34–7.04 (m, 12H), 6.83–6.82 (m, 2H), 5.53–5.41 (m, 2H), 4.58–4.55 (m, 1H), 4.22–4.16 (m, 1H), 3.71 (s, 3H), 3.26–3.17 (m, 2H), 3.18–2.91 (m, 1H), 2.70–2.65 (m, 1H), 2.30, 2.29 (2s, 3H), 1.32, 1.33 (2s, 9H). ^13^C NMR (126 MHz, DMSO-*d*_6_): δ 171.5, 171.3, 170.3, 158.2, 155.8, 155.7, 144.0, 143.9, 136.9, 136.9, 136.5, 136.3, 130.6, 130.4, 129.1, 128.4, 128.4, 127.2, 127.2, 126.5, 126.4, 113.9, 79.6, 78.6, 78.6, 71.5, 71.5, 56.5, 56.4, 56.0, 56.0, 55.3, 47.3, 47.2, 40.3, 40.1, 39.9, 39.8, 39.6, 28.5, 21.1, 21.1.

#### *tert*-Butyl ((2*S*)-1-(((2*S*)-1-((2-Hydroxy-2-phenylethyl)­amino)-4-methyl-1-oxopentan-2-yl)­amino)-3-(4-methoxyphenyl)-1-oxopropan-2-yl)­carbamate (**26e**)

**6e** (1.81 g, 5.17 mmol) was dissolved in acetonitrile (50 mL), and an aqueous 36% conc. HCl solution (10.0 mL) was added portionwise over 20 min at room temperature, and stirring was continued for another 10 min. The reaction mixture was then concentrated under reduced pressure, coevaporated with toluene (10 mL), and further dried under high vacuum for 1 h. Then **21** (1.68 g, 5.69 mmol), HATU (1.96 g, 5.17 mmol), and DIPEA (1.80 mL, 10.3 mmol) in DCM (20 mL) were added to it. LC–MS showed complete conversion after stirring at room temperature for 45 min. The reaction mixture was diluted with DCM (100 mL), washed with an aqueous 1 M HCl solution (2 × 50 mL), saturated aqueous sodium bicarbonate solution (2 × 50 mL), and brine (2 × 50 mL), dried over anhydrous Na_2_SO_4_, filtered, and concentrated under reduced pressure. The resulting residue was then loaded on a silica gel column and purified using 50% EtOAc in *n*-hexane to 10% MeOH in EtOAc as the mobile phase to give **26e** (1.68 g, 2.68 mmol, 52%) as a colorless viscous liquid. HRMS (ESI) *m*/*z*: calcd for C_29_H_41_N_3_O_6_Na [M + Na]^+^, 550.2888; found, 550.2906. ^1^H NMR (500 MHz, DMSO-*d*_6_): δ 7.97–7.76 (m, 3H), 7.35–7.28 (m, 4H), 7.25–7.20 (m, 2H), 7.19–7.12 (m, 2H), 6.92–6.88 (m, 1H), 6.85–6.78 (m, 2H), 5.42 (s, 1H), 4.61–4.57 (m, 1H), 4.36–4.23 (m, 1H), 4.11–4.06 (m, 1H), 3.31–3.21 (m, 2H), 3.21–3.08 (m, 1H), 2.89–2.85 (m, 1H), 2.70–2.62 (m, 1H), 1.60–1.46 (m, 2H), 1.45–1.33 (m, 2H), 1.31, 1.30 (2s, 9H), 0.88–0.76 (m, 6H). ^13^C NMR (126 MHz, DMSO-*d*_6_): δ 172.0, 171.9, 171.3, 171.3, 157.7, 155.2, 155.2, 143.6, 143.5, 130.1, 129.9, 127.9, 127.9, 127.0, 126.9, 126.1, 126.0, 113.4, 79.1, 78.0, 78.0, 71.2, 71.1, 56.0, 55.9, 54.9, 50.8, 50.8, 46.6, 46.6, 41.4, 41.3, 40.1, 40.0, 39.9, 39.8, 39.7, 39.6, 39.6, 39.5, 39.4, 39.3, 39.1, 39.0, 36.3, 36.2, 28.1, 27.8, 23.9, 23.9, 23.1, 23.0, 21.6, 21.6.

#### *tert*-Butyl ((2*S*)-1-(((2*S*)-1-((2-Hydroxy-2-phenylethyl)­amino)-1-oxo-3-(pyridin-2-yl)­propan-2-yl)­amino)-3-(4-methoxyphenyl)-1-oxopropan-2-yl)­carbamate (**26g**)

To a solution of methyl (*S*)-2-((*S*)-2-((*tert*-butoxycarbonyl)­amino)-3-(4-methoxyphenyl)­propanamido)-3-(pyridin-2-yl)­propanoate (**27,** 222 mg, 0.49 mmol) in dimethylformamide (23 mL), *rac*-2-amino-1-phenylethan-1-ol (**23,** 2.7 g, 20 mmol) was added, and the resulting reaction mixture was stirred at 70 °C. After 4 d, the solvent was removed in vacuo, and the crude product was purified using silica gel chromatography with a gradient of 40–100% ethyl acetate in heptane, followed by a purification using reversed phase HPLC with a gradient of 5–95% acetonitrile in water (modified with 0.1 M ammonium bicarbonate, pH 9) to give crude *tert*-butyl ((*S*)-1-(((*S*)-1-(((*RS*)-2-hydroxy-2-phenylethyl)­amino)-1-oxo-3-(pyridin-2-yl)­propan-2-yl)­amino)-3-(4-methoxyphenyl)-1-oxopropan-2-yl)­carbamate, which was used in the next step without further purification. To a solution of crude *tert*-butyl ((*S*)-1-(((*S*)-1-(((*RS*)-2-hydroxy-2-phenylethyl)­amino)-1-oxo-3-(pyridin-2-yl)­propan-2-yl)­amino)-3-(4-methoxyphenyl)-1-oxopropan-2-yl)­carbamate in ethyl acetate (2.1 mL) and dimethyl sulfoxide (0.4 mL), 2-iodoxybenzoic acid (0.32 g, 0.51 mmol) was added portionwise over 30 min, and the resulting reaction mixture was stirred at room temperature. After 24 h, the reaction mixture was diluted with ethyl acetate (5 mL) and the solid was removed by filtration through Celite. The filtrate was washed with aqueous sodium bicarbonate (2 × 2 mL) and brine (2 × 2 mL), dried over anhydrous magnesium sulfate, and filtered. The solvent was removed in vacuo, and the crude product was purified using silica gel chromatography with a gradient of 40–100% ethyl acetate in heptane, followed by a purification using reversed phase HPLC with a gradient of 5–95% acetonitrile in water (modified with 0.1 M ammonium bicarbonate, pH 9) to give *tert*-butyl ((*S*)-3-(4-methoxyphenyl)-1-oxo-1-(((*S*)-1-oxo-1-((2-oxo-2-phenylethyl)­amino)-3-(pyridin-2-yl)­propan-2-yl)­amino)­propan-2-yl)­carbamate (**26g,** 2.7 mg, 5 μmol, 1%) as a colorless solid. HRMS (ESI) *m*/*z*: calcd for C_31_H_37_N_4_O_6_ [M + H]^+^, 561.2708; found, 561.2698. ^1^H NMR (500 MHz, DMSO-*d*_6_): δ 8.49–8.48 (m, 1H), 8.24–8.21 (m, 2H), 8.00–7.97 (m, 2H), 7.69–7.65 (m, 2H), 7.56–7.52 (m, 2H), 7.30 (d, *J* = 7.7 Hz, 1H), 7.22 (ddd, *J* = 7.5, 4.9, 0.9 Hz, 1H), 7.10 (d, *J* = 8.4 Hz, 2H), 6.84 (d, *J* = 8.9 Hz, 1H), 6.79 (d, *J* = 8.5 Hz, 2H), 4.84–4.80 (m, 1H), 4.66–4.59 (m, 2H), 4.08–4.03 (m, 1H), 3.68 (s, 3H), 3.23 (dd, *J* = 14.2, 4.8 Hz, 1H), 3.04 (dd, *J* = 14.3, 9.0 Hz, 1H), 2.82 (dd, *J* = 14.0, 4.2 Hz, 1H), 2.59 (dd, *J* = 13.8, 10.2 Hz, 1H), 1.28 (s, 9H). ^13^C NMR (126 MHz, DMSO-*d*_6_): δ 194.9, 171.5, 171.3, 157.7, 157.6, 155.1, 148.8, 136.3, 134.9, 133.6, 130.2, 129.9, 128.8, 127.8, 123.7, 121.7, 113.4, 78.1, 56.1, 54.9, 52.3, 46.0, 40.1, 36.6, 28.1.

#### (*S*)-2-((*S*)-2-Acetamido-3-(4-Methoxyphenyl)­propanamido)-5-methyl-*N*-(2-oxo-2-phenylethyl)­hexanamide (**5a**)

**26a** (0.417 g, 0.769 mmol) was dissolved in acetonitrile (20 mL), and an aqueous 36% conc. HCl solution (4.0 mL) was added to it. The reaction was monitored on LC–MS and completed in 20 min. The reaction mixture was then concentrated under reduced pressure, coevaporated with toluene (20 mL), and further dried under high vacuum for 1 h. THF (5 mL) was then added to it, followed by the addition of triethylamine (535 μL, 3.83 mmol) and acetic anhydride (97 μL, 1.00 mmol) simultaneously. The reaction was completed after stirring at room temperature for 10 min, as shown by LC–MS. Ethyl acetate (20 mL) was added, washed with an aqueous 1 M HCl (1 × 10 mL), saturated aqueous sodium bicarbonate solution (2 × 10 mL), and brine (1 × 10 mL), dried, filtered, concentrated under reduced pressure, and purified on a silica gel column by 100% EtOAc to 10% MeOH/EtOAc as the mobile phase to give the acetylated analogue (338 mg, 0.700 mmol, 91%). This compound was oxidized without any characterization. This compound (338 mg, 0.700 mmol) was dissolved in ethyl acetate (8 mL) and DMSO (1 mL), and IBX (45 wt %, 3.57 mmol, 1.00 g) was added portionwise over 20 min with stirring at 85 °C and monitoring by LC–MS. After 20 min, the reaction mixture was allowed to cool to room temperature and centrifuged (to separate undissolved IBX), and ethyl acetate was added (2 × 50 mL), washed with saturated aqueous sodium bicarbonate solution (2 × 50 mL), dried over anhydrous Na_2_SO_4_, concentrated under reduced pressure, and purified on reverse phase HPLC using a gradient from 30% to 90% acetonitrile in water to give **5a** (37 mg, 10% yield) as a colorless viscous liquid. HRMS (ESI) *m*/*z*: calcd for C_27_H_36_N_3_O_5_ [M + H]^+^, 482.2655; found, 482.2651. ^1^H NMR (400 MHz, DMSO-*d*_6_): δ 8.20–8.12 (m, 1H), 8.09–7.95 (m, 4H), 7.71–7.62 (m, 1H), 7.59–7.49 (m, 2H), 7.21–7.10 (m, 2H), 6.85–6.76 (m, 2H), 4.65–4.56 (m, 2H), 4.58–4.43 (m, 1H), 4.37–4.26 (m, 1H), 3.69 (s, 3H), 2.99–2.89 (m, 1H), 2.72–2.61 (m, 1H), 1.78–1.64 (m, 4H), 1.61–1.43 (m, 2H), 1.26–1.11 (m, 2H), 0.85 (2d 2.1 Hz, 6H). ^13^C NMR (101 MHz, DMSO-*d*_6_): δ 195.0, 195.0, 171.8, 171.2, 169.0, 157.6, 134.9, 133.5, 130.1, 129.8, 128.7, 127.8, 113.3, 54.8, 54.0, 52.5, 45.8, 40.2, 40.1, 39.9, 39.9, 39.7, 39.7, 39.5, 39.5, 39.3, 39.3, 39.1, 38.8, 36.5, 34.1, 30.1, 27.3, 22.5, 22.4, 22.3.

#### (*S*)-2-((*S*)-2-Acetamido-3-(4-methoxyphenyl)­propanamido)-*N*-(2-oxo-2-phenylethyl)-4-phenylbutanamide (**5b**)

**26b** (1.28 g, 2.22 mmol) was dissolved in acetonitrile (60 mL), and an aqueous 36% conc. HCl solution (12.0 mL) was added to it. The reaction was monitored on LC–MS and completed in 20 min. The reaction mixture was then concentrated under reduced pressure, coevaporated with toluene (20 mL), and further dried under high vacuum for 1 h. THF (15 mL) was then added to it, followed by the addition of triethylamine (1.55 mL, 11.1 mmol) and acetic anhydride (280 μL, 2.88 mmol) simultaneously. The reaction was completed after stirring at room temperature for 10 min, as shown by LC–MS. Ethyl acetate (60 mL) was added, washed with an aqueous 1 M HCl (1 × 20 mL), saturated aqueous sodium bicarbonate solution (2 × 20 mL), and brine (1 × 20 mL), dried, filtered, concentrated under reduced pressure, and purified on a silica gel column by 100% EtOAc to 10% MeOH/EtOAc as the mobile phase to give the acetylated analogue. This compound was oxidized without any characterization and dissolved in ethyl acetate (24 mL) and DMSO (3 mL), and IBX (45 wt %, 10.71 mmol, 3.00 g) was added portionwise over 20 min with stirring at 85 °C and monitoring by LC–MS. After 20 min, the reaction mixture was allowed to cool to room temperature and centrifuged (to separate undissolved IBX), and ethyl acetate was added (2 × 50 mL), washed with saturated aqueous sodium bicarbonate solution (2 × 50 mL), dried over anhydrous Na_2_SO_4_, concentrated under reduced pressure, and purified on reverse phase HPLC using a gradient from 30% to 90% acetonitrile in water to give **5b** (80 mg, 0.15 mmol, 7% yield) as a colorless powder. HRMS (ESI) *m*/*z*: calcd for C_30_H_34_N_3_O_5_ [M + H]^+^, 516.2498; found, 516.2483. ^1^H NMR (400 MHz, DMSO-*d*_6_): δ 8.25–8.17 (m, 1H), 8.15–8.07 (m, 2H), 8.04–7.95 (m, 2H), 7.72–7.62 (m, 1H), 7.59–7.50 (m, 2H), 7.36–7.14 (m, 7H), 6.86–6.77 (m, 2H), 4.72–4.48 (m, 3H), 4.40–4.32 (m, 1H), 3.68 (s, 3H), 3.02–2.94 (m, 1H), 2.76–2.55 (m, 3H), 2.09–1.81 (m, 2H), 1.78 (s, 3H). ^13^C NMR (101 MHz, DMSO-*d*_6_): δ 195.0, 171.6, 169.2, 157.7, 141.5, 134.9, 133.5, 130.1, 129.8, 128.7, 128.3, 127.8, 125.7, 113.4, 54.8, 54.2, 52.1, 45.9, 36.5, 34.1, 31.2, 22.4.

#### (*S*)-2-Acetamido-3-(4-methoxyphenyl)-*N*-((*S*)-1-oxo-1-((2-oxo-2-phenylethyl)­amino)-3-phenylpropan-2-yl)­propanamide (**5c**)

**26c** (700 mg, 1.24 mmol) was dissolved in acetonitrile (30 mL), and an aqueous 36% conc. HCl solution (6.0 mL) was added to it. The reaction was monitored on LC–MS and completed in 20 min. The reaction mixture was then concentrated under reduced pressure, coevaporated with toluene (20 mL), and further dried under high vacuum for 1 h. THF (7 mL) was then added to it, followed by the addition of triethylamine (0.865 mL, 6.2 mmol) and acetic anhydride (155 μL, 1.60 mmol) simultaneously. The reaction was completed after stirring at room temperature for 10 min, as shown by LC–MS. Ethyl acetate (30 mL) was added, washed with an aqueous 1 M HCl (1 × 10 mL), saturated aqueous sodium bicarbonate solution (2 × 10 mL), and brine (1 × 10 mL), dried, filtered, concentrated under reduced pressure, and purified on a silica gel column by 100% EtOAc to 10% MeOH/EtOAc as the mobile phase to give the acetylated analogue. This compound was oxidized without any characterization and dissolved in ethyl acetate (12 mL) and DMSO (1.5 mL), and IBX (45 wt %, 5.98 mmol, 1.67 g) was added portionwise over 20 min with stirring at 85 °C and monitoring by LC–MS. After 20 min, the reaction mixture was allowed to cool to room temperature and centrifuged (to separate undissolved IBX), and ethyl acetate was added (2 × 50 mL), washed with saturated aqueous sodium bicarbonate solution (2 × 50 mL), dried over anhydrous Na_2_SO_4_, concentrated under reduced pressure, and purified on reverse phase HPLC using a gradient from 30% to 90% acetonitrile in water to give **5c** (96 mg, 0.19 mmol, 15% yield) as a colorless powder. HRMS (ESI) *m*/*z*: calcd for C_29_H_31_N_3_O_5_ [M – H]^−^, 500.2185; found, 500.2184. ^1^H NMR (400 MHz, DMSO-*d*_6_): δ 8.28 (t, *J* = 5.5 Hz, 1H), 8.10 (d, *J* = 8.4 Hz, 1H), 8.03–7.90 (m, 3H), 7.65 (d, *J* = 7.4 Hz, 1H), 7.53 (dd, *J* = 8.2, 7.0 Hz, 2H), 7.31–7.14 (m, 6H), 7.11–7.04 (m, 2H), 6.80–6.70 (m, 2H), 4.69–4.55 (m, 3H), 4.41 (dt, *J* = 5.3, 4.0 Hz, 1H), 3.66 (s, 3H), 3.08 (dd, *J* = 13.9, 4.4 Hz, 1H), 2.90–2.78 (m, 2H), 2.58 (dd, *J* = 13.9, 9.8 Hz, 1H), 1.70 (s, 3H). ^13^C NMR (101 MHz, DMSO-*d*_6_): δ 195.4, 171.7, 171.7, 169.4, 158.1, 138.1, 135.3, 134.0, 130.5, 130.5, 130.2, 129.7, 129.6, 129.2, 128.4, 128.2, 126.6, 113.8, 55.3, 54.4, 54.1, 46.3, 38.1, 37.0, 22.8.

#### (*S*)-2-Acetamido-3-(4-methoxyphenyl)-*N*-((*S*)-2-oxo-2-((2-oxo-2-phenylethyl)­amino)-1-(*p*-tolyl)­ethyl)­propanamide (**5d**)

**26d** (1.21 g, 2.16 mmol) was dissolved in acetonitrile (50 mL), and an aqueous 36% conc. HCl solution (10 mL) was added to it. The reaction was monitored on LC–MS and completed in 20 min. The reaction mixture was then concentrated under reduced pressure, coevaporated with toluene (20 mL), and further dried under high vacuum for 1 h. THF (12 mL) was then added to it, followed by the addition of triethylamine (1.50 mL, 10.8 mmol) and acetic anhydride (270 μL, 2.78 mmol) simultaneously. The reaction was completed after stirring at room temperature for 10 min, as shown by LC–MS. Ethyl acetate (52 mL) was added, washed with an aqueous 1 M HCl (1 × 20 mL), saturated aqueous sodium bicarbonate solution (2 × 20 mL), and brine (1 × 20 mL), dried, filtered, concentrated under reduced pressure, and purified on a silica gel column by 100% EtOAc to 10% MeOH/EtOAc as the mobile phase to give the acetylated analogue. This compound was oxidized without any characterization and dissolved in ethyl acetate (20 mL) and DMSO (2.6 mL), and IBX (45 wt %, 10.40 mmol, 2.90 g) was added portionwise over 20 min with stirring at 85 °C and monitoring by LC–MS. After 20 min, the reaction mixture was allowed to cool to room temperature and centrifuged (to separate undissolved IBX), and ethyl acetate was added (2 × 50 mL), washed with saturated aqueous sodium bicarbonate solution (2 × 50 mL), dried over anhydrous Na_2_SO_4_, concentrated under reduced pressure, and purified on reverse phase HPLC using a gradient from 30% to 90% acetonitrile in water to give **5d** (43 mg, 86 μmol, 10% yield) as a colorless powder. HRMS (ESI) *m*/*z*: calcd for C_29_H_31_N_3_O_5_ [M – H]^−^, 500.2185; found, 500.2184. HRMS (APCI) *m*/*z*: calcd for C_29_H_32_N_3_O_5_ [M + H]^+^, 502.2336; found, 502.2335. ^1^H NMR (500 MHz, DMSO-*d*_6_): δ 8.57 (t, *J* = 5.6 Hz, 1H), 8.50 (d, *J* = 8.2 Hz, 1H), 8.08 (d, *J* = 8.4 Hz, 1H), 7.98 (dd, *J* = 8.1, 1.4 Hz, 2H), 7.71–7.62 (m, 1H), 7.53 (t, *J* = 7.7 Hz, 2H), 7.35 (d, *J* = 7.9 Hz, 2H), 7.17 (dd, *J* = 10.6, 8.2 Hz, 4H), 6.82 (d, *J* = 8.6 Hz, 2H), 5.58 (d, *J* = 8.1 Hz, 1H), 4.73–4.55 (m, 3H), 3.70 (s, 3H), 2.96 (dd, *J* = 13.9, 4.4 Hz, 1H), 2.69 (dd, *J* = 13.8, 10.0 Hz, 1H), 2.30 (s, 3H), 1.75 (s, 3H). ^13^C NMR (126 MHz, DMSO-*d*_6_): δ 195.3, 171.3, 170.5, 169.6, 158.1, 137.1, 136.1, 135.3, 134.0, 130.6, 130.2, 129.2, 129.2, 128.2, 127.6, 113.8, 56.1, 55.3, 54.4, 46.4, 40.4, 40.4, 40.3, 40.2, 40.1, 40.0, 39.9, 39.9, 39.8, 39.6, 39.4, 37.0, 22.9, 21.1.

#### (*S*)-2-((*S*)-2-Acetamido-3-(4-methoxyphenyl)­propanamido)-4-methyl-*N*-(2-oxo-2-phenylethyl)­pentanamide (**5e**)

**26e** (1.09 g, 2.07 mmol) was dissolved in acetonitrile (50 mL), and an aqueous 36% conc. HCl solution (10 mL) was added to it. The reaction was monitored on LC–MS and completed in 20 min. The reaction mixture was then concentrated under reduced pressure, coevaporated with toluene (20 mL), and further dried under high vacuum for 1 h. THF (12 mL) was then added to it, followed by the addition of triethylamine (1.56 mL, 11.26 mmol) and acetic anhydride (281 μL, 2.89 mmol) simultaneously. The reaction was completed after stirring at room temperature for 10 min, as shown by LC–MS. Ethyl acetate (52 mL) was added, washed with an aqueous 1 M HCl (1 × 20 mL), saturated aqueous sodium bicarbonate solution (2 × 20 mL), and brine (1 × 20 mL), dried, filtered, concentrated under reduced pressure, and purified on a silica gel column by 100% EtOAc to 10% MeOH/EtOAc as the mobile phase to give the acetylated analogue. This compound was oxidized without any characterization and dissolved in ethyl acetate (20 mL) and DMSO (2.6 mL), and IBX (45 wt %, 10.84 mmol, 3.02 g) was added portionwise over 20 min with stirring at 85 °C and monitoring by LC–MS. After 20 min, the reaction mixture was allowed to cool to room temperature and centrifuged (to separate undissolved IBX), and ethyl acetate was added (2 × 50 mL), washed with saturated aqueous sodium bicarbonate solution (2 × 50 mL), dried over anhydrous Na_2_SO_4_, concentrated under reduced pressure, and purified on reverse phase HPLC using a gradient from 20% to 70% acetonitrile in water to give **5e** (310 mg, 664 μmol, 32% yield) as a colorless powder. HRMS (ESI) *m*/*z*: calcd for C_26_H_33_N_3_O_5_Na [M + Na]^+^, 490.2312; found, 490.2307. ^1^H NMR (400 MHz, CDCl_3_): δ 8.02–7.93 (m, 2H), 7.68–7.59 (m, 1H), 7.51 (t, *J* = 7.6 Hz, 2H), 7.12 (d, *J* = 8.2 Hz, 2H), 6.93 (t, *J* = 4.4 Hz, 1H), 6.77 (d, *J* = 8.3 Hz, 2H), 6.53 (d, *J* = 8.0 Hz, 1H), 6.30 (d, *J* = 7.5 Hz, 1H), 4.78–4.61 (m, 3H), 4.54 (td, *J* = 8.4, 5.6 Hz, 1H), 3.66 (s, 3H), 3.03 (d, *J* = 6.9 Hz, 2H), 2.01 (s, 3H), 1.79–1.41 (m, 4H), 0.93 (d, *J* = 6.3, 3H), 0.91 (d, *J* = 6.3, 3H). ^13^C NMR (101 MHz, CDCl_3_): δ 193.7, 171.6, 171.0, 170.1, 158.5, 134.3, 134.1, 130.3, 128.9, 128.7, 128.2, 127.9, 114.0, 77.3, 77.0, 76.6, 55.0, 54.7, 51.8, 46.2, 41.2, 37.4, 24.7, 23.1, 22.8, 21.9.

#### (*S*)-3-(4-Methoxyphenyl)-*N*-((*S*)-1-oxo-1-((2-oxo-2-phenylethyl)­amino)-3-phenylpropan-2-yl)-2-propionamidopropanamide (**5f**)

**26c** (1.3 g, 2.31 mmol) was dissolved in acetonitrile (50 mL), and an aqueous 36% conc. HCl solution (10 mL) was added. LC–MS showed complete conversion after stirring at room temperature for 30 min. The reaction mixture was concentrated under reduced pressure and dried by coevaporation with toluene (2 × 10 mL). This material was dissolved in THF (20 mL), and TEA (1.68 mL, 12.0 mmol) and propionyl chloride (1.58 mL, 18.1 mmol) were added simultaneously, followed by the addition of DMF (2 mL). The reaction was complete after stirring at room temperature for 1 h, as shown by LC–MS. Ethyl acetate (50 mL) was added, washed with an aqueous 1 M HCl solution (30 mL), saturated aqueous sodium bicarbonate solution (2 × 50 mL), and brine (2 × 50 mL), concentrated under reduced pressure, and purified on reverse phase HPLC using a gradient from 20% to 78% acetonitrile in water to give tripeptide (478 mg, 923 μmol, 40%) as a colorless powder. This compound was then subjected to oxidation without any characterization and dissolved in ethyl acetate (22 mL) and DMSO (2.8 mL), and IBX (45 wt %, 11.12 mmol, 3.10 g) was added portionwise over 20 min with stirring at 85 °C and monitoring by LC–MS. After 20 min, the reaction mixture was allowed to cool to room temperature and centrifuged (to separate undissolved IBX), and ethyl acetate was added (2 × 50 mL), washed with saturated aqueous sodium bicarbonate solution (2 × 50 mL), dried over anhydrous Na_2_SO_4_, concentrated under reduced pressure, and purified on reverse phase HPLC using a gradient from 30% to 90% acetonitrile in water to give **5f** (252 mg, 489 μmol, 53%) as a colorless powder. HRMS (ESI) *m*/*z*: calcd for C_30_H_32_N_3_O_5_ [M – H]^−^, 514.2342; found, 514.2334. ^1^H NMR (400 MHz, DMSO-*d*_6_): δ 8.05–7.96 (m, 2H), 7.88 (dd, *J* = 8.4, 2.7 Hz, 1H), 7.38–7.29 (m, 4H), 7.29–7.15 (m, 6H), 7.10 (dd, *J* = 8.6, 3.4 Hz, 2H), 6.79 (d, *J* = 8.3 Hz, 2H), 5.47 (s, 1H), 4.63–4.56 (m, 1H), 4.54–4.47 (m, 1H), 4.44–4.37 (m, 1H), 3.70 (s, 3H), 3.35–3.10 (m, 3H), 2.95 (ddd, *J* = 13.8, 4.8, 2.8 Hz, 1H), 2.86 (ddd, *J* = 13.1, 8.0, 4.4 Hz, 1H), 2.75 (ddd, *J* = 13.8, 9.1, 1.8 Hz, 1H), 2.61 (ddd, *J* = 13.4, 9.9, 2.7 Hz, 1H), 2.01 (q, *J* = 7.6 Hz, 2H), 0.87 (td, *J* = 7.6, 1.2 Hz, 3H). ^13^C NMR (101 MHz, DMSO-*d*_6_): δ 173.2, 173.2, 171.6, 171.5, 171.3, 171.3, 158.1, 144.0, 143.9, 138.1, 130.6, 130.2, 129.6, 128.4, 127.5, 127.5, 126.6, 126.5, 126.4, 113.8, 71.7, 71.6, 55.3, 54.4, 54.2, 47.2, 38.2, 38.1, 37.0, 36.9, 28.7, 10.2.

#### (*S*)-2-Acetamido-3-(4-methoxyphenyl)-*N*-((*S*)-1-oxo-1-((2-oxo-2-phenylethyl)­amino)-3-(pyridin-2-yl)­propan-2-yl)­propanamide (**5g**)

To a solution of *tert*-butyl ((*S*)-3-(4-methoxyphenyl)-1-oxo-1-(((*S*)-1-oxo-1-((2-oxo-2-phenylethyl)­amino)-3-(pyridin-2-yl)­propan-2-yl)­amino)­propan-2-yl)­carbamate **26g** (30 mg, 54 μmol) in acetonitrile (1.0 mL), a 12 M aqueous hydrogen chloride solution (0.12 mL, 1.4 mmol) was added, and the resulting solution was stirred at room temperature. After 20 min, the solvent was removed in vacuo, and the resulting residue was coevaporated with toluene (1.0 mL) and further dried under high vacuum for 1 h. The residue was dissolved in tetrahydrofuran (0.7 mL), DIPEA (0.36 mL, 0.27 mmol) and acetic anhydride (6.4 μL, 0.070 mmol) were added simultaneously, and the reaction mixture was stirred at room temperature. After 10 min, ethyl acetate (5 mL) was added; the organic phase was washed with an aqueous 1 M hydrogen chloride solution (1 5 mL), a saturated aqueous sodium bicarbonate solution (2 5 mL), and brine (1 5 mL), dried over anhydrous magnesium sulfate, and filtered, and the solvent was removed in vacuo. The crude product was purified using silica gel chromatography with a gradient of 40–100% ethyl acetate in heptane, followed by a purification using reversed phase HPLC with a gradient of 5–95% acetonitrile in water (modified with 0.1 M ammonium bicarbonate, pH 9) to give (*S*)-2-acetamido-3-(4-methoxyphenyl)-*N*-((*S*)-1-oxo-1-((2-oxo-2-phenylethyl)­amino)-3-(pyridin-2-yl)­propan-2-yl)­propanamide (3.8 mg, 7.8 μmol, 14%) as a colorless solid. HRMS (ESI) *m*/*z*: calcd for C_28_H_31_N_4_O_5_ [M + H]^+^, 503.2289; found, 503.2272. ^1^H NMR (500 MHz, DMSO-*d*_6_): δ 8.49–8.47 (m, 1H), 8.29 (d, *J* = 8.2 Hz, 1H), 8.15 (t, *J* = 5.5 Hz, 1H), 8.02–7.98 (m, 3H), 7.69–7.65 (m, 2H), 7.54 (t, *J* = 7.8 Hz, 2H), 7.28 (d, *J* = 7.7 Hz, 1H), 7.22 (ddd, *J* = 7.5, 4.9, 0.8 Hz, 1H), 7.13–7.10 (m, 2H), 6.80–6.77 (m, 2H), 4.82–4.78 (m, 1H), 4.65–4.56 (m, 2H), 4.43–4.38 (m, 1H), 3.68 (s, 3H), 3.23 (dd, *J* = 14.2, 4.9 Hz, 1H), 3.02 (dd, *J* = 14.3, 9.2 Hz, 1H), 2.89 (dd, *J* = 13.9, 4.5 Hz, 1H), 2.61 (dd, *J* = 14.0, 10.0 Hz, 1H), 1.72 (s, 3H). ^13^C NMR (126 MHz, DMSO-*d*_6_): δ 194.9, 171.3, 171.3, 169.1, 157.7, 157.6, 148.9, 136.3, 134.9, 133.6, 130.1, 129.8, 128.8, 127.8, 123.6, 121.7, 113.4, 54.9, 54.1, 52.5, 46.0, 39.2, 36.5, 22.4.

### log *D*_7.4_, Aqueous Solubility and Cell Permeability

log *D*_7.4_,^30^ aqueous solubility in PBS at pH 7.4,^30^ and permeability across Caco-2 cell monolayers^24^ were determined for all compounds using the procedures reported previously in the cited articles. In brief, log *D*_7.4_ was determined by sampling of the octanol and PBS phases using an automated version of the shake-flask methodology, while aqueous solubility was determined by the concentration of a DMSO stock solution of the compound under vacuum, followed by resolubilization in PBS buffer at pH 7.4. Passive, transcellular permeability across a Caco-2 cell monolayer was determined in the apical-to-basolateral direction using an optimized cocktail consisting of quinidine, benzbromarone, and sulfasalazine as inhibitors of the three major efflux transporters ABCB1, ABCC2, and ABCG2.

### p*K*_a_

Acid dissociation constants were measured potentiometrically using a SiriusT3 instrument (Sirius Analytical Instruments), as reported previously.^51^

### Single Crystal X-ray Diffraction and Structural Refinements

Single crystals of compound **1g**, **1j**, **2c**, and **21c** were grown by the solvent evaporation method using trichloroethane (for **1g**), chlorobenzene (**1j** and **2c**), acetonitrile (**21c**), and acetone (**5c**) as solvents. Suitable single crystals of **1g**, **1j,** and **2c** were mounted on a Rigaku 007HF diffractometer (Rigaku, Japan) equipped with Varimax confocal mirrors, an AFC11 goniometer, and a HyPix 6000 detector (Rigaku, Japan) and an Oxford Cryostream 800 (Oxford Cryosystem, UK). The data were recorded using Cu Kα radiation generated from a microfocus rotating anode (40 kV, 30 mA) at 100 K. Suitable single crystals of **21c** and **5c** were mounted on an XtaLab Synergy-S diffractometer (Rigaku, Japan) equipped with a HyPix-Arc 100 curve detector (Rigaku, Japan) and an Oxford Cryostream 800 (Oxford Cryosystem, UK). Data were recorded using Cu Kα radiation generated from a microfocus sealed tube (50 kV, 1 mA) at 100 K. The measurement strategy was calculated using CrysAlisPro software.^52^

Data reduction and correction were performed using the CrysAlisPro software,^52^ where numerical absorption correction based on Gaussian integration over a multifaceted crystal model and empirical absorption correction using spherical harmonics, implemented in the SCALE3 ABSPACK scaling algorithm, were used. For each of the compounds, the structure was solved with the ShelXT^53^ structure solution program using the direct methods solution method within Olex2.^54^ The model was refined on *F*_o_^2^ with ShelXL 2014.^55^ All non-hydrogen atoms were refined anisotropically. CCDC 2537476–2537478, 2553542, and 2556094 contain the supplementary crystallographic data for **1g**, **2c**, **21c**, **1j**, and **5c**, respectively. These data can be obtained free of charge via www.ccdc.cam.ac.uk/data_request/cif, by emailing data_request@ccdc.cam.ac.uk, or by contacting The Cambridge Crystallographic Data Centre, 12 Union Road, Cambridge CB2 1EZ, UK; fax: +44 1223 336033.

### Energy Minimization of Crystal Structures

The crystal structures of macrocycles **1g** and **2c** were energy-minimized with B3LYP using the Jaguar tool from the Schrödinger suite, as previously reported for **1c**.^18^ The total number of iterations was set to 100, and the Poisson–Boltzmann finite-based solvation model for chloroform was used. Other settings were kept at their default values. An energy-minimized structure of **3c** was obtained by conversion of the Boc group of **21c** to an acetyl group, followed by minimization using the same protocol as for **1g** and **2c**.

### Conformational Analysis

Conformational analysis of linear analogue **5c** was performed using Monte Carlo multiple-minimum conformational sampling essentially as reported previously by Sethio et al.^20^ Briefly, a nonpolar environment (CHCl_3_, ε = 4.8) was chosen as **5c** had been studied in CDCl_3_ by NMR spectroscopy and to mimic the interior of the plasma membrane. The following parameters were used: an energy window of 10 kcal/mol, an RMSD threshold of 0.75 Å for duplicate conformer elimination, a total of 10,000 iterations, and geometry optimization using the Polak–Ribiere Conjugate Gradient algorithm in combination with the OPLS3e force field. This provided a total of 589 conformers for **5c**.

A property-based hierarchical clustering approach was applied to reduce the number of conformers prior to DFT geometry optimization. Specifically, for each Monte Carlo-generated conformation, the radius of gyration (*R*_gyr_) and the solvent-accessible 3D polar surface area (SA 3D PSA) were calculated using VegaZZ. These descriptors were used to construct an *n* × 2 matrix, where *n* corresponds to the number of conformers. The Euclidean distance in this two-dimensional property space was computed to quantify the distance *d*(*i*,*q*) between conformers. This distance was then converted into a similarity metric defined as

$$s\left(i,q\right)=1-\frac{d\left(i,q\right)}{d_{\max}}$$

where *d*_max_ represents the maximum possible distance between any two conformers. The similarity values range from 0 to 1. The resulting similarity matrix was employed for agglomerative hierarchical clustering, in which each conformer initially represents an individual cluster. Clusters were iteratively merged using the Ward linkage method, which minimizes the increase in intracluster variance at each step. The final number of clusters (*k*) was set to 10. From each cluster, the minimum-energy conformer (MEC) was selected, as calculated by the OPLS3e force field.

Geometry optimization of the 10 representative conformers was also performed essentially as reported by Sethio et al.^20^ In summary, the 10 conformers were subjected to DFT calculations using the M06-2X functional in combination with Pople’s 6-31+G­(d,p) to accurately describe nonbonded interactions. Solvent effects were modeled using the conductor-like polarizable continuum model, with chloroform (CHCl_3_) as the solvent. To ensure the optimized geometry corresponds to a minimum on the potential energy surface, vibrational frequency calculations were conducted at the same level of theory. For each conformer, the Gibbs free energy was obtained from frequency analysis. Relative free energies were then calculated by referencing each Gibbs free energy to the global minimum according to

$$\Delta G_{i}=G_{i}-G_{\min}$$

These Δ*G* values were used to compute the Boltzmann weights. The probability of observing each conformer was calculated by normalizing the Boltzmann weights with the partition function, defined as the sum of all Boltzmann weights.

Conformational analysis of macrocycle **1c** started from the 10 conformations obtained for its Boc-protected precursor.^20^ In each of these, the Boc group was converted into an acetyl group, after which each conformation was energy-minimized, first using the OPLS3e force field and then at the DFT level as described above.

### Calculation of 3D Descriptors

The solvent-accessible 3D polar surface area (SA 3D PSA) and the solvent-accessible 3D nonpolar surface area (SA 3D NPSA), both defined using a solvent probe radius of 1.4 Å, as well as the *R*_gyr_, were calculated using VEGA ZZ (Release 3.2.3).^56^

### VT NMR Spectroscopy

Amide temperature coefficients, ΔδNH/Δ*T* (ppb K^–1^), were determined for compounds **1c** and **5c** using a Bruker AVANCE Neo NMR spectrometer equipped with a TCI cryogenic probe operating at 600 MHz. Amide protons were first assigned by the use of ^1^H, COSY, and NOESY (*t*_mix_ = 700 ms) spectra. Then, ΔδNH/Δ*T* was determined from ^1^H NMR spectra recorded at 10–30 °C in CDCl_3_ and at 25–45 °C in DMSO-*d*_6_ by increasing the temperature by 5° between each ^1^H NMR spectrum.

## Supplementary Material





## Notes

Molecular formula strings document with the structure (SMILES code), log *D*, efflux-inhibited permeability across Caco-2 cells, and aqueous solubility for each of macrocycles **1a**–**1k**, **2c**, **3c**, **3e**, **4a**–**4c**, **4e,** and **4j**, as well as linear analogues **5a**–**5g** (CSV)
^1^H and ^13^C NMR spectra for all synthesized compounds, HPLC purity analyses for all 26 tested compounds, information for the in silico conformational analysis of **1c** and **5c**, and crystallographic data for **1g**, **2c**, and **21c** (PDF)

## Abbreviations

bRo5: beyond the rule of 5
DFT: density functional theory
DIPEA: *N*,*N*-diisopropylethylamine
HATU: hexafluorophosphate azabenzotriazole tetramethyl uronium
IBX: 2-iodoxybenzoic acid
IMHB: intramolecular hydrogen bond
MECs: minimum energy conformations
NRotB: number of rotatable bonds
PyBOP: benzotriazole-1-yl-oxy-tris-pyrrolidino-phosphonium hexafluorophosphate
PyBroP: bromo-tris-pyrrolidino-phosphonium hexafluorophosphate
*R*_gyr_: radius of gyration
SA 3D NPSA: solvent-accessible 3D nonpolar surface area
SA 3D PSA: solvent-accessible 3D polar surface area
SMILES: simplified molecular input line-entry system
TBDMS: *tert*-butyldimethylsilyl
TPSA: topological polar surface area
VT: variable temperature
