# Supplementary material for: What Is in a Structure? Cell Permeability and Solubility of Series of Macrocycles and Linear Matched Pairs
Source: J Med Chem. 2026 Jun 22;69(13):15702–30. doi: 10.1021/acs.jmedchem.6c00830 (PMC13370866; doi:10.1021/acs.jmedchem.6c00830)
Supplement: Supplementary file 2 [file jm6c00830_si_002.pdf]

SUPPORTING INFORMATION

What Is in a Structure? Cell permeability and Solubility of Series of Macrocycles and Linear Matched Pairs

Mohit Tyagi,[a,b] Vasanthanathan Poongavanam,[b] Stefanie Zich,[a,c] Marika Lindhagen,[d] Ioannis Asproudis,[d] Okky Dwichandra Putra,[d] Jie Yang,[b] Zackary J. R. Ashworth,[b] Anna Guadagni,[a] Luca J. Hagemeyer,[b] Alessandro Oliva,[b] Peter Sjö,[e] Stefan Schiesser,[a] and Jan Kihlberg[b,*]

[a]Department of Medicinal Chemistry, Discovery Sciences, R&D, AstraZeneca, Gothenburg, 431 83 Mölndal, Sweden

[b]Department of Chemistry for Life Sciences, Uppsala University, Box 576, 751 23 Uppsala, Sweden

[c]Department of Chemistry and Molecular Biology, University of Gothenburg, Medicinaregatan 7b, 41390 Gothenburg, Sweden

[d]Early Product Development, Pharmaceutical Sciences, R&D, AstraZeneca, Gothenburg, 431 83 Mölndal, Sweden

[e]Drugs for Neglected Diseases initiative (DNDi), 15 Chemin Louis Dunant, 1202 Geneva, Switzerland

Corresponding author

Jan Kihlberg, jan.kihlberg@kemi.uu.se, ORCID: 0000-0002-4205-6040

Contents

NMR spectra of synthesized compounds 3
Table S1. Calculated descriptors for macrocycles and linear compounds 143
Table S2. Crystallographic data for compounds 1g, 2c and 21c 144
Table S3. Calculated data for the conformations in the in silico ensembles of macrocycle 1c and linear matched pair 5c 145
Crystal structures 146
Conformational analysis of 1c and 5c 155
References 158

NMR spectra of synthesized compounds

*tert-Butyl ((2S)-1-((2-(4-fluoro-3-nitrophenyl)-2-hydroxyethyl)amino)-4-methyl-1-oxopentan-2-yl)carbamate (**9e**).*

^{1}H NMR in $CDCl_3$

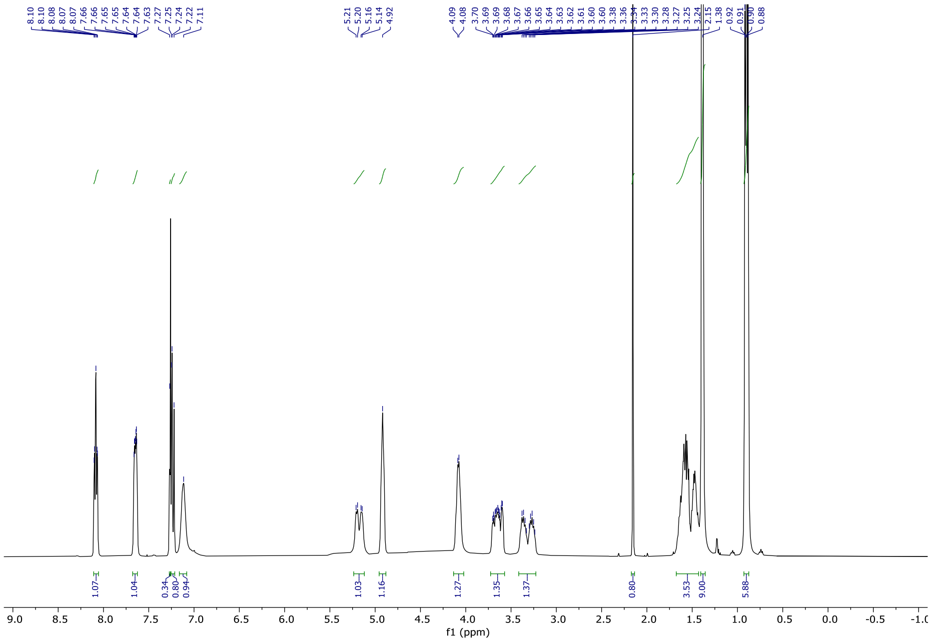

^{1}H NMR in $CDCl_3$ (expanded view)

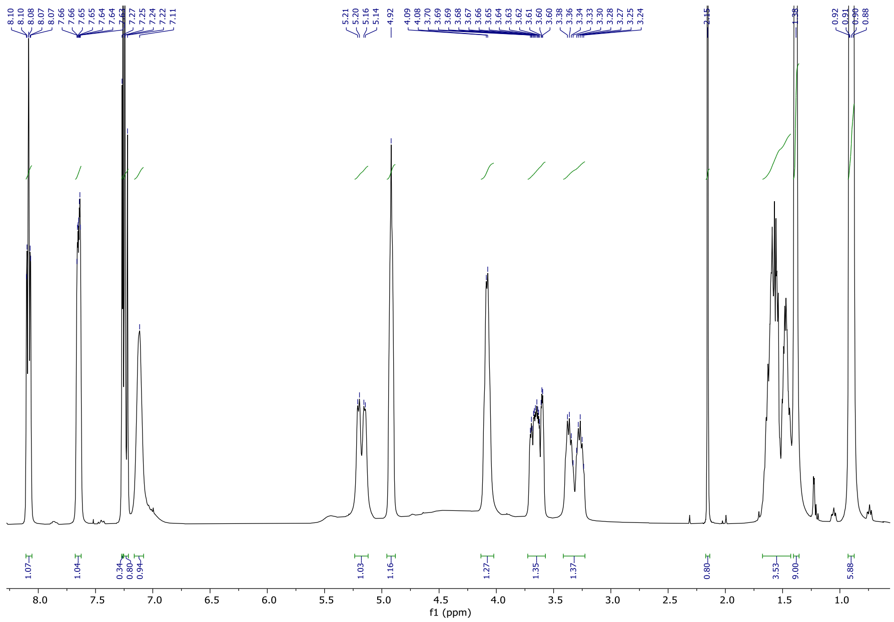

^{13}C NMR in $CDCl_3$

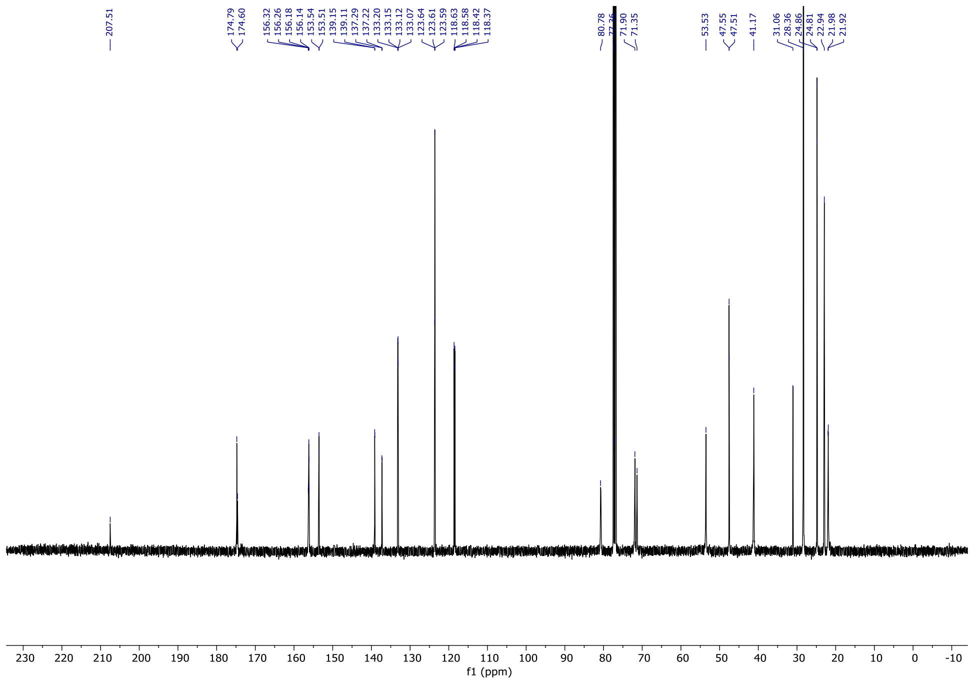

COSY in $CDCl_3$

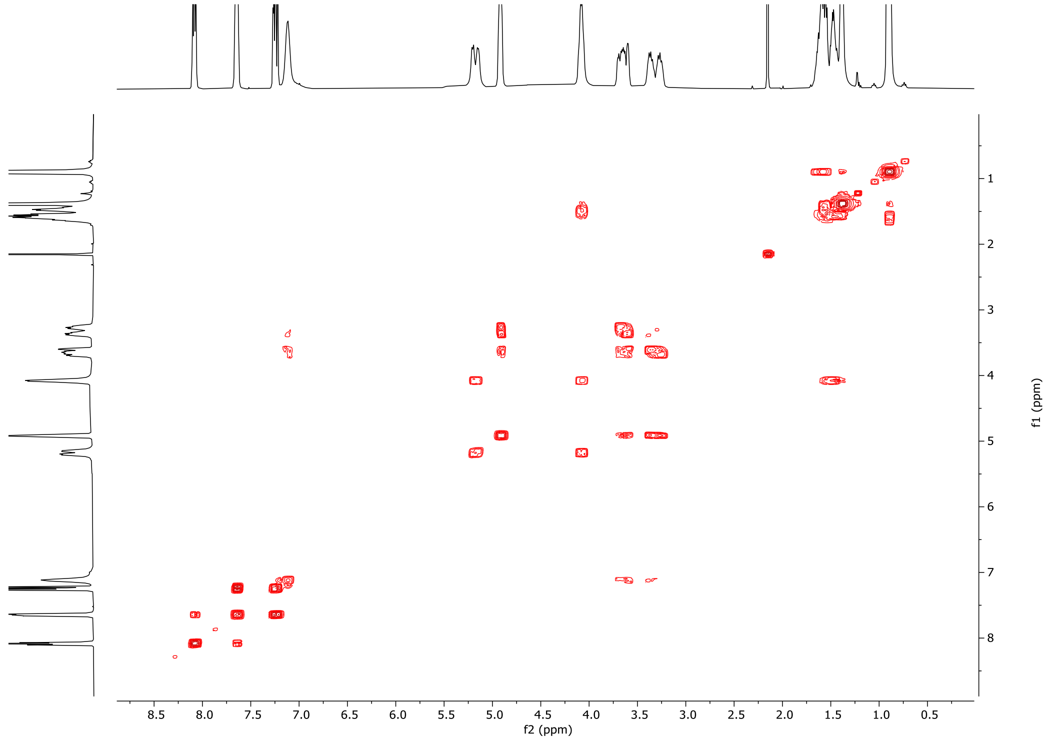

HSQC in $CDCl_3$

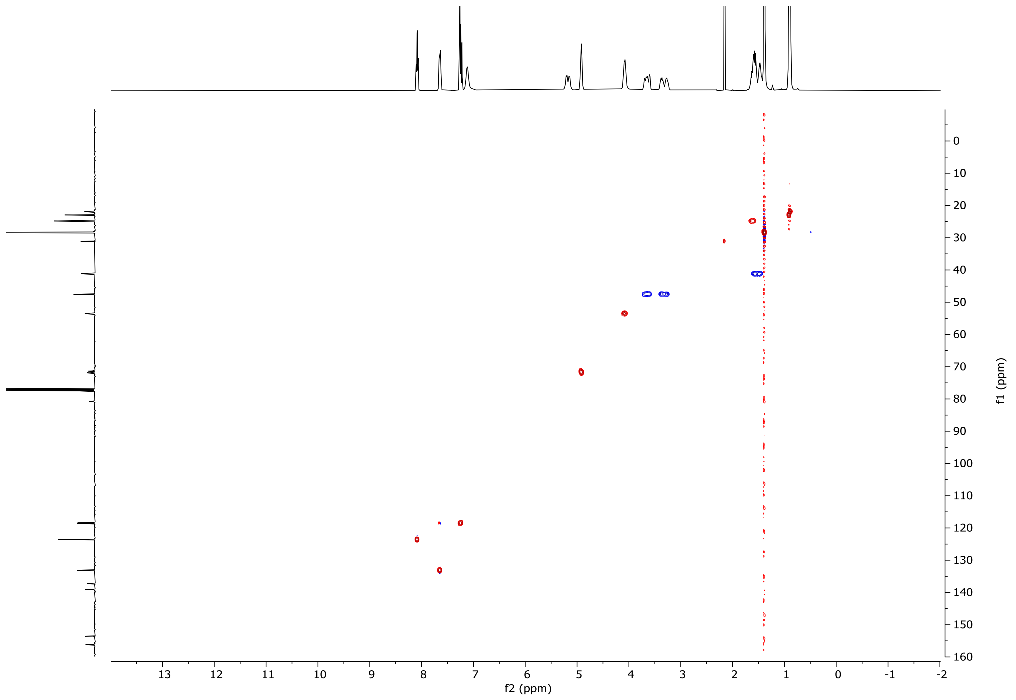

*tert-Butyl ((2S)-1-((2-(4-fluoro-3-nitrophenyl)-2-hydroxyethyl)amino)-1-oxo-3-(p-tolyl)propan-2-yl)carbamate (**9h**).*

^{1}H NMR in $CDCl_3$

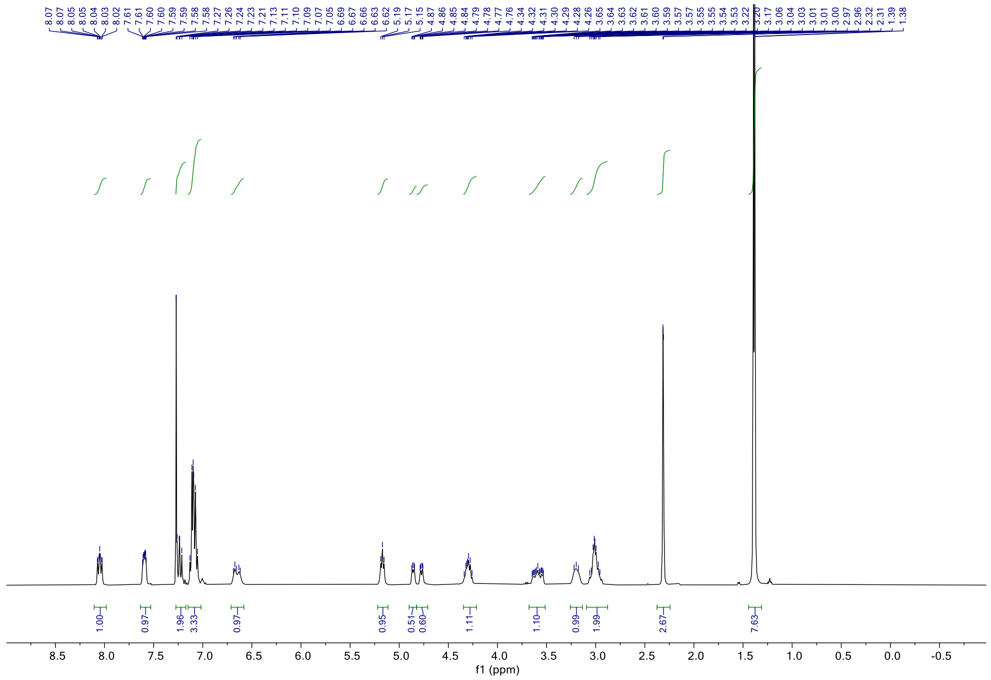

^{1}H NMR in $CDCl_3$ (expanded view)

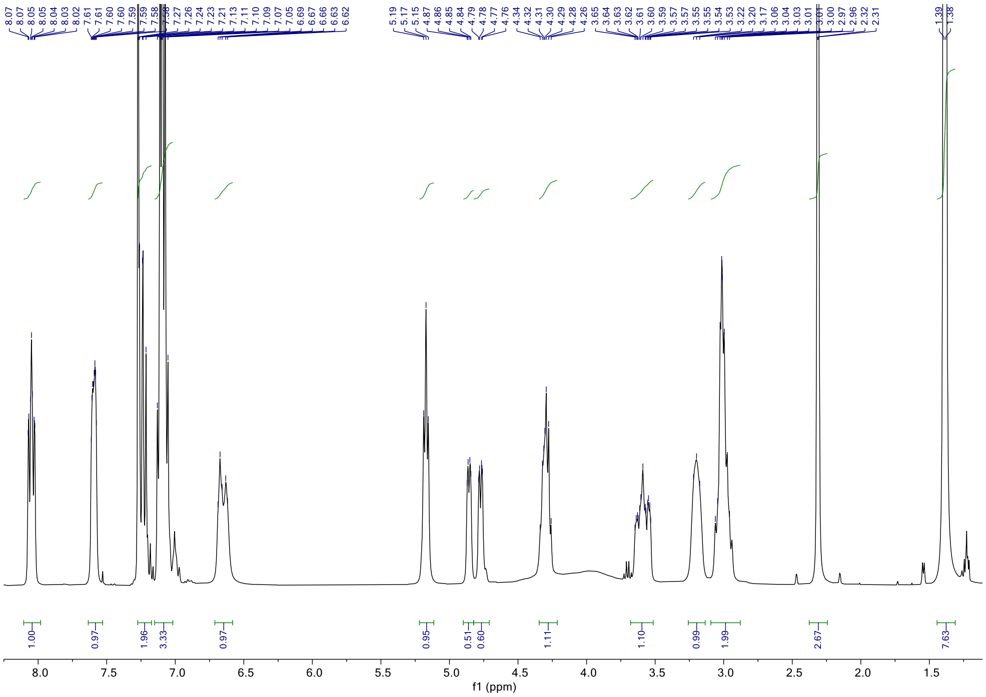

^{13}C NMR in $CDCl_3$

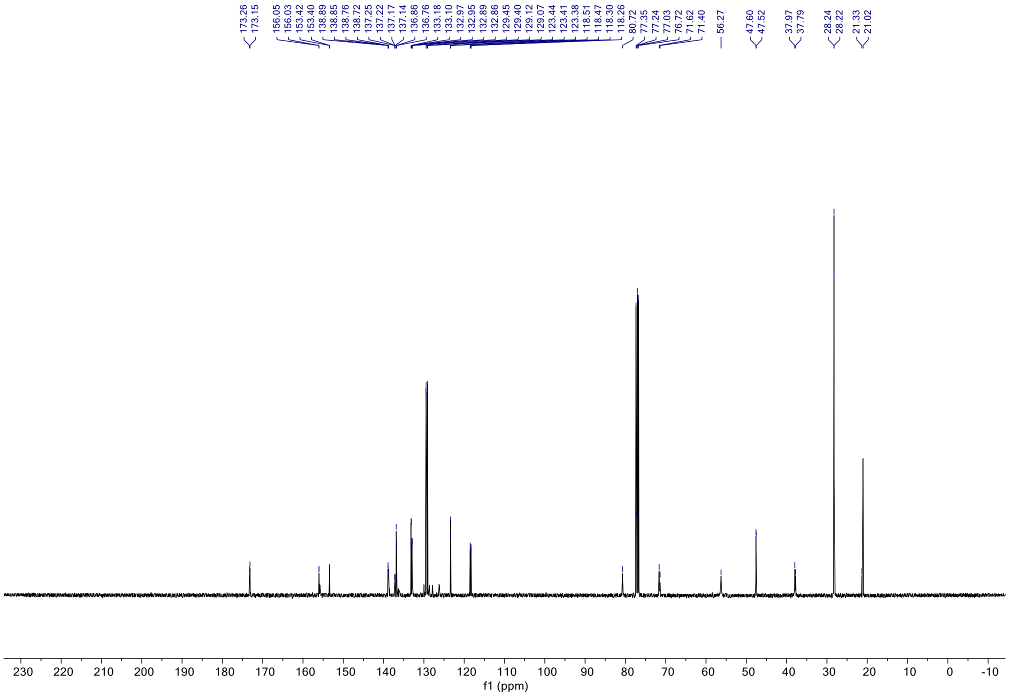

^{13}C NMR in $CDCl_3$ (expaned view)

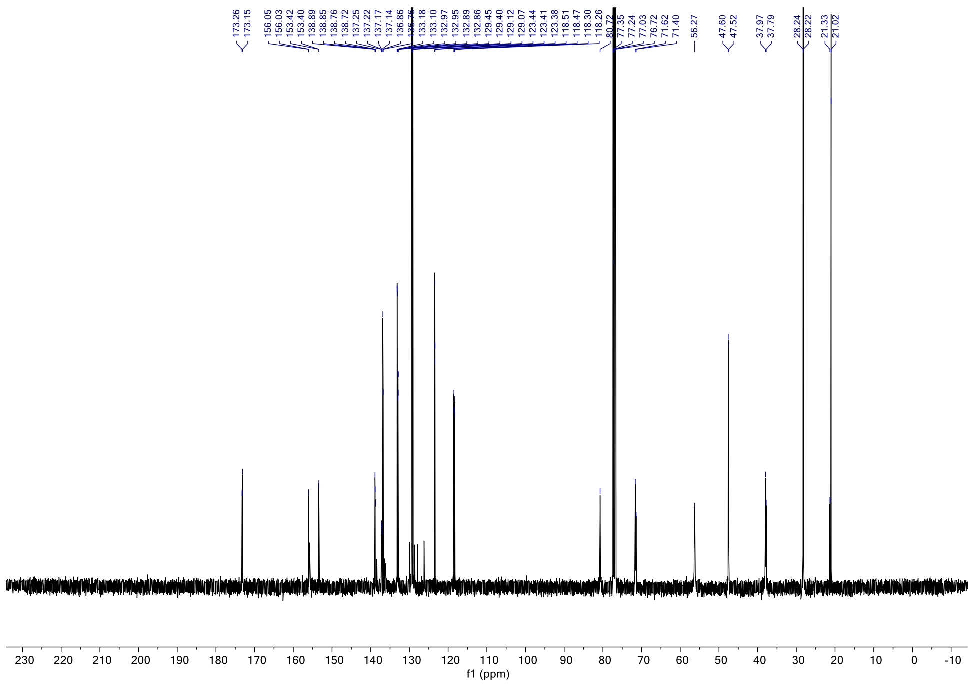

COSY in $CDCl_3$

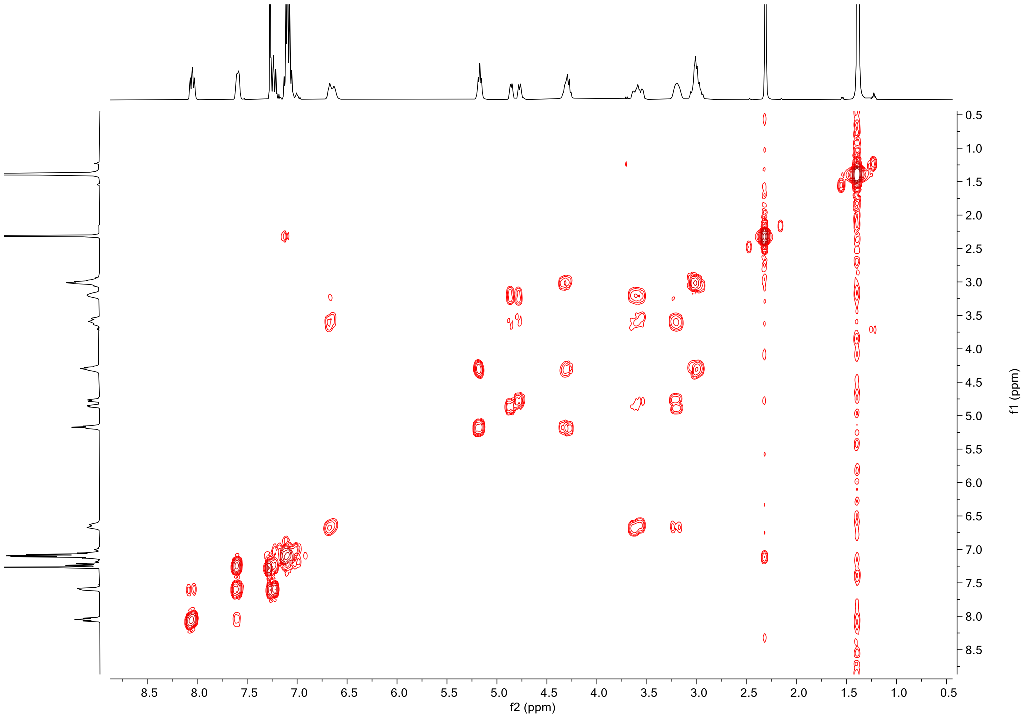

HSQC in $CDCl_3$

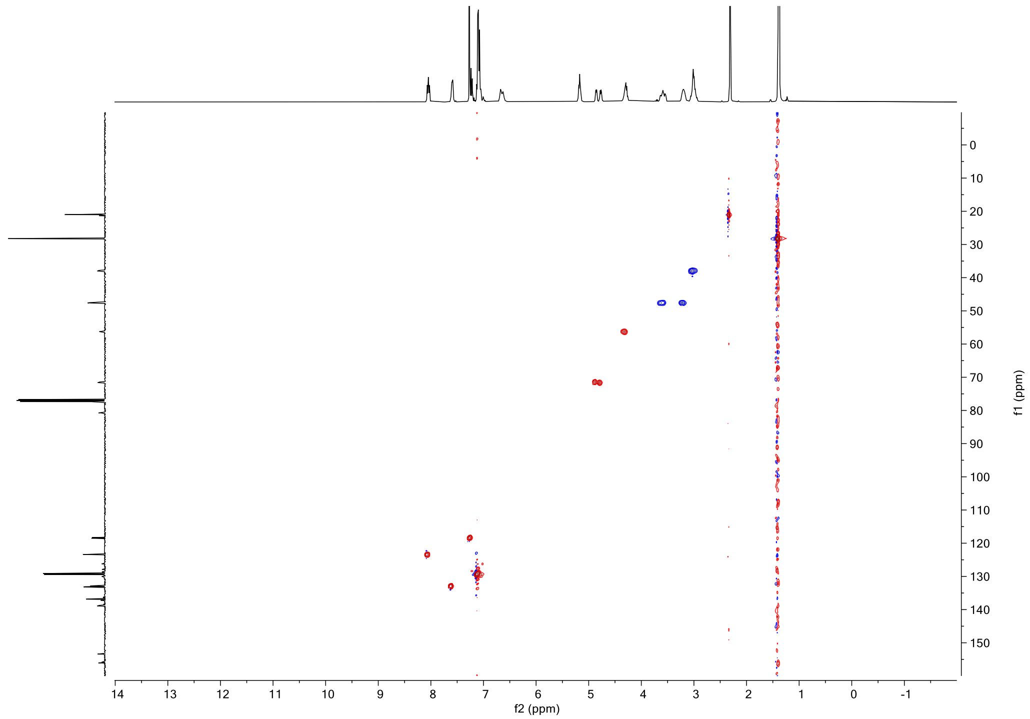

*tert-Butyl ((2S)-1-((2-(4-fluoro-3-nitrophenyl)-2-hydroxyethyl)amino)-3-(4-fluorophenyl)-1-oxopropan-2-yl)carbamate (**9i**).*

^{1}H NMR in $CDCl_3$

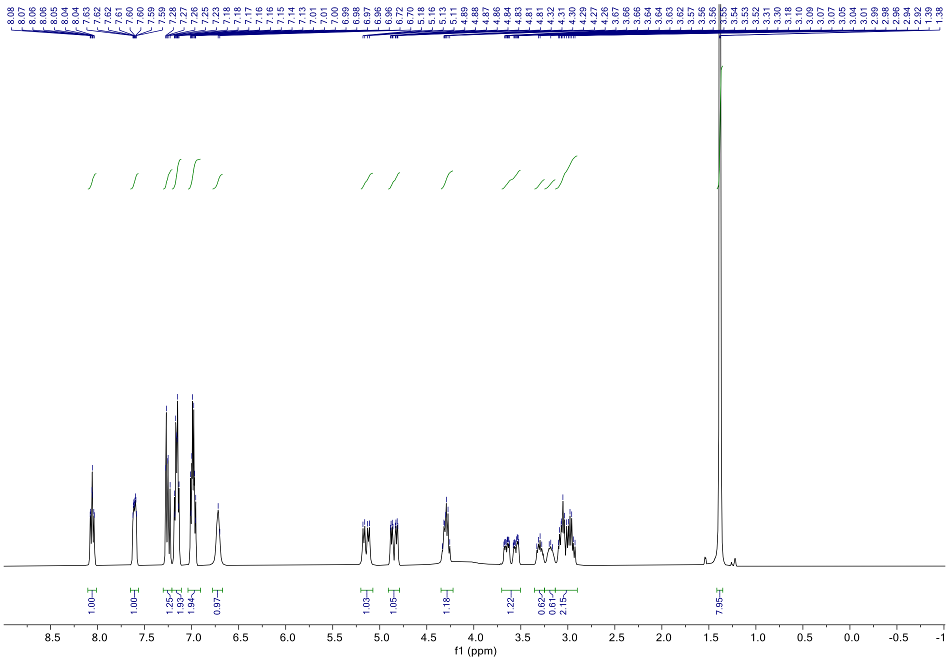

^{1}H NMR in $CDCl_3$ (expanded view)

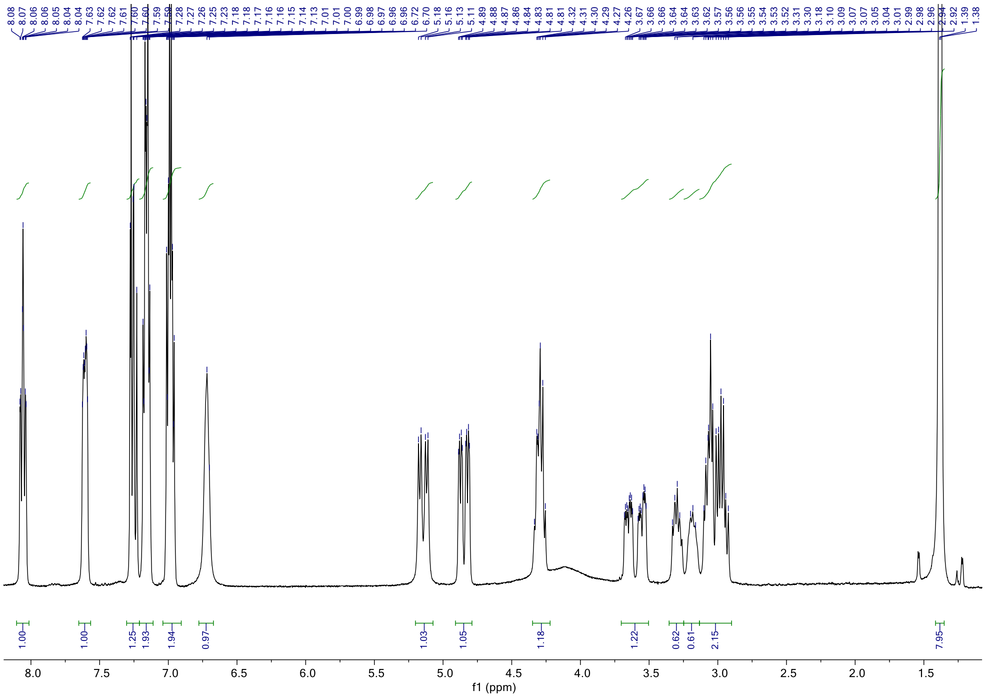

^{13}C NMR in $CDCl_3$

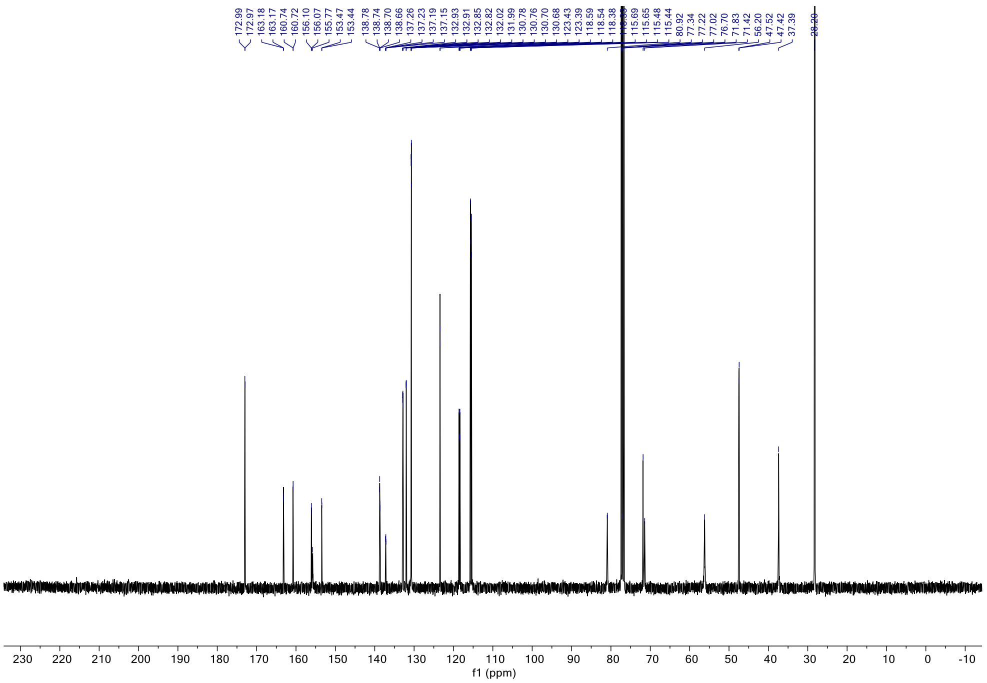

COSY in $CDCl_3$

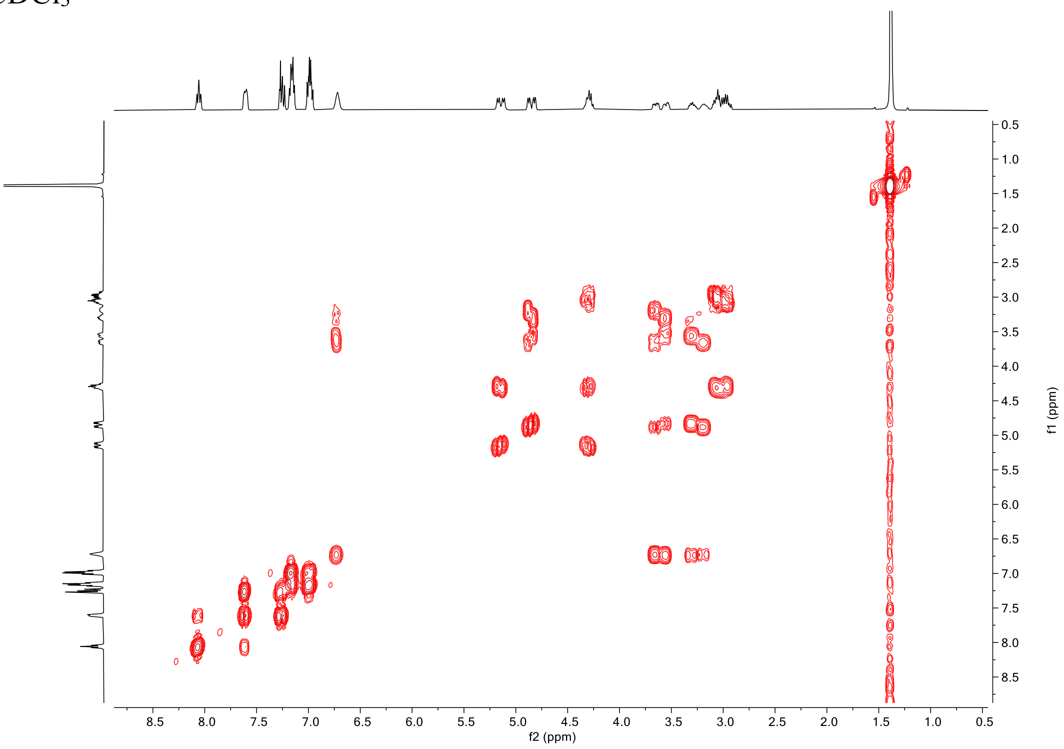

HSQC in $CDCl_3$

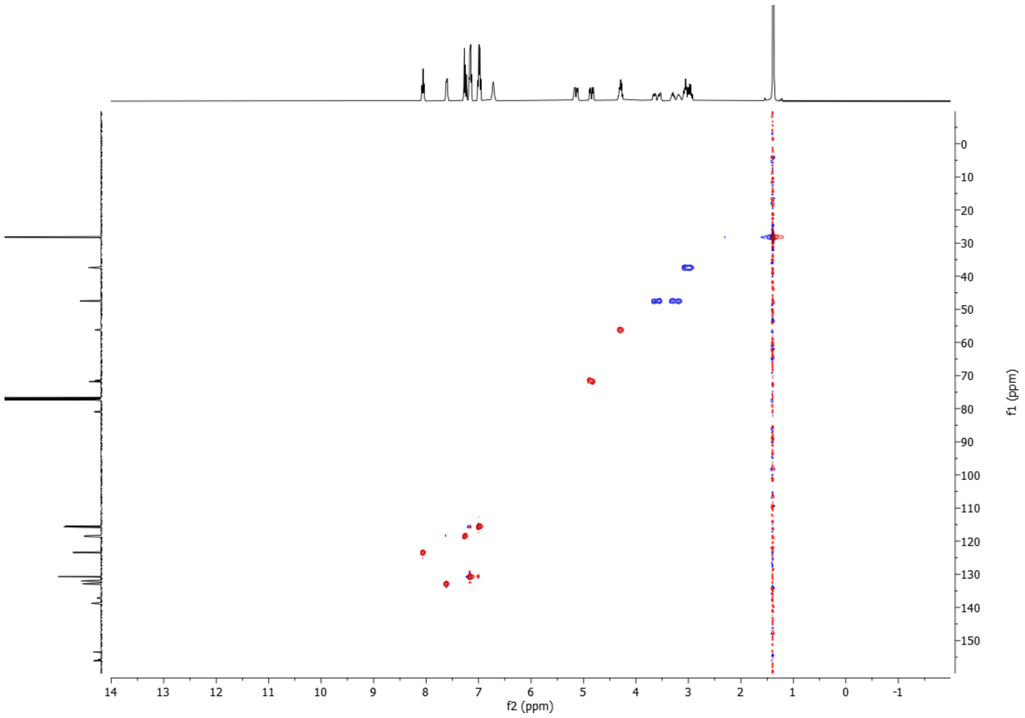

*tert-Butyl ((2S)-4-((2-(4-fluoro-3-nitrophenyl)-2-hydroxyethyl)amino)-3-oxo-1-phenylbutan-2-yl)carbamate (**10c**)*.

^{1}H NMR in DMSO-d$_6$

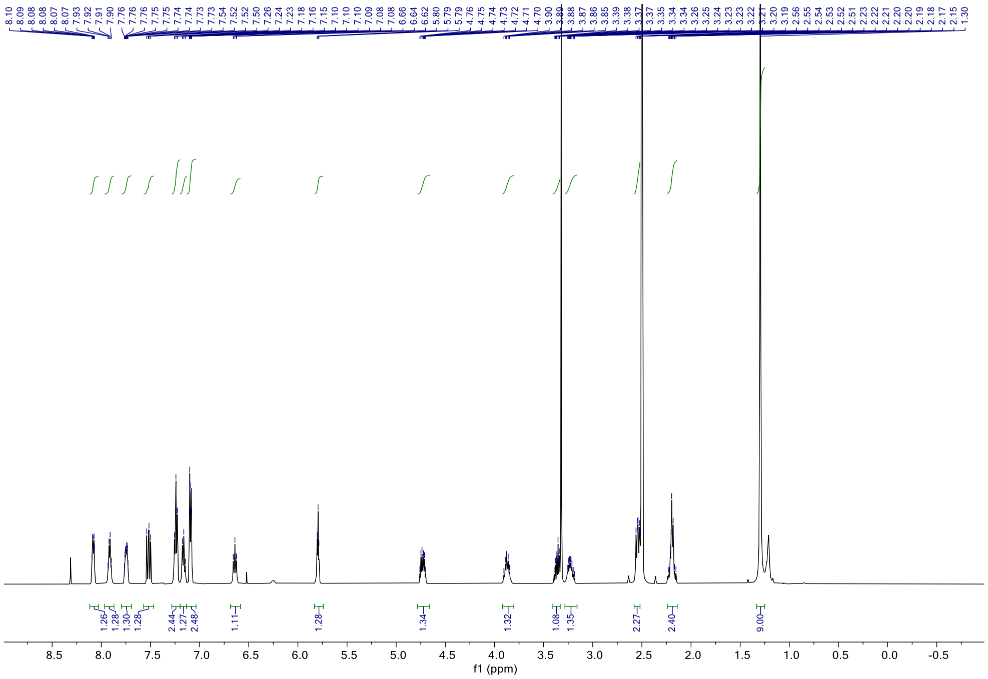

^{1}H NMR in DMSO-d$_6$ (expanded view)

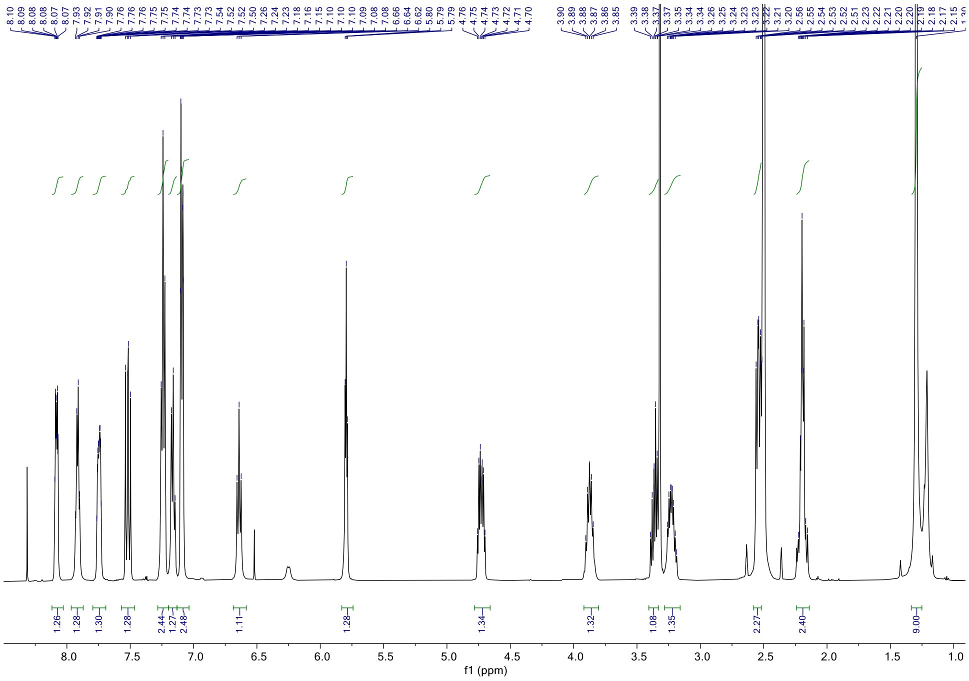

^{13}C NMR in DMSO-d$_6$

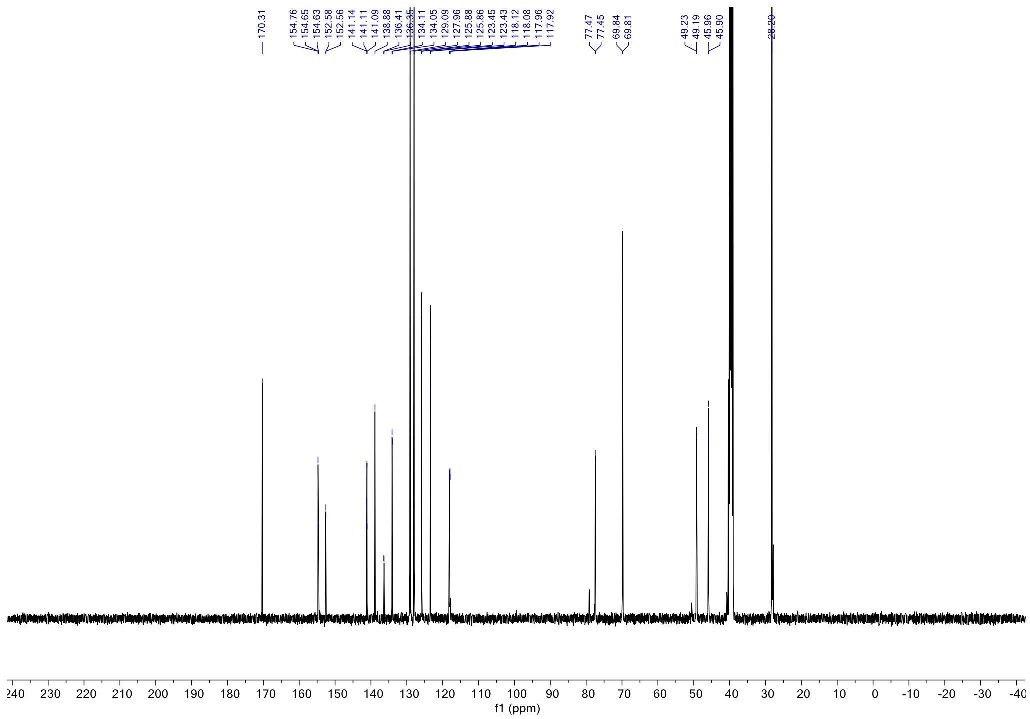

*tert-Butyl ((3S)-1-((2-(4-fluoro-3-nitrophenyl)-2-hydroxyethyl)amino)-5-methyl-2-oxohexan-3-yl)carbamate (**10e**).*

^1H NMR in DMSO-d$_6$

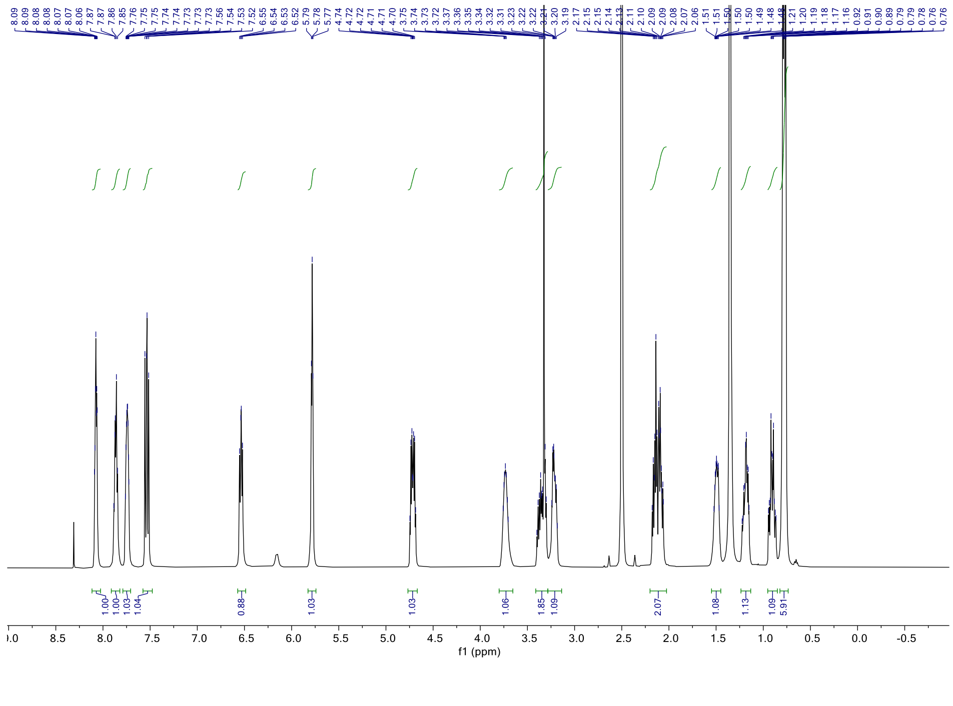

^{13}C NMR in DMSO-d$_6$

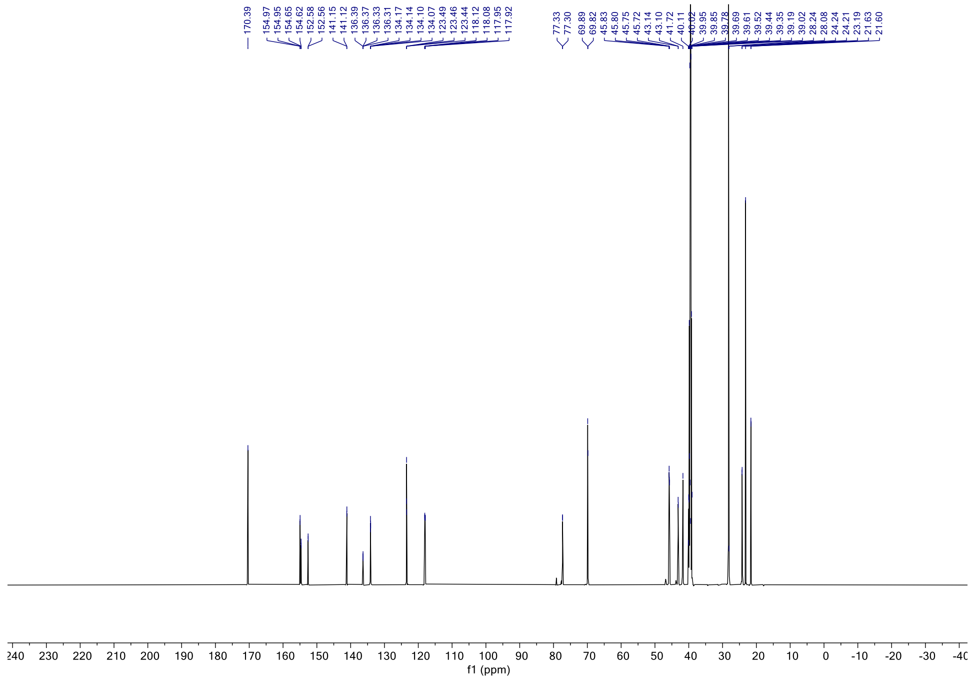

*tert-Butyl ((2S)-3-(4-((tert-butyldimethylsilyl)oxy)phenyl)-1-(((2S)-1-((2-(4-fluoro-3-nitrophenyl)-2-hydroxyethyl)amino)-4-methyl-1-oxopentan-2-yl)amino)-1-oxopropan-2-yl)carbamate **(13e)***.

^{1}H NMR in CDCl$_3$

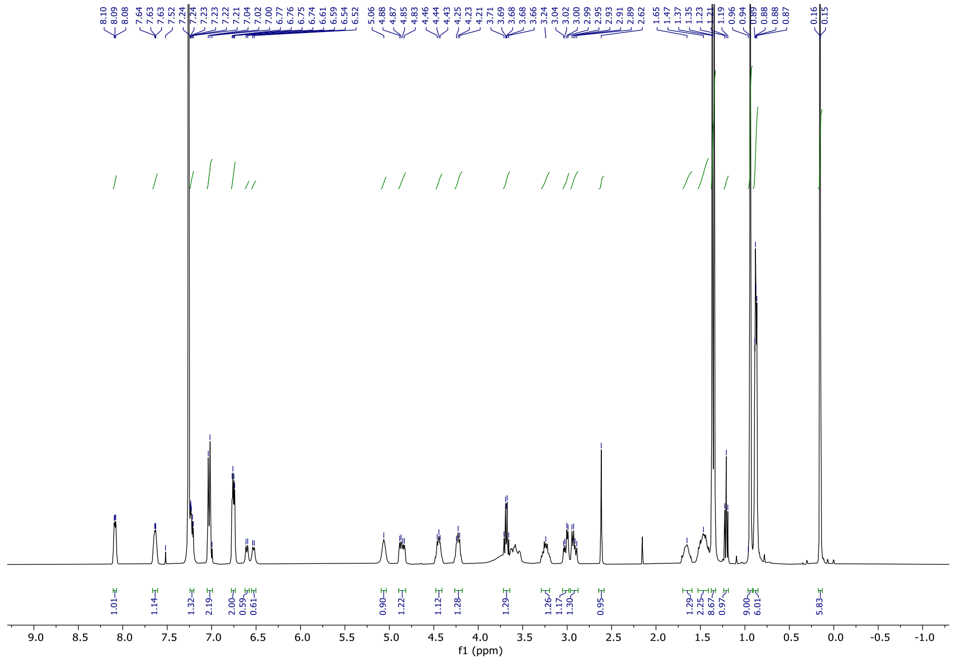

^{1}H NMR in $CDCl_3$ (expanded view)

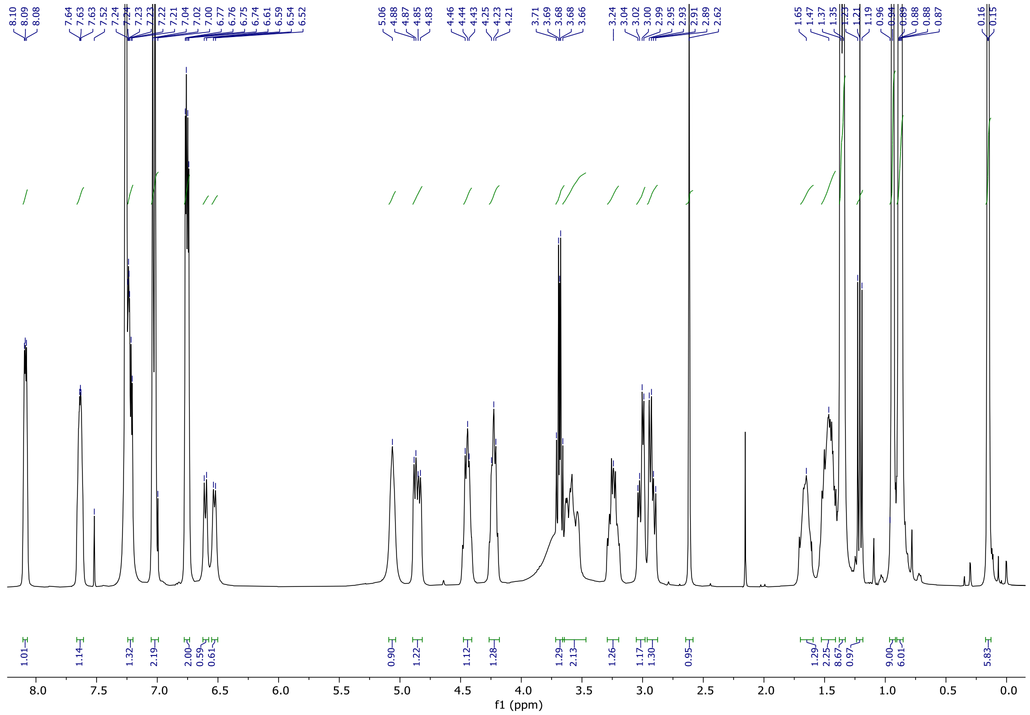

^{13}C NMR in $CDCl_3$

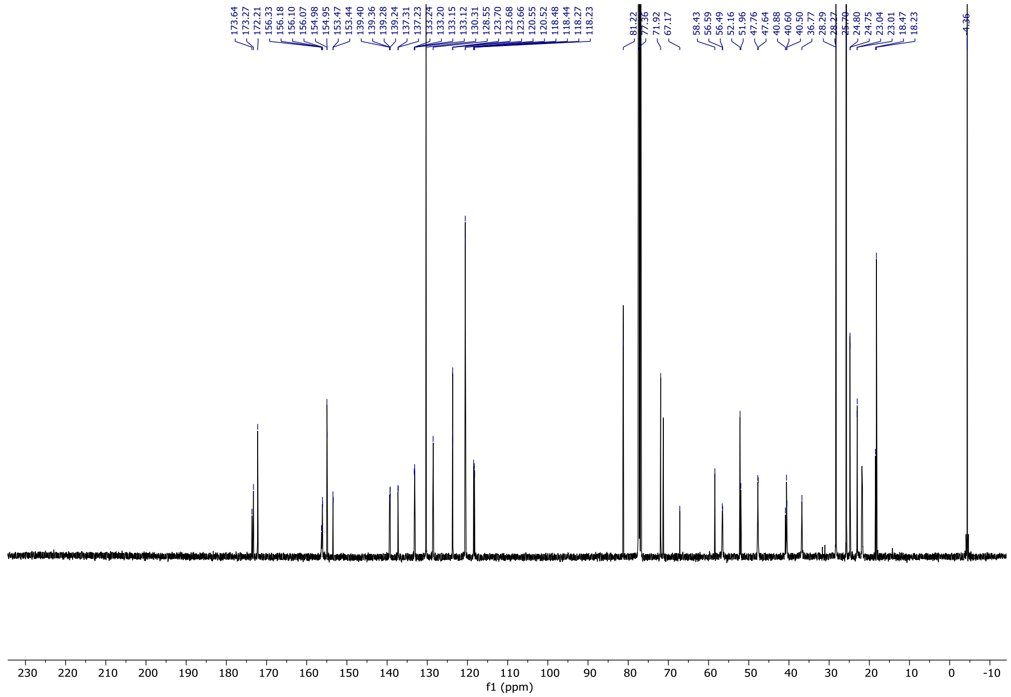

COSY in $CDCl_3$

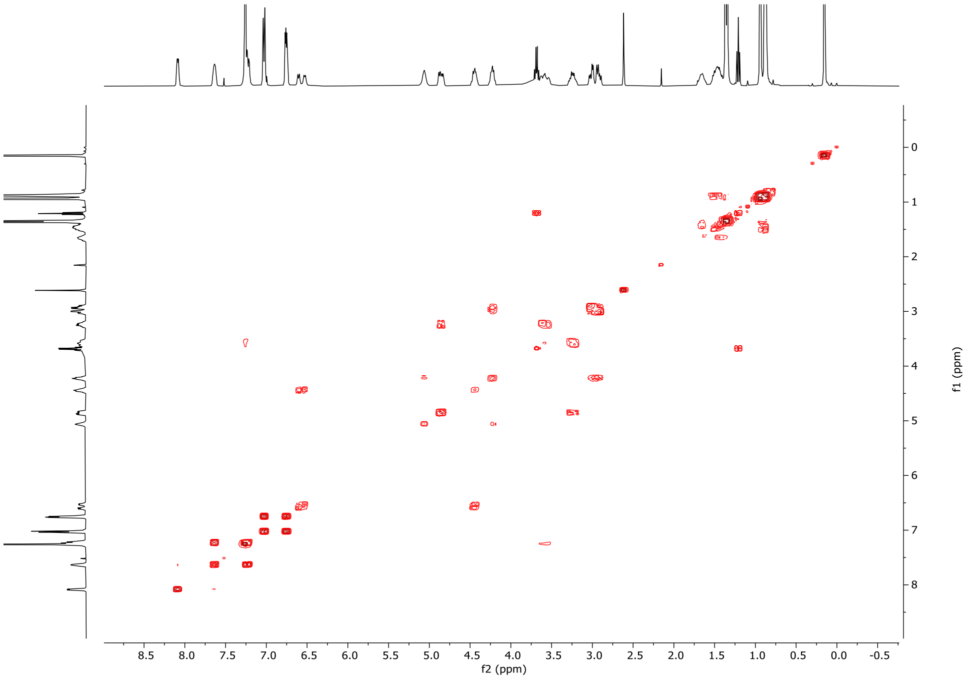

HSQC in $CDCl_3$

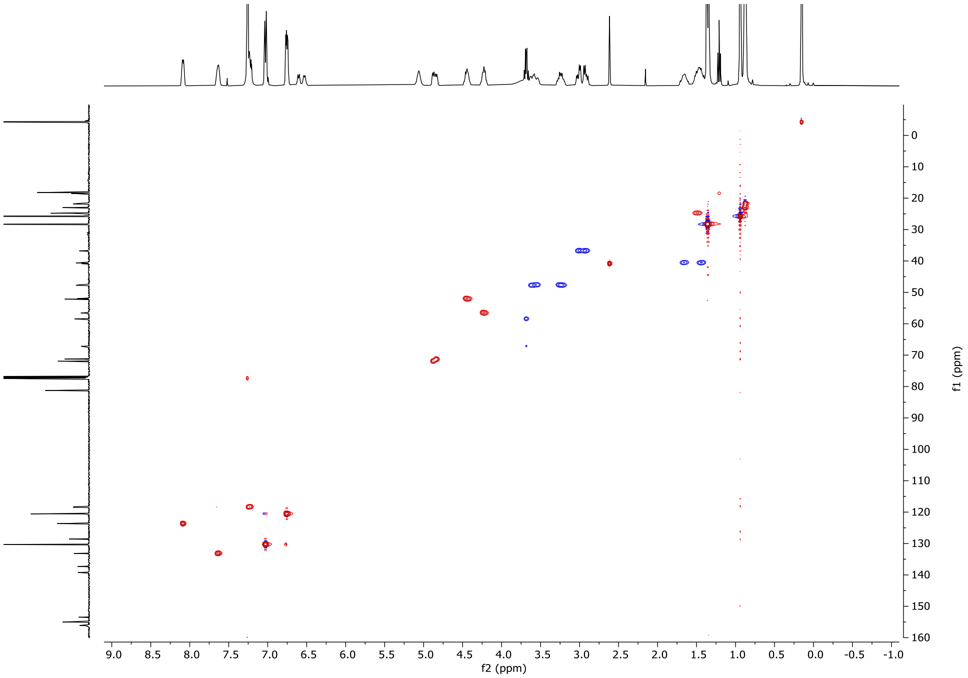

tert-Butyl ((2S)-3-(4-((tert-butyldimethylsilyl)oxy)phenyl)-1-(((2S)-1-((2-(4-fluoro-3-nitrophenyl)-2-hydroxyethyl)amino)-1-oxo-3-(p-tolyl)propan-2-yl)amino)-1-oxopropan-2-yl)carbamate ***(13h)***.

^{1}H NMR in $CDCl_3$

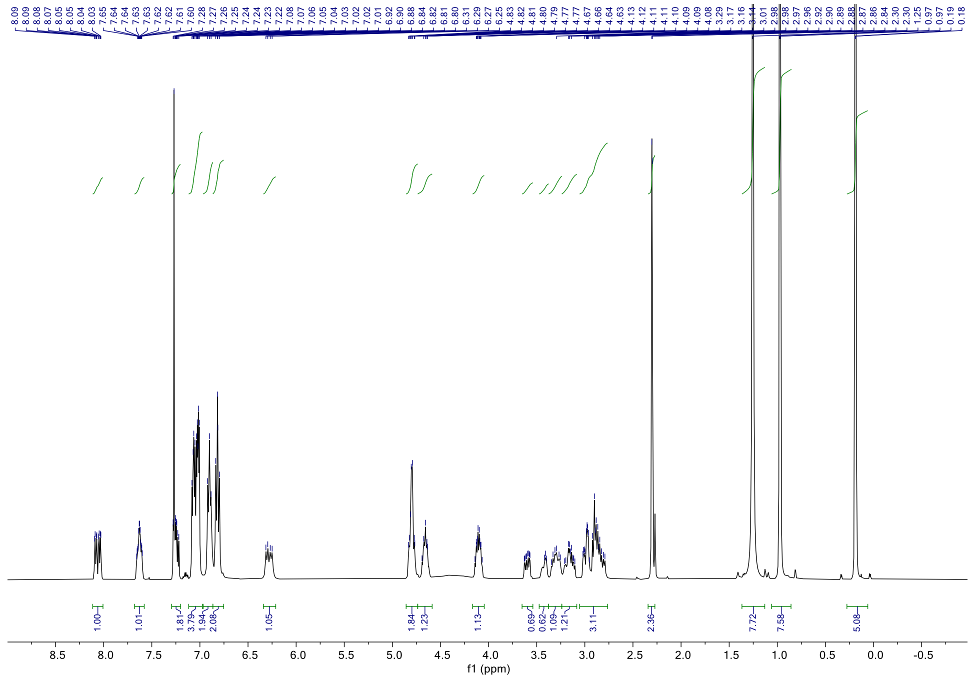

^{1}H NMR in $CDCl_3$ (expanded view)

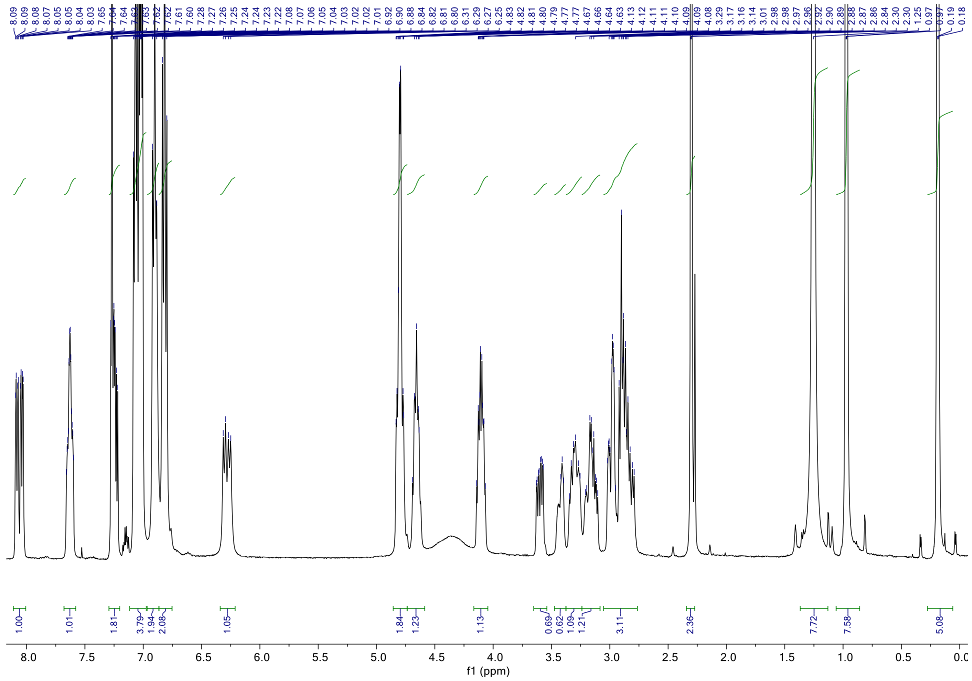

^{13}C NMR in $CDCl_3$

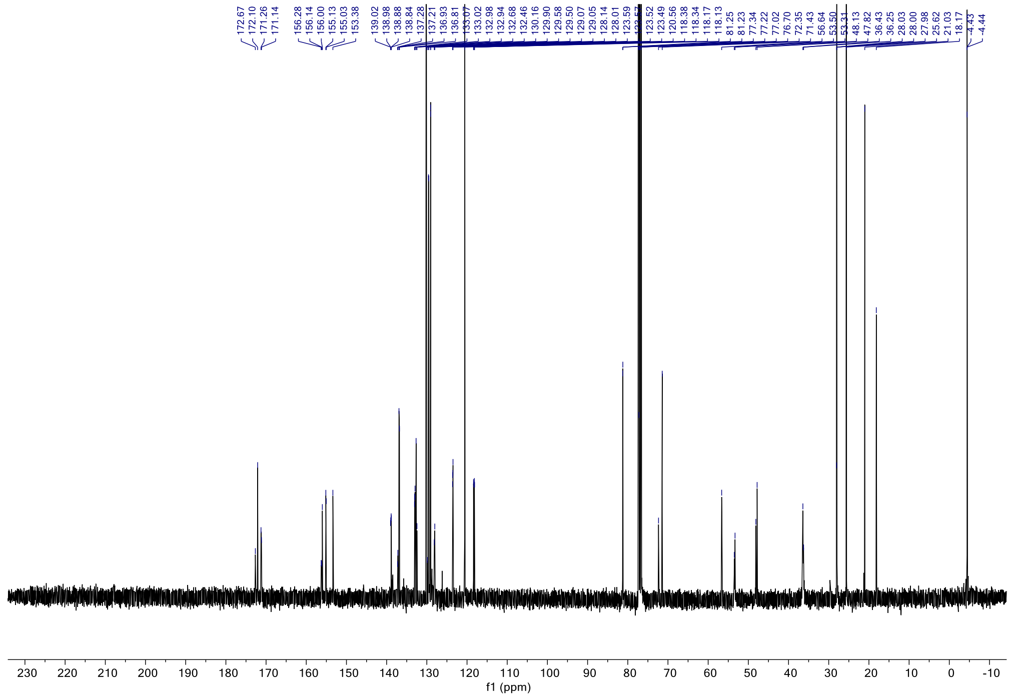

COSY in $CDCl_3$

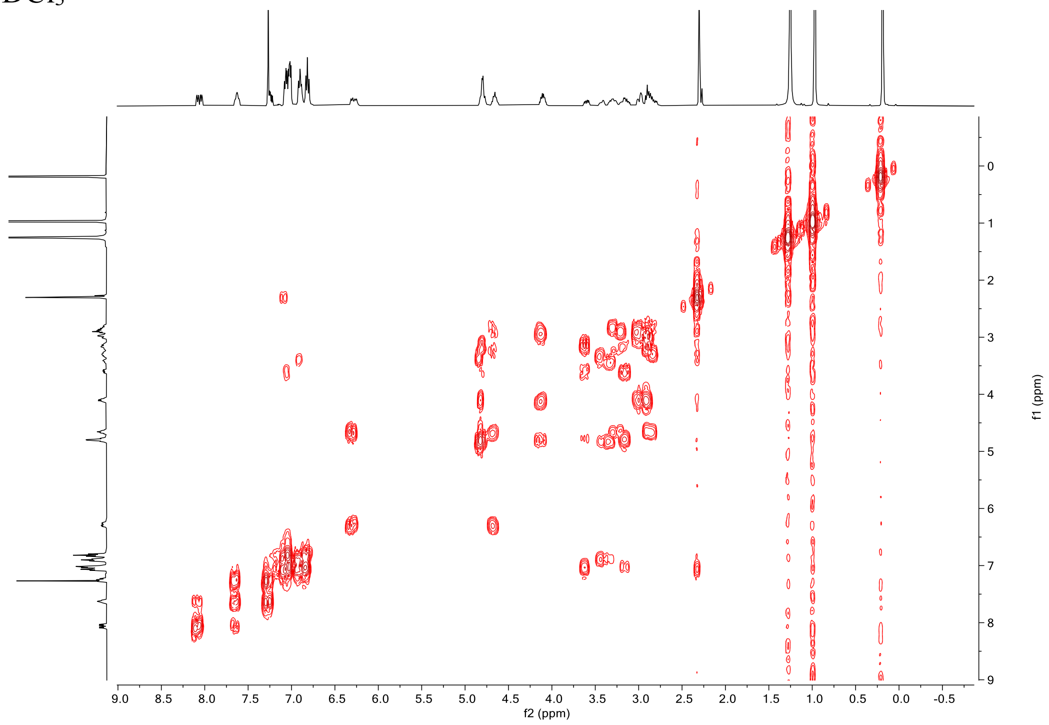

HSQC in $CDCl_3$

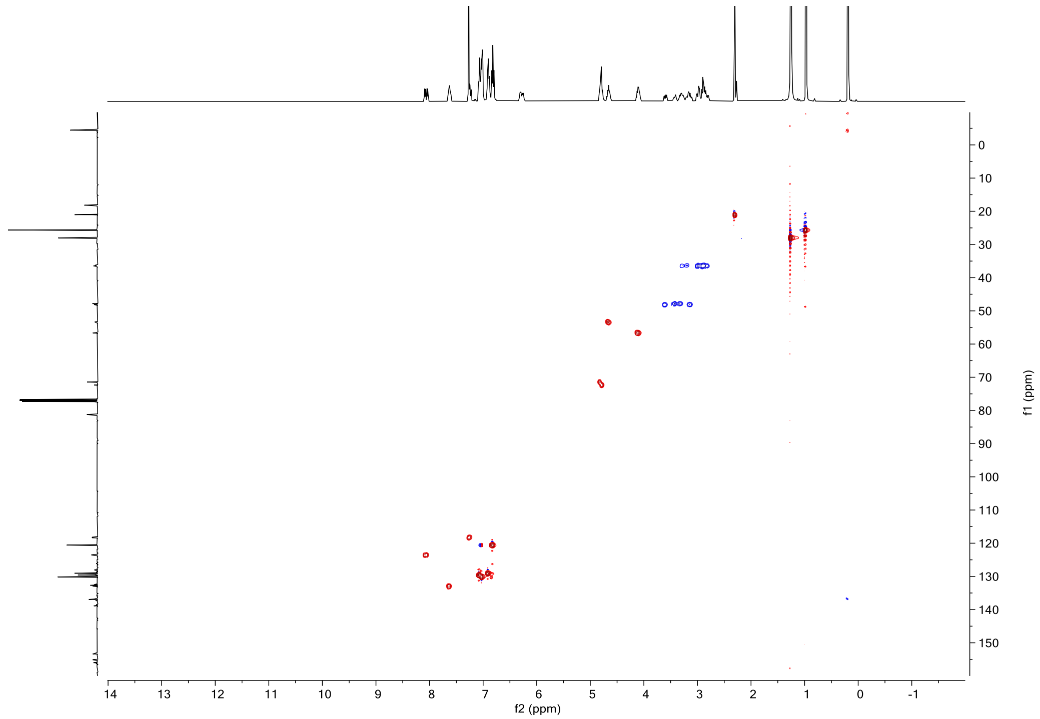

*tert-Butyl ((2S)-3-(4-((tert-butyldimethylsilyl)oxy)phenyl)-1-(((2S)-1-((2-(4-fluoro-3-nitrophenyl)-2-hydroxyethyl)amino)-3-(4-fluorophenyl)-1-oxopropan-2-yl)amino)-1-oxopropan-2-yl)carbamate **(13i)***.

^{1}H NMR in $CDCl_3$

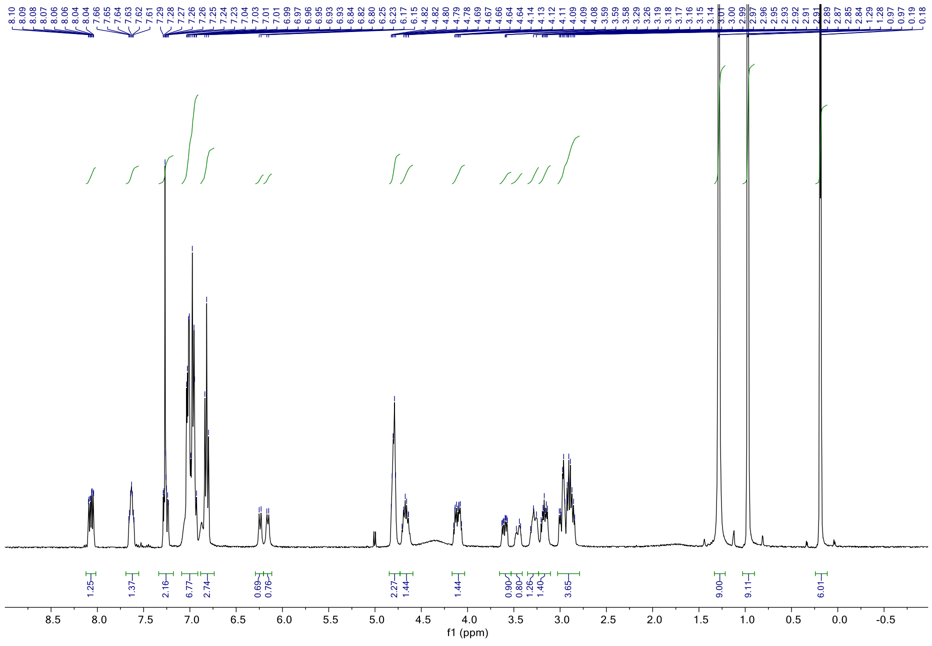

^{1}H NMR in $CDCl_3$ (expanded view)

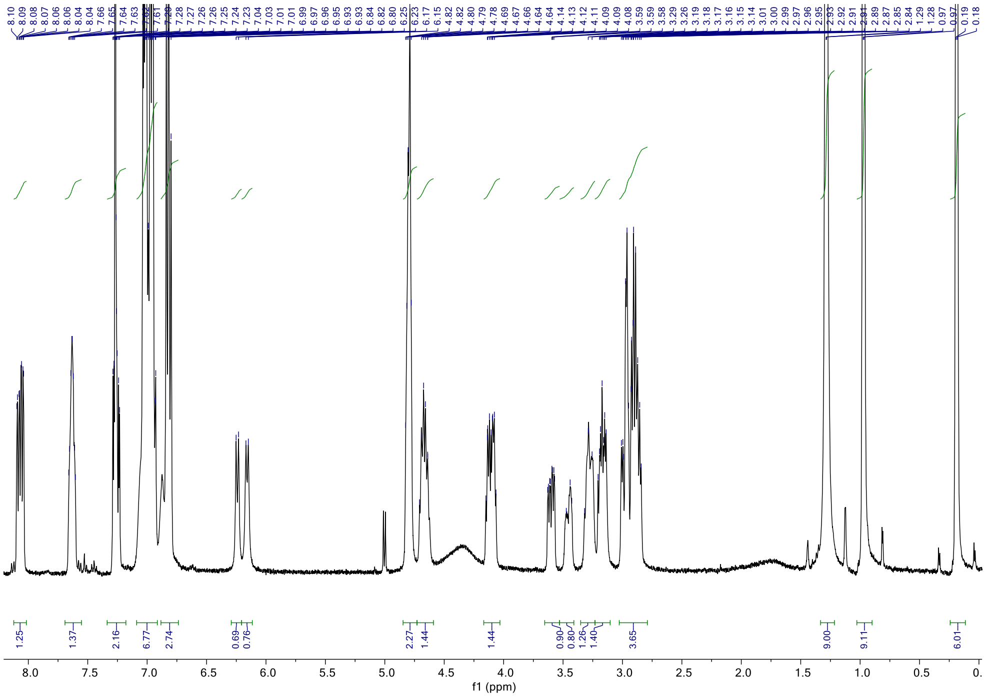

^{13}C NMR in $CDCl_3$

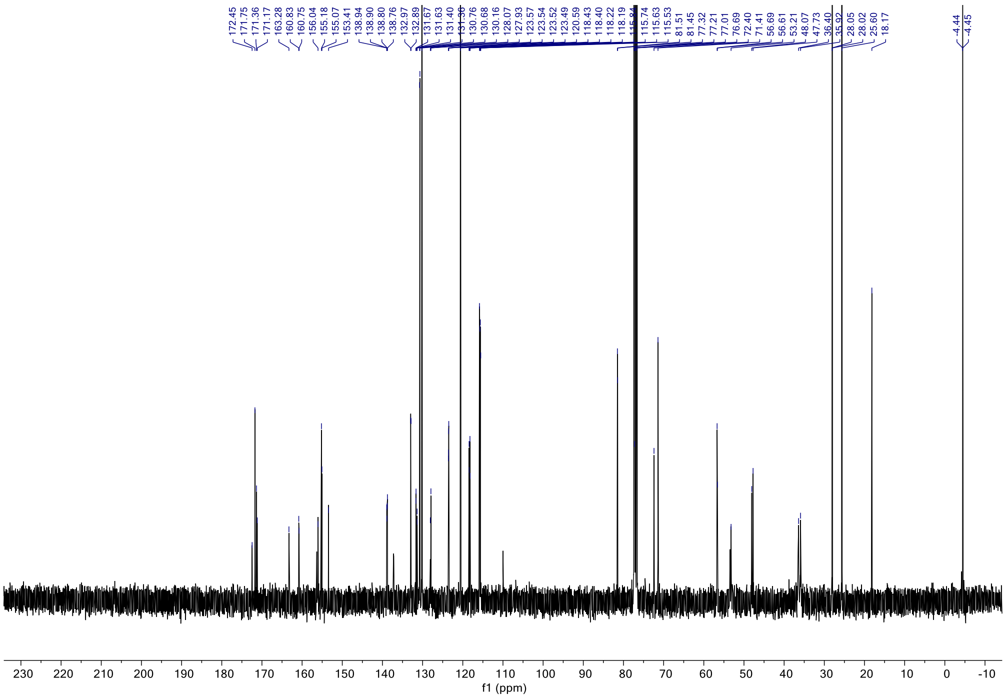

COSY in $CDCl_3$

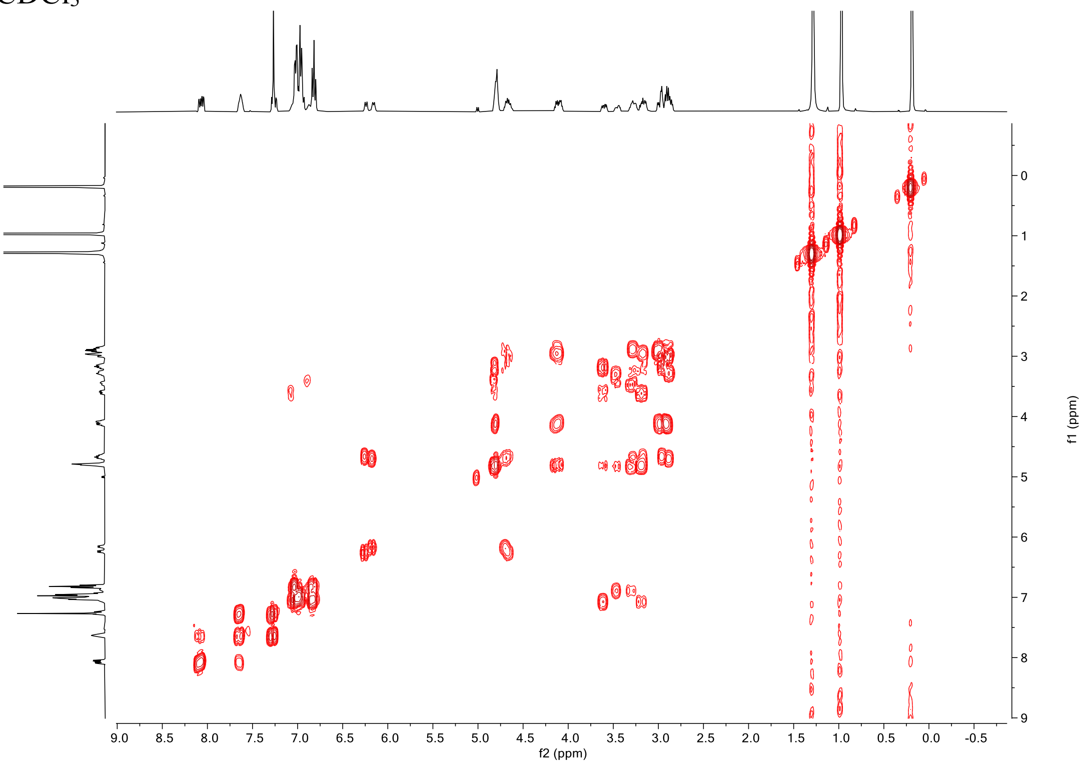

HSQC in $CDCl_3$

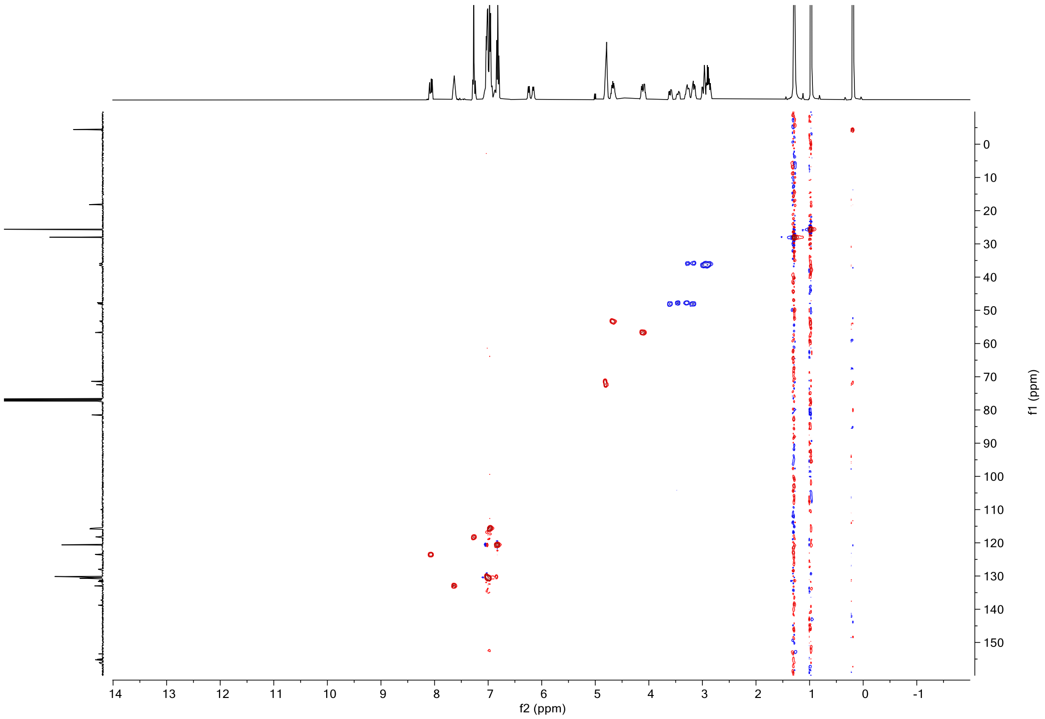

*tert-Butyl ((2S)-4-(4-((tert-butyldimethylsilyl)oxy)phenyl)-1-(((2S)-1-((2-(4-fluoro-3-nitrophenyl)-2-hydroxyethyl)amino)-1-oxo-3-phenylpropan-2-yl)amino)-1-oxobutan-2-yl)carbamate (**14c**)*.

^{1}H NMR in DMSO-d$_6$

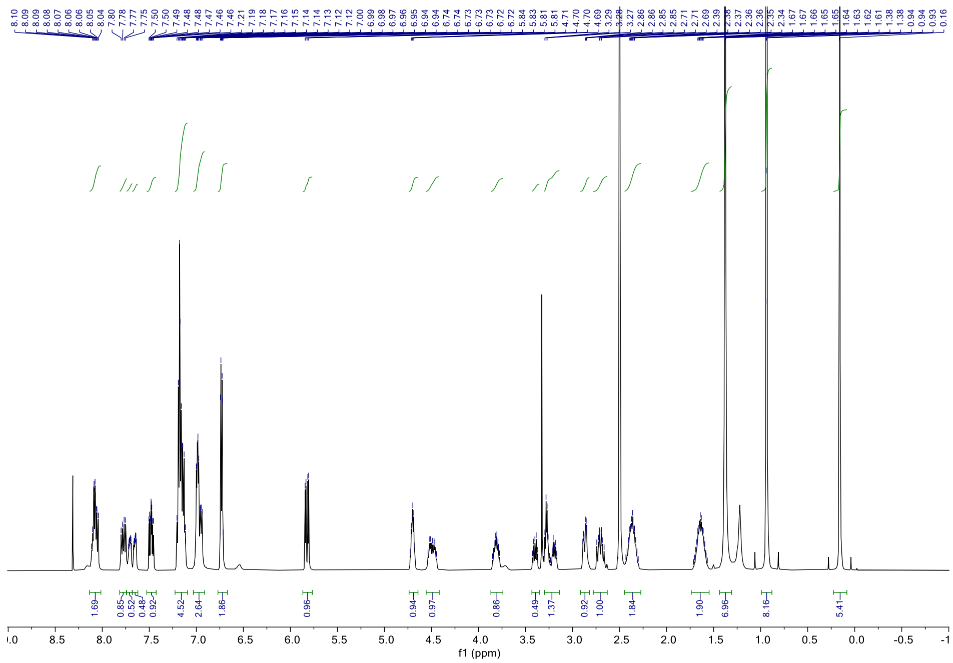

^{1}H NMR in DMSO-d$_6$ (expanded view)

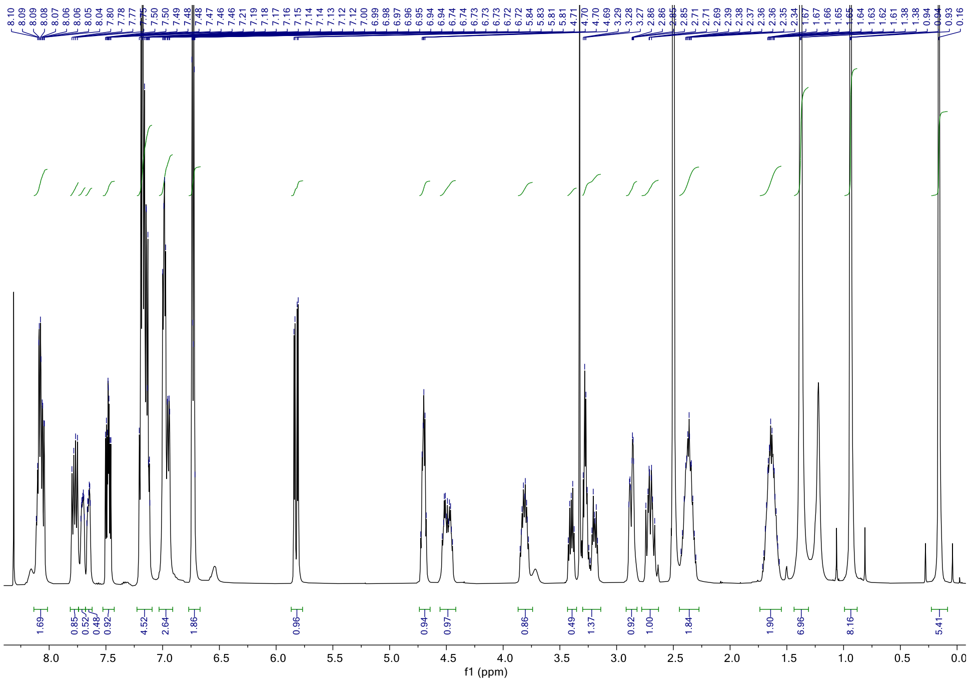

^{13}C NMR in DMSO-d$_6$

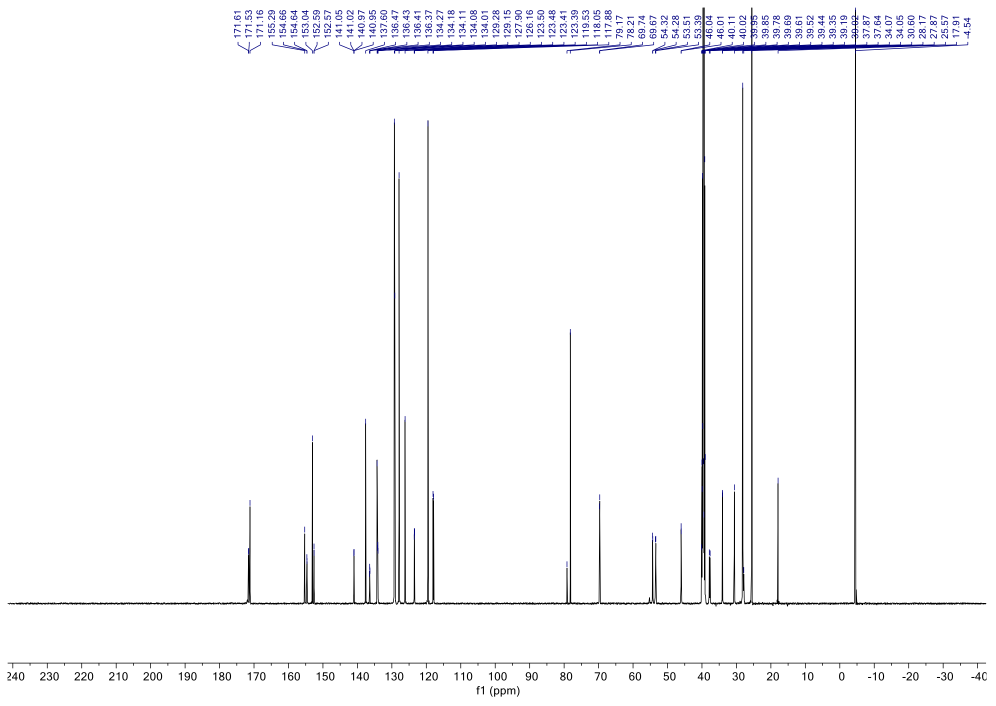

COSY in DMSO-d$_6$

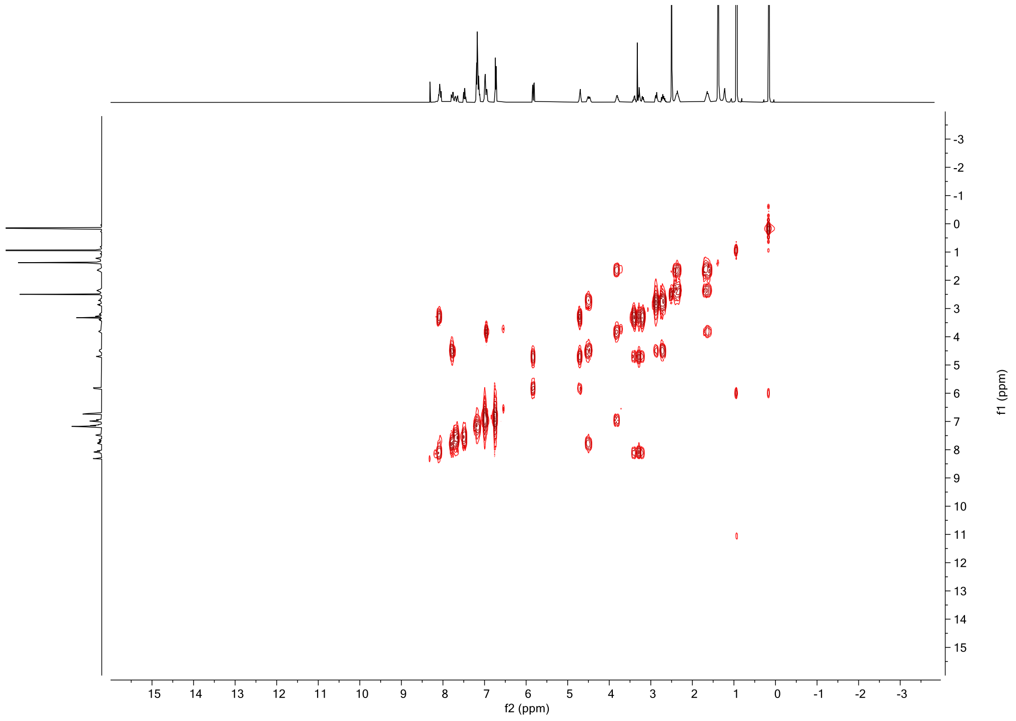

HSQC in DMSO-d_6

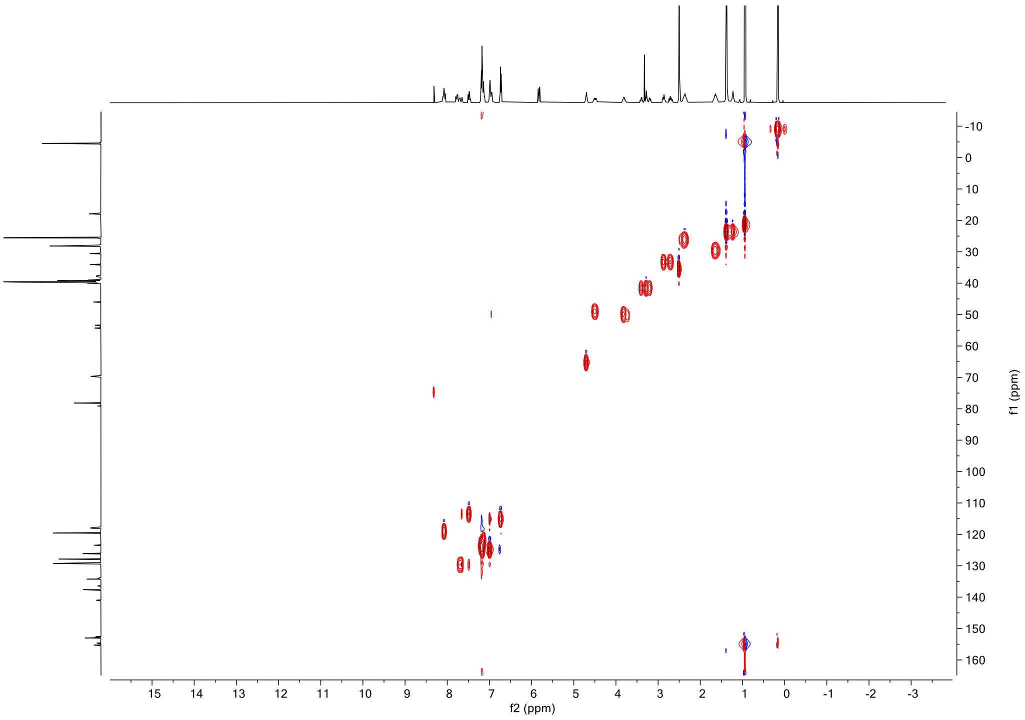

*tert-Butyl ((2S)-3-(4-((tert-butyldimethylsilyl)oxy)phenyl)-1-(((2S)-4-((2-(4-fluoro-3-nitrophenyl)-2-hydroxyethyl)amino)-3-oxo-1-phenylbutan-2-yl)amino)-1-oxopropan-2-yl)carbamate (**15c**).*

^{1}H NMR in DMSO-d_6

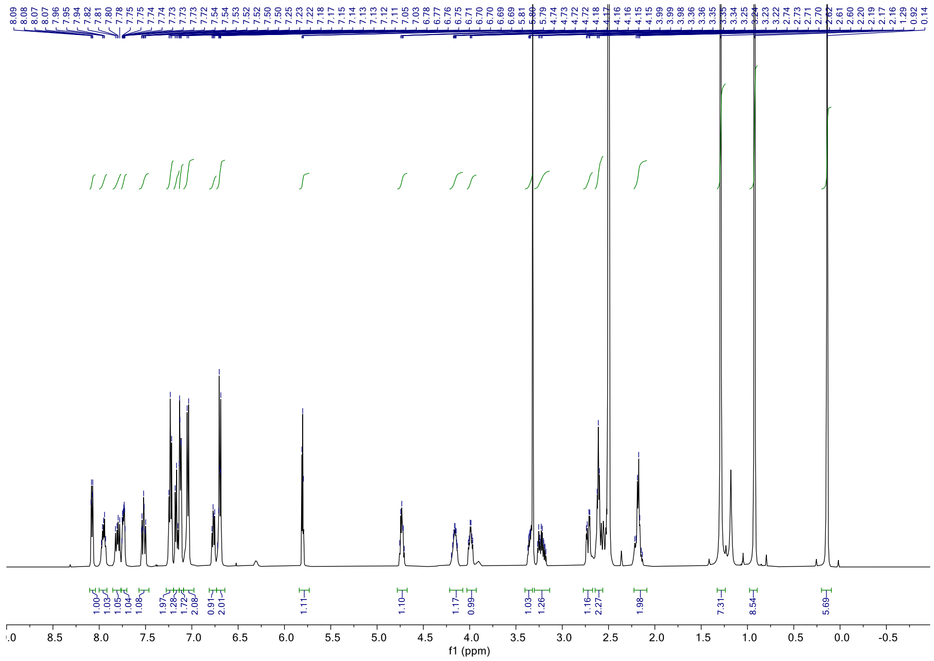

^{1}H NMR in DMSO-d_6 (expanded view)

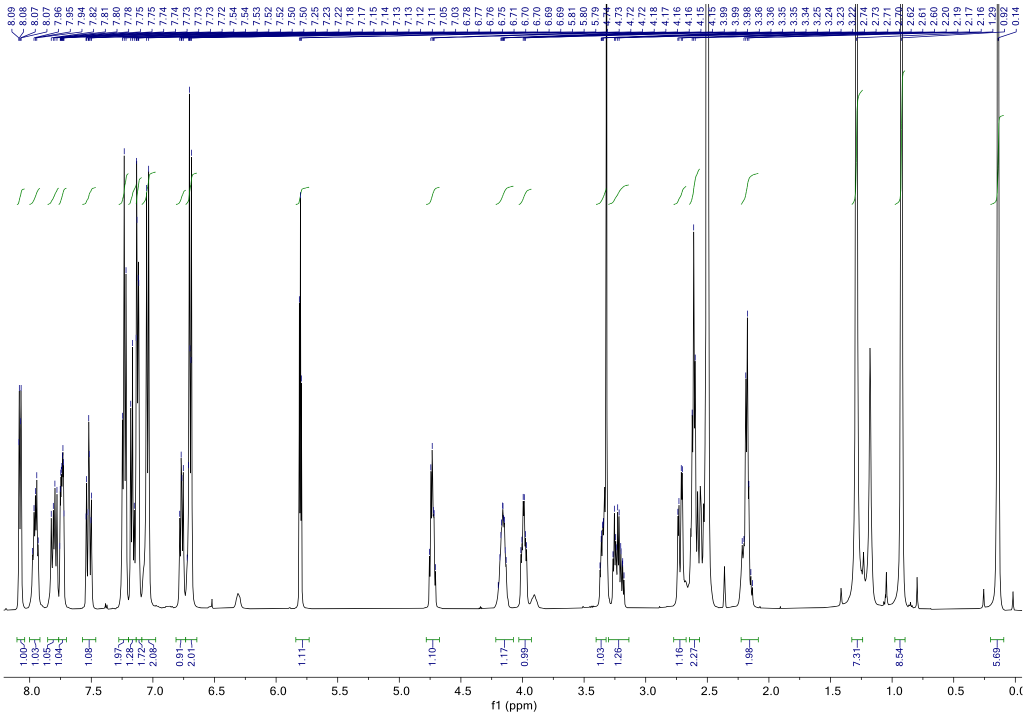

^{13}C NMR in DMSO-d_6

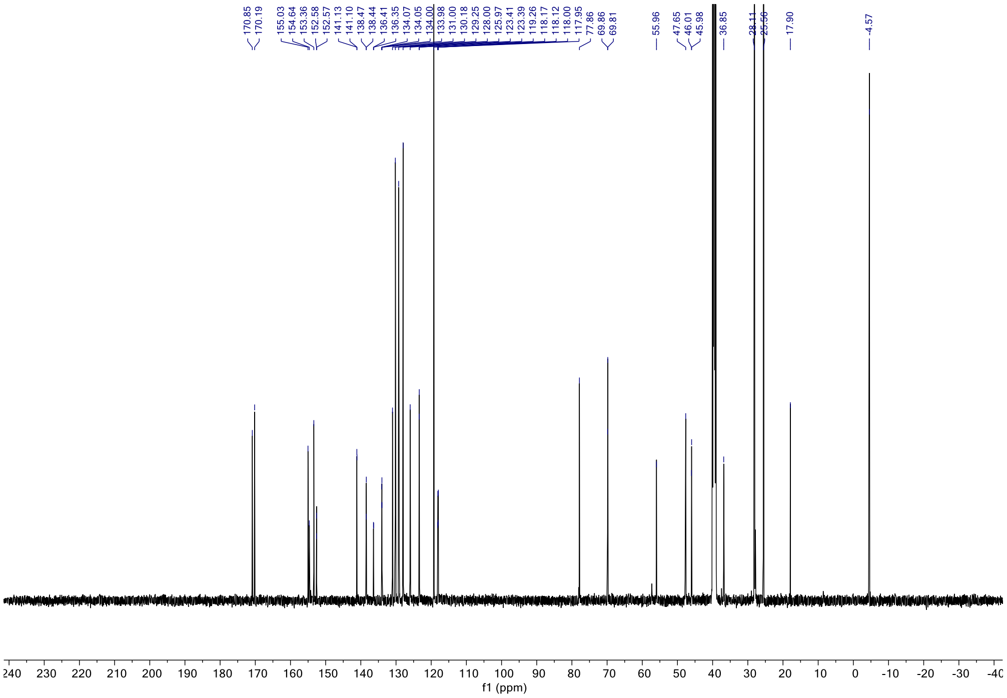

*tert-Butyl ((2S)-3-(4-((tert-butyldimethylsilyl)oxy)phenyl)-1-(((3S)-1-((2-(4-fluoro-3-nitrophenyl)-2-hydroxyethyl)amino)-5-methyl-2-oxohexan-3-yl)amino)-1-oxopropan-2-yl)carbamate (**15e**)*.

^{1}H NMR in DMSO-d$_6$

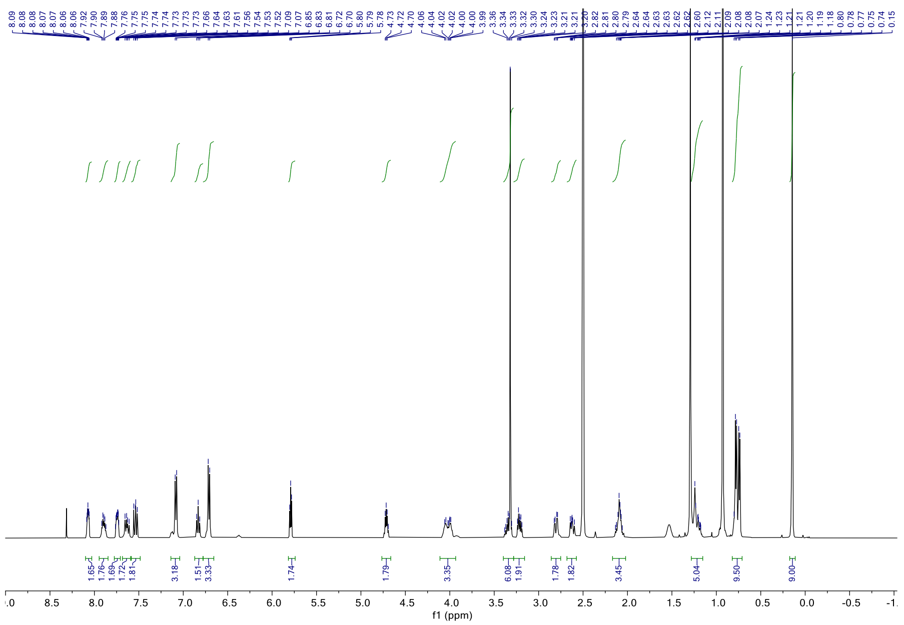

^{1}H NMR in DMSO-d$_6$ (expanded view)

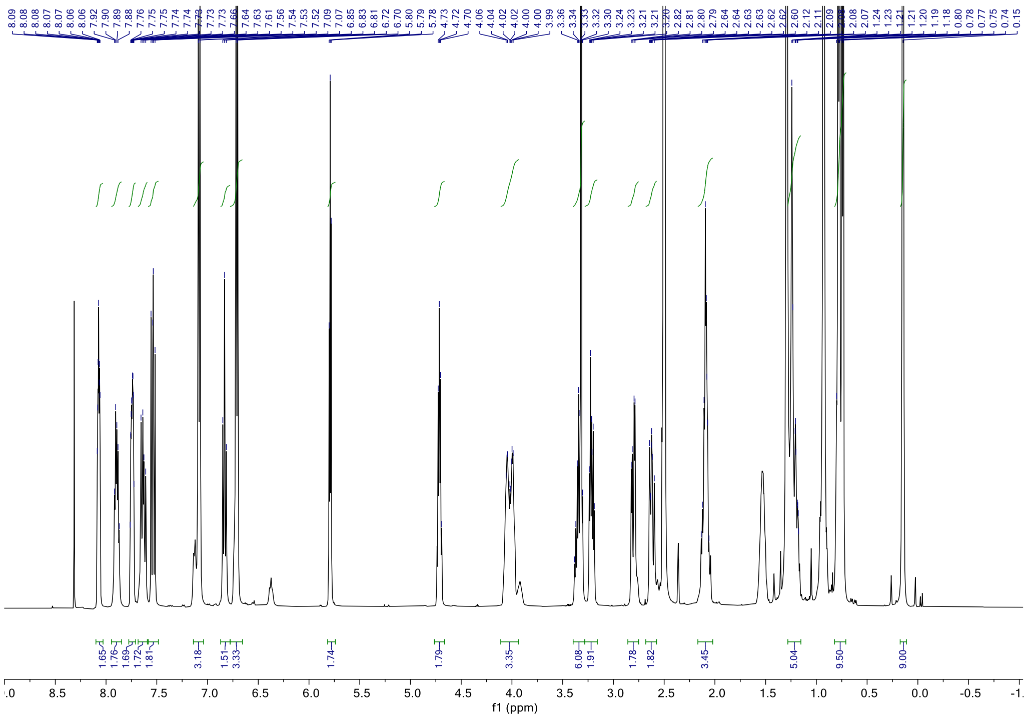

^{13}C NMR in DMSO-d$_6$

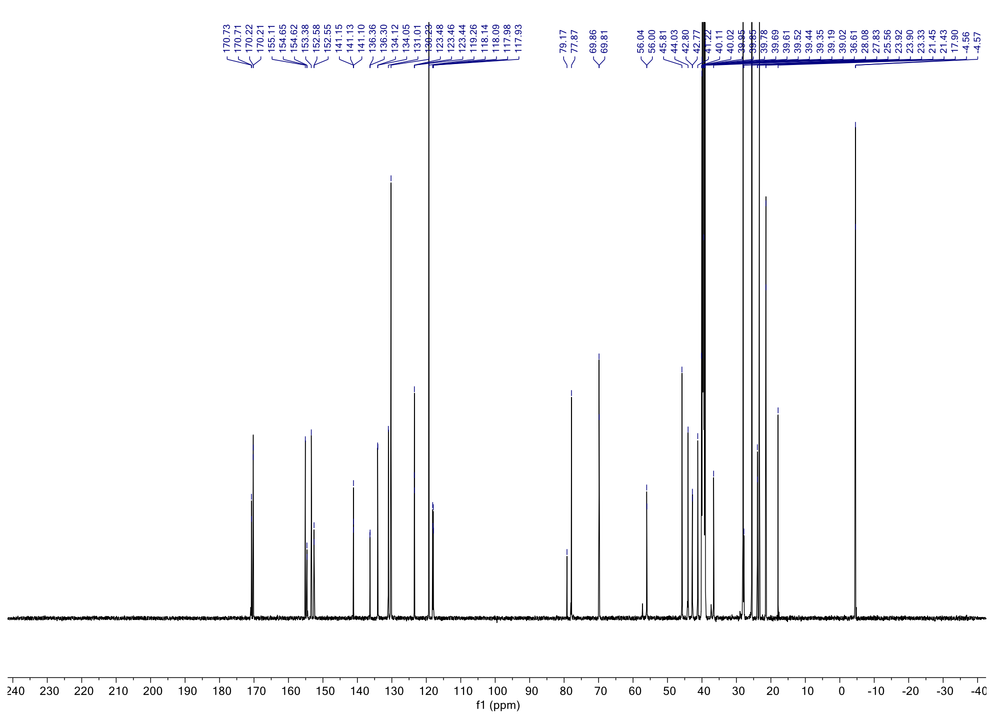

*tert-Butyl ((8S,11S)-4-hydroxy-8-isobutyl-32-nitro-7,10-dioxo-2-oxa-6,9-diaza-1,3(1,4)-dibenzenacyclododecaphane-11-yl)carbamate (**16e**)*.

^{1}H NMR in DMSO-d$_6$

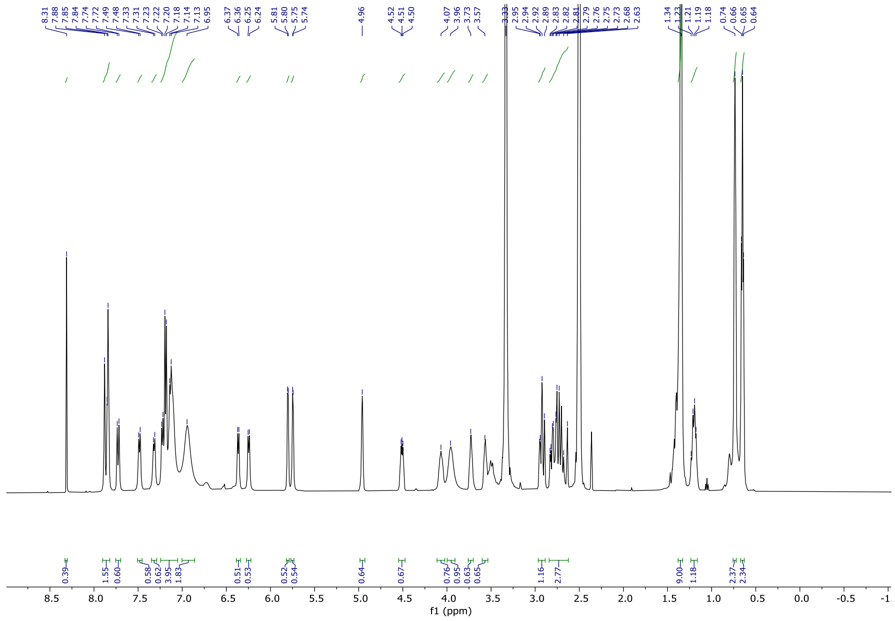

^{1}H NMR in DMSO-d_6 (expanded view)

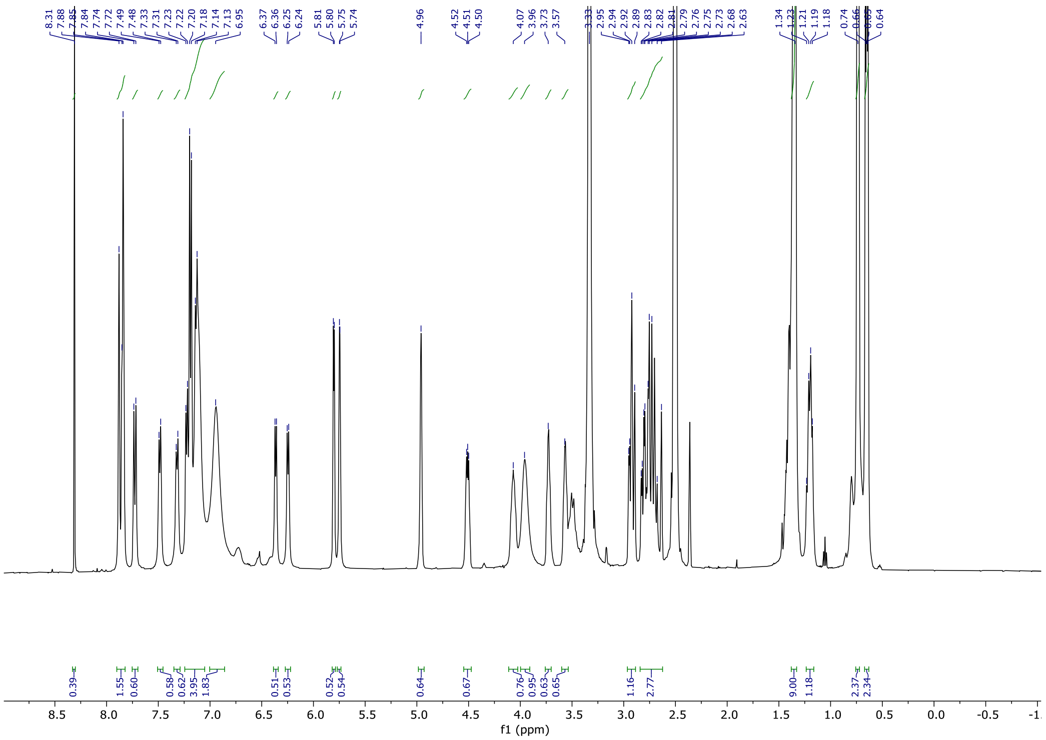

^{13}C NMR in DMSO-d_6

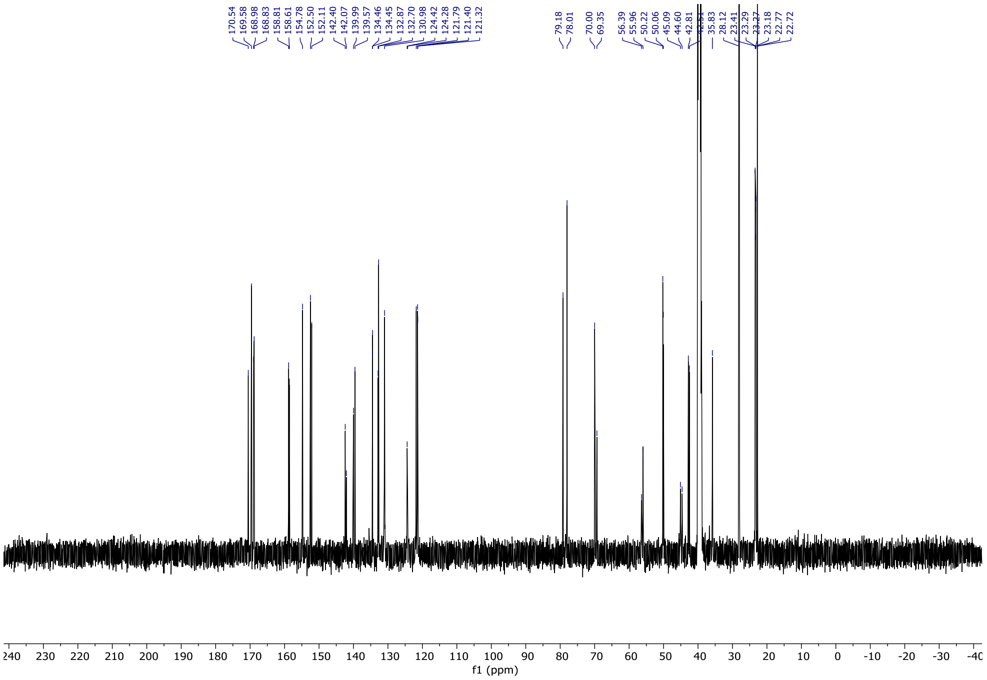

COSY in DMSO-d_6

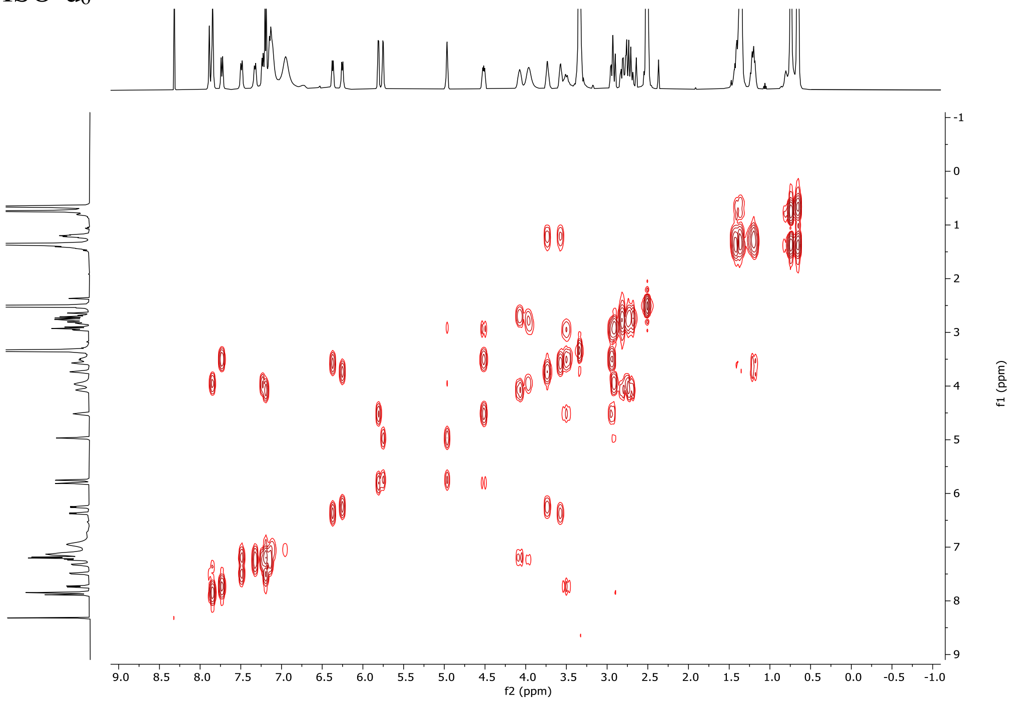

HSQC in DMSO-d_6

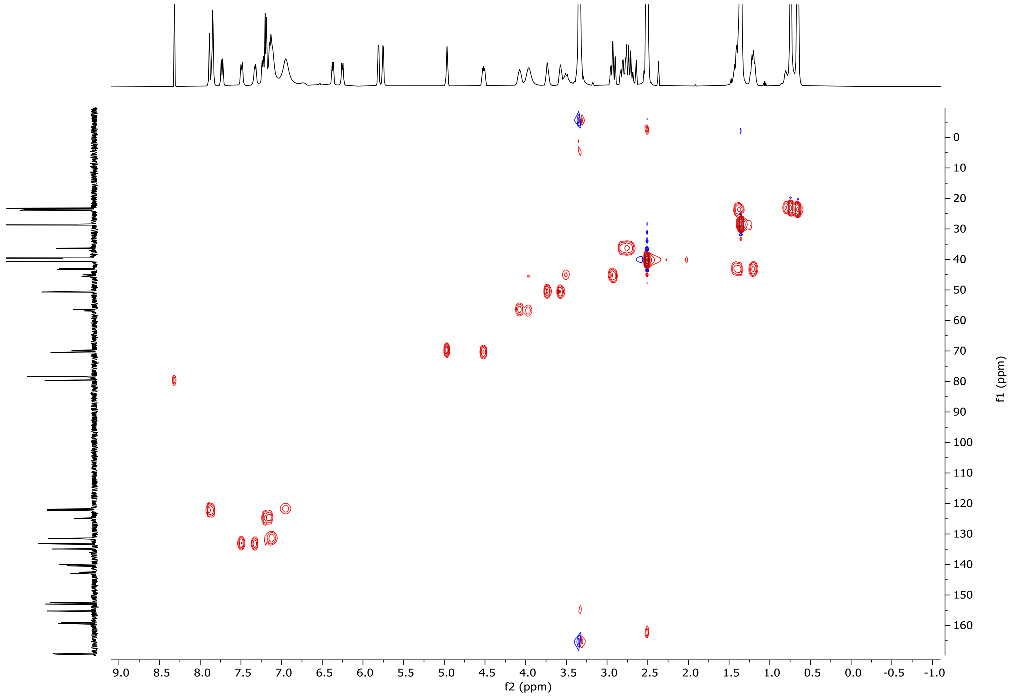

*tert-Butyl ((8S,11S)-4-hydroxy-8-(4-methylbenzyl)-32-nitro-7,10-dioxo-2-oxa-6,9-diaza-1,3(1,4)-dibenzenacyclododecaphane-11-yl)carbamate (**16h**)*.

^{1}H NMR in DMSO-d_6

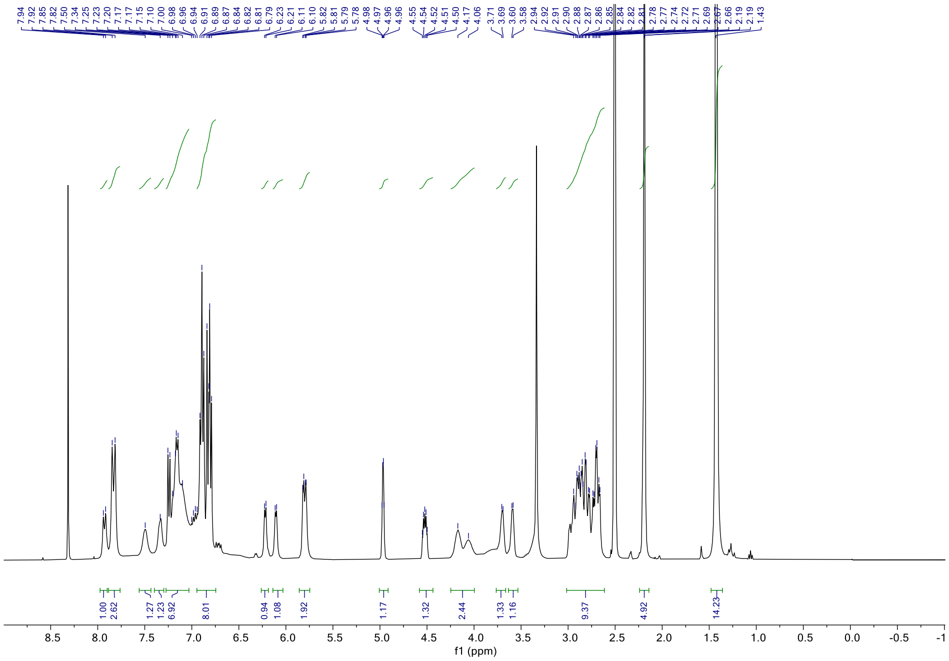

^{1}H NMR in DMSO-d_6 (expanded view)

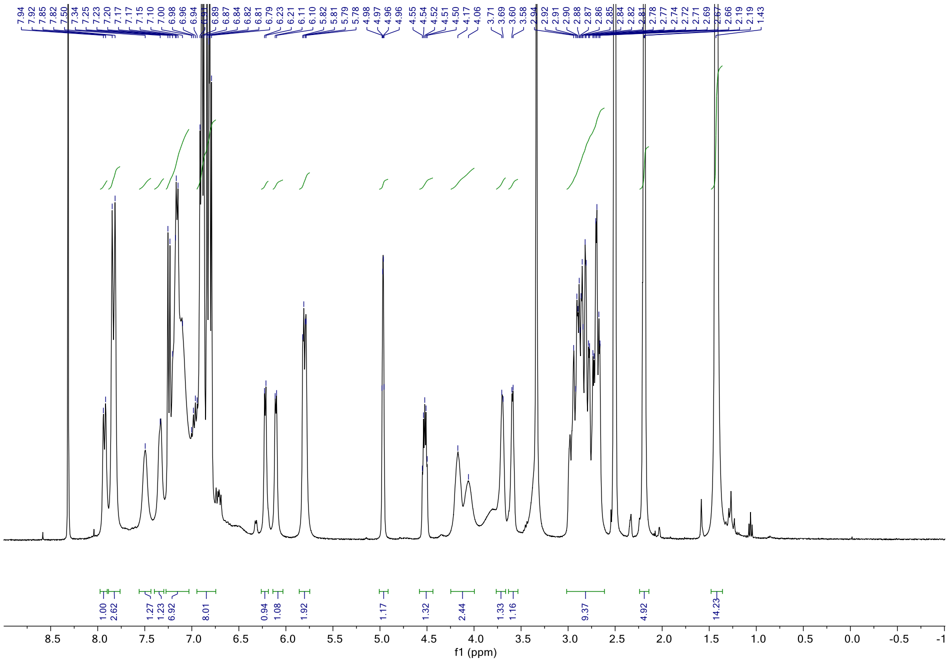

^{13}C NMR in DMSO-d$_6$

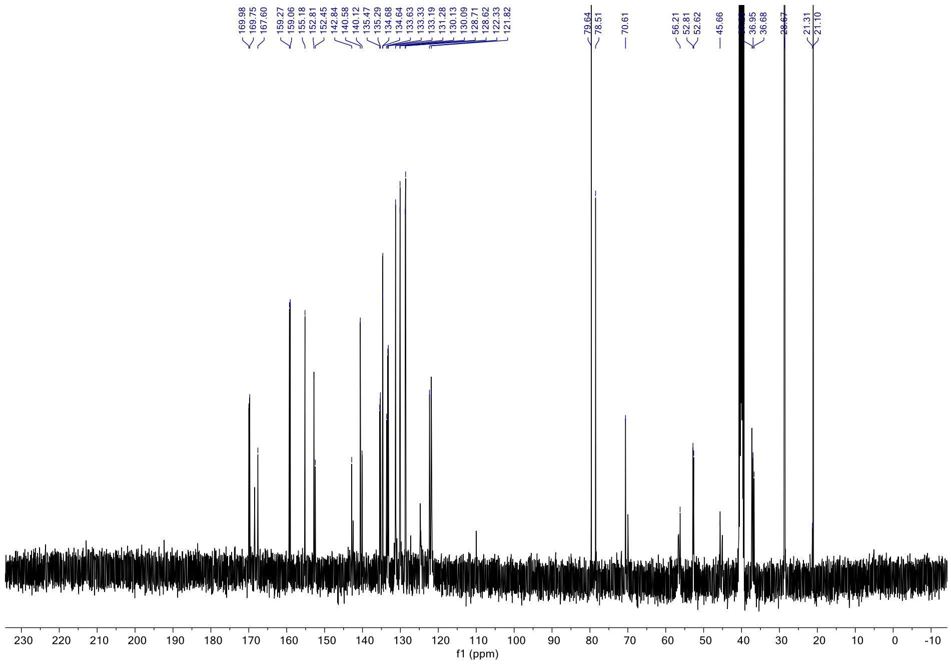

COSY in DMSO-d$_6$

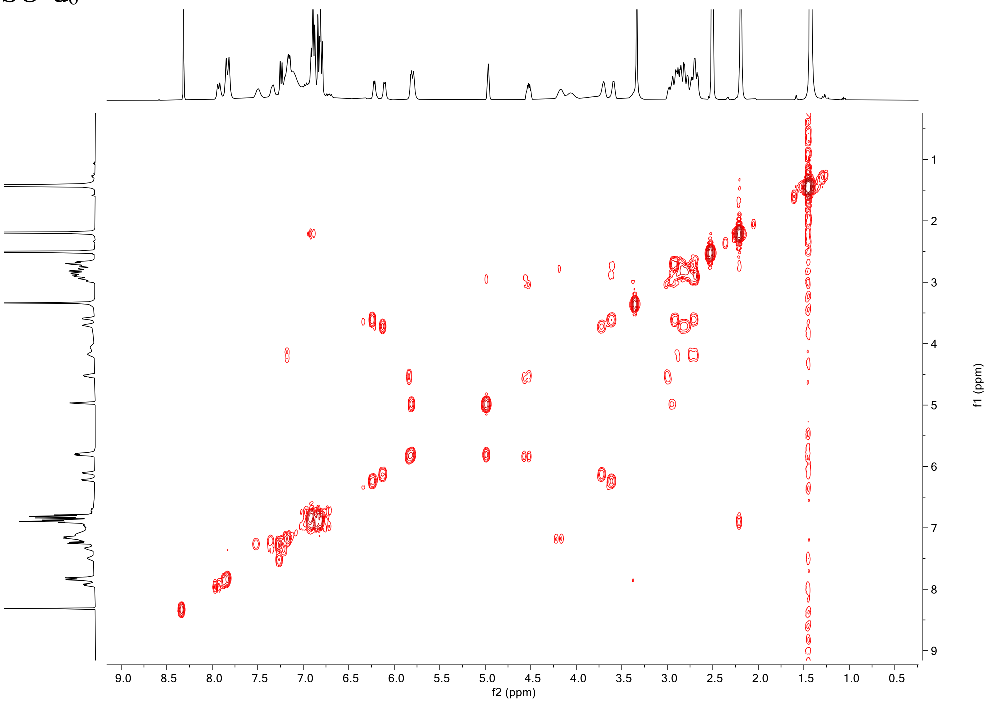

HSQC in DMSO-d_6

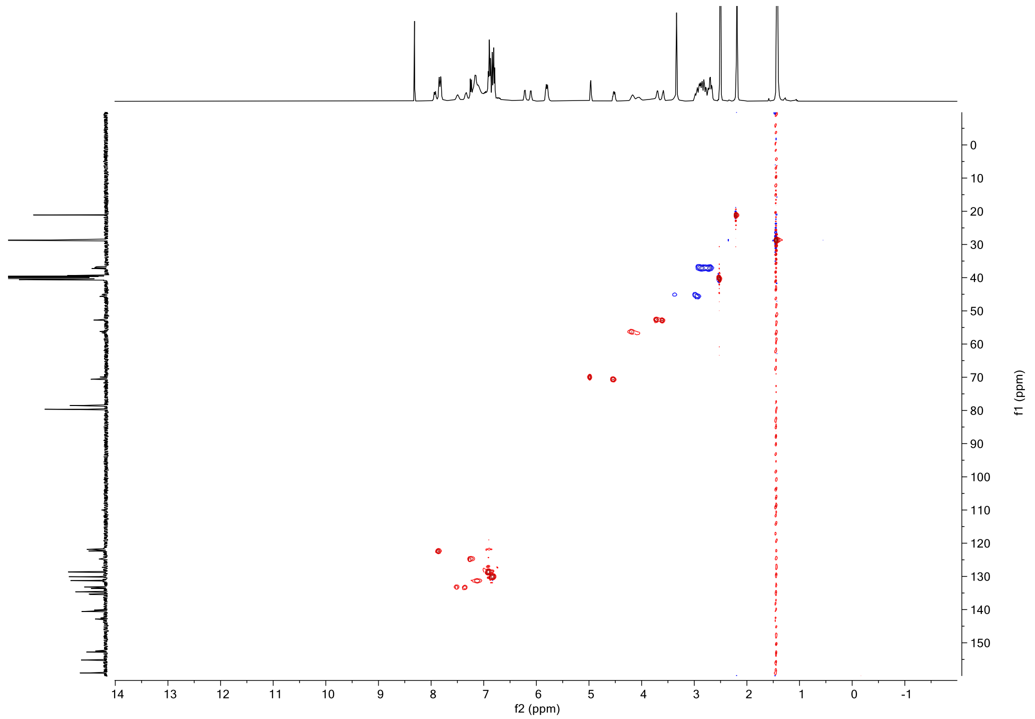

tert-Butyl ((8S,11S)-8-(4-fluorobenzyl)-4-hydroxy-32-nitro-7,10-dioxo-2-oxa-6,9-diaza-1,3(1,4)-dibenzenacyclododecaphane-11-yl)carbamate ***(16i)***.

^{1}H NMR in DMSO-d_6

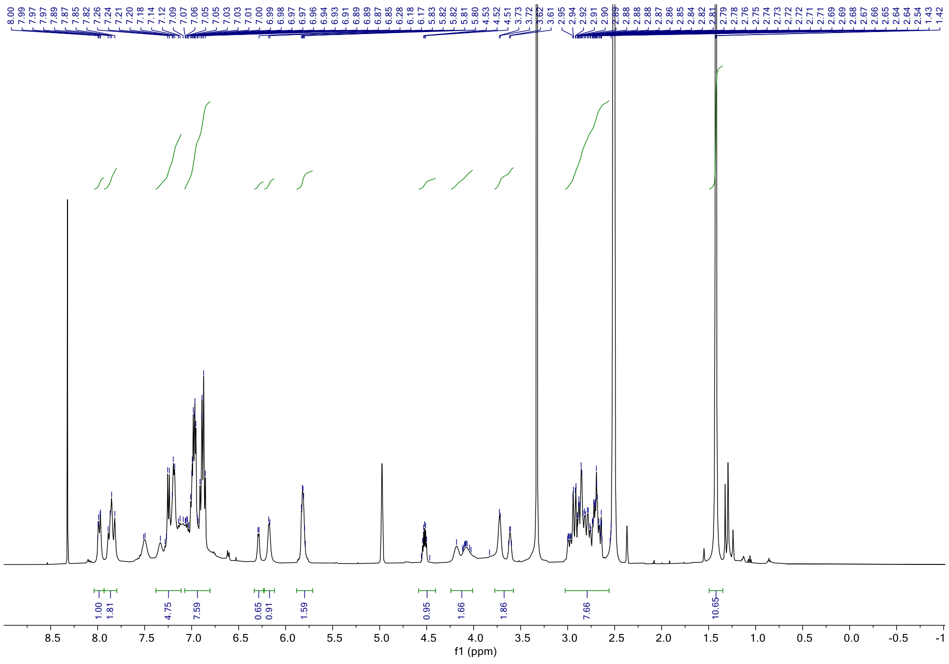

^{1}H NMR in DMSO-d$_6$ (expanded view)

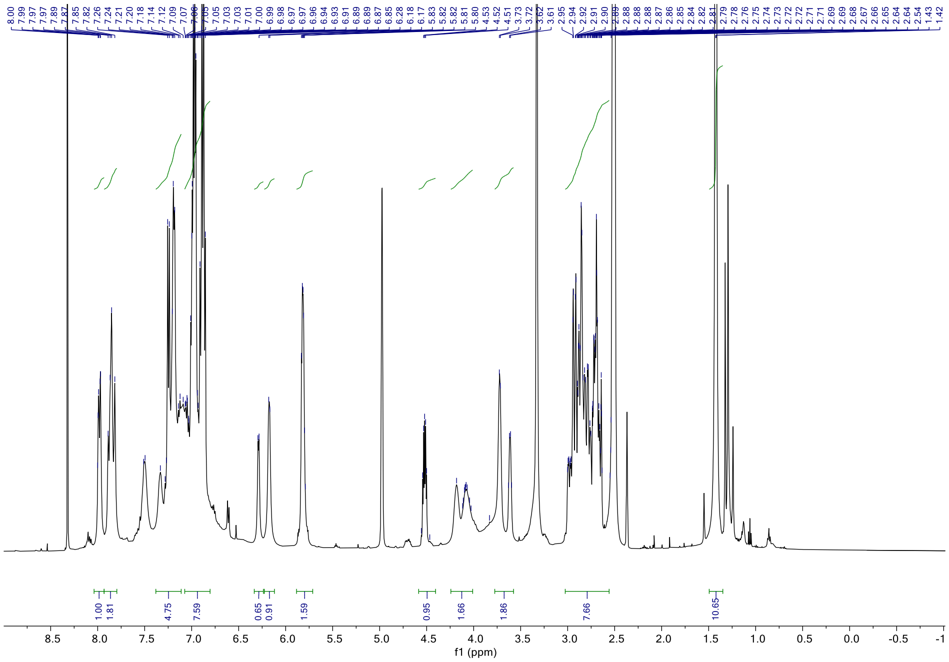

^{13}C NMR in DMSO-d$_6$

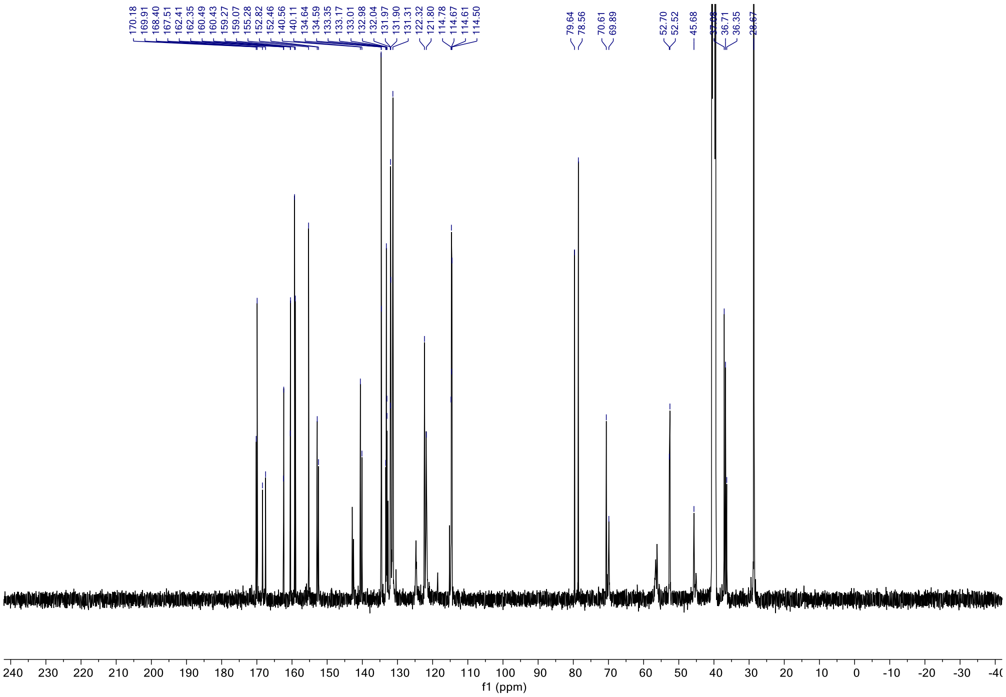

COSY in DMSO-d_6

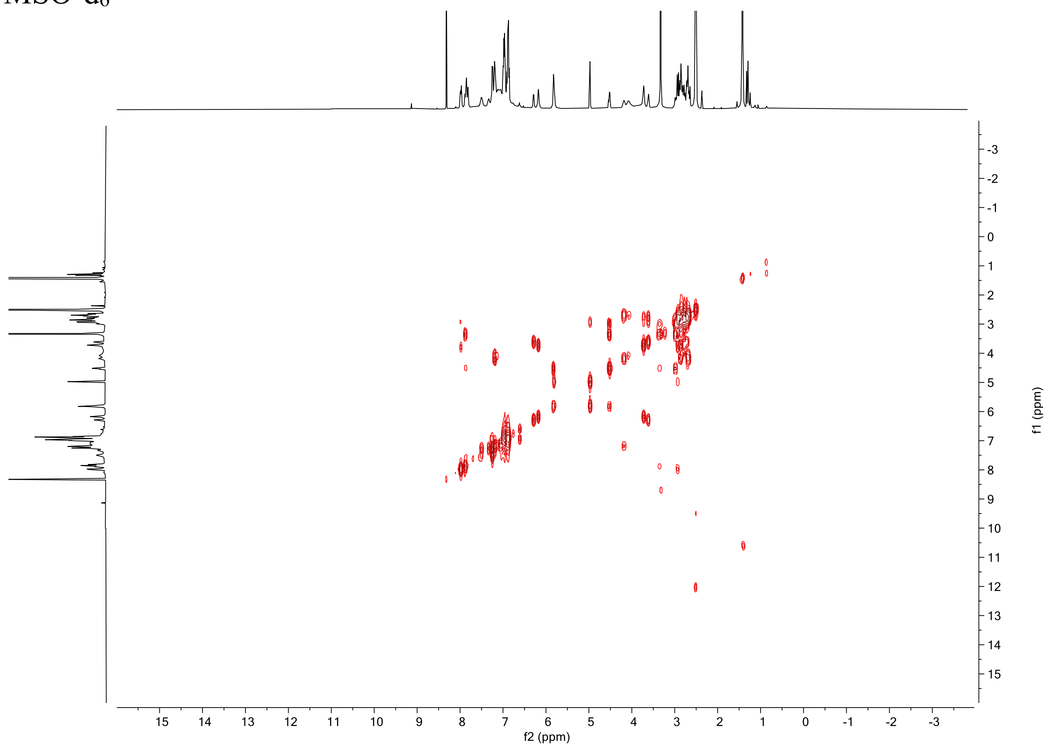

HSQC in DMSO-d_6

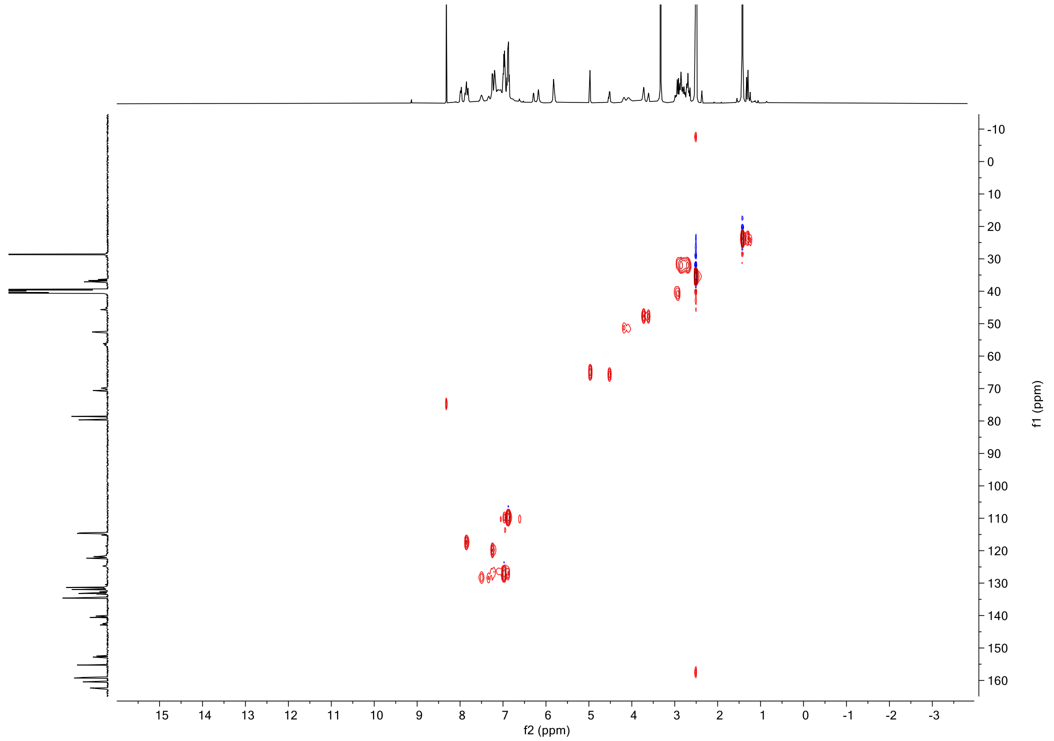

*tert-Butyl ((8S,11S)-8-benzyl-4-hydroxy-32-nitro-7,10-dioxo-2-oxa-6,9-diaza-1,3(1,4)-dibenzenacyclotridecaphane-11-yl)carbamate (**17c**)*.

^{1}H NMR in DMSO-d$_6$

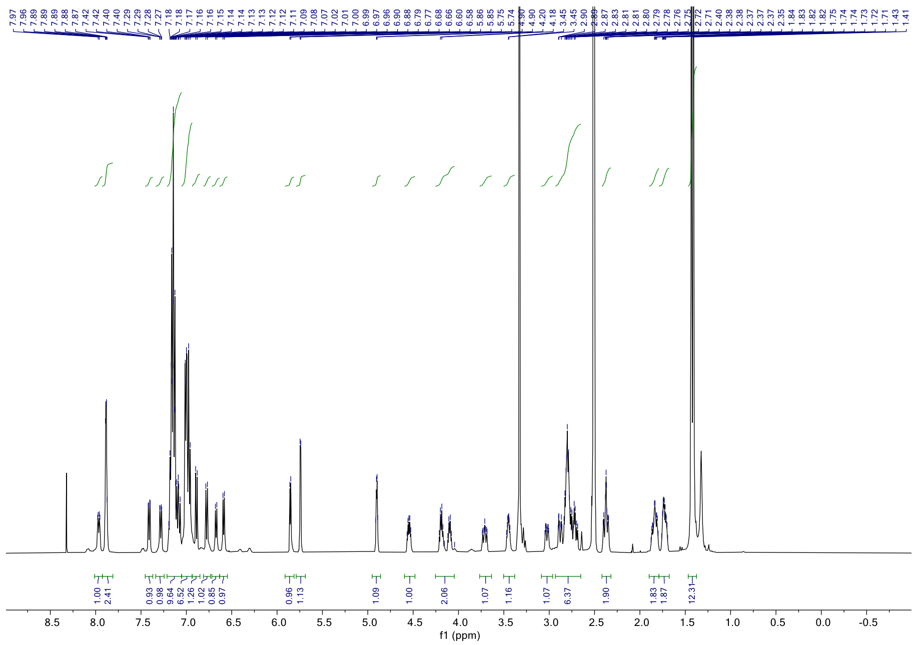

^{1}H NMR in DMSO-d$_6$ (expanded view)

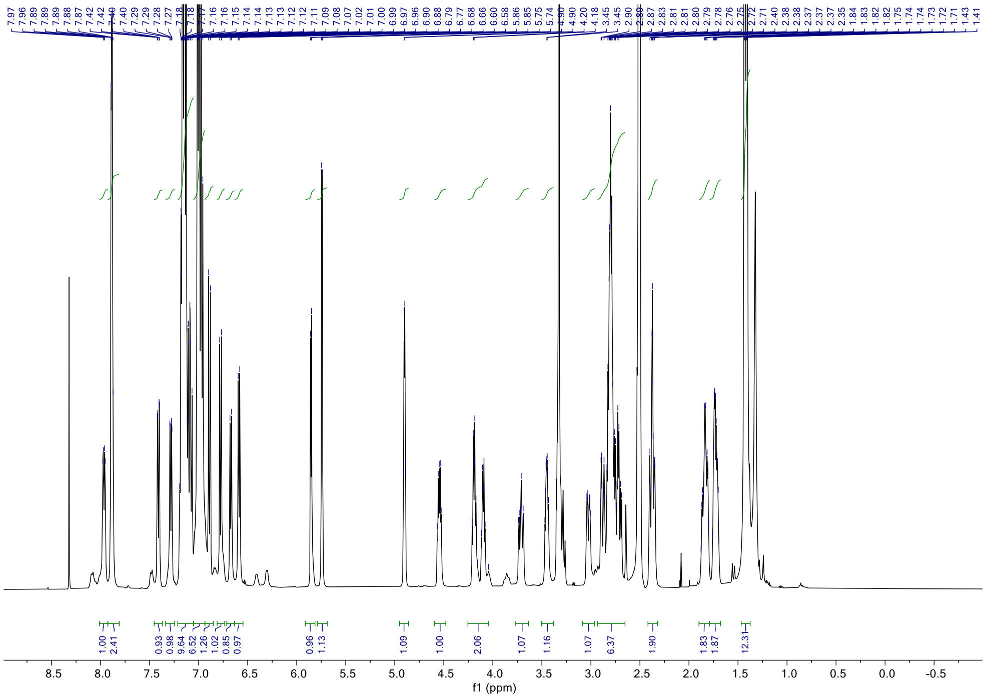

^{13}C NMR in DMSO-d$_6$

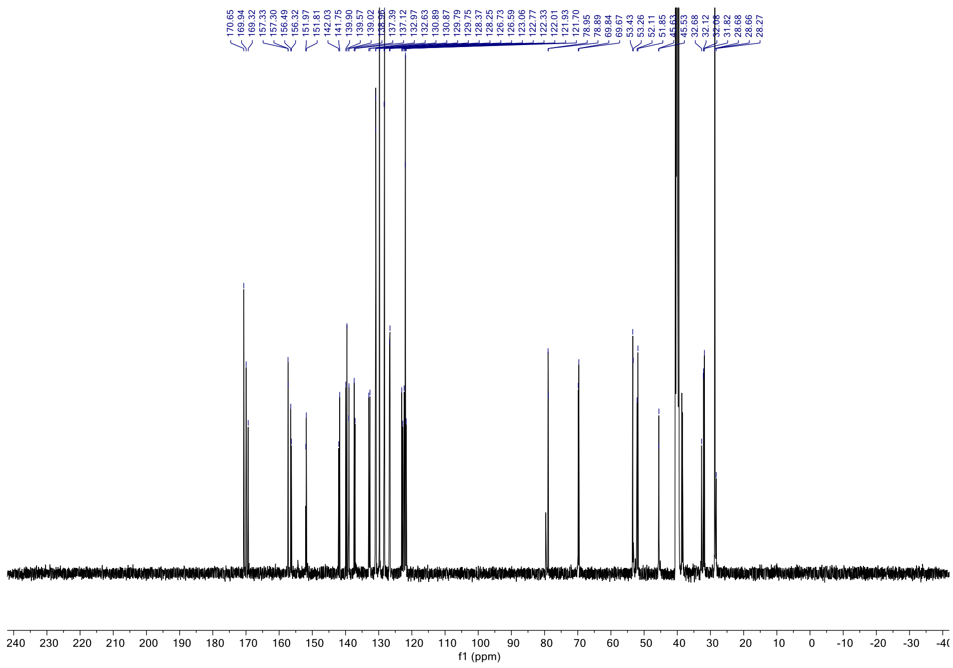

*tert-Butyl ((9S,12S)-9-benzyl-4-hydroxy-32-nitro-7,11-dioxo-2-oxa-6,10-diaza-1,3(1,4)-dibenzenacyclotridecaphane-12-yl)carbamate **(18c)**.*

^{1}H NMR in DMSO-d$_6$

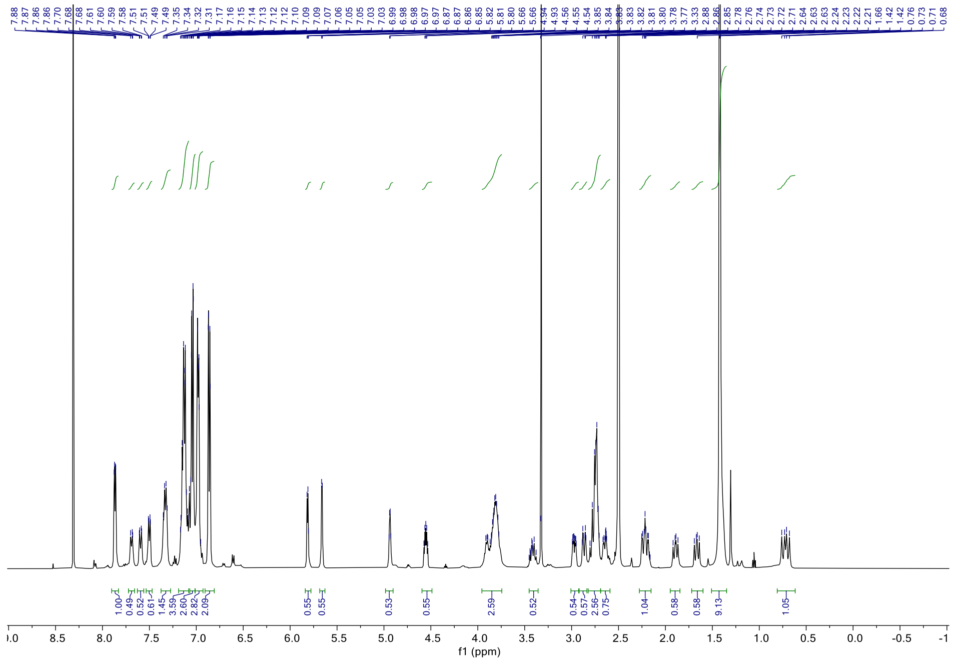

^{1}H NMR in DMSO-d_6 (expanded view)

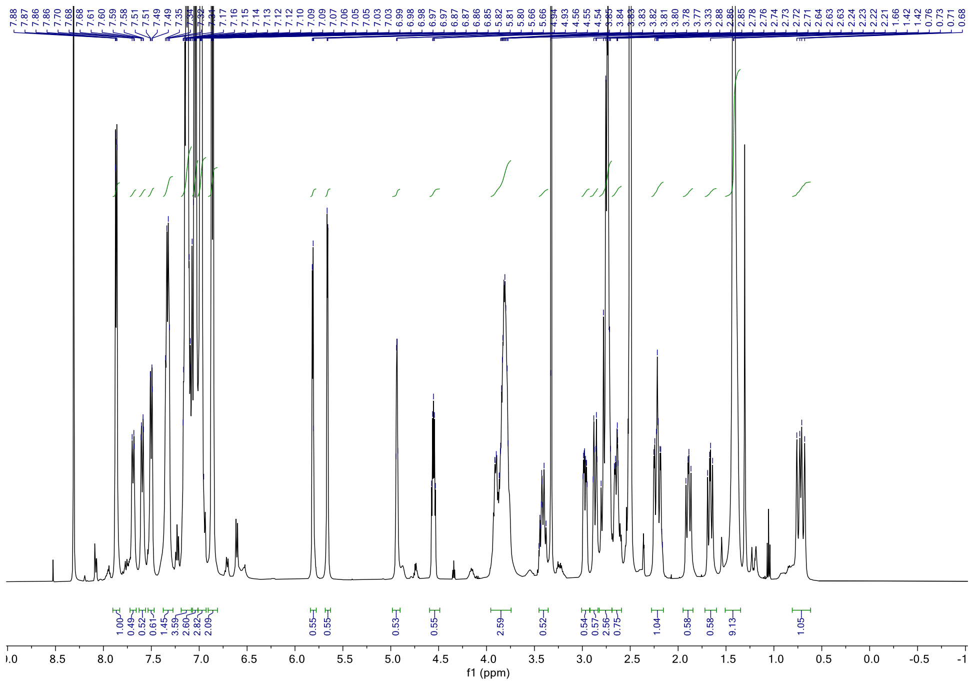

^{13}C NMR in DMSO-d_6

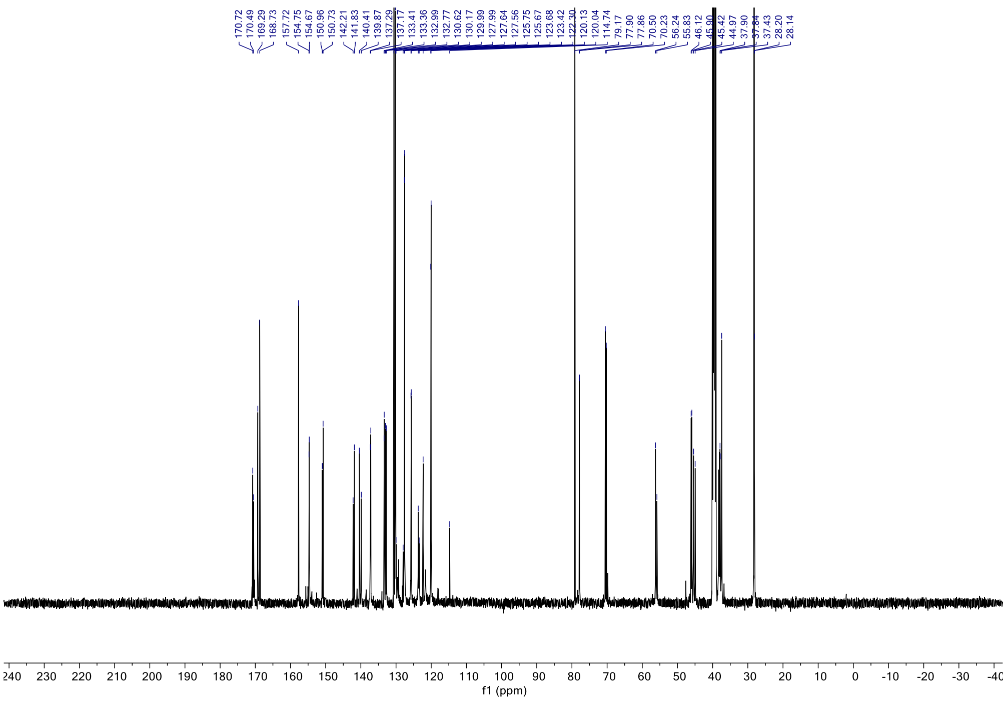

*tert-Butyl ((9S,12S)-4-hydroxy-9-isobutyl-32-nitro-7,11-dioxo-2-oxa-6,10-diaza-1,3(1,4)-dibenzenacyclotridecaphane-12-yl)carbamate **(18e)**.*

^{1}H NMR in DMSO-d_6

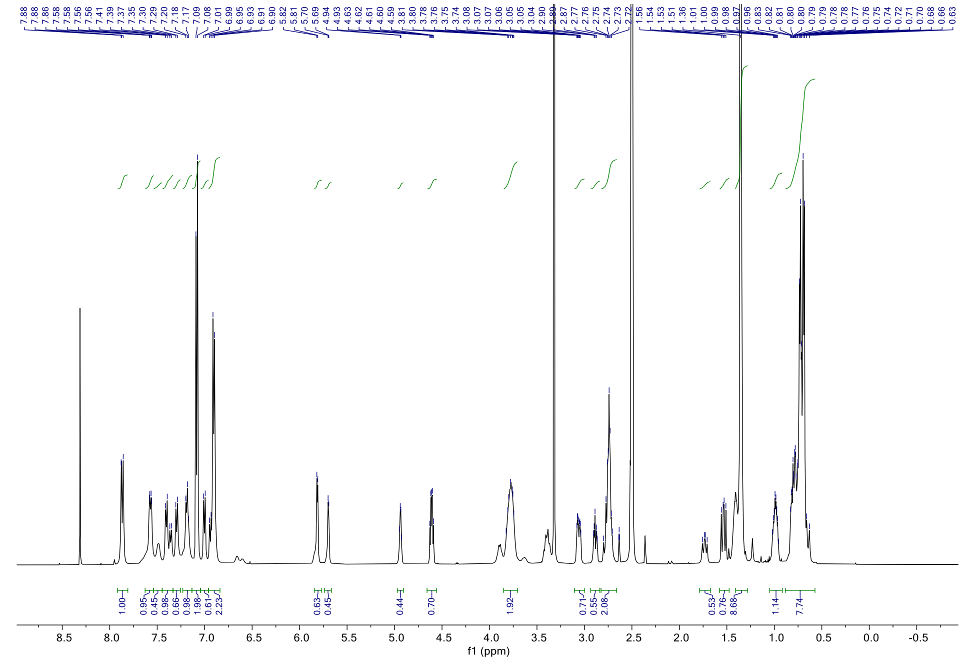

^{1}H NMR in DMSO-d_6 (expanded view)

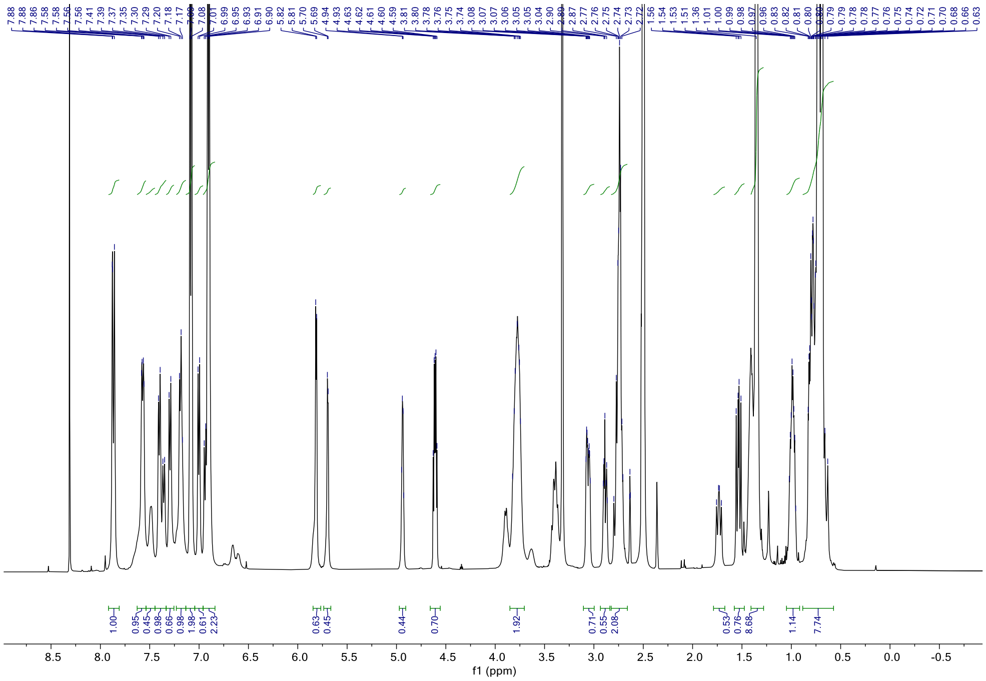

^{13}C NMR in DMSO-d$_6$

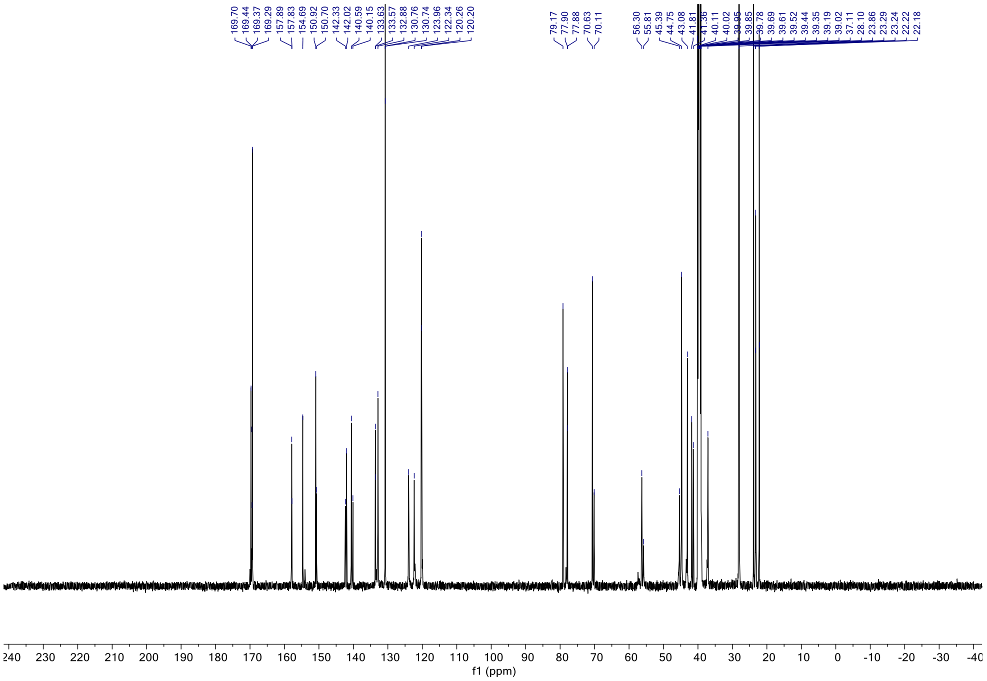

*tert-Butyl ((8S,11S)-8-isobutyl-4,7,10-trioxo-2-oxa-6,9-diaza-1,3(1,4)-dibenzenacyclododecaphane-11-yl)carbamate (**19e**).*

^{1}H NMR in $CDCl_3$

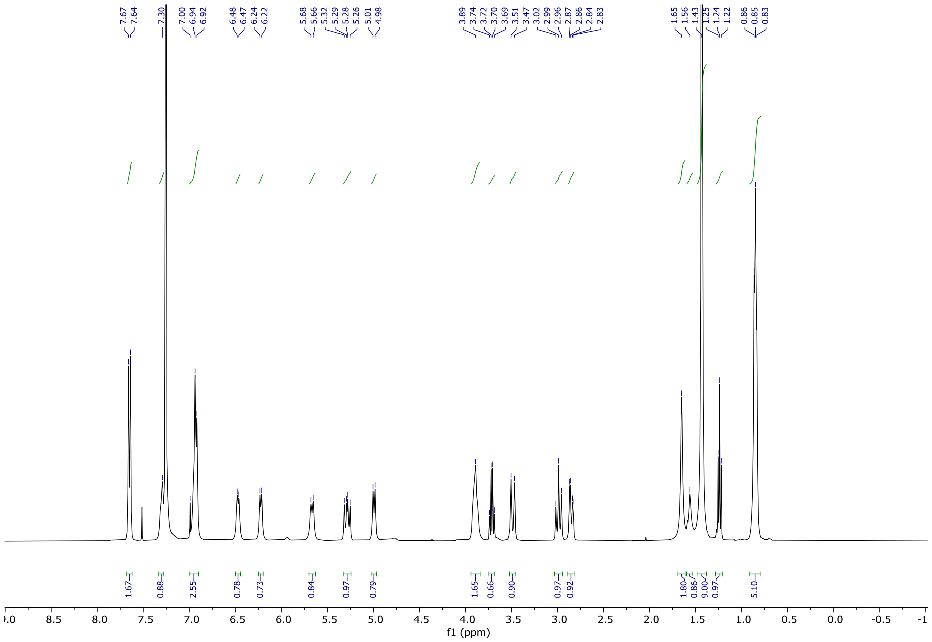

^{1}H NMR in $CDCl_3$ (expanded view)

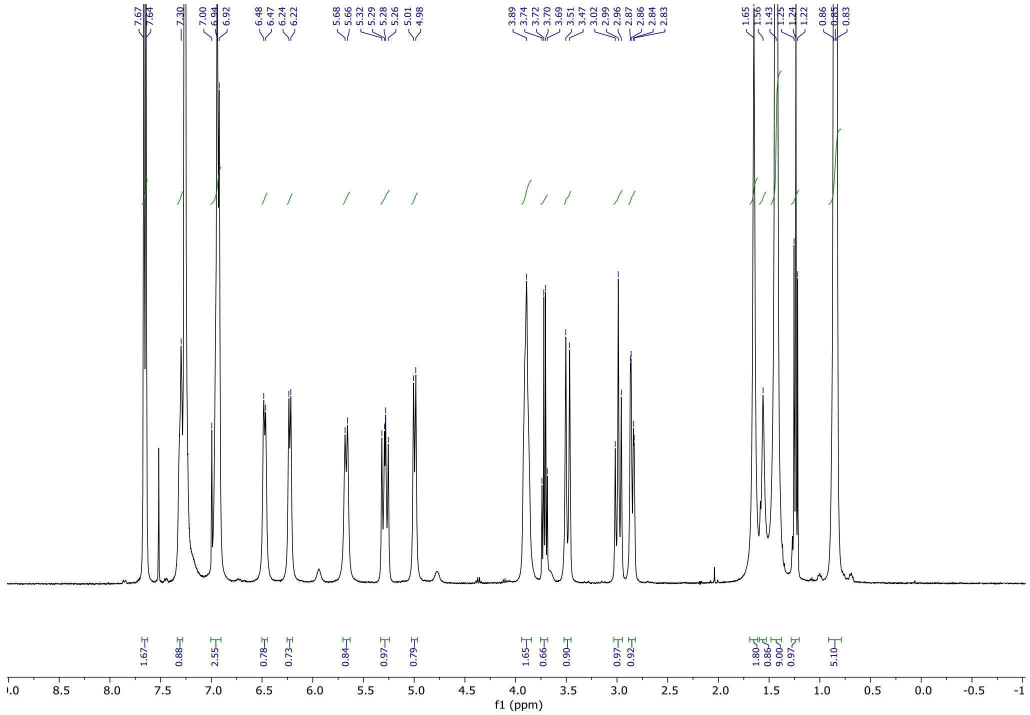

^{13}C NMR in $CDCl_3$

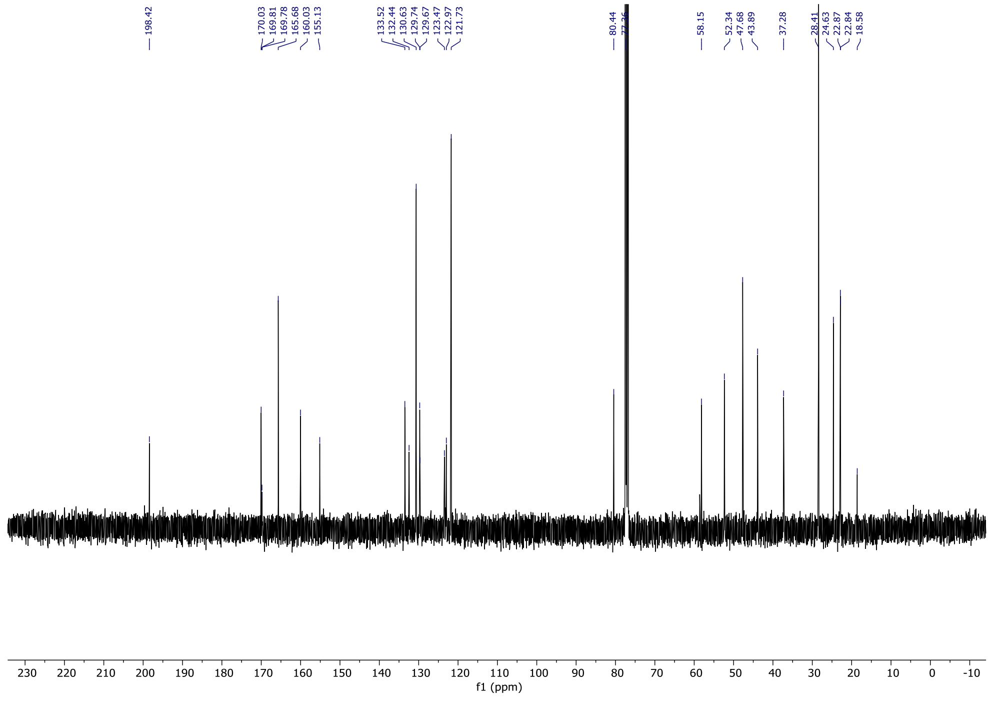

COSY in $CDCl_3$

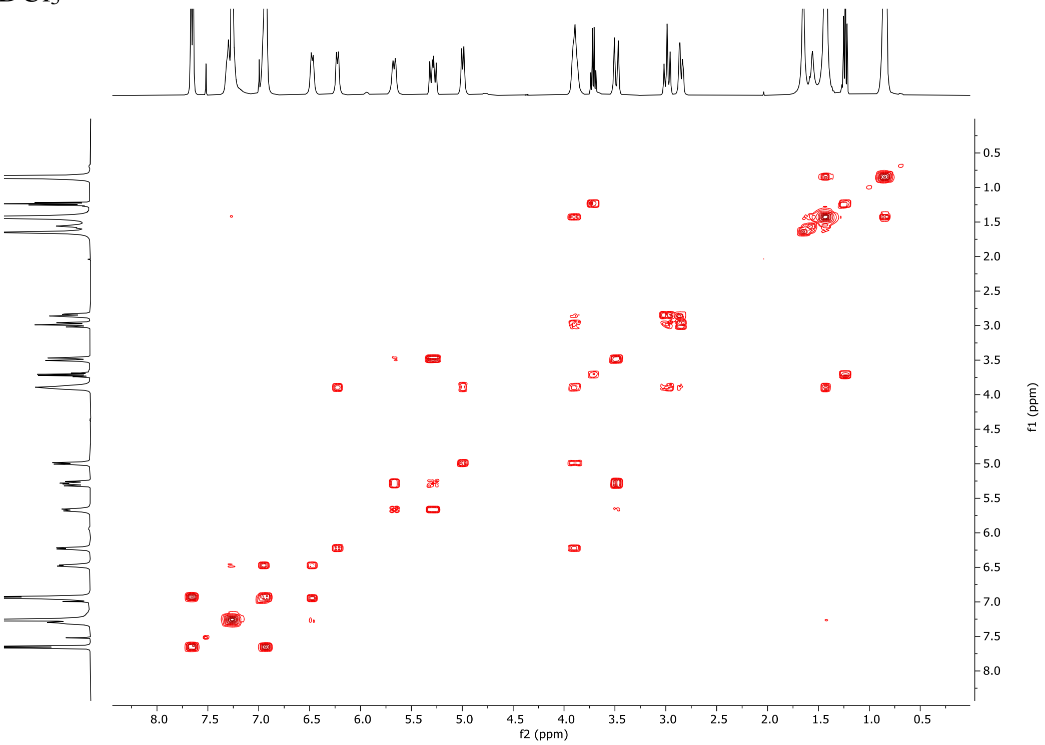

HSQC in $CDCl_3$

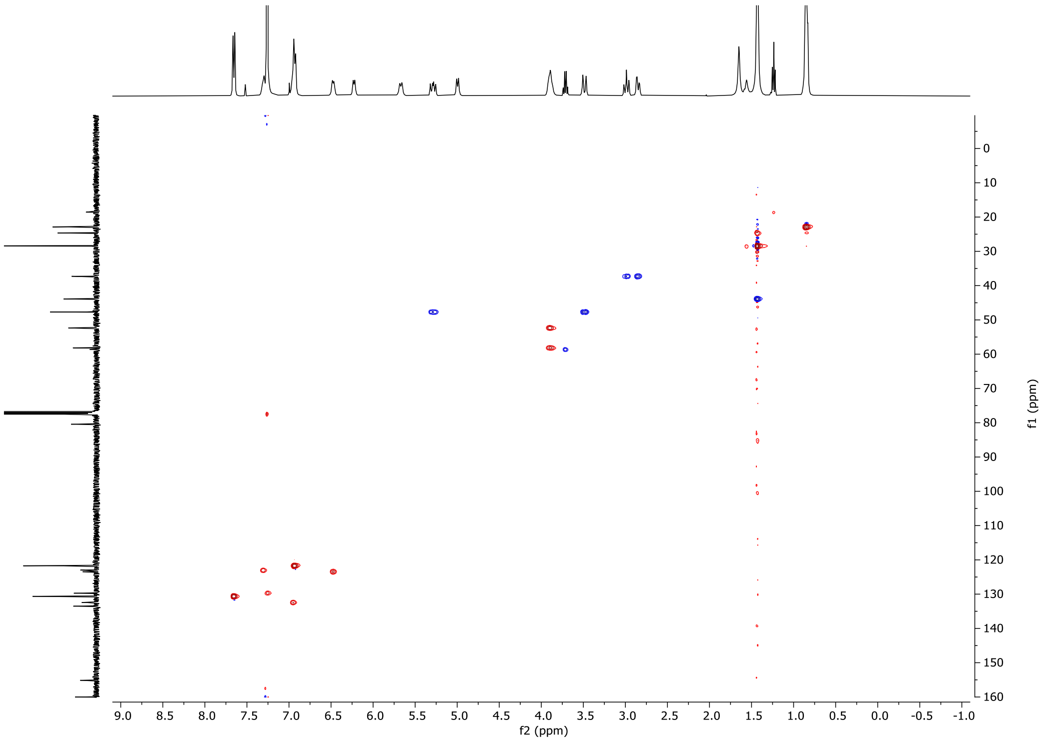

*tert-Butyl ((8S,11S)-8-isobutyl-4,7,10-trioxo-2-oxa-6,9-diaza-1,3(1,4)-dibenzenacyclododecaphane-11-yl)carbamate (**19e**)*.

^{1}H NMR in DMSO-d$_6$

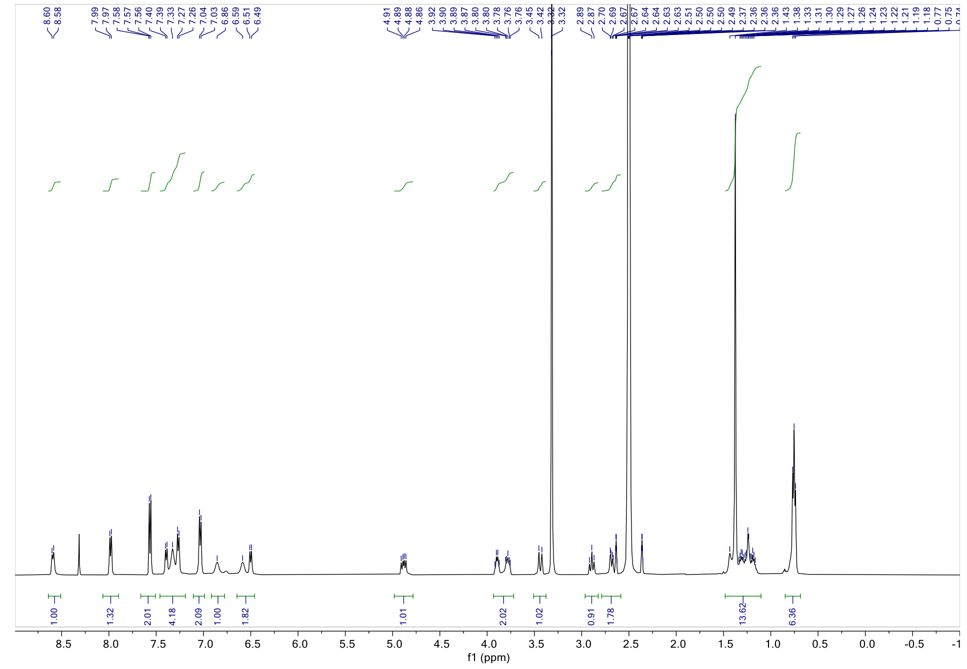

^{1}H NMR in DMSO-d$_6$ (expanded view)

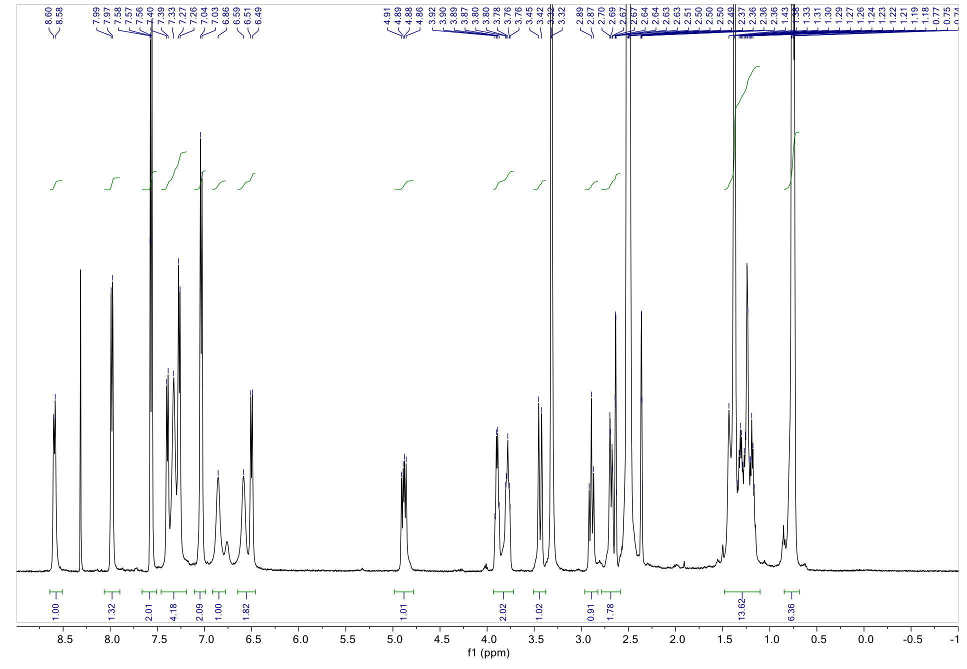

^{13}C NMR in DMSO-d_6

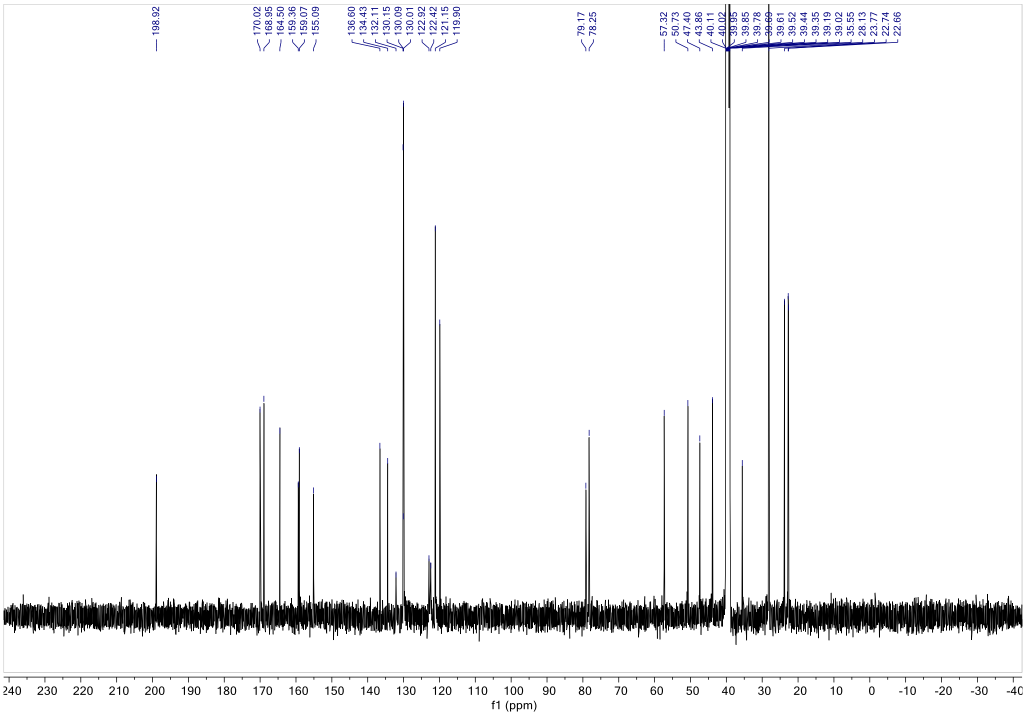

COSY in DMSO-d_6

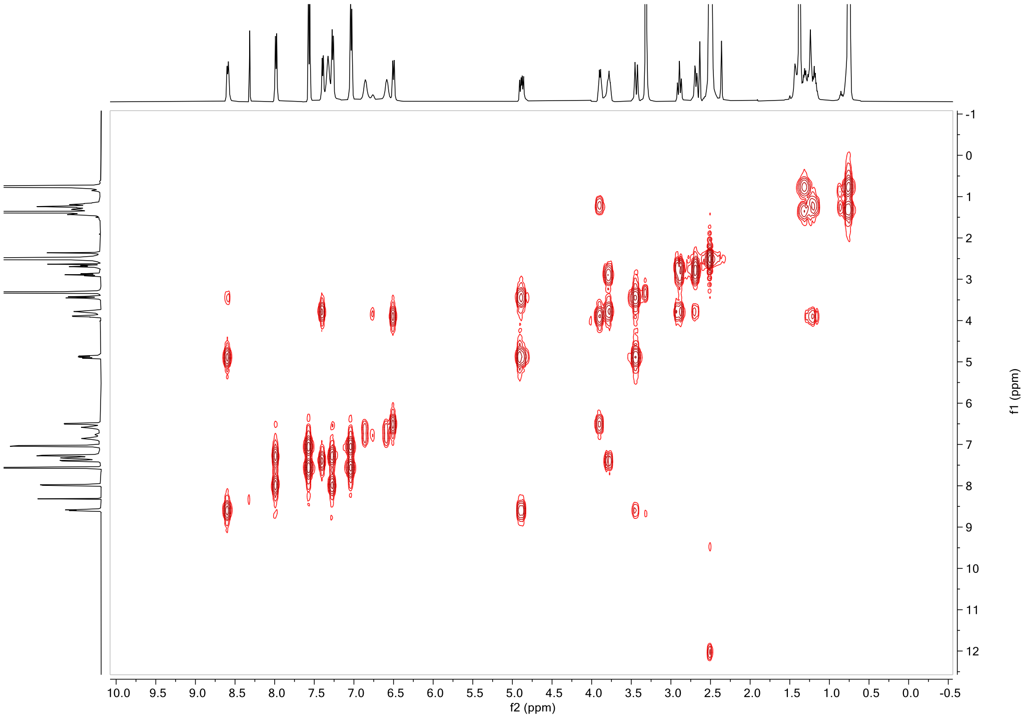

HSQC in DMSO-d$_6$

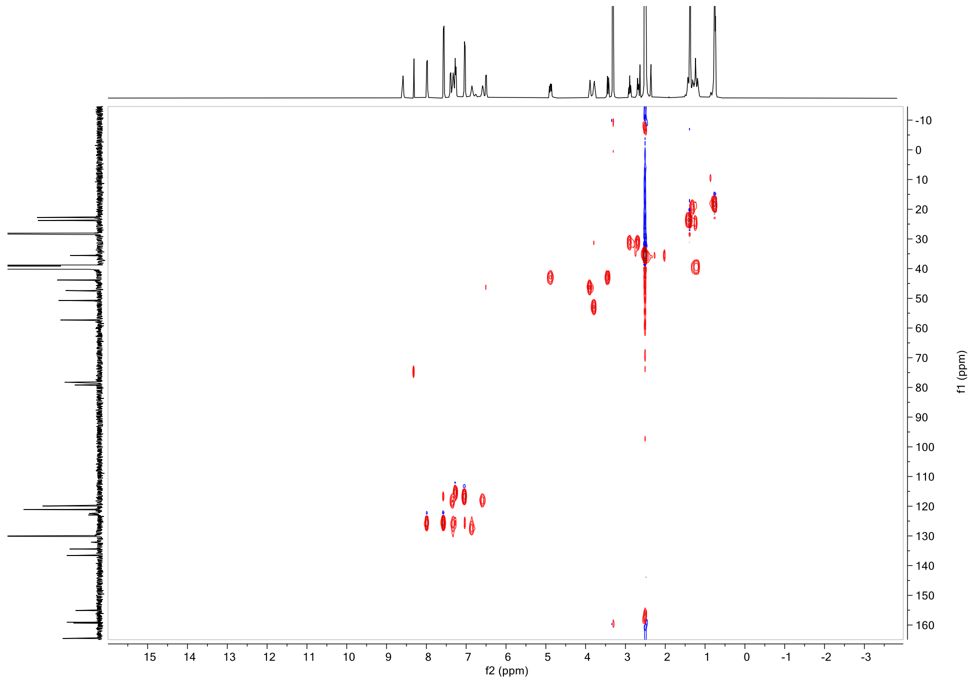

*tert-Butyl ((8S,11S)-8-(4-methylbenzyl)-4,7,10-trioxo-2-oxa-6,9-diaza-1,3(1,4)-dibenzenacyclododecaphane-11-yl)carbamate (**19h**)*.

^{1}H NMR in CDCl$_3$

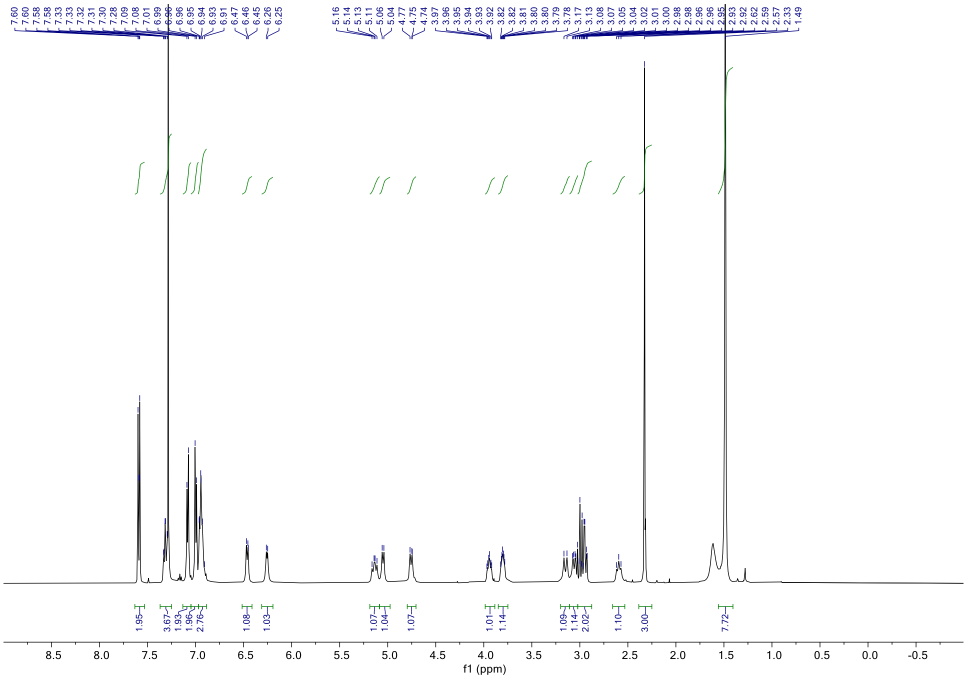

^{1}H NMR in $CDCl_3$ (expanded view)

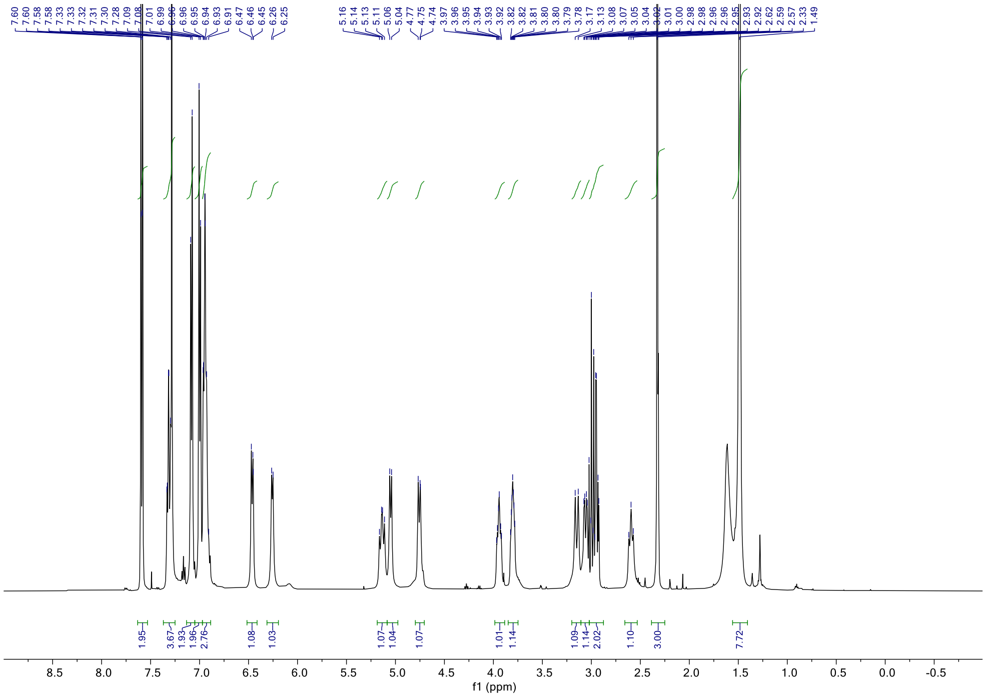

^{13}C NMR in $CDCl_3$

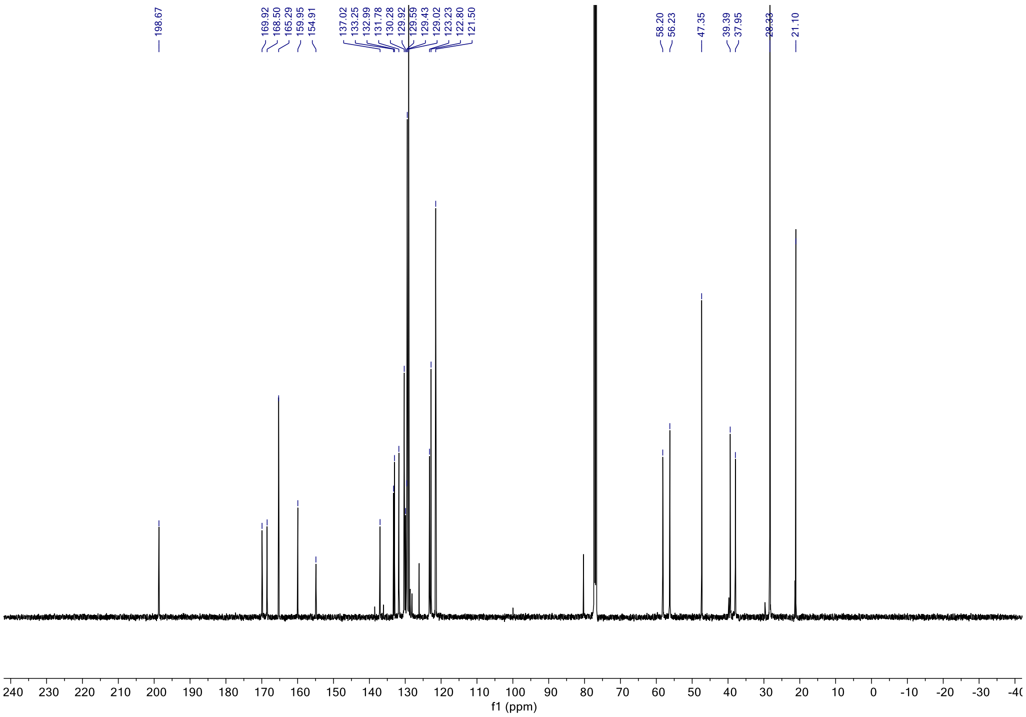

COSY in $CDCl_3$:

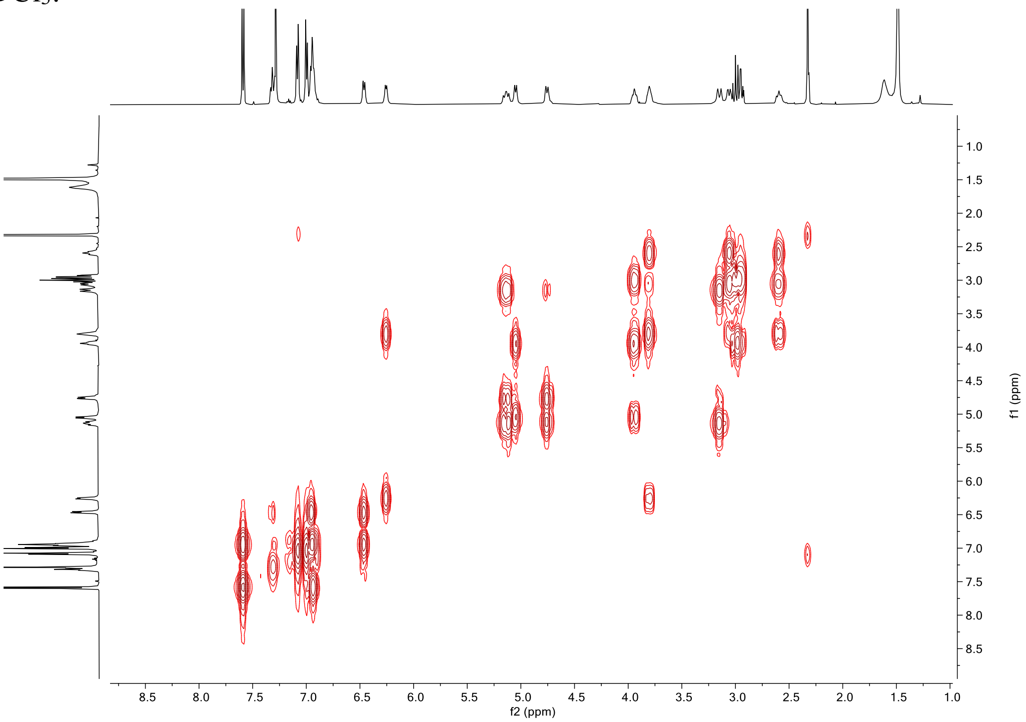

HSQC in $CDCl_3$

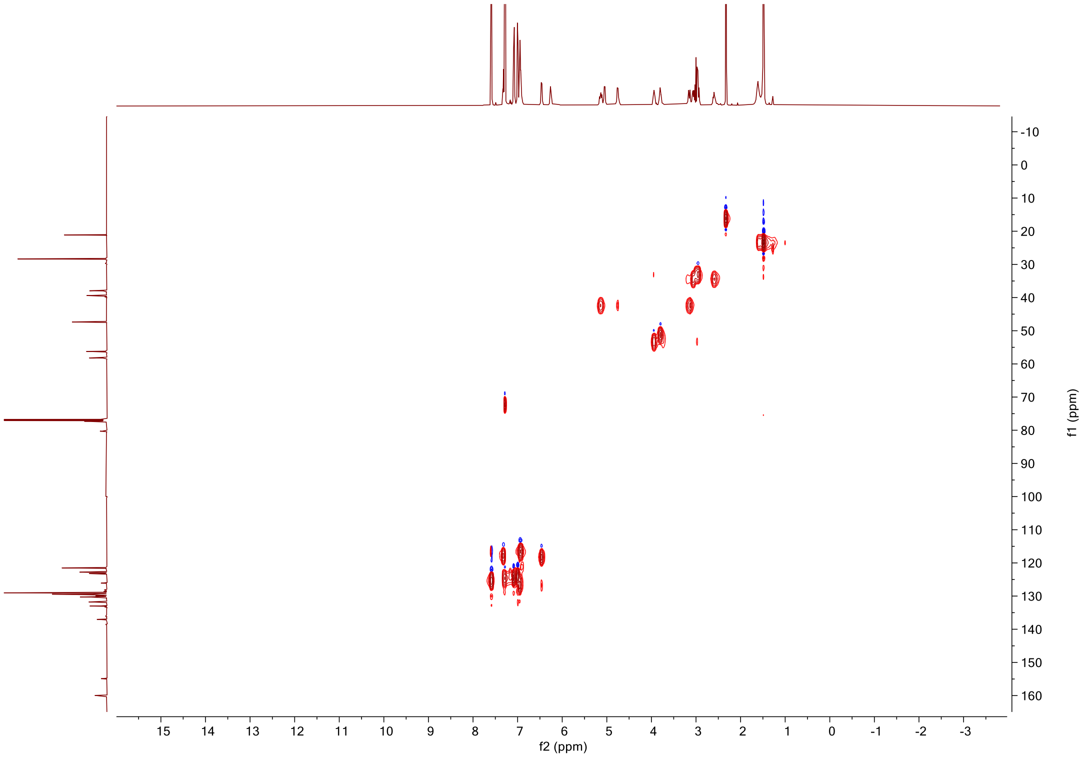

*tert-Butyl ((8S,11S)-8-(4-methylbenzyl)-4,7,10-trioxo-2-oxa-6,9-diaza-1,3(1,4)-dibenzenacyclododecaphane-11-yl)carbamate (**19h**)*.

^{1}H NMR in DMSO-d$_6$

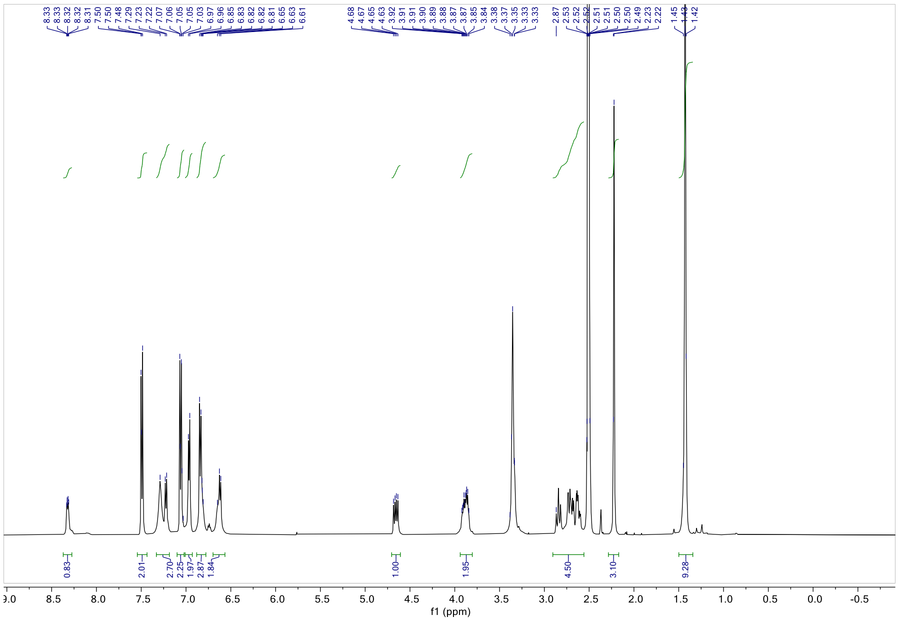

^{1}H NMR in DMSO-d$_6$ (expanded view)

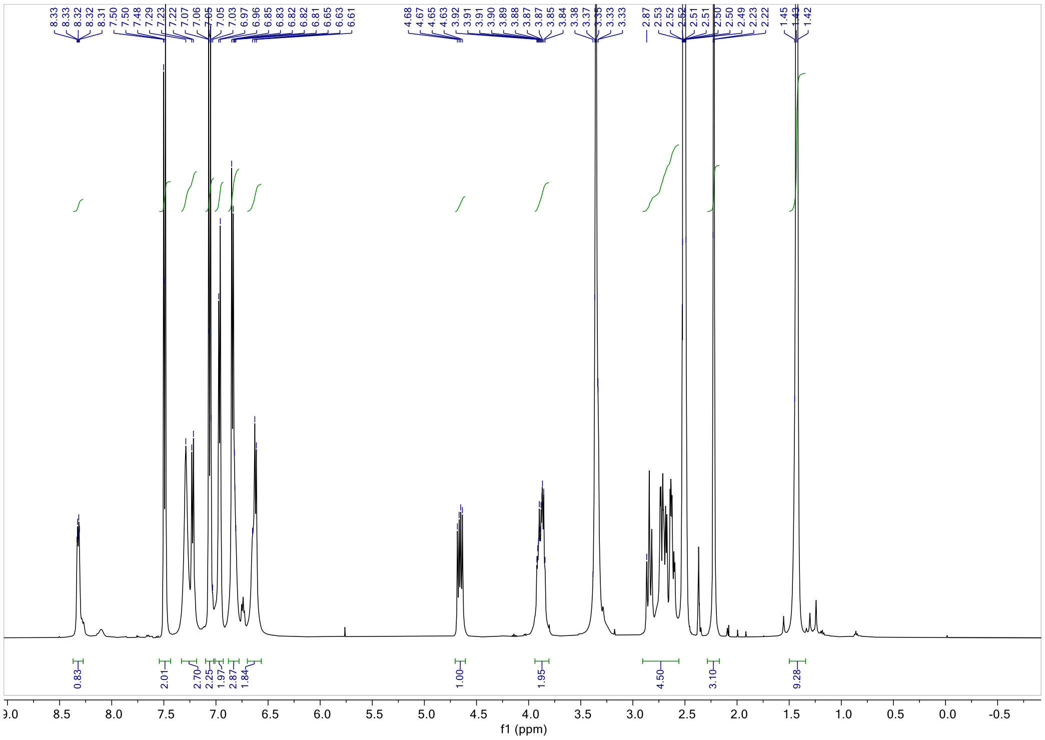

^{13}C NMR in DMSO-d$_6$

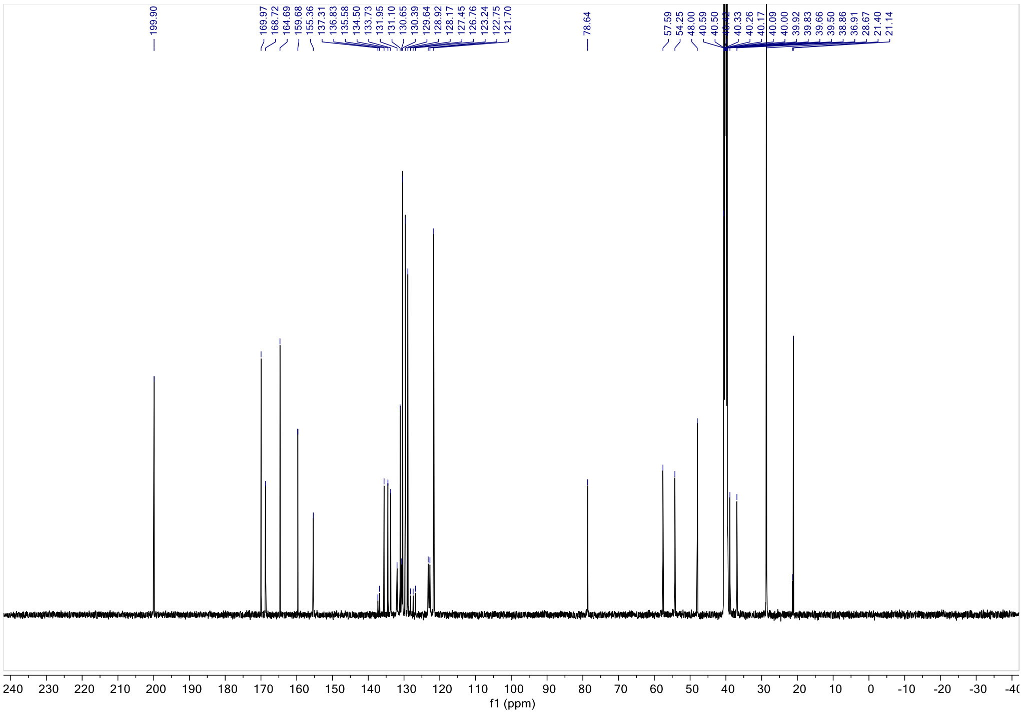

COSY in DMSO-d$_6$

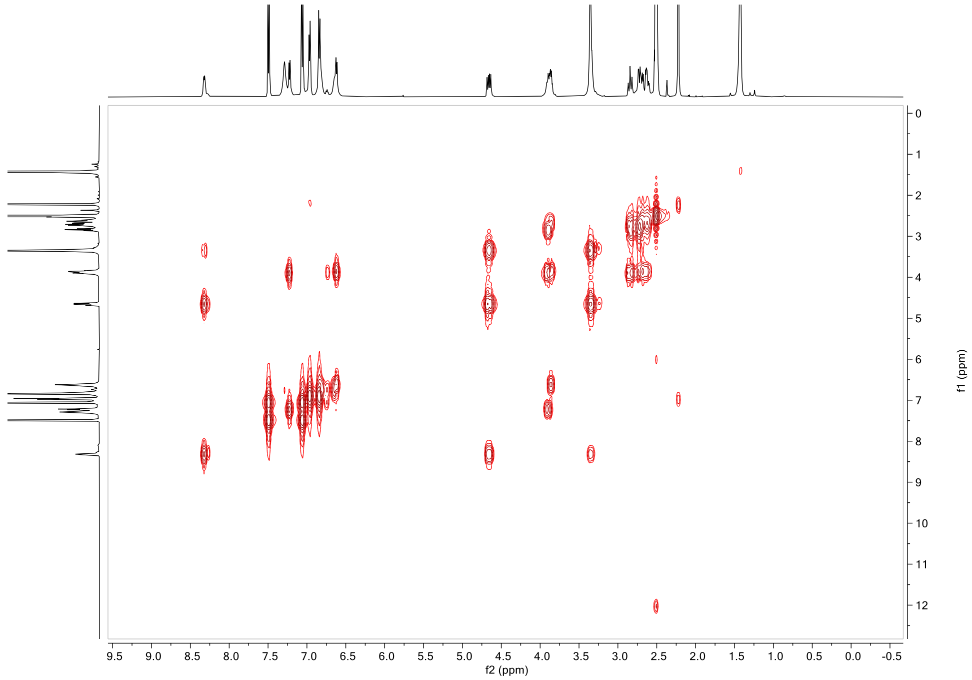

HSQC in DMSO-d_6

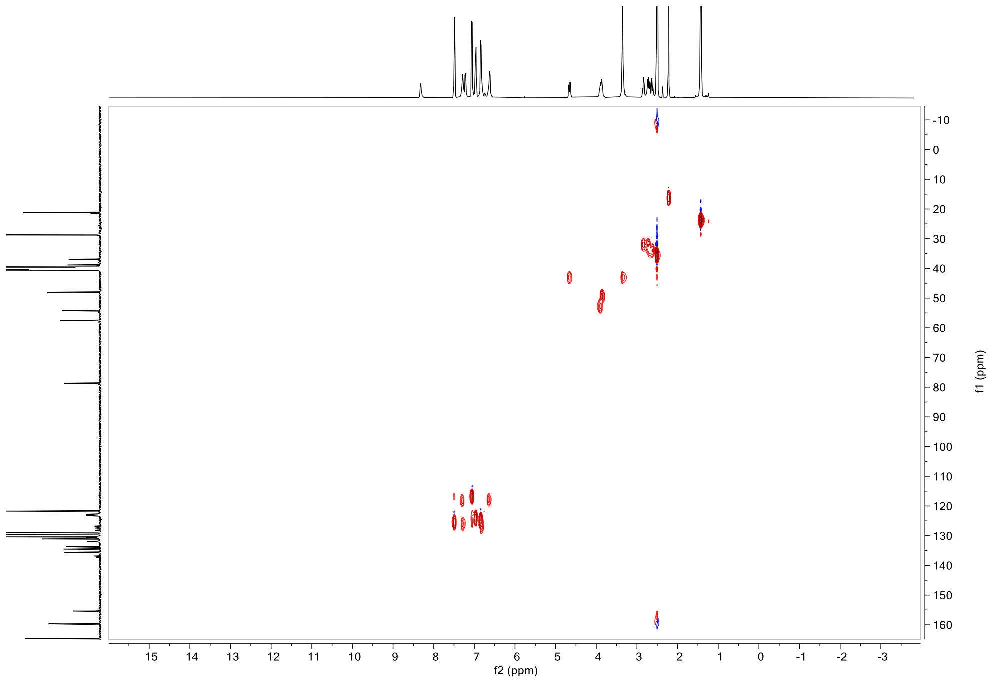

tert-Butyl ((8S,11S)-8-(4-fluorobenzyl)-4,7,10-trioxo-2-oxa-6,9-diaza-1,3(1,4)-dibenzenacyclododecaphane-11-yl)carbamate ***(19i)***.

^{1}H NMR in $CDCl_3$

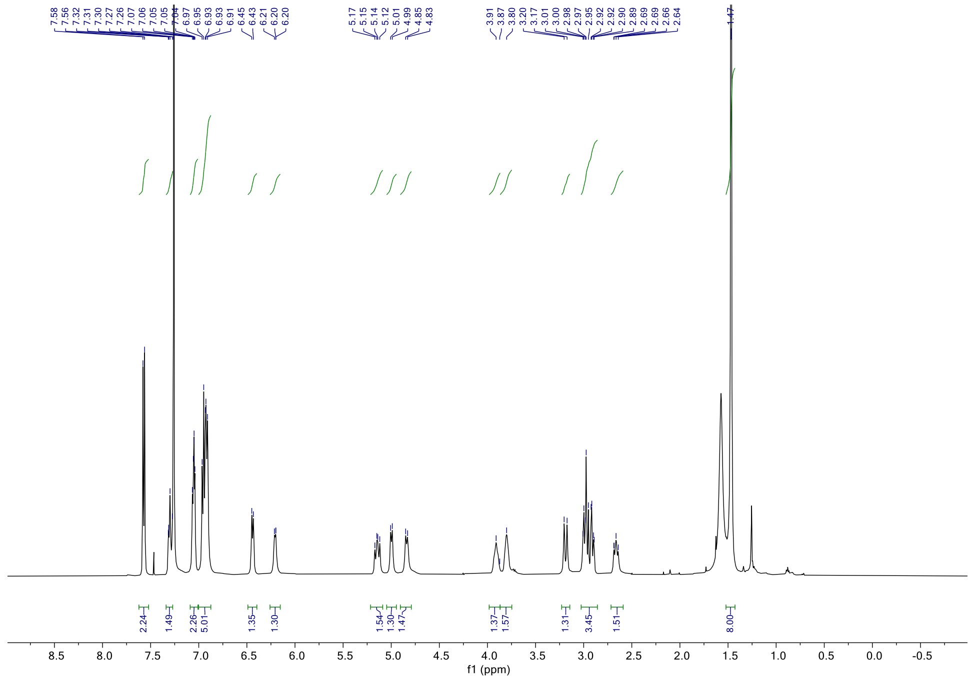

^{13}C NMR in $CDCl_3$

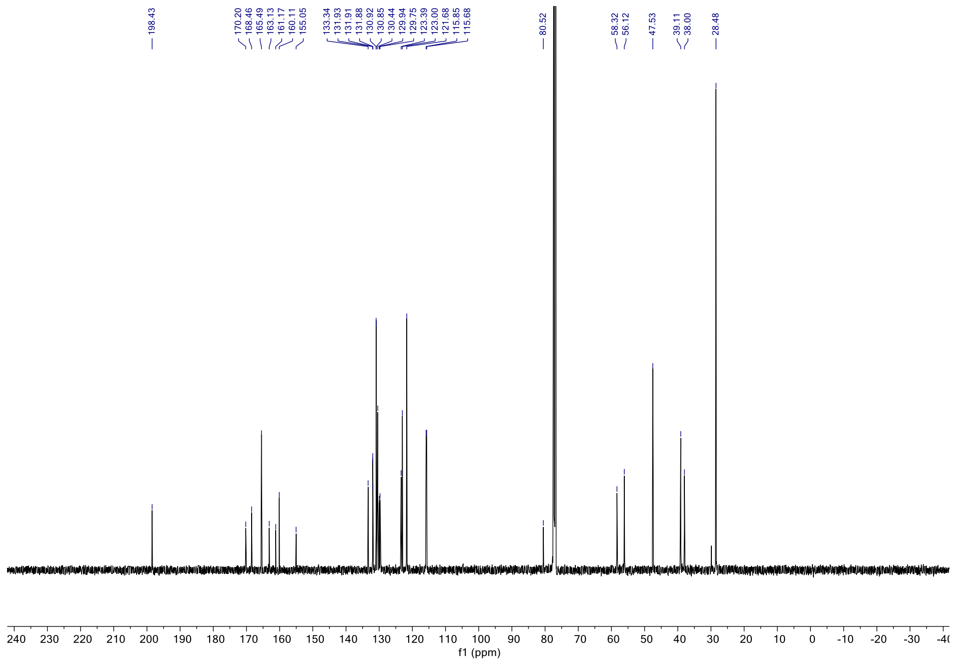

COSY in $CDCl_3$

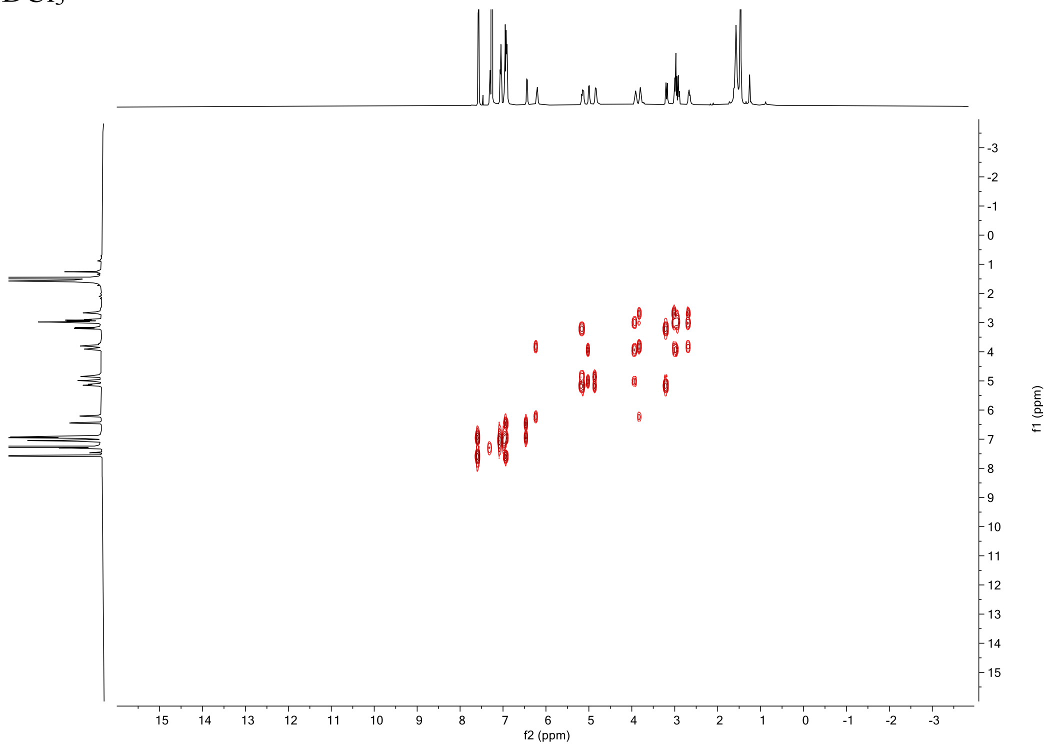

HSQC in $CDCl_3$

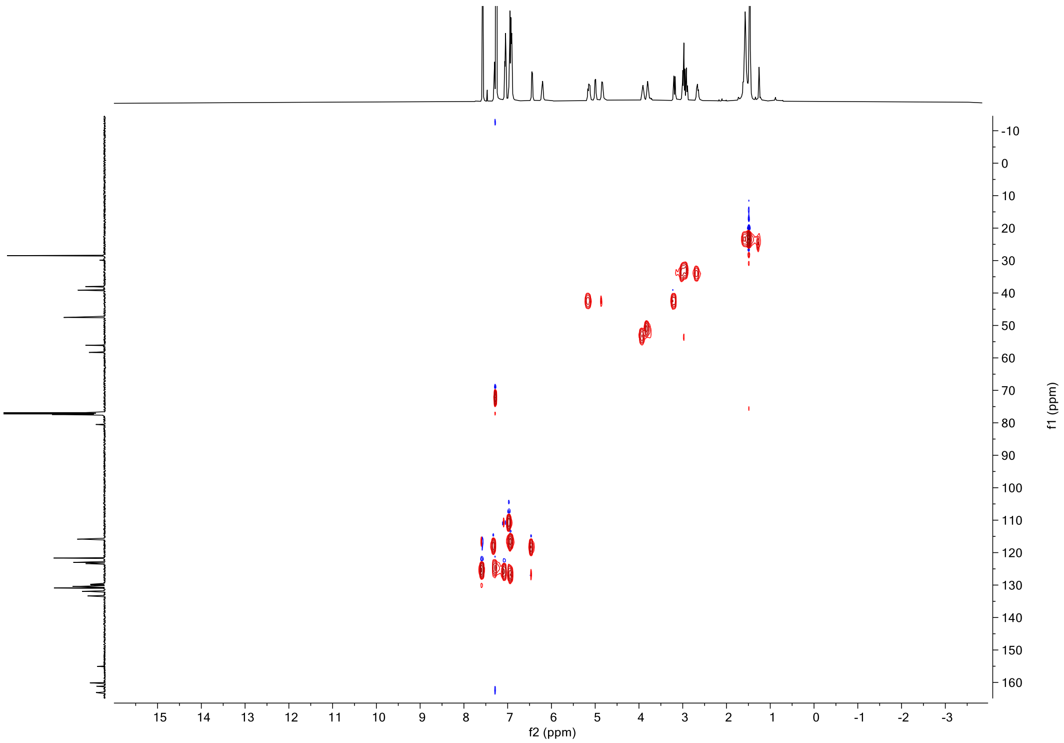

*tert-Butyl ((8S,11S)-8-(4-fluorobenzyl)-4,7,10-trioxo-2-oxa-6,9-diaza-1,3(1,4)-dibenzenacyclododecaphane-11-yl)carbamate (**19i**).*

^{1}H NMR in DMSO-d_6

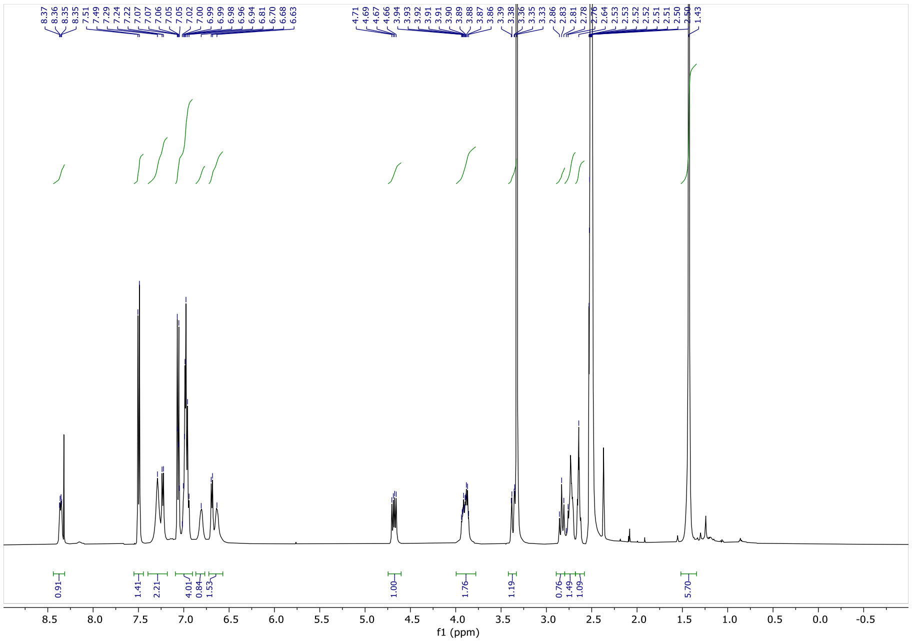

^{13}C NMR in DMSO-d_6

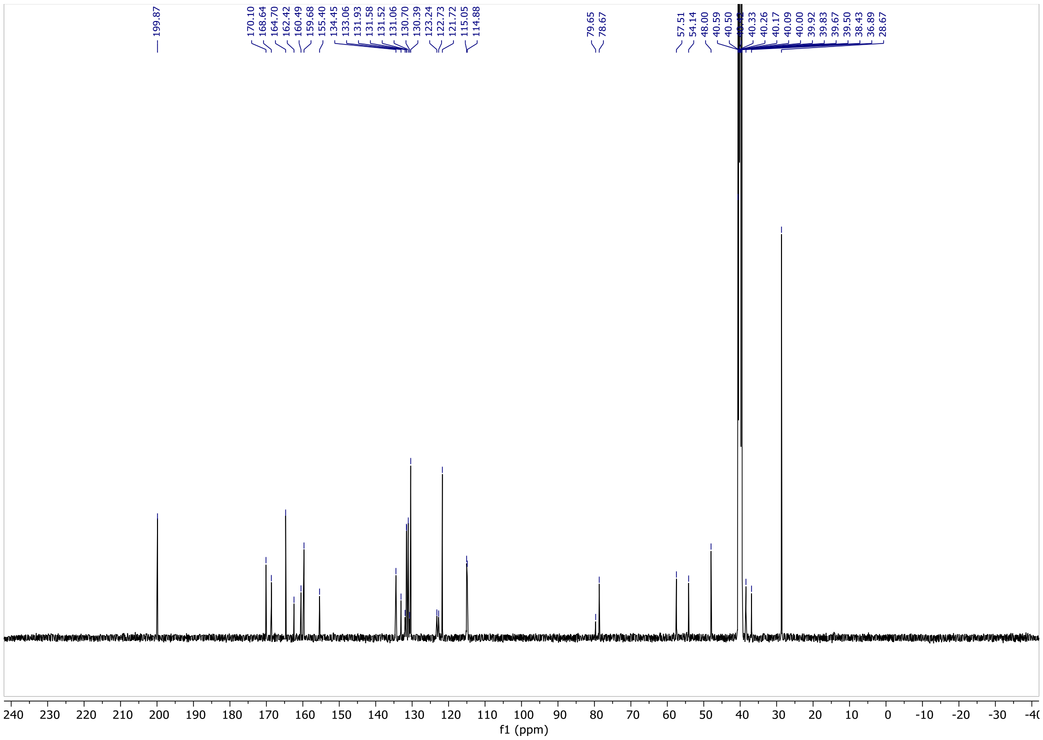

COSY in DMSO-d_6

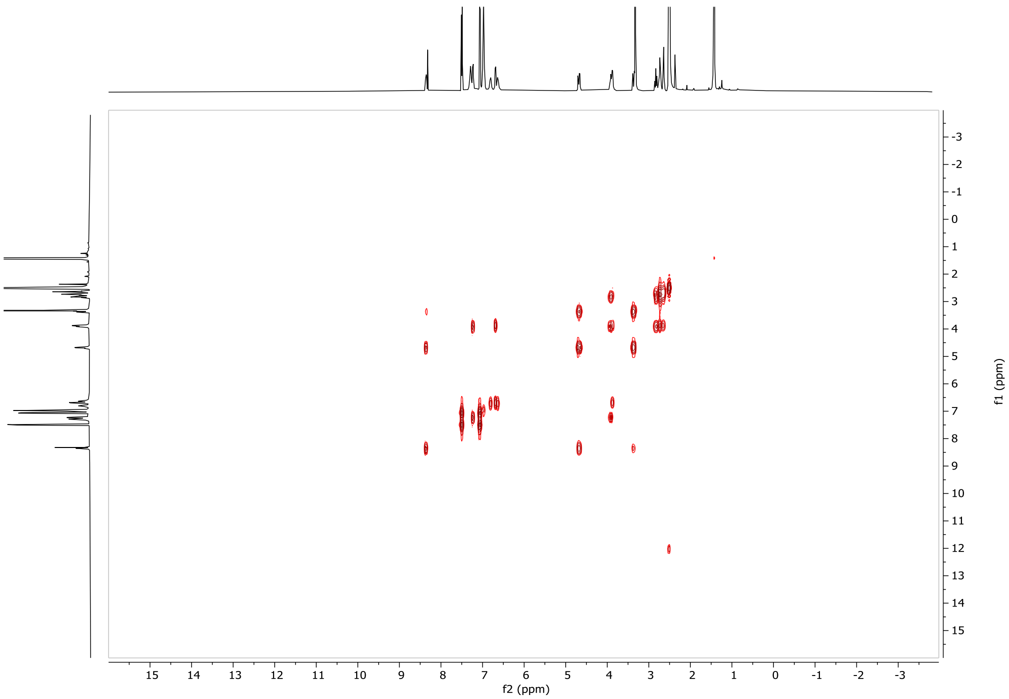

HSQC in DMSO-d_6

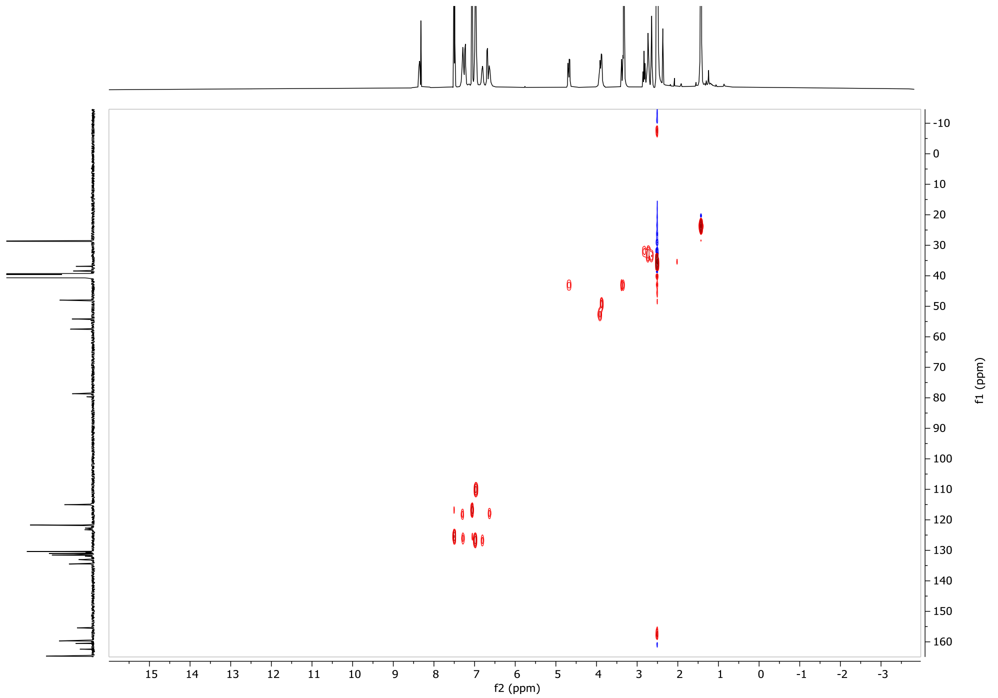

tert-Butyl ((8S,11S)-8-benzyl-4,7,10-trioxo-2-oxa-6,9-diaza-1,3(1,4)-dibenzenacyclotridecaphane-11-yl)carbamate ***(20c)***.

^{1}H NMR in $CDCl_3$

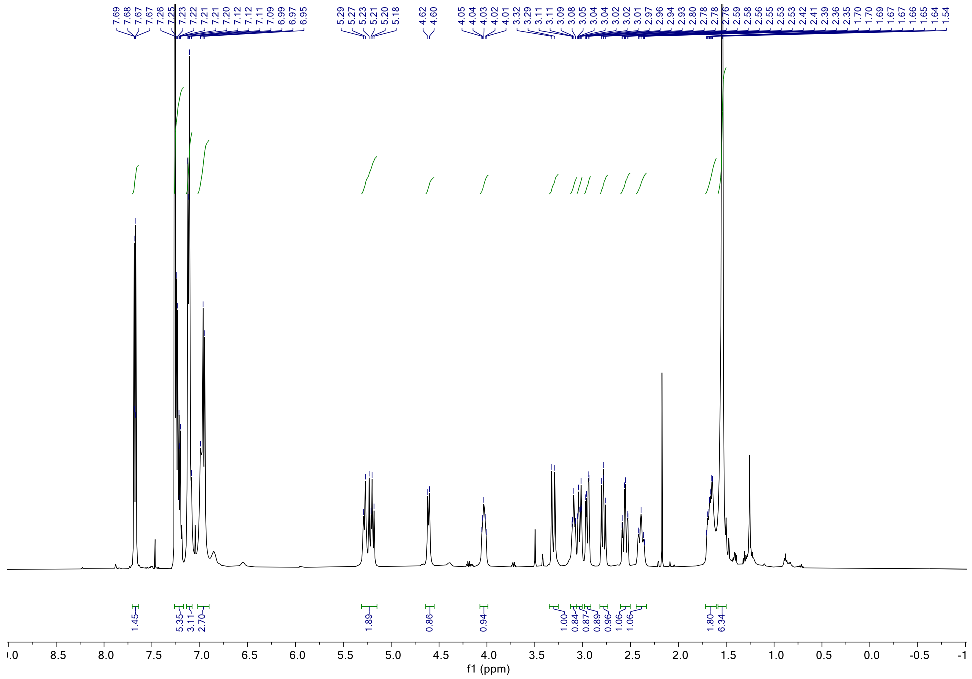

^{13}C NMR in $CDCl_3$

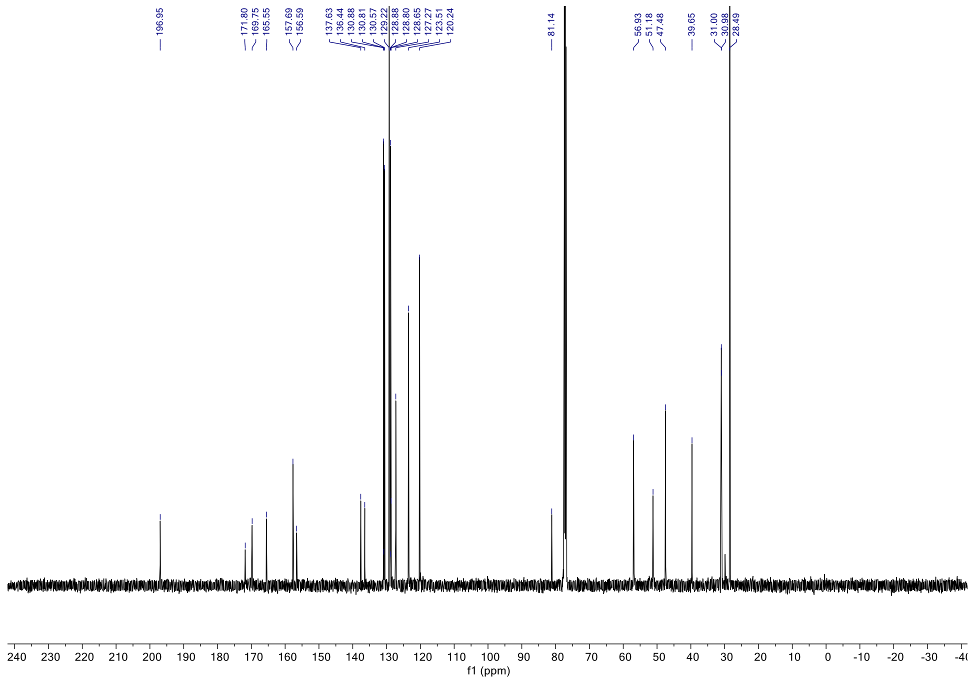

COSY in $CDCl_3$

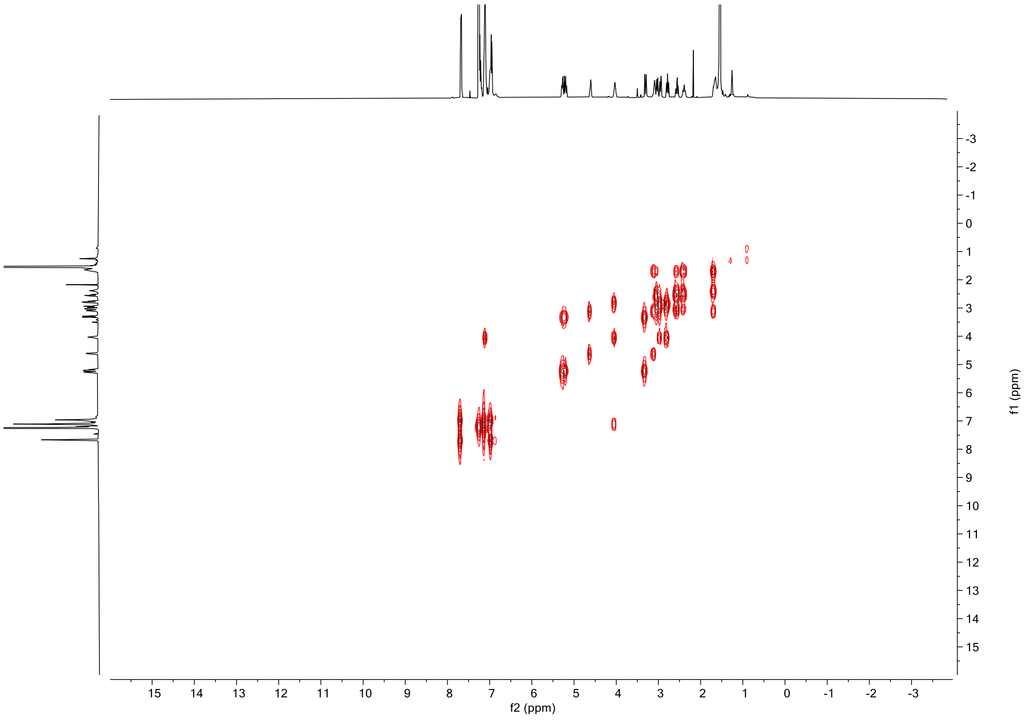

HSQC in $CDCl_3$

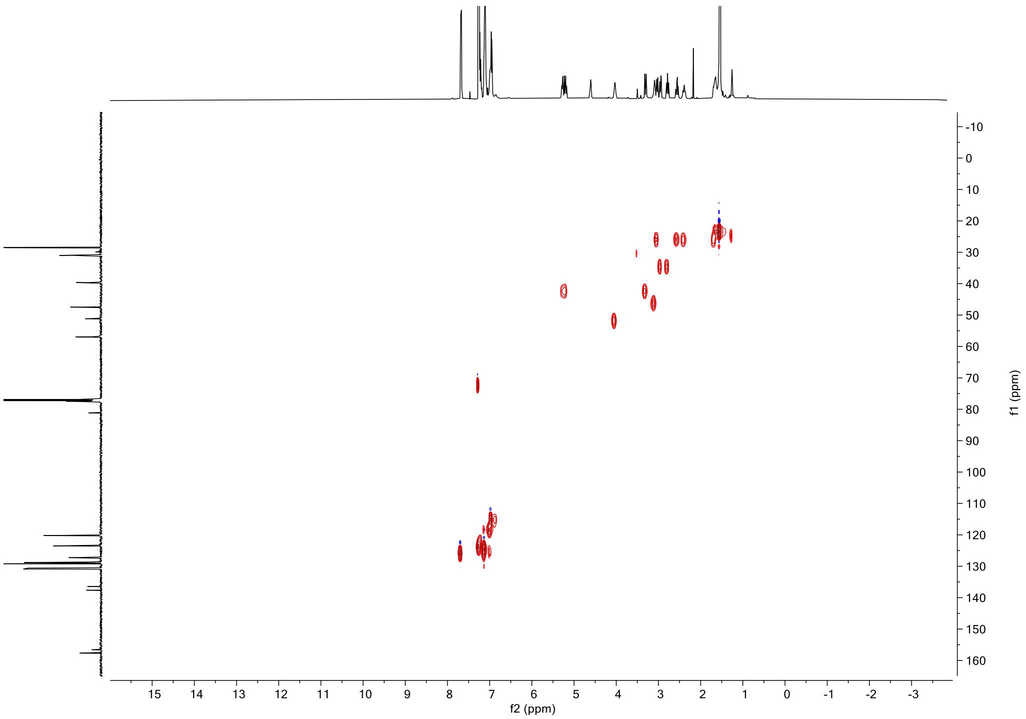

*tert-Butyl ((8S,11S)-8-benzyl-4,7,10-trioxo-2-oxa-6,9-diaza-1,3(1,4)-dibenzenacyclotridecaphane-11-yl)carbamate (**20c**).*

^{1}H NMR in DMSO-d_6

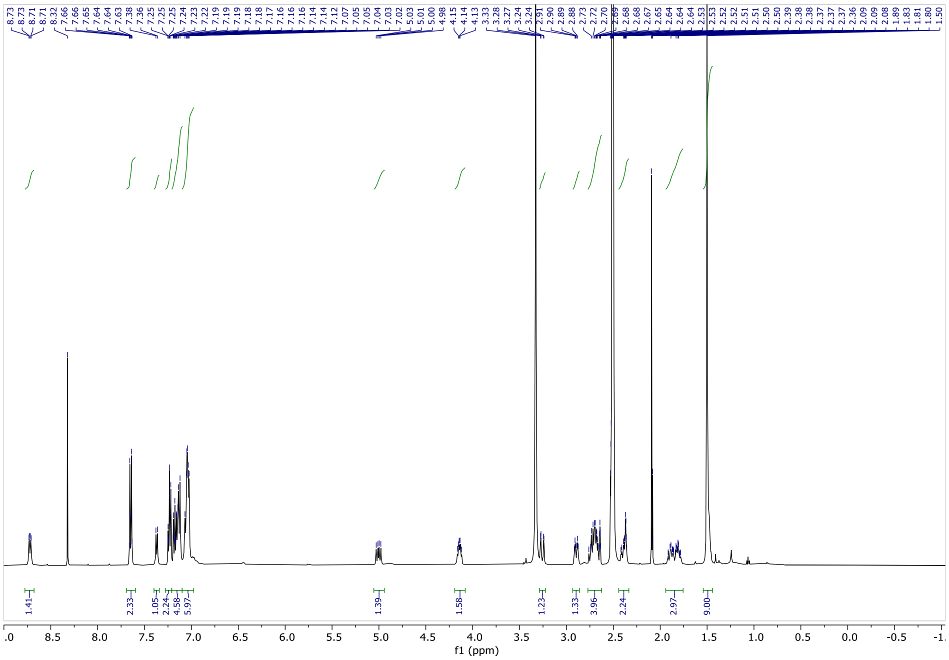

^1H NMR in DMSO-d_6 (expanded view)

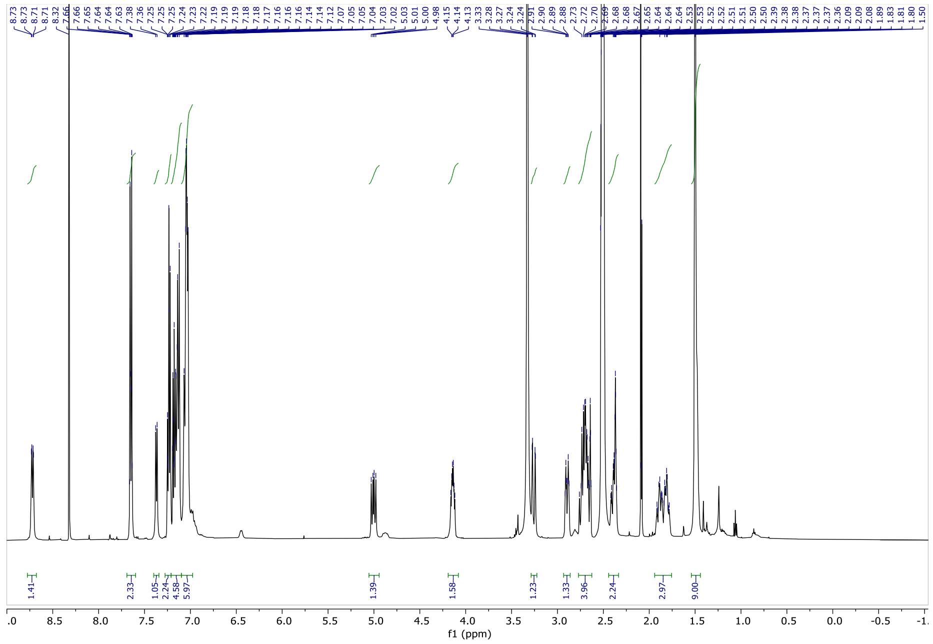

^{13}C NMR in DMSO-d_6

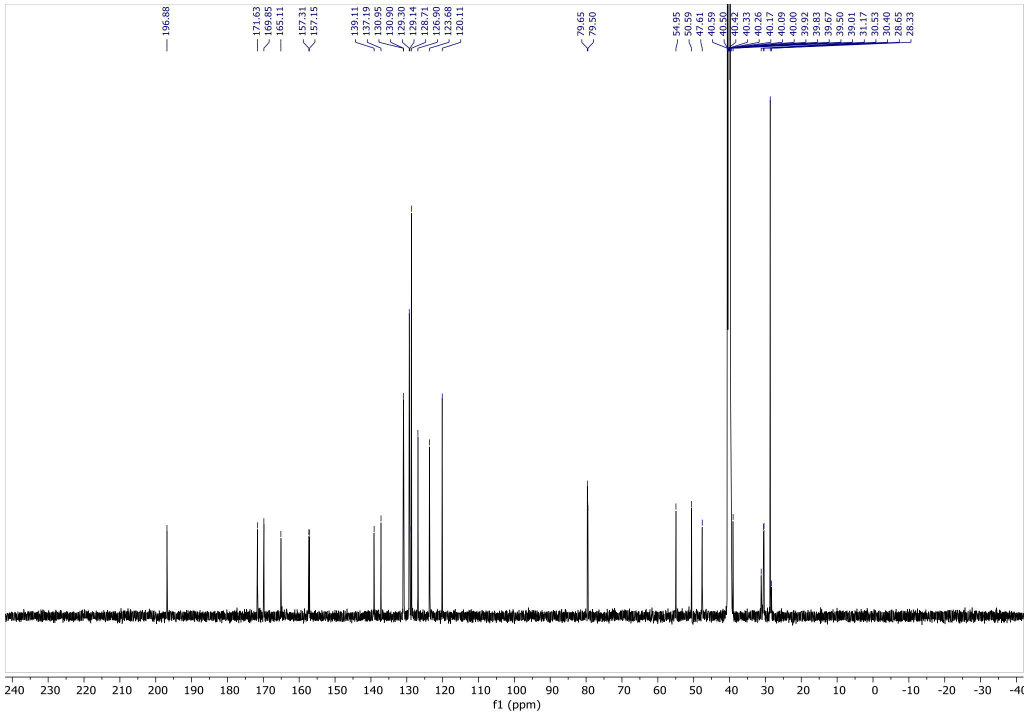

COSY in DMSO-d_6

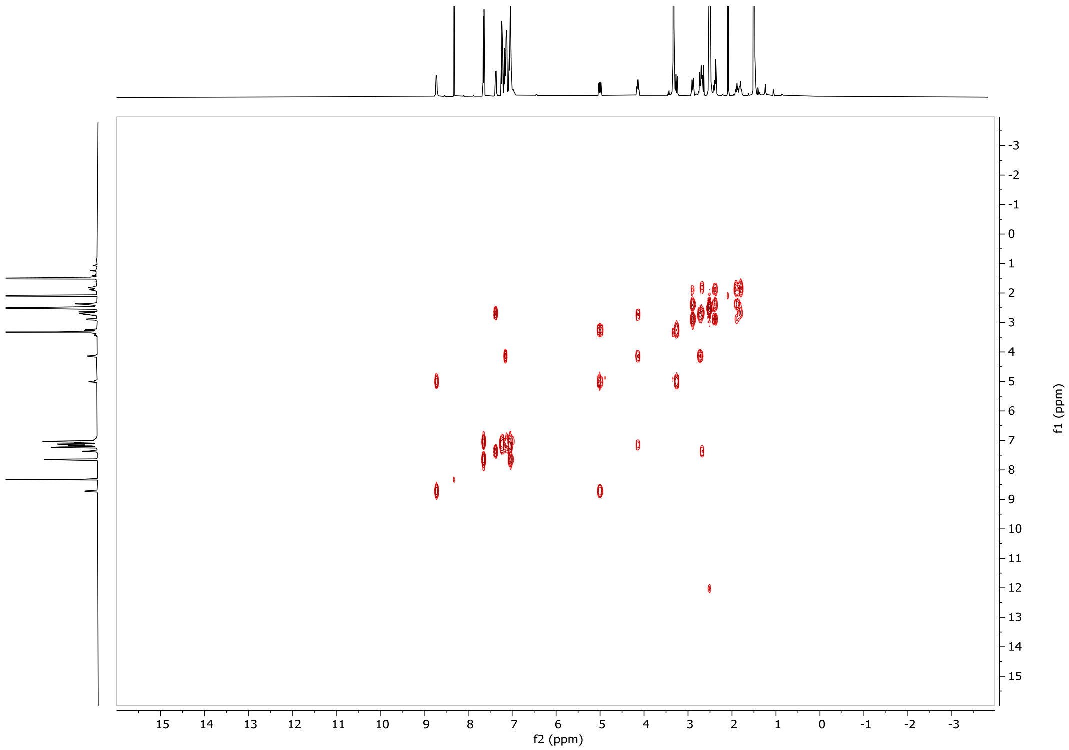

HSQC in DMSO-d_6

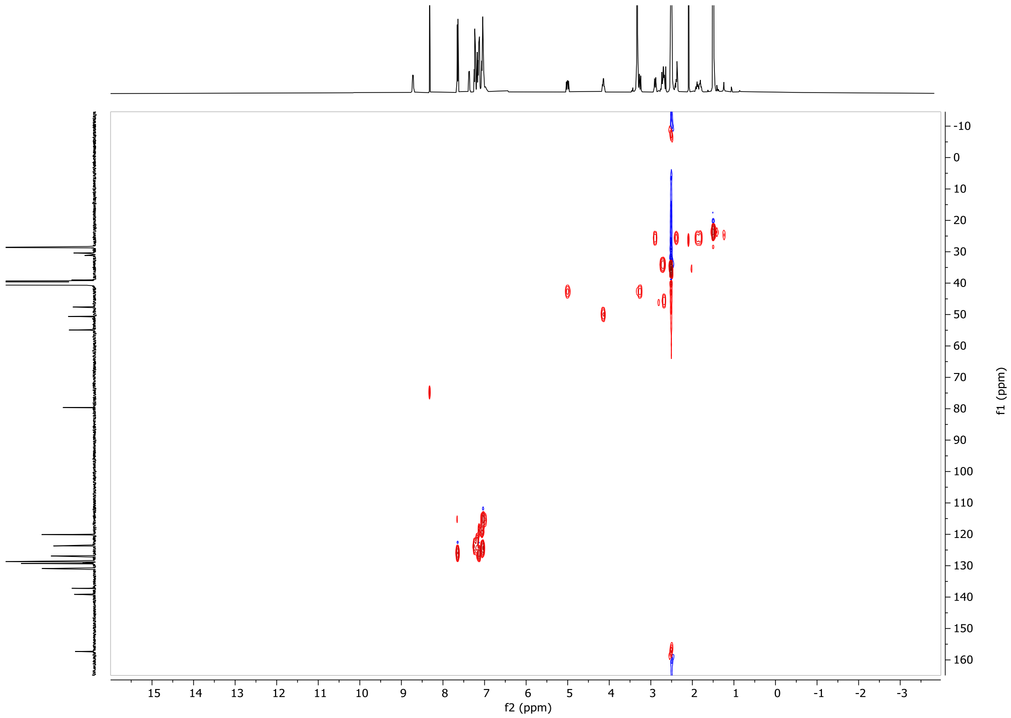

*tert-Butyl ((9S,12S)-9-benzyl-4,7,11-trioxo-2-oxa-6,10-diaza-1,3(1,4)-dibenzenacyclotridecaphane-12-yl)carbamate (**21c**).*

^{1}H NMR in $CDCl_3$

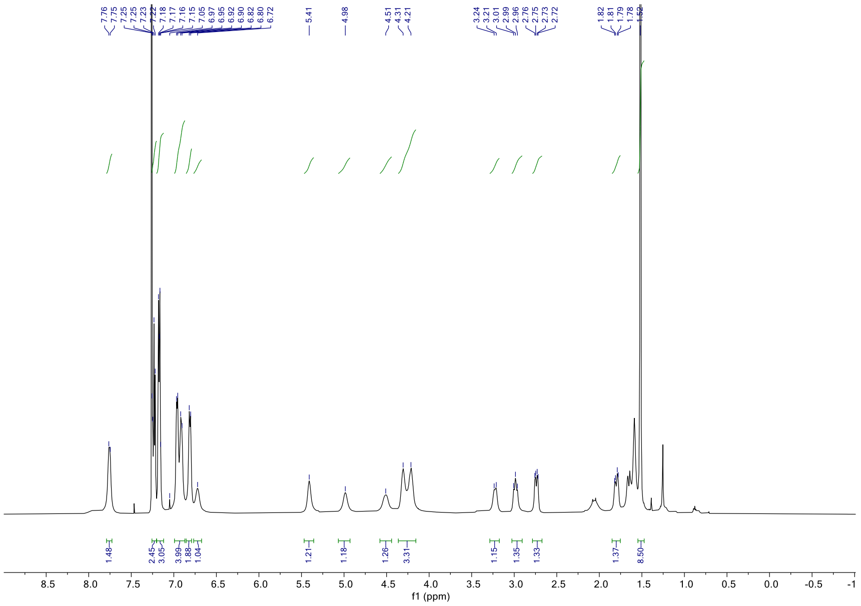

^{1}H NMR in $CDCl_3$ (expanded view)

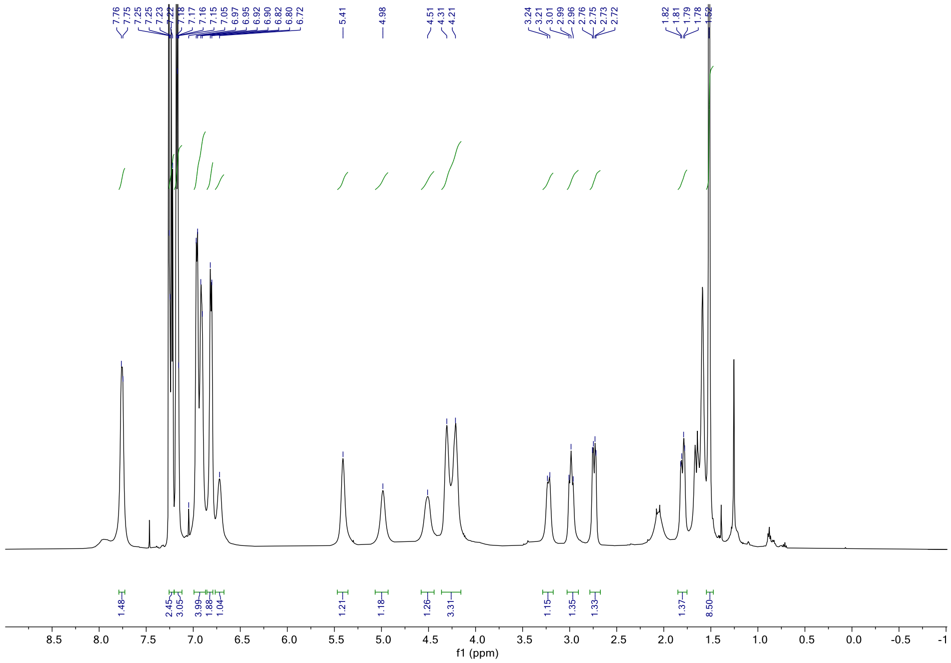

^{13}C NMR in $CDCl_3$

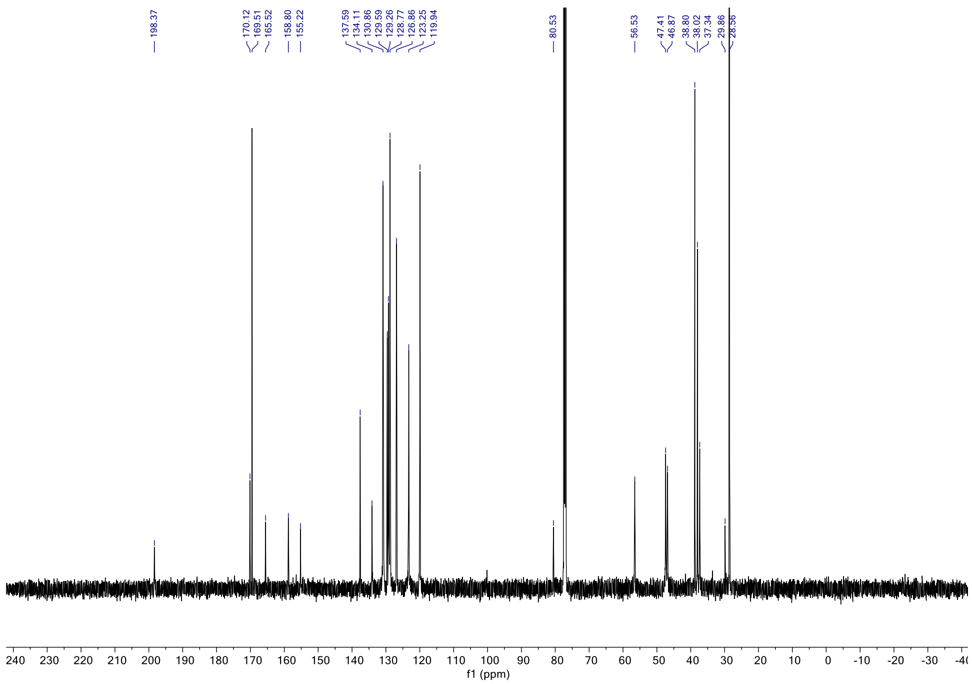

COSY in $CDCl_3$

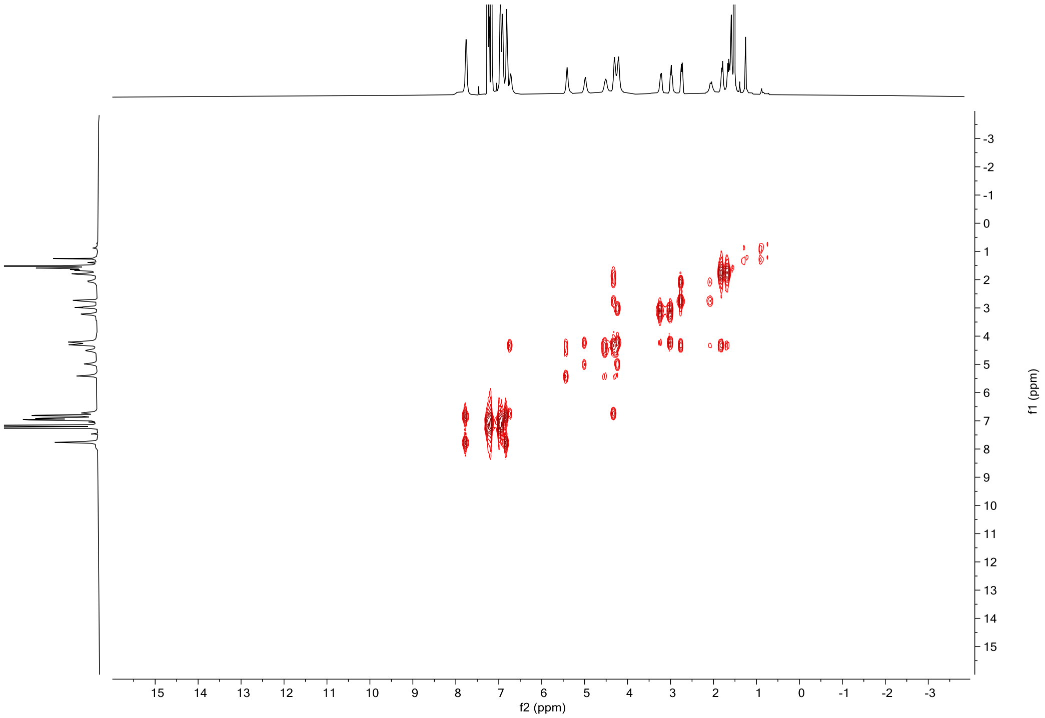

HSQC in $CDCl_3$

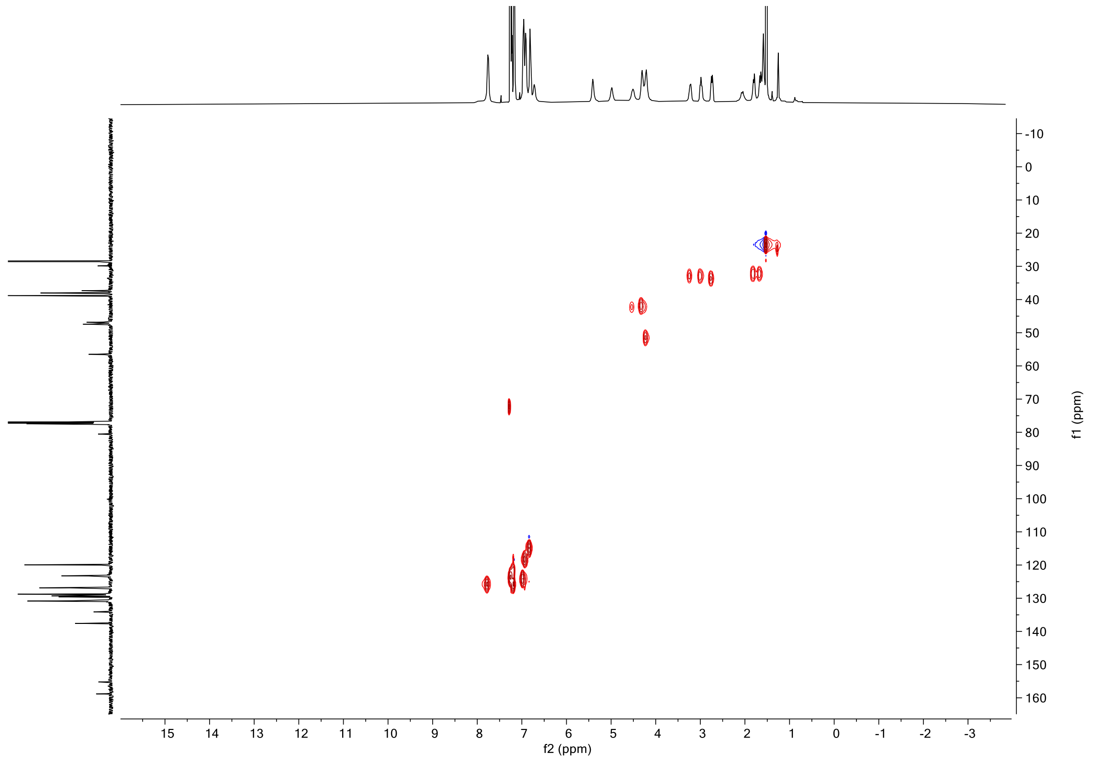

*tert-Butyl ((9S,12S)-9-benzyl-4,7,11-trioxo-2-oxa-6,10-diaza-1,3(1,4)-dibenzenacyclotridecaphane-12-yl)carbamate (**21c**).*

^{1}H NMR in DMSO-d_6

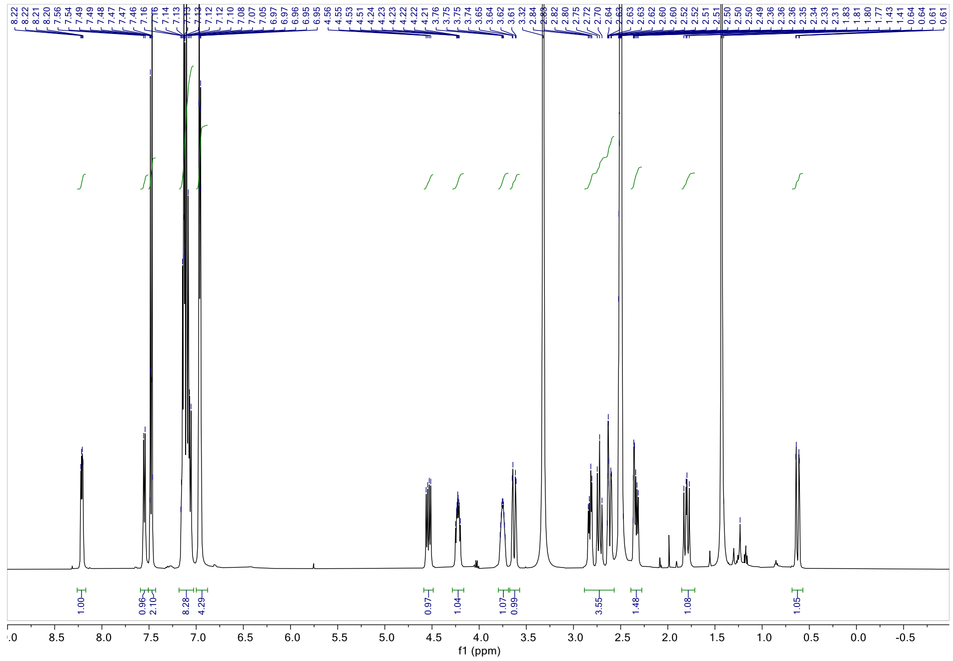

^{13}C NMR in DMSO-d_6

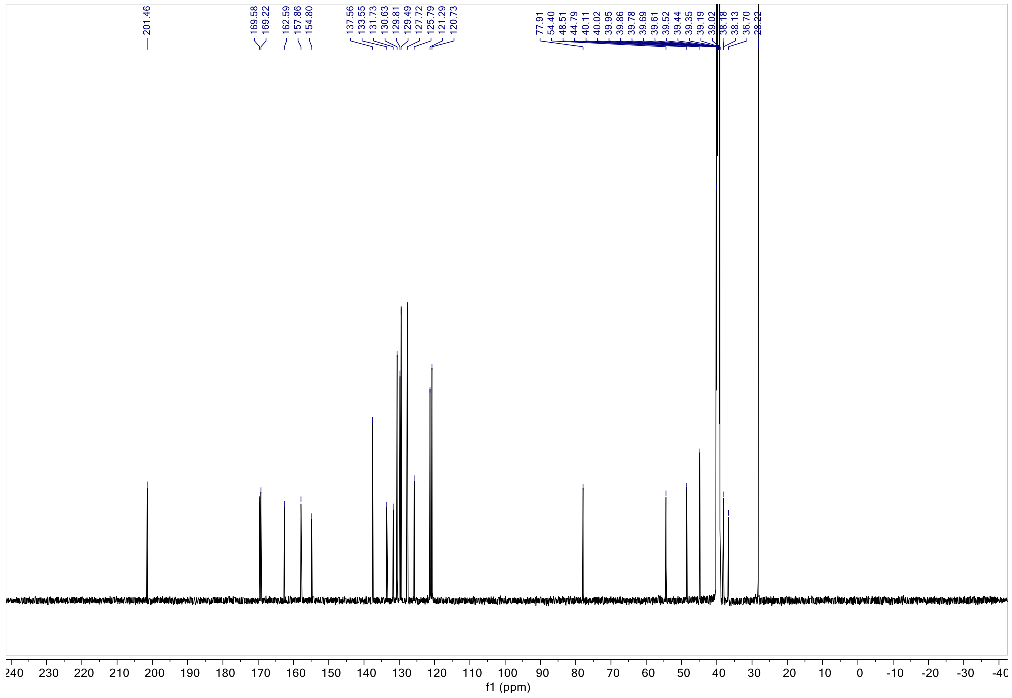

COSY in DMSO-d_6

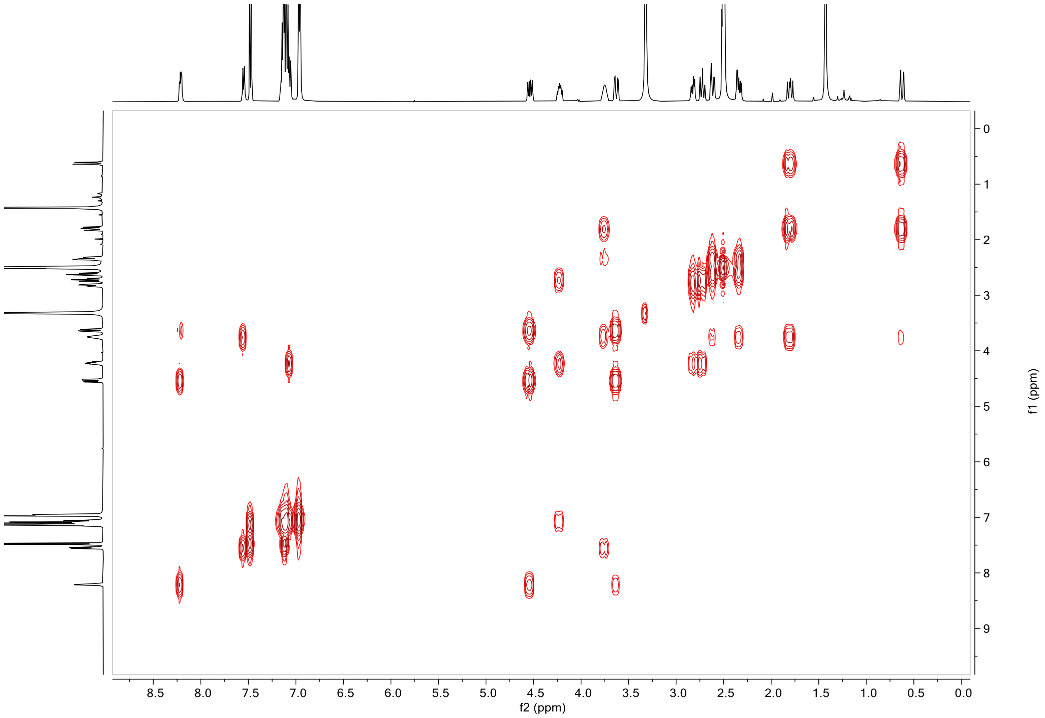

HSQC in DMSO-d$_6$

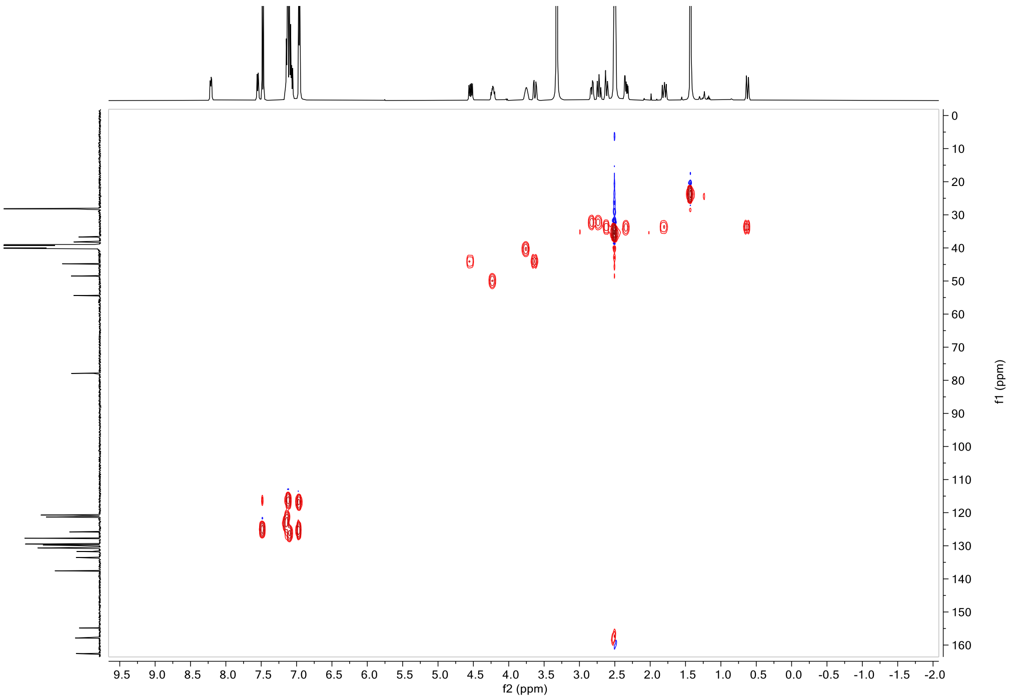

tert-Butyl ((9S,12S)-9-isobutyl-4,7,11-trioxo-2-oxa-6,10-diaza-1,3(1,4)-dibenzenacyclotridecaphane-12-yl)carbamate ***(21e)***.

^{1}H NMR in $CDCl_3$

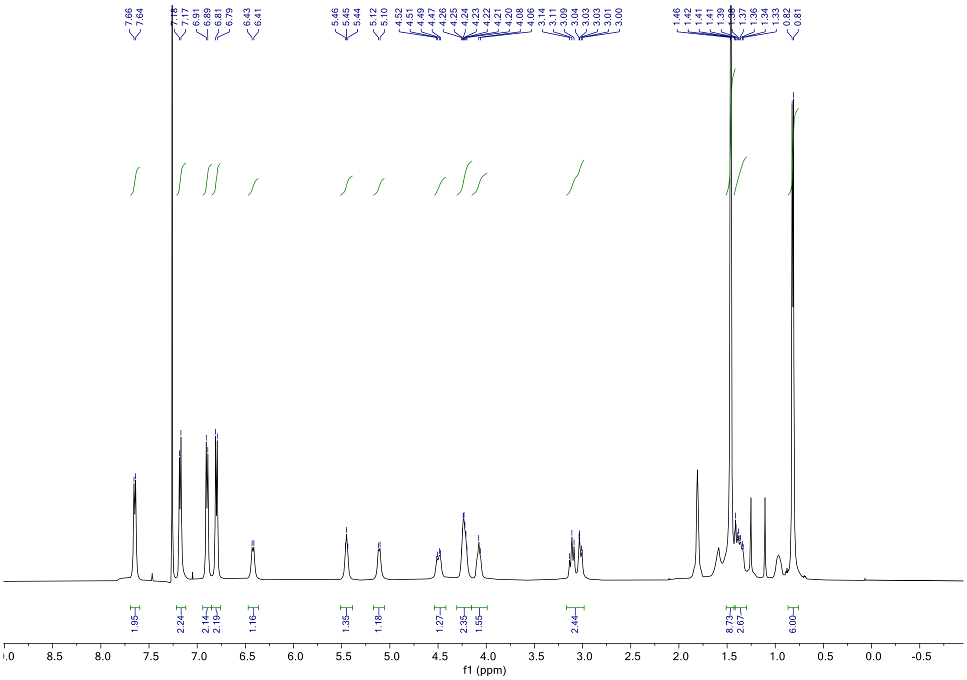

^{1}H NMR in $CDCl_3$ (expanded view)

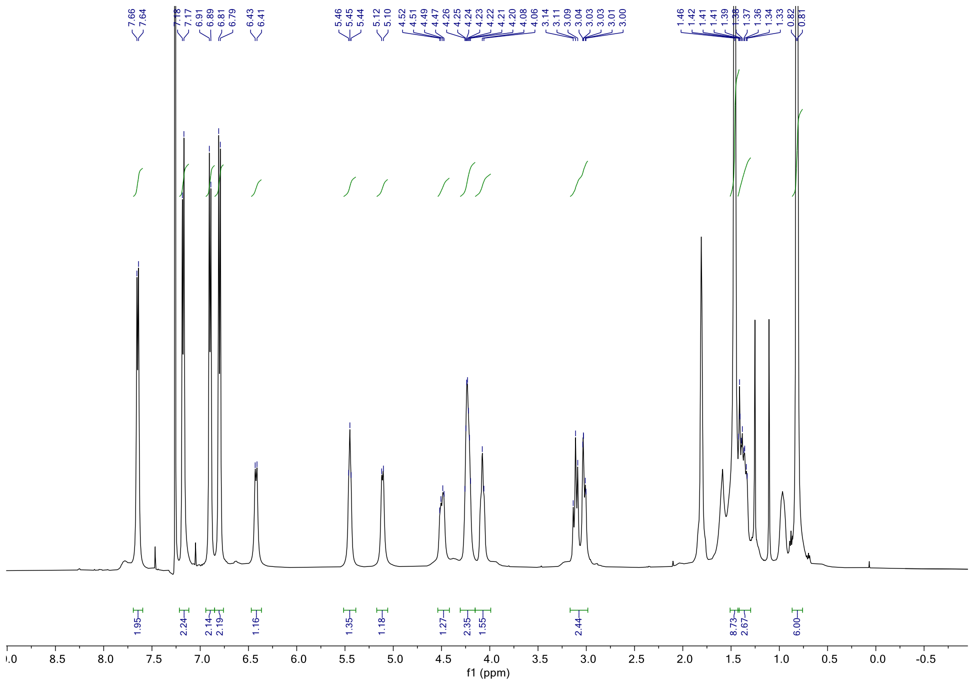

^{13}C NMR in $CDCl_3$

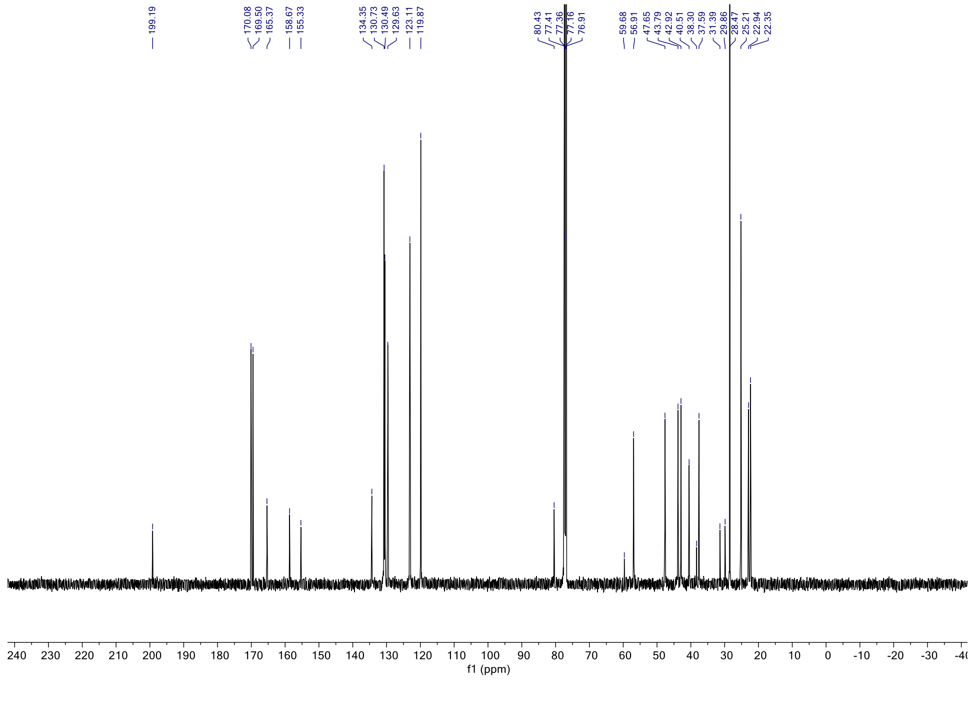

COSY in $CDCl_3$

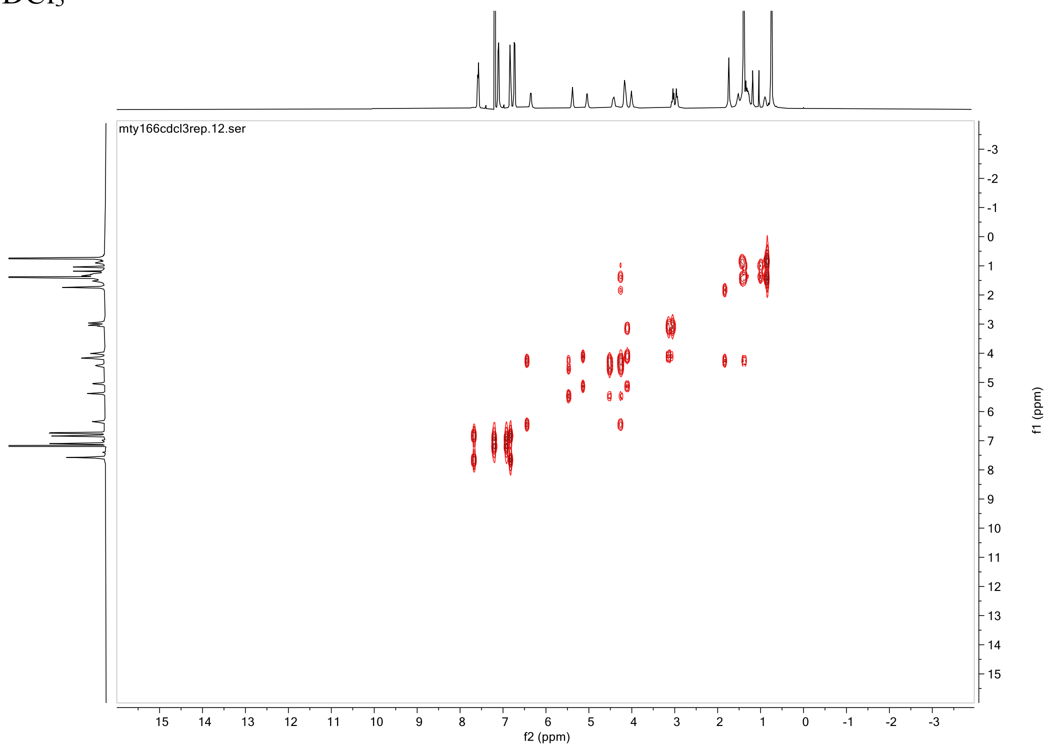

HSQC in $CDCl_3$

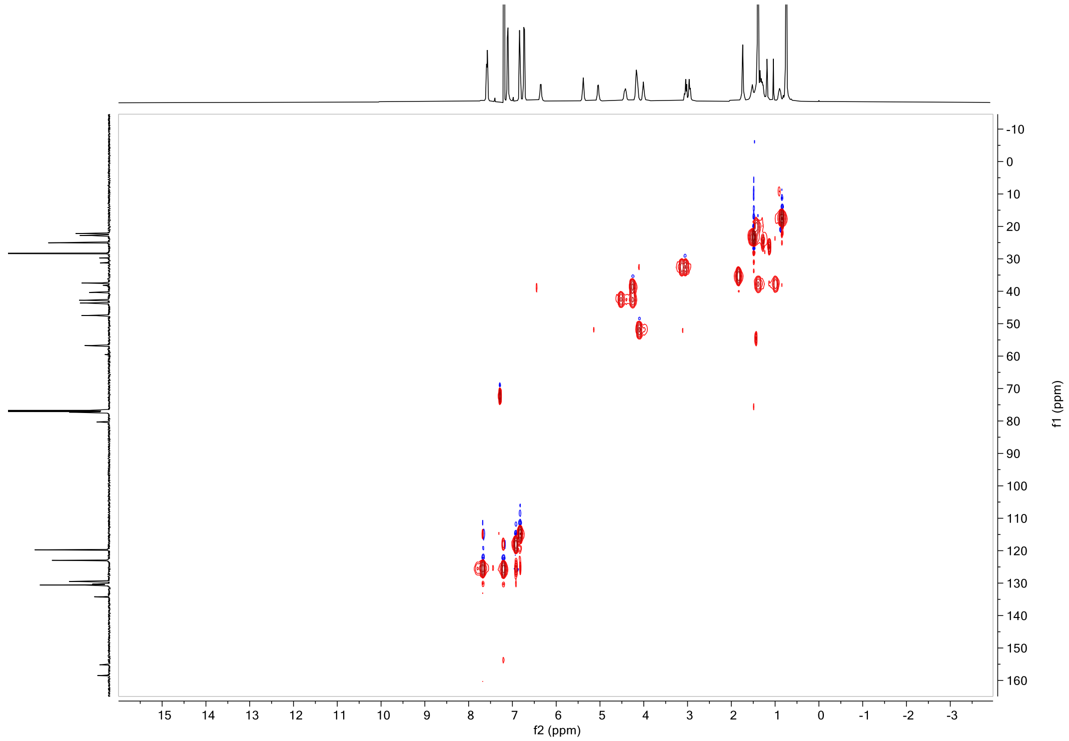

*tert-Butyl ((9S,12S)-9-isobutyl-4,7,11-trioxo-2-oxa-6,10-diaza-1,3(1,4)-dibenzenacyclotridecaphane-12-yl)carbamate (**21e**).*

^{1}H NMR in DMSO-d_6

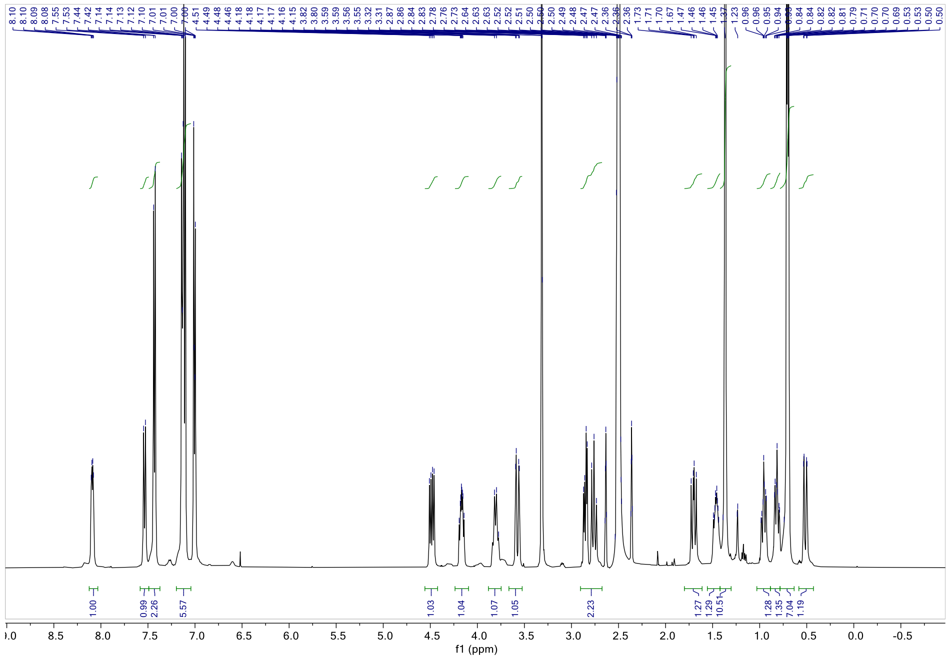

^{13}C NMR in DMSO-d_6

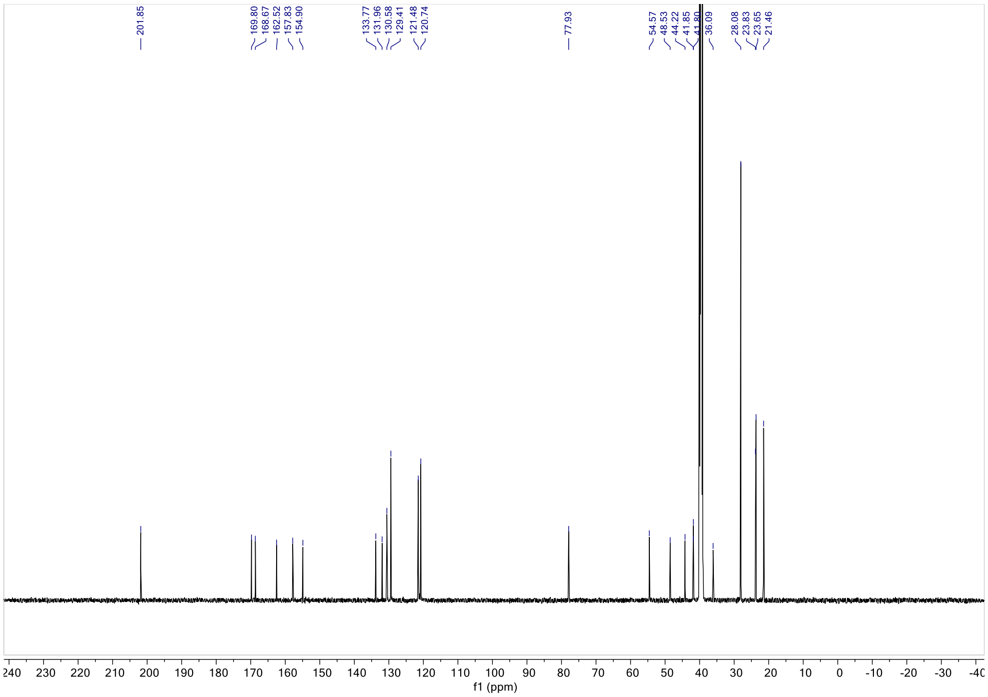

COSY in DMSO-d_6

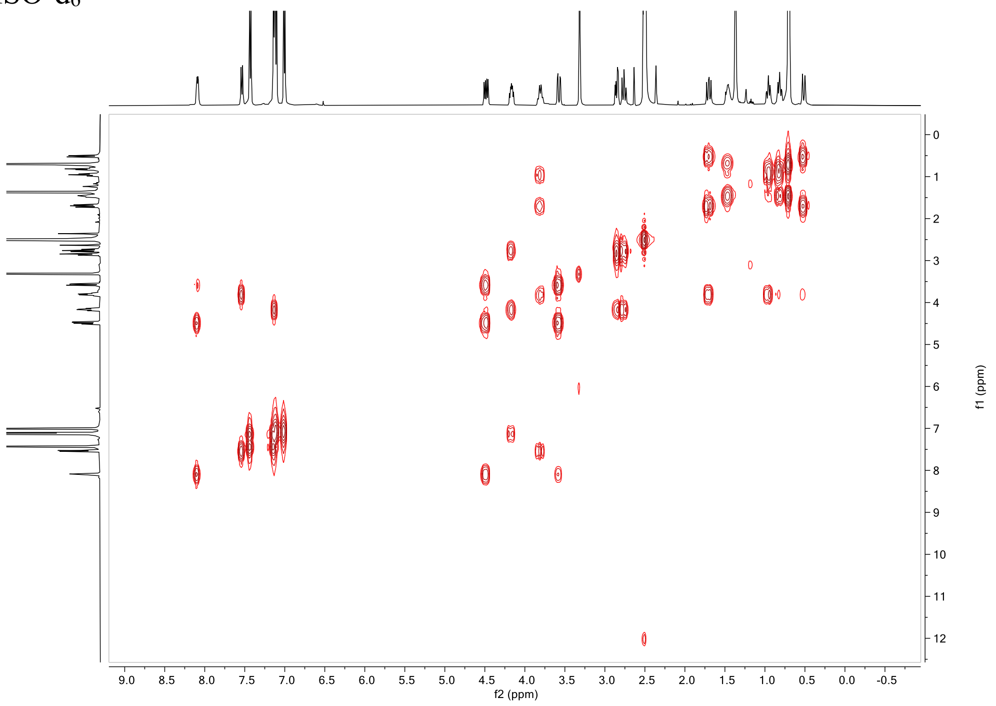

HSQC in DMSO-d_6

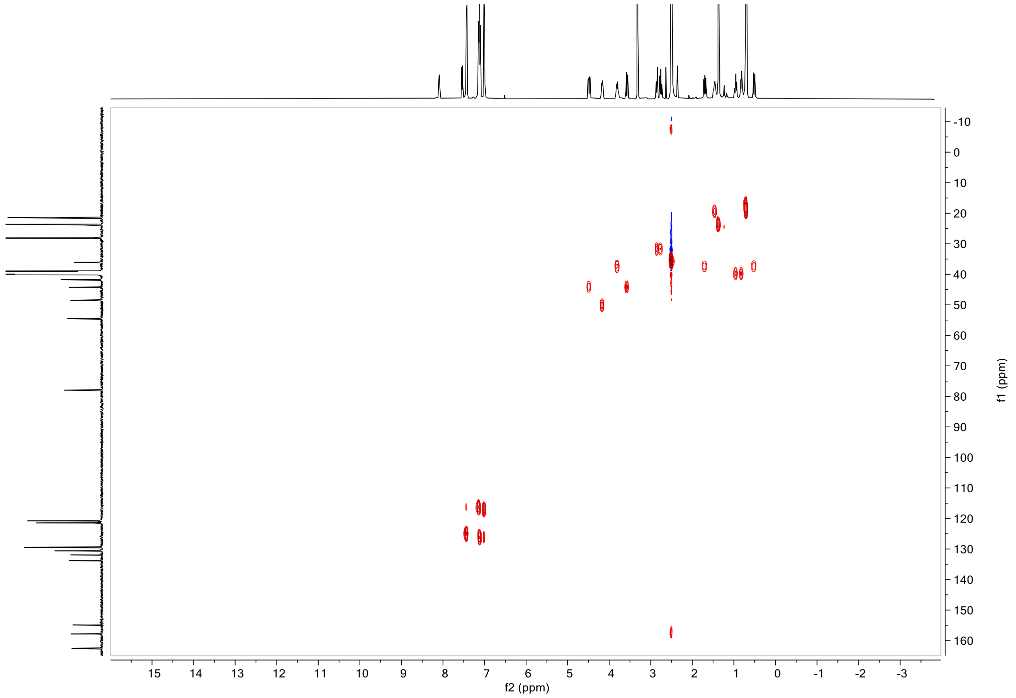

N-((8S,11S)-8-Isobutyl-4,7,10-trioxo-2-oxa-6,9-diaza-1,3(1,4)-dibenzenacyclododecaphane-11-yl)acetamide ***(1e)***.

^{1}H NMR in DMSO-d_6

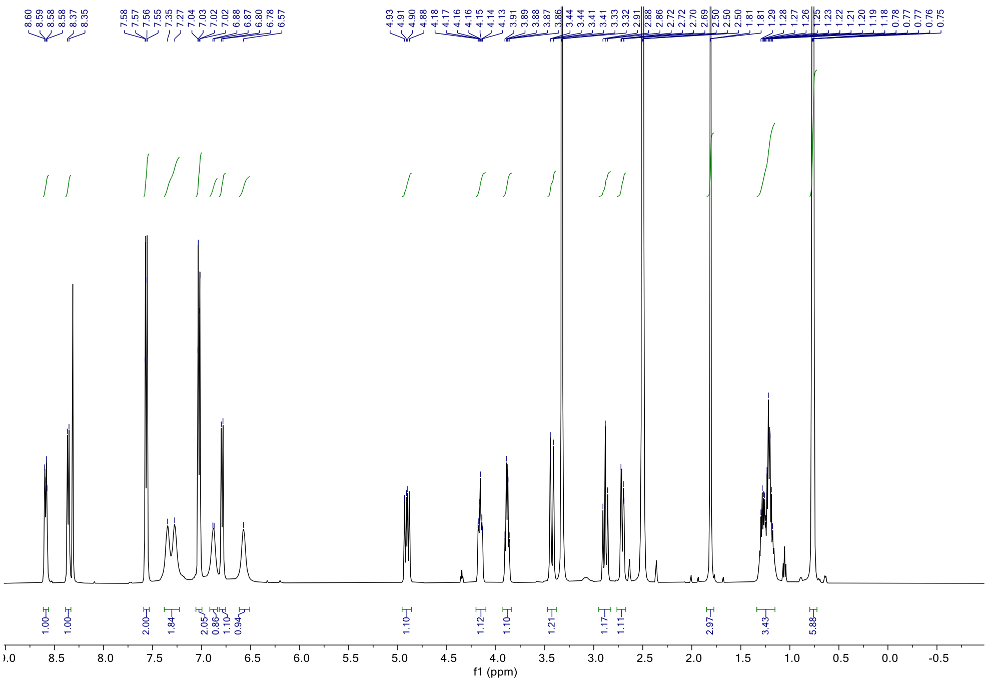

^{13}C NMR in DMSO-d_6

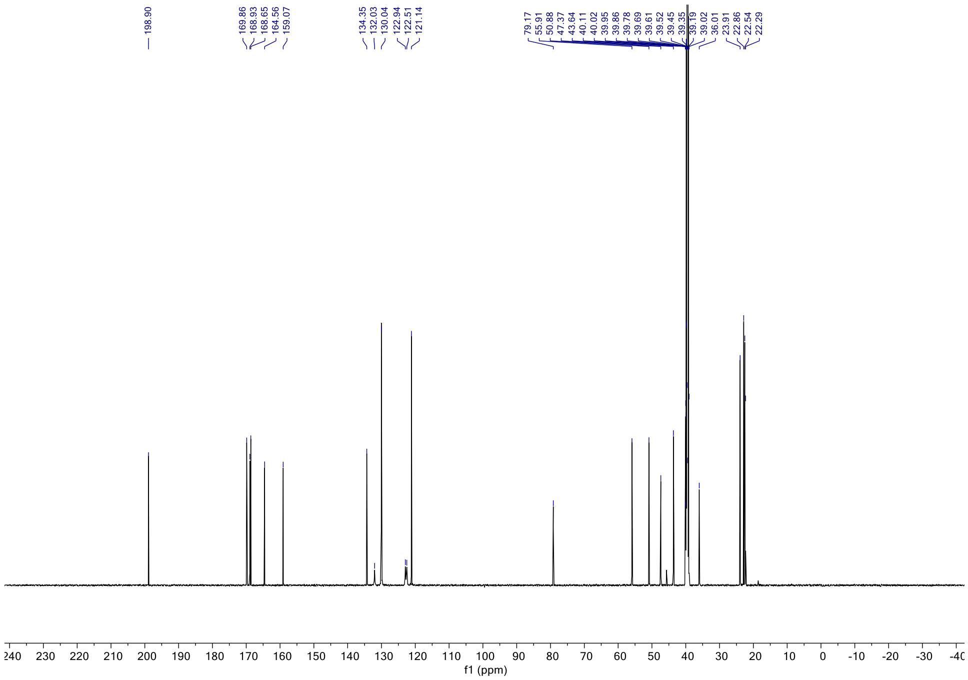

COSY in DMSO-d_6

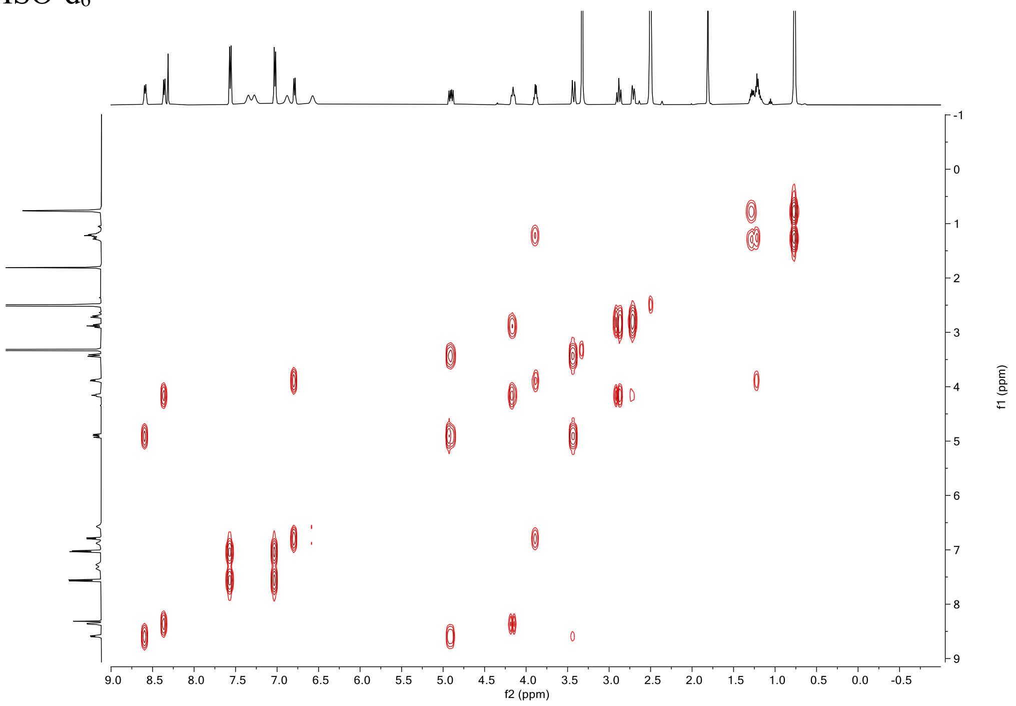

HSQC in DMSO-d_6

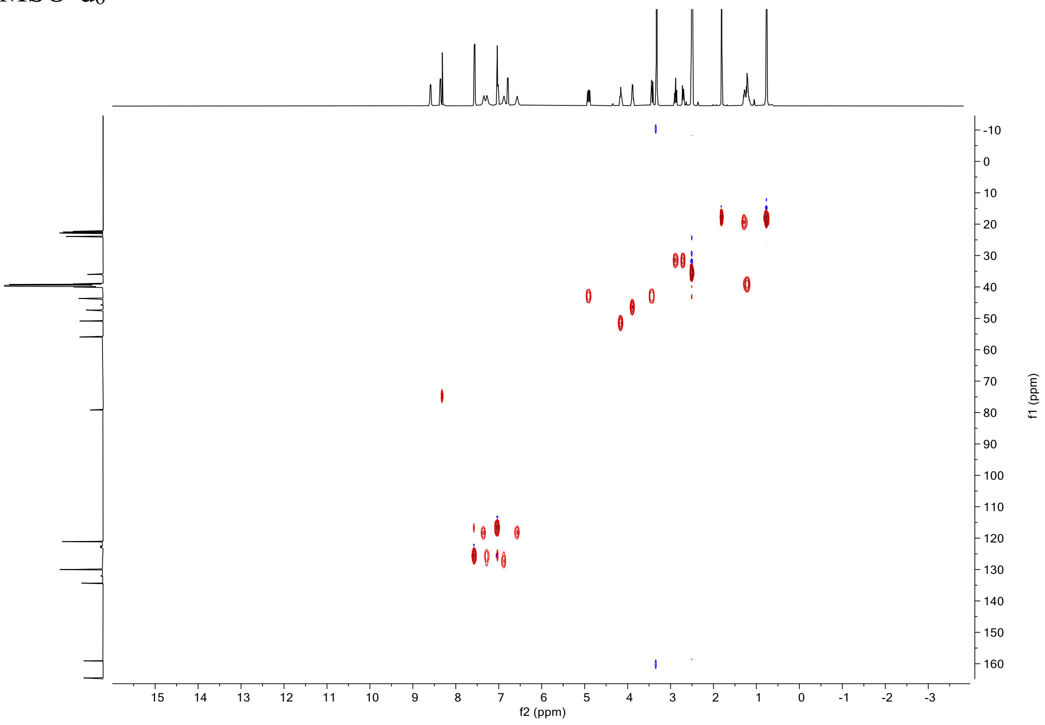

*N-((8S,11S)-8-(4-Methylbenzyl)-4,7,10-trioxo-2-oxa-6,9-diaza-1,3(1,4)-dibenzenacyclododecaphane-11-yl)acetamide (**1h**)*.

^{1}H NMR in DMSO-d_6

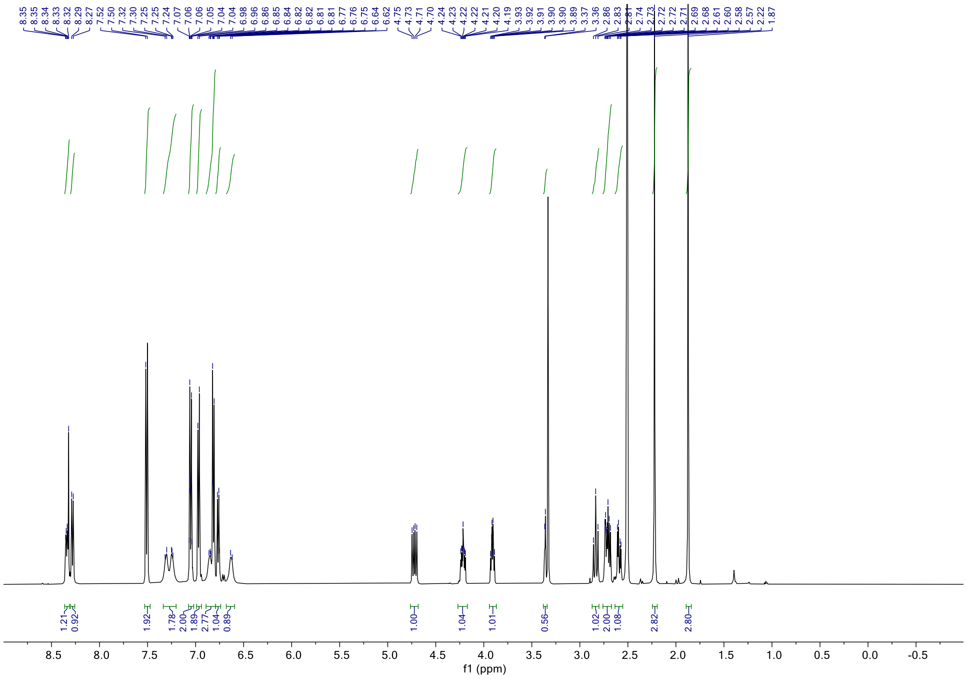

^{1}H NMR in DMSO-d_6 (expanded view)

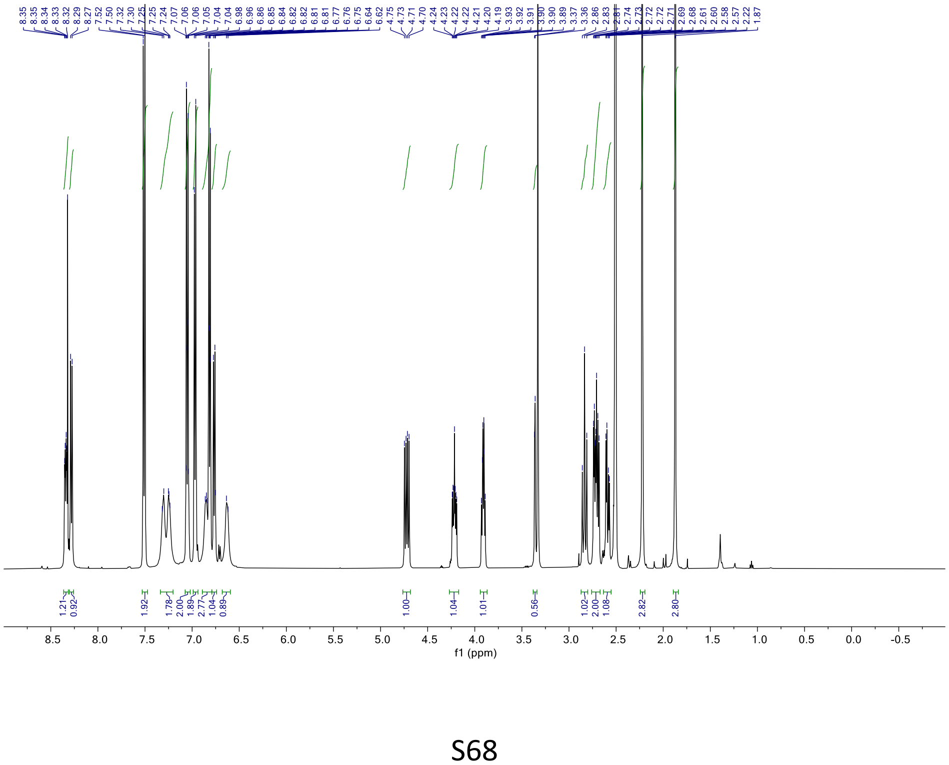

^{13}C NMR in DMSO-d$_6$

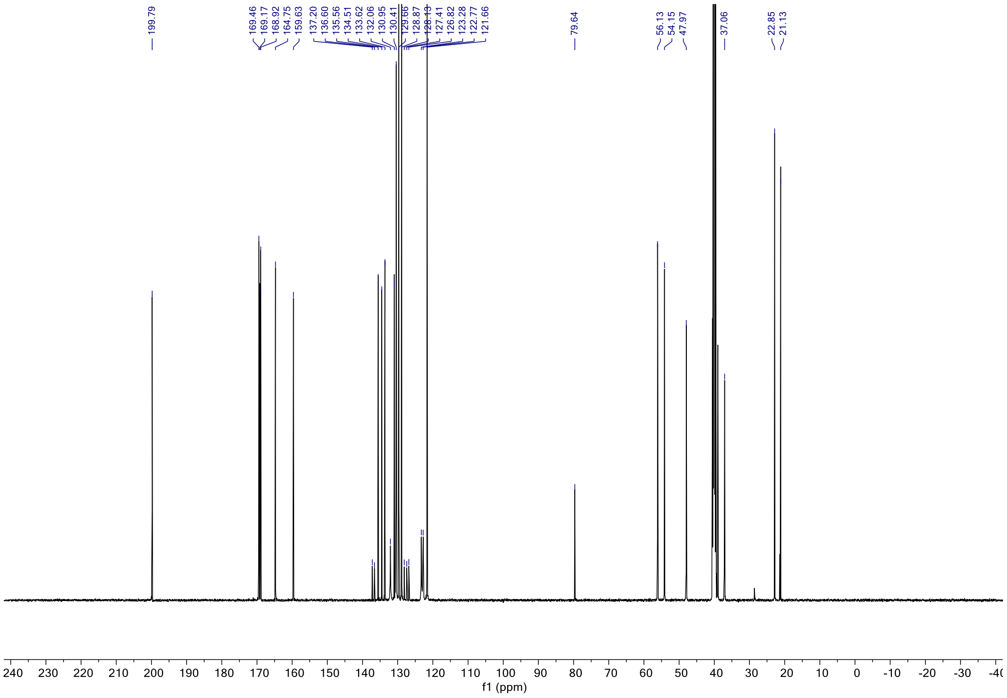

COSY in DMSO-d$_6$

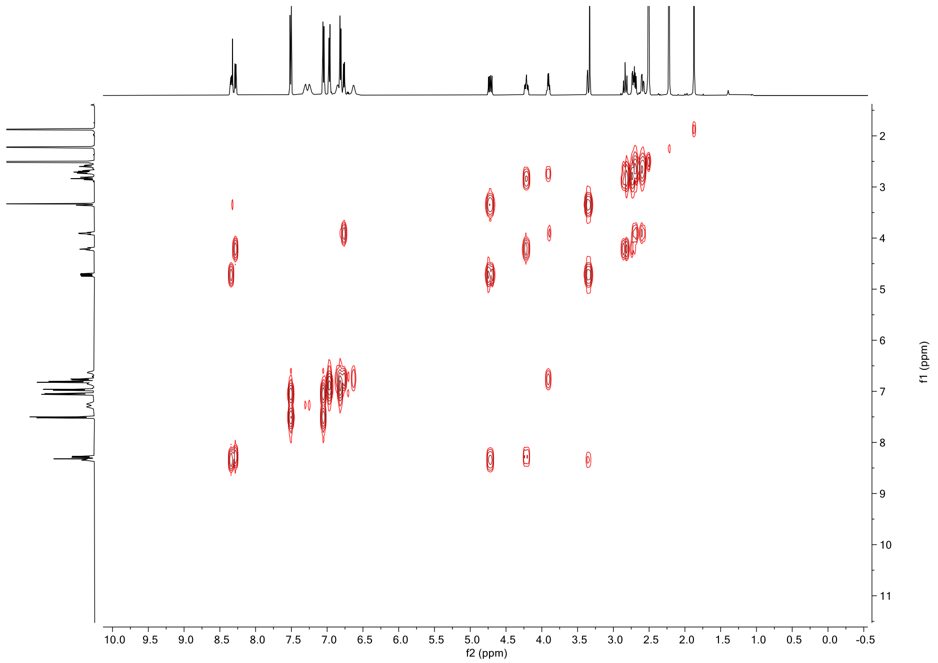

HSQC in DMSO-d6

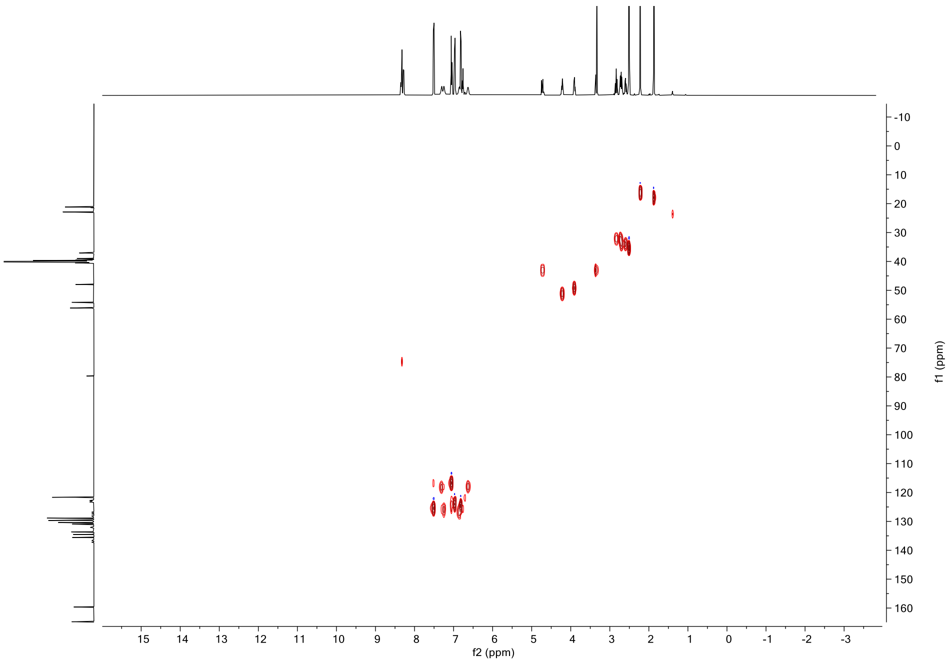

*N-((8S,11S)-8-(4-Fluorobenzyl)-4,7,10-trioxo-2-oxa-6,9-diaza-1,3(1,4)-dibenzenacyclododecaphane-11-yl)acetamide (**1i**)*.

^{1}H NMR in DMSO-d6

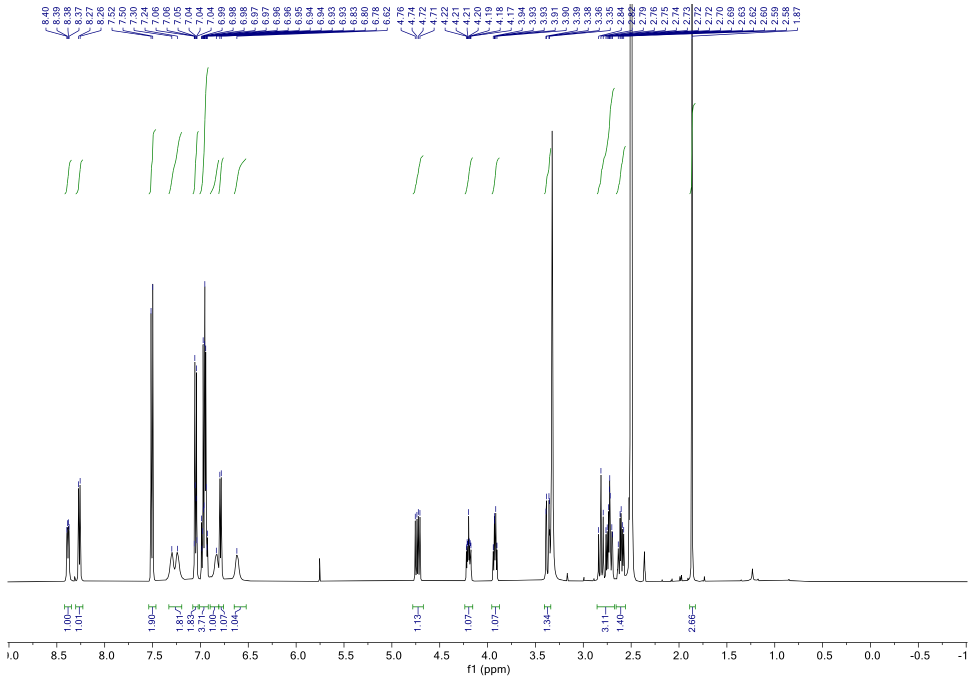

^{13}C NMR in DMSO-d_6

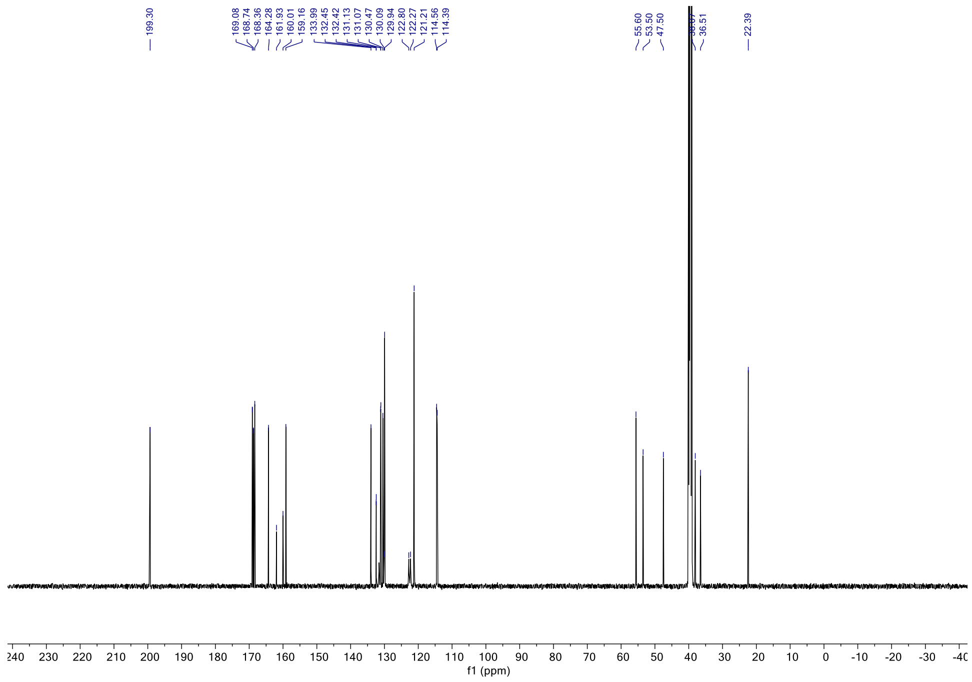

COSY in DMSO-d_6

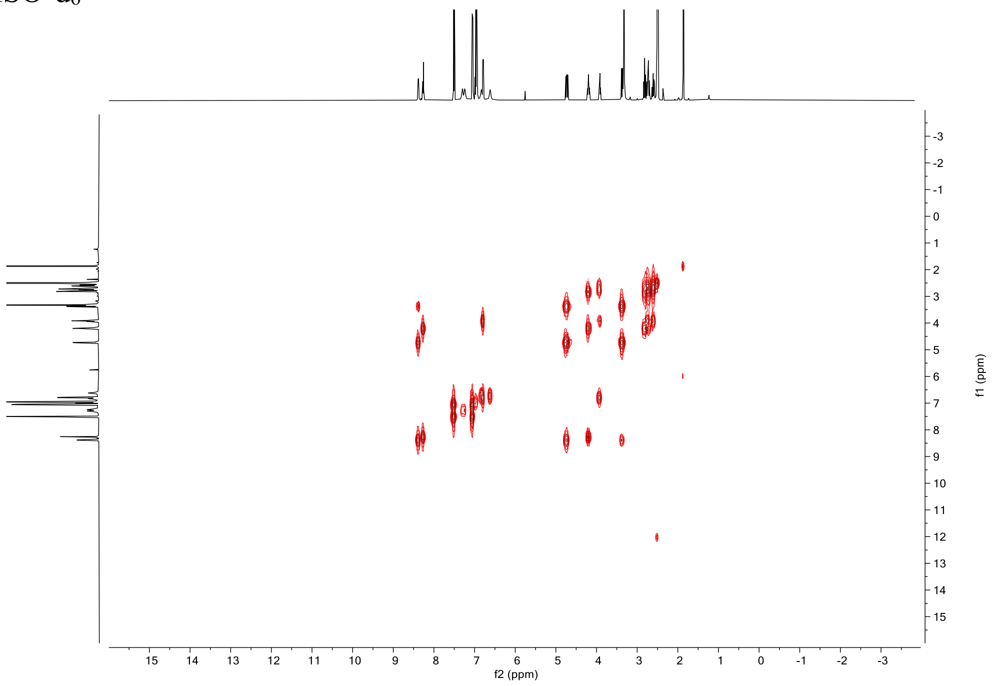

HSQC in DMSO-d$_6$

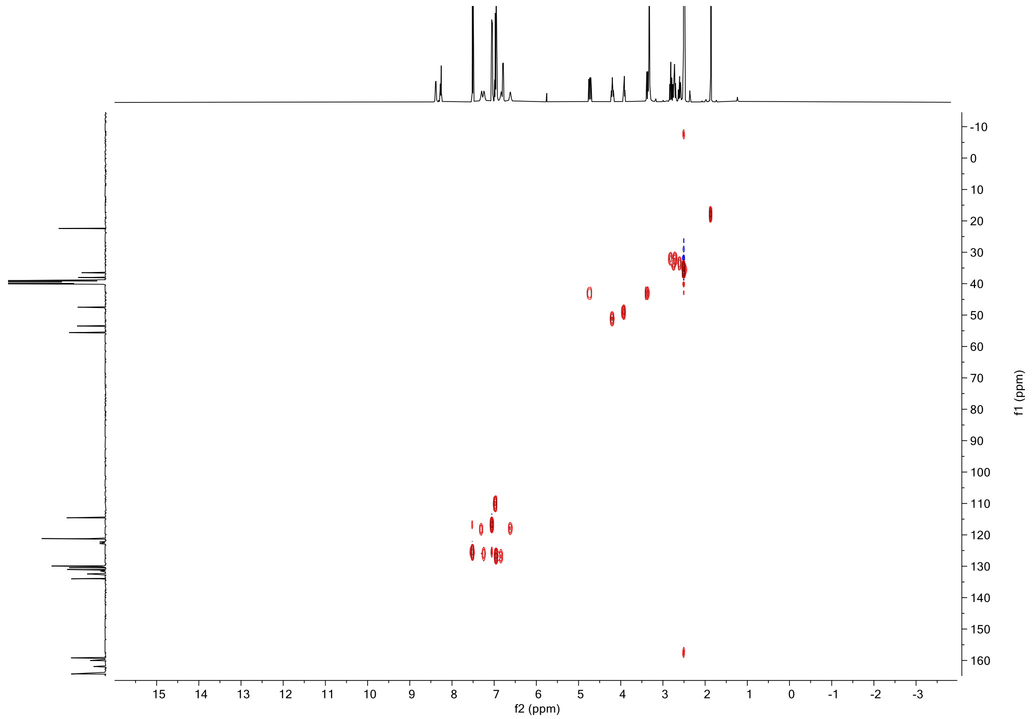

*N-((8S,11S)-8-Isobutyl-4,7,10-trioxo-2-oxa-6,9-diaza-1,3(1,4)-dibenzenacyclododecaphane-11-yl)propionamide (**1k**).*

^{1}H NMR in DMSO-d$_6$

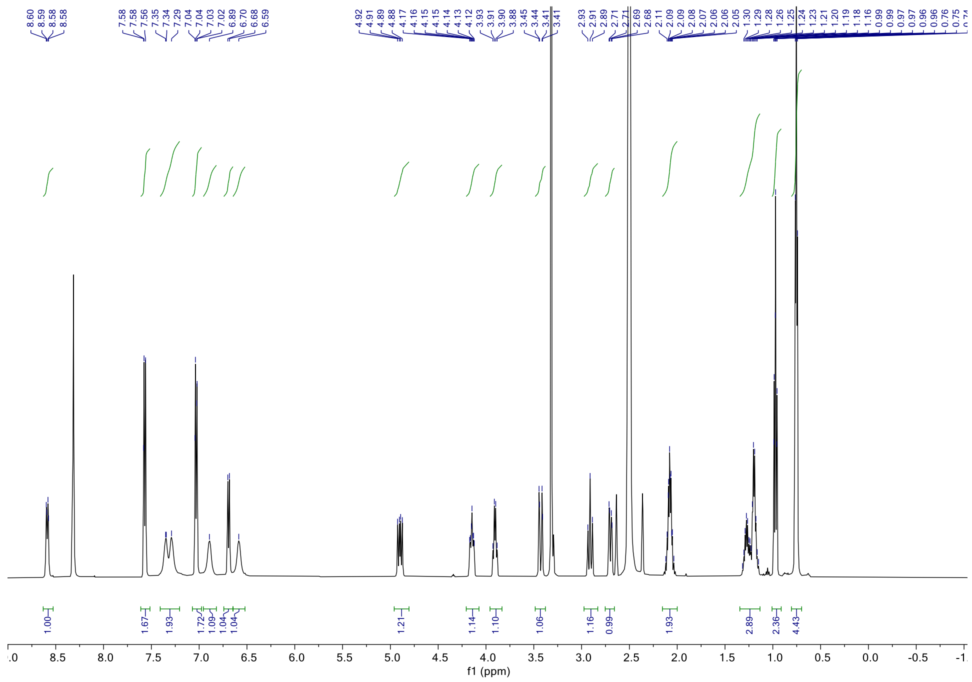

^{13}C NMR in DMSO-d_6

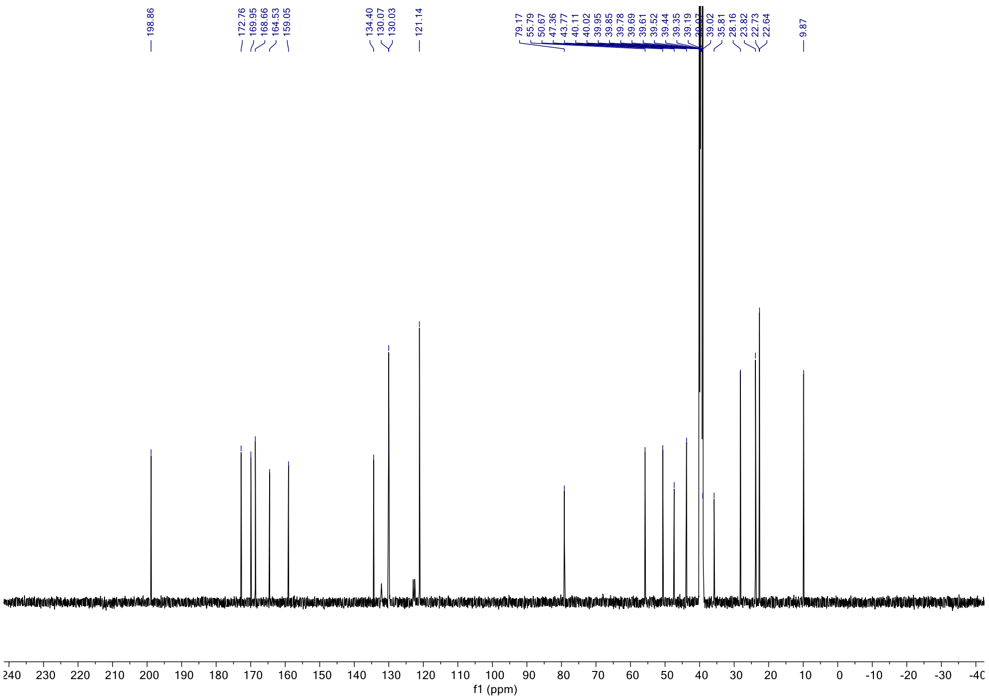

COSY in DMSO-d_6

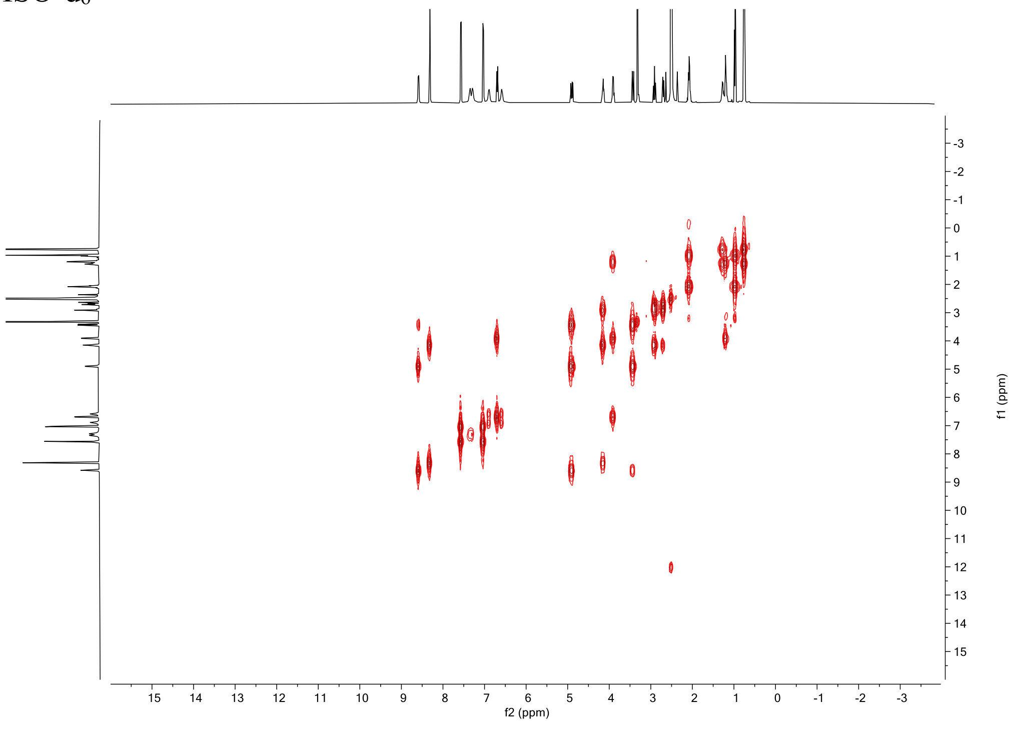

HSQC in DMSO-d$_6$

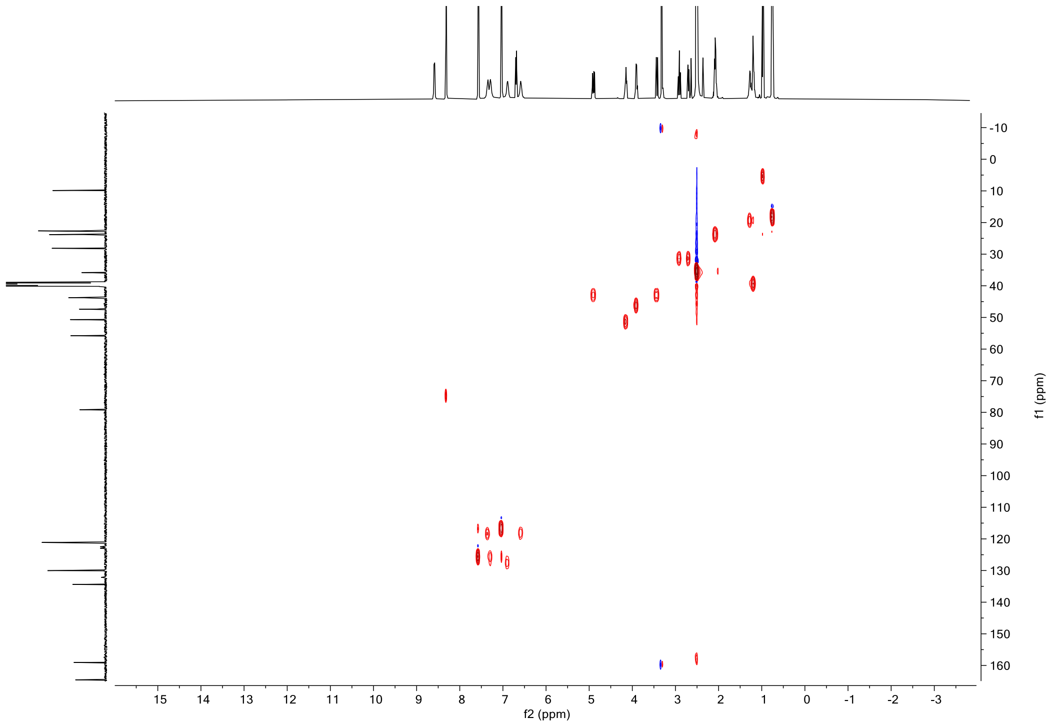

N-((8S,11S)-8-Benzyl-4,7,10-trioxo-2-oxa-6,9-diaza-1,3(1,4)-dibenzenacyclotridecaphane-11-yl)acetamide ***(2c)***.

^{1}H NMR in DMSO-d$_6$

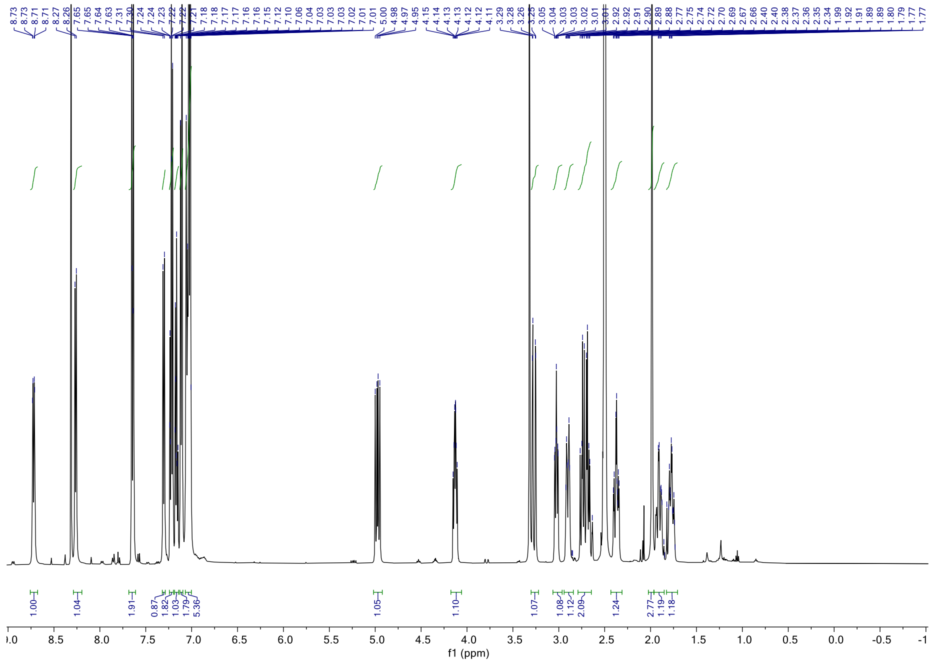

^{13}C NMR in DMSO-d_6

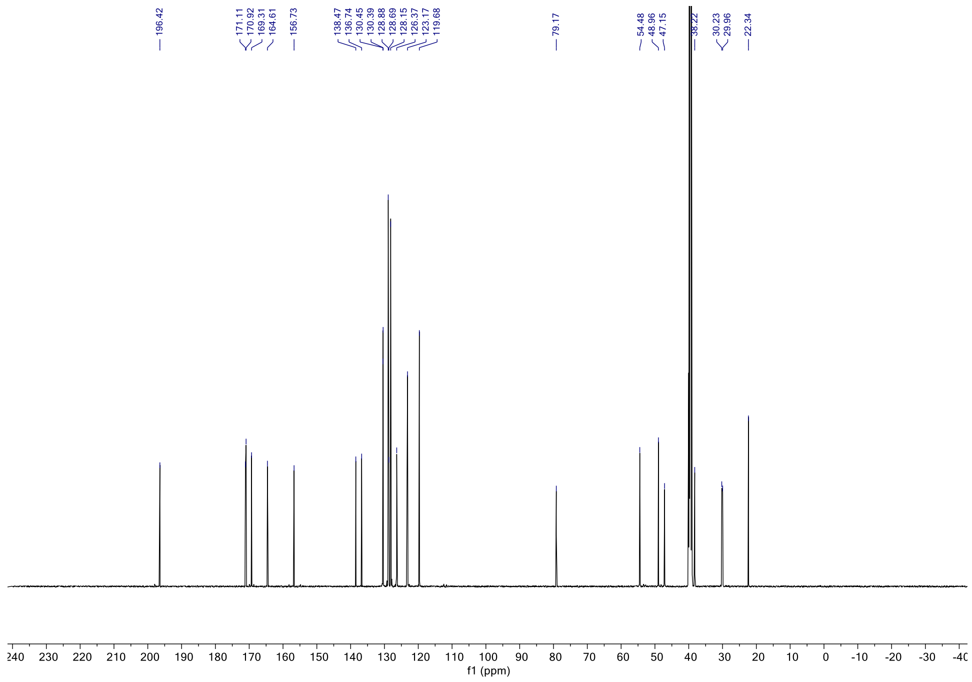

COSY in DMSO-d_6

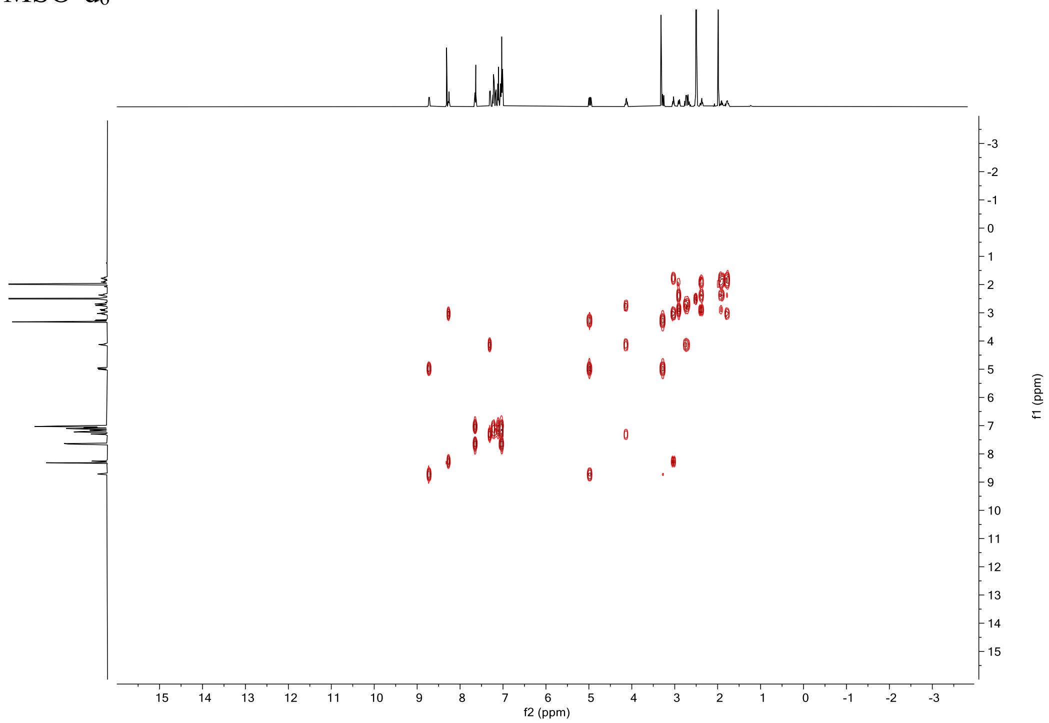

HSQC in DMSO-d_6

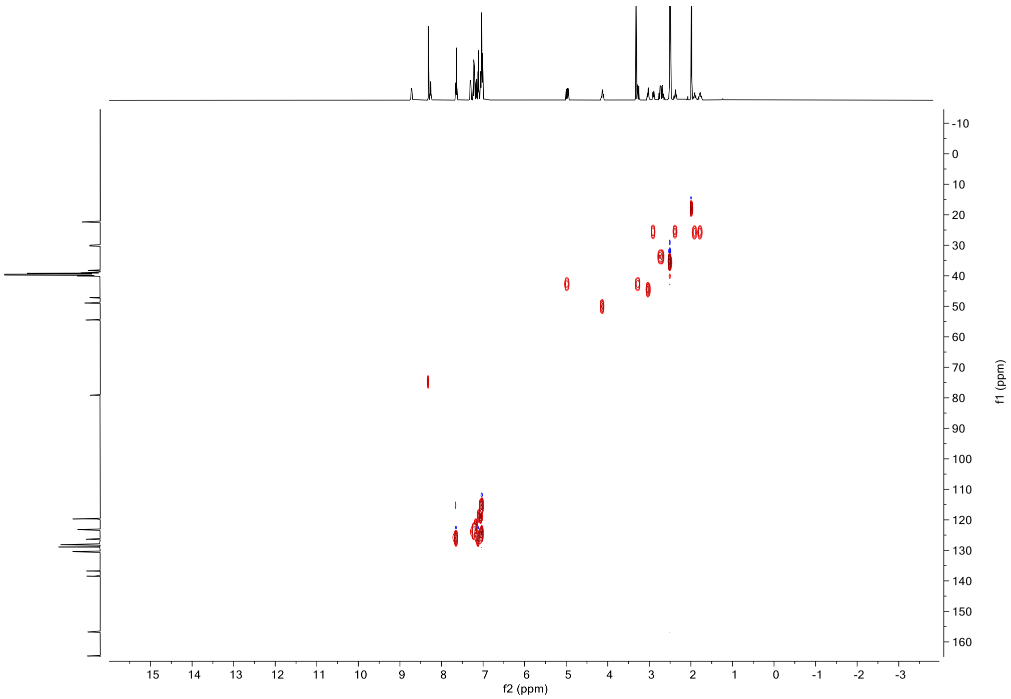

N-((9S,12S)-9-Benzyl-4,7,11-trioxo-2-oxa-6,10-diaza-1,3(1,4)-dibenzenacyclotridecaphane-12-yl)acetamide ***(3c)***.

^{1}H NMR in DMSO-d_6

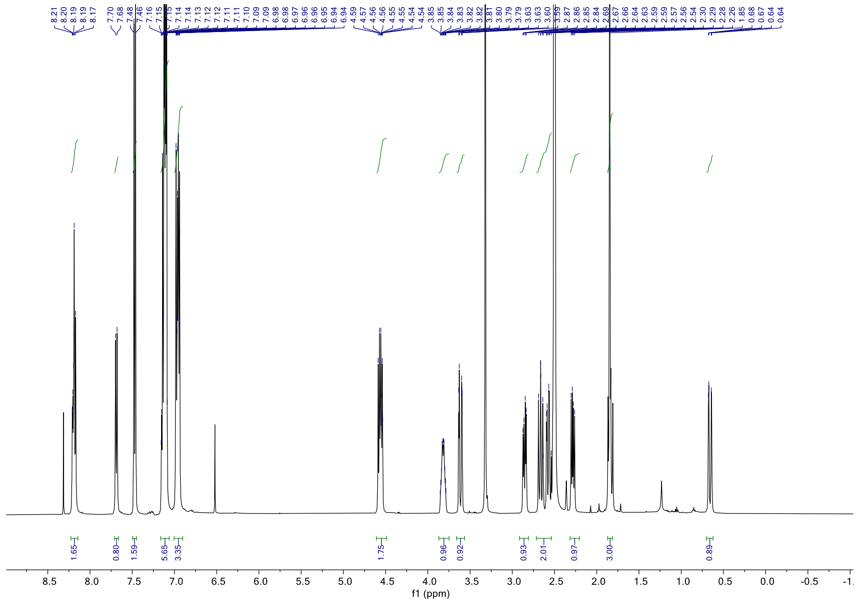

^{13}C NMR in DMSO-d$_6$

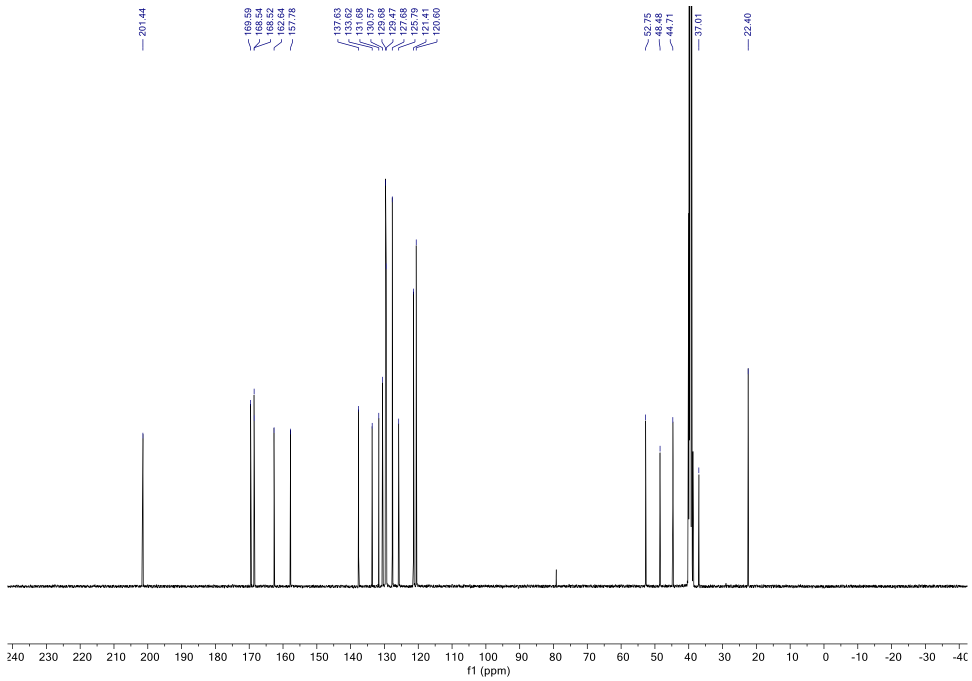

COSY in DMSO-d$_6$

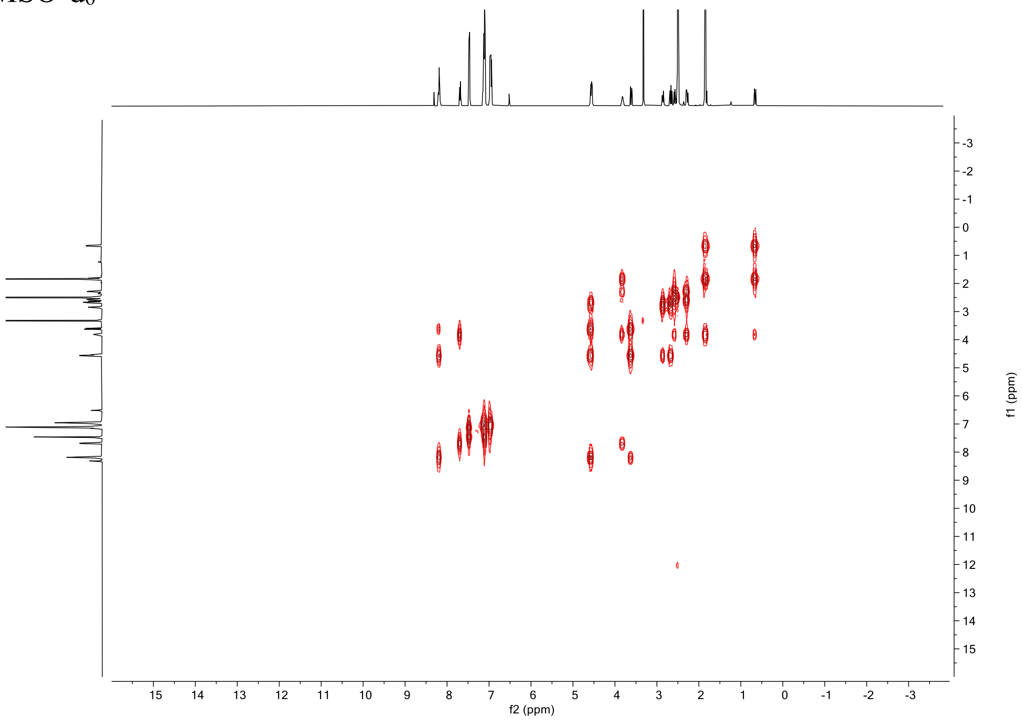

HSQC in DMSO-d$_6$

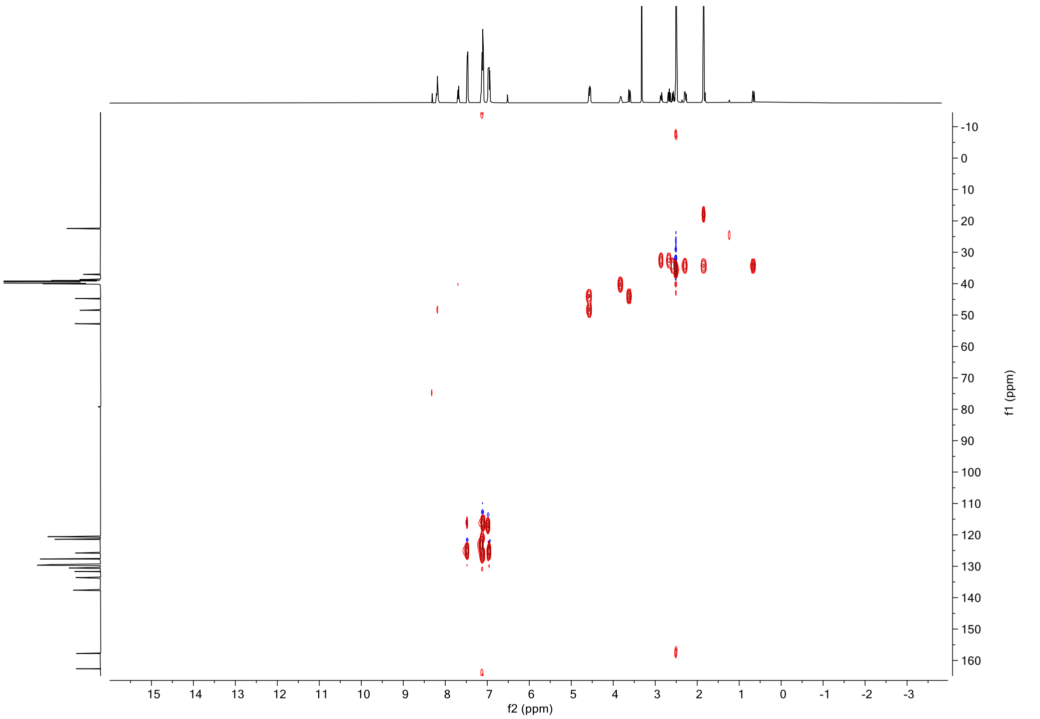

*N-((9S,12S)-9-Isobutyl-4,7,11-trioxo-2-oxa-6,10-diaza-1,3(1,4)-dibenzenacyclotridecaphane-12-yl)acetamide (**3e**).*

^{1}H NMR in DMSO-d$_6$

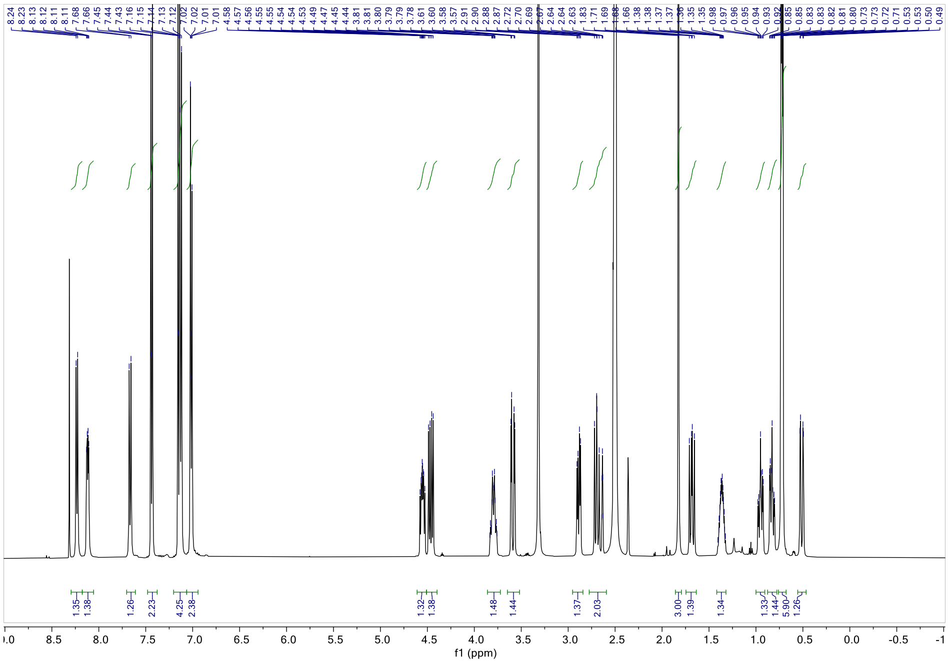

^{13}C NMR in DMSO-d_6

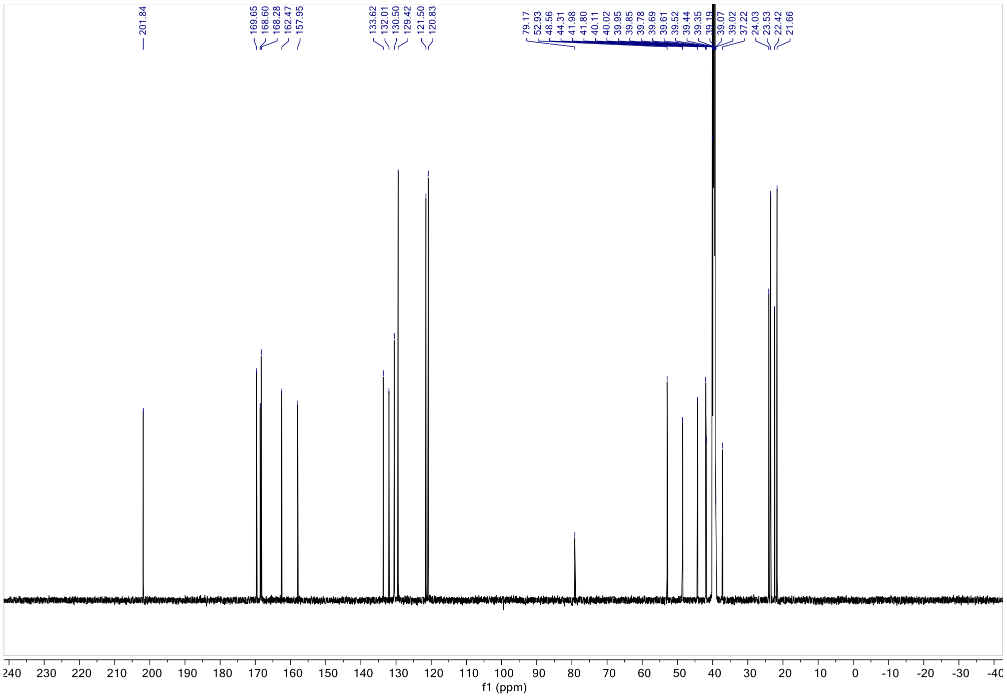

COSY in DMSO-d_6

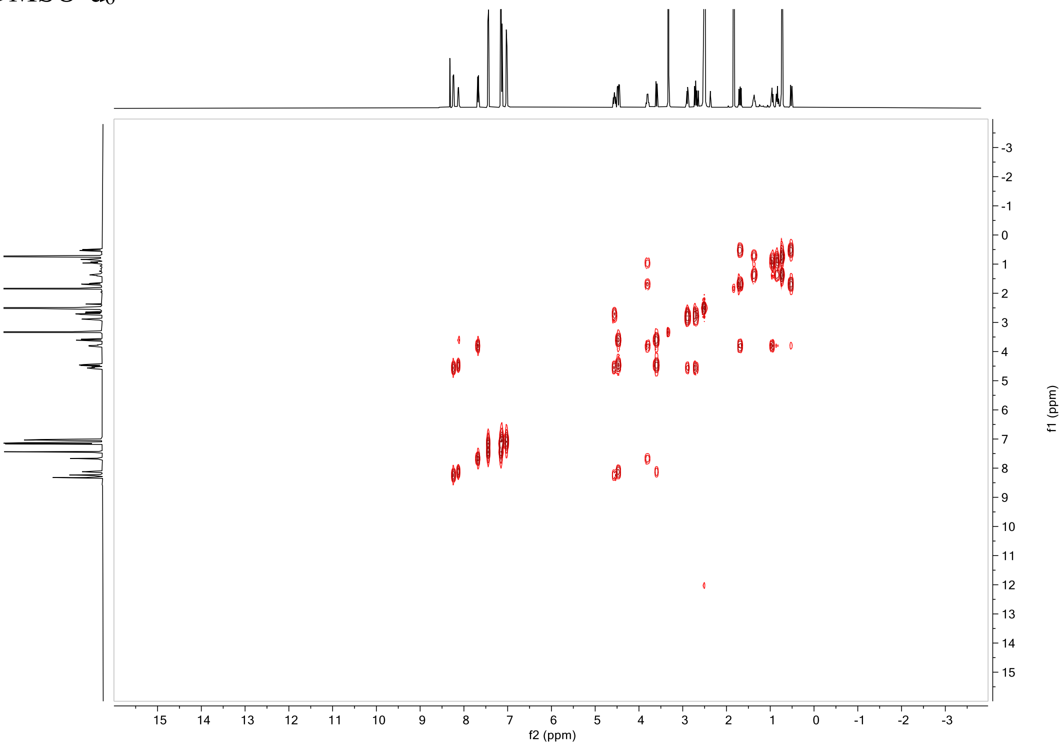

HSQC in DMSO-d$_6$

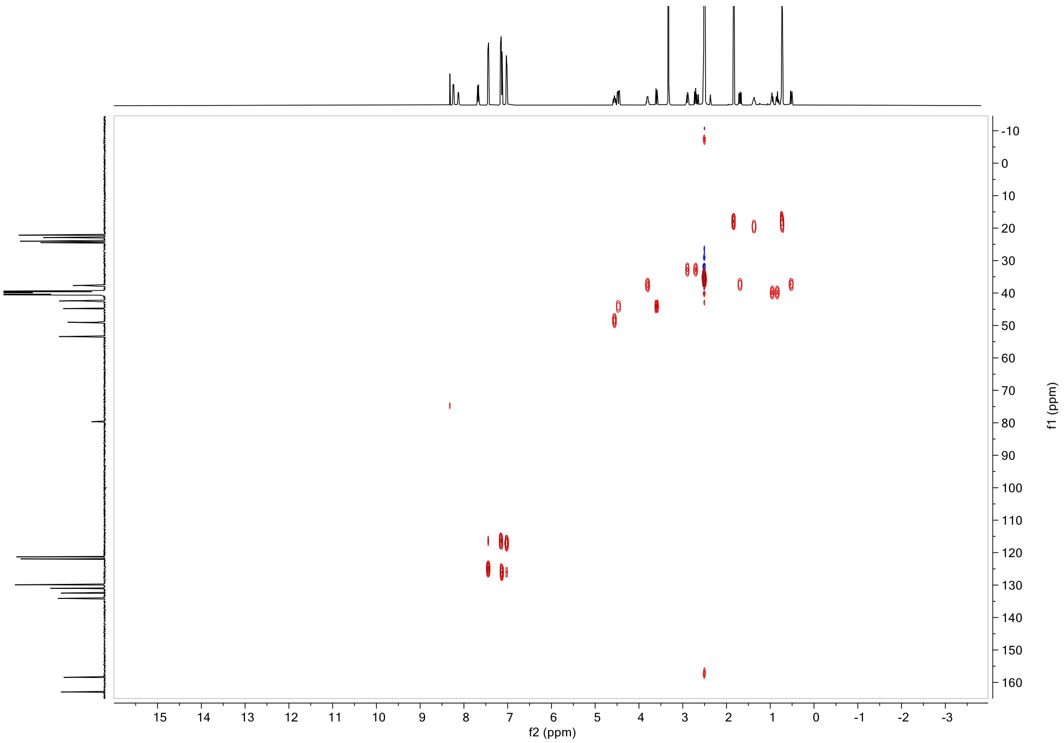

*(8S,11S)-11-(Dimethylamino)-8-isopentyl-2-oxa-6,9-diaza-1,3(1,4)-dibenzenacyclododecaphane-4,7,10-trione (**4a**).*

^{1}H NMR in $CDCl_3$

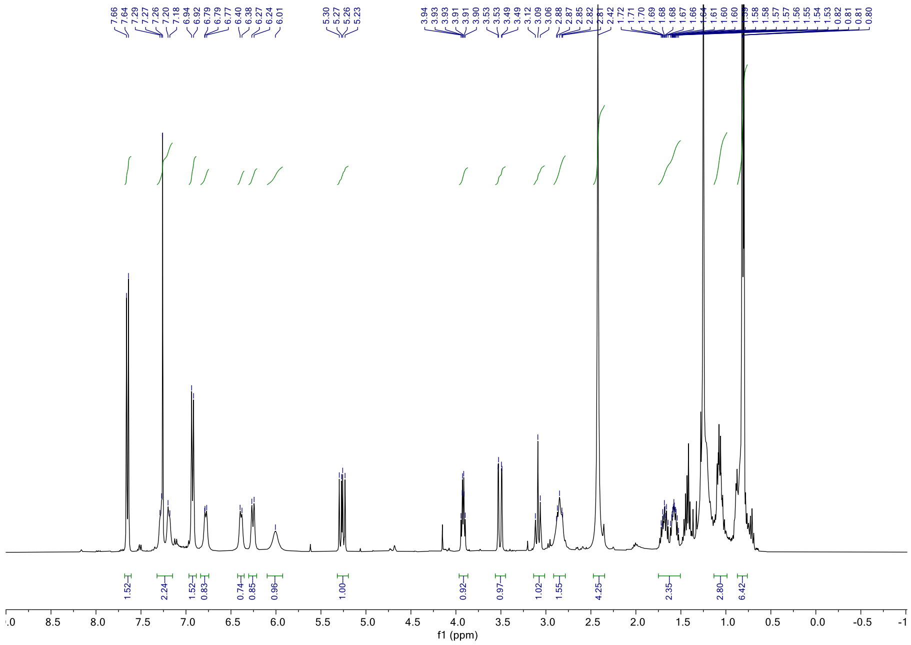

^{13}C NMR in $CDCl_3$

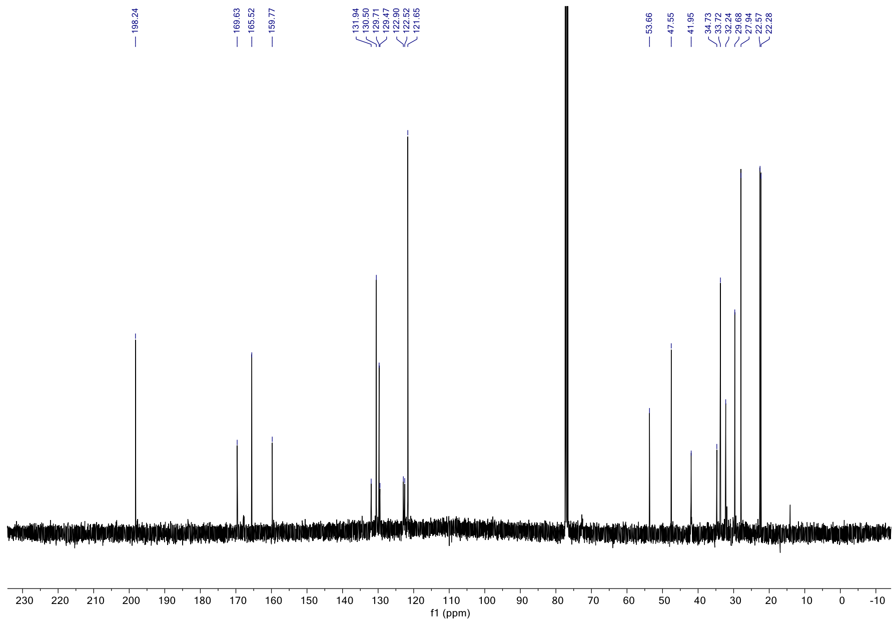

COSY in $CDCl_3$

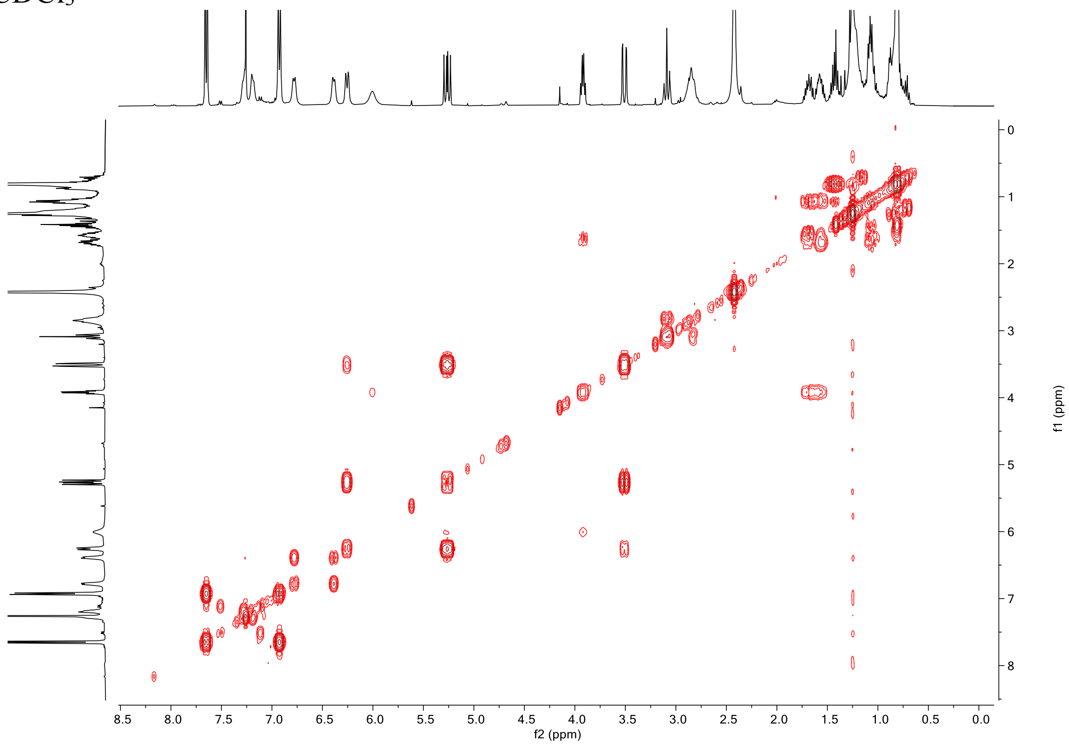

HSQC in $CDCl_3$

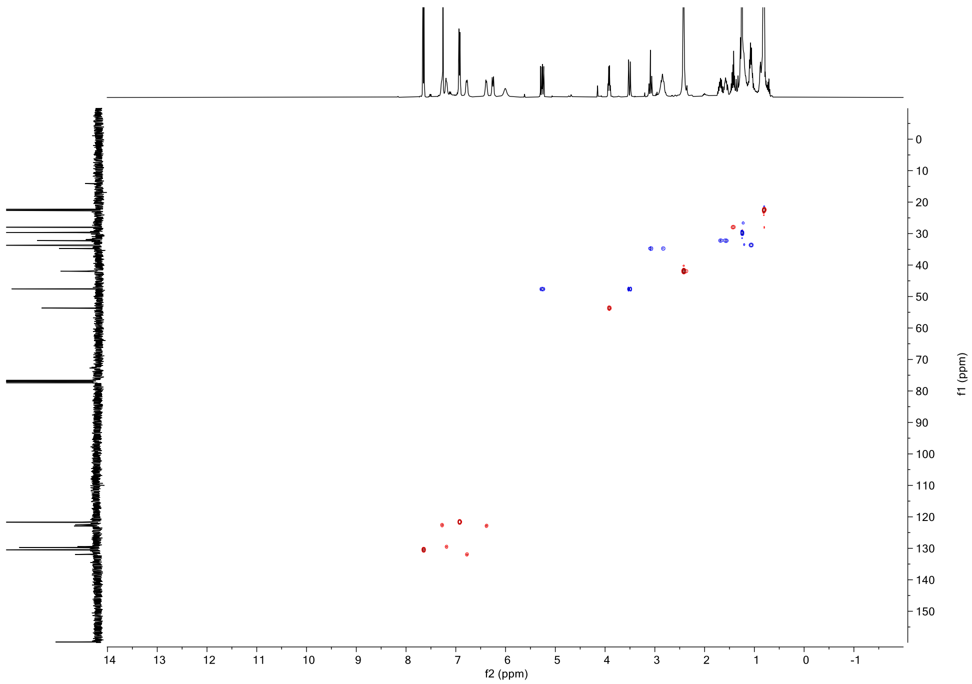

(8S,11S)-11-(Dimethylamino)-8-phenethyl-2-oxa-6,9-diaza-1,3(1,4)-dibenzenacyclododecaphane-4,7,10-trione ***(4b)***.

^{1}H NMR in $CDCl_3$:

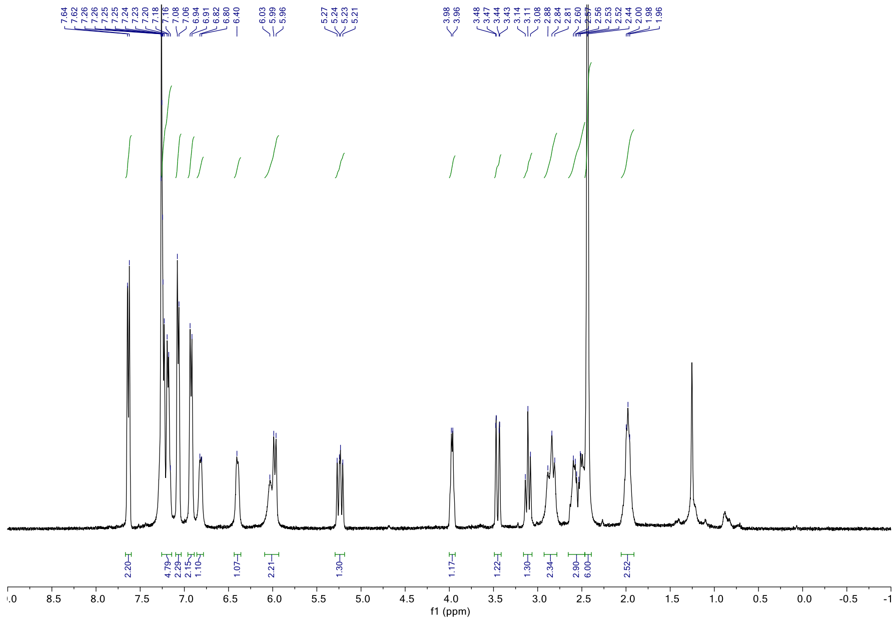

^{13}C NMR in $CDCl_3$

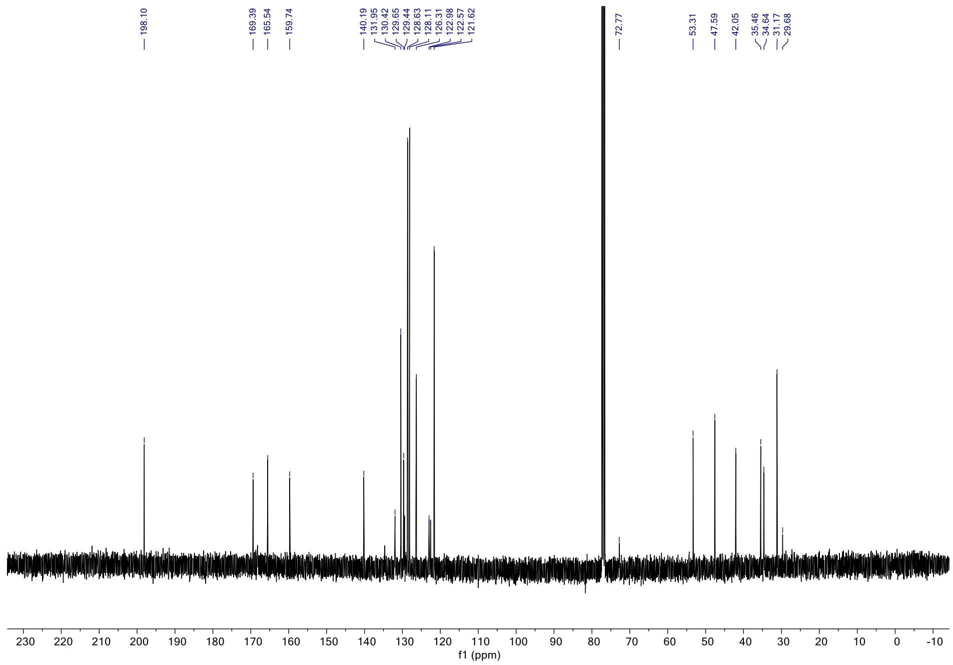

COSY in $CDCl_3$

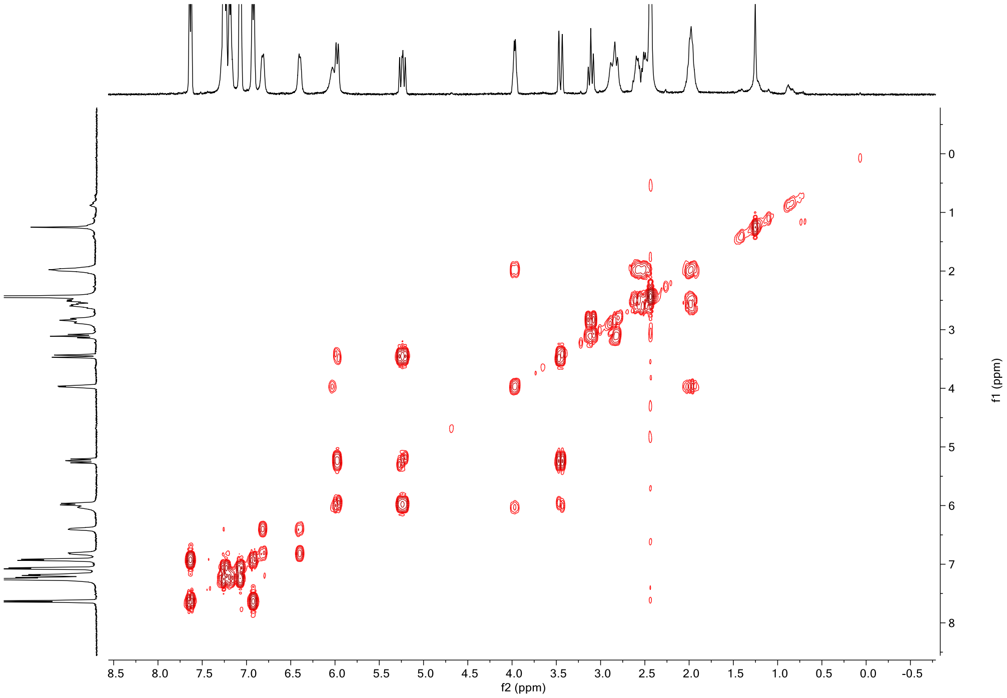

HSQC in $CDCl_3$

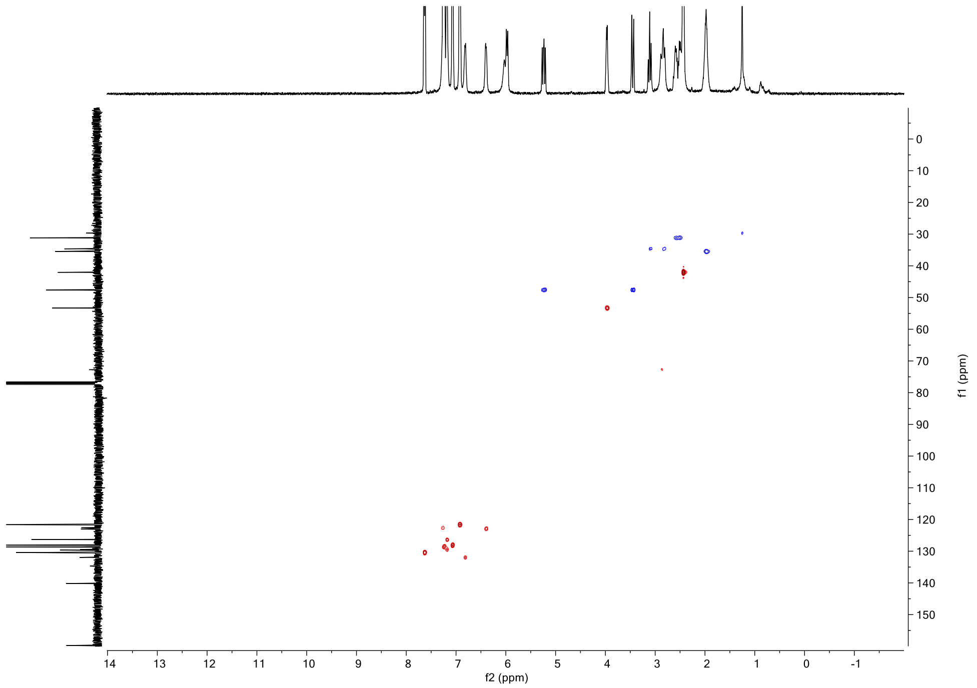

(8S,11S)-8-Benzyl-11-(dimethylamino)-2-oxa-6,9-diaza-1,3(1,4)-dibenzenacyclododecaphane-4,7,10-trione ***(4c)***.

^{1}H NMR in $CDCl_3$

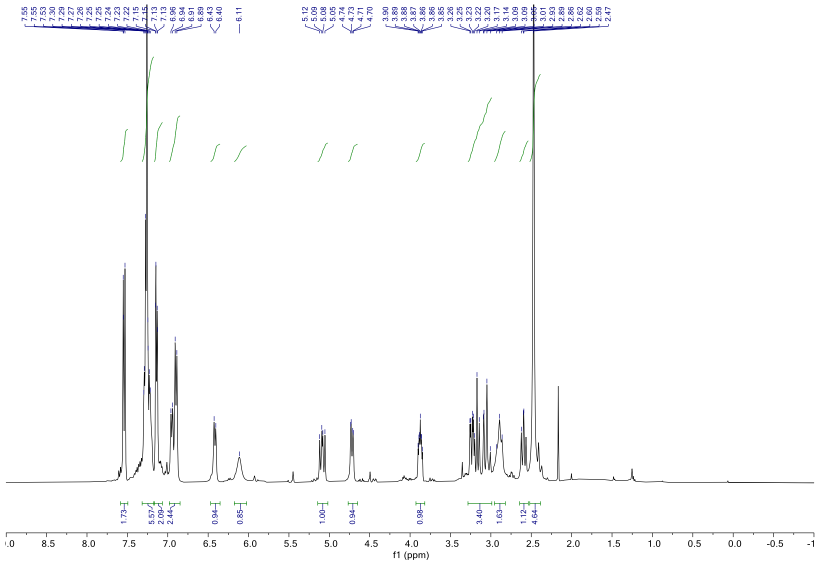

^{13}C NMR in $CDCl_3$

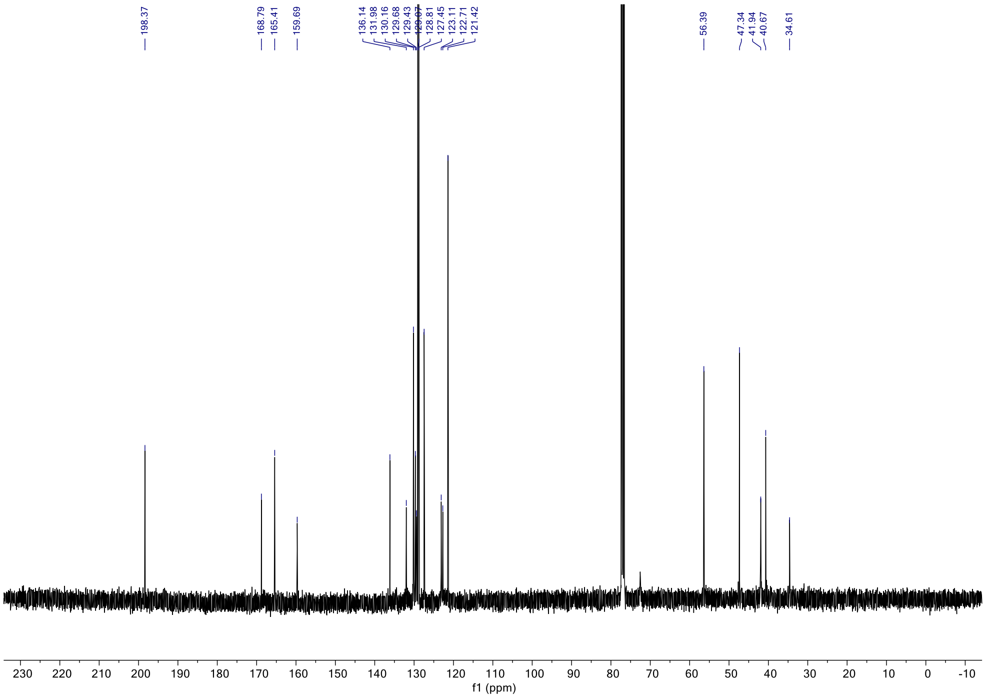

COSY in CDCl$_3$

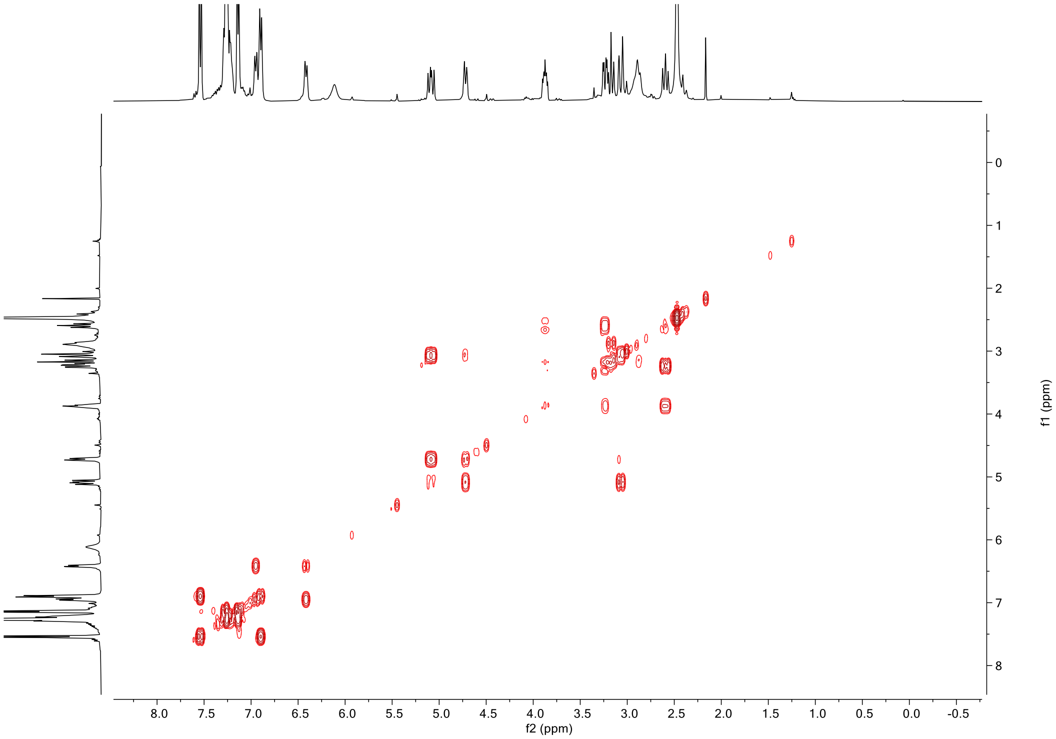

HSQC in CDCl$_3$

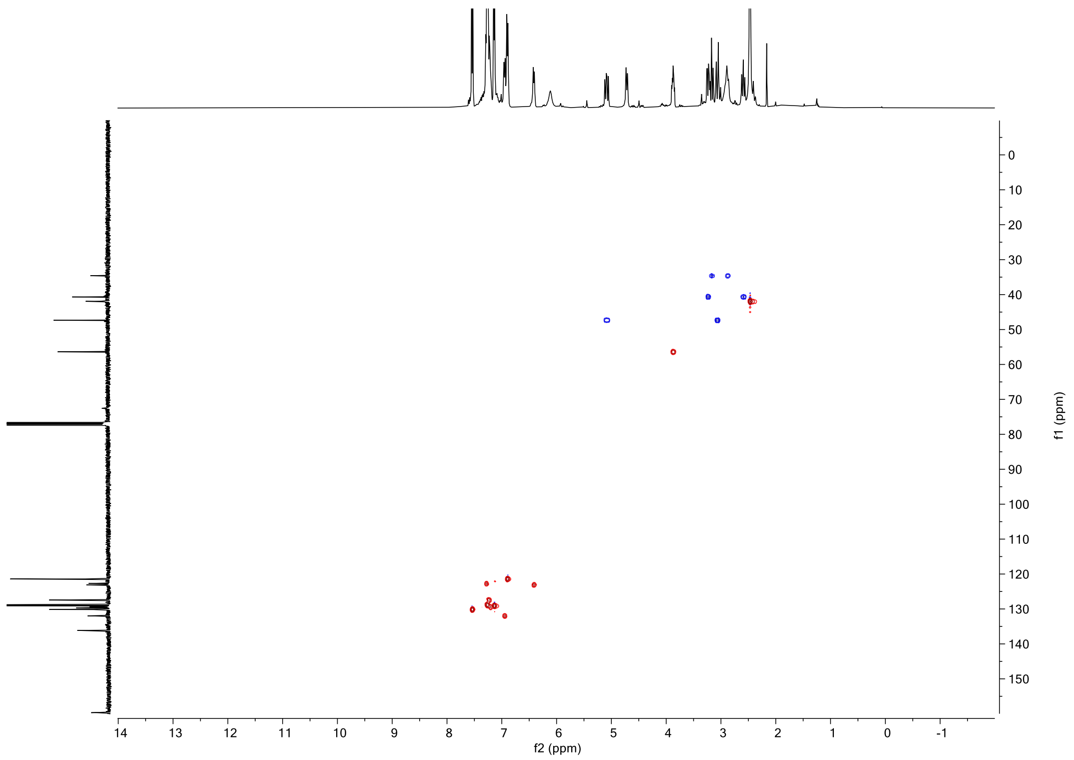

*(8S,11S)-11-(Dimethylamino)-8-isobutyl-2-oxa-6,9-diaza-1,3(1,4)-dibenzenacyclododecaphane-4,7,10-trione **(4e)***.

^{1}H NMR in $CDCl_3$

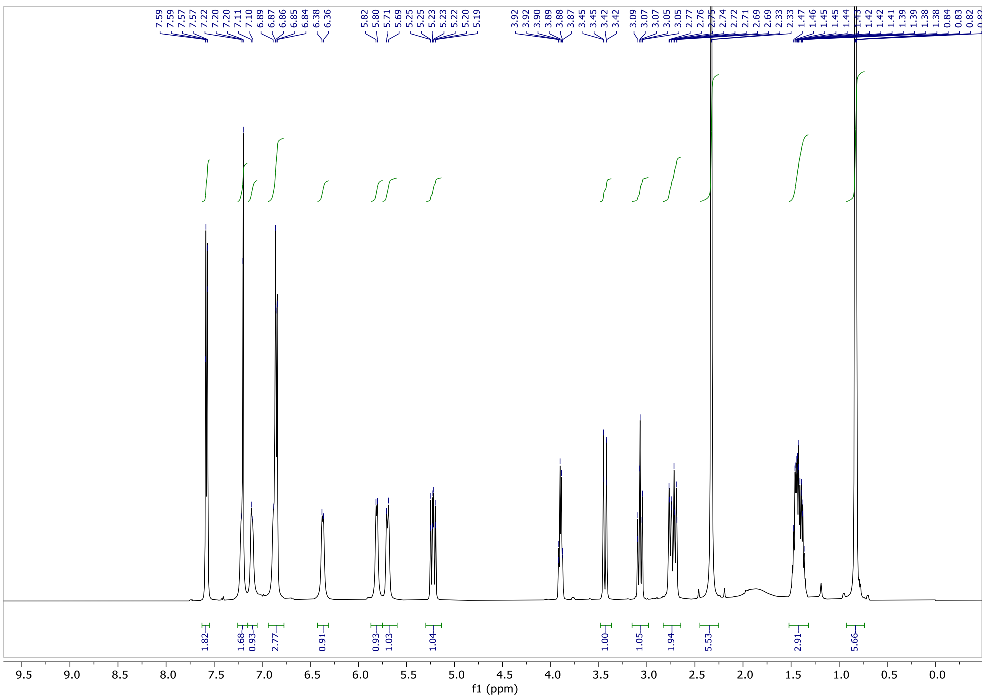

^{13}C NMR in $CDCl_3$

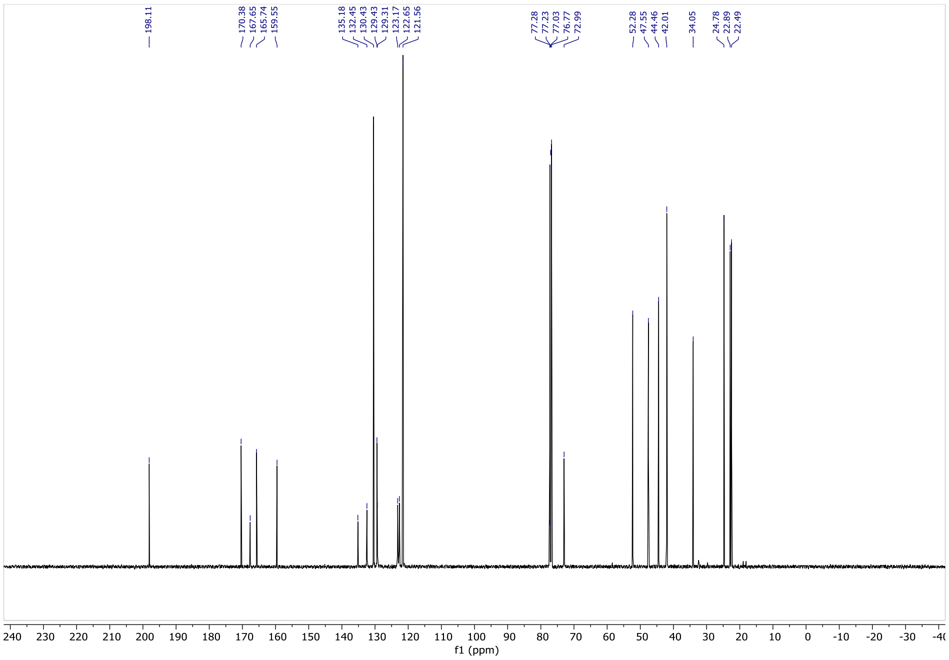

COSY in $CDCl_3$

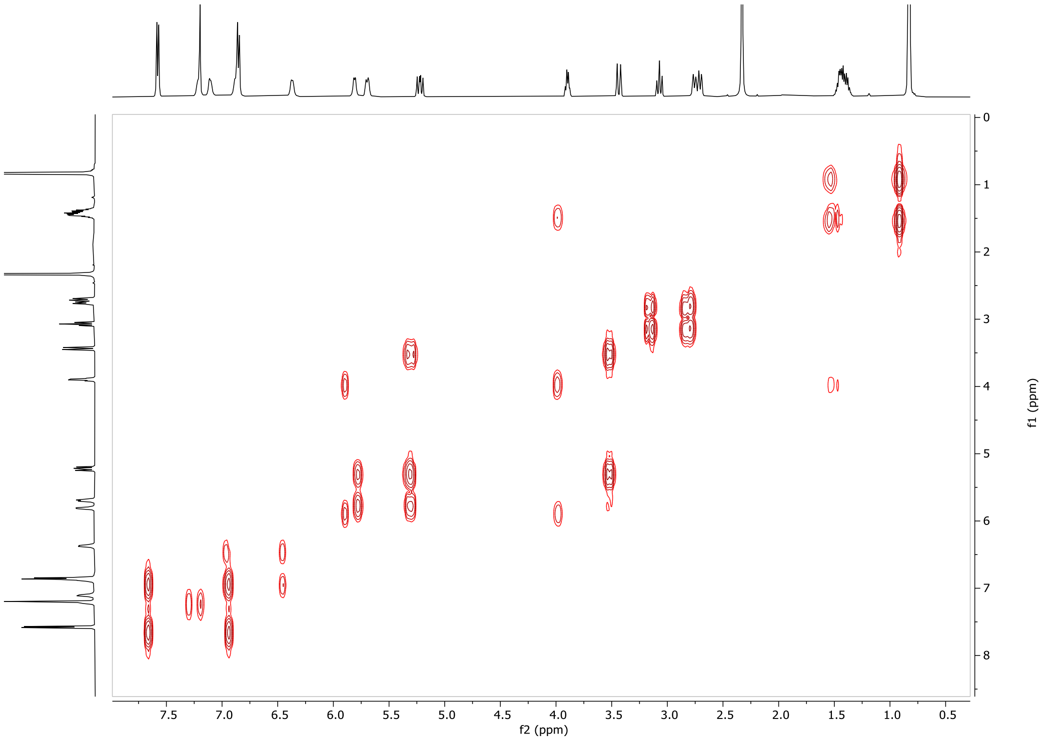

HSQC in $CDCl_3$

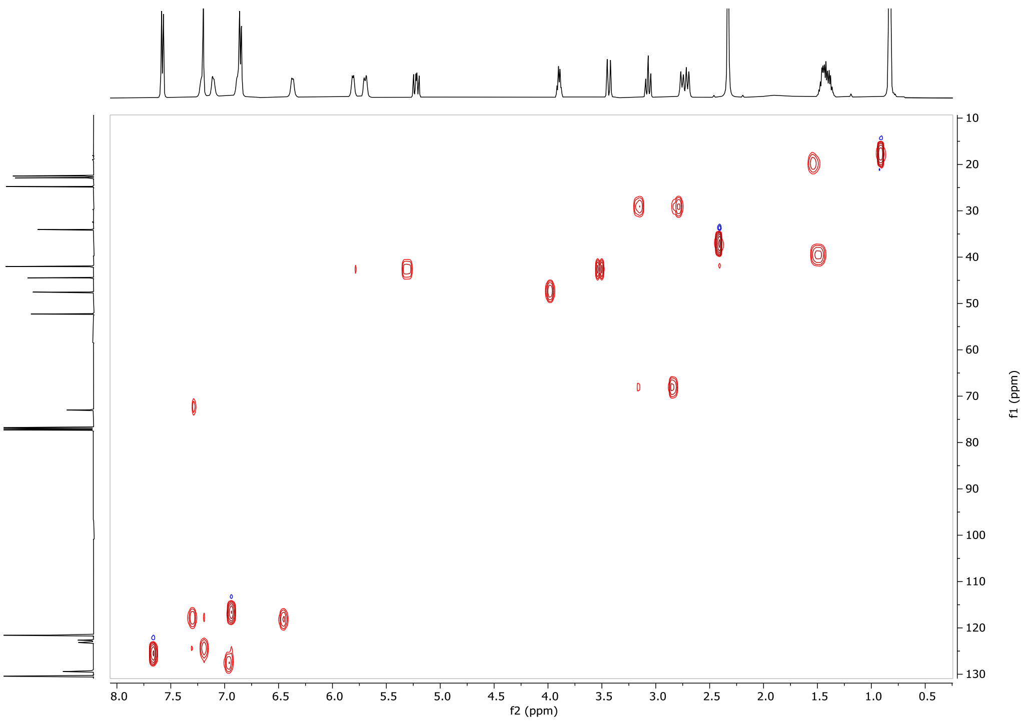

*(8S,11S)-11-(Dimethylamino)-8-phenyl-2-oxa-6,9-diaza-1,3(1,4)-dibenzenacyclododecaphane-4,7,10-trione **(4j)***.

^{1}H NMR in $CDCl_3$

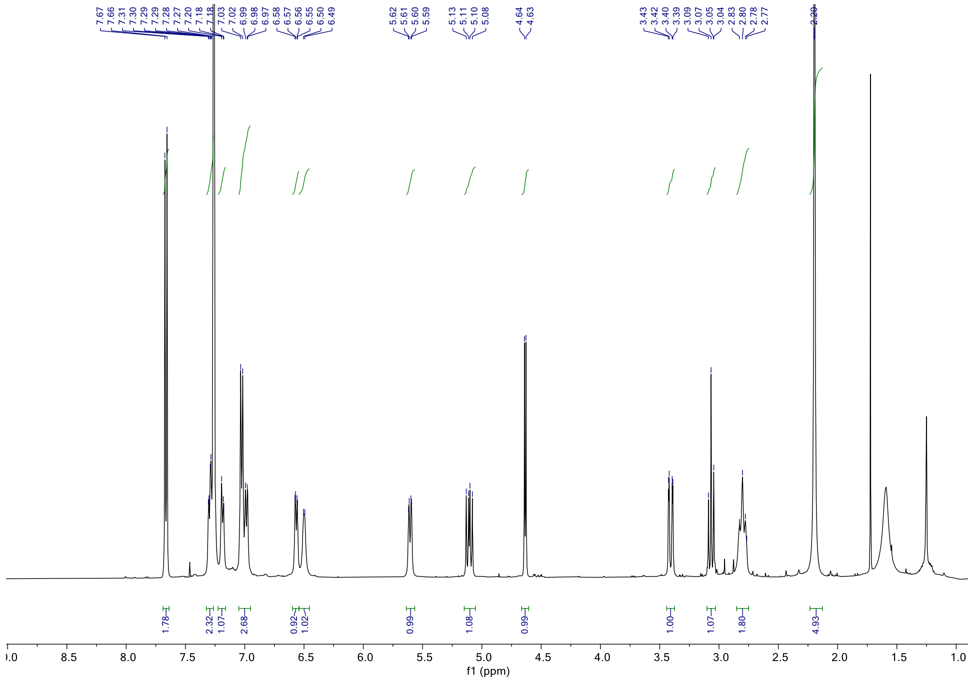

^{13}C NMR in $CDCl_3$

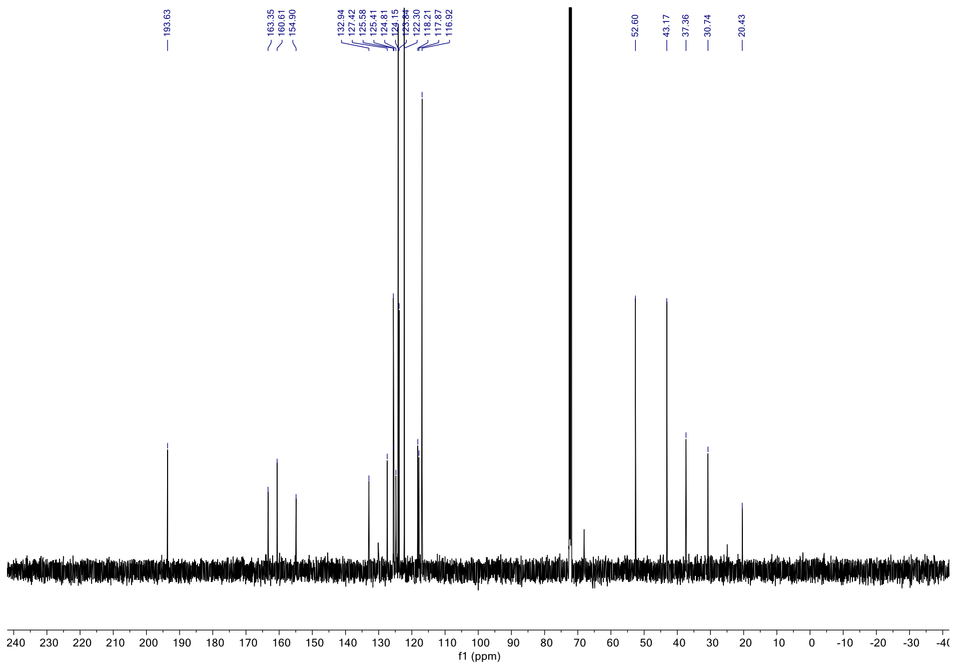

COSY in $CDCl_3$

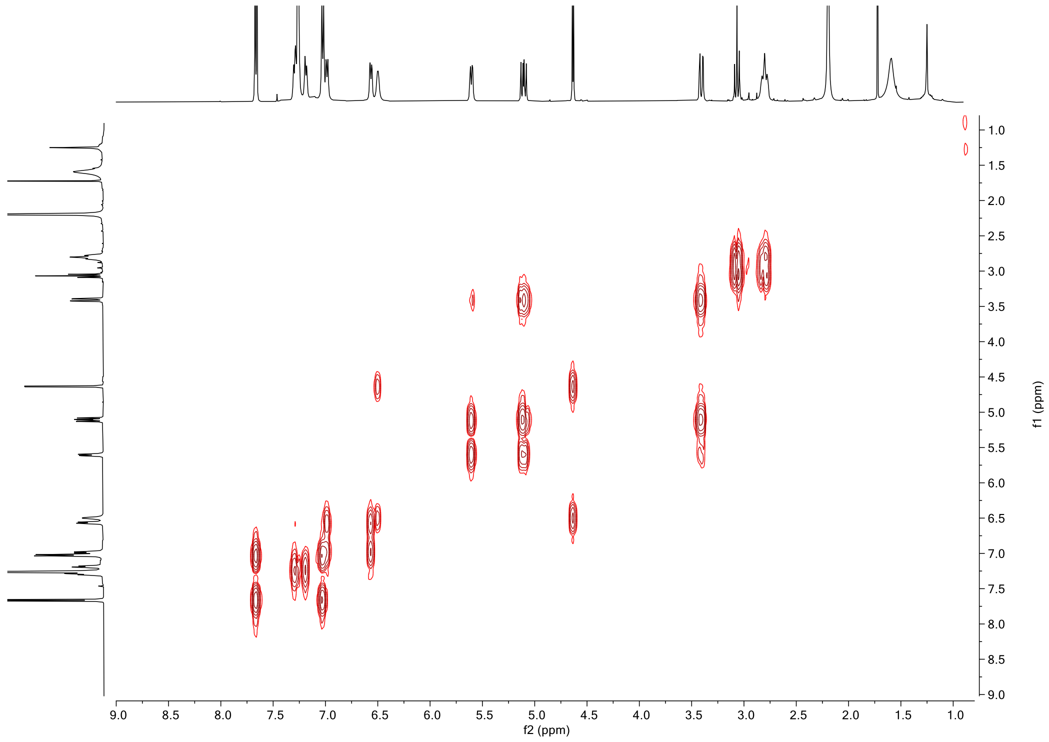

HSQC in $CDCl_3$

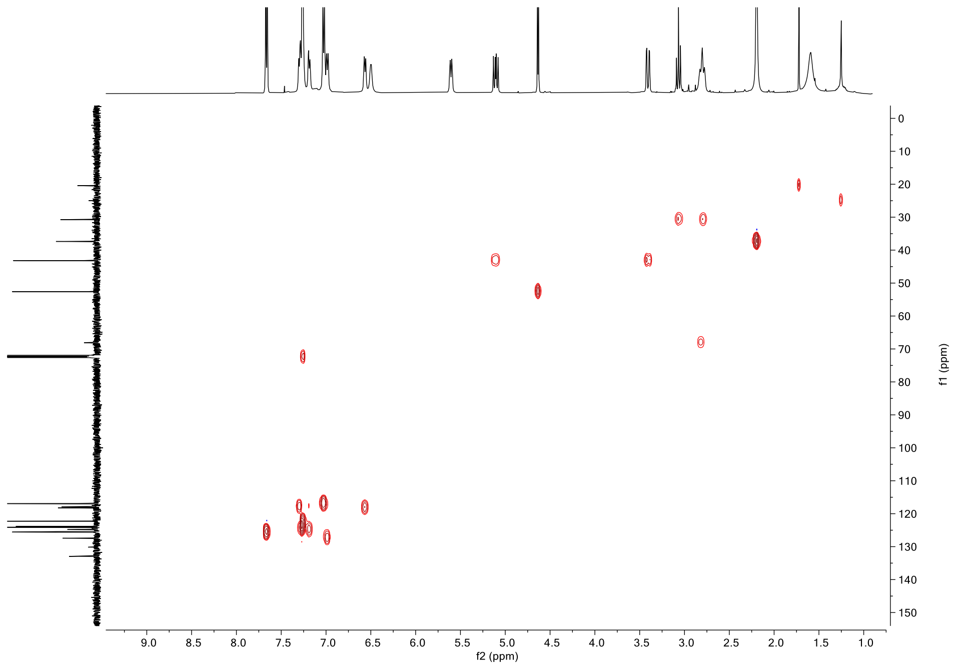

(8S,11S)-8-Isopentyl-N,N-dimethyl-4,7,10-trioxo-2-oxa-6,9-diaza-1,3(1,4)-dibenzenacyclododecaphan-11-aminium 2,2,2-trifluoroacetate ***(22a)***.

^{1}H NMR in $CDCl_3$

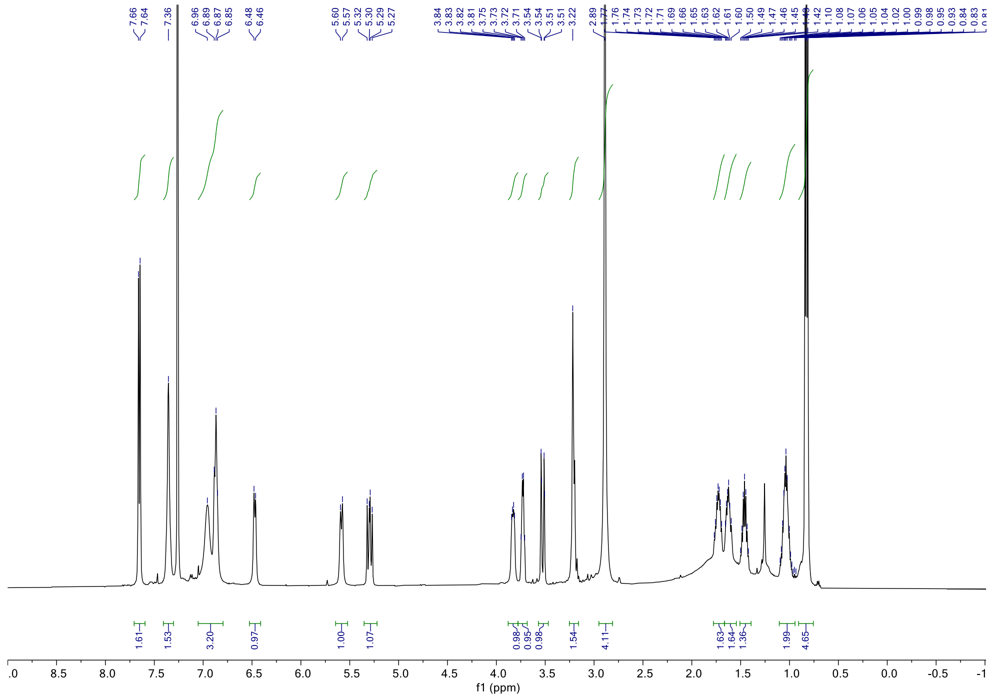

^{13}C NMR in $CDCl_3$

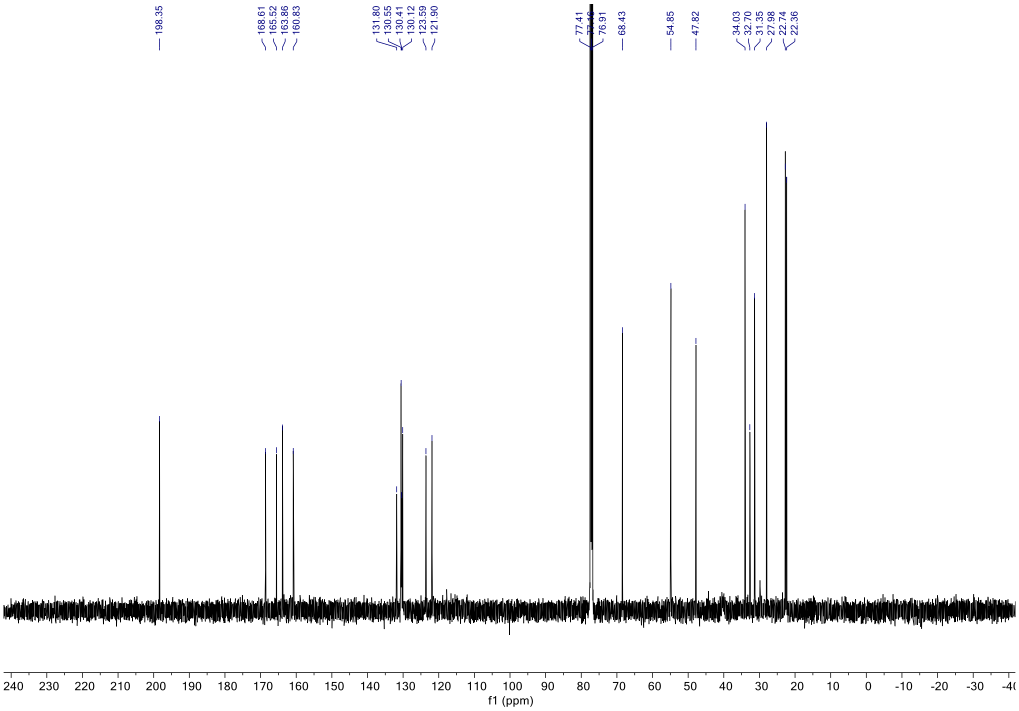

COSY in $CDCl_3$

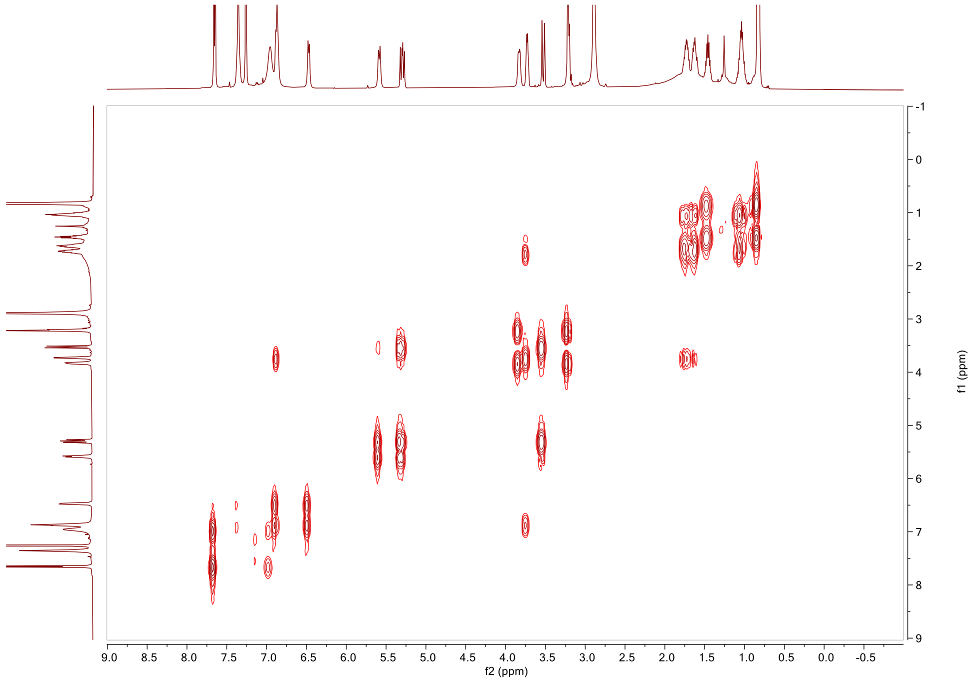

HSQC in $CDCl_3$

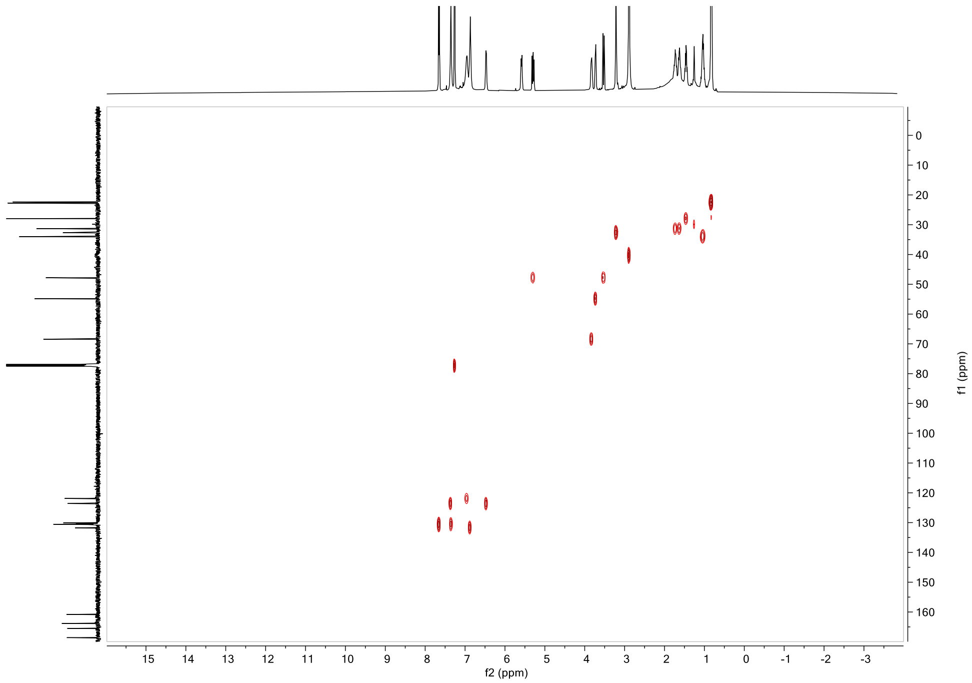

(8S,11S)-8-Isopentyl-N,N-dimethyl-4,7,10-trioxo-2-oxa-6,9-diaza-1,3(1,4)-dibenzenacyclododecaphan-11-aminium 2,2,2-trifluoroacetate ***(22a)***.

^{1}H NMR in DMSO-d_6

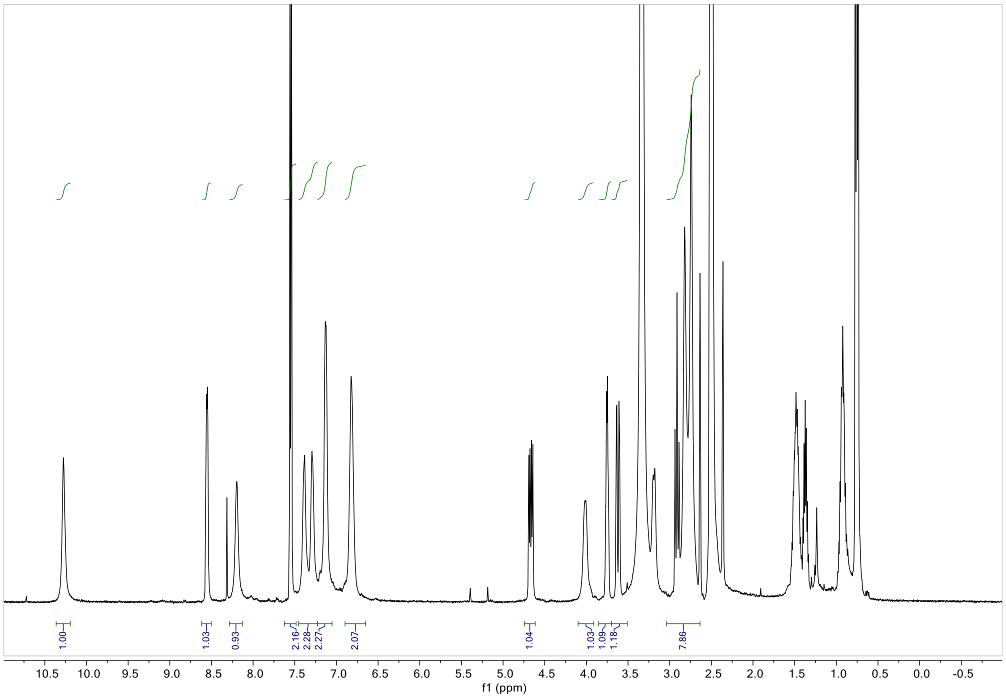

^{13}C NMR in DMSO-d$_6$

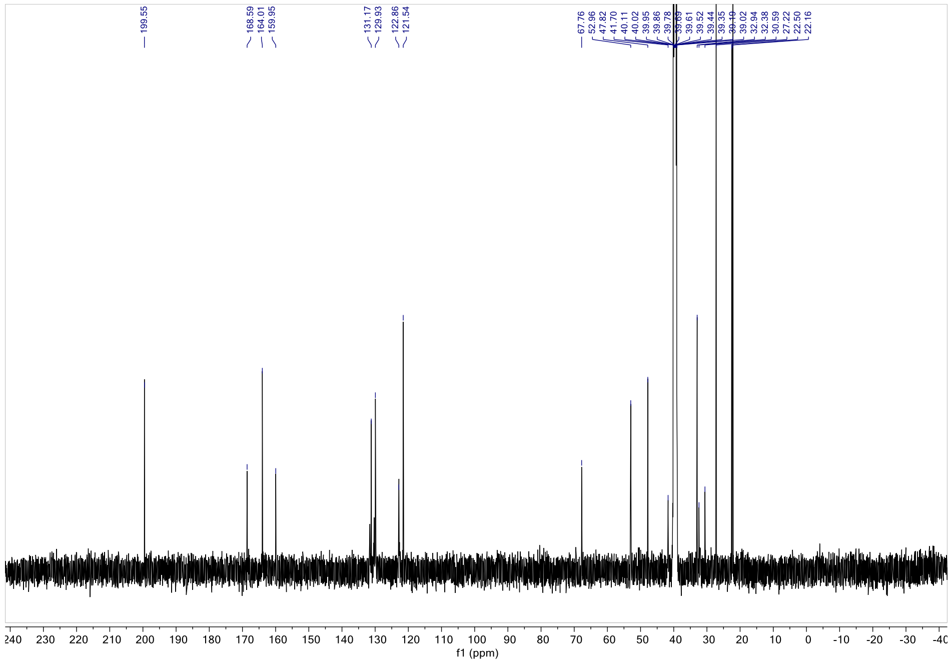

COSY in DMSO-d$_6$

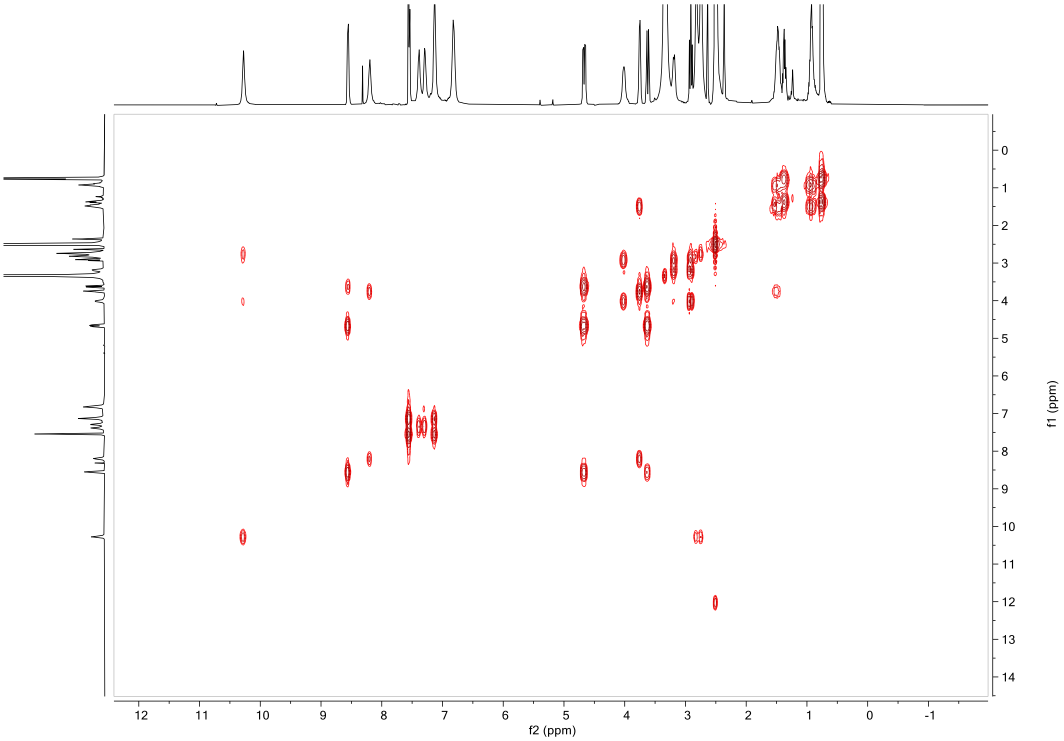

HSQC in DMSO-d6

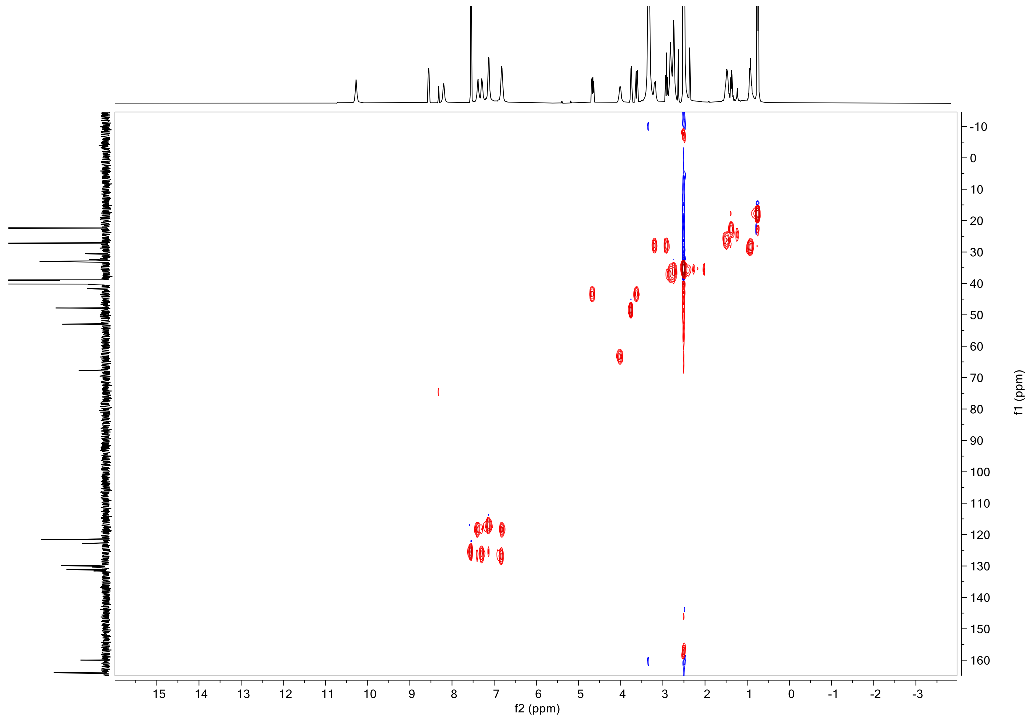

*(8S,11S)-N,N-Dimethyl-4,7,10-trioxo-8-phenethyl-2-oxa-6,9-diaza-1,3(1,4)-dibenzenacyclododecaphan-11-aminium 2,2,2-trifluoroacetate (**22b**).*

^{1}H NMR in $CDCl_3$

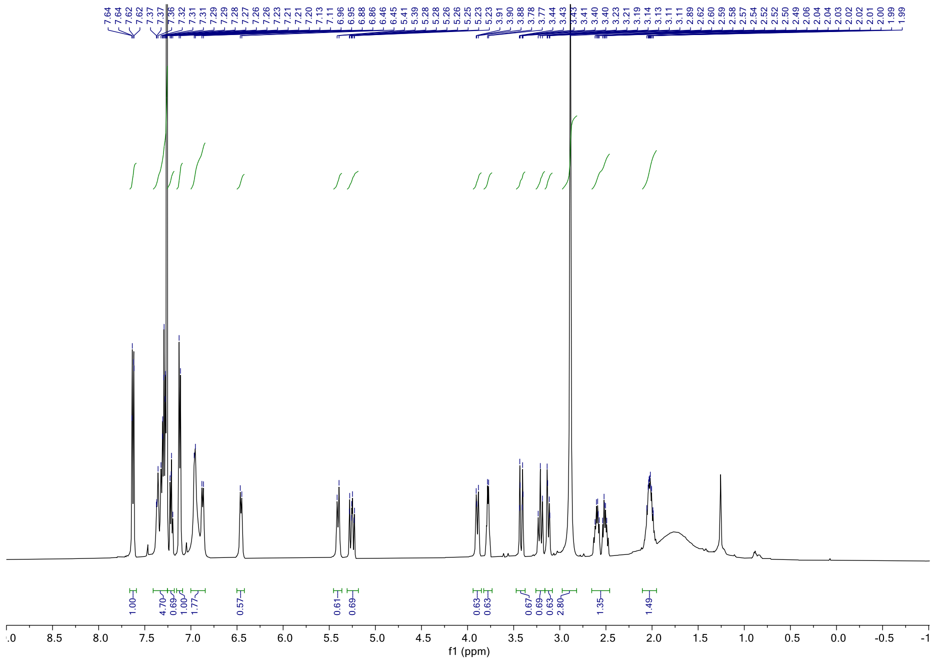

^{13}C NMR in $CDCl_3$

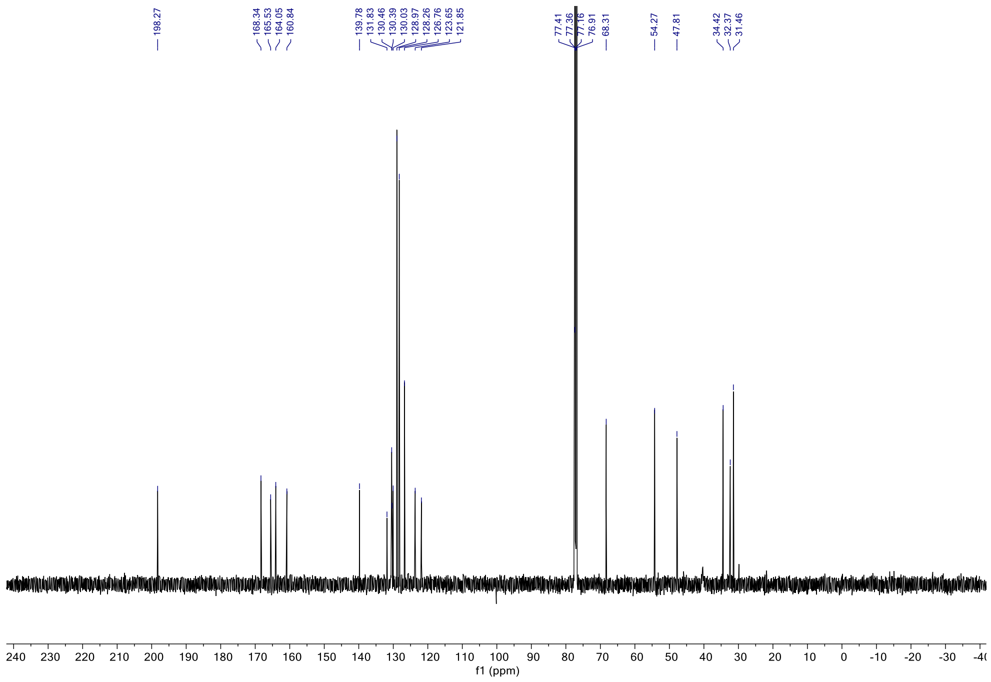

COSY in $CDCl_3$

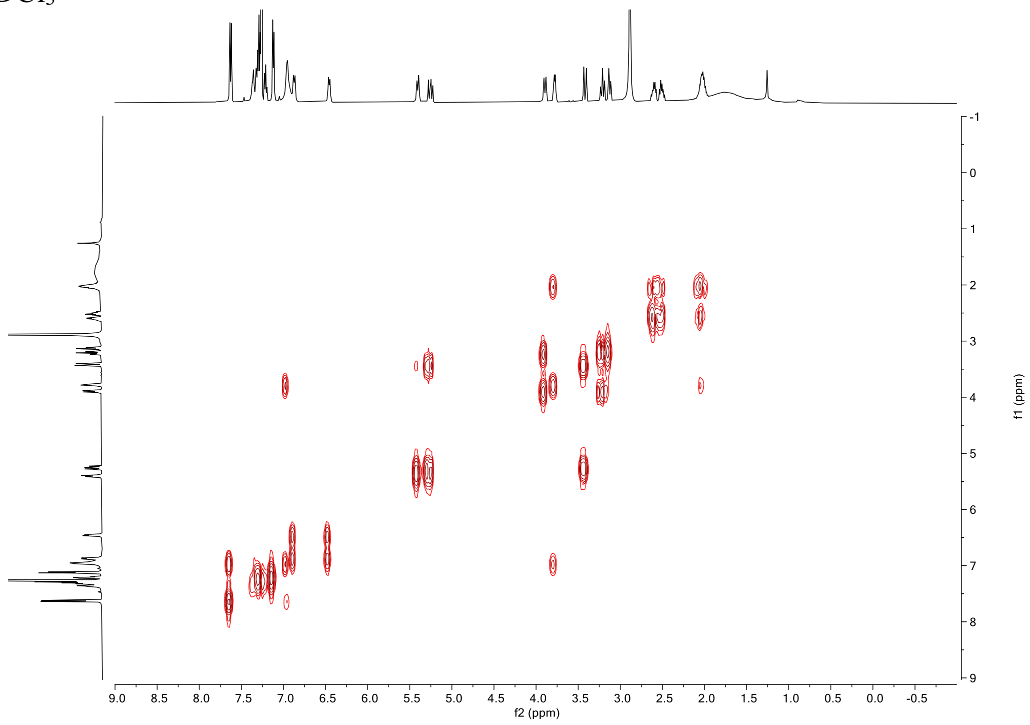

HSQC in $CDCl_3$

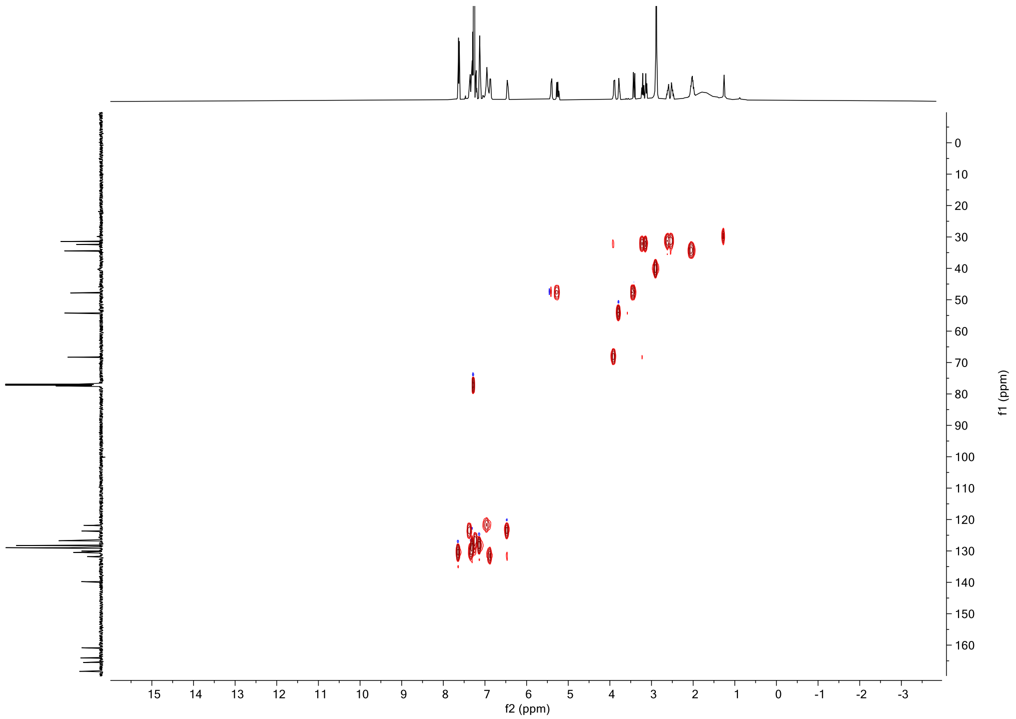

*(8S,11S)-N,N-Dimethyl-4,7,10-trioxo-8-phenethyl-2-oxa-6,9-diaza-1,3(1,4)-dibenzenacyclododecaphan-11-aminium 2,2,2-trifluoroacetate **(22b)**.*

^{1}H NMR in DMSO-d_6

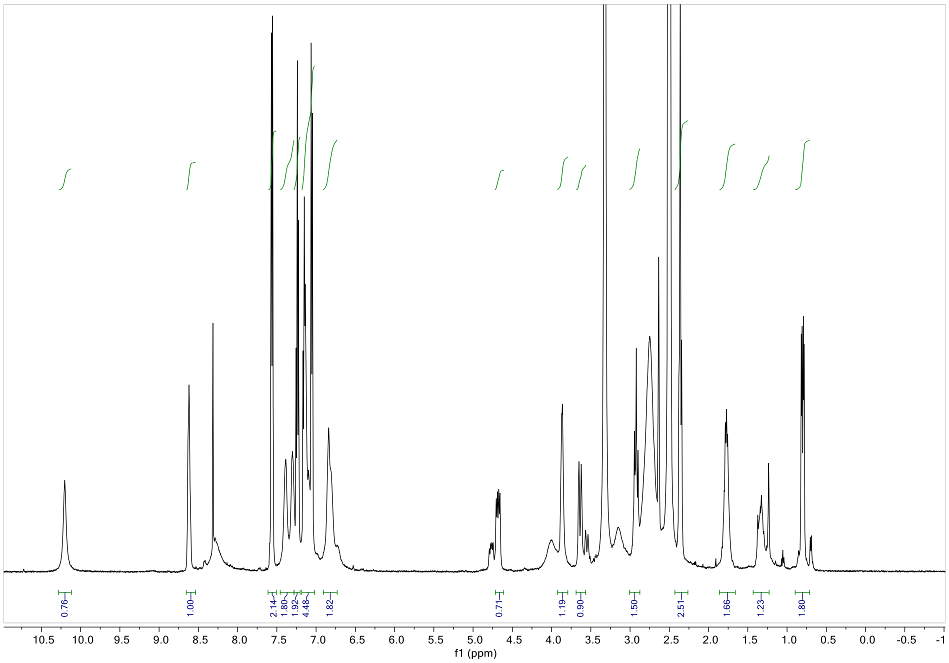

^{13}C NMR in DMSO-d$_6$

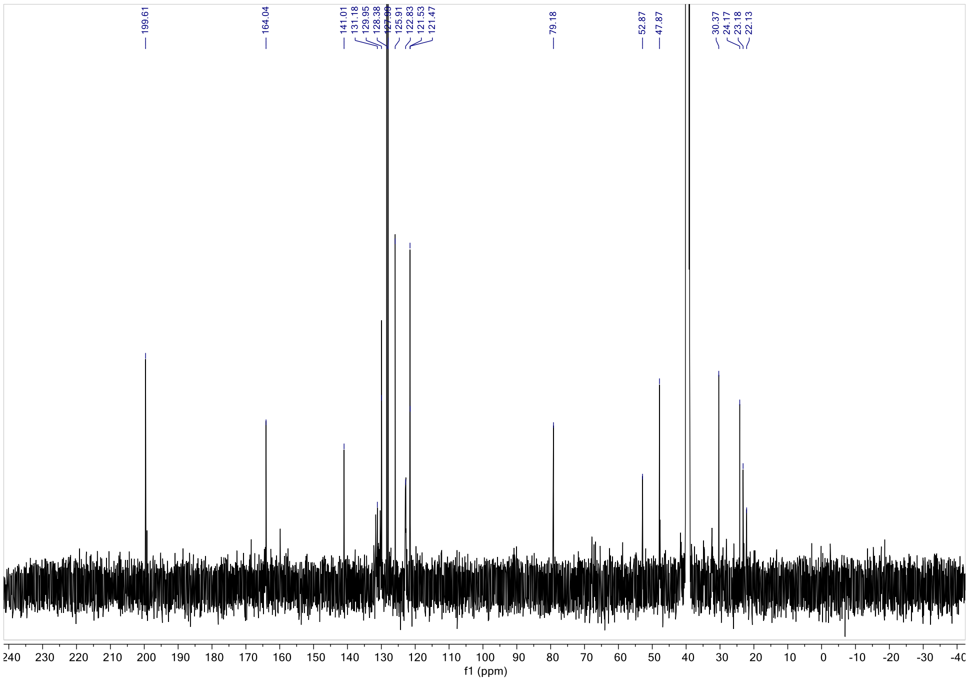

COSY in DMSO-d$_6$

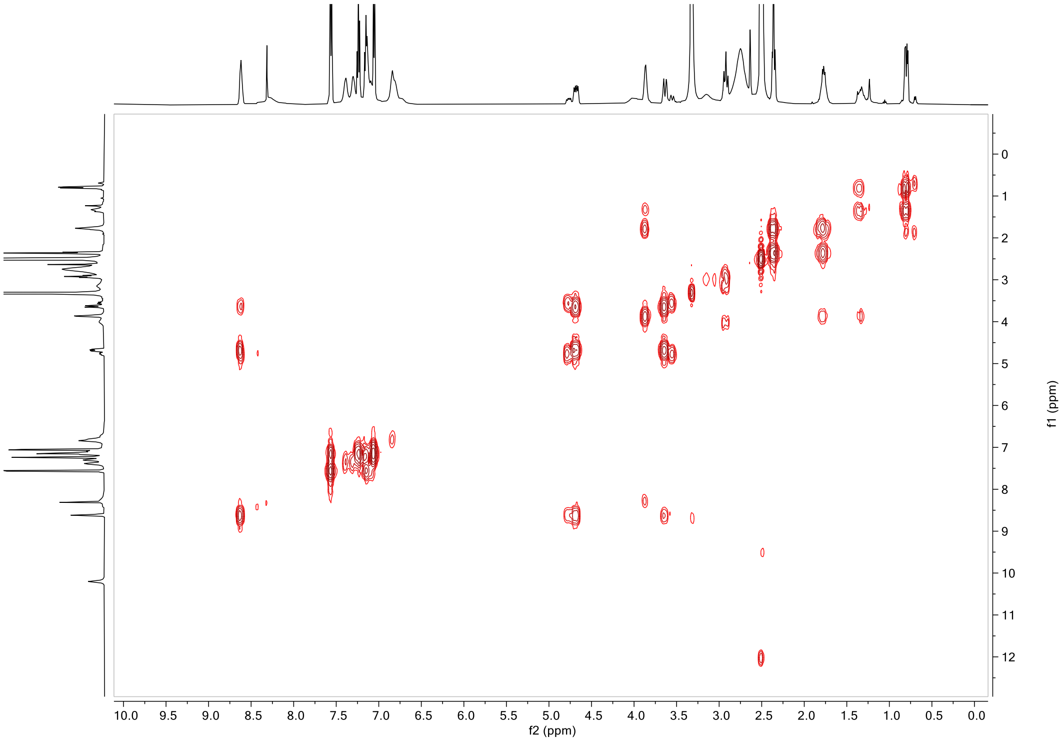

HSQC in DMSO-d$_6$

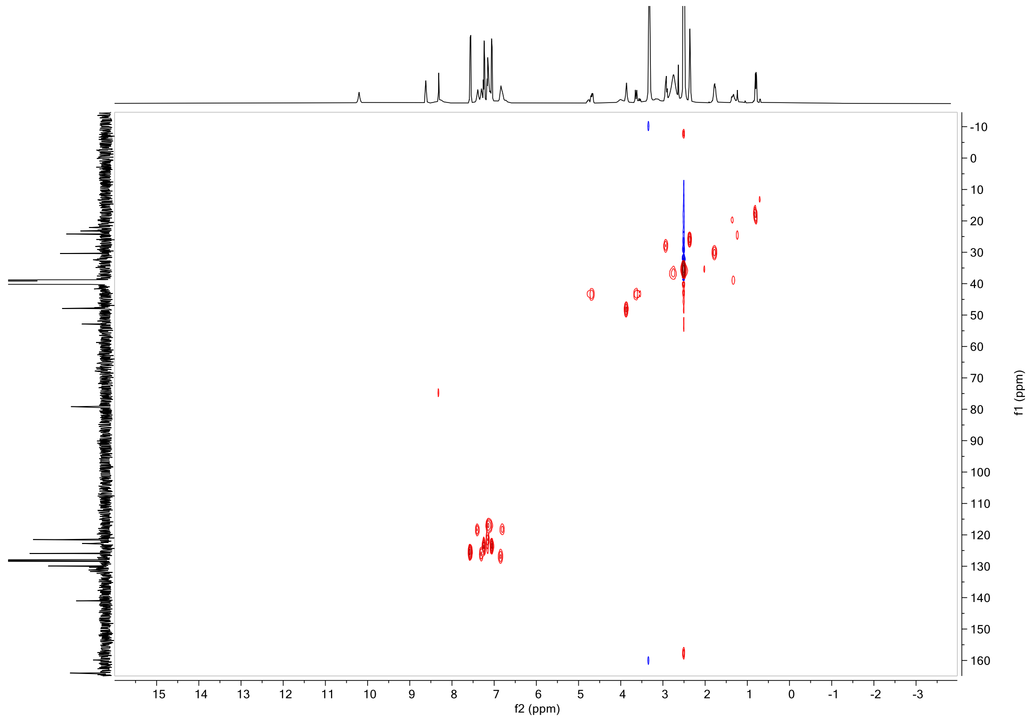

*(8S,11S)-8-Benzyl-N,N-dimethyl-4,7,10-trioxo-2-oxa-6,9-diaza-1,3(1,4)-dibenzenacyclododecaphan-11-aminium 2,2,2-trifluoroacetate (**22c**).*

^{1}H NMR in $CDCl_3$

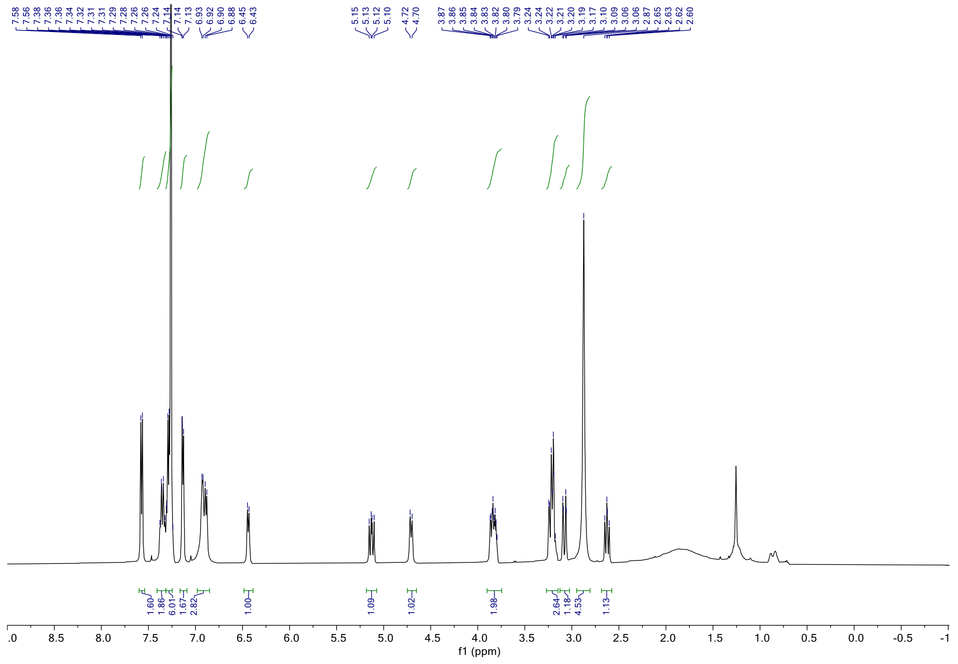

^{13}C NMR in $CDCl_3$

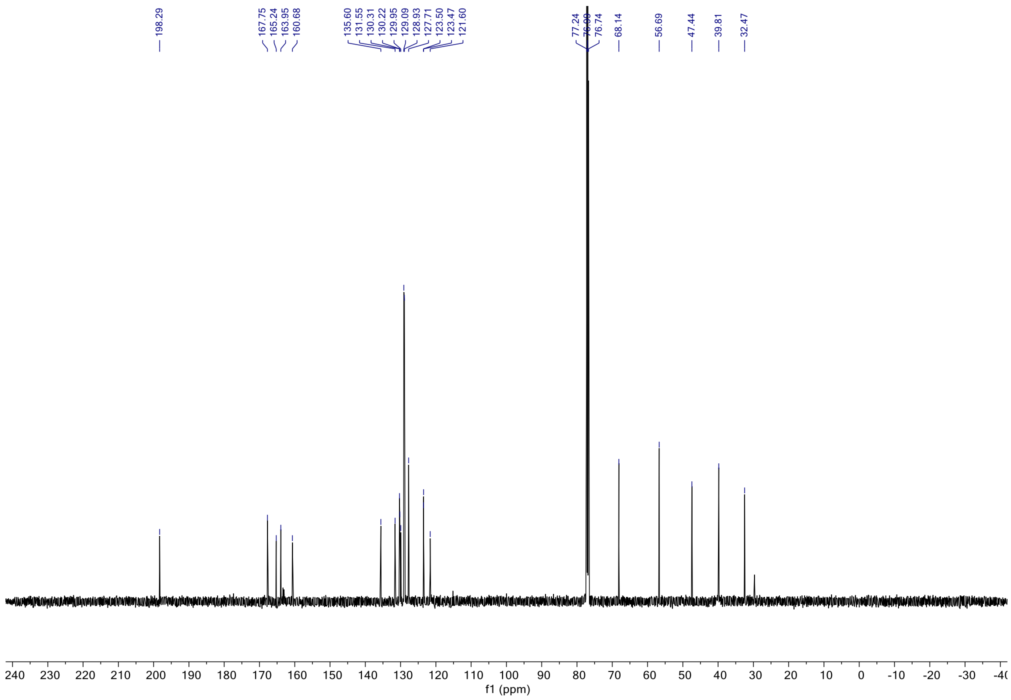

COSY in $CDCl_3$

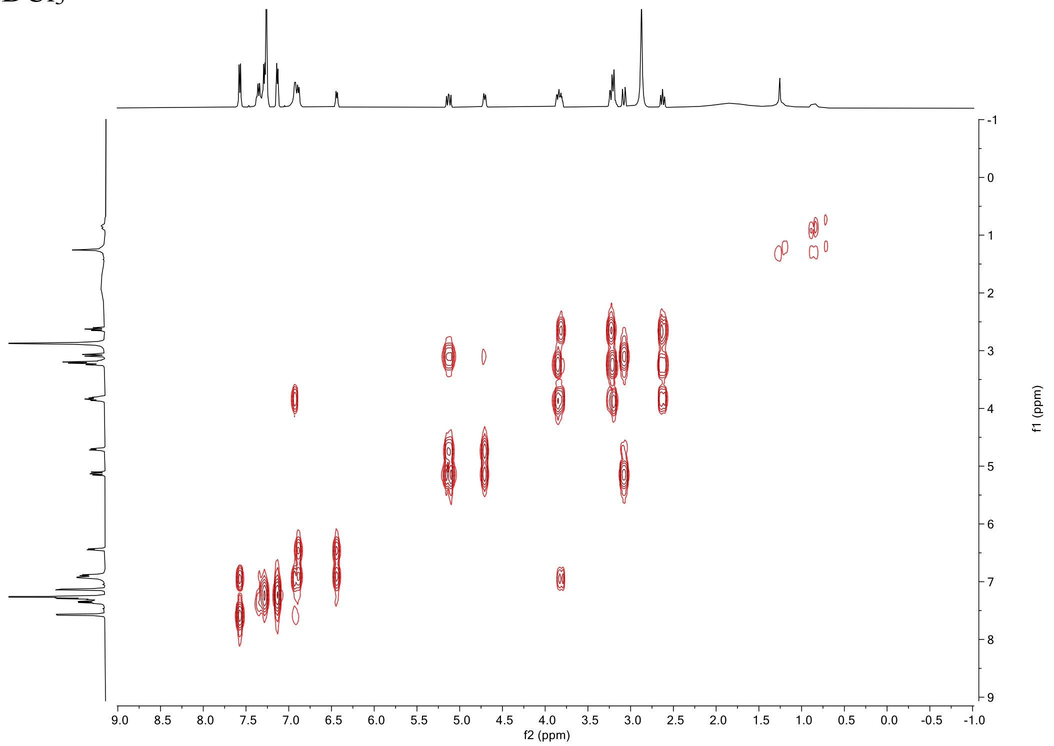

HSQC in $CDCl_3$

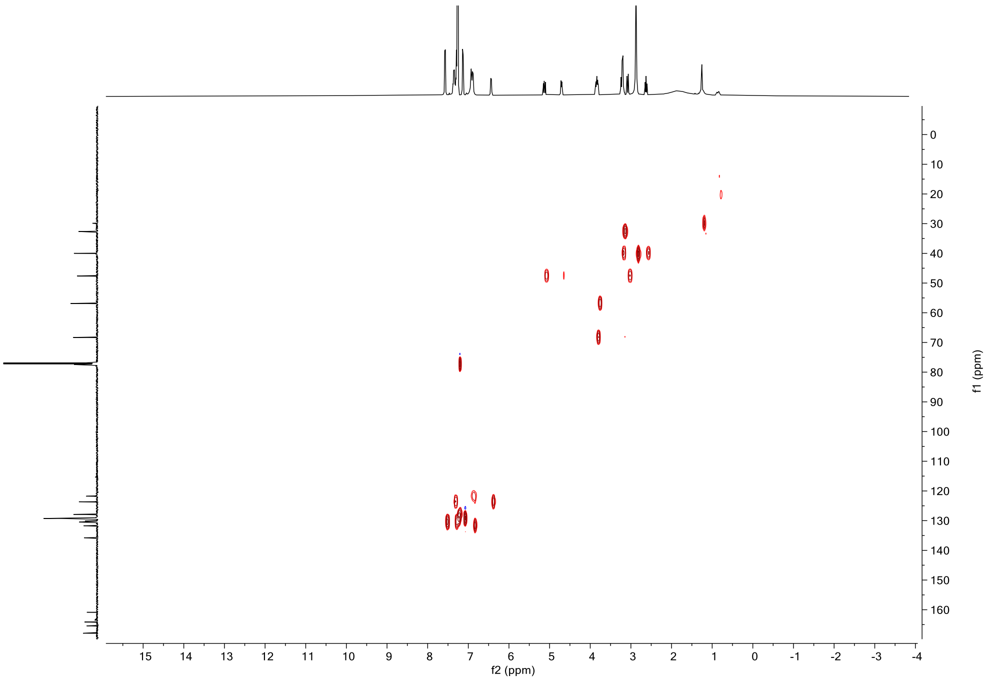

*(8S,11S)-8-Benzyl-N,N-dimethyl-4,7,10-trioxo-2-oxa-6,9-diaza-1,3(1,4)-dibenzenacyclododecaphan-11-aminium 2,2,2-trifluoroacetate (**22c**).*

^{1}H NMR in DMSO-d_6

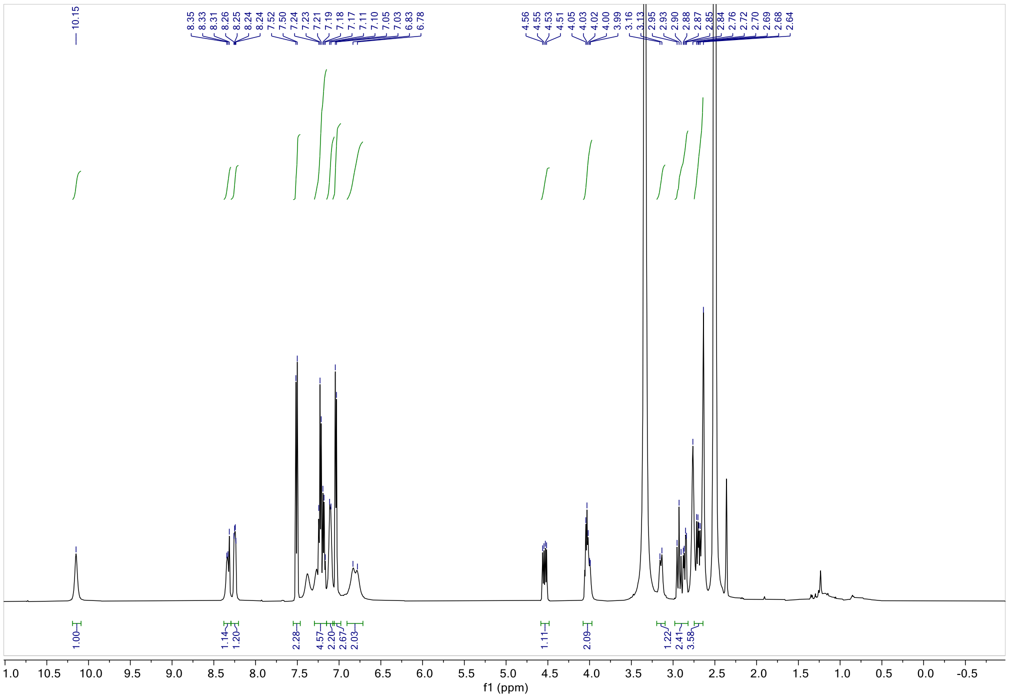

^{13}C NMR in DMSO-d$_6$

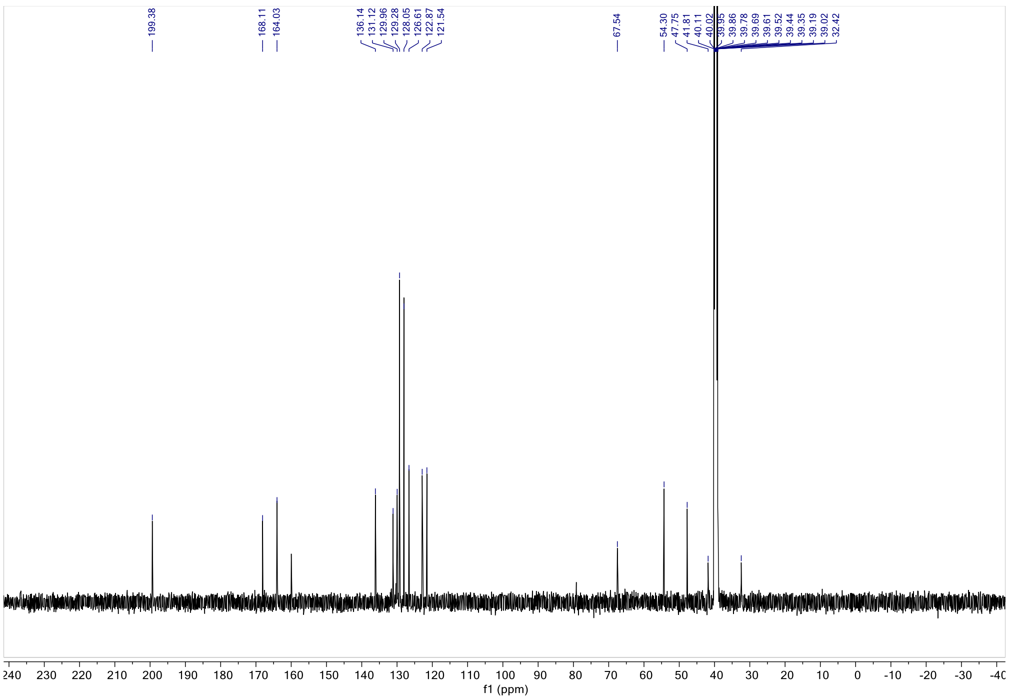

COSY in DMSO-d$_6$

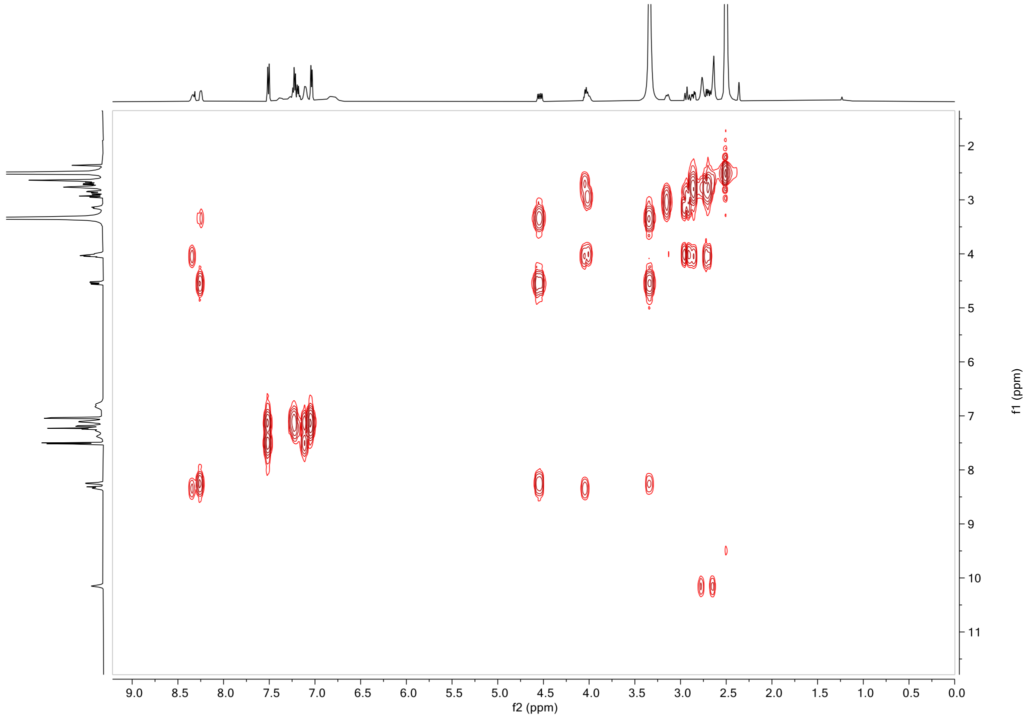

HSQC in DMSO-d_6

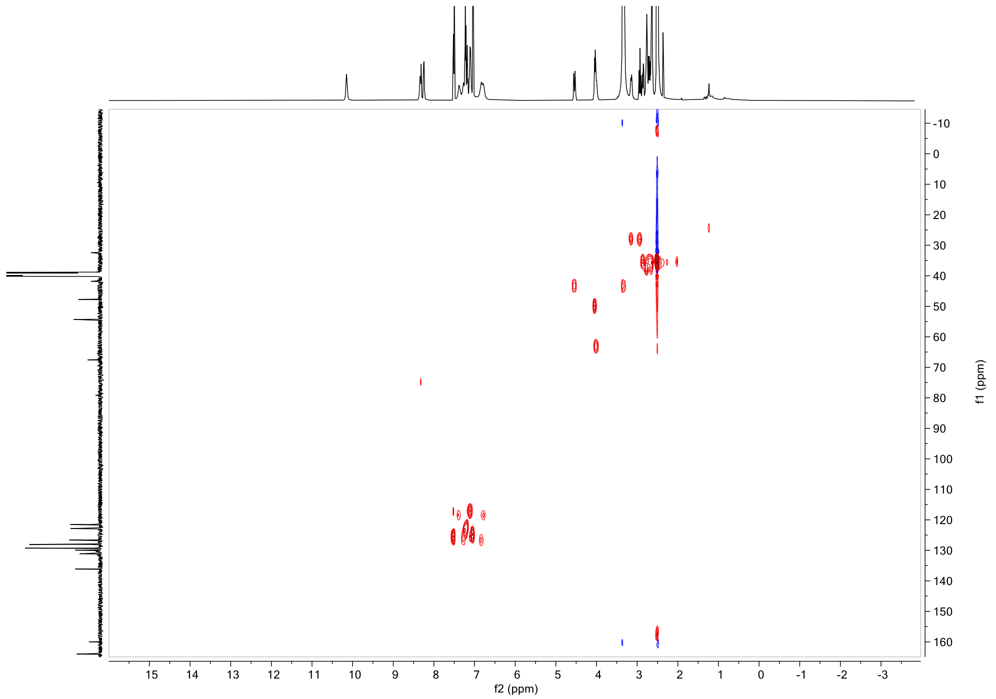

*(8S,11S)-8-Isobutyl-N,N-dimethyl-4,7,10-trioxo-2-oxa-6,9-diaza-1,3(1,4)-dibenzenacyclododecaphan-11-aminium 2,2,2-trifluoroacetate (**22e**).*

^{1}H NMR in $CDCl_3$

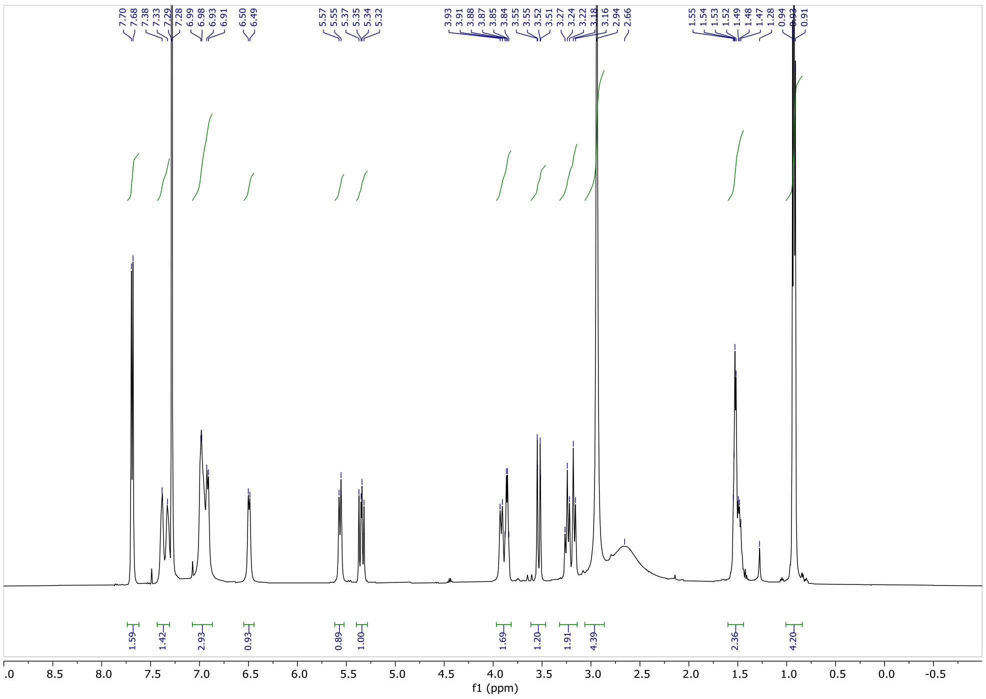

^{13}C NMR in $CDCl_3$

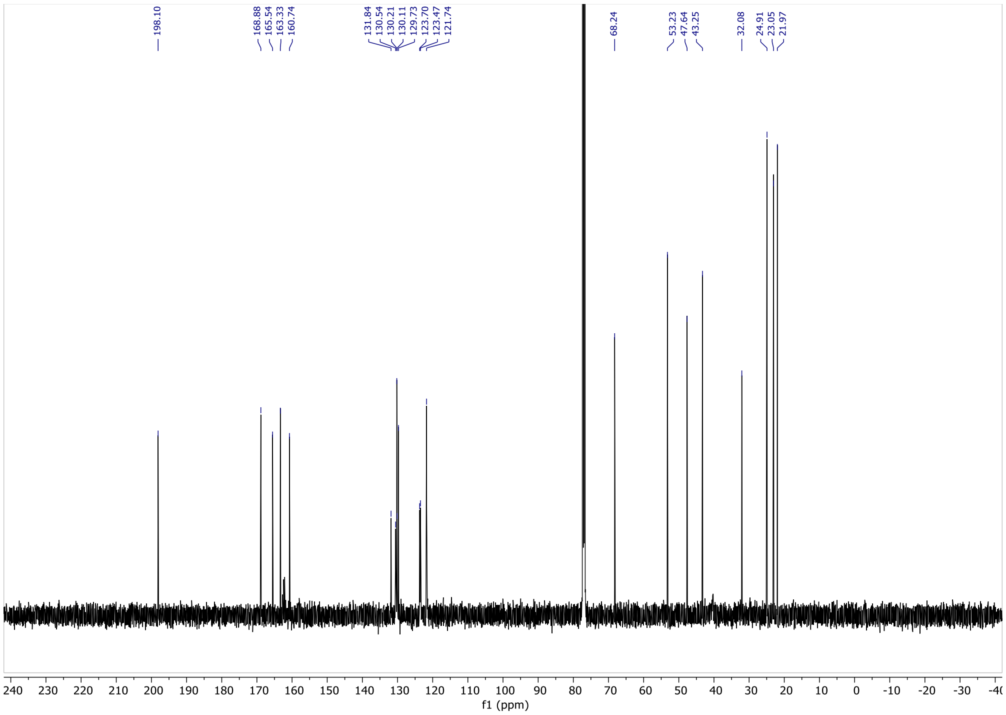

COSY in $CDCl_3$

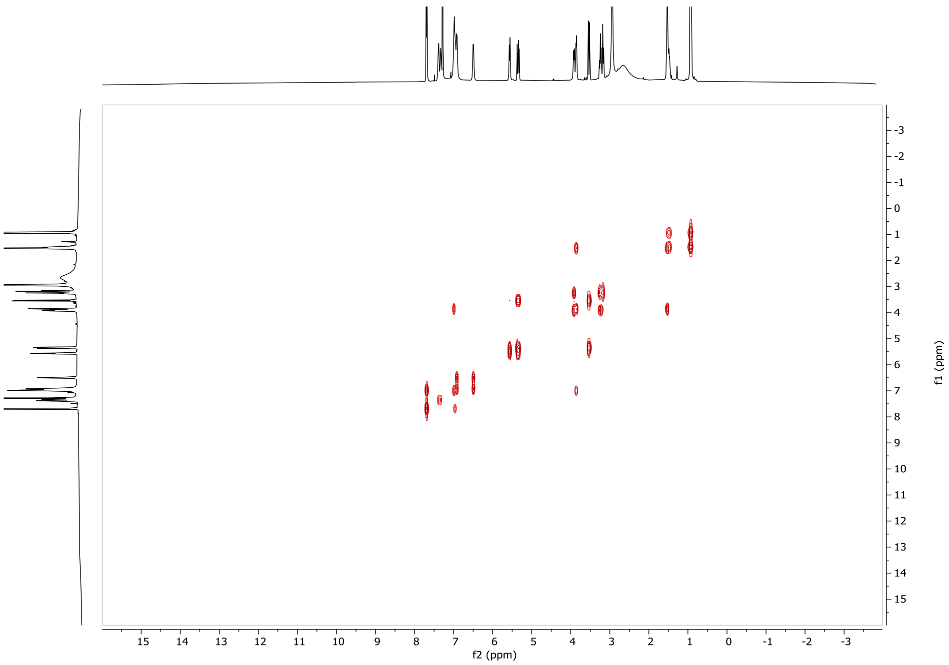

HSQC in $CDCl_3$

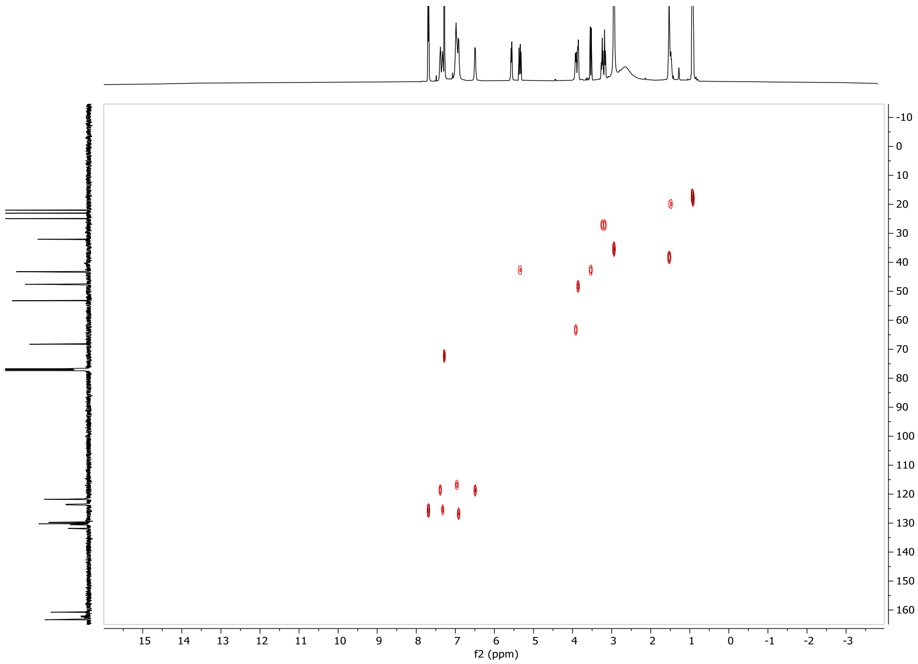

*(8S,11S)-8-Isobutyl-N,N-dimethyl-4,7,10-trioxo-2-oxa-6,9-diaza-1,3(1,4)-dibenzenacyclododecaphan-11-aminium 2,2,2-trifluoroacetate (**22e**).*

^1H NMR in DMSO-d_6

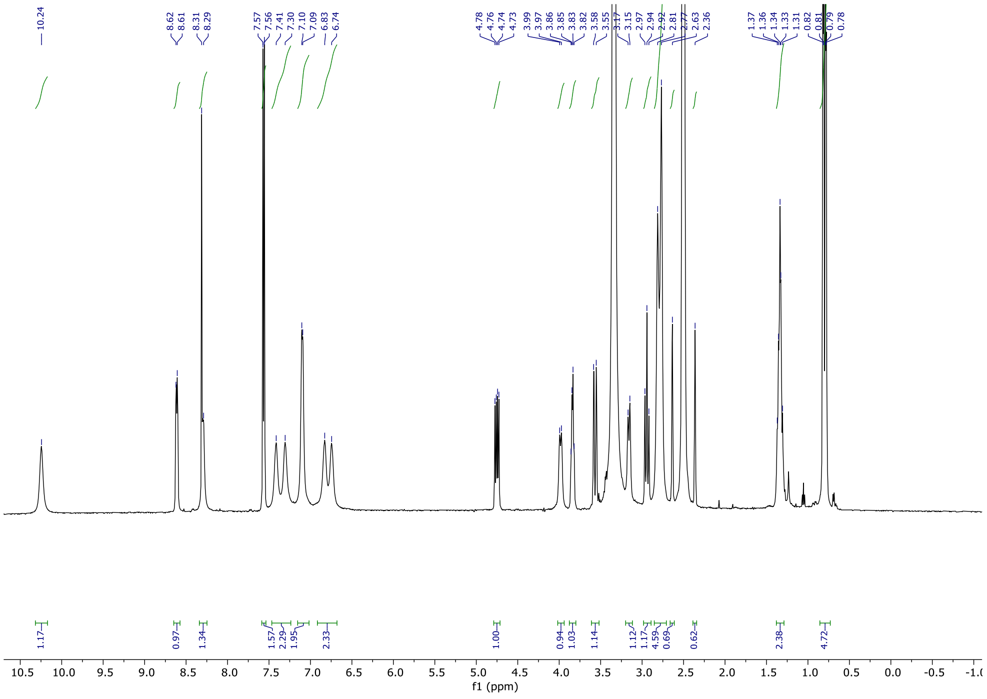

^{13}C NMR in DMSO-d$_6$

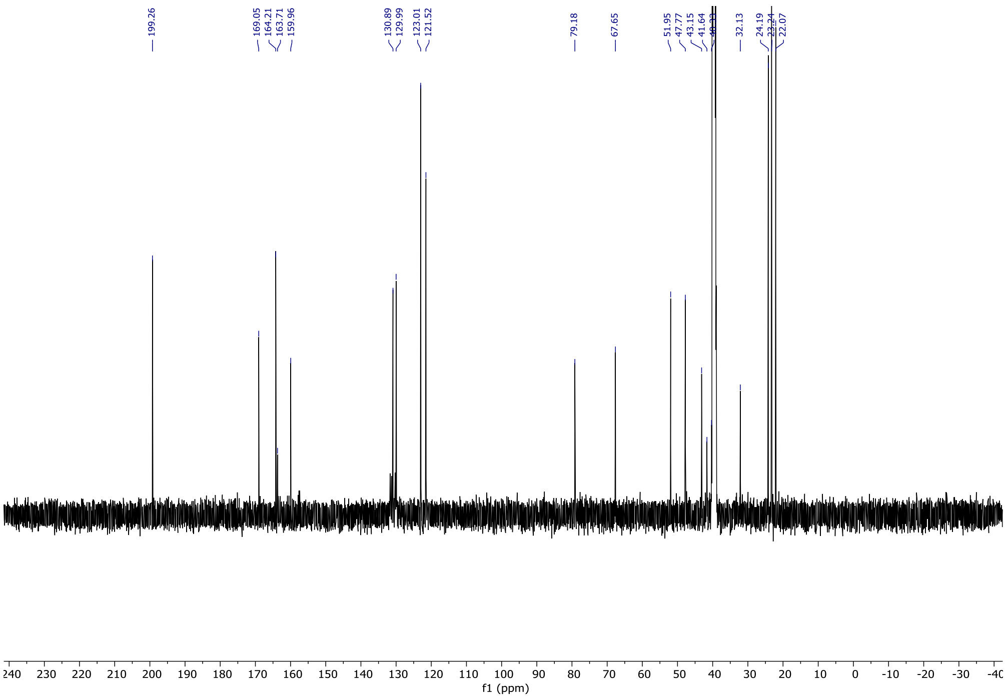

COSY in DMSO-d$_6$

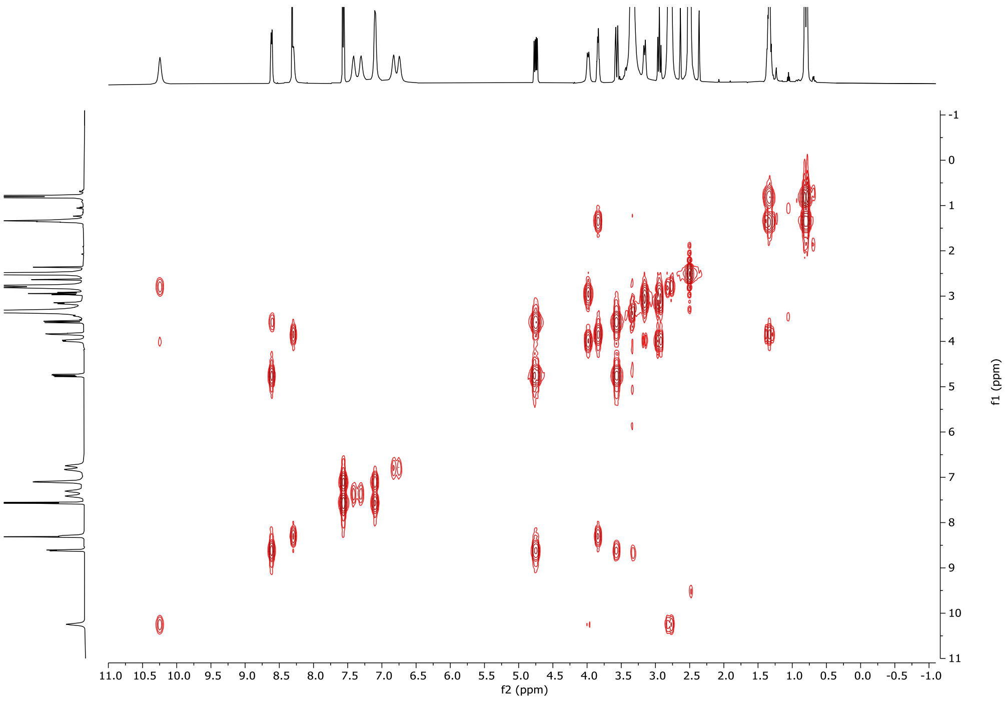

HSQC in DMSO-d_6

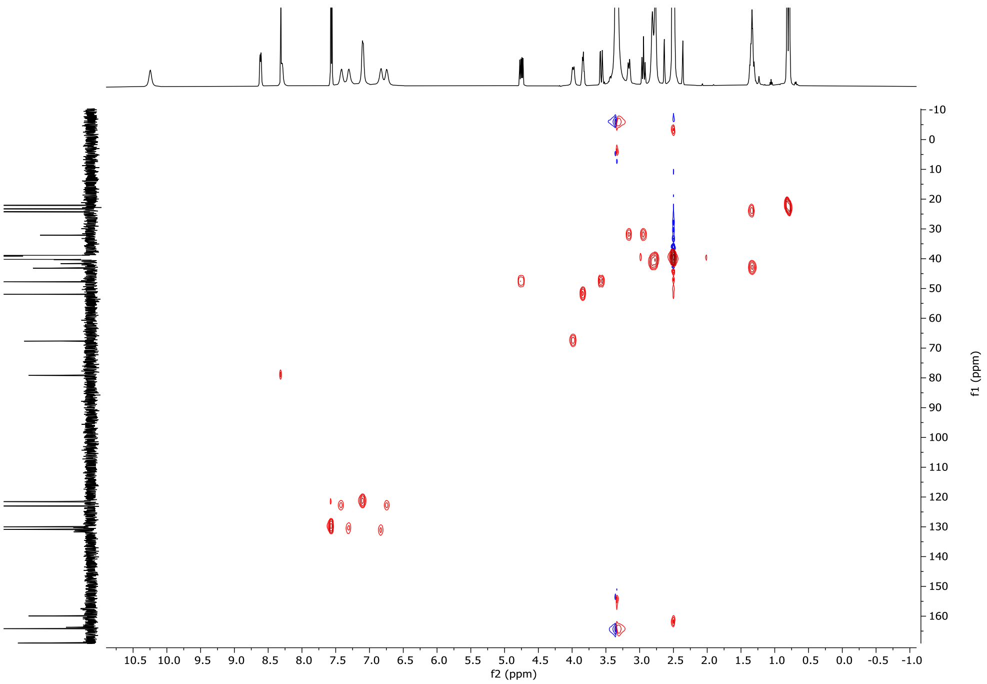

*(8S,11S)-N,N-Dimethyl-4,7,10-trioxo-8-phenyl-2-oxa-6,9-diaza-1,3(1,4)-dibenzenacyclododecaphan-11-aminium 2,2,2-trifluoroacetate (**22j**).*

^{1}H NMR in $CDCl_3$

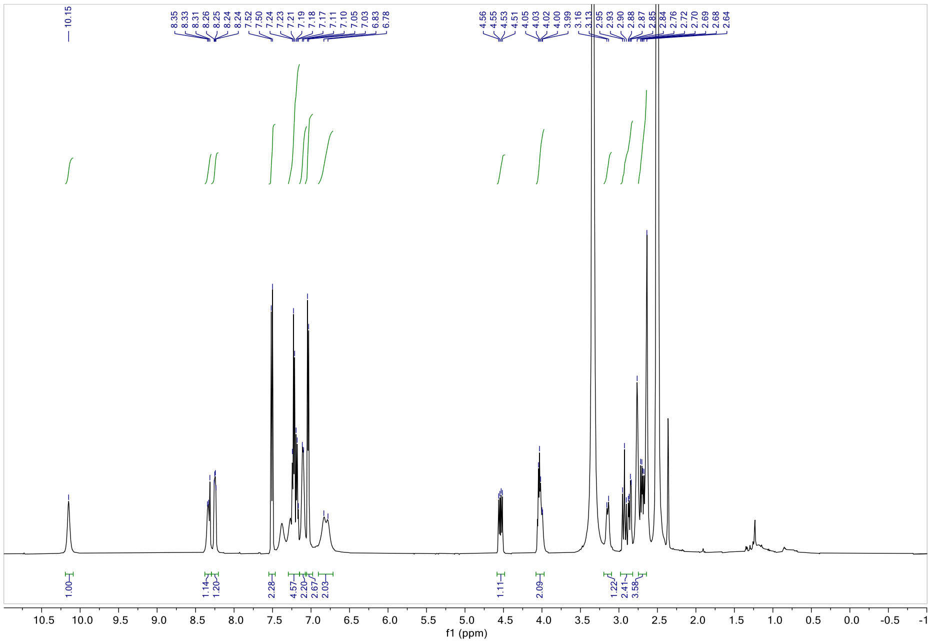

^{13}C NMR in $CDCl_3$

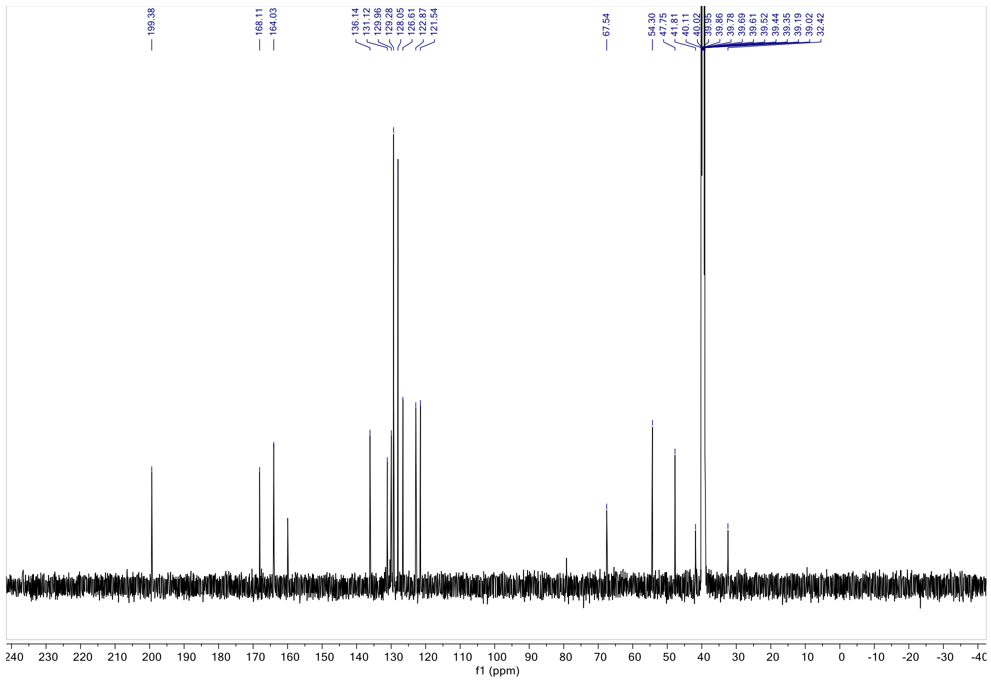

COSY in $CDCl_3$

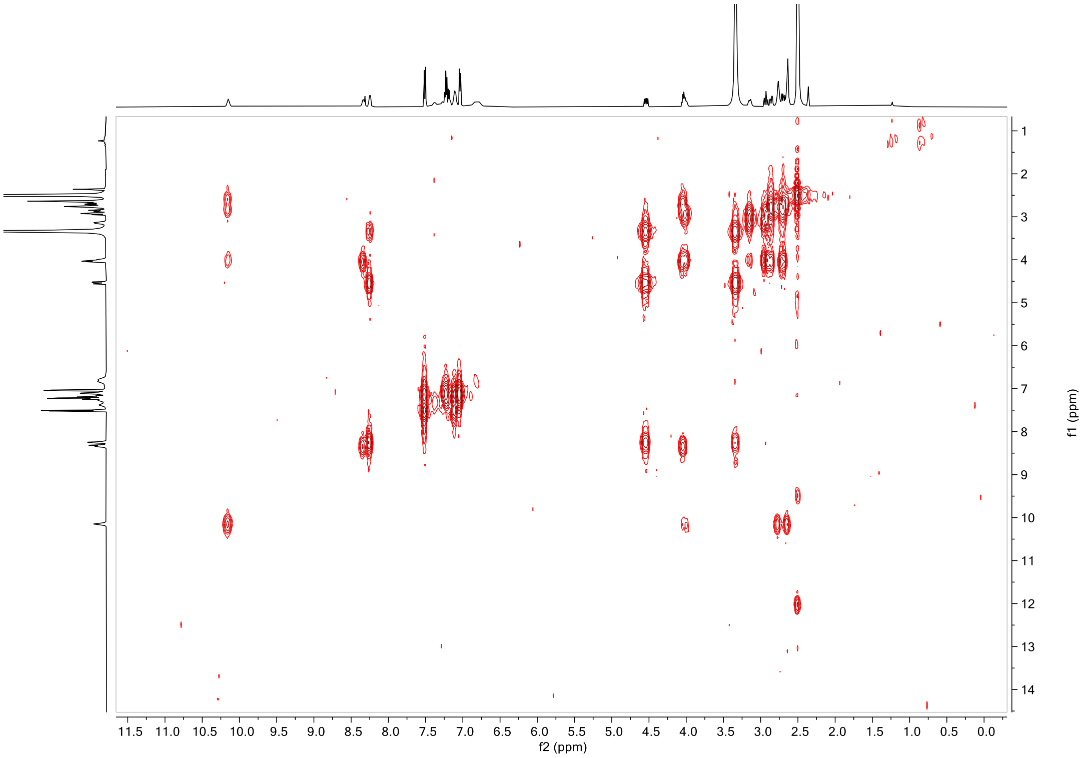

HSQC in $CDCl_3$

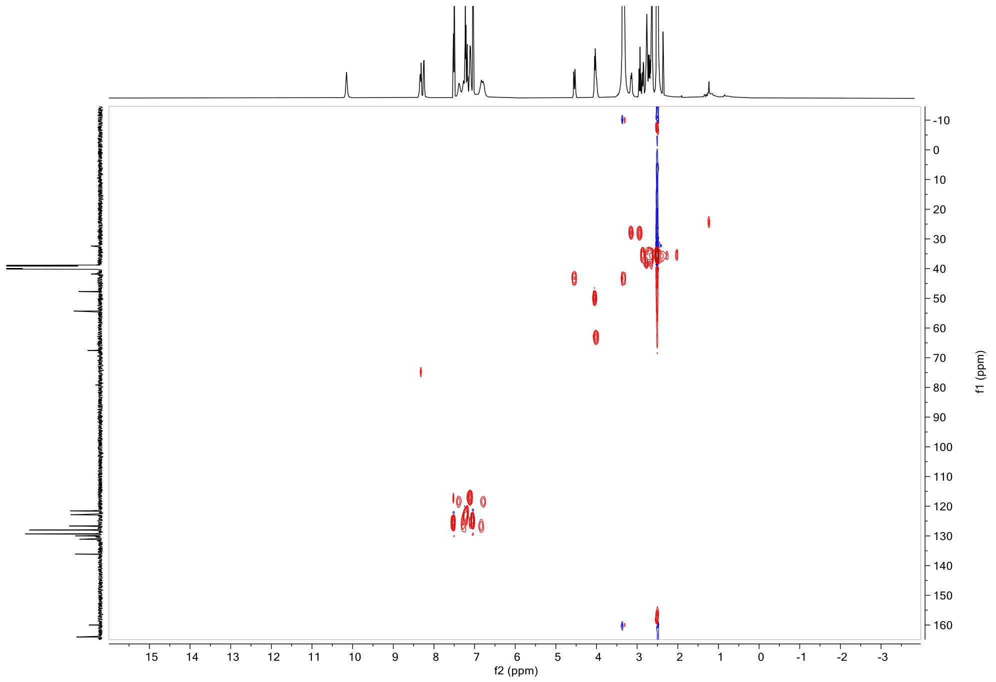

*(8S,11S)-N,N-Dimethyl-4,7,10-trioxo-8-phenyl-2-oxa-6,9-diaza-1,3(1,4)-dibenzenacyclododecaphan-11-aminium 2,2,2-trifluoroacetate (**22j**).*

^1H NMR in DMSO-d_6

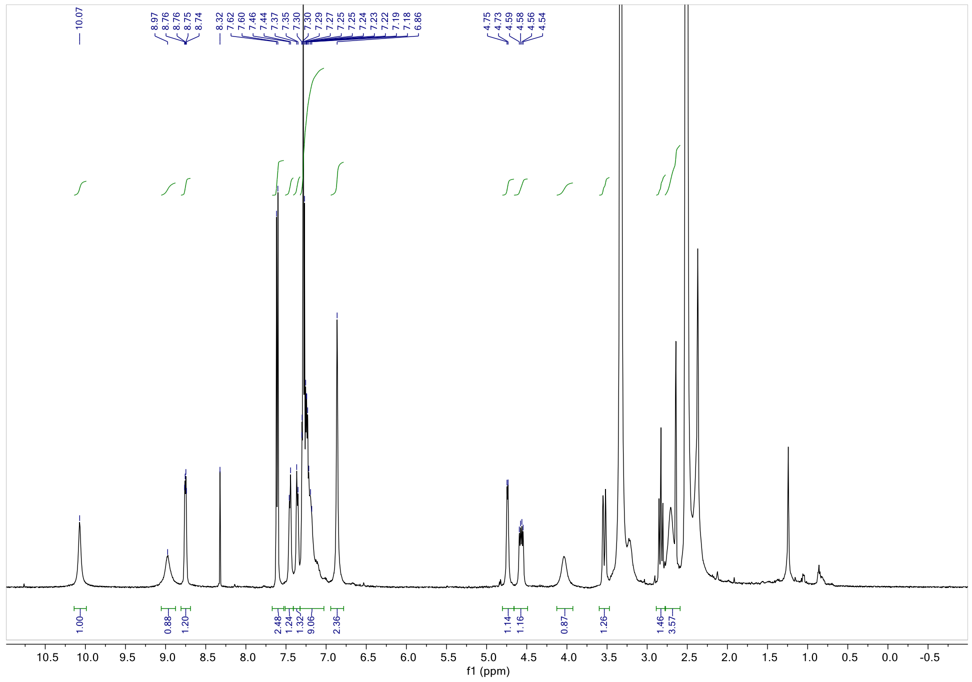

^{13}C NMR in DMSO-d_6

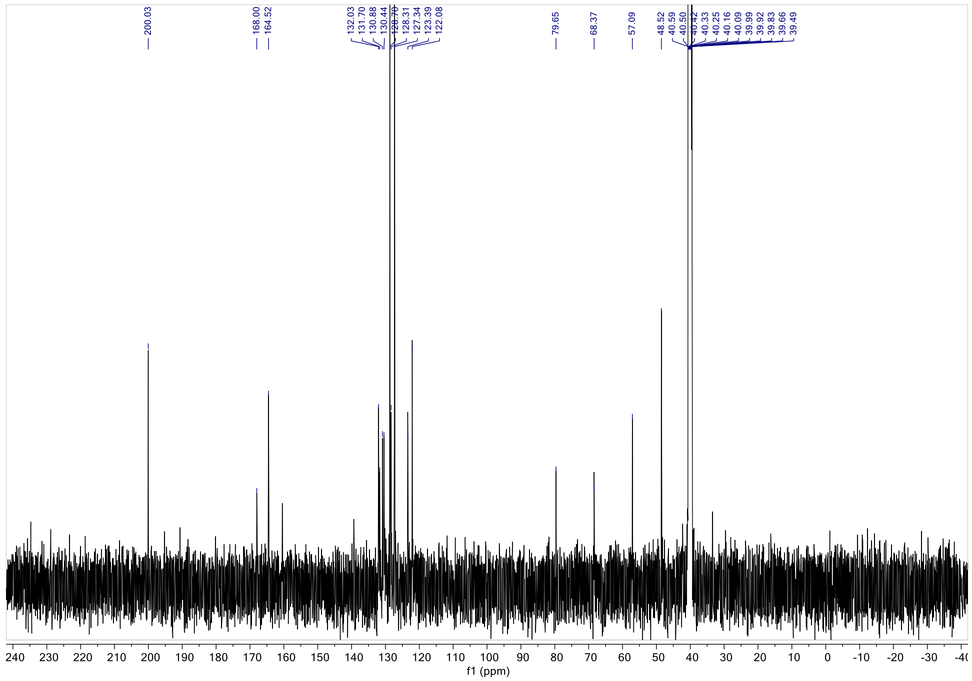

COSY in DMSO-d_6

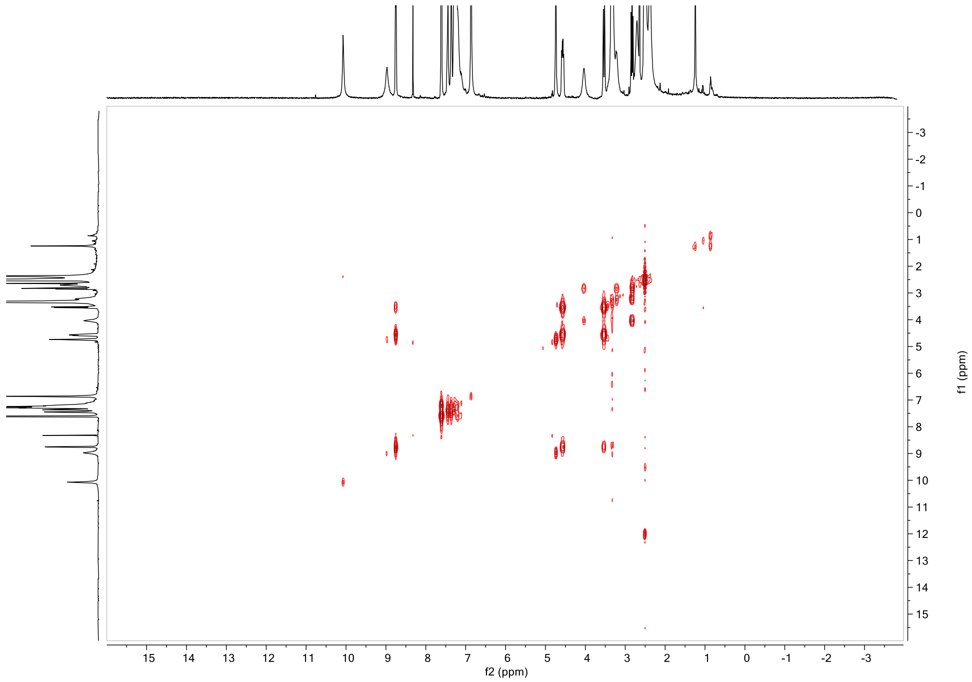

HSQC in DMSO-d$_6$

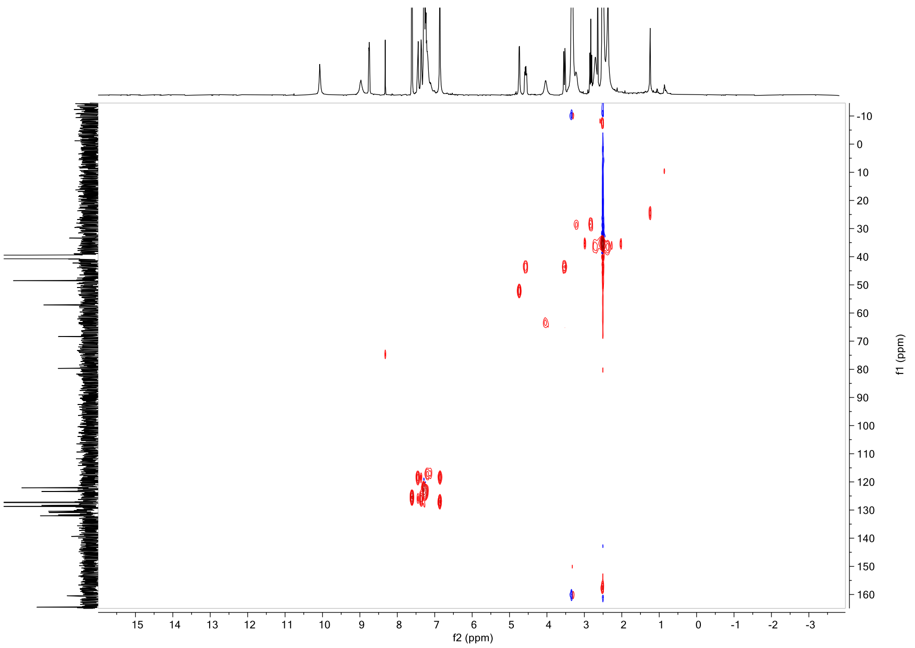

tert-Butyl ((2S)-1-((2-hydroxy-2-phenylethyl)amino)-5-methyl-1-oxohexan-2-yl)carbamate ***(24a)***.
^{1}H NMR in DMSO-d$_6$

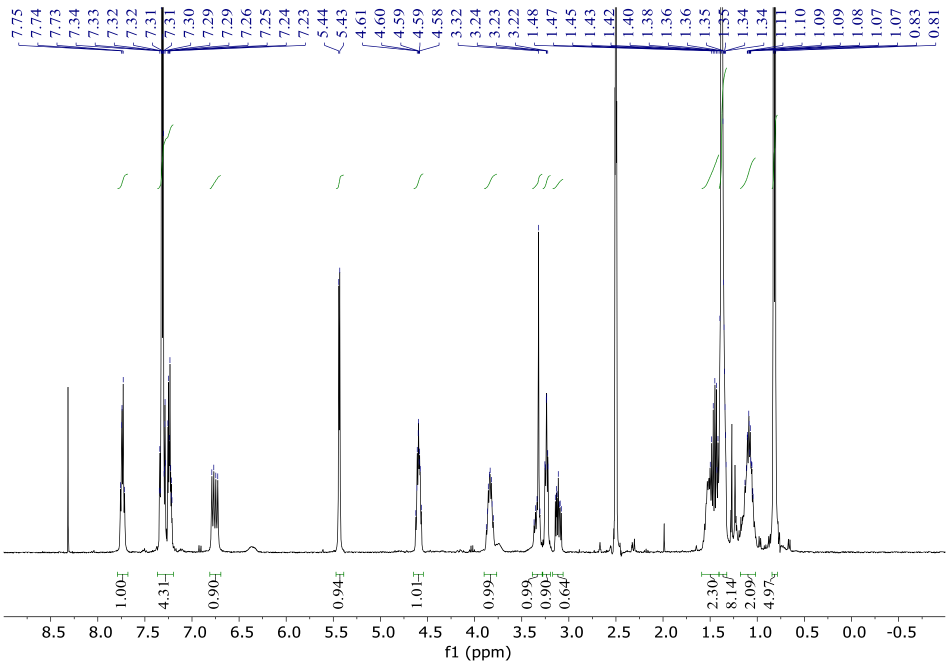

^{13}C NMR in DMSO-d$_6$

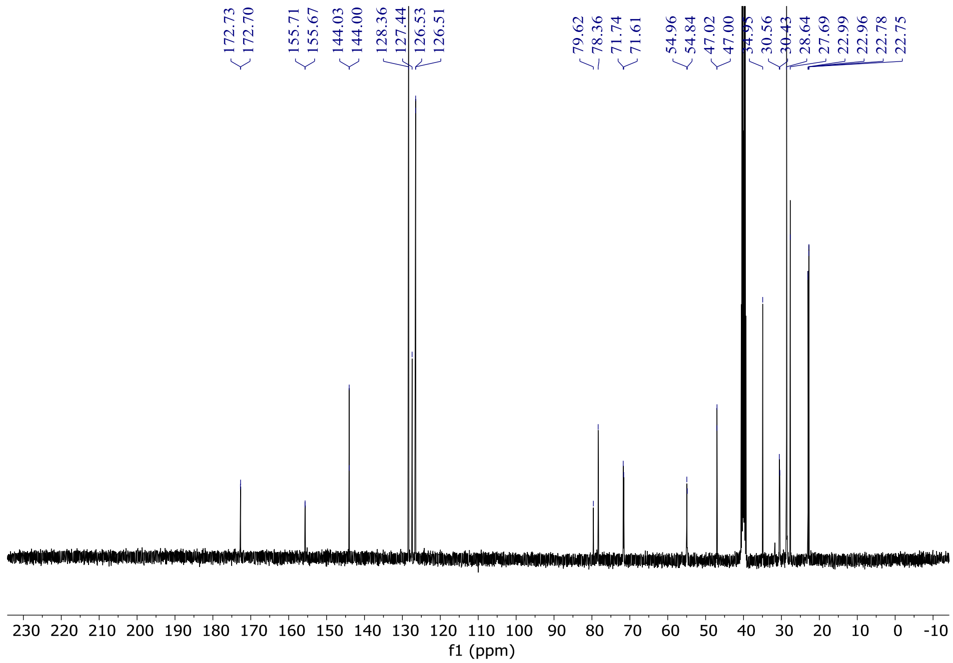

*tert-Butyl ((2S)-1-((2-hydroxy-2-phenylethyl)amino)-1-oxo-4-phenylbutan-2-yl)carbamate **(24b)**.*
^{1}H NMR in DMSO-d$_6$

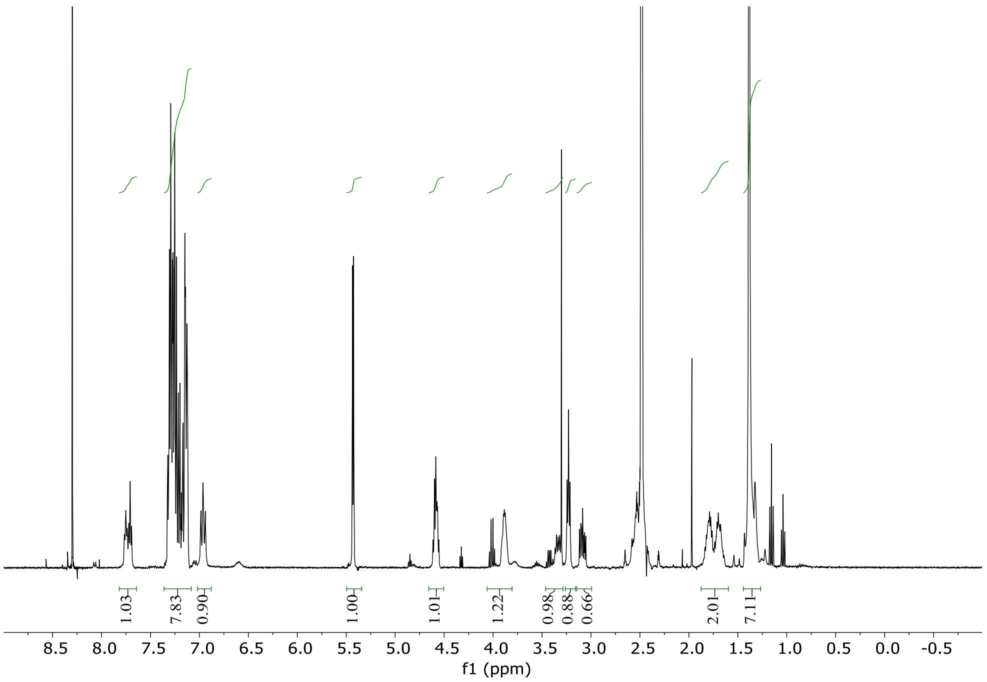

^{13}C NMR in DMSO-d$_6$

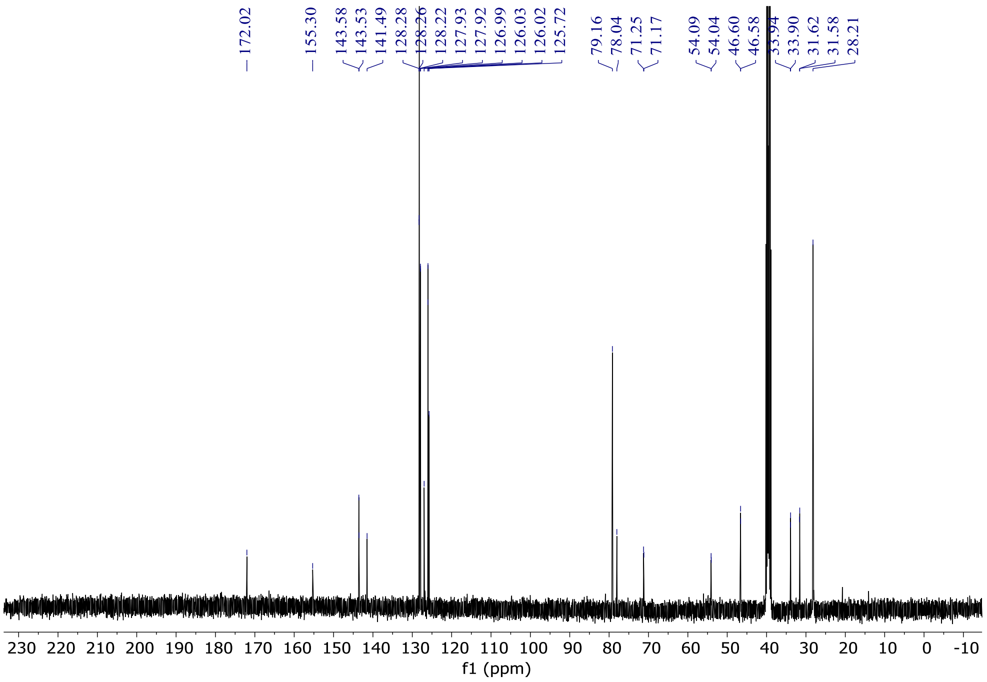

*tert-Butyl ((2S)-1-((2-hydroxy-2-phenylethyl)amino)-1-oxo-3-phenylpropan-2-yl)carbamate (**24c**).*
^{1}H NMR in $CDCl_3$

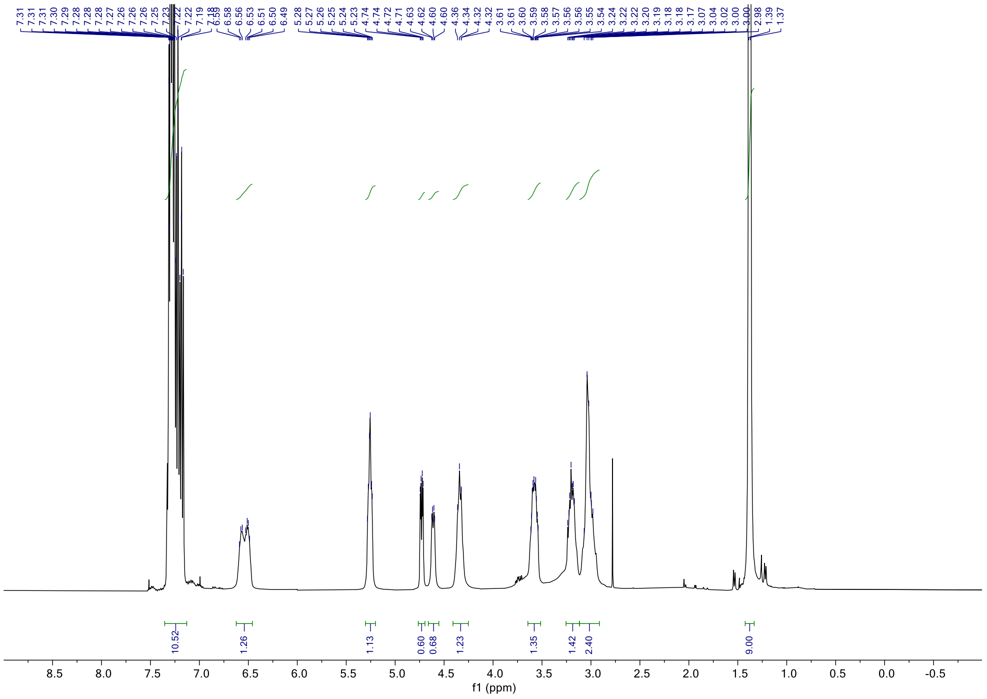

^{13}C NMR in $CDCl_3$

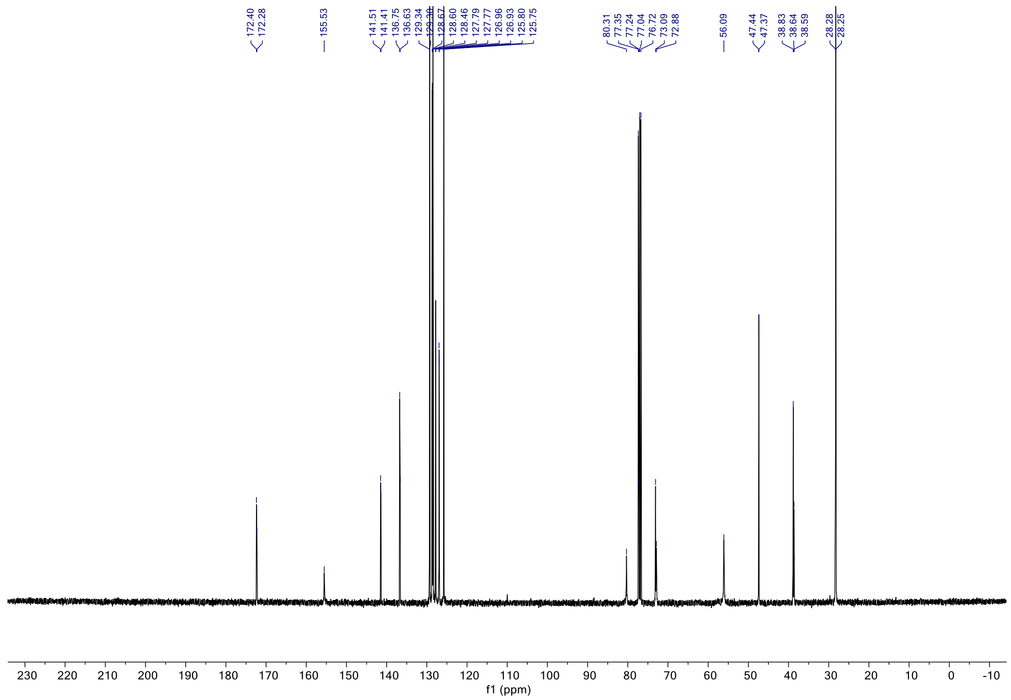

*tert-Butyl ((1S)-2-((2-hydroxy-2-phenylethyl)amino)-2-oxo-1-(p-tolyl)ethyl)carbamate (**24d**).*

^{1}H NMR in $CDCl_3$

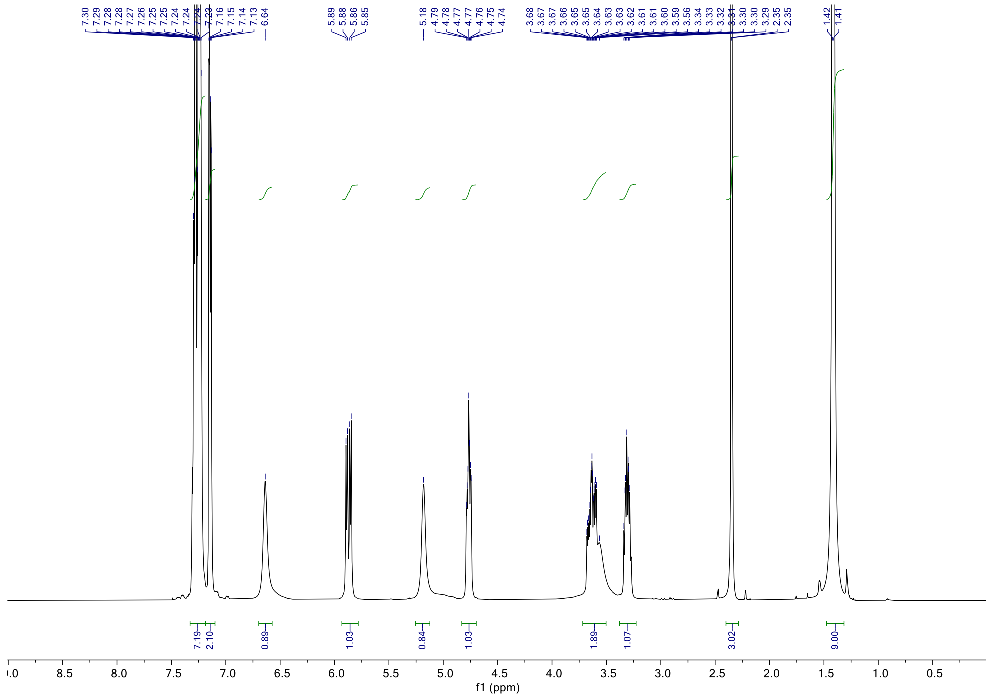

^{13}C NMR in $CDCl_3$

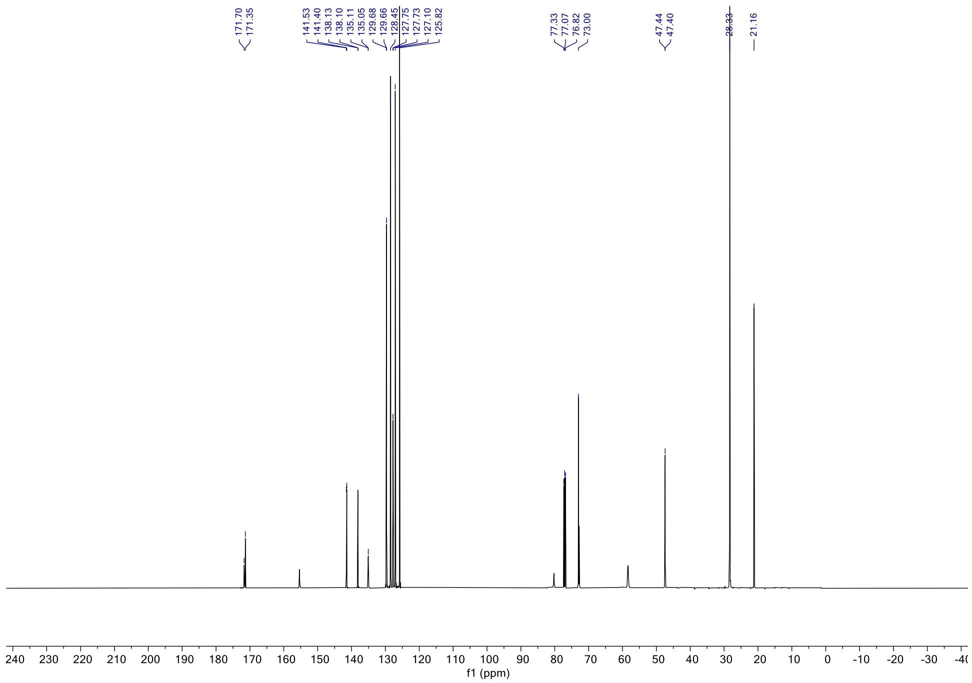

*tert-Butyl ((2S)-1-((2-hydroxy-2-phenylethyl)amino)-4-methyl-1-oxopentan-2-yl)carbamate (**24e**).*
^{1}H NMR in $CDCl_3$

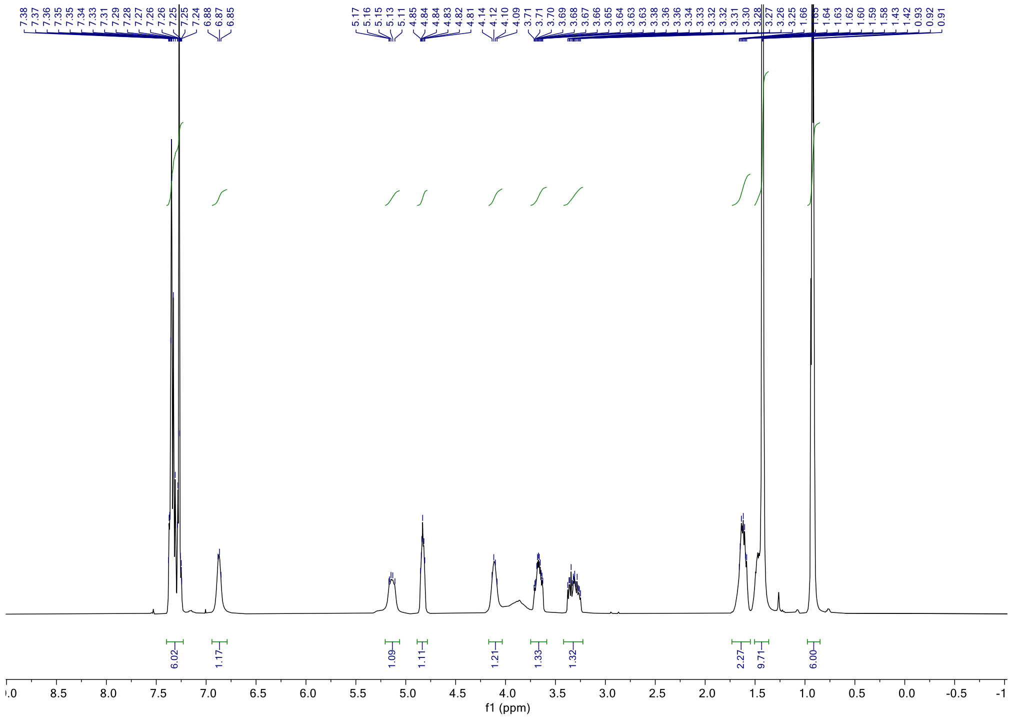

^{13}C NMR in $CDCl_3$

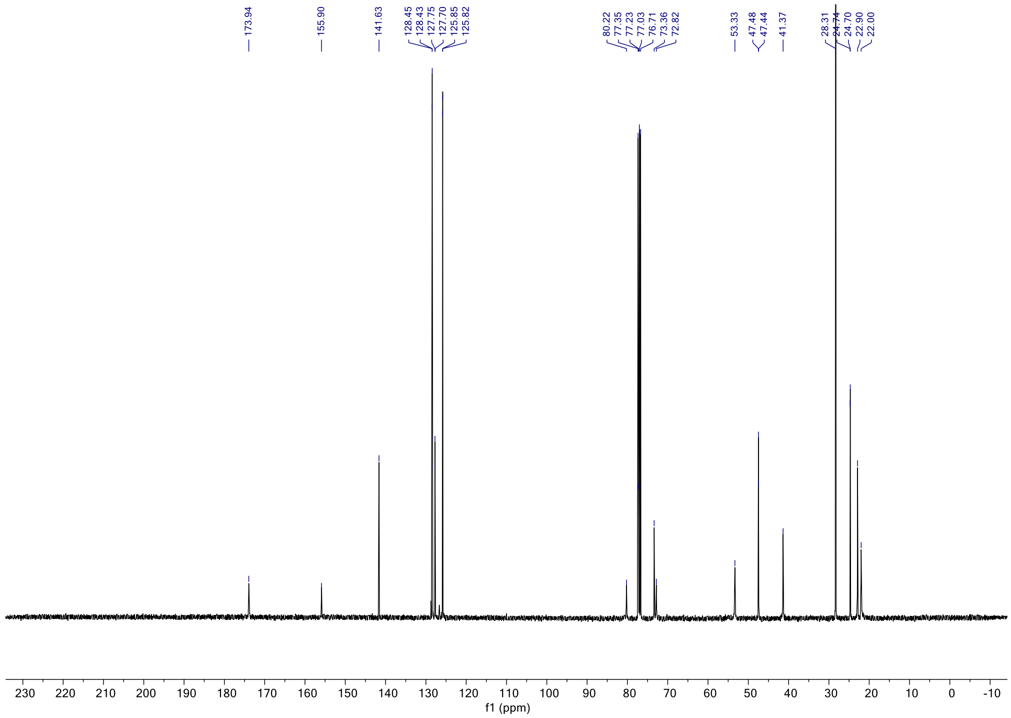

COSY in $CDCl_3$

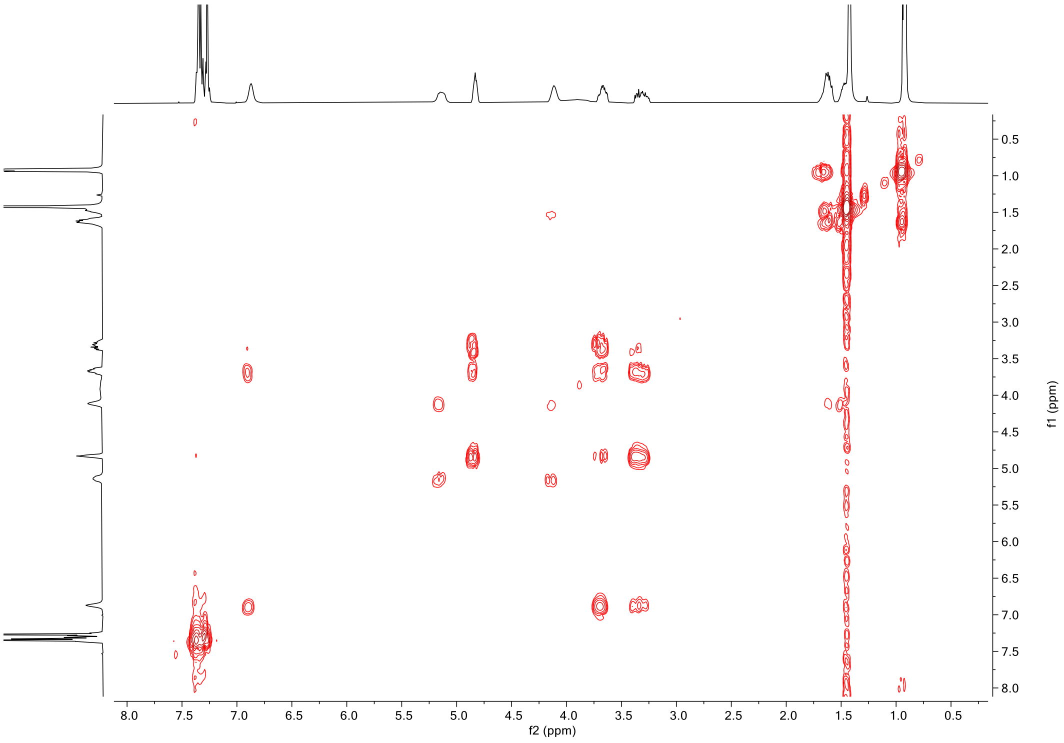

HSQC in $CDCl_3$

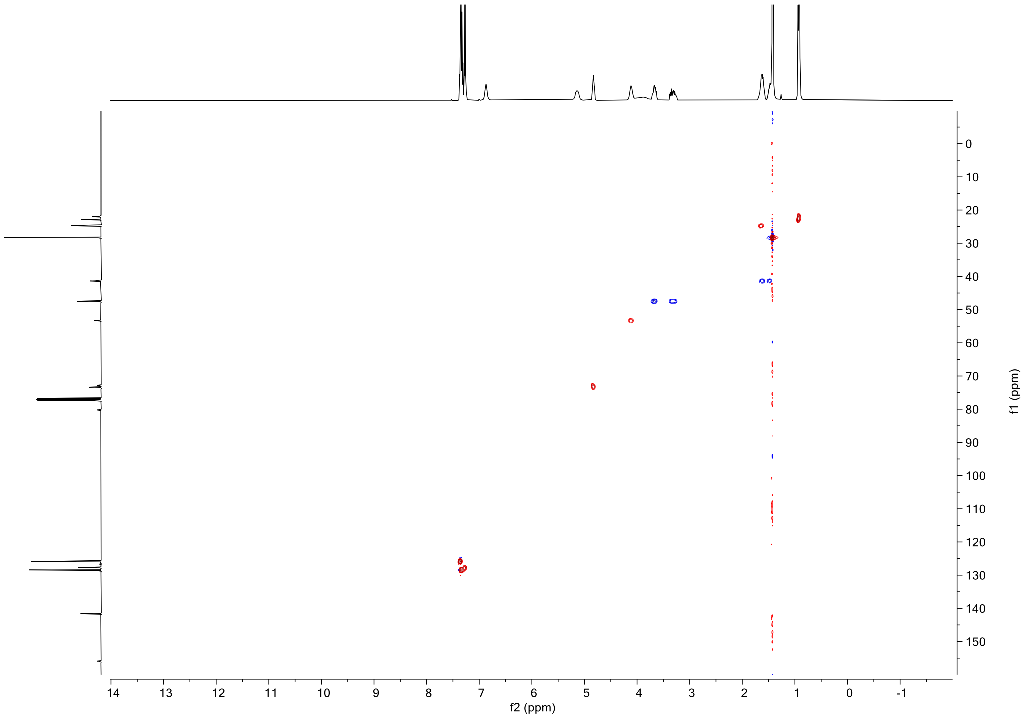

*Methyl (S)-2-((S)-2-((tert-butoxycarbonyl)amino)-3-(4-methoxyphenyl)propanamido)-3-(pyridin-2-yl)propanoate (**27**).*

^{1}H NMR in $CDCl_3$

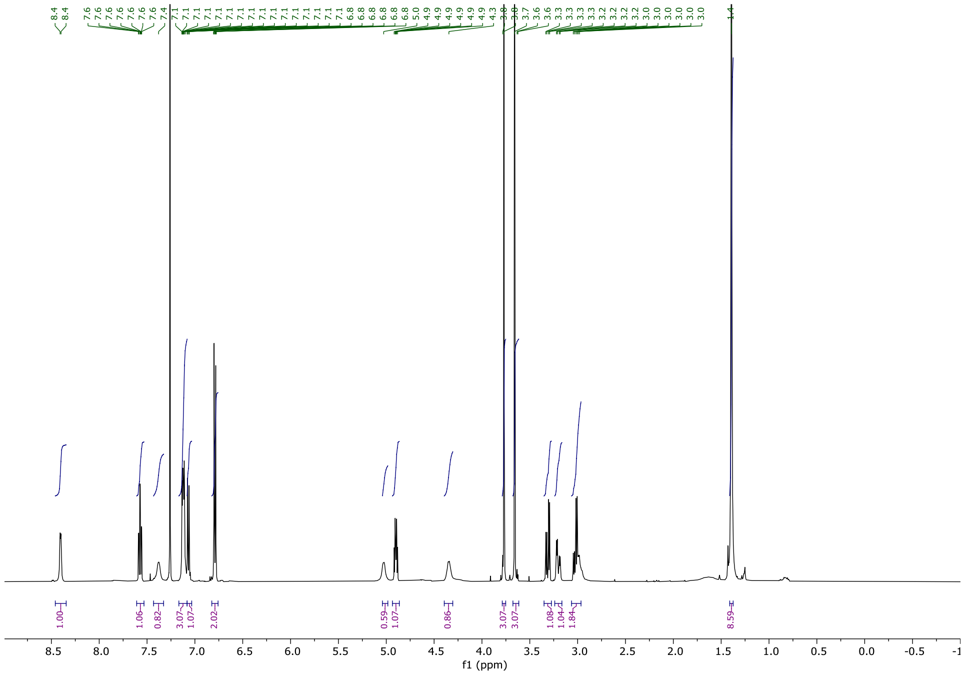

^{13}C NMR in $CDCl_3$

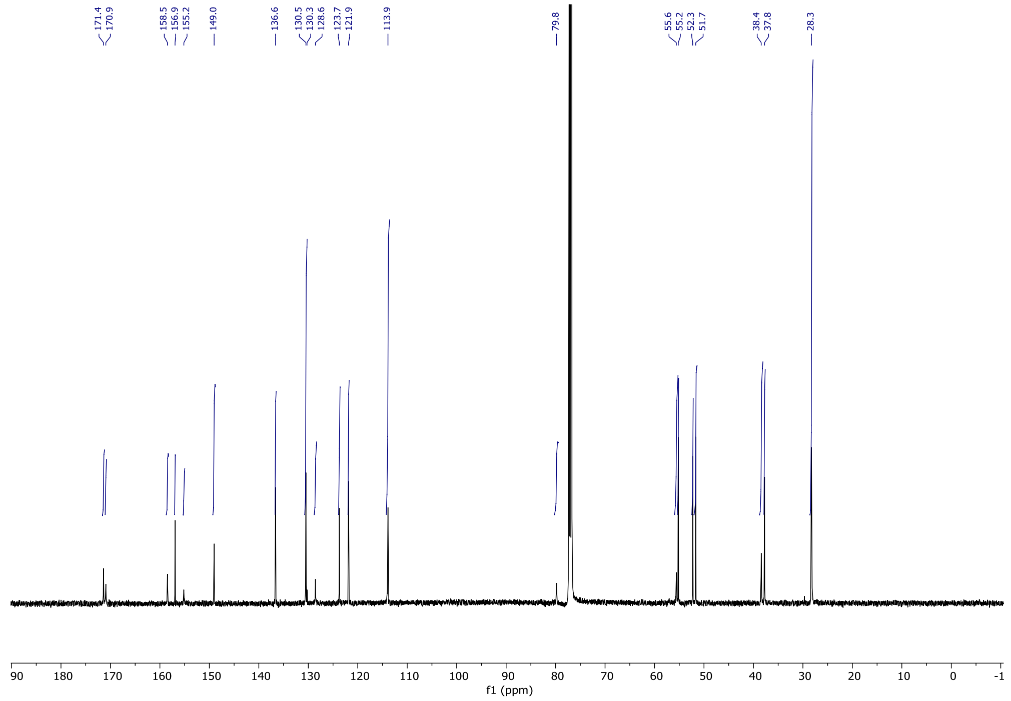

COSY in $CDCl_3$

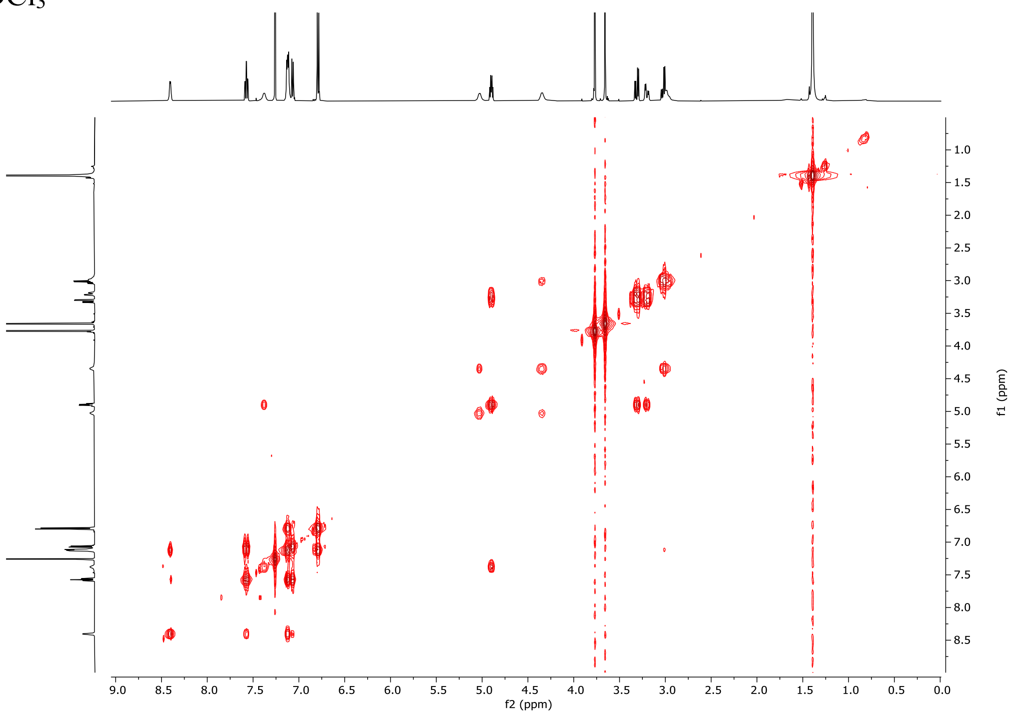

HSQC in $CDCl_3$

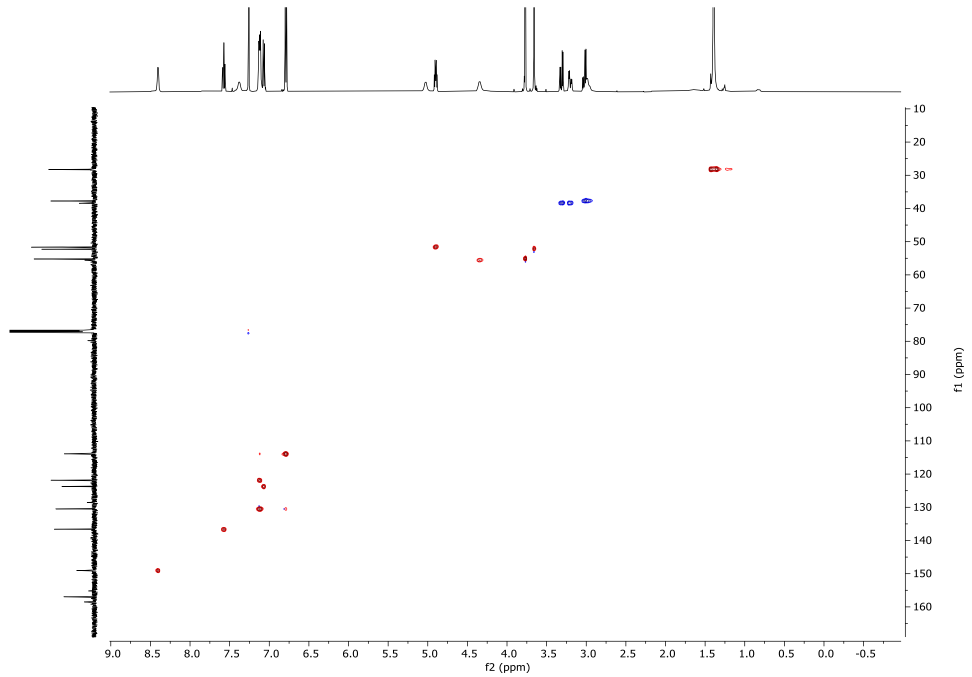

*tert-Butyl ((2S)-1-(((2S)-1-((2-hydroxy-2-phenylethyl)amino)-5-methyl-1-oxohexan-2-yl)amino)-3-(4-methoxyphenyl)-1-oxopropan-2-yl)carbamate (**26a**).*

^{1}H NMR in DMSO-d_6

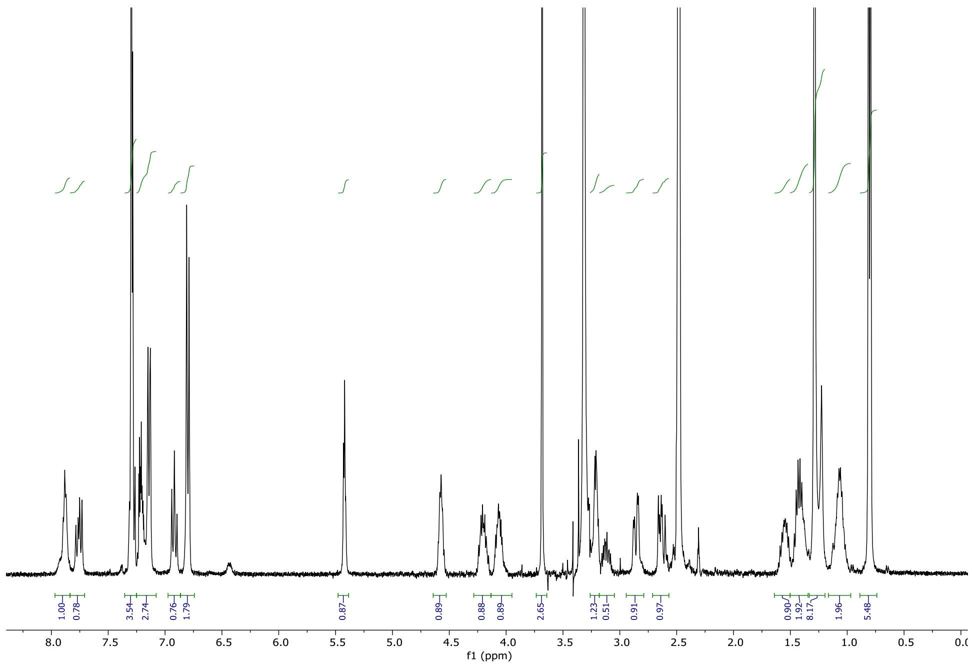

^{13}C NMR in DMSO-d$_6$

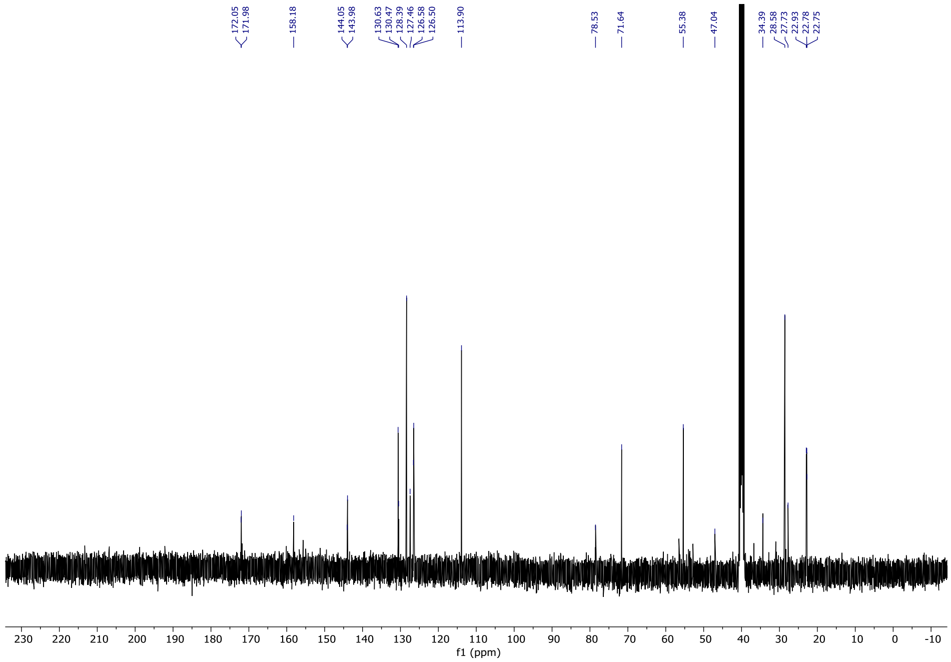

*tert-Butyl ((2S)-1-(((2S)-1-((2-hydroxy-2-phenylethyl)amino)-1-oxo-4-phenylbutan-2-yl)amino)-3-(4-methoxyphenyl)-1-oxopropan-2-yl)carbamate (**26b**).*

^{1}H NMR in DMSO-d$_6$

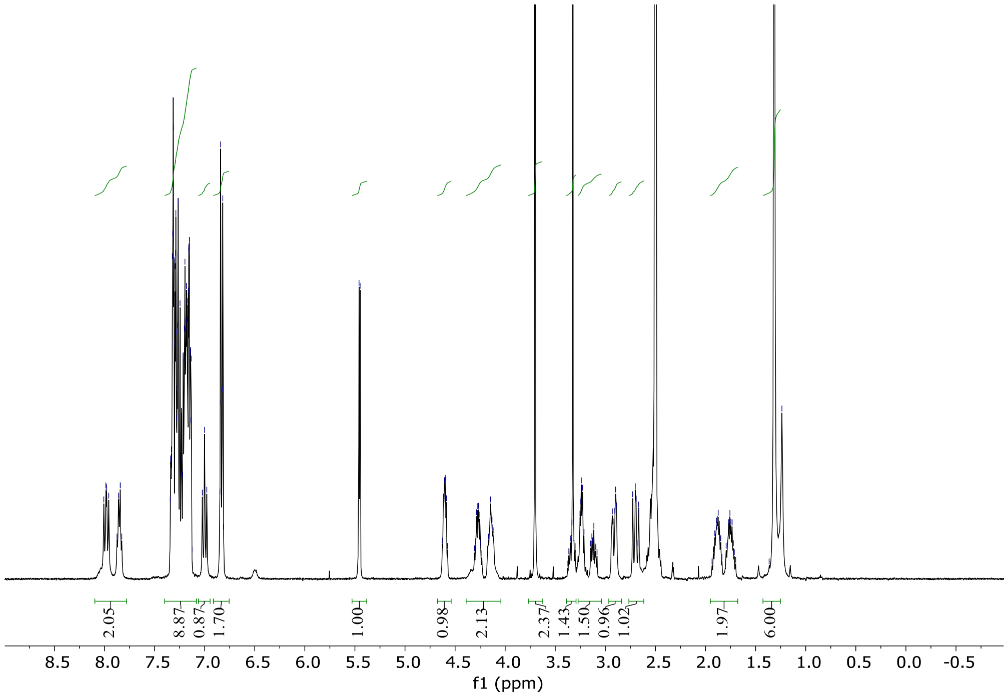

^{13}C NMR in DMSO-d$_6$

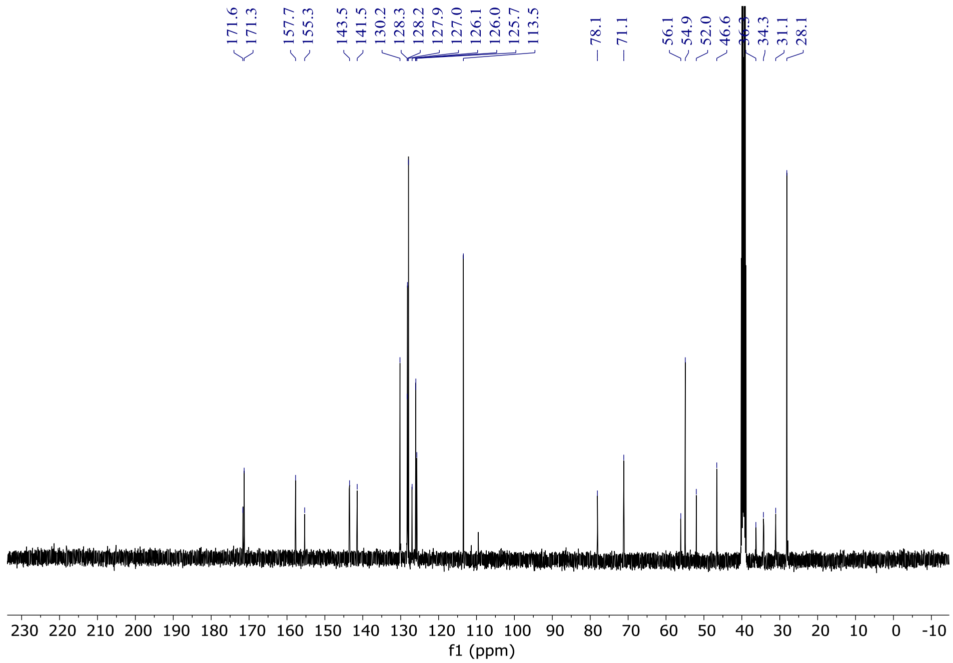

*tert-Butyl ((2S)-1-(((2S)-1-((2-hydroxy-2-phenylethyl)amino)-1-oxo-3-phenylpropan-2-yl)amino)-3-(4-methoxyphenyl)-1-oxopropan-2-yl)carbamate (**26c**).*

^{1}H NMR in $CDCl_3$

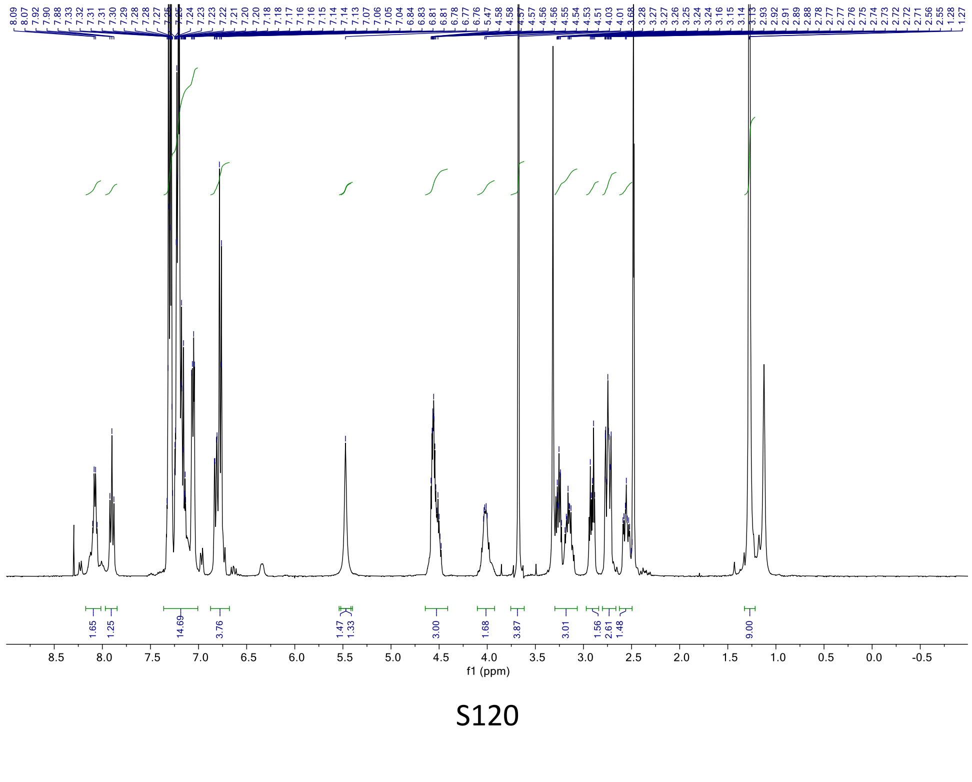

^{13}C NMR in $CDCl_3$

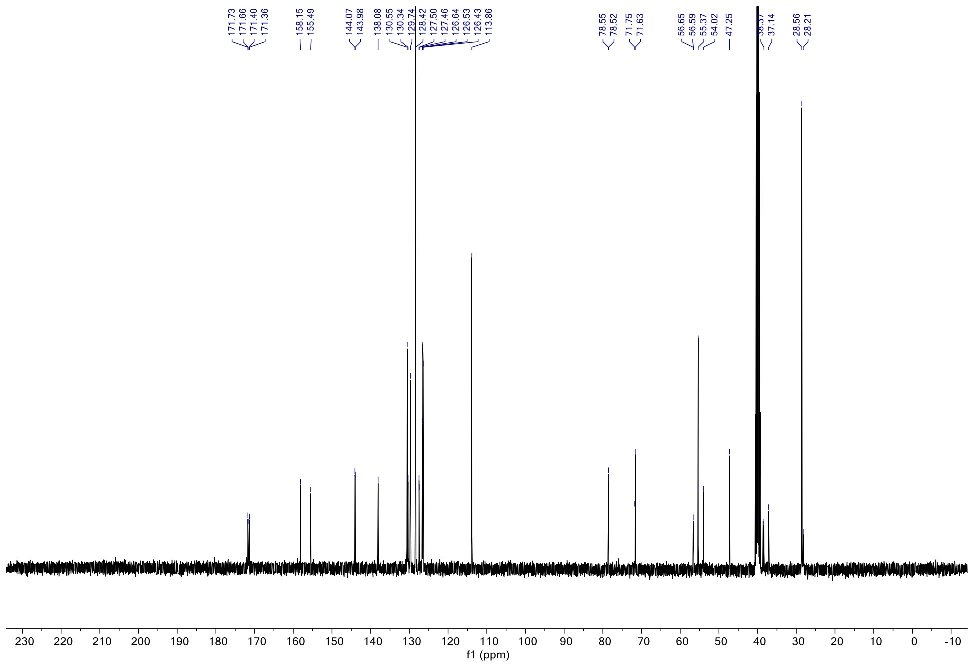

tert-Butyl ((2S)-1-(((1S)-2-((2-hydroxy-2-phenylethyl)amino)-2-oxo-1-(p-tolyl)ethyl)amino)-3-(4-methoxyphenyl)-1-oxopropan-2-yl)carbamate ***(26d)***.

^{1}H NMR in DMSO-d_6

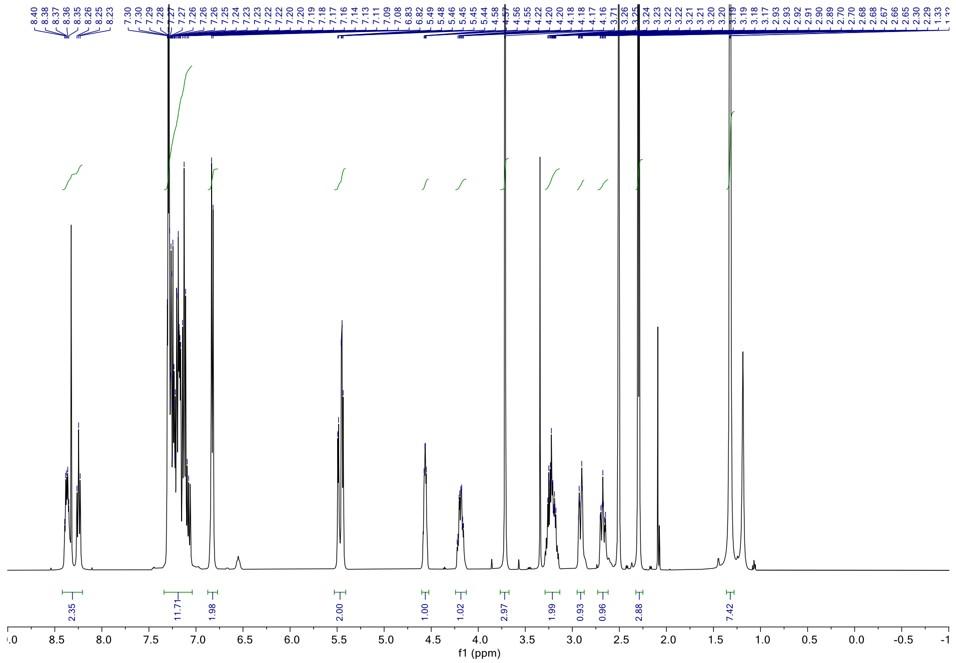

^{13}C NMR in DMSO-d$_6$

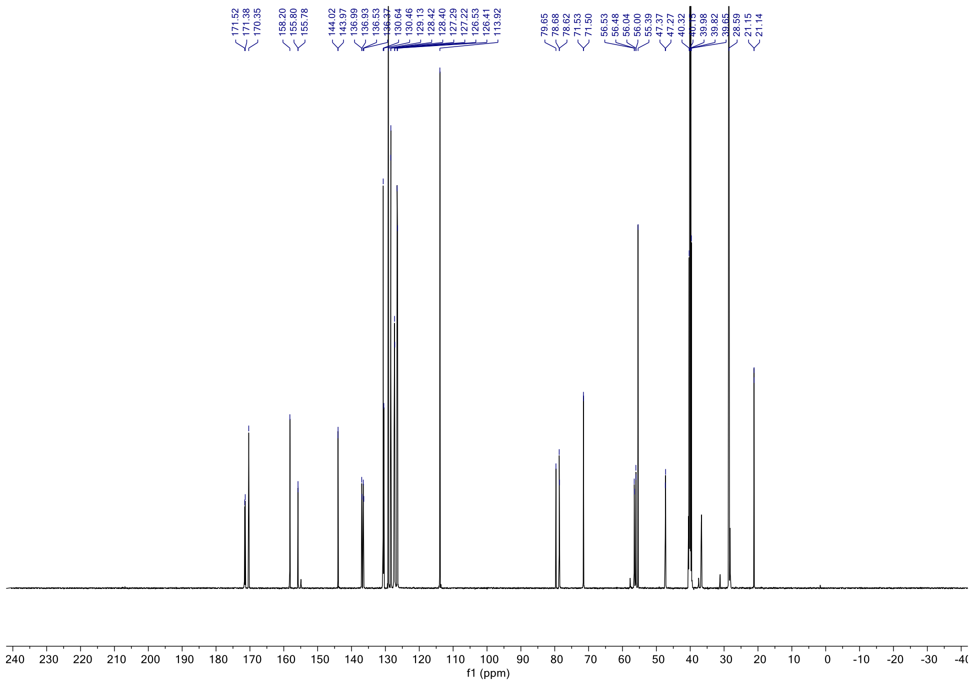

tert-Butyl ((2S)-1-(((2S)-1-((2-hydroxy-2-phenylethyl)amino)-4-methyl-1-oxopentan-2-yl)amino)-3-(4-methoxyphenyl)-1-oxopropan-2-yl)carbamate ***(26e)***.

^{1}H NMR in DMSO-d$_6$

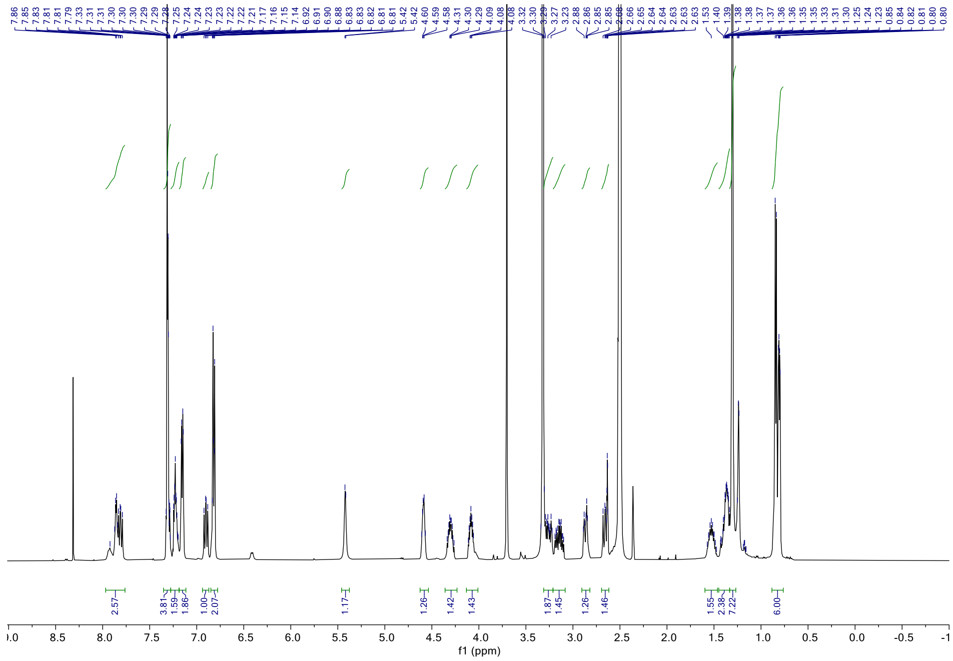

^{13}C NMR in DMSO-d_6

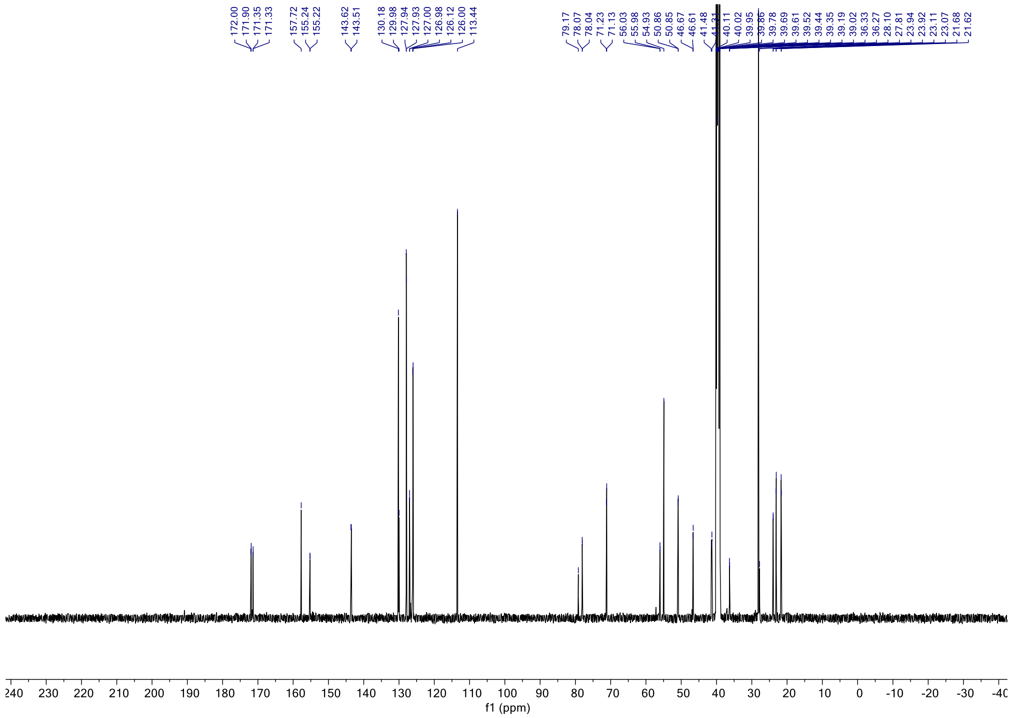

COSY in DMSO-d_6

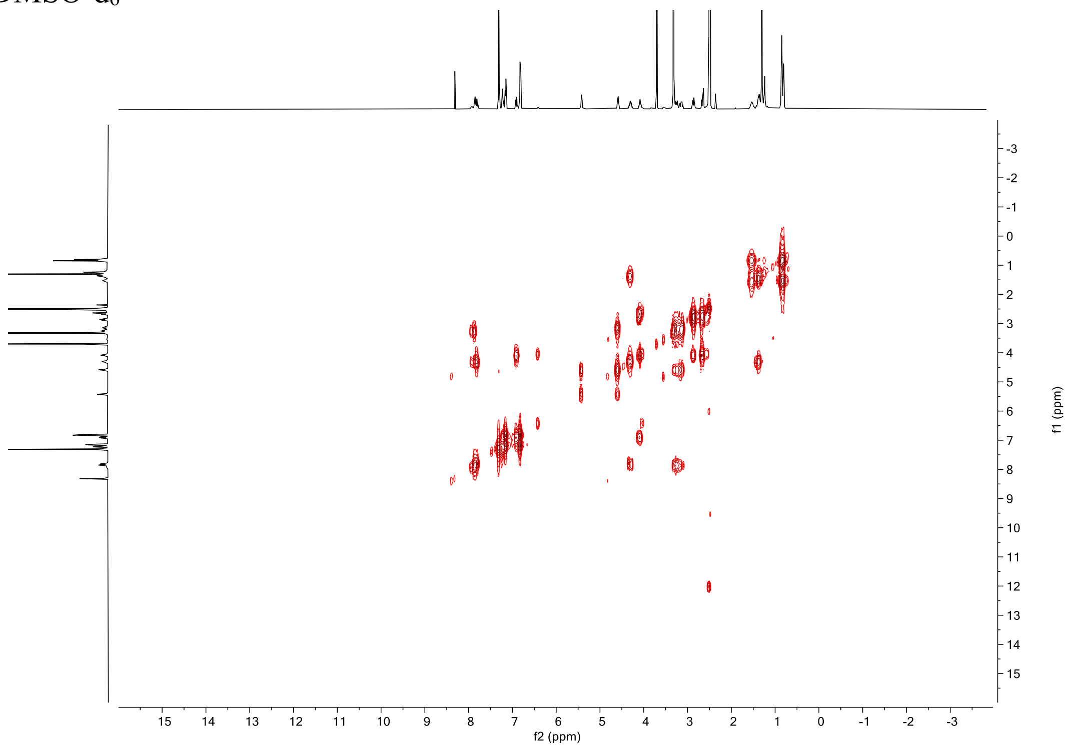

HSQC in DMSO-d$_6$

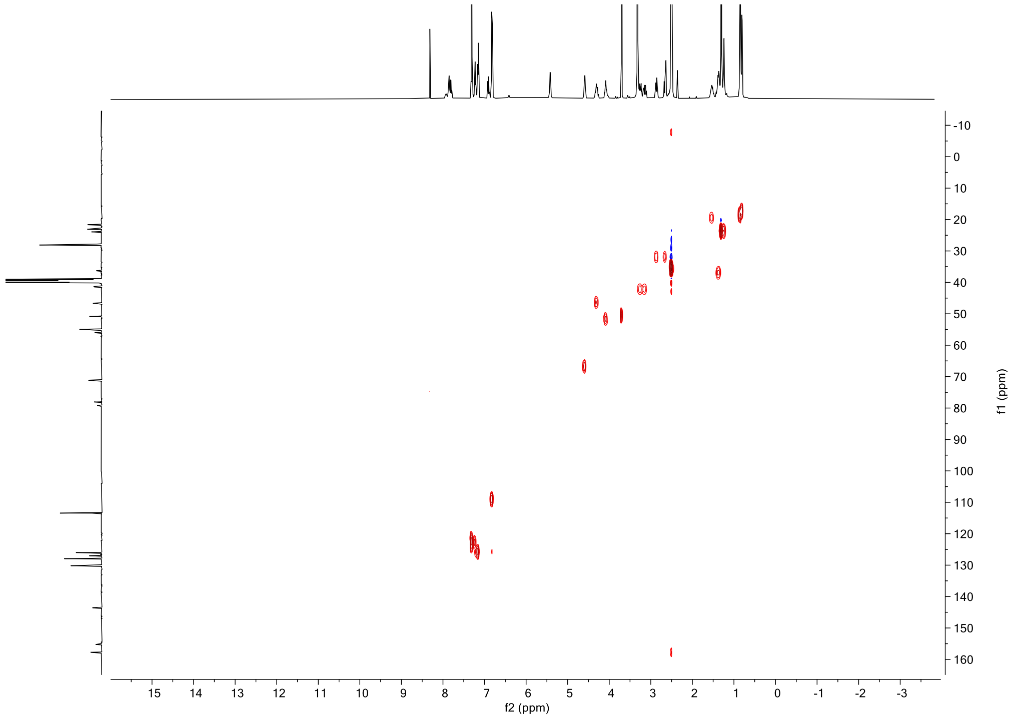

*tert-Butyl ((2S)-1-(((2S)-1-((2-hydroxy-2-phenylethyl)amino)-1-oxo-3-(pyridin-2-yl)propan-2-yl)amino)-3-(4-methoxyphenyl)-1-oxopropan-2-yl)carbamate (**26g**).*

^{1}H NMR in DMSO-d$_6$

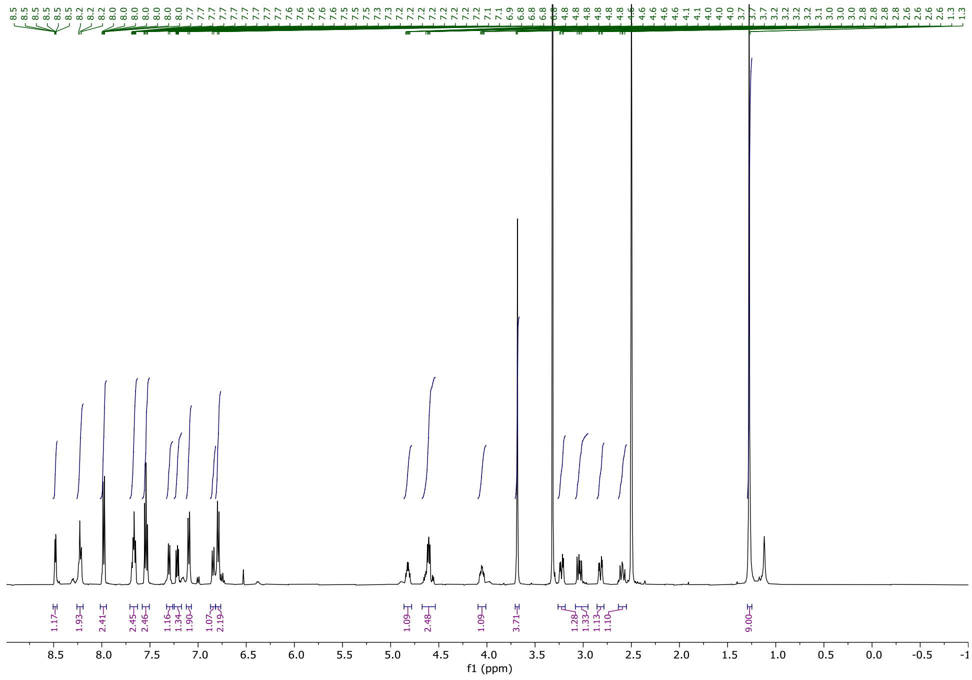

^{13}C NMR in DMSO-d_6

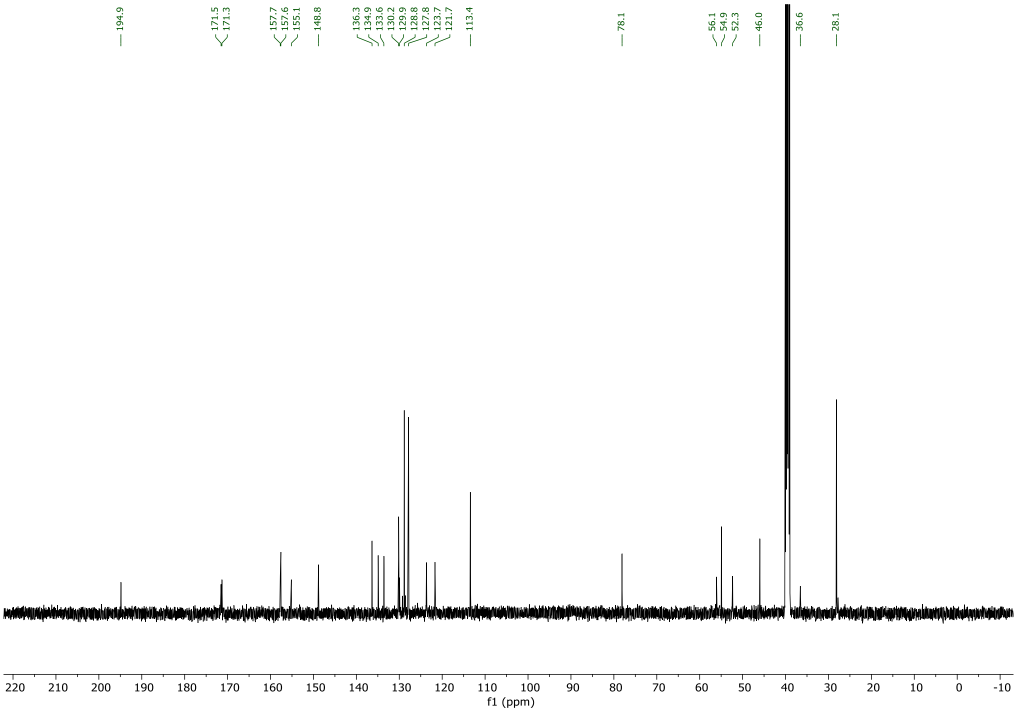

COSY in DMSO-d_6

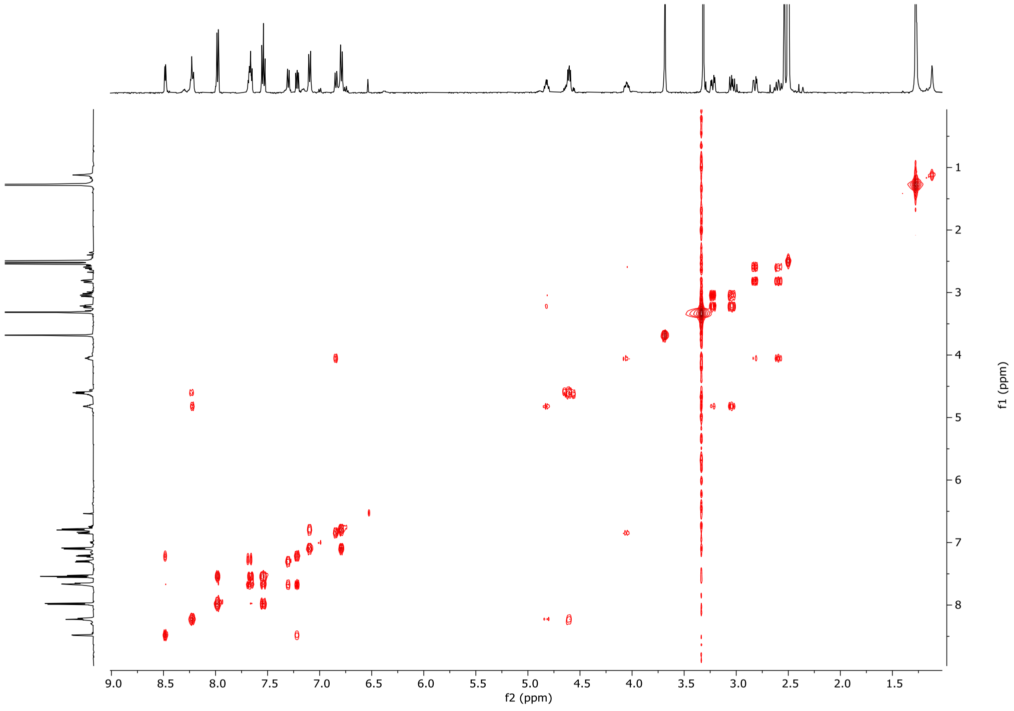

HSQC in DMSO-d6

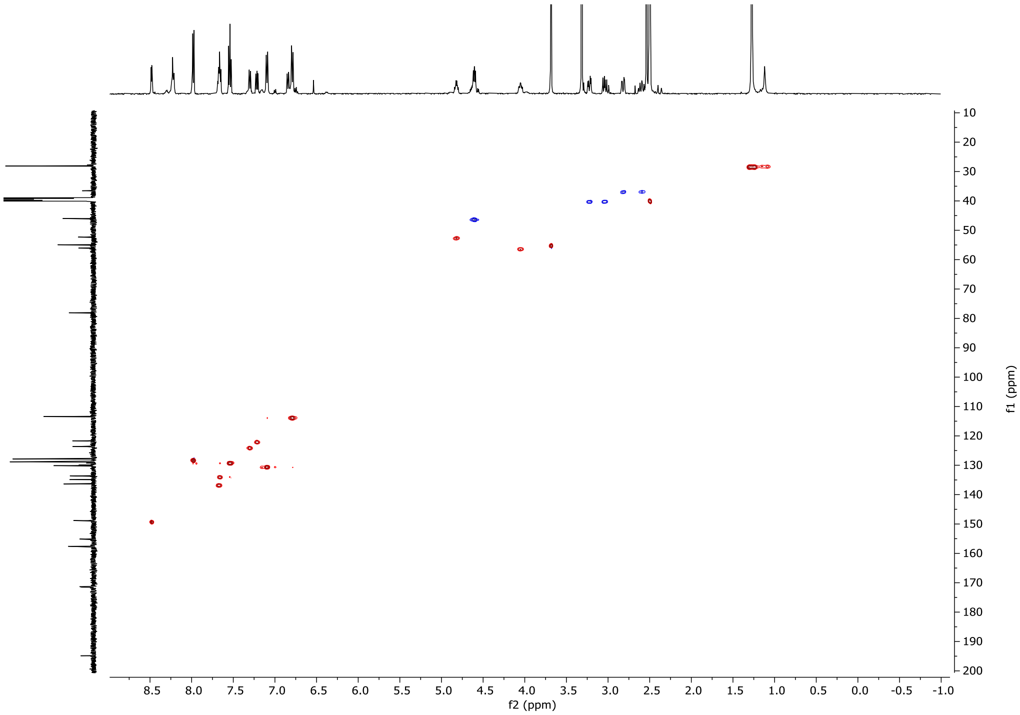

*(S)-2-((S)-2-Acetamido-3-(4-methoxyphenyl)propanamido)-5-methyl-N-(2-oxo-2-phenylethyl)hexanamide (**5a**).*

^{1}H NMR in DMSO-d6

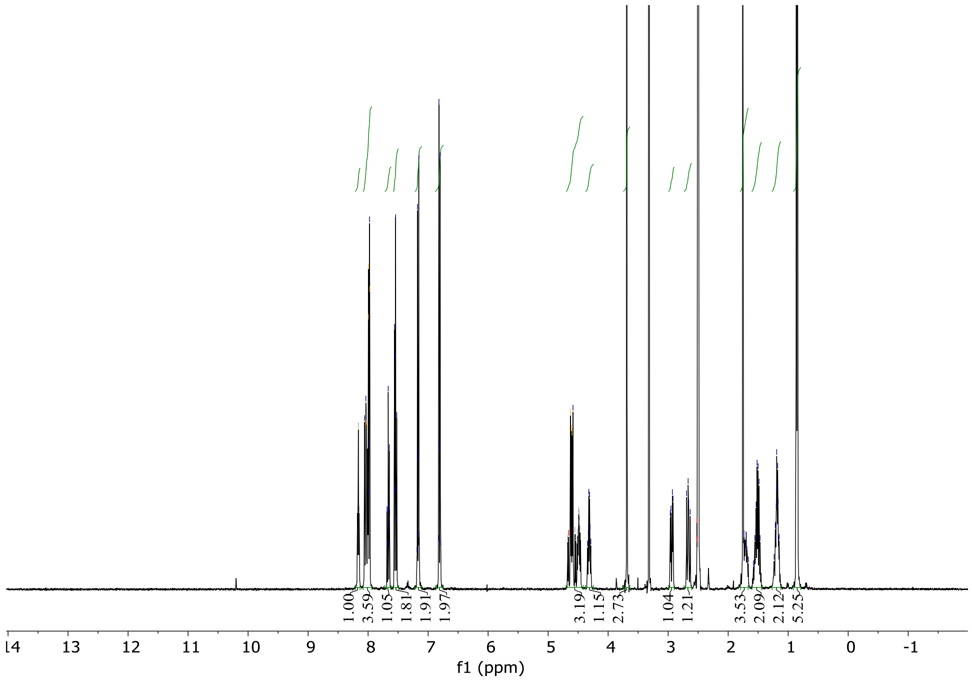

^{13}C NMR in DMSO-d$_6$

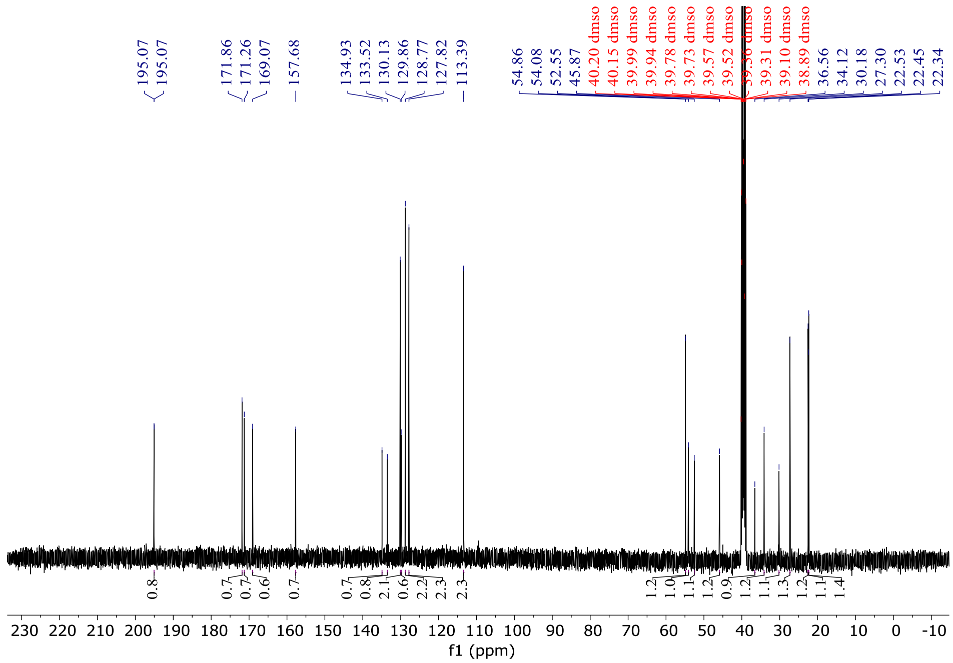

*(S)-2-((S)-2-Acetamido-3-(4-methoxyphenyl)propanamido)-N-(2-oxo-2-phenylethyl)-4-phenylbutanamide (**5b**).*

^{1}H NMR in CDCl$_3$

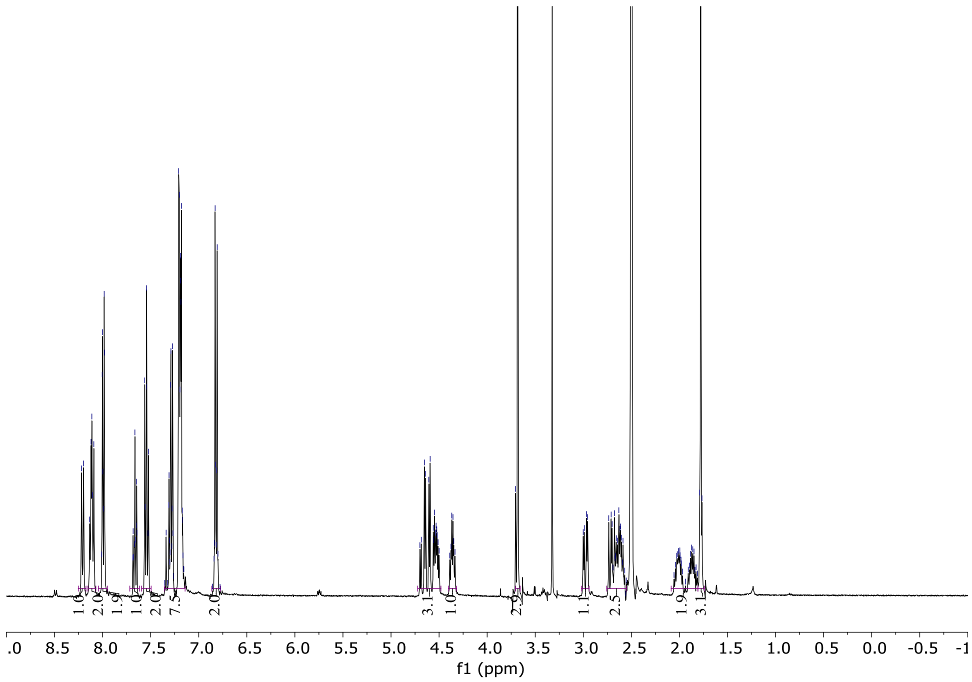

^{13}C NMR in $CDCl_3$

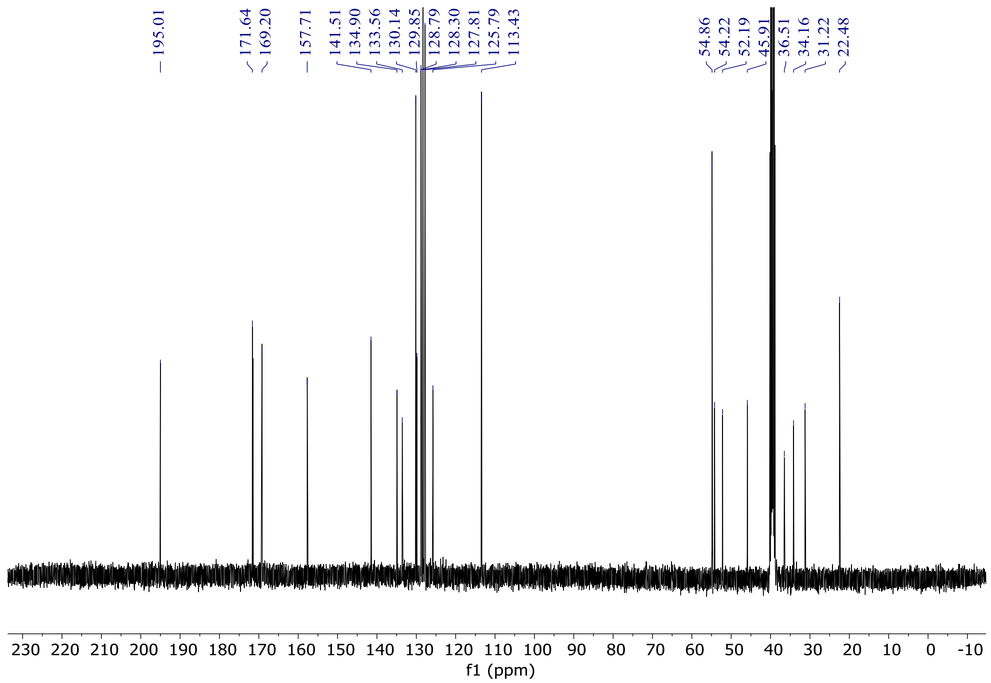

*(S)-2-Acetamido-3-(4-methoxyphenyl)-N-((S)-1-oxo-1-((2-oxo-2-phenylethyl)amino)-3-phenylpropan-2-yl)propanamide (**5c**).*

^{1}H NMR in DMSO-d_6

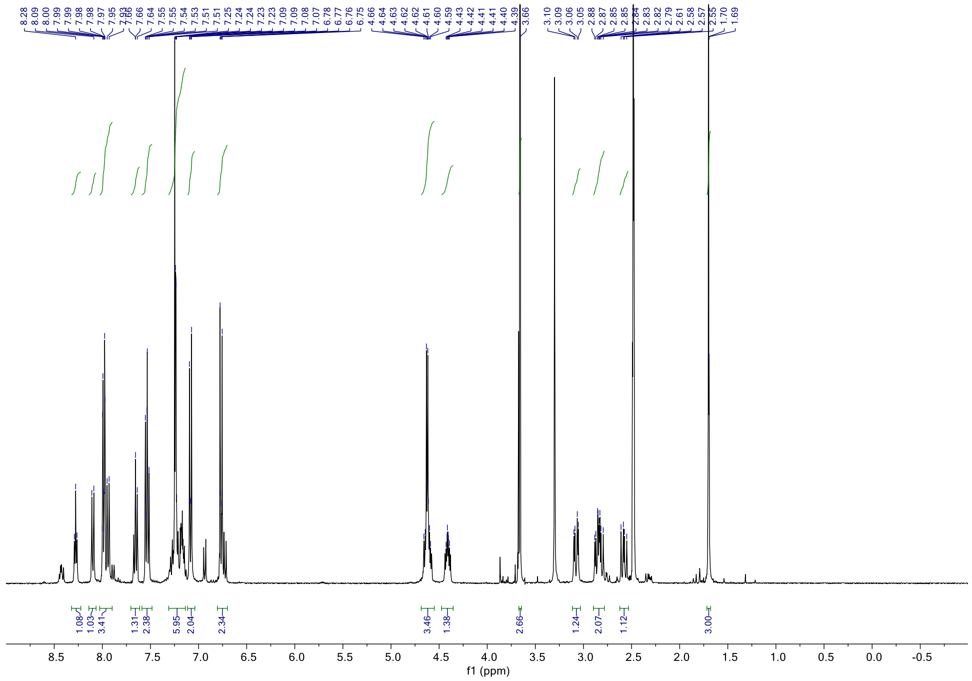

^{13}C NMR in DMSO-d$_6$

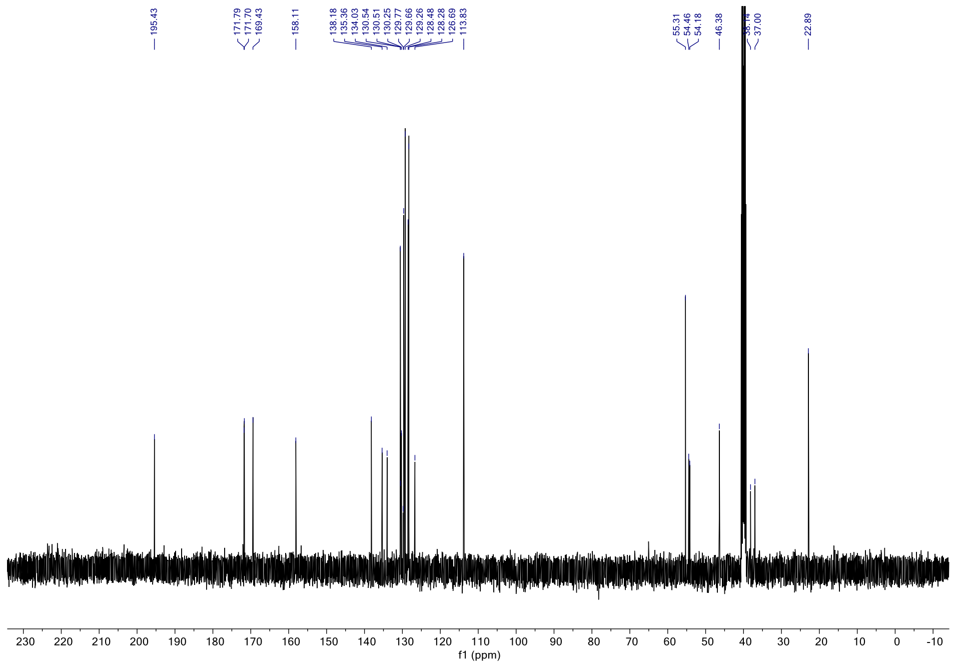

*(S)-2-Acetamido-3-(4-methoxyphenyl)-N-((S)-2-oxo-2-((2-oxo-2-phenylethyl)amino)-1-(p-tolyl)ethyl)propanamide (**5d**).*

^1H NMR in DMSO-d$_6$

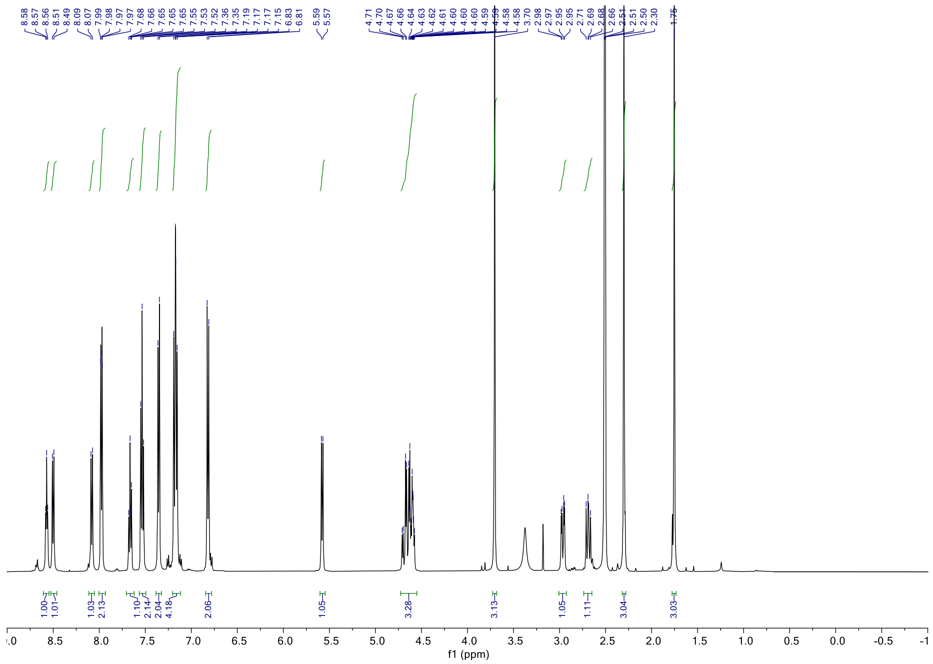

^{13}C NMR in DMSO-d_6

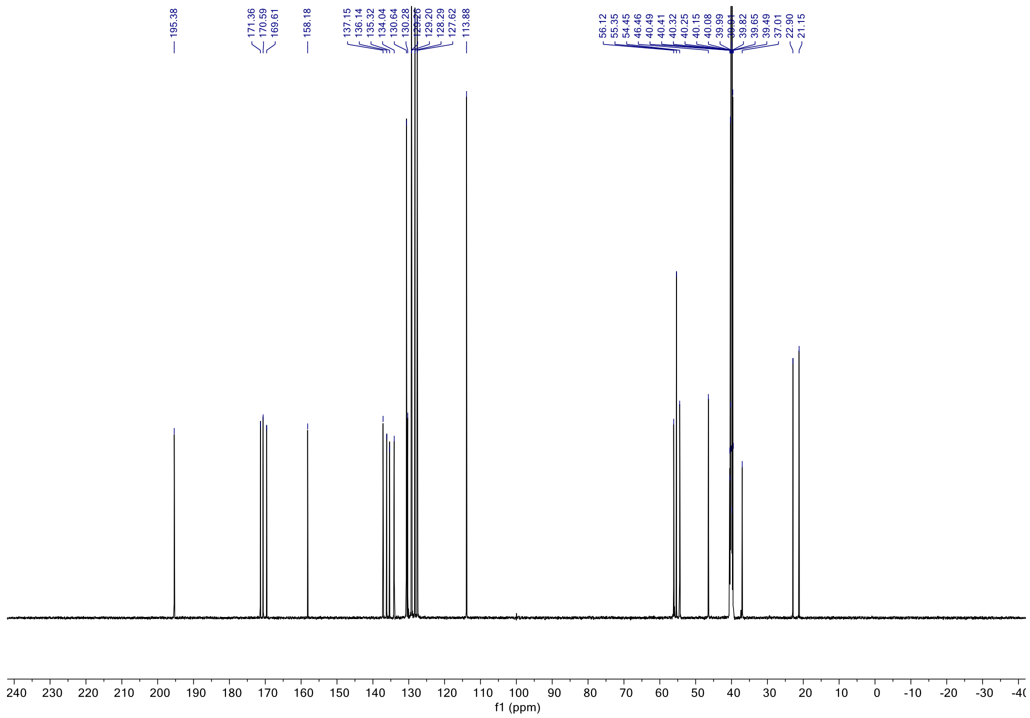

*(S)-2-((S)-2-Acetamido-3-(4-methoxyphenyl)propanamido)-4-methyl-N-(2-oxo-2-phenylethyl)pentanamide (**5e**).*

^{1}H NMR in $CDCl_3$

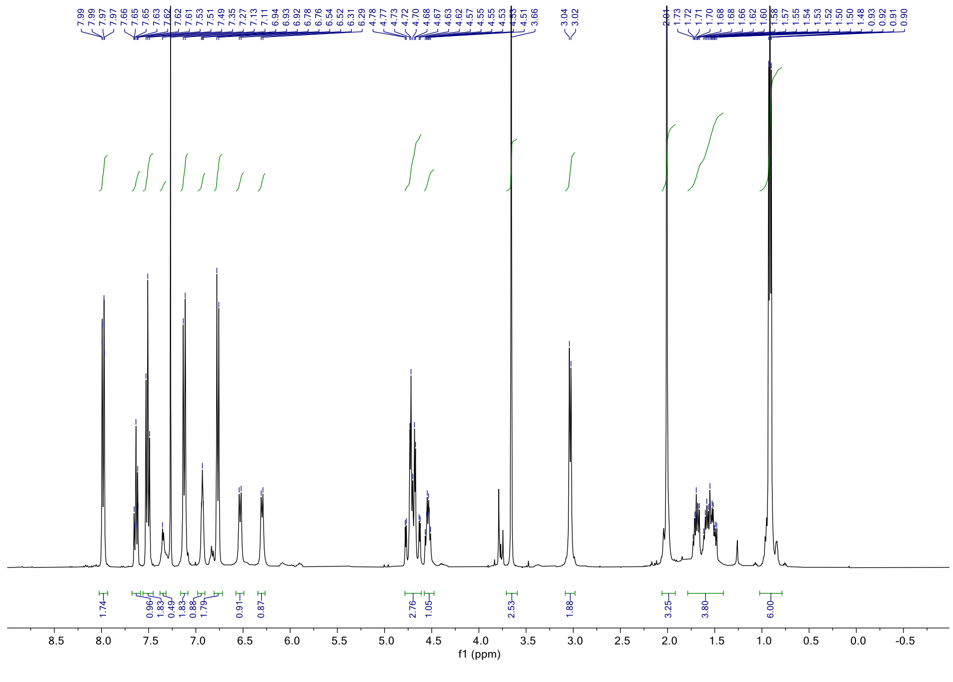

^{13}C NMR in $CDCl_3$

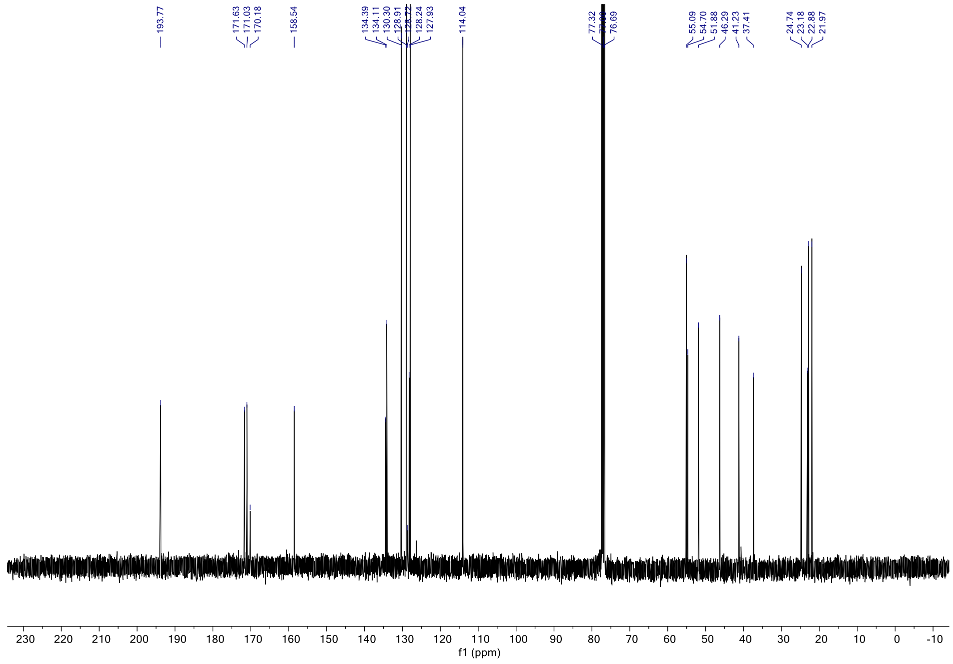

COSY in $CDCl_3$

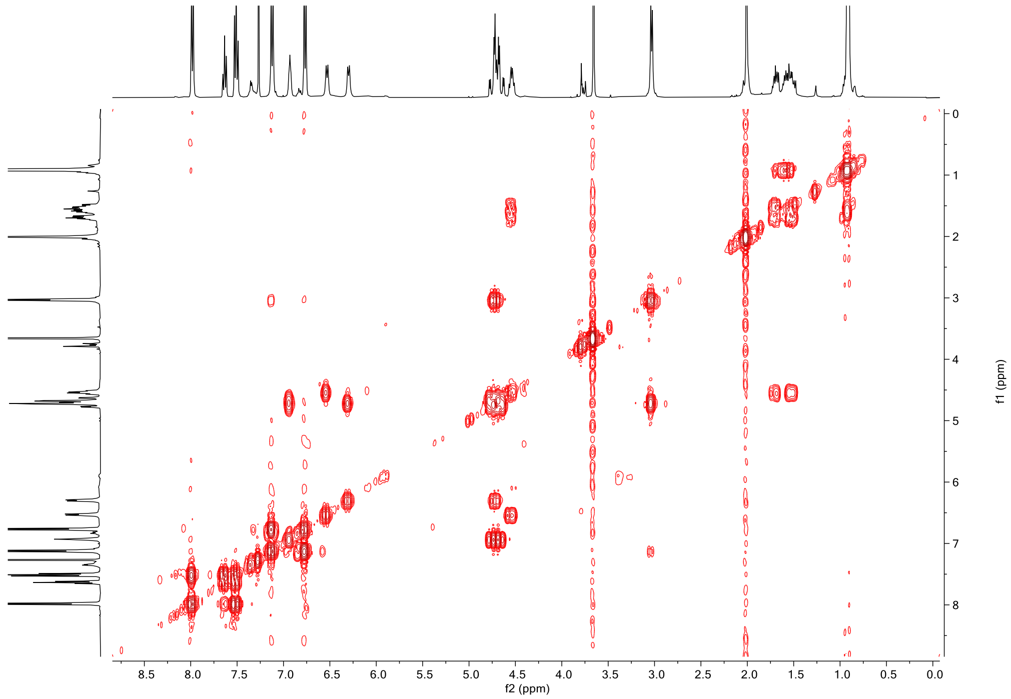

HSQC in $CDCl_3$

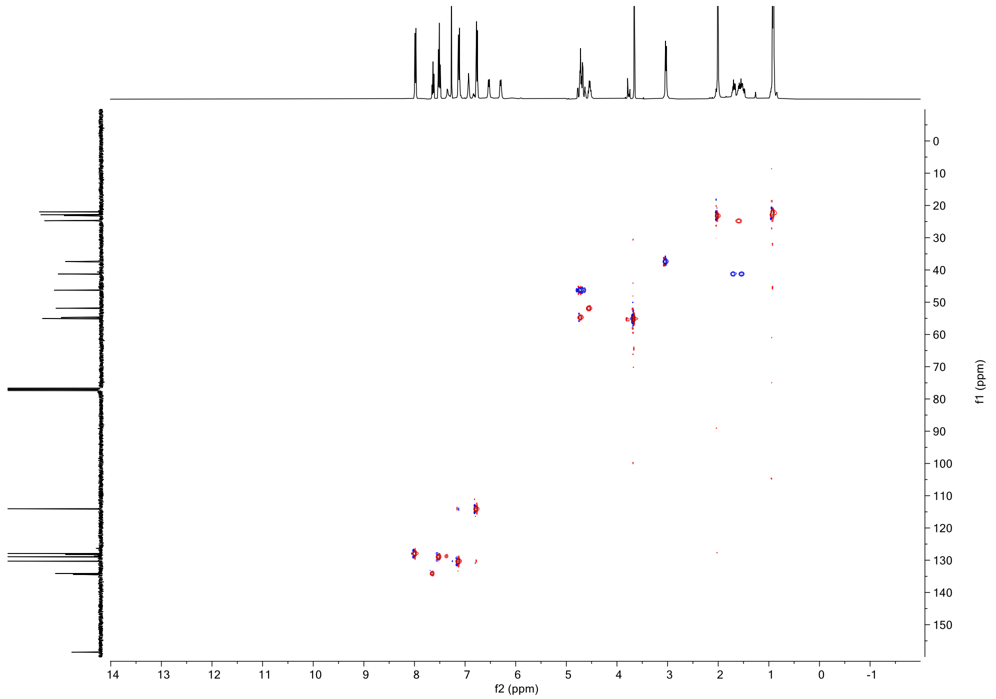

*(S)-3-(4-Methoxyphenyl)-N-((S)-1-oxo-1-((2-oxo-2-phenylethyl)amino)-3-phenylpropan-2-yl)-2-propionamidopropanamide (**5f**).*

^1H NMR in DMSO-d_6

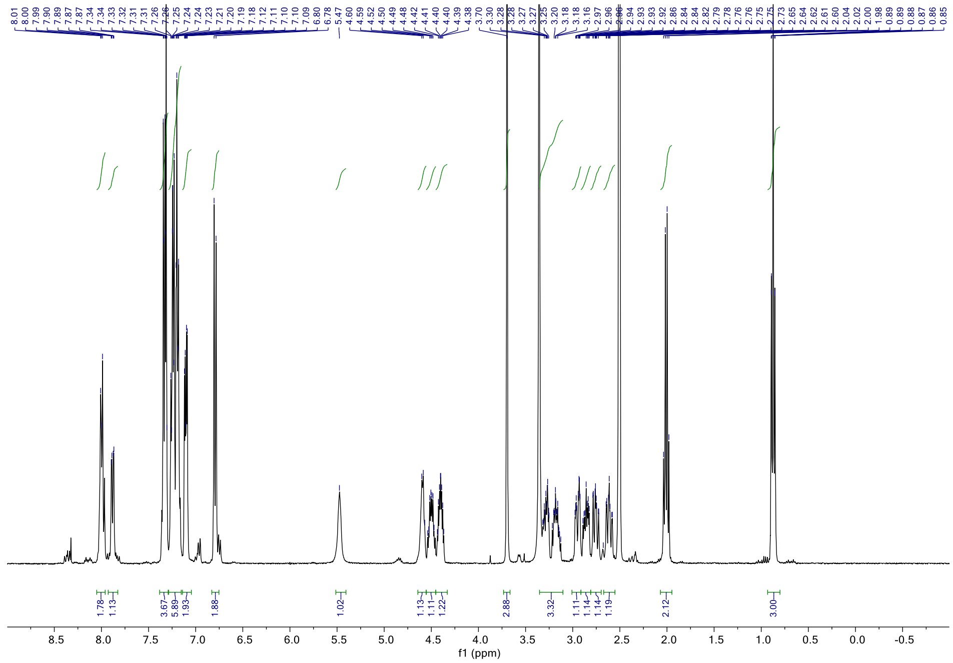

^{13}C NMR in DMSO-d$_6$

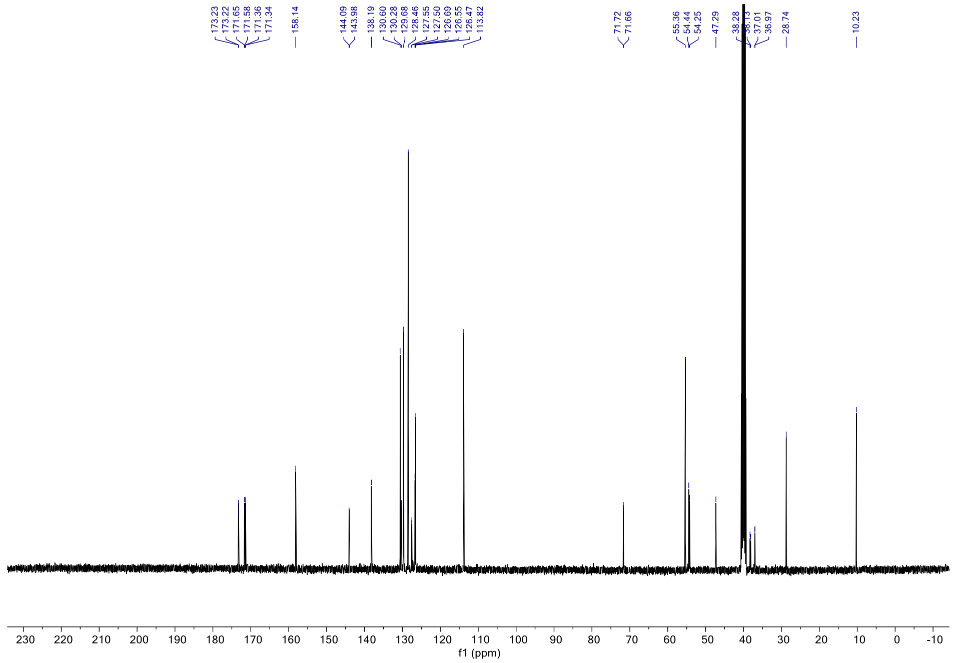

*(S)-2-Acetamido-3-(4-methoxyphenyl)-N-((S)-1-oxo-1-((2-oxo-2-phenylethyl)amino)-3-(pyridin-2-yl)propan-2-yl)propanamide (**5g**).*

^{1}H NMR in DMSO-d$_6$

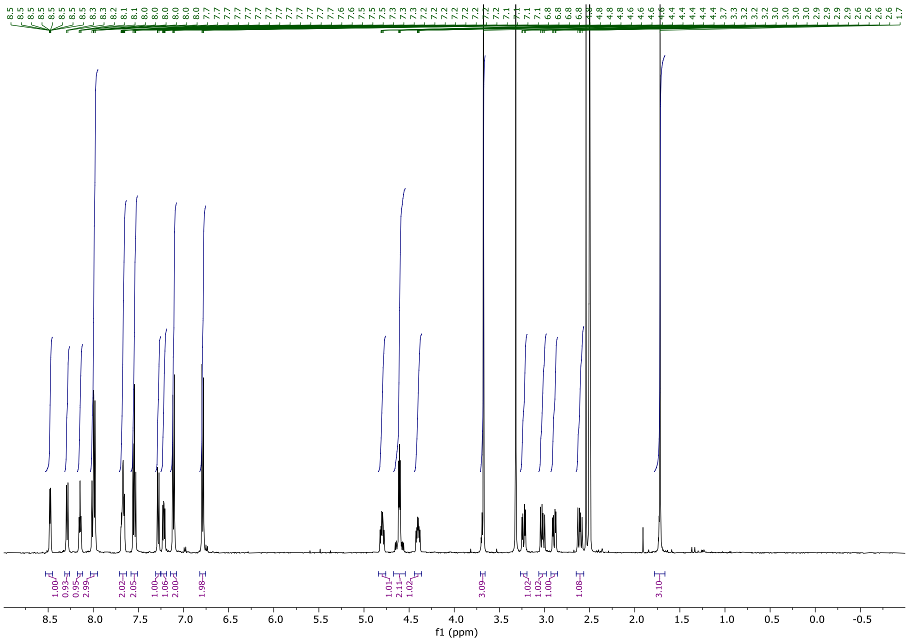

^{13}C NMR in DMSO-d$_6$

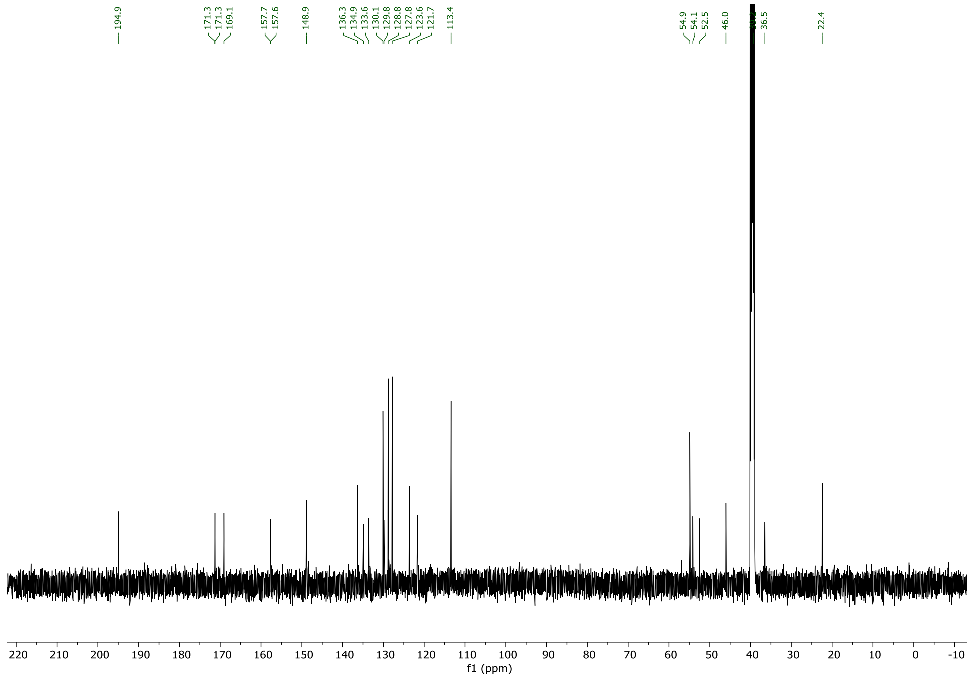

COSY in DMSO-d$_6$

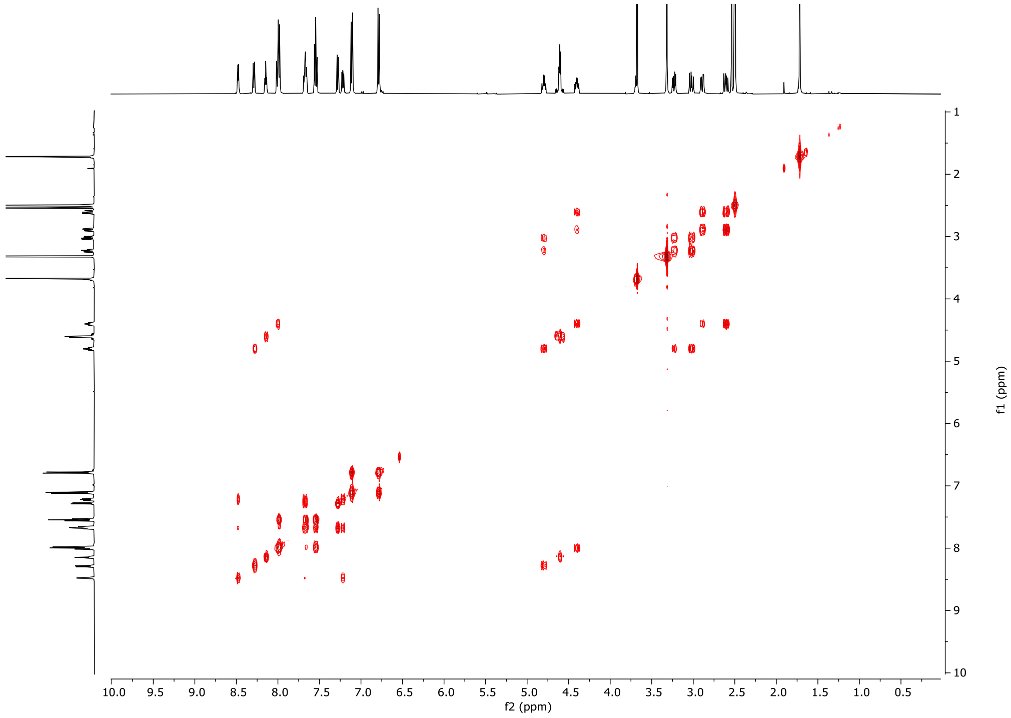

HSQC in DMSO-d$_6$

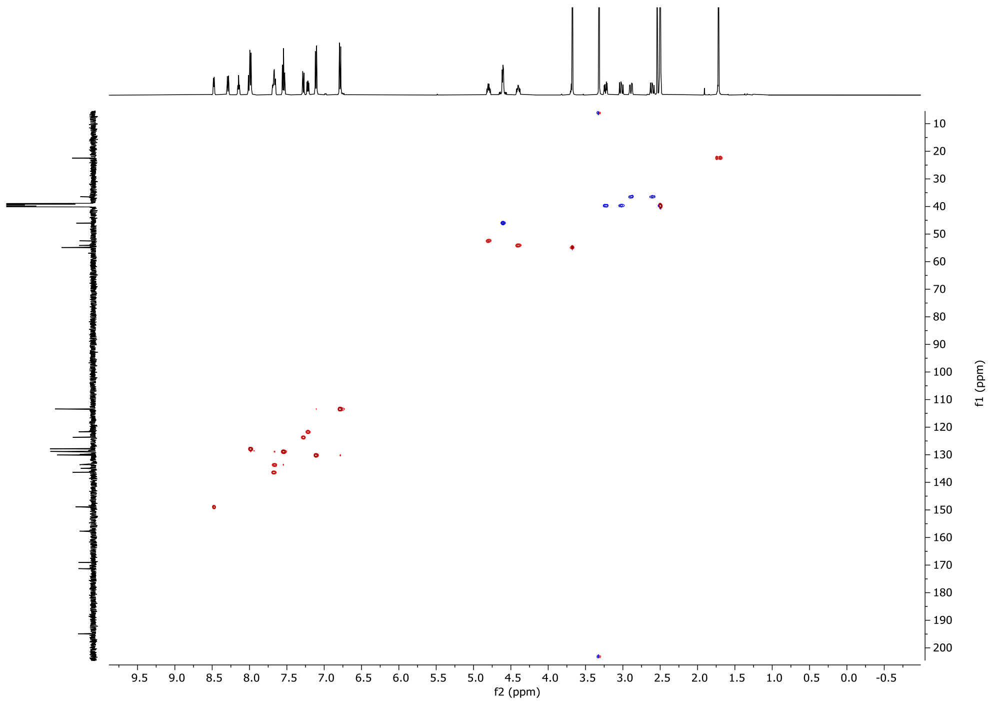

Purity Analysis

1a

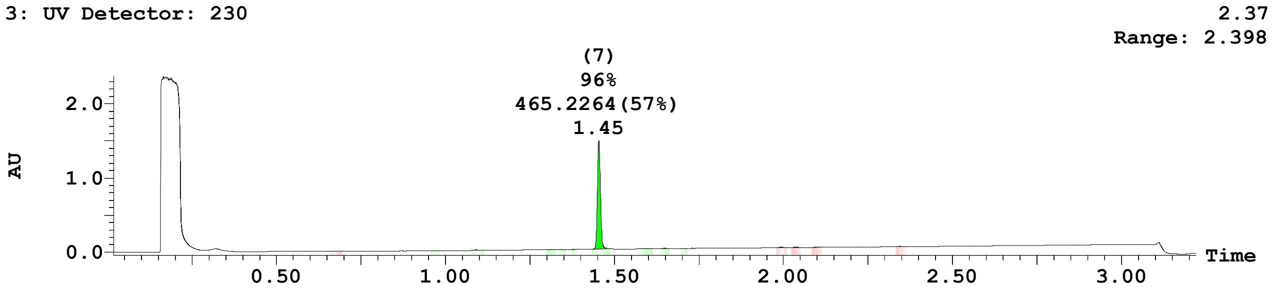

1b

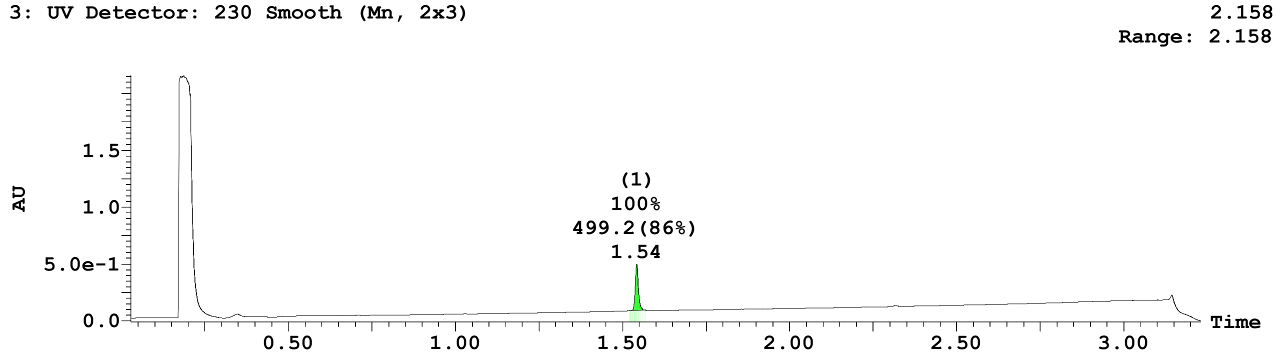

1c

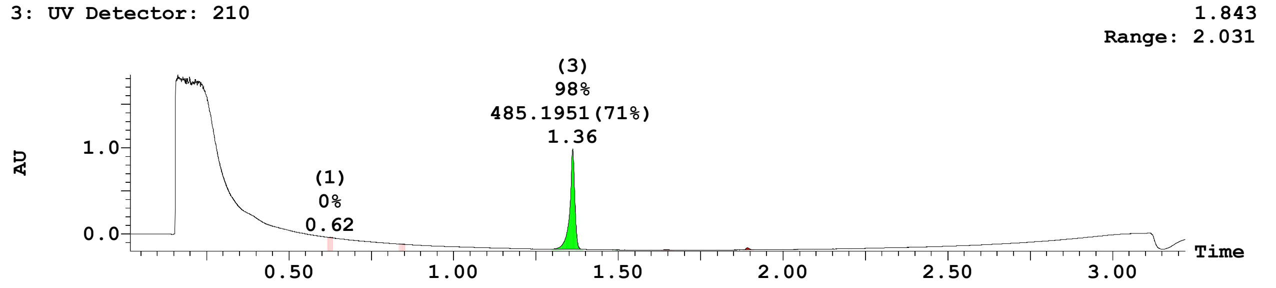

1d

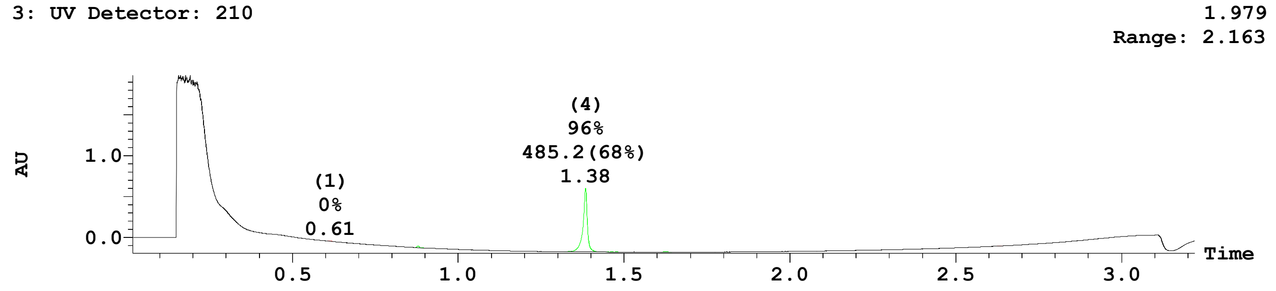

1e

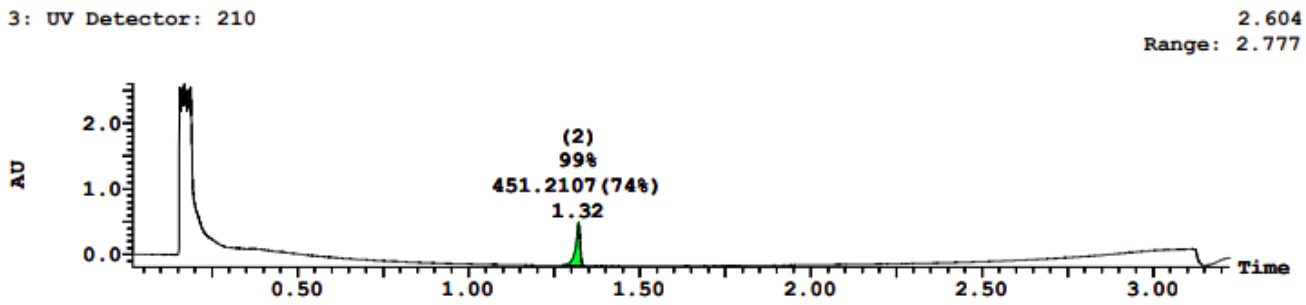
3: UV Detector: 210
2.604
Range: 2.777
(2)
99%
451.2107(74%)
1.32
AU
Time

1f

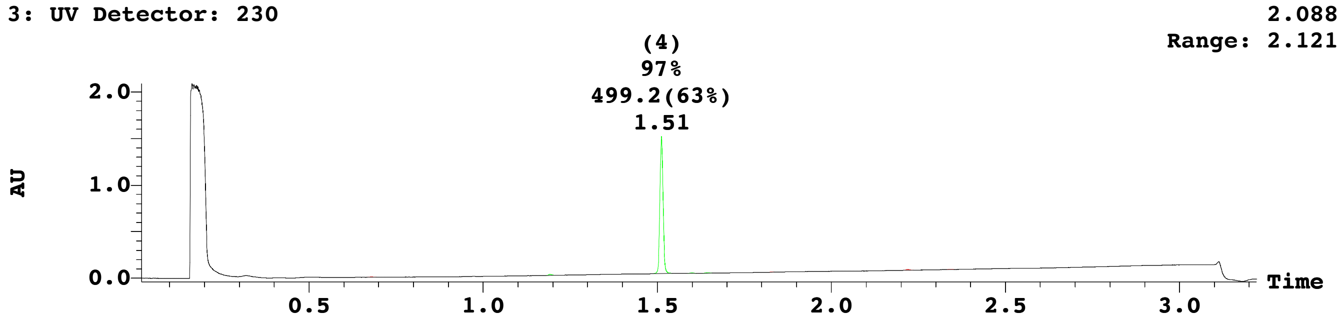
3: UV Detector: 230
2.088
Range: 2.121
(4)
97%
499.2(63%)
1.51
AU
Time

1g

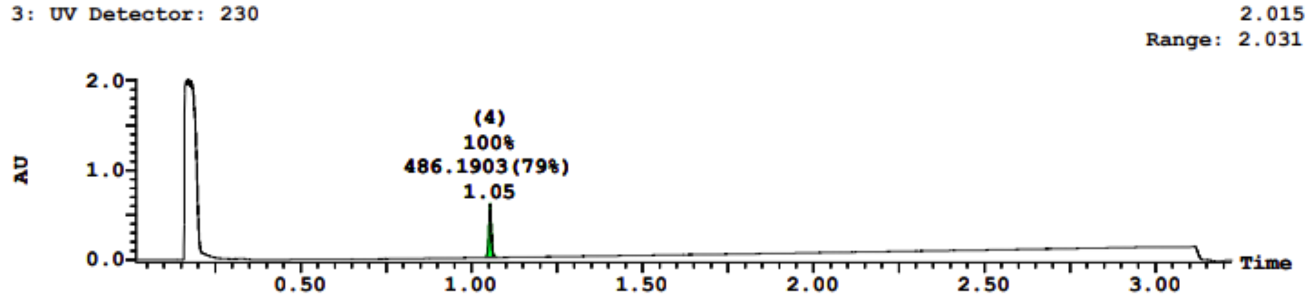
3: UV Detector: 230
2.015
Range: 2.031
(4)
100%
486.1903(79%)
1.05
AU
Time

1h

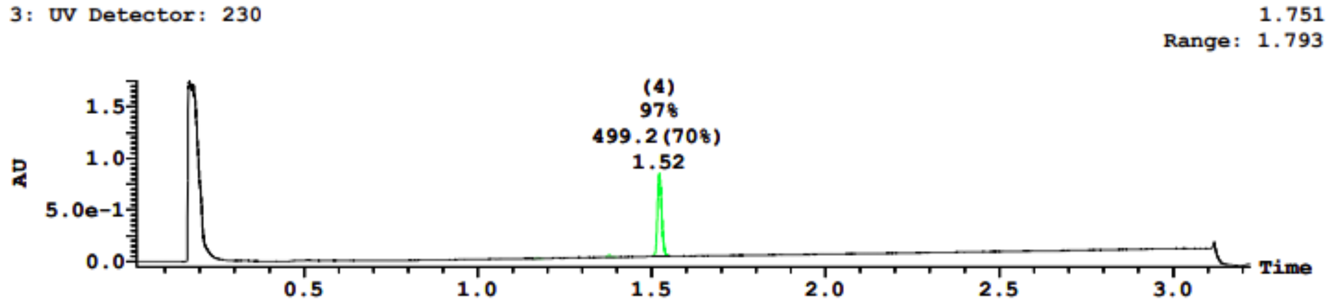
3: UV Detector: 230
1.751
Range: 1.793
(4)
97%
499.2(70%)
1.52
AU
Time

1i

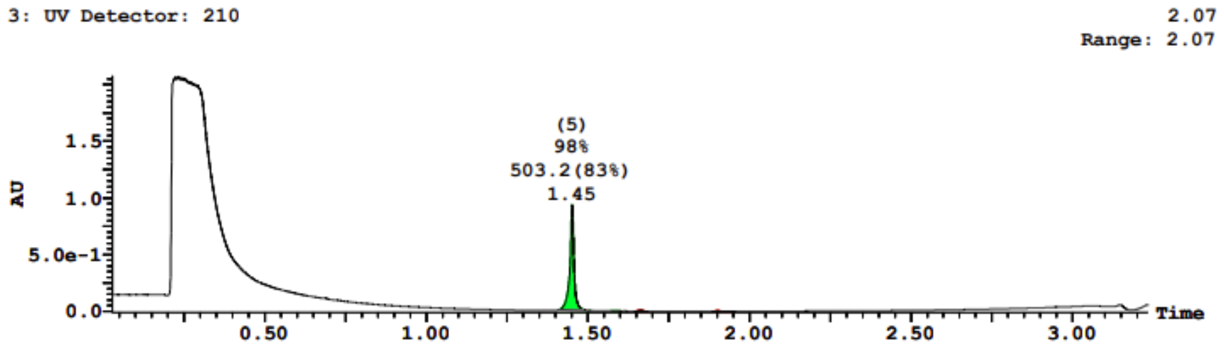
3: UV Detector: 210
2.07
Range: 2.07
(5)
98%
503.2(83%)
1.45
AU
1.5
1.0
5.0e-1
0.0
0.50
1.00
1.50
2.00
2.50
3.00
Time

1j

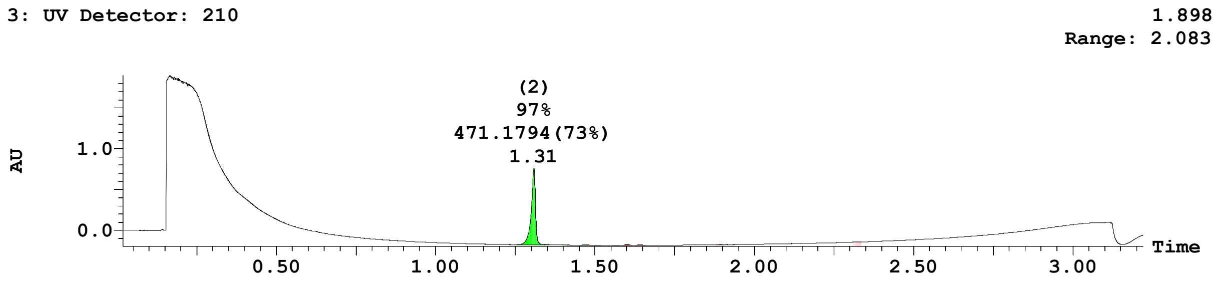
3: UV Detector: 210
1.898
Range: 2.083
(2)
97%
471.1794(73%)
1.31
AU
1.0
0.0
0.50
1.00
1.50
2.00
2.50
3.00
Time

1k

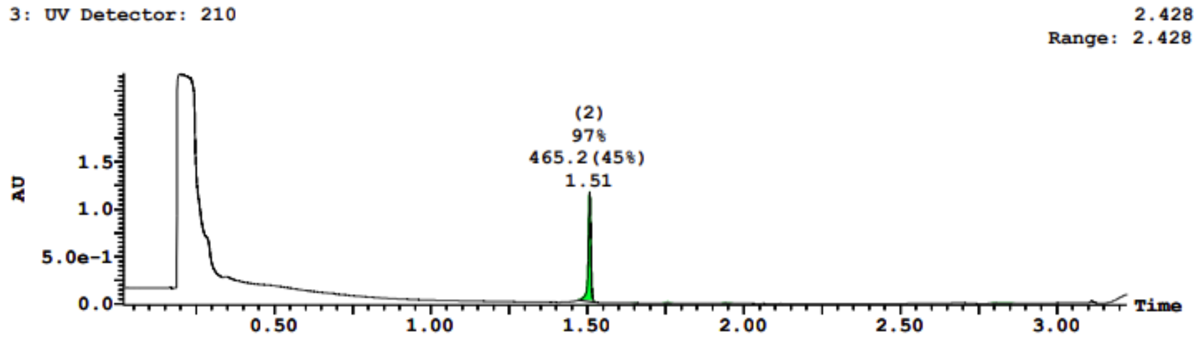
3: UV Detector: 210
2.428
Range: 2.428
(2)
97%
465.2(45%)
1.51
AU
1.5
1.0
5.0e-1
0.0
0.50
1.00
1.50
2.00
2.50
3.00
Time

2c

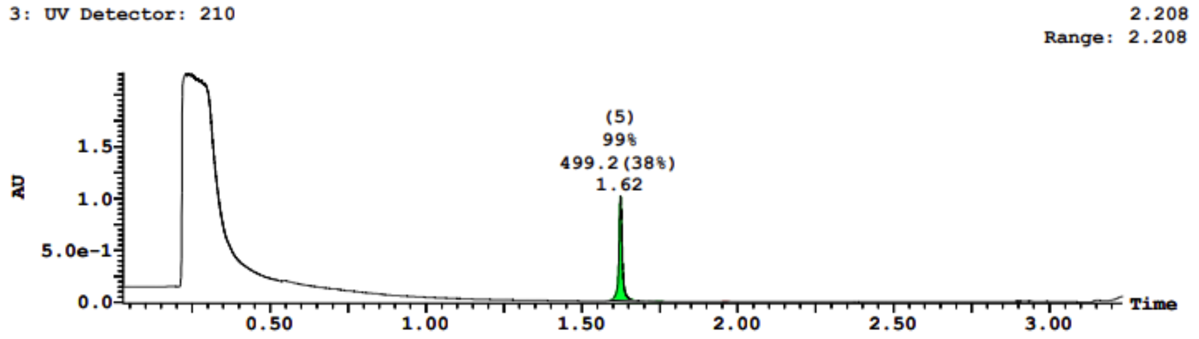
3: UV Detector: 210
2.208
Range: 2.208
(5)
99%
499.2(38%)
1.62
AU
1.5
1.0
5.0e-1
0.0
0.50
1.00
1.50
2.00
2.50
3.00
Time

3c

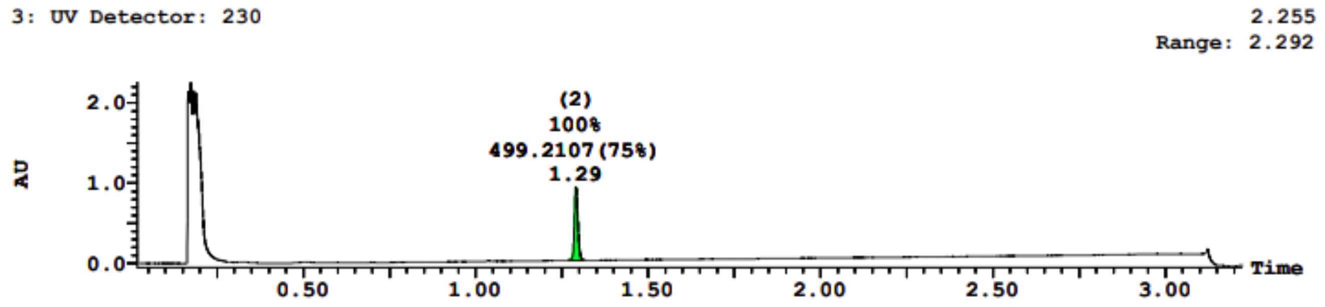
3: UV Detector: 230
2.255
Range: 2.292
AU
2.0
1.0
0.0
(2)
100%
499.2107 (75%)
1.29
0.50
1.00
1.50
2.00
2.50
3.00
Time

3e

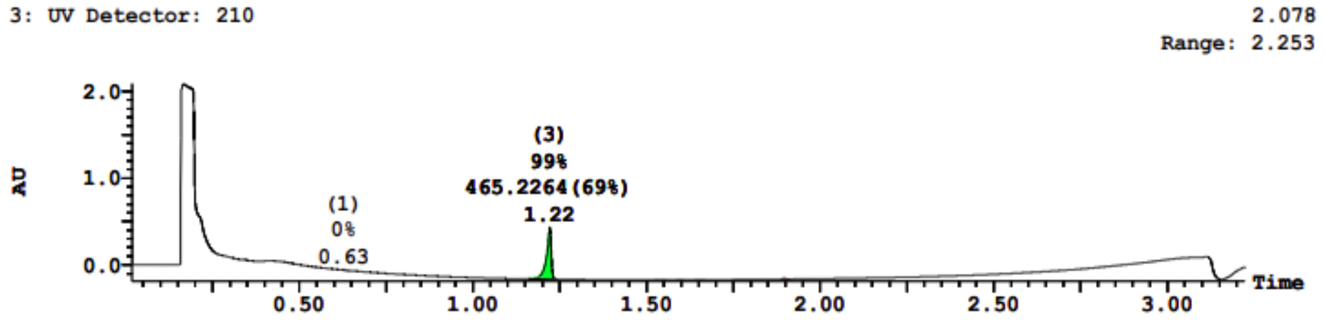
3: UV Detector: 210
2.078
Range: 2.253
AU
2.0
1.0
0.0
(1)
0%
0.63
(3)
99%
465.2264 (69%)
1.22
0.50
1.00
1.50
2.00
2.50
3.00
Time

4a

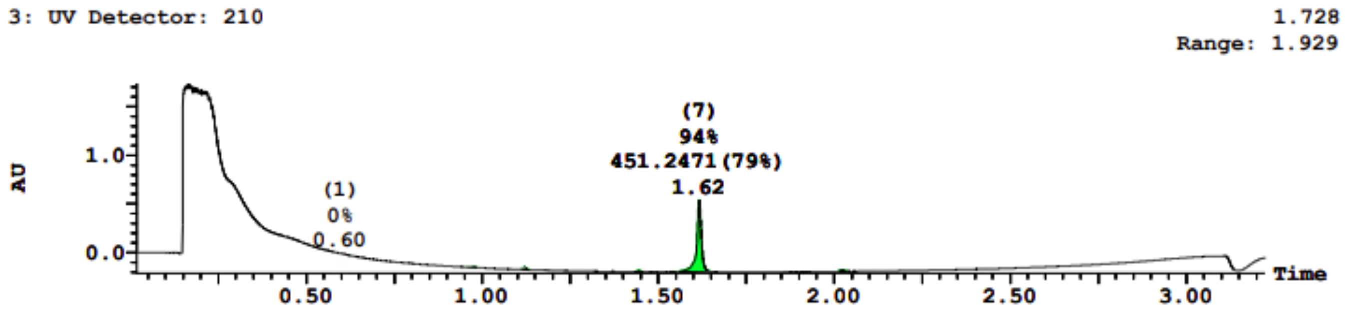
3: UV Detector: 210
1.728
Range: 1.929
AU
1.0
0.0
(1)
0%
0.60
(7)
94%
451.2471 (79%)
1.62
0.50
1.00
1.50
2.00
2.50
3.00
Time

4b

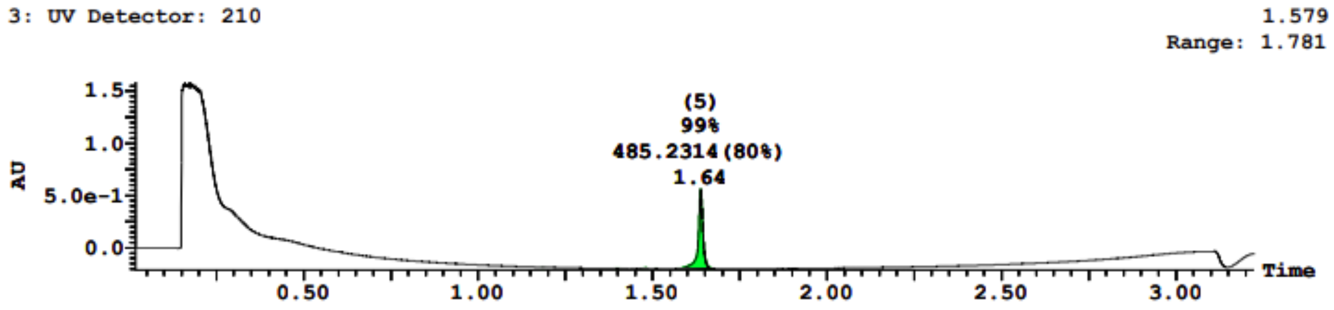
3: UV Detector: 210
1.579
Range: 1.781
AU
1.5
1.0
5.0e-1
0.0
(5)
99%
485.2314 (80%)
1.64
0.50
1.00
1.50
2.00
2.50
3.00
Time

4c

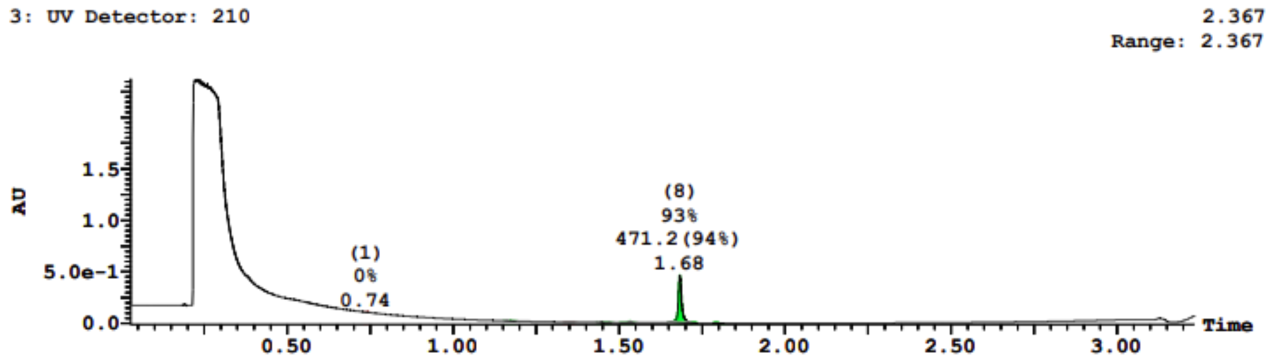
3: UV Detector: 210
2.367
Range: 2.367
AU
(8)
93%
471.2 (94%)
1.68
(1)
0%
0.74
Time

4e

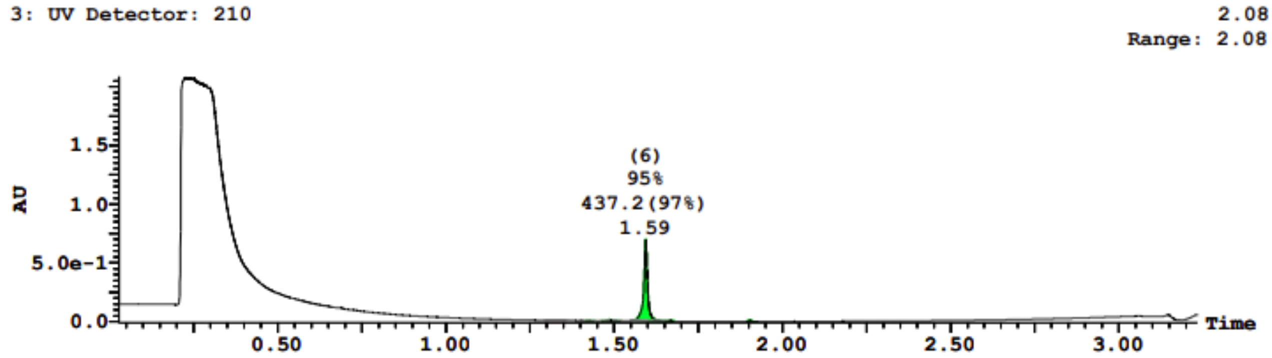
3: UV Detector: 210
2.08
Range: 2.08
AU
(6)
95%
437.2 (97%)
1.59
Time

4j

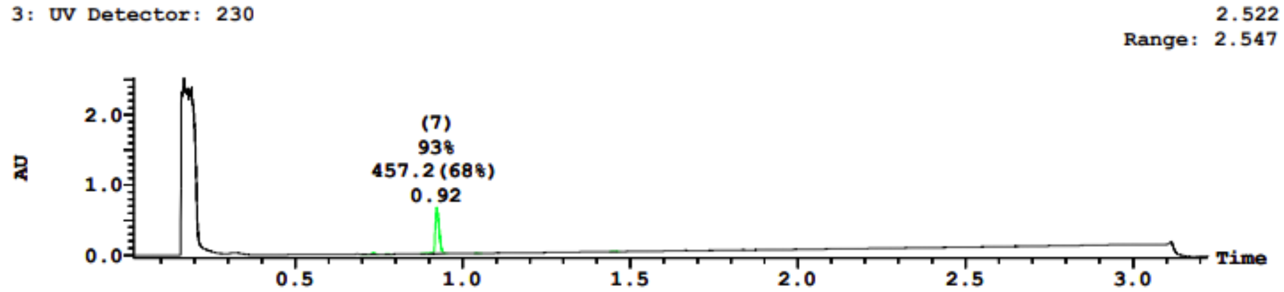
3: UV Detector: 230
2.522
Range: 2.547
AU
(7)
93%
457.2 (68%)
0.92
Time

5a

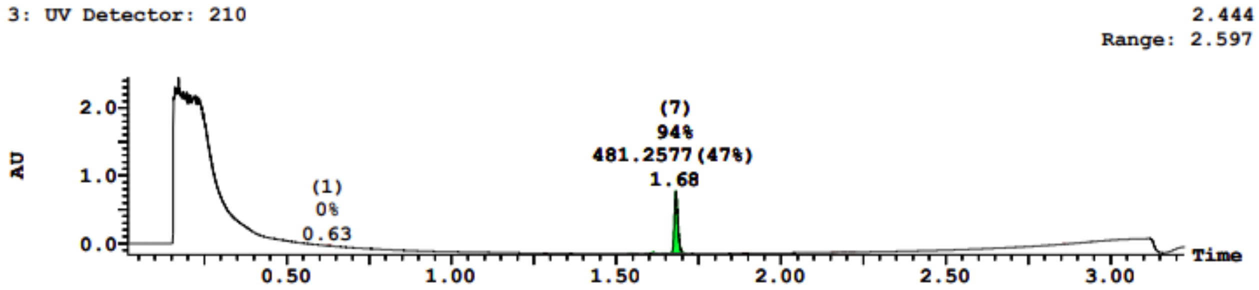
3: UV Detector: 210
2.444
Range: 2.597
AU
(7)
94%
481.2577 (47%)
1.68
(1)
0%
0.63
Time

5b

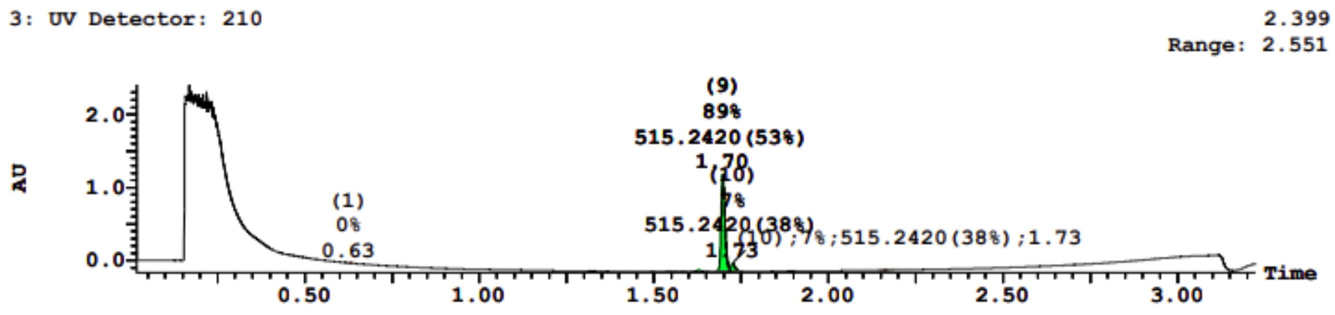
3: UV Detector: 210
2.399
Range: 2.551
(9)
89%
515.2420(53%)
1.70
(10)
7%
515.2420(38%)
1.73
(10);7%;515.2420(38%);1.73
(1)
0%
0.63
AU
Time

5c

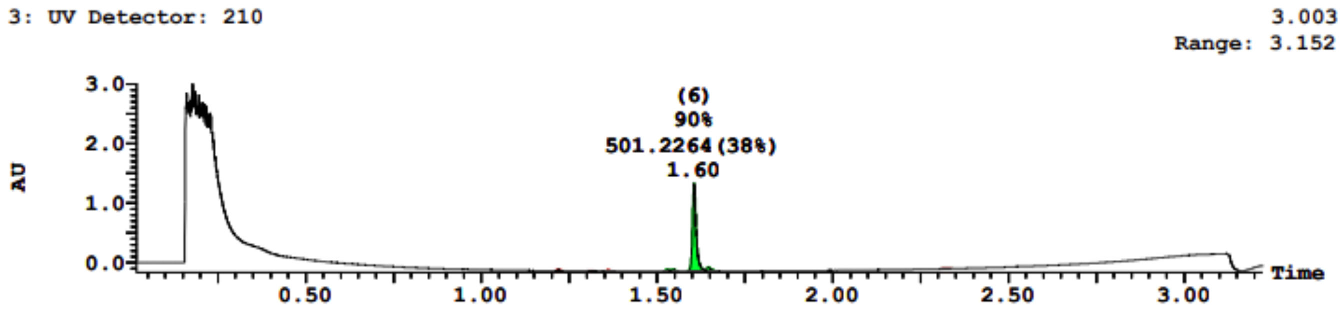
3: UV Detector: 210
3.003
Range: 3.152
(6)
90%
501.2264(38%)
1.60
AU
Time

5d

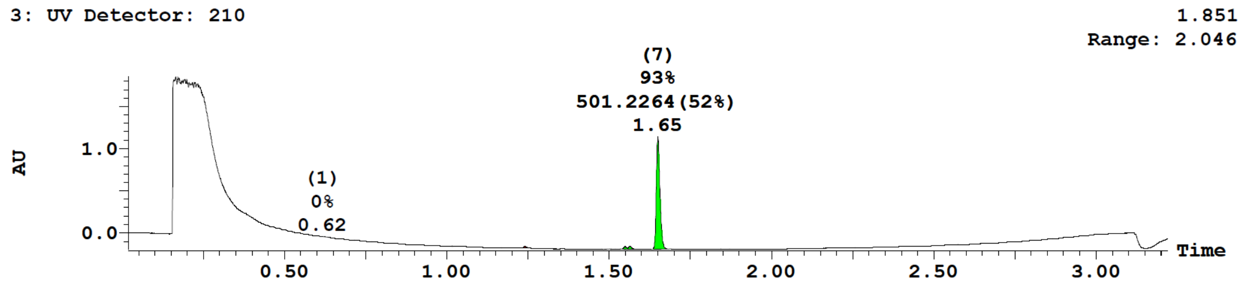
3: UV Detector: 210
1.851
Range: 2.046
(7)
93%
501.2264(52%)
1.65
(1)
0%
0.62
AU
Time

5e

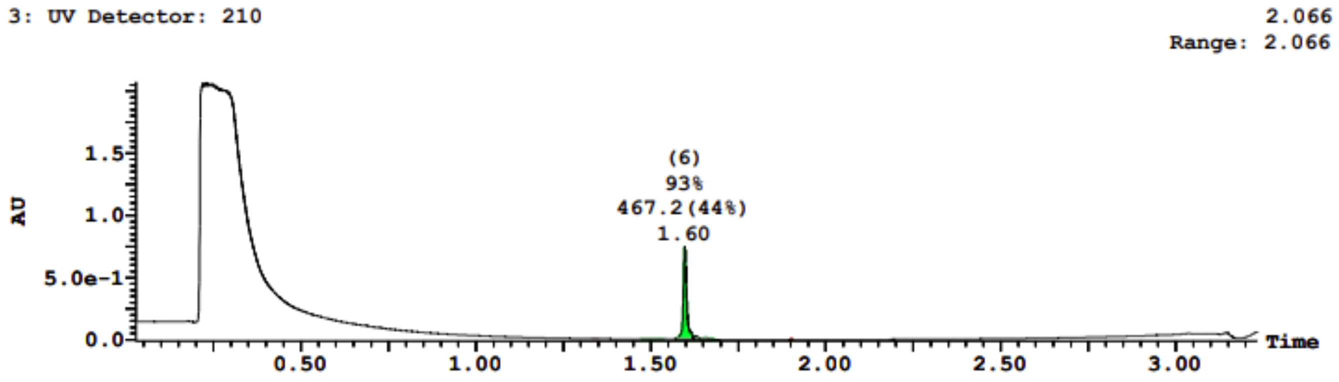
3: UV Detector: 210
2.066
Range: 2.066
(6)
93%
467.2(44%)
1.60
AU
Time

5f

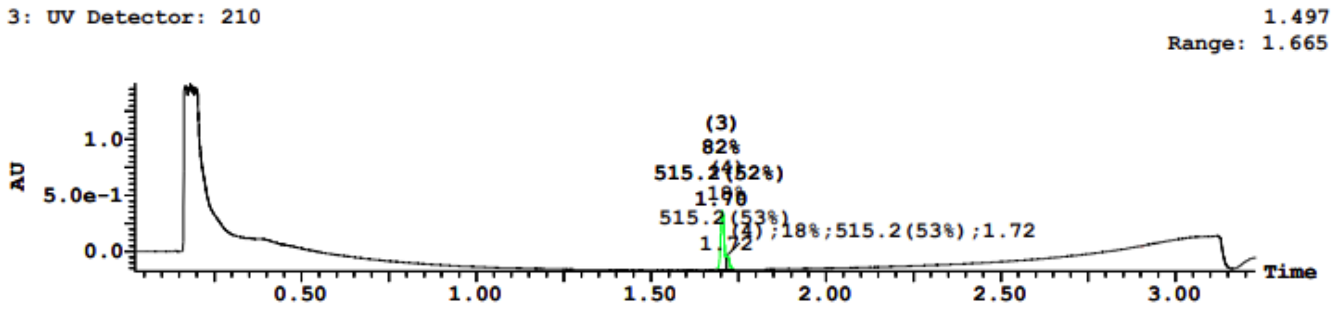
3: UV Detector: 210
1.497
Range: 1.665
AU
1.0
5.0e-1
0.0
(3)
82%
515.2 (52%)
1.70
515.2 (53%)
1.72
(4) ;18%;515.2(53%);1.72
Time
0.50
1.00
1.50
2.00
2.50
3.00

5g

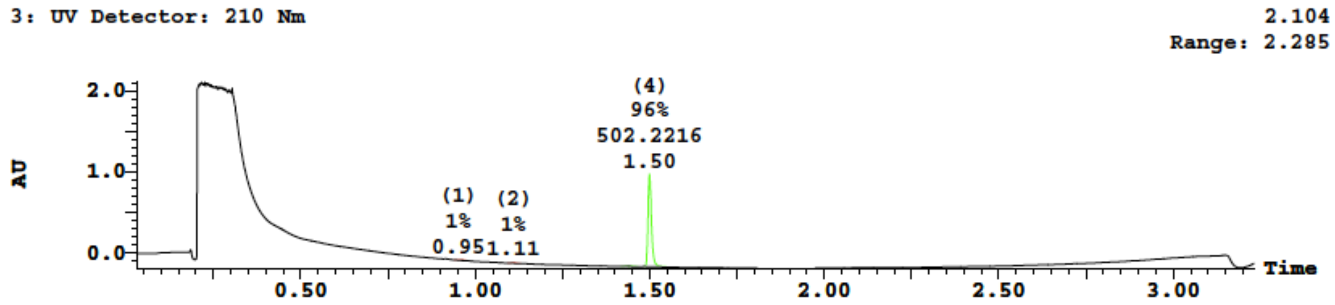
3: UV Detector: 210 Nm
2.104
Range: 2.285
AU
2.0
1.0
0.0
(4)
96%
502.2216
1.50
(1)
1%
0.95
(2)
1%
1.11
Time
0.50
1.00
1.50
2.00
2.50
3.00

Table S1. Calculated descriptors for macrocycles and linear compounds

Compound	MW (Da)	cLogP	HBD	HBA	TPSA (Å^2)	NRotB	Kier's Flexibility Index
**1a**	465.55	2.76	3.0	5.0	113.6	4.0	8.4
**1b**	499.57	2.96	3.0	5.0	113.6	4.0	8.31
**1c**	485.54	2.57	3.0	5.0	113.6	3.0	7.75
**1d**	485.54	3.0	3.0	5.0	113.6	2.0	7.44
**1e**	451.52	2.37	3.0	5.0	113.6	3.0	7.82
**1f**	499.57	2.96	3.0	5.0	113.6	4.0	8.31
**1g**	486.53	1.96	3.0	6.0	126.49	3.0	7.69
**1h**	499.57	2.87	3.0	5.0	113.6	3.0	7.98
**1i**	503.53	2.71	3.0	5.0	113.6	3.0	7.92
**1j**	471.51	2.7	3.0	5.0	113.6	2.0	7.21
**1k**	465.55	2.76	3.0	5.0	113.6	4.0	8.4
**2c**	499.57	2.96	3.0	5.0	113.6	3.0	8.31
**3c**	499.57	2.96	3.0	5.0	113.6	3.0	8.31
**3e**	465.55	2.76	3.0	5.0	113.6	3.0	8.4
**4a**	451.57	3.19	2.0	5.0	87.74	4.0	8.22
**4b**	485.58	3.38	2.0	5.0	87.74	4.0	8.13
**4c**	471.56	2.99	2.0	5.0	87.74	3.0	7.57
**4e**	437.54	2.8	2.0	5.0	87.74	3.0	7.64
**4j**	457.53	3.12	2.0	5.0	87.74	2.0	7.04
**5a**	481.59	2.66	3.0	5.0	113.6	13.0	10.94
**5b**	515.61	2.86	3.0	5.0	113.6	13.0	10.6
**5c**	501.58	2.47	3.0	5.0	113.6	12.0	9.96
**5d**	501.58	2.91	3.0	5.0	113.6	11.0	9.55
**5e**	467.57	2.27	3.0	5.0	113.6	12.0	10.26
**5f**	515.61	2.86	3.0	5.0	113.6	13.0	10.6
**5g**	502.57	1.86	3.0	6.0	126.49	12.0	9.9

Table S2. Crystallographic data for compounds 1g, 1j, 2c, 21c and 5c

Parameter	Compound 1g	Compound 1j	Compound 2c	Compound 21c	Compound 5c
Formula	$C_{27}H_{26}N_4O_5$	$C_{27}H_{25}N_3O_5$	$C_{29}H_{29}N_3O_5 .0.5(C_6H_5Cl)$	$C_{32}H_{35}N_3O_6$	$C_{29}H_{31}N_3O_5$
*D*calc	1.288	1.276	1.274	1.257	1.282
μ (mm^{-1})	0.742	0.730	1.112	0.711	0.718
Formula Weight	486.52	471.50	555.83	557.63	501.57
Colour	colourless	colourless	colourless	clear colourless	clear colourless
Shape	block	lath	lath	plate-shaped	prism-shaped
Size (mm^3)	0.132×0.114×0.040	0.120×0.030×0.010	0.196×0.063×0.020	0.21×0.04×0.02	0.75×0.06×0.04
T (K)	100(2)	100(2)	100(2)	100(2)	100(2)
Crystal System	monoclinic	orthorhombic	triclinic	monoclinic	monoclinic
Space Group	$P2_1$	$P2_12_12_1$	$P1$	$P2_1$	0.01(2)
a (Å)	14.33979(8)	9.39860(10)	13.52786(11)	12.7214(6)	0.006(17)
b (Å)	9.55470(6)	10.3162(2)	14.85505(18)	9.5076(4)	$P2_1$
c (Å)	18.51821(10)	25.3226(4)	16.6607(2)	13.4855(8)	11.21605(4)
α (˚)	90	90	112.3935(11)	90	29.81377(8)
β (˚)	98.6132(5)	90	104.8159(8)	115.368(6)	11.92336(4)
Υ (˚)	90	90	97.1547(8)	90	90
V (Å^3)	2508.61(2)	2455.22(7)	2898.94(6)	1473.79(14)	102.1073(3)
Z; Z'	4; 2	4;1	4; 4	2;1	6; 3
θ_{min} (˚)	2.413	3.491	3.039	3.627	2.964
θ_{max} (˚)	68.234	68.217	68.245	67.997	77.605
Measured Reflections	79125	43766	92989	25766	411709
Independent Reflections	9161	4491	20095	5290	16304
Observed Reflections	8971	4193	18396	4567	15568
R_{int}	0.0823	0.0687	0.0507	0.0913	0.0587
Parameters	669	326	1508	373	1119
Largest Peak	0.259	0.159	0.252	0.485	157
Deepest Hole	-0.236	-0.115	-0.371	-0.433	0.193
GooF	1.055	1.065	1.051	1.062	-0.226
wR_2 (all data)	0.0844	0.0731	0.1309	0.2491	0.1074
wR_2	0.0839	0.0714	0.1270	0.2364	0.1064
R_1 (all data)	0.0339	0.0372	0.0534	0.0971	0.0395
R_1	0.0333	0.0331	0.0492	0.0860	0.0381
Hooft Parameter	-0.05(4)	-0.05(9)	-0.005(6)	-0.05(10)	0.006(17)

Table S3. Calculated data for the conformations in the in silico ensembles of macrocycle 1c and linear matched pair 5c

	R_{gyr} (Å)	SA 3D PSA ($Å^2$)	SA 3D NPSA ($Å^2$)	ΔG (kcal/mol)	Population (%)
**1c_1**	3,87	126,0	515,9	2,14	9,8
**1c_2**	3,88	138,4	507,8	3,75	5,1
**1c_3**	3,85	128,4	530,4	2,80	7,5
**1c_4**	4,29	150,6	544,6	1,09	15,0
**1c_5**	4,19	144,9	535,6	0,00	23,3
**1c_6**	3,85	129,0	530,4	2,79	7,6
**1c_7**	3,87	141,8	513,9	5,34	2,7
**1c_8**	4,24	149,1	555,5	1,20	14,4
**1c_9**	4,13	138,0	520,5	1,17	14,6
**5c_1**	4,65	124,6	640,0	5,64	4,7
**5c_2**	4,17	110,5	632,9	9,34	1,0
**5c_3**	4,65	124,6	640,0	8,00	1,8
**5c _4**	5,18	131,9	673,3	5,93	4,1
**5c _5**	4,63	129,9	631,4	5,81	4,4
**5c_6**	5,06	135,5	676,0	3,44	11,3
**5c_7**	4,48	125,7	621,8	0,00	45,4
**5c_8**	5,29	125,4	701,6	1,79	22,1
**5c_9**	4,17	97,1	614,5	6,14	3,8
**5c_10**	4,17	118,3	601,3	8,65	1,4

Crystal structures

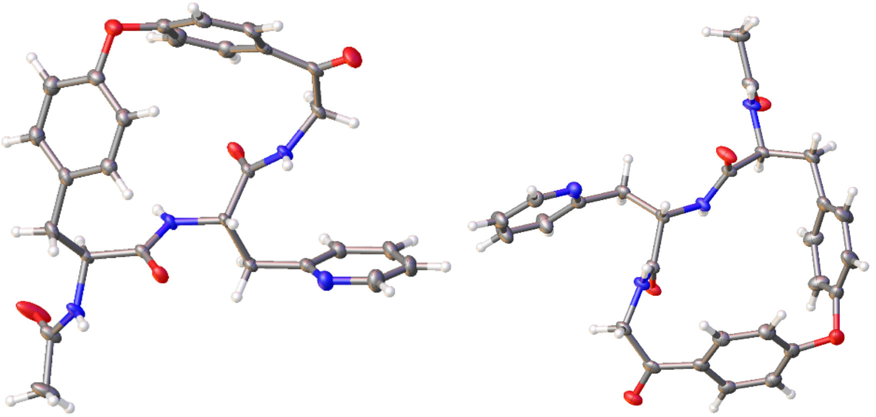

Figure S1. Thermal ellipsoid drawing of independent molecules of compound **1g** drawn at 50% probability level. Each independent molecule is drawn separately.

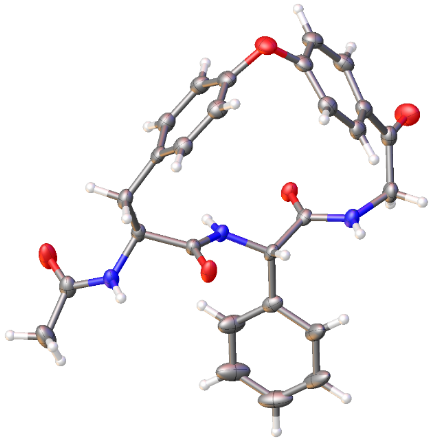

Figure S2. Thermal ellipsoid drawing of independent molecules of compound **1j** drawn at 50% probability level.

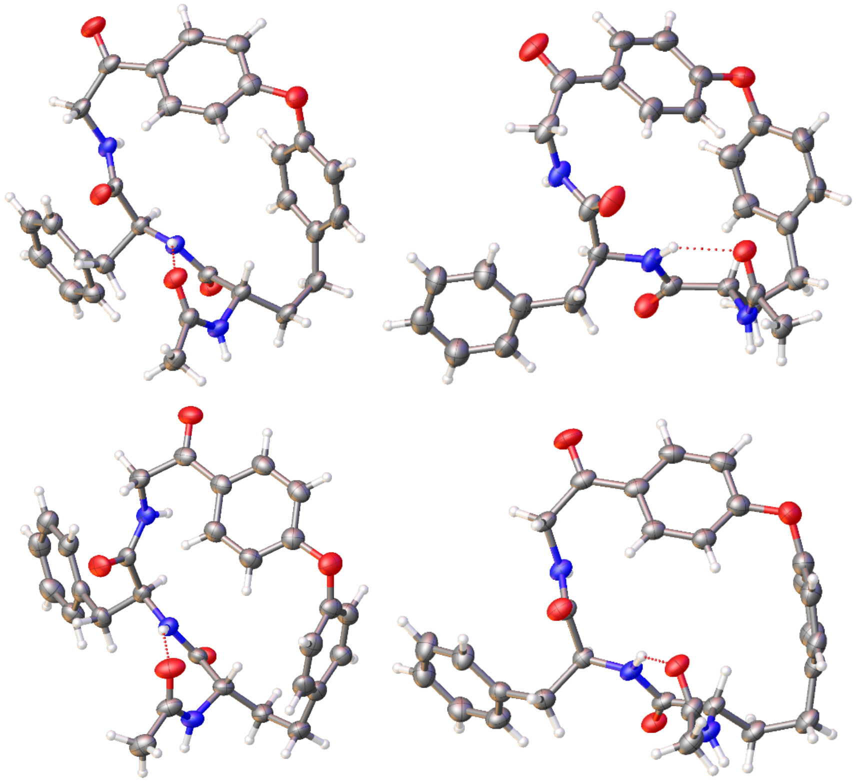

Figure S3. Thermal ellipsoid drawing of independent molecules of compound **2c** drawn at 50% probability level. Each independent molecule is drawn separately. Solvent molecules are excluded for clarity.

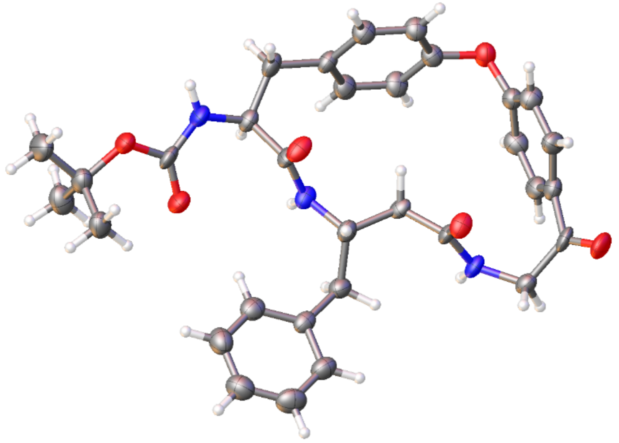

Figure S4. Thermal ellipsoid drawing of independent molecules of compound **21c** drawn at 50% probability level.

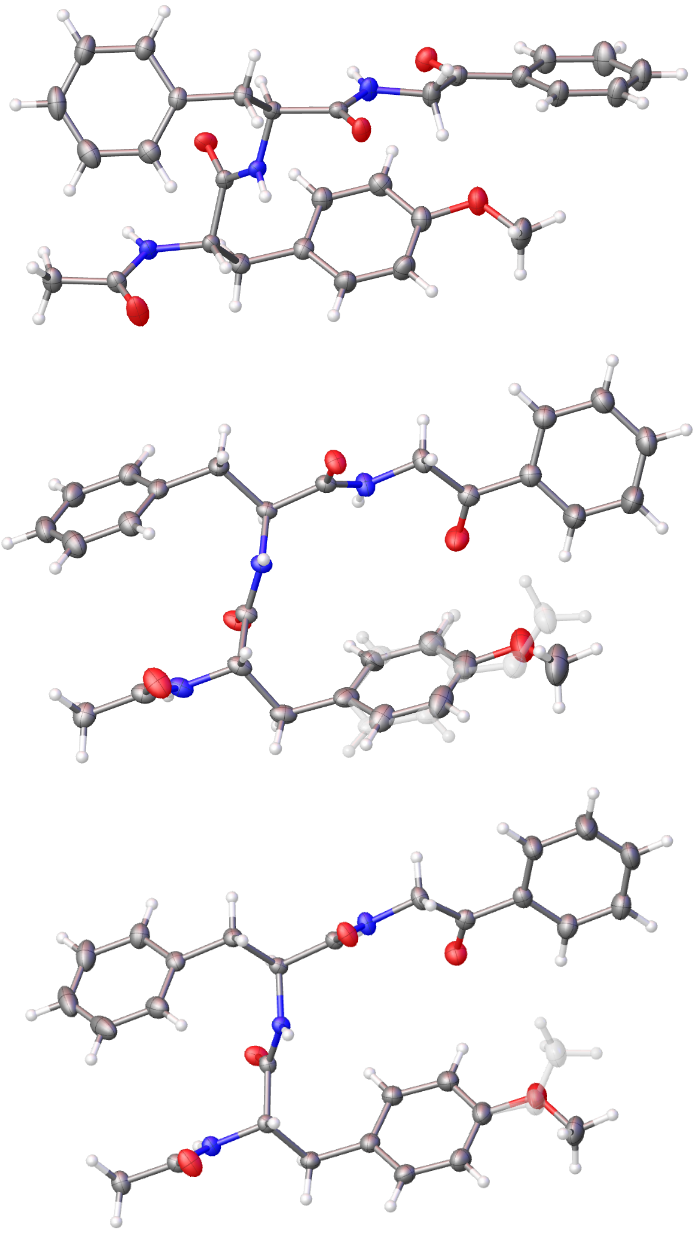

Figure S5. Thermal ellipsoid drawing of independent molecules of compound **5c** drawn at 50% probability level. Each independent molecule is drawn separately. The minor part of the disorder is depicted as a semi-transparent layer over the major part of the disorder which is shown as opaque.

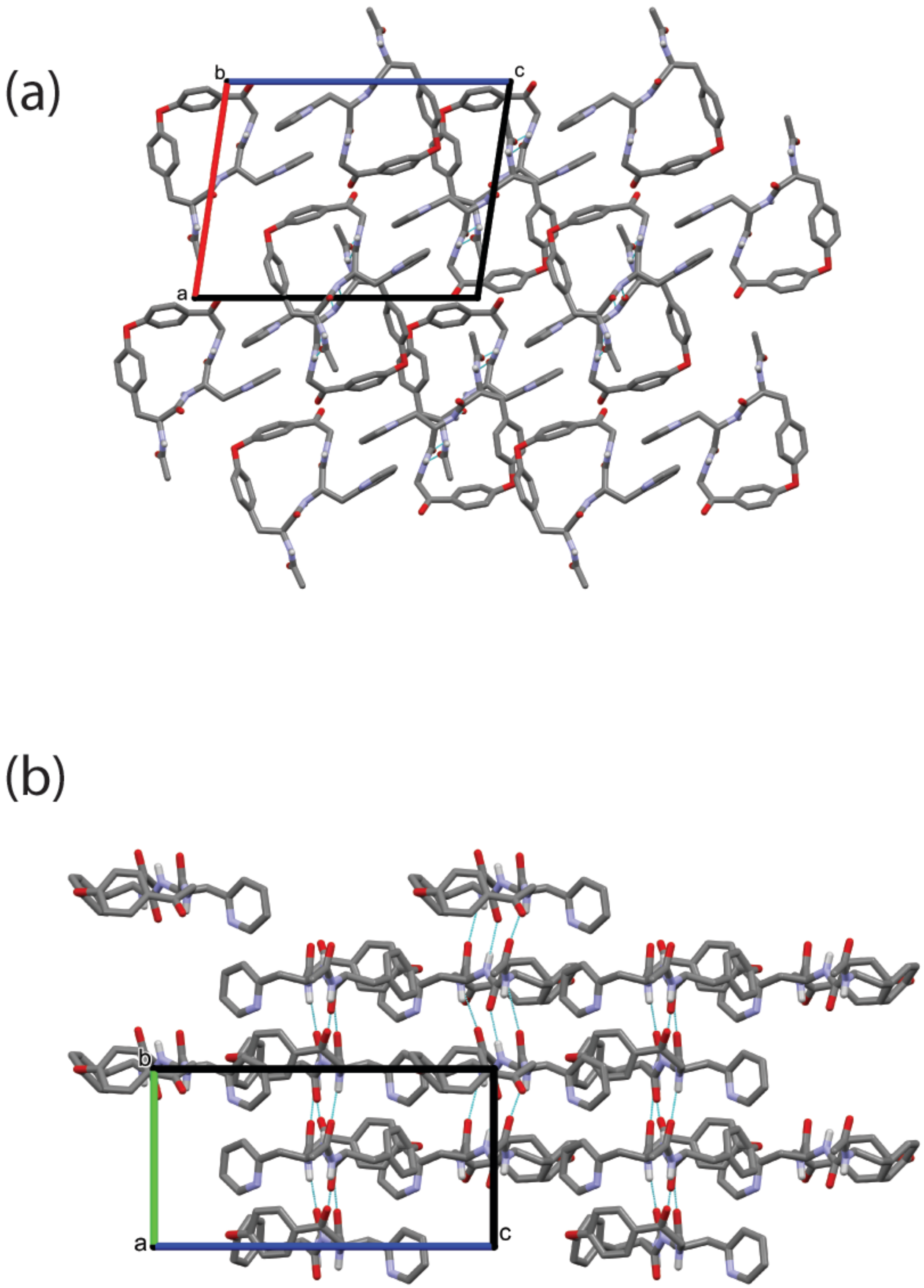

Figure S6. Crystal packing view of **1g** along (**a**) *b*-axis and (**b**) *a*-axis. Hydrogen bonds are indicated by blue dashed lines in panel (b). Only hydrogen atoms participating in hydrogen bond are shown.

(a)

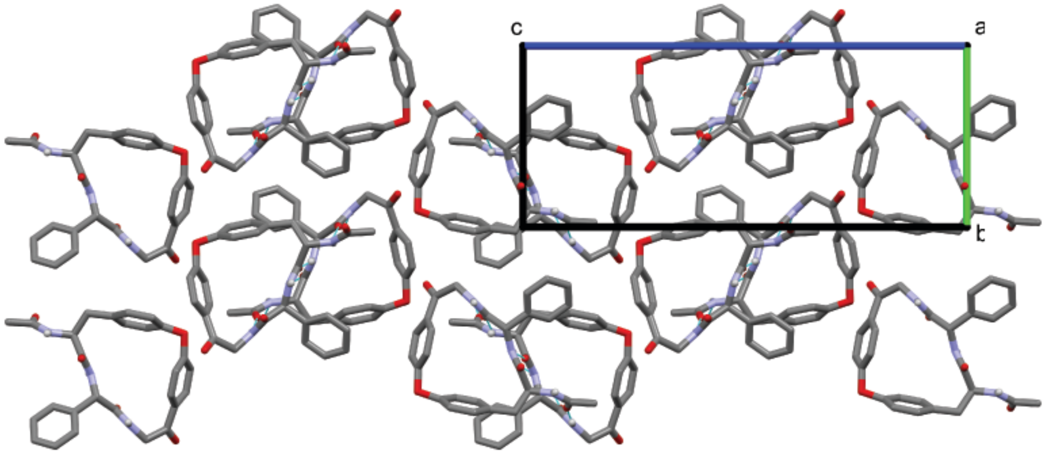

(b)

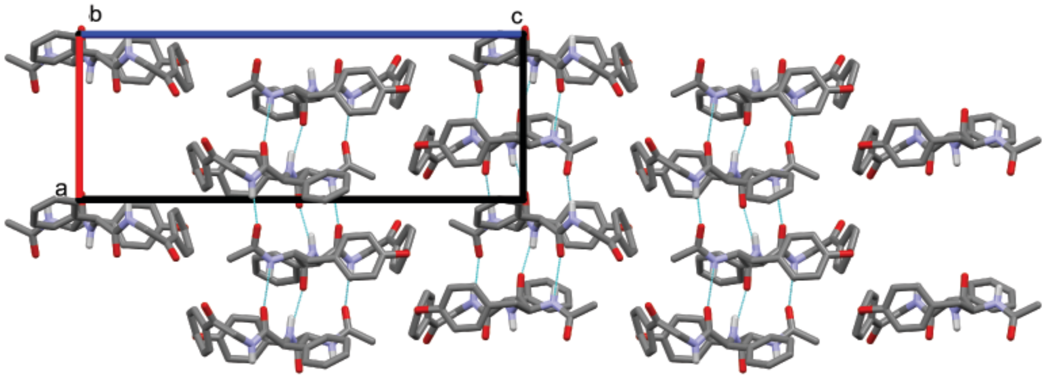

Figure S7. Crystal packing view of **1j** along (**a**) *a*-axis and (**b**) *b*-axis. Hydrogen bonds are indicated by blue dashed lines in panel (b). Only hydrogen atoms participating in hydrogen bond are shown.

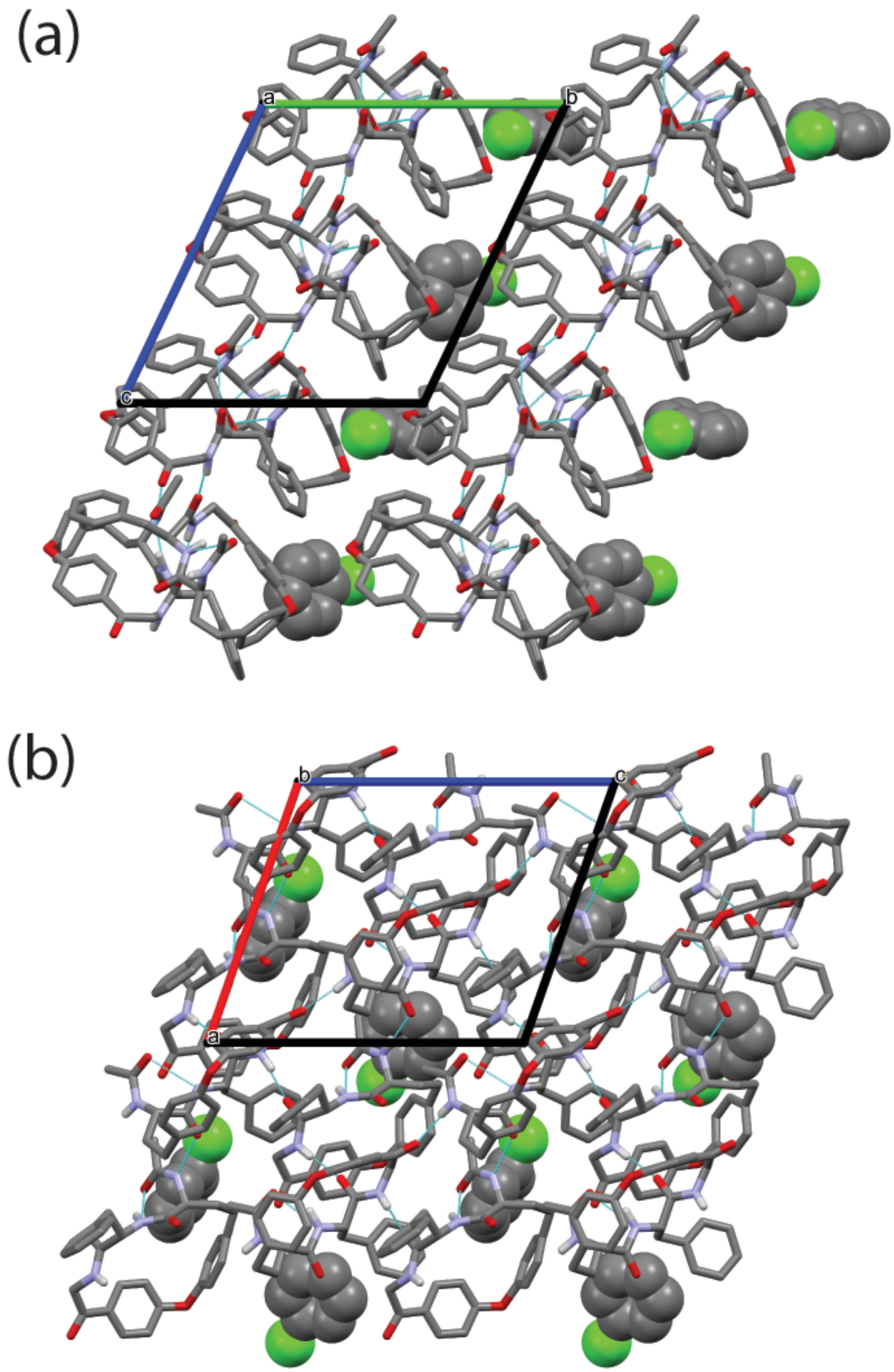

Figure S8. Crystal packing view of **2c** along (**a**) *a*-axis and (**b**) *b*-axis. Hydrogen bonds are indicated by blue dashed lines. Only hydrogen atoms participating in hydrogen bond are shown. Chlorobenzene molecules are drawn in space filling mode.

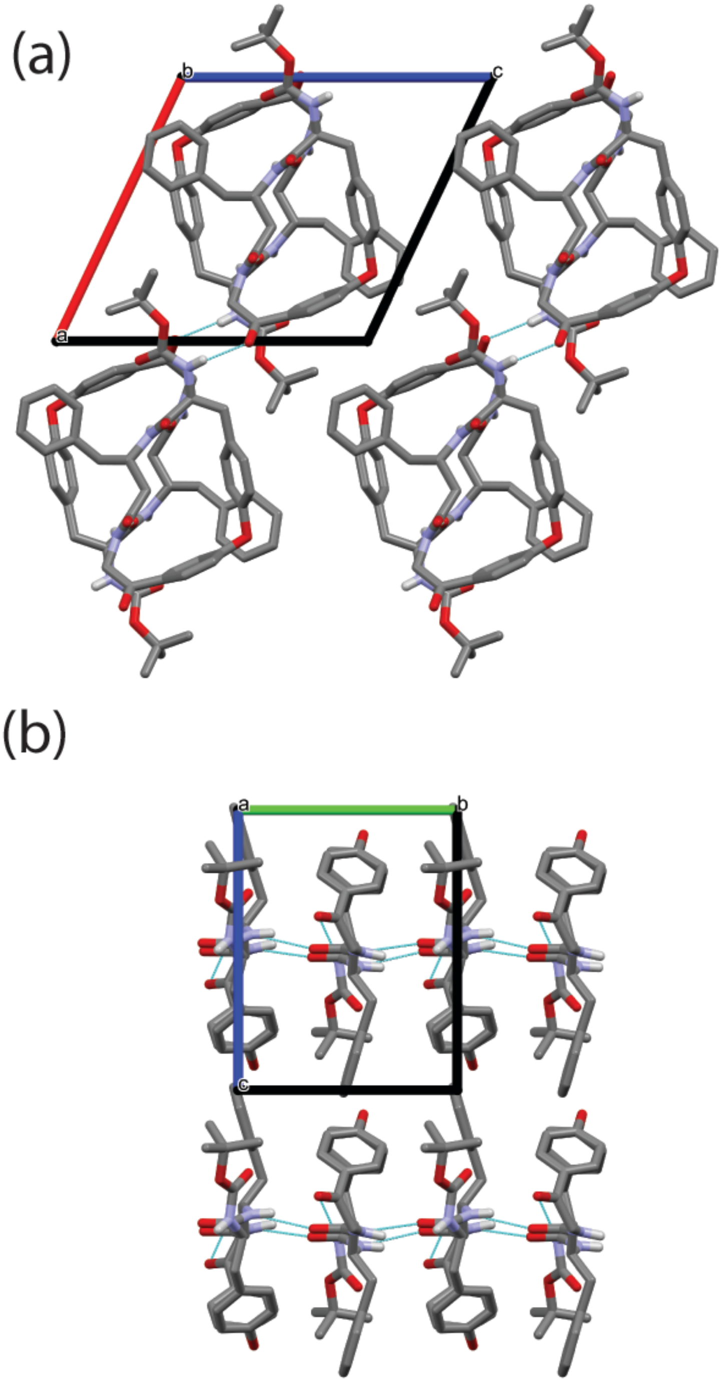

Figure S9. Crystal packing view of **21c** along (**a**) *b*-axis and (**b**) *a*-axis. Hydrogen bonds are indicated by blue dashed lines. Only hydrogen atoms participating in hydrogen bond are shown.

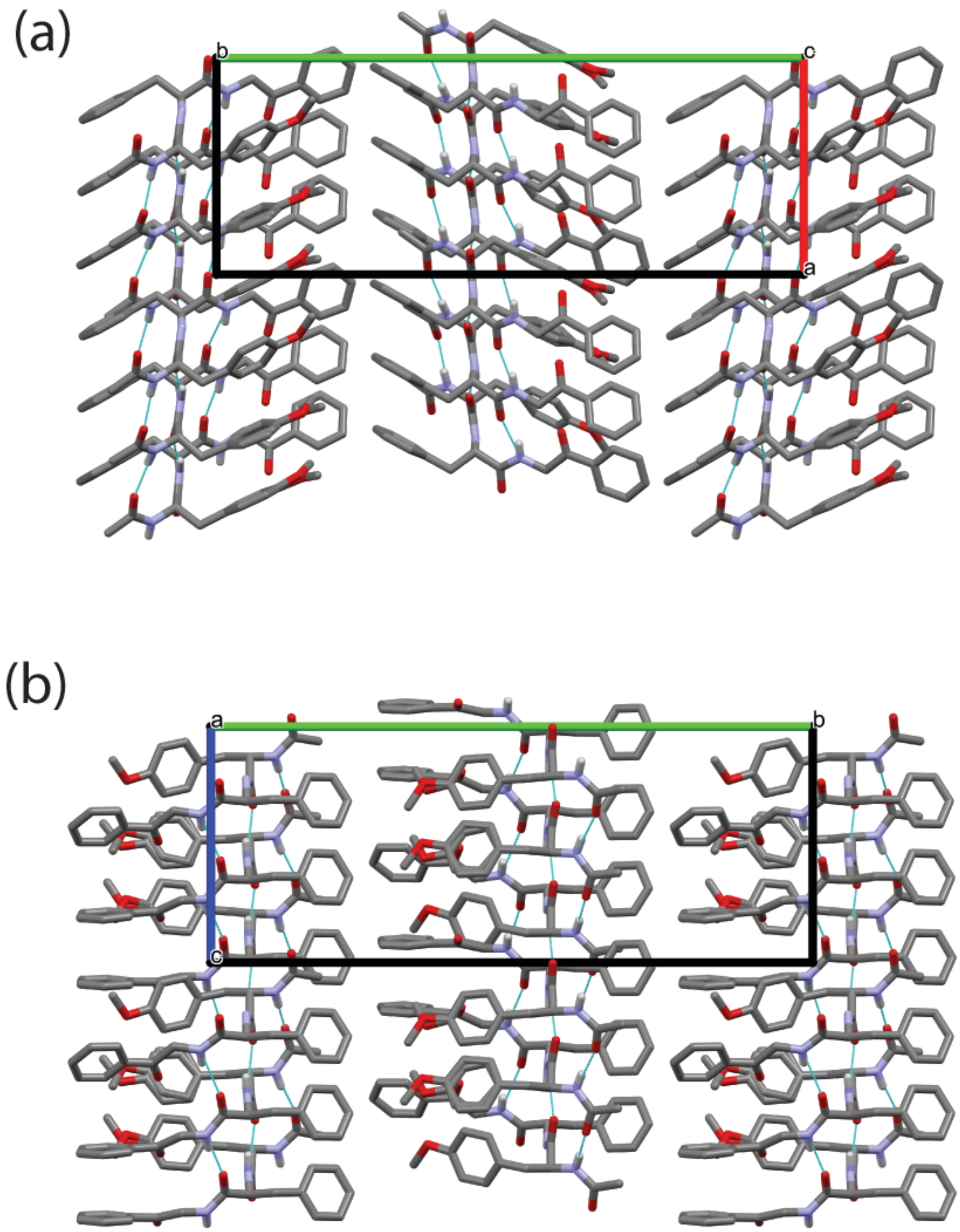

Figure S10. Crystal packing view of **5c** along (**a**) *c*-axis and (**b**) *a*-axis. Hydrogen bonds are indicated by blue dashed lines. Only hydrogen atoms participating in hydrogen bond are shown.

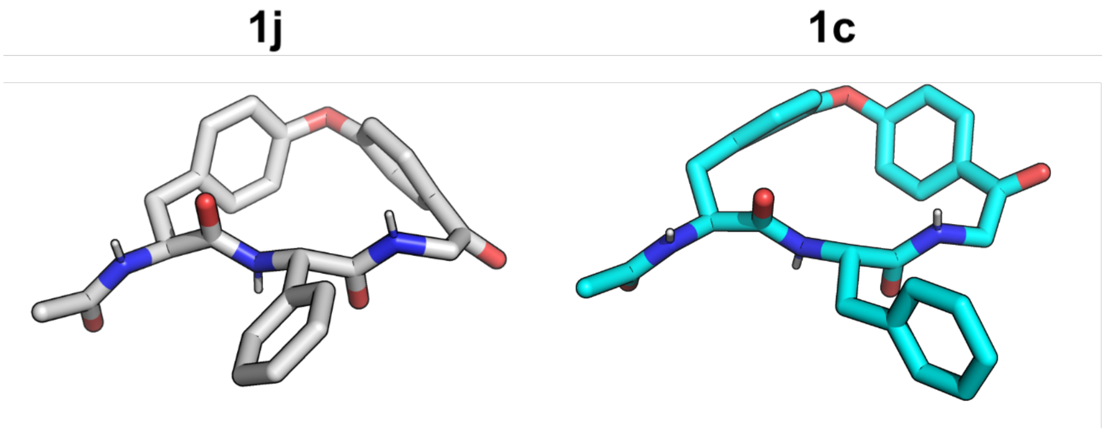

Figure S11. Comparison of conformations adopted by **1j** and **1c** as determined by X-ray crystallography.

Conformational analysis of 1c and 5c

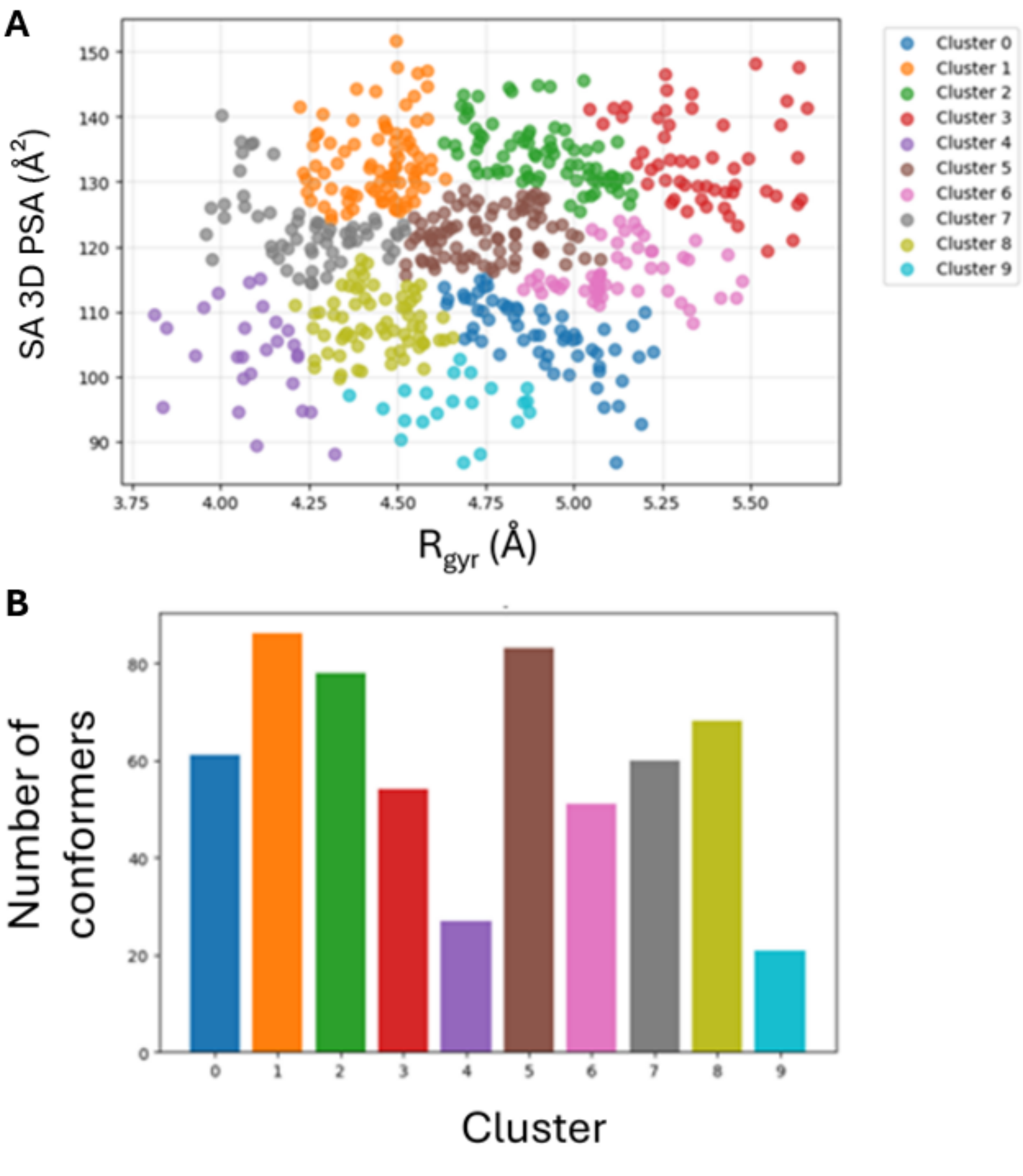

Figure S12. (**A**) Solvent accessible 3D polar surface area (SA 3D PSA) versus radius of gyration (R_{gyr}) for the conformations obtained by MCMM conformational sampling for linear analogue **5c**. The color coding indicates the 10 clusters into which the conformational ensemble was divided by property-based hierarchical clustering. (**B**) Number of conformations by cluster in the *in silico* ensemble of **5c**.

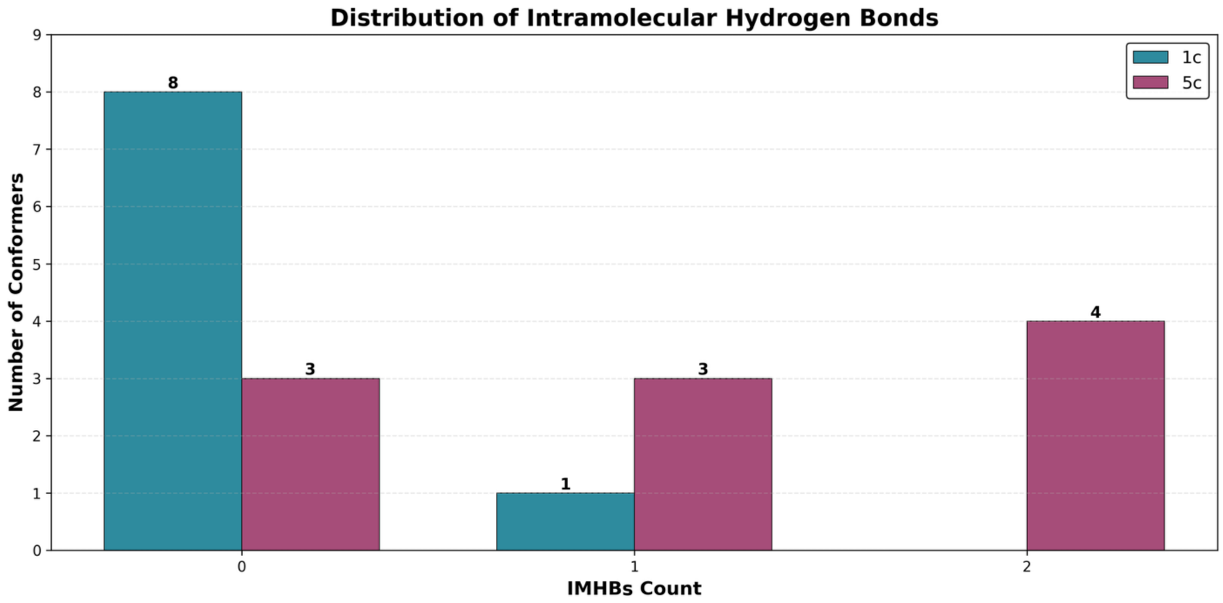

Figure S13. Distribution of intramolecular hydrogen bonds (IMHBs) for the conformations in the QM energy minimized in silico ensembles of **1c** and **5c**.

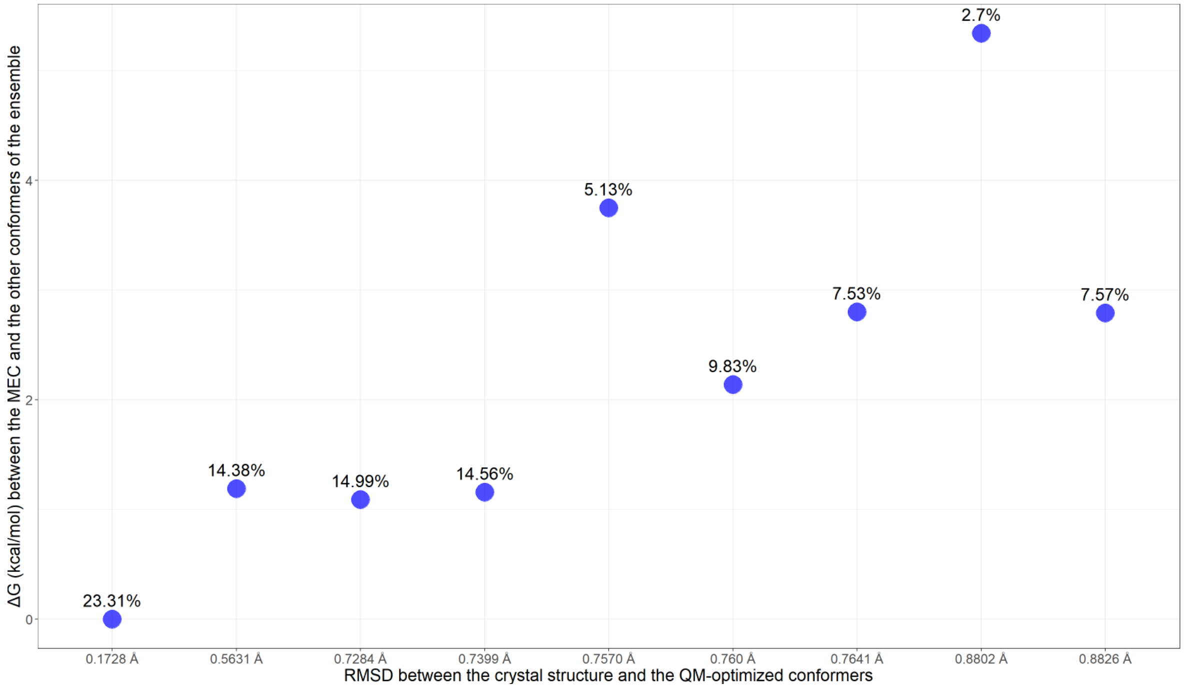

Figure S14. Difference in energy between the minimum energy conformation of **1c** and the other eight conformations in the *in silico* ensemble of **1c** plotted versus the similarity between each conformation and the crystal structure[1] of **1c**. The population (in %) of each conformation is given in the figure.

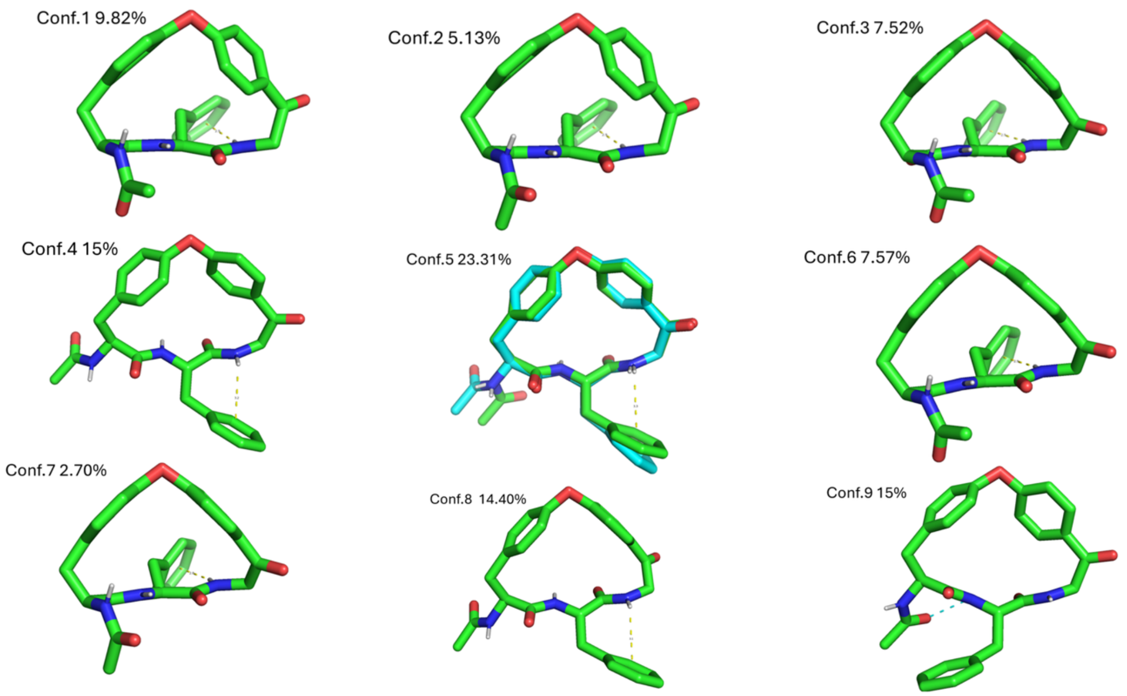

Figure S15. Conformations in the *in silico* ensemble of macrocycle **1c**, with populations given in %. Intramolecular interactions are shown with dotted lines.

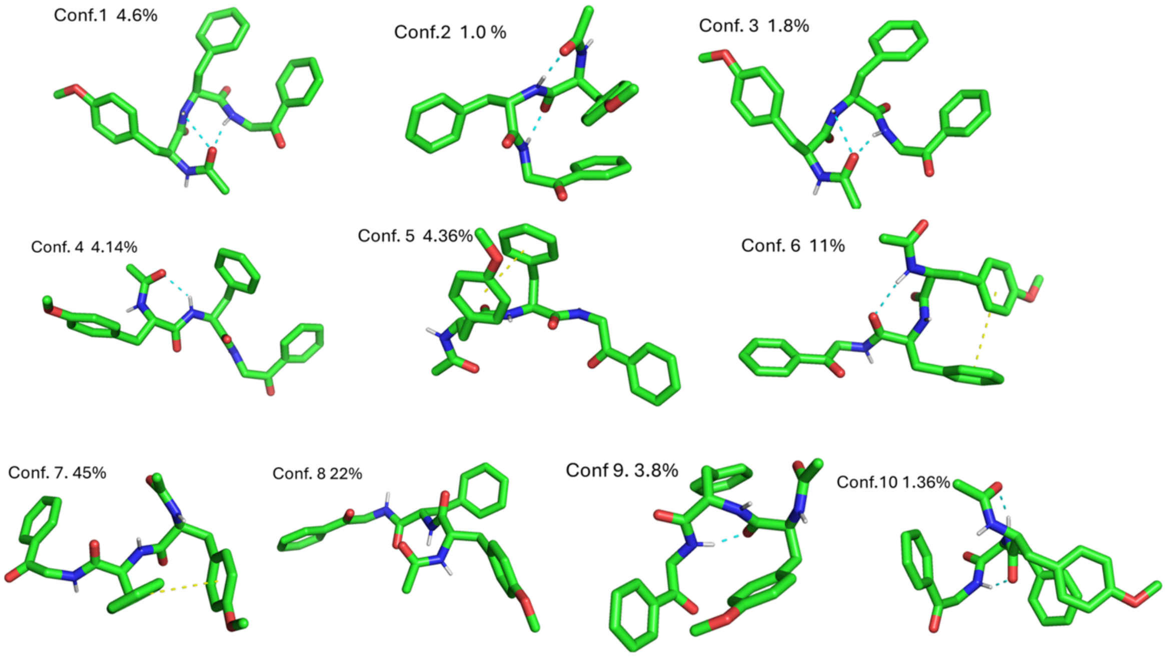

Figure S16. Conformations in the *in silico* ensemble of linear analogue **5c**, with populations given in %. Intramolecular interactions are shown with dotted lines.

References

1. Tyagi, M.; Poongavanam, V.; Lindhagen, M.; Pettersen, A.; Sjö, P.; Schiesser, S.; Kihlberg, J. Toward the Design of Molecular Chameleons: Flexible Shielding of an Amide Bond Enhances Macrocycle Cell Permeability. *Org. Lett.* **2018**, *20*, 5737−5742.
